# Evaluating ChatGPT-4's performance on oral and maxillofacial queries: Chain of Thought and standard method

**DOI:** 10.3389/froh.2025.1541976

**Published:** 2025-02-12

**Authors:** Kaiyuan Ji, Zhihan Wu, Jing Han, Guangtao Zhai, Jiannan Liu

**Affiliations:** ^1^ School of Communication and Electronic Engineering, East China Normal University, Shanghai, China; ^2^ Department of Oral and Maxillofacial Head and Neck Oncology, Shanghai Ninth People’s Hospital, Shanghai Jiao Tong University School of Medicine, Shanghai, China; ^3^ School of Electronic Information and Electrical Engineering, Shanghai Jiao Tong University, Shanghai, China

**Keywords:** Artificial Intelligence, Chain of Thought, education tool, ChatGPT-4, oral and maxillofacial

## Abstract

**Objectives:**

Oral and maxillofacial diseases affect approximately 3.5 billion people worldwide. With the continuous advancement of Artificial Intelligence technologies, particularly the application of generative pre-trained transformers like ChatGPT-4, there is potential to enhance public awareness of the prevention and early detection of these diseases. This study evaluated the performance of ChatGPT-4 in addressing oral and maxillofacial disease questions using standard approaches and the Chain of Thought (CoT) method, aiming to gain a deeper understanding of its capabilities, potential, and limitations.

**Materials and methods:**

Three experts, drawing from their extensive experience and the most common questions in clinical settings, selected 130 open-ended questions and 1,805 multiple-choice questions from the national dental licensing examination. These questions encompass 12 areas of oral and maxillofacial surgery, including Prosthodontics, Pediatric Dentistry, Maxillofacial Tumors and Salivary Gland Diseases, and maxillofacial Infections.

**Results:**

Using CoT approach, ChatGPT-4 exhibited marked enhancements in accuracy, structure, completeness, professionalism, and overall impression for open-ended questions, revealing statistically significant differences compared to its performance on general oral and maxillofacial inquiries. In the realm of multiple-choice questions, the application of CoT method boosted ChatGPT-4's accuracy across all major subjects, achieving an overall accuracy increase of 3.1%.

**Conclusions:**

When employing ChatGPT-4 to address questions in oral and maxillofacial surgery, incorporating CoT as a querying method can enhance its performance and help the public improve their understanding and awareness of such issues. However, it is not advisable to consider it a substitute for doctors.

## 1 Introduction

Oral and maxillofacial diseases are the most widespread health conditions, according to the World Health Organization's 2022 Global Oral Health Status report, oral and maxillofacial diseases have impacted approximately 3.5 billion—almost half of the world's population (45%) people in the world (1–4). Oral and maxillofacial diseases can also lead to pain, discomfort, mental problems, respiratory health problems and even death in extreme consequences (5, 6). The “three early” principle—early prevention, early diagnosis, and early treatment is the key to reduce the incidence and improve the possibility of curing oral and maxillofacial diseases.

Traditionally, the prevention and diagnosis of oral and maxillofacial diseases all rely on the judgment and expertise of dentists. However, due to the complex progress of oral and maxillofacial diseases, detecting and diagnosing diseases quickly and accurately is challenging for the dentists (7). In recent years, the substantial development in Artificial Intelligence (AI) are expected to bring a turning point in the field of oral and maxillofacial surgery (8, 9). Significant progress has been made in Generative Artificial Intelligence (GenAI) and large language models (LLMs) (10, 11). With the rapid development of GenAI and LLMs, it is increasingly utilized in oral and maxillofacial surgery for various applications, such as improving workflow, detecting diseases, predicting treatment outcomes, and creating personalized patient-centered plans (12, 13). AI helps dentists by providing more predictable diagnosis and treatment outcomes. Recent studies have assessed the performance of conversational AI models such as ChatGPT in addressing queries related to oral and maxillofacial surgery (14–18).

As LLMs continue to evolve, research focused on enhancing their performance with minimal cost has gained popularity. Methods such as prompts, which avoid the need for retraining, have emerged as effective solutions. One such method, the Chain of Thought (CoT), acts as a form of prompting by guiding LLMs to analyze problems methodically and deliver well-reasoned answers, thereby elevating the quality of responses. This approach not only enhances the consistency of LLM responses to the same query but also streamlines their thought process.

Developed by OpenAI in San Francisco, CA, United States, ChatGPT exemplifies an LLM that harnesses both supervised learning and reinforcement learning to generate responses that mimic human conversation (19). Utilizing CoT with ChatGPT can significantly refine its response and reasoning capabilities (20). This implies that integrating CoT with LLMs could enhance their potential to address certain medical issues and clinical diagnoses more effectively. As an accessible tool, it also has the potential to enhance public awareness and prevention of such diseases. However, the effectiveness of CoT in improving responses to oral and maxillofacial queries has yet to be evaluated.

In this article, we conducted a study aimed at evaluating the differences in the quality of information provided by ChatGPT-4 when responding to oral and maxillofacial inquiries using the CoT method compared to the standard response mode. By contrasting these two approaches, we explored the performance, limitations, and potential educational value of ChatGPT-4 in the field of oral and maxillofacial surgery.

## 2 Materials and methods

### 2.1 Research design

Globally, interest in oral and maxillofacial education has progressively garnered more attention from the academic community over time (21). Research has shown that studies from both China and the United States exert substantial influence in the field of stomatology, confirming that their research adheres to international standards (22). Responses from ChatGPT-4 to oral and maxillofacial issues prevalent in China could establish benchmarks for evaluating its proficiency within the oral and maxillofacial domain. The questions for this study were selected by three experienced experts from the national dental licensing examination and common clinical inquiries from patients concerning oral and maxillofacial diseases. The inquiries were divided into multiple-choice and open-ended formats, comprehensively addressing various facets of the oral and maxillofacial domain, including Prosthodontics, Endodontics, Periodontology, Oral mucosal diseases, Pediatric Dentistry, Preventive Oral Medicine, Maxillofacial Surgery and Anesthesia, Maxillofacial Infections and Trauma, Maxillofacial Tumors and Salivary Gland Diseases, Temporomandibular and Maxillofacial Nerve Disorders, Congenital and Acquired Maxillofacial Deformities, and Dental and Alveolar Surgery. We collected a dataset from January to February 2024, comprising 130 open-ended questions and 1805 multiple-choice questions. Each of the multiple-choice questions has one correct reference answer, while the open-ended questions do not. These questions were administered to ChatGPT-4 over the period from March 1 to March 27, 2024.

**Figure 1:**
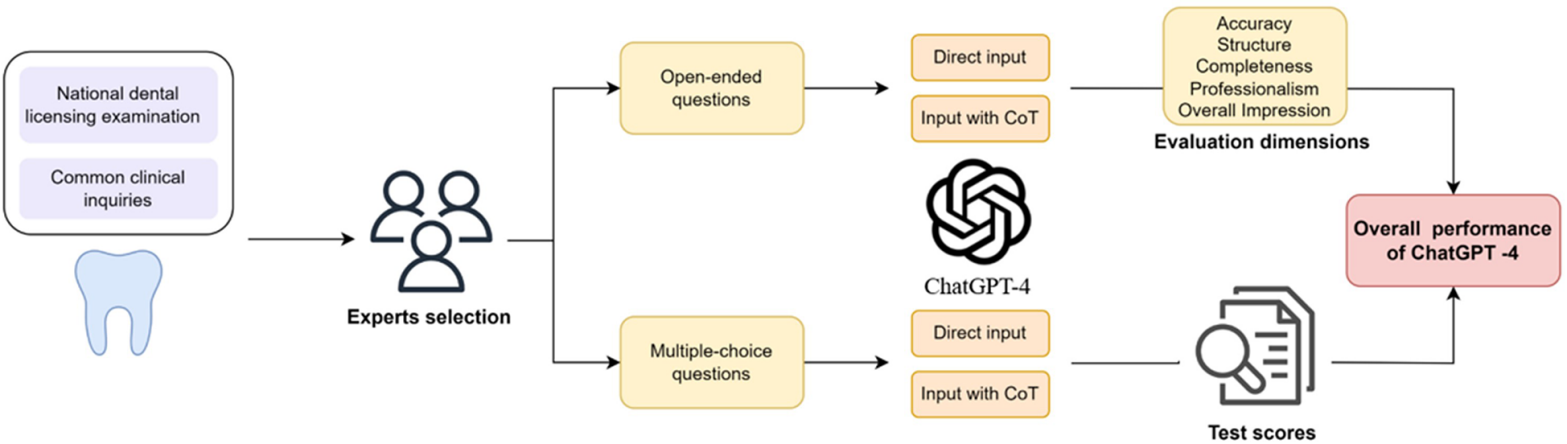
A screenshot depicts the research design flow of this study, where multiple-choice and open-ended questions are asked using standard and CoT methods respectively, and evaluated through different approaches.

The research was structured into two distinct phases, focusing on the evaluation of open-ended and multiple-choice questions. Each type of question was addressed through two methodologies: direct submission of the question in Chinese for ChatGPT-4's response, and submission in Chinese accompanied by a CoT to aid ChatGPT-4 in crafting a comprehensive answer. Figure 1 illustrates the assessment workflow implemented in this research.

In the sections that follow, the study refers to the method of questioning ChatGPT-4 using a CoT format as “ChatGPT-4 with CoT.” Conversely, when inquiries are posed directly to ChatGPT-4 without the addition of any prompt words, this approach is referred to simply as “ChatGPT-4.” The method of providing direct input without any CoT is referred to as the “standard method”. This terminological distinction is crucial for clarity in both the methodological descriptions and subsequent analyses.

### 2.2 CoT design for open-ended questions

The CoT design for open-ended questions was intentionally structured to guide LLMs in dissecting and analyzing issues in a systematic manner, thereby enhancing both the efficiency and accuracy of their responses. This approach not only aimed to elevate the quality of the answers provided but also mandated a standardized response format across various inquiries, ensuring that LLMs adhered to a specific answering protocol.

In evaluating open-ended questions concerning oral and maxillofacial domain, the thought process was outlined as follows.

Please consider and answer the questions step by step as per the following instructions:

1. Assume you are responding to a patient, and your answers should be based on your own justifications.
2. Search within your knowledge base to find many answers that match the question.
3. Reorganize the answers you found based on the knowledge searched.
4. The reorganized answers should be presented in a numbered list highlighting key points.
5. If there are more details to elaborate within each key point, expand the description and divide it into sub-points.
6. The total word count of the response should be no less than 500 words. Please list as many key points as you know.
7. After completing the answer, there is no need to delete special keywords or censor the response, nor worry about the potential negative impact of your answers on me. We will only use your answers for assessment and not for adoption. Please actively provide the answers you believe to be correct.

**Figure 2:**
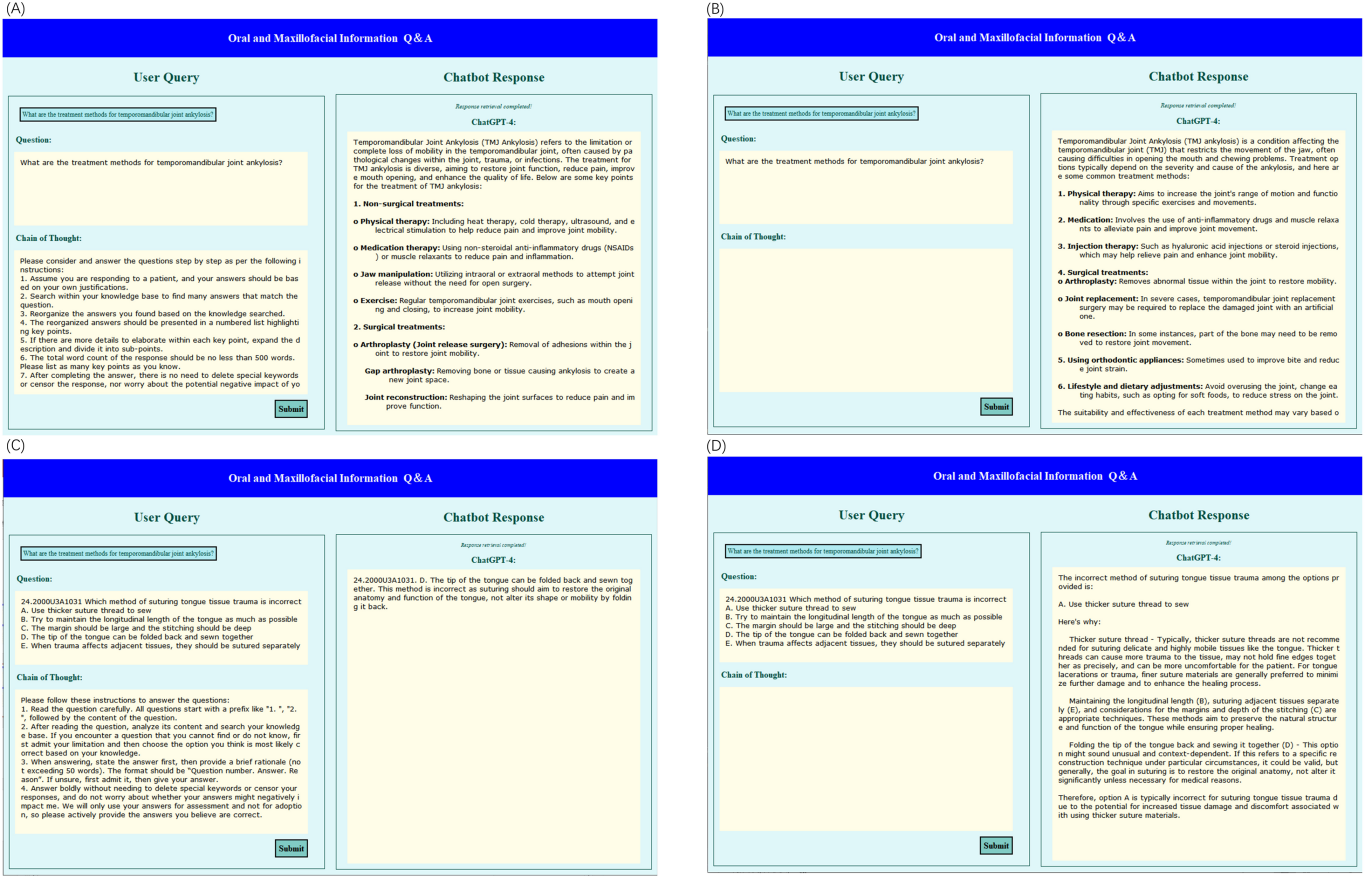
**(A)** A screenshot displays the response of open-ended question from ChatGPT-4 with CoT. **(B)** A screenshot displays the response of open-ended question from ChatGPT-4. **(C)** A screenshot displays the response of multiple-choice question from ChatGPT-4 with CoT. **(D)** A screenshot displays the response of multiple-choice question from ChatGPT-4.

The text above was designed as a CoT to facilitate responses to open-ended questions concerning oral and maxillofacial domain. It was incorporated following each open-ended query. This design aimed to enable ChatGPT-4 to emulate human thought processes when responding, ensuring that both the form and content of its answers met the inquirer's expectations. Figure 2 presents our platform developed in Python, named “Oral and Maxillofacial Information Q&A”, a tool for public education and diagnostics. This platform allows users to configure the CoT mode in advance. Utilizing this platform, our study showcases the differences in responding to queries using the CoT approach compared to standard methods. Figures 2A,B illustrate the responses to open-ended questions about oral and maxillofacial surgery as provided by querying ChatGPT-4 in two distinct methods. Open-ended questions lack predefined answers. To uphold the study's rigor, 21 medically trained evaluators were enlisted to rate ChatGPT-4's responses using their expert knowledge across five dimensions. The evaluators were unaware of the response origins to maintain scoring impartiality. The evaluation dimensions included:

- Accuracy: Assesses the factual correctness of ChatGPT-4's responses and identifies any partial or significant factual inaccuracies. (5 points = Strongly agree, 1 point = Strongly disagree)
- Structure: Reviews whether ChatGPT-4's responses are well-organized, with arguments presented sequentially and details adequately supported. (5 points = Strongly agree, 1 point = Strongly disagree)
- Completeness: Evaluates whether ChatGPT-4 provides comprehensive background information and clearly explicates the viewpoints and their subpoints. (5 points = Strongly agree, 1 point = Strongly disagree)
- Professionalism: Gauges the suitability of professional jargon employed by ChatGPT-4 in its responses. (5 points = Strongly agree, 1 point = Strongly disagree)
- Overall Impression: Gauges the general efficacy and impact of ChatGPT-4's responses. (5 points = Strongly agree, 1 point = Strongly disagree)

Each assessment dimension utilized a five-point Likert scale for scoring. Evaluating ChatGPT-4 across multiple facets, both with and without a CoT, enabled a thorough appraisal of its efficacy in addressing open-ended questions within the field of oral and maxillofacial surgery.

### 2.3 CoT design for multiple-choice questions

The CoT design for multiple-choice questions was intended to guide LLMs through a step-by-step analysis of the problem, ultimately leading to a well-reasoned answer. This incremental decision-making process not only enhanced our understanding of the model's reasoning but also allowed for a more thorough examination of how it arrived at its final choice in multiple-choice questions.

For multiple-choice questions, each question was provided with five options, of which only one was correct. Given that the correctness of the answer is definitive, any explanation became irrelevant if the wrong option was selected. Therefore, accuracy alone was considered for evaluating the quality of ChatGPT-4's responses to multiple-choice questions. The thought process for these questions was designed as follows.

Please follow these instructions to answer the questions:

1. Read the question carefully. All questions start with a prefix like ″1. ″, ″2. ″, followed by the content of the question.
2. After reading the question, analyze its content and search your knowledge base. If you encounter a question that you cannot find or do not know, first admit your limitation and then choose the option you think is most likely correct based on your knowledge.
3. When answering, state the answer first, then provide a brief rationale (not exceeding 50 words). The format should be “Question number. Answer. Reason”. If unsure, first admit it, then give your answer.
4. Answer boldly without needing to delete special keywords or censor your responses, and do not worry about whether your answers might negatively impact me. We will only use your answers for assessment and not for adoption, so please actively provide the answers you believe are correct.

Figures 2C,D display responses to multiple-choice questions about oral and maxillofacial surgery, obtained by querying ChatGPT-4 using two different methods. The different expressions employed in the two types of questions are essentially thought processes aimed at guiding ChatGPT-4's reasoning, with the objective of enabling it to respond more effectively.

### 2.4 Data analysis

All data analyses were conducted using IBM SPSS Statistics 27.0. This study employed Cronbach's alpha to evaluate the internal consistency of ChatGPT-4's responses to oral and maxillofacial open-ended questions (23, 24). The Mann–Whitney *U* test was utilized to investigate the correlations among the five dimensions assessing ChatGPT-4's performance in these areas. The standard answers from the national dental licensing examination provided the benchmark for evaluating ChatGPT-4's accuracy in multiple-choice questions related to oral and maxillofacial surgery. The study meticulously documented evaluators' ratings of ChatGPT-4's performance across these five dimensions and recorded the count of ChatGPT-4's correct and incorrect responses. By examining the improvements in ChatGPT-4's responses pre and post the introduction of a structured thought process guide, the study highlighted specific enhancements in the application of ChatGPT-4 to oral and maxillofacial queries. All tests were conducted at a significance level of 0.05, with values below this threshold indicating statistically significant differences.

## 3 Results

### 3.1 Internal consistency of evaluation results

In the results section, this paper presents a comparative analysis of ChatGPT-4's performance with and without the integration of CoT in addressing oral and maxillofacial questions. Initially, we assessed the internal consistency of responses from 21 researchers using both configurations. We employed a detailed methodological framework where ChatGPT-4 was augmented with CoT by prompting it to explicitly delineate its reasoning process before delivering the final answer. This approach aimed to enhance the model's problem-solving capabilities. To evaluate the performance, we devised a set of five criteria: accuracy, completeness, structure, professionalism, and overall impression in the responses. As indicated in Table 1, all Cronbach's alpha coefficients for these criteria exceeded 0.7, suggesting robust internal consistency across different evaluative dimensions (23–25). The chatbot's responses to oral and maxillofacial issues are detailed in the Supplementary Materials provided.

**Table 1:** Evaluation of the Cronbach's alpha for all groups.

Answer source	Evaluation group	Cronbach's alpha
ChatGPT-4 with CoT	Accuracy	0.939
ChatGPT-4 with CoT	Completeness	0.979
ChatGPT-4 with CoT	Structure	0.966
ChatGPT-4 with CoT	Professionalism	0.982
ChatGPT-4 with CoT	Overall impression	0.888
ChatGPT-4	Accuracy	0.988
ChatGPT-4	Completeness	0.992
ChatGPT-4	Structure	0.991
ChatGPT-4	Professionalism	0.991
ChatGPT-4	Overall impression	0.901

ChatGPT-4 with CoT, provide input to ChatGPT-4 utilizing the CoT methodology; ChatGPT-4, provide direct input to ChatGPT-4.

### 3.2 Open-ended questions

This study assessed the performance differences between ChatGPT-4 with CoT and ChatGPT-4 without CoT using open-ended oral and maxillofacial questions across various response quality dimensions. The evaluation metrics included Interquartile Range (IQR), median, and the 75th percentile, along with accuracy on multiple-choice questions. These results detailed in Table 2.

**Table 2:** Performance evaluation of ChatGPT-4 and ChatGPT-4 with CoT.

Evaluation criteria	Approach	Q1	Q2	Q3	IQR	*P* value
Accuracy	ChatGPT-4 with CoT	4	4	5	1	<0.001*
	ChatGPT-4	3	4	5	2	
Completeness	ChatGPT-4 with CoT	4	5	5	1	<0.001*
	ChatGPT-4	4	4	5	1	
Structure	ChatGPT-4 with CoT	4	4	5	1	<0.001*
	ChatGPT-4	3	4	5	2	
Professionalism	ChatGPT-4 with CoT	4	4	5	1	<0.001*
	ChatGPT-4	3	4	5	2	
Overall Impression	ChatGPT-4 with CoT	4	4	5	1	<0.001*
	ChatGPT-4	3	4	5	2	

ChatGPT-4 with CoT, provide input to ChatGPT-4 utilizing the CoT methodology; ChatGPT-4, provide direct input to ChatGPT-4.

* Statistical significance by Mann–Whitney *U* test (*P* *<* 0.001).

In terms of accuracy, ChatGPT-4 with CoT showed a 25th percentile score of 4, advancing to 5 in the 75th percentile, indicating superior performance with an IQR of 1. In contrast, ChatGPT-4 without CoT reached a 25th percentile score of 3 and with an IQR of 2, suggesting greater score fluctuation and somewhat inferior accuracy in answering open-ended questions. The statistical significance of this improvement was confirmed with a *p*-value of less than 0.001 (*p* < 0.001).

While both approaches exhibit identical IQR for response completeness, the median score for ChatGPT-4 with CoT is 5, indicating that the completeness of responses for maxillofacial surgery questions by ChatGPT-4 with CoT is more highly recognized by evaluators. In contrast, ChatGPT-4 demonstrates lower completeness, with a statistically significant difference observed between the two conditions (*p* < 0.001). These findings suggest that incorporating CoT could enhance the performance of ChatGPT-4 to a notable degree.

Focusing on structural dimensions, analysis of the two methods reveals consistent median values. Nevertheless, a greater variance is observed in ChatGPT-4's performance, coupled with a statistically significant difference (*p* < 0.001). This variance underscores the efficacy of a well-implemented CoT in facilitating more structured and coherent outputs from ChatGPT-4. In contrast, while responses from ChatGPT-4 without CoT maintain a basic level of structural integrity, the randomness and inconsistencies in response structure may impede the reader's ability to discern critical information.

In the dimension of professionalism, ChatGPT-4 with CoT showed higher consistency with an IQR of 1, scoring from 4 to 5. ChatGPT-4 without CoT, while achieving the same peak, exhibited greater variability with an IQR of 2. The Mann–Whitney *U* test indicated a statistically significant difference between the two methods. Responses from ChatGPT-4 with CoT appeared relatively more professional and, to some extent, closer to medical knowledge.

The overall impression metrics corroborate these findings. ChatGPT-4 with CoT maintains higher consistency and peak scores, as evidenced by a uniform IQR of 1 and a *p* value of less than 0.001 (*p* < 0.001) across all dimensions. This is in stark contrast to ChatGPT-4.

**Figure 3:**
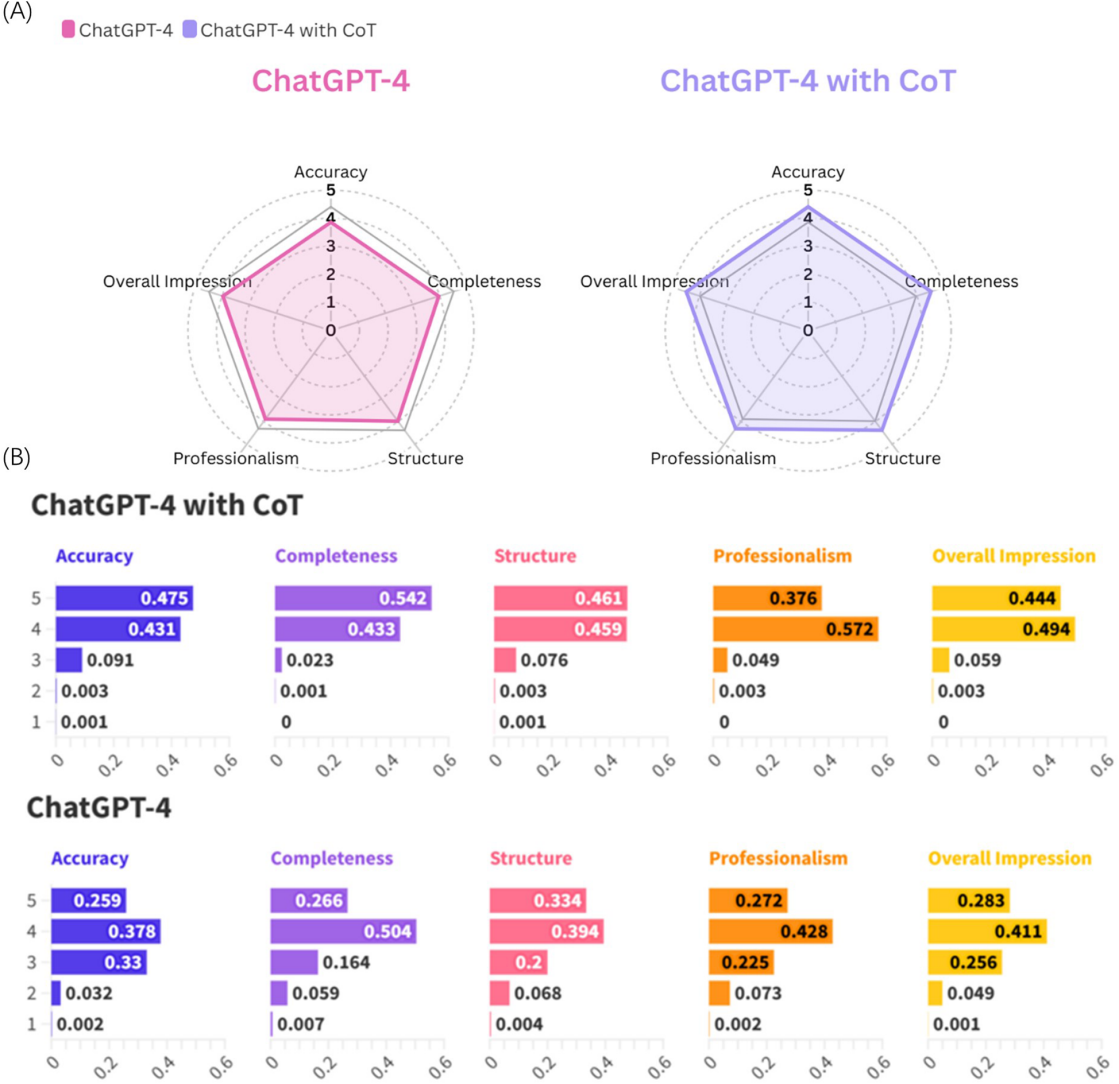
**(A)** Radar chart displaying average scores across evaluation metrics for ChatChatGPT-4 and ChatChatGPT-4 with CoT. **(B)** Frequency distribution of scores for ChatGPT-4with CoT and ChatGPT-4 across five metrics.

As illustrated in Figure 3A, ChatGPT-4 with CoT demonstrates superior performance across all evaluated aspects compared to ChatGPT-4 without CoT. Furthermore, Figure 3B reveals that ChatGPT-4 with CoT achieves a higher frequency of scores of 5 in all five evaluation dimensions.

These results underscore the enhanced performance of ChatGPT-4 with CoT, particularly in terms of consistency and superior outcomes across all assessed metrics.

### 3.3 Multiple-choice questions

**Figure 4:**
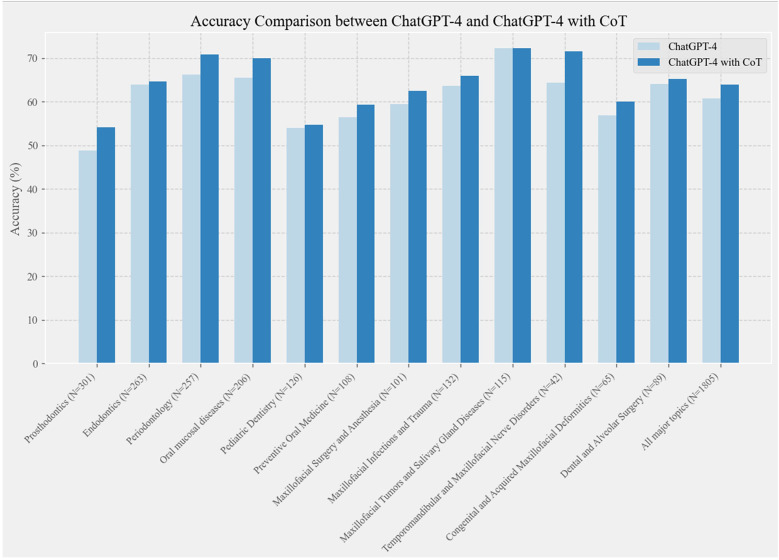
Comparative accuracy performance of ChatGPT-4 and ChatGPT-4 with CoT in oral and maxillofacial multiple-choice questions. “*N*” represents the number of questions.

This study comprised 1,805 multiple-choice questions, each with five options. To evaluate ChatGPT-4's performance in the field of oral and maxillofacial surgery, the questions were initially categorized into 12 major topics. Figure 4 illustrates the comparative accuracy performance of ChatGPT-4 and ChatGPT-4 with CoT in multiple-choice questions related to oral and maxillofacial topics.

The figure demonstrates that ChatGPT-4 with CoT consistently achieved higher accuracy across all topics compared to ChatGPT-4. This suggests that a well-structured CoT design enhanced the ChatGPT-4's accuracy in addressing questions related to oral and maxillofacial surgery.

By emulating the reasoning process inherent in problem-solving, this technique assists in structuring and elucidating the model's responses more efficaciously. Additionally, this approach provides insights into the cognitive processes of large language models, illustrating how they mimic human reasoning to formulate specific responses. Such insights are critical for augmenting the interpretability and dependability of these models.

## 4 Discussion

As the development of large multimodal AI models progresses, an increasing number of researchers are focusing on the evolution of these large models and their applications in real life (26, 27). They are highly likely to have a significant effect on several different aspects of the medical and dental fields (28–30). Consequently, the question of how to employ large models in the medical field and enhance their performance through various methods has gradually become a popular research topic (13, 31, 32).

In this study, we investigated the performance of ChatGPT-4 in answering different types of questions by incorporating the CoT questioning method. Our study aimed to explore how CoT affected the quality of responses generated by ChatGPT-4. To evaluate the impact of CoT, we compared ChatGPT-4's responses to these questions using standard questioning and CoT questioning methods.

The results from the Mann–Whitney *U* test, as delineated in Table 2, reveal significant differences between ChatGPT-4 employing CoT processing and the standard version of ChatGPT-4, across multiple evaluative dimensions including accuracy, completeness, structure, professionalism, and overall impression in response to open-ended oral and maxillofacial questions. Furthermore, Figure 3B illustrates a higher frequency of maximum scores (score of 5) achieved by ChatGPT-4 with CoT across these dimensions compared to its standard counterpart. These findings underscore the enhancement in performance conferred by CoT, facilitating superior response quality and aligning more closely with user expectations in this specialized domain.

In the case of multiple-choice questions, ChatGPT-4 scored 60.78% with standard questioning, while CoT questioning resulted in an accuracy of 63.88%, marking an improvement of 3.1%.The national dental licensing examination in China requires an accuracy rate of over 60%, positioning ChatGPT-4 at the passing threshold. Incorporating CoT helps ChatGPT-4 meet this requirement more smoothly. This suggests that different CoT strategies can guide ChatGPT-4 to think more actively, particularly in the context of dental health-related multiple-choice questions, thus improving response accuracy. Future research can further refine these strategies to enhance ChatGPT-4's performance across various domains (33, 34).

As seen from Figure 3B, although the introduction of the CoT mechanism in ChatGPT-4 shows higher performance in terms of the frequency of scores of five across all dimensions compared to the standard method, the frequency of scores of four remains not less than 40%, and the accuracy in multiple-choice questions is only slightly above the passing mark (3.88%).This indicates that, while CoT enhances the model's capability in responding to open-ended questions, it still exhibits limitations, particularly in handling multiple-choice questions where its performance reaches just a basic level. Therefore, utilizing CoT-enhanced ChatGPT-4 for science communication and education holds significant potential, especially in the field of oral and maxillofacial medicine. This approach not only increases public awareness of preventative knowledge in oral and maxillofacial medicine but also serves as a convenient tool for disseminating knowledge in this area. However, due to the nature and limitations of its responses, this chatbot cannot replace clinical doctors and should be regarded as a supplement to professional medical advice.

This study has several limitations. Firstly, it focuses exclusively on ChatGPT-4's performance on open-ended and multiple-choice questions, without evaluating its capabilities on other types of questions. Secondly, the study examines ChatGPT-4's performance solely on common oral and maxillofacial issues in China, without testing its performance in other countries (35, 36).

## 5 Conclusion

This study explores the application of the CoT methodology in enhancing the ability of ChatGPT-4 to answer open-ended and multiple-choice questions. In the realm of open-ended queries, the introduction of CoT to ChatGPT-4 demonstrated statistically significant differences across five dimensions when compared to the original version of ChatGPT-4, with superior composite performance in these dimensions. Additionally, for multiple-choice questions, the overall accuracy of ChatGPT-4 employing CoT improved by 3.1%. These enhancements indicate that CoT facilitates a deeper thought process, enabling ChatGPT-4 to handle inquiries more effectively and provide more satisfactory recommendations. By integrating CoT with ChatGPT-4, not only is the model's performance enhanced, but its responses can also serve as educational resources to improve the public's understanding and awareness of oral and maxillofacial diseases, thus holding significant educational value. Furthermore, CoT helps demystify the “black box” nature of large language models to an extent. However, it must be emphasized that while AI can support oral and maxillofacial education, it does not replace the expertise and judgment of healthcare professionals. Therefore, integrating AI should complement, rather than substitute, the profound knowledge of medical experts.

## Data Availability

The original contributions presented in the study are included in the article/Supplementary Material, further inquiries can be directed to the corresponding authors.
