# Supplementary material for: Evaluating ChatGPT-4's performance on oral and maxillofacial queries: Chain of Thought and standard method
Source: Front Oral Health. 2025 Feb 12;6:1541976. doi: 10.3389/froh.2025.1541976 (PMC11860867; doi:10.3389/froh.2025.1541976)
Supplement: Supplementary file 1 [file Table1.docx]

Supplementary Material

# Supplementary Data

## Querying GPT-4 with Chain of Thought (Open-ended Questions)

1. **Endodontics-related problems**
2. What are the common symptoms of endodontic disease?

Endodontic disease, commonly known as a disease of the structure and pulp (the soft tissue inside the teeth), involves a range of problems, from minor tooth damage to severe pulp infection. Understanding its common symptoms is essential for early diagnosis and treatment. The following information is based on an existing knowledge base search and collation to provide patients with a comprehensive overview.

1. **pain**
   - **Persistent pain**: A telltale sign of endodontic disease is a persistent toothache, which may be dull or severe.
     - **Spontaneous pain**: Pain may occur spontaneously for no apparent reason and does not require external stimulation.
     - **Nocturnal pain**: Especially at night, when the body is in a recumbent position, the pain may be exacerbated.
   - **Increased sensitivity to heat or cold**: The tooth takes longer to respond to hot and cold stimuli, and pain may persist after the stimulus is removed.
     - **Heat sensitivity**: The affected tooth reacts strongly to hot food or drink.
     - **Cold sensitivity**: A reaction to a cold stimulus, such as pain triggered when drinking cold water or inhaling cold air.
2. **Color change**
   - The tooth color may become dark or discolored, which is a sign that the pulp blood vessels are damaged or dead.
     - **Gray or black**: The tooth may appear abnormally gray or black, indicating that the blood supply to the pulp has been cut off.
3. **swelling**
   - **Swollen gums**: The gums around the teeth may be swollen, which can be a sign of pulpitis or periapical inflammation.
     - **Swelling can be accompanied by pain**: Swollen gums are often accompanied by tenderness.
     - **Abscess formation**: Abscesses may form in the apical area of the tooth and appear as protrusions on the gums, which may be accompanied by pus discharge.
4. **Loose teeth**
   - Teeth may become loose, possibly due to alveolar bone loss due to pulpitis.
5. **Difficulty or pain in chewing**
   - Teeth may experience pain or discomfort when chewing, especially when food pressure is applied to the damaged tooth.
6. **Difficulty opening**
   - If pulpitis causes swelling of the face, it may affect the opening and closing of the mouth, making it difficult to open.
7. **fever**
   - Pulp infections can sometimes cause localized or generalized fever, although this is not the most common symptom.

Each symptom needs to be diagnosed by a professional dental examination, including a visual examination of the teeth, a tactile examination, a knock test on the teeth, and X-rays if necessary. If you experience any of the above symptoms, you should seek help from a dental professional as soon as possible to avoid worsening your condition. Untreated endodontics can lead to more serious oral health problems, including tooth loss and the further spread of infection. The right treatment may include removal of the diseased pulp, root canal treatment, or extraction of the tooth if necessary, and subsequent restoration work, such as crown overlay, to restore the function and appearance of the tooth.

1. How can I relieve toothache caused by endodontics?

Toothache caused by endodontics can be very intense and requires prompt treatment to relieve the pain. Here are some of the key points for toothache relief, and they are based on extensive medical knowledge and practical experience:

1. **Immediate Action**:
   - **1.1 Cold compress**: Gently apply an ice pack to the outside of the cheek to the painful tooth area for 10-15 minutes each time. This can help reduce inflammation and pain.
   - **1.2 Avoid irritating foods**: Avoid excessively hot, cold, sweet, or spicy foods, as these may exacerbate toothache.
   - **1.3 Temporarily avoid chewing on the side of the sore tooth**: Reducing pressure on the diseased tooth can help reduce pain.
2. **Medications**:
   - **2.1 Over-the-counter pain relievers**: Take over-the-counter pain relievers, such as ibuprofen or acetaminophen, to provide temporary pain relief. Be careful to follow the recommended dosage on the package and do not overdose.
   - **2.2 Oral local anesthetics**: Temporary pain relief can be provided with oral local anesthetic sprays or gels containing ingredients such as benzocaine.
3. **Oral Hygiene Maintenance**:
   - **3.1 Brush and floss gently**: Maintain oral hygiene by brushing and flossing gently to avoid touching pain points.
   - **3.2 Salt water gargle**: Gargling with warm salt water can help reduce pain and reduce inflammation several times a day.
4. **Seek medical attention**:
   - **4.1 Make an appointment with a dentist as soon as possible**: Endodontic diseases often require professional treatment, such as root canal treatment or possible nerve extraction of the tooth, so it is necessary to make an appointment with a dentist as soon as possible.
   - **4.2 Emergency**: If the pain is unbearable, or if it is accompanied by symptoms such as swelling of the face, fever, etc., emergency medical help should be sought.
5. **Long-term precautions**:
   - **5.1 Regular dental check-ups**: Regular dental check-ups can detect and treat potential dental and pulp problems early.
   - **5.2 Healthy eating habits**: Reduce sugar intake and avoid acidic drinks and foods to reduce the risk of tooth erosion and disease.
   - **5.3 Enhance oral hygiene**: Maintain good oral hygiene habits with proper brushing and flossing techniques to reduce the accumulation of plaque and tartar.

Toothache due to endodontic disease requires a comprehensive approach. When taking any self-mitigation measures, the most important thing is to realize that these can only provide temporary relief. Addressing the root cause of the problem usually requires evaluation and treatment by a dental professional. Therefore, seeking medical attention in a timely manner and obtaining a professional diagnosis and treatment plan is the key to solving the problem of toothache. In addition, through daily oral hygiene maintenance and regular dental check-ups, the occurrence of endodontic disease can be effectively prevented, so as to avoid the related toothache problem.

1. Do endodontic patients have to have root canal treatment?

Root canal treatment is a common treatment in dentistry to address pulp lesions or infections. Endodontic disease is a condition in which the hard tissues of the teeth are damaged and the pulp (the soft tissue inside the teeth) becomes inflamed or infected. Not all patients with endodontics must undergo root canal treatment, depending on the severity and specifics of the condition. Here are the key points explained in detail:

1. **Stages of pulpitis**:
   - Incipient pulpitis: The pulp is in a reversible inflammatory state and may not require root canal treatment, which can be promoted by removing the decay and placing medications.
   - Advanced pulpitis: pulp inflammation may be irreversible at this point, but conservative treatment can sometimes be tried in the absence of bacterial infection.
   - Advanced pulpitis or necrosis: The pulp has died or there is a bacterial infection, which usually requires root canal treatment.
2. **Degree of symptoms**:
   - Mild symptoms: Root canal treatment may not be necessary, such as increased sensitivity or mild reactions to hot and cold stimuli.
   - Moderate to severe symptoms: such as persistent pain, nocturnal pain, bite, usually indicate the need for root canal treatment.
3. **Imaging tests**:
   - X-rays: can show the hard tissue of the teeth and the bone around the roots.
   - CBCT (Cone Beam Computed Tomography): Used in complex cases, it provides three-dimensional images to help evaluate pulp lesions.
4. **Choice of treatment options**:
   - Conservative treatment: When pulp inflammation is reversible, methods such as direct or indirect pulp capping can be chosen.
   - Root canal treatment: In cases where the pulp is irreversible or has necrotic, the diseased pulp is removed and the root canal system is cleaned and filled.
5. **Steps of root canal treatment**:
   - Removal of diseased pulp tissue.
   - Root canal formation: The root canal is shaped using a special tool.
   - Perfusion disinfection: Clean the root canal system with an antimicrobial solution.
   - Root canal filling: Root canal filling material is used to fill the root canal to prevent bacteria from re-invading.
   - Composite filling or crown restoration: to ensure the function and aesthetics of the tooth.
6. **Possible alternative treatments**:
   - Antibiotic therapy: In some cases, antibiotics are initially used to control the infection.
   - Endodonticectomy: A method of partial removal of the pulp of a tooth to preserve the pulp of an immature tooth.
   - Tooth extraction: Tooth extraction may be an option when the tooth cannot be preserved or the patient is unwilling to undergo root canal treatment.
7. **Precautions after treatment**:
   - Avoid chewing hard objects: Teeth may be weaker after root canal treatment.
   - Regular check-ups: Monitor the effectiveness of your treatment and the health of your teeth.
   - Timely restoration: If needed, a crown restoration should be performed in a timely manner to enhance the strength of the tooth.
8. **Individual Differences in Patients**:
   - Age, health status, and oral hygiene habits can all influence treatment choices.
   - Economic considerations and patient preferences are also part of the decision.

In summary, whether endodontic patients must undergo root canal treatment needs to consider the severity of the disease, symptoms, imaging examination results, treatment options, and the patient's individual situation. It is advisable to develop a personalized treatment plan according to the specific situation of the patient under the guidance of a professional dentist.

1. What are the symptoms of pulpitis?

Pulpitis is inflammation of the pulp (the soft tissue inside the tooth), usually caused by a bacterial infection, which can be due to tooth decay, cracked teeth, or a hard blow to the tooth. Here is a list of symptoms of pulpitis based on my knowledge base search and integration:

1. **pain**
   - **Acute pain**: Pulpitis may cause sudden, acute pain that can be intense and may worsen at night.
     - **Spontaneous pain**: The pain of pulpitis can occur suddenly without any apparent trigger.
     - **Persistent pain**: Pain may be constant and not relieved by regular pain relief measures, such as taking over-the-counter pain relievers.
   - **Reactive pain**: Pulpitis can cause the tooth to be particularly sensitive to hot and cold stimuli, and the pain may persist for some time even after the stimulus has cleared up.
2. **Discoloration of teeth**
   - Pulpitis may cause the teeth to darken in color, and this is because the pulp tissue is damaged or dies, which can cause changes in the blood composition inside the teeth.
3. **swelling**
   - **Swollen gums**: Pulpitis may be accompanied by swelling of the gums at the intervals, which is usually due to an inflammatory response caused by an infection.
   - **Swelling of the face**: In severe cases, the infection may spread to the soft tissues of the face, causing swelling of the face or jaw.
4. **Swollen lymph nodes**
   - Pulpitis can cause swelling of adjacent lymph nodes (near the lower jaw angle), which is the body's immune response to infection.
5. **Fever and general malaise**
   - In some cases, pulpitis may cause a fever, indicating that the body is battling an infection.
   - Infections can cause general malaise or fatigue because the body's immune system is actively fighting the infection.
6. **Loose teeth**
   - If pulpitis is left untreated, it can lead to bone destruction around the teeth, which can loosen the teeth.
7. **Difficulty chewing**
   - Pain and tooth sensitivity may cause the patient to experience discomfort or pain while chewing, resulting in difficulty chewing.
8. **Bad breath or foul smell**
   - Infection can lead to unpleasant odors in the mouth, which may be due to tissue necrosis and bacterial activity caused by the infection.
9. **Teeth are sensitive to occlusal pressure**
   - Patients may find discomfort when biting or touching their teeth, which may be due to increased pressure on the root or surrounding tissues of the tooth due to inflammation.
10. **headache**
    - Pulpitis can sometimes cause headaches, especially if the infection affects the upper teeth, which can cause frontal or temporal pain.

Symptoms of pulpitis can vary from person to person, and not all symptoms will occur in every patient. If you think you may have pulpitis, you should seek help from a dentist as soon as possible. If left untreated, pulpitis can lead to more serious problems, including pulp necrosis, apical periodontitis, tooth loss, and even more serious systemic infections. Dentists usually diagnose pulpitis through clinical examination and x-rays and, depending on the situation, recommend appropriate treatments such as medication, root canal treatment, or in some cases, tooth extraction.

1. What is Root Canal Treatment?

Root canal treatment, often referred to as root canal treatment or root canal filling, is a tooth-preserving treatment used to treat infected or damaged pulp (the soft tissue inside the tooth). This treatment saves and preserves a damaged or infected tooth, avoiding tooth extraction. The following are the key points of root canal treatment, in order of serial number:

1. **The necessity and purpose of root canal treatment**
   - 1.1 When the pulp of a tooth is infected or severely damaged, it can lead to more serious dental problems, including tooth loss, if left untreated.
   - 1.2 The purpose of root canal treatment is to remove the infected tissue from the pulp cavity, prevent further spread of infection, and preserve the structure of the tooth.
2. **The course of treatment**
   - 2.1 **Diagnosis and examination**: The dentist will examine the condition of the teeth and surrounding bones through X-rays to confirm the need for root canal treatment.
   - 2.2 **Anesthesia and pulp opening**: Before the treatment begins, the dentist will use a local anesthetic to numb the affected tooth and then make a small hole into the pulp cavity.
   - 2.3 **Cleaning and disinfection**: Use small tools to remove infected or dead pulp tissue, and thoroughly clean the pulp cavity and root canal.
   - 2.4 **Filling and sealing**: After cleaning, the root canal is filled with a biocompatible material (such as guta rubber), and then the opening is closed to prevent bacteria from re-entering.
   - 2.5 **Restoration and crowning**: After root canal treatment, the tooth may need further restoration, such as crowning, to restore the shape, function and aesthetics of the tooth.
3. **Precautions after treatment**
   - 3.1 **Temporary discomfort**: There may be transient tooth sensitivity or discomfort after treatment, which can usually be managed with over-the-counter pain relievers.
   - 3.2 **Oral hygiene**: Maintain good oral hygiene habits, brush and floss regularly, and have regular check-ups to maintain the effectiveness of the treatment.
4. **Effects and risks of treatment**
   - 4.1 **Success rate**: Root canal treatment has a high success rate, and most root canal treated teeth can be retained for a long time.
   - 4.2 **Potential Risks**: Although rare, problems that may be encountered with treatment include the failure of the treatment to completely remove the infection, or the slight displacement of the filling material over time.
5. **Cost and duration of treatment**
   - 5.1 **Cost**: The cost of root canal treatment varies depending on the region, the location of the tooth and the dentist's fees, and is usually an investment to avoid the higher cost of tooth replacement.
   - 5.2 **Duration**: Treatment usually requires one to three visits to the dentist's office, each visit may last 1-2 hours, depending on the complexity of the treatment.
6. **Preparation before treatment and recovery after treatment**
   - 6.1 **Preparation**: Before treatment, your dentist may recommend avoiding hard or sticky foods to reduce additional pressure on the affected tooth.
   - 6.2 **Recovery**: Immediately after treatment, chewing hard foods should be avoided until the teeth are fully recovered, as well as following up with any follow-up examinations recommended by the dentist.

Root canal treatment is an effective way to save damaged or infected teeth, avoid tooth extraction, and maintain oral health. If you have tooth pain or discomfort, you should consult your dentist as soon as possible to determine if root canal treatment is needed.

1. What do I need to pay attention to after root canal treatment?

Root canal treatment (also known as root canal treatment or root canal filling) is a common dental procedure designed to treat the inside of an infected or damaged tooth. This procedure can effectively save the tooth and avoid tooth extraction. After completing root canal treatment, patients need to pay special attention to a few things to ensure the success of the treatment and the long-term health of the teeth. The following is a detailed explanation of what I need to pay attention to after root canal treatment based on what I know:

1. **Short-term care**
   - **Avoid chewing**
     - Chew food with your treated tooth until the anesthesia is completely gone to avoid biting yourself due to tooth sensitivity or numbness.
   - **Medication management**
     - Use pain medication as directed by your dentist. If antibiotics are prescribed, make sure to complete the entire course on time and do not stop taking them even if symptoms disappear early.
   - **Protection of temporary fillings**
     - Avoid chewing hard or sticky foods on the treated side to prevent the temporary filling from falling off.
2. **Long-term care**
   - **Good oral hygiene**
     - Brush your teeth twice a day and floss between your teeth to keep your mouth clean and prevent new infections.
   - **Review regularly**
     - After completing root canal treatment, you should return to your dentist regularly for x-rays and necessary follow-ups to ensure that there are no further infections or problems in the treated area.
   - **Consideration for wearing braces**
     - Depending on your dentist's advice, braces (crowns) may need to be fitted to the treated tooth to increase the strength and longevity of the tooth.
3. **Dietary advice**
   - **pap**
     - At the beginning of treatment, choose soft foods and foods that are easy to chew to avoid putting extra pressure on your teeth.
   - **Avoid extremely hot or cold foods**
     - Avoid foods that are too hot or too cold as your teeth may be sensitive after treatment.
4. **Pay attention to the symptoms**
   - **Pain management**
     - Mild to moderate pain is normal for a few days after treatment, but if the pain persists or worsens, a dentist should be contacted.
   - **Observe the swelling**
     - If there is abnormal swelling in the treated area or face, you should contact your dentist immediately.
5. **Avoid bad habits**
   - **Don't smoke**
     - Smoking slows down the healing process in the mouth and increases the risk of infection.
   - **Limit alcohol consumption**
     - Alcohol may interfere with the healing process of your teeth.

From the above elaboration, we can see that the care after root canal treatment includes not only short-term considerations such as chewing avoidance, medication management, and protection of temporary fillings, but also long-term care such as maintaining good oral hygiene, regular check-ups, and possible braces (crowns) installation. Dietary advice and observation of symptoms are also very important to ensure maximum treatment and avoid possible complications. Finally, lifestyle changes, such as quitting smoking and limiting alcohol consumption, are equally important to speed up the healing process and maintain oral health. Following these guidelines can help ensure optimal recovery and long-term health of your teeth after root canal treatment.

1. What should I do if my root canal treatment fails?

When root canal treatment fails, you may feel worried and unsure as a patient about how to proceed with the treatment. Here's the answers, which I searched and reassembled based on my knowledge base, listing the possible key points and corresponding explanations:

1. **Determine the reason for the failure**:
   - **Re-examination**: See your dentist to re-evaluate the cause of root canal failure, which may include root canal not being cleaned, periapical lesions, missing infected areas within the root canal, or inadequate root canal filling.
   - **Imaging tests**: X-rays or CT scans are used to learn more about the specific nature of the problem, such as root fractures, fissures, or other problems that affect the effectiveness of treatment.
2. **Choice of treatment options**:
   - **Non-surgical retreatment**: If the initial root canal treatment fails, but structural conditions permit, non-surgical root canal retreatment, i.e., re-cleaning, sterilization, and filling, may be considered.
     - **Medications**: Antibiotics or anti-inflammatory drugs are used to control infection and inflammation.
     - **Root canal cleaning again**: Remove the old root canal filling and clean the root canal system thoroughly.
     - **Root canal re-sterilization**: Use an effective disinfectant to kill residual bacteria.
     - **Root canal refilling**: Use a new root canal filling material to ensure the tightness of the root canal system.
   - **Surgical treatment**: If non-surgical treatment is not appropriate or fails, surgery such as apicoectomy may be required.
     - **Apical resection**: Removal of the infected part of the root apex, sometimes with reverse filling of the end of the root canal.
     - **Apical minimally invasive surgery**: Delicate surgical manipulation using equipment such as a microscope to improve the success rate.
3. **Consider alternatives**:
   - **Tooth extraction**: Tooth extraction may need to be considered when the tooth cannot be treated again or the prognosis is poor.
     - **Management after tooth extraction**: After tooth extraction, dental implants, fixed bridges, and removable dentures can be considered to restore chewing function.
   - **Dental implants**: If the tooth cannot be preserved, dental implants are a better restoration option that can provide a similar appearance and function to natural teeth.
4. **Prevention and maintenance**:
   - **Regular check-ups**: Even if you feel good after root canal treatment, you should visit your dentist regularly to monitor the effectiveness of your treatment.
   - **Oral hygiene**: Maintaining good oral hygiene habits, such as daily brushing, flossing, and mouthwash, can prevent re-infection of your teeth.
   - **Regular cleaning**: Regular professional cleanings at the dental clinic can help maintain dental health by removing tartar and plaque.
5. **Psychological support**:
   - **Mental Preparedness**: It is important to maintain a positive mindset in the face of treatment failure, understand that treatment failure is not uncommon, and that there are multiple solutions available.
   - **Communicate with** your doctor: Communicate your concerns and expectations with your dentist to understand the pros and cons of each treatment option and make the best decision together.
6. **Economic considerations**:
   - **Cost Assessment**: The cost of root canal treatment may vary by region, clinic, and complexity of treatment, understand and assess the financial burden of different treatment options.
   - **Insurance Inquiries**: If you have dental insurance, understand the coverage and limitations, as well as the possible out-of-pocket percentage.

In summary, the management of root canal treatment after failure requires a comprehensive consideration of various factors, including the cause of failure, feasible treatment options, alternatives, preventive and maintenance measures, psychological and economic factors. It is important to maintain communication with your dentist and work together to develop a personalized treatment plan that works best for you.

1. What are the options for dental fillings?

Regarding the choice of filling materials, here are the answers that have been recombined based on what I have searched:

1. **银汞合金（Amalgam）**：
   - **A long history**: Amalgam is a traditional filling material that has been used for more than 150 years.
   - **High strength**: It has good durability and high compressive strength, and is suitable for filling the chewing surface of the back teeth.
   - **Cost-effective**: Amalgam is generally more economical than other materials.
   - **Poor aesthetics**: Because its color does not match the natural teeth, it is not as aesthetically pleasing as other filling materials.
   - **Potential controversy**: Contains mercury, which is considered safe by most studies, but there are concerns about the potential health risks of mercury.
2. **Composite resin**:
   - **Aesthetics**: Can match the color of the teeth for a more natural look.
   - **Adhesion**: Bonds with the tooth structure and helps to restore the strength of the tooth.
   - **Mercury-free**: Mercury-free, it is a more popular modern option.
   - **Less durable**: May not be as durable as amalgam, especially when subjected to large chewing forces.
   - **Price**: Usually more expensive than amalgam.
3. **Glass Ionomer（Glass Ionomer）**：
   - **Fluoride release**: Fluoride is released to help prevent further tooth decay.
   - **Adhesion**: Able to bond firmly with teeth.
   - **Strength**: Low strength, not suitable for areas of the tooth that are subjected to high pressure.
   - **Beautiful**: The color can be adjusted, but the gloss and transparency are not as good as those of composite resins.
4. **Ceramic**:
   - **High aesthetics**: The color and light transmittance are similar to those of natural teeth, and the aesthetics are excellent.
   - **Abrasion resistance**: wear-resistant, good durability, suitable for long-term use.
   - **Price**: The cost is high and requires a precise manufacturing process.
   - **Brittleness**: Although abrasion-resistant, it is more brittle than composite resins and may shatter when subjected to strong impacts.
5. **Metal filling (e.g. gold, platinum):**
   - **Durability**: Very durable, suitable for long-term filling.
   - **Biocompatibility**: Good biocompatibility, not easy to cause allergic reactions.
   - **Price**: Expensive, the highest cost of all filling materials.
   - **Aesthetics**: The color of the metal is very different from that of natural teeth, and it is not as beautiful as other materials.
6. **Indirect filling（Inlays和Onlays）**：
   - **Adaptability**: Suitable for large areas of damaged teeth, which can be metal, ceramic, or composite.
   - **Durability**: Because it is made in a dental lab and then bonded to the teeth, it is usually stronger than a direct filling.
   - **Cost**: The cost is higher because of the need for lab work and multiple dental visits.
   - **Procedure**: Two or more dental visits are required to complete.
7. **Temporary Filling Materials**:
   - **Transient**: Usually used for teeth that require follow-up treatment, such as while waiting for a permanent filling or crown to be made.
   - **Ingredients**: May contain pharmaceutical ingredients to reduce pain or reduce infection.
   - **Durability**: Designed as a short-term solution only, with limited durability.

Factors to consider when choosing a dental filling include the size and location of the filling area, the functional needs of the tooth (e.g., the amount of chewing force), the patient's sensitivity to the material, aesthetic needs, and economic considerations. It is advisable to communicate with your dentist in detail before making a choice in order to find the most suitable filling material for your situation.

1. What do I need to pay attention to after a tooth filling?

After a tooth filling, as a patient, you need to be aware of the following key points to ensure the long-term success of the filling and oral health:

1. **Precautions after filling**:
   - **Short-term considerations**: 1.1 **Anesthetic effect**: If you have used an anesthetic during treatment, avoid eating until the anesthetic effect wears off to avoid biting your tongue or cheek. 1.2. **Avoid hard food**: Avoid chewing hard or sticky foods within 24 hours of filling to avoid damage to the new filling. 1.3. **Temperature sensitivity**: Newly filled teeth may be sensitive to heat or cold, so foods or drinks with extreme temperatures should be avoided for the first few days.
   - **Long-term considerations**: 1.4 **Oral hygiene**: Brush your teeth twice a day, use fluoride toothpaste, and floss daily to maintain oral hygiene. 1.5. **Regular check-ups**: Visit your dentist regularly to check the fillings, which are generally recommended every 6 months to ensure that the fillings are not loose or worn.
2. **Dietary advice**:
   - **Avoid specific foods**: 2.1 **Sticky foods**: Avoid eating too many sticky or hard foods like candy, caramel, etc., which may pull or stick to the filling, causing them to fall off. 2.2. **Acidic foods**: Too many acidic foods and beverages, such as citrus fruits and carbonated drinks, may erode teeth and fillings.
   - **Balanced diet**: 2.3 **Nutritionally** balanced: Maintaining a balanced diet is not only good for your overall health, but also for your dental health.
3. **Adjustment of lifestyle habits**:
   - **Avoid bad habits**: 3.1 **Teeth grinding and clenching**: If you have a habit of grinding or clenching, you may need to use a night guard to protect your teeth and fillings. 3.2. **Quit smoking**: Smoking increases the risk of oral problems and can cause discoloration of the teeth around the filling.
   - **Protections**: 3.3 **Use braces**: If you are involved in sports that can cause a blow to your face, you should wear braces to protect your teeth and fillings.
4. **Symptom Monitoring**:
   - **Common reactions**: 4.1 **Mild discomfort**: It is normal to experience mild discomfort or sensitivity for a few days after filling.
   - **Ongoing issues**: 4.2 **Persistent pain**: If the pain persists or gets worse, you should contact your dentist immediately. 4.3. **Abnormal bite**: If you don't feel normal when biting, you may need to go back to your dentist's office for adjustments.
5. **Emergency Handling**:
   - **Filling shedding**: 5.1 **Contact your dentist immediately**: If a filling comes off or is damaged, you should contact your dentist as soon as possible for a restoration.
   - **Signs of infection**: 5.2 **Keep an eye out for infection**: If you notice signs of infection such as redness, swelling, pain, or pus in the filled area, seek immediate medical attention.
6. **Medication use**:
   - **Pain Management**: 6.1 **Use pain relievers**: If you experience pain after a filling, you can use an over-the-counter pain reliever as recommended by your dentist.
   - **As directed by a physician**: 6.2 **Take your medications as directed**: If your dentist prescribes antibiotics or other medications, make sure to complete the entire course as directed.
7. **Long-term maintenance**:
   - **Filling life**: 7.1 **Know the lifespan**: Different materials of fillings have different lifespans, know the life expectancy of your fillings, and replace them when needed.
   - **Periodic review**: 7.2 **Follow-up visits**: Make regular follow-up visits to your dentist to ensure that the filling and teeth are in good condition.

The above key points provide comprehensive guidance to help you maintain your oral health after your teeth have been filled. If there is any discomfort or doubt, it is very important to consult your dentist promptly.

and subsequent restorations, such as crown overlays, to restore the function and appearance of the teeth.

1. What is the effect of diet on tooth decay?

The relationship between diet and tooth decay (commonly known as tooth decay) is a very important aspect of dental health. The following are the key points about the effect of diet on tooth decay, along with a detailed description of them:

1. **Sugar intake**:
   - Sugar and sugary foods are one of the main causes of tooth decay.
   - When you eat sugary foods, bacteria in your mouth use these sugars to produce acids.
   - This acid erodes the enamel on the tooth surface, causing mineral loss and the formation of tiny holes, known as initial caries.
   - If sugar intake is not controlled, these cavities will gradually enlarge and become apparent cavities.
2. **Frequency of eating**:
   - Frequent eating, especially sweets, will continue to provide bacteria with sugar for energy, resulting in a persistent acidic environment.
   - After each meal, the surface of the tooth will be eroded by the acidic environment for about 20 to 30 minutes, and frequent eating will reduce the time it takes for the tooth to repair itself.
3. **The stickiness of food**:
   - Sticky foods are more likely to adhere to the surface of the tooth and are not easily washed off by saliva.
   - These food residues provide a constant source of energy for the bacteria, increasing the chances of acid production.
4. **Acidic food and** drinks:
   - Acidic foods and beverages (e.g., lemon juice, carbonated drinks) inherently have the property of eroding tooth enamel.
   - Long-term exposure can cause tooth enamel to soften, increasing the risk of caries.
5. **Eating time**:
   - Eating before bedtime is a high-risk behavior for tooth decay because saliva production decreases during sleep and saliva self-purification decreases.
   - This means that food debris and acids produced by bacteria are not effectively removed at night, and the damage to teeth is even more severe.
6. **Nutrient intake**:
   - Calcium and phosphorus are important minerals that make up teeth, and moderate intake can contribute to the health of your teeth.
   - Vitamin D promotes the absorption and utilization of calcium, and a lack of vitamin D can affect the mineralization process of teeth.
   - Moderate amounts of fluoride can promote the remineralization of tooth enamel and enhance resistance to acids.
7. **The role of saliva**:
   - Saliva not only washes away food debris but also contains minerals that can help remineralize your teeth.
   - Consuming fiber-rich foods, such as fresh vegetables and fruits, can stimulate saliva production.
8. **Integrative Eating Patterns**:
   - A balanced diet contributes to overall health, including dental health.
   - Limiting foods high in sugar while increasing your intake of vitamin- and mineral-rich foods can help prevent tooth decay.
9. **Personal oral hygiene habits**:
   - Dietary habits work together with an individual's oral hygiene habits to contribute to the formation of dental caries.
   - Even if you eat a healthy diet, tooth decay can occur if you don't pay attention to your oral hygiene.

In summary, the effect of diet on dental caries is multifaceted. Limiting sugar intake, eating less frequently, avoiding sticky and acidic foods, paying attention to the timing of eating, ensuring nutrient intake, promoting saliva production, and maintaining a balanced diet are all effective measures to prevent tooth decay. At the same time, good oral hygiene habits are also an integral part of maintaining healthy teeth. As a patient, understanding these key points and applying them to your daily life can significantly reduce your risk of developing caries.

1. **Periodontal disease**
2. What is Periodontal Disease?

Periodontal disease is a common oral disease that mainly affects the tissues around the teeth, including the gums and alveolar bone. Here is a detailed explanation of periodontal disease, including key points and related descriptions:

1. **Definition and classification of periodontal disease**:
   - Periodontal disease is a chronic inflammatory disease of the supporting structures of the teeth, including gingivitis and periodontitis.
   - Gingivitis is an early stage of periodontal disease that presents with red, swollen and bleeding gums that do not affect the bones that are retained in the teeth.
   - Periodontitis is a more serious condition that includes the loss of alveolar bone in addition to the symptoms of gingivitis, which can lead to loose or even loss of teeth.
2. **Etiology**:
   - Periodontal disease is mainly caused by plaque formed by bacteria, which is a biofilm that adheres to the surface of teeth.
   - Long-term plaque and tartar accumulation can lead to gum inflammation, which can then develop into periodontitis.
   - Factors such as genetics, smoking, diabetes, stress, medication side effects, and poor nutrition may also increase the risk of periodontal disease.
3. **Symptoms**:
   - Bleeding, red, swollen and sore gums.
   - Bad breath and bad breath.
   - The teeth are loose and the alignment changes.
   - The gums are receding and the teeth look longer.
   - Discomfort in the bite or sensitive teeth.
4. **Diagnosis**:
   - The diagnosis of periodontal disease is usually made by a dentist through an oral examination and a periodontal probe to measure the depth of the periodontal pocket.
   - X-rays can help assess the loss of alveolar bone.
5. **Treatment**:
   - Initial treatment includes a professional teeth cleaning to remove plaque and tartar.
   - Deep cleaning (curettage and flattening) removes bacteria and tartar from periodontal pockets.
   - Severe periodontal disease may require periodontal surgery, including tooth retention surgery or gingival orthopedic surgery.
   - Antibiotics and antimicrobials are sometimes used as adjunctive therapy.
6. **Prevention**:
   - Good oral hygiene habits, including brushing and flossing twice a day.
   - Regular dental check-ups and cleanings can detect and treat periodontal problems early.
   - A healthy lifestyle, such as quitting smoking and eating a balanced diet, plays an important role in preventing periodontal disease.
7. **Complications**:
   - Periodontal disease is associated with whole-body health and may increase the risk of heart disease, diabetes, and other diseases.
   - Tooth loss can affect chewing function and facial aesthetics.
   - Periodontal disease can lead to pregnancy complications, such as premature birth and low birth weight babies.
8. **Long-term management**:
   - Periodontal disease is a chronic condition that can be managed but not completely cured.
   - Regular periodontal maintenance treatment and good personal oral hygiene habits are required.
   - Your dentist may recommend special toothbrushes, dental floss, and other cleaning aids.

The treatment and management of periodontal disease is a long-term process that requires the joint efforts of both the patient and the dentist. Regular dental check-ups and professional cleaning, combined with good daily oral hygiene habits, are essential to control the progression of periodontal disease. If you suspect that you have periodontal disease, you should consult a dentist as soon as possible for evaluation and treatment.

1. What are the causes of periodontal disease?

Periodontal disease is an inflammatory condition of the tissues surrounding the teeth that can affect the soft tissues and bones that support the teeth. Here are some of the key factors that cause periodontal disease to occur and a detailed explanation of them:

1. **Plaque accumulation:**
   - Plaque is the main cause of periodontal disease. It is a bacterial biofilm that adheres to the surface of the teeth.
   - When plaque is not removed regularly by brushing and flossing, it can harden and form tartar.
   - The formation of tartar makes it more difficult to remove plaque, providing a protective barrier against bacteria and exacerbating inflammation in the periodontal tissues.
2. **Smoking and use of tobacco products:**
   - Smoking is considered a major risk factor for periodontal disease.
   - Smoking reduces blood flow in the mouth, which reduces the ability of periodontal tissues to repair.
   - The chemicals in tobacco can also cause direct damage to the periodontal tissues.
3. **Personal Oral Hygiene Habits:**
   - Poor oral hygiene habits, such as incorrect or irregular brushing and flossing, can lead to the accumulation of plaque and tartar.
4. **Genetic factors:**
   - There is a certain genetic predisposition to periodontal disease, and some people may be more susceptible to periodontal disease due to genetic factors.
5. **Malnutrition:**
   - Malnutrition, especially vitamin C deficiency, can affect the health and repair ability of periodontal tissues.
6. **Hormonal changes:**
   - Changes in hormone levels during pregnancy, puberty, menstrual cycles, and menopause in women may make the gums more susceptible to inflammation.
7. **Disease:**
   - Certain diseases, such as diabetes and HIV/AIDS, can affect the body's immune system, making individuals more susceptible to periodontal disease.
   - Diabetic patients may develop and progress periodontal disease due to poor blood sugar control.
8. **Effects of the drug:**
   - Certain medications, such as antidepressants, heart medications, and birth control pills, can affect the flow of saliva in the mouth, reducing the natural cleansing effects of saliva.
   - In addition, certain medications can cause gum overgrowth, which increases the risk of periodontal disease.
9. **Stress:**
   - Stress is considered a factor that affects the body's ability to fight off infections and may increase the incidence of periodontal disease.
10. **Age:**
    - The risk of periodontal disease increases with age.
11. **Molars and crooked teeth:**
    - Teeth grinding increases pressure on the teeth and periodontal tissues, which can lead to periodontal disease.
    - Crooked teeth can lead to the accumulation of plaque and tartar, as crooked teeth are more difficult to clean.
12. **Improper use of oral equipment:**
    - Improper use or inappropriate dental restorations, such as crowns and bridges, can lead to plaque and tartar buildup.

The treatment of periodontal disease includes professional periodontal cleaning and possible periodontal surgery, as well as daily oral hygiene maintenance at home for the patient. The key to preventing periodontal disease is to maintain good oral hygiene habits, including regular brushing, flossing, and regular visits to the dentist for check-ups and cleanings. If you have any symptoms of periodontal disease, such as red, swollen gums, bleeding, or loose teeth, you should consult a dentist immediately.

1. What are the symptoms of periodontal disease?

Periodontal disease is a condition that affects the tissues around the teeth, including the gums and alveolar bone, and it includes gingivitis and the more severe periodontitis. Here are the main symptoms of periodontal disease, according to your requirements, I have arranged them in serial numbers and described them in detail:

1. **Bleeding gums**:
   - When brushing or flossing, your gums may bleed.
   - Bleeding is often one of the earliest signs of periodontal disease.
2. **Gum swelling and redness**：
   - The gums may become red, swollen and soft.
   - This inflammation is due to a bacterial infection caused by periodontal disease.
3. **Bad breath**:
   - Chronic bad breath or bad smell can be a sign of periodontal disease.
   - This condition may be due to the accumulation of bacteria in the mouth caused by periodontal disease.
4. **Gum recession**:
   - The gums may retract from the teeth, making the teeth appear longer.
   - This can lead to tooth sensitivity because the root of the tooth is exposed.
5. **Loose teeth**:
   - As periodontal disease progresses, teeth may become loose or move.
   - This is due to damage to the alveolar bone that can no longer support the tooth firmly.
6. **Chewing pain**:
   - Due to inflammation of the teeth and gums, there may be pain or discomfort when chewing.
7. **Periodontal abscess**:
   - Periodontal disease can lead to the formation of an abscess, which is a localized infection that is often accompanied by pain and swelling.
8. **Tooth alignment changes**:
   - Teeth may become displaced, resulting in a change in bite or misalignment of teeth.
9. **Sensitive Teeth**:
   - Teeth may become sensitive to hot, cold, or sweet foods.
   - This is usually due to gum recession exposing the tooth roots.
10. **Alveolar bone loss**:
    - In the advanced stages of periodontal disease, x-rays may show a loss of alveolar bone.
    - This type of osteoporosis can be irreversible and can lead to tooth loss in severe cases.
11. **Gum recession**:
    - The gums may no longer cling to the teeth, forming pockets or gaps.
    - These pockets can harbor food debris and bacteria that can exacerbate the condition.
12. **Alteration of bite force**:
    - When the teeth are loose or the alveolar bone is lost, the bite force may change.
    - This may affect the overall chewing efficiency.

Treatment of periodontal disease usually depends on the severity of the condition. Early periodontal disease, such as gingivitis, can usually be controlled with thorough cleaning and good oral hygiene. However, more severe periodontitis may require specialized dental treatment, including deep cleaning (scaling and root planing), medications, and possibly even periodontal surgery.

If you notice that you have any of the above symptoms, it is advisable to consult a dentist as soon as possible. If left untreated, periodontal disease can not only lead to tooth loss, but can also affect overall health because of the link between periodontal disease and heart disease, diabetes, and other chronic diseases. Therefore, maintaining good oral hygiene and regular dental check-ups are essential to prevent periodontal disease.

1. Is periodontal disease related to genetics?

Periodontal disease, an inflammatory disease of the periodontal tissues, is one of the main causes affecting oral health. Regarding the relationship between periodontal disease and heredity, here are the answers based on the information in my knowledge base:

1. Influence of genetic factors: a. Studies have shown that there is a correlation between the occurrence of periodontal disease and genetic factors. b. Specific genetic variants, such as IL-1 gene polymorphisms, have been associated with the development of periodontal disease. c. Genetic predisposition may result in individuals having different immune and inflammatory responses to periodontal pathogens. d. Hereditary periodontal disease, such as familial early-onset periodontal disease, is directly related to heredity.
2. The role of environmental factors: a. Smoking is an important risk factor for periodontal disease and can exacerbate genetic predisposition. b. Oral hygiene habits, such as brushing and flossing frequency, have a significant impact on the development of periodontal disease. c. Dietary habits, especially high-sugar diets, can also increase the risk of periodontal disease. d. Chronic diseases, such as diabetes, are strongly associated with periodontal disease.
3. Interaction between genetics and environment: a. Genetic predisposition and environmental factors often interact, and a person may inherit a predisposition to periodontal disease, but it will only manifest itself when certain environmental factors act up. b. Environmental factors may affect the expression of genes, a phenomenon known as epigenetics.
4. Relationship between clinical manifestations and heredity: a. The severity and rate of progression of periodontal disease varies between individuals, which may be partly explained by genetic factors. b. Patients with a family history of periodontal disease may require more frequent dental check-ups and more aggressive preventive measures.
5. Treatment and genetics: a. Recognizing the role of genetic factors in periodontal disease is important for developing a personalized treatment plan. b. Family members of patients with periodontal disease may need to be screened and intervened earlier.
6. Prevention strategies: a. Even if there is a genetic predisposition, good oral hygiene habits and regular dental check-ups are key to preventing periodontal disease. b. Quitting smoking and living a healthy lifestyle can significantly reduce the risk of periodontal disease.
7. Advances in scientific research: a. With the development of genomics and epigenetics, the role of genetic factors in periodontal disease is increasingly understood. b. Future research may uncover more relevant genetic markers that can help identify high-risk individuals at an early stage.
8. The Importance of Personalized Medicine: a. Genetic information can assist dentists in providing a more personalized treatment plan for their patients. b. Genetic testing may be part of the management of periodontal disease in the future.

In summary, periodontal disease is a complex disease whose development is influenced by a combination of genetic and environmental factors. Genetic factors may increase the susceptibility of certain populations to periodontal disease, but environmental factors, particularly lifestyle choices, also play a crucial role in the onset and progression of periodontal disease. Therefore, even if there is a genetic predisposition, the risk of periodontal disease can still be significantly reduced by taking proper precautions.

1. How is diabetes related to periodontal disease?

Diabetes and periodontal disease are intricately linked, with evidence showing a bidirectional relationship between the two. This means that not only does diabetes increase the risk for periodontal disease, but periodontal disease may also have the potential to affect blood glucose control and contribute to the progression of diabetes. Here, I will outline the connection between these two conditions in a structured format:

1. **Increased Risk of Periodontal Disease in Diabetic Patients**
   - **Higher susceptibility to infections:** Individuals with diabetes are generally more susceptible to bacterial infections due to a weakened immune system. This susceptibility also extends to the gums, making it easier for periodontal disease to develop.
     - *Impaired neutrophil function:* Diabetes can impair neutrophil function, which is crucial for fighting periodontal pathogens.
     - *Altered oral microbiome:* High blood sugar levels can lead to changes in the oral microbiome, favoring the growth of bacteria that contribute to gum disease.
2. **Impact of Periodontal Disease on Diabetes Control**
   - **Influence on blood glucose levels:** Severe periodontal disease can influence blood glucose levels and contribute to increased periods of time when the body functions with a high blood sugar level, complicating diabetes management.
     - *Inflammatory mediators:* Periodontal disease causes elevated levels of systemic inflammatory mediators such as TNF-alpha and IL-6, which can affect insulin resistance.
     - *Glycemic control challenges:* The ongoing inflammation from periodontal disease makes it harder to control glucose levels, thus exacerbating diabetes.
3. **Treatment of Periodontal Disease Improves Diabetes Outcomes**
   - **Better diabetes control with periodontal therapy:** Studies have shown that treating periodontal disease can help improve blood sugar control in diabetic patients, highlighting the importance of oral health in managing diabetes.
     - *Reduction in HbA1c:* Periodontal treatment has been associated with a reduction in levels of HbA1c, a marker of long-term blood glucose control.
     - *Decreased need for diabetes medications:* Some patients may experience a decrease in the need for insulin or other diabetes medications after successful treatment of their periodontal disease.
4. **Common Risk Factors Contributing to Both Conditions**
   - **Smoking:** Smoking is a significant risk factor for both diabetes and periodontal disease, exacerbating inflammation and impairing the body's immune response.
   - **Poor oral hygiene and diabetes management:** Neglecting oral hygiene can lead to the development of periodontal disease, just as poor management of diabetes leads to higher sugar levels, further increasing the risk for periodontal complications.
5. **Preventive Measures**
   - **Regular dental check-ups:** Regular visits to a dentist for cleanings and check-ups can help prevent the onset or progression of periodontal disease, which is particularly crucial for people with diabetes.
   - **Control of blood sugar levels:** Maintaining good control over blood glucose levels can reduce the risk of developing periodontal disease and help manage existing conditions more effectively.
   - **Lifestyle modifications:** Diet, exercise, and smoking cessation are critical in managing both diabetes and periodontal disease.
6. **Educational Aspect**
   - **Patient education:** Educating patients about the link between diabetes and periodontal disease is vital for early intervention and management. Patients should be aware of how interlinked these conditions are and the steps they can take to mitigate risks.
7. **Research and Future Directions**
   - **Ongoing research:** Research continues to explore the mechanisms linking diabetes and periodontal disease, aiming to develop more effective treatments and management strategies that address both conditions simultaneously.
   - **Innovative treatment approaches:** Future treatments may include targeted therapies that address the inflammatory processes common to both diabetes and periodontal disease.

Understanding the connection between diabetes and periodontal disease can significantly affect how both conditions are managed. It's essential for individuals with diabetes to be proactive about their oral health and vice versa, ensuring comprehensive care that considers the impact of one condition on the other.

1. How is periodontal disease related to pregnancy?

The association between periodontal disease and pregnancy is a complex and multifaceted topic, and here are some detailed answers based on what I know:

1. **Changes in hormone levels**:
   - During pregnancy, hormone levels in a woman's body change significantly, especially estrogen and progesterone levels increase significantly.
   - These hormonal changes affect the response of blood vessels within the mouth, causing the gums to be more sensitive, prone to bleeding and inflammation.
   - The inflammatory response of the gums can be exacerbated, and even a slight buildup of plaque can lead to symptoms of periodontal disease, a phenomenon sometimes referred to as "gingivitis gestationa".
2. **Immune system changes**:
   - During pregnancy, a woman's immune system adjusts to protect the fetus from being rejected by the mother's immune system.
   - This immune adjustment may reduce resistance to periodontal pathogens, making pregnant women more susceptible to periodontal disease.
3. **Changes in oral hygiene habits**:
   - Pregnant women may experience changes in their oral hygiene habits due to early pregnancy sickness, vomiting or changes in appetite.
   - These changes may reduce the frequency of brushing and flossing, increasing the accumulation of plaque and tartar, which can increase the risk of periodontal disease.
4. **Changes in nutrient intake**:
   - The need for certain nutrients, such as calcium and vitamin D, increases during pregnancy, which are essential for dental and periodontal tissue health.
   - If a pregnant woman's diet does not contain enough of these nutrients, it may affect the health of her teeth and periodontal tissues.
5. **Association between periodontal disease and maternal and fetal health during pregnancy**:
   - Studies have shown that severe periodontal disease may be associated with an increased risk of preterm birth and low birth weight babies.
   - Mechanisms that produce this association may include a systemic inflammatory response caused by periodontal disease that may trigger the physiological mechanisms of preterm birth.
   - Periodontal pathogens may also have a direct impact on fetal health, although research in this area is not sufficient.
6. **Prevention and treatment**:
   - Pregnant women should practice good oral hygiene, including regular brushing and flossing.
   - Regular dental check-ups are available to detect and treat periodontal disease in a timely manner.
   - Follow the advice of your dentist or periodontist, which may include a professional periodontal cleaning or other treatment.
   - Ensure balanced nutrition, especially calcium and vitamin D intake.
7. **Safety of dental treatment during pregnancy**:
   - Dental treatment during pregnancy is safe, but non-urgent treatment should be avoided during the first and last months of pregnancy.
   - The dentist should be informed about the pregnancy before any treatment so that appropriate precautions can be taken.

To summarize the above key points, there is a clear association between periodontal disease and pregnancy, which is mainly manifested in changes in hormone levels, immune system adjustment, changes in oral hygiene habits, changes in nutritional needs, and possible risks of periodontal disease to maternal and infant health. To ensure the health of the mother and baby, pregnant women should maintain good oral hygiene, have regular dental check-ups, and follow professional advice for the prevention and treatment of periodontal disease. When undergoing dental treatment during pregnancy, it is important to pay attention to the timing and safety of treatment.

1. Does periodontal disease increase the risk of heart disease?

The association between periodontal disease and heart disease is a complex medical topic, and current research suggests that there is a link between the two. Here's how you answered your question based on current scientific knowledge and research:

1. Role of inflammation:
   - Periodontal disease is a chronic inflammatory disease caused by bacteria that affects not only the teeth and gums, but also the health of the entire body.
   - Bacteria and inflammatory agents in periodontal disease, such as bacterial endotoxins and inflammatory mediators, may enter the bloodstream and cause or exacerbate inflammatory responses in distant organs, including the heart.
2. Atherosclerosis (lipid deposition in the walls of the arteries):
   - Studies have shown that periodontal disease may accelerate the progression of atherosclerosis, a key process in the onset of heart disease.
   - Patients with periodontal disease may have impaired vascular endothelial function, which is also a risk factor for heart disease.
3. Risk Factors for Heart Disease:
   - Periodontal disease is associated with several heart disease risk factors, such as diabetes, smoking, and obesity.
   - These common risk factors may be part of the explanation for the link between periodontal disease and heart disease.
4. Immune System Response:
   - The immune system of people with periodontal disease may overreact to the bacteria in the gums, and this reaction can have a negative impact on the heart.
   - Chronic inflammation can lead to long-term activation of the immune system, which may be associated with the development of heart disease.
5. Direct and circumstantial evidence:
   - Studies have shown that people with periodontal disease are more likely to develop heart disease than people with healthy periodontal disease.
   - Although there is a correlation, current evidence does not establish that periodontal disease is the direct cause of heart disease.
6. Statistical and epidemiological studies:
   - Numerous epidemiological studies support a statistically significant correlation between periodontal disease and heart disease.
   - These studies are usually observational, and they cannot prove causality, but they can reveal a potential link between the two diseases.
7. Clinical recommendations:
   - Many dental and cardiologists suggest that maintaining good oral hygiene may be beneficial for heart health.
   - Regular dental check-ups and treatment for periodontal disease may help reduce the risk of heart disease.
8. Endocarditis:
   - Periodontal disease is also considered a risk factor for infective endocarditis, a serious heart infection.
9. Summary:
   - Although the direct causal relationship between periodontal disease and heart disease has not been clearly proven, the correlation and underlying biological mechanisms suggest that we should pay sufficient attention to this association.
   - Maintaining good oral hygiene and regular check-ups may have potential benefits in preventing heart disease.

The above information is a synthesis of available scientific research and the opinions of medical experts, and it is recommended that you discuss your individual health situation with a medical professional for more specific advice and treatment.

1. Does periodontal disease affect digestive disorders?

Periodontal disease refers to a disease that affects the tissues around the teeth (gums, alveolar bone, etc.) and can have some impact on the digestive system. The following answers are recombined according to your requirements, based on the knowledge I searched, and arranged by key points:

1. **Periodontal disease is directly linked to the digestive system**
   - Periodontal disease can lead to decreased chewing efficiency because sore or loose teeth may affect the chewing of food.
     - a. Reduced chewing efficiency can lead to larger food particles, which can affect digestion.
     - b. Inadequate food chewing may lead to an increased burden on the gastrointestinal tract, which may cause indigestion.
   - Periodontal disease can cause tooth loss, which can affect normal chewing function.
2. **Possibility of germ transmission**
   - Pathogens in periodontal disease can spread through the digestive tract and affect the digestive system.
     - a. Germs in the mouth of people with periodontal disease may enter the digestive system with food.
     - b. These germs may cause infections inside the digestive tract.
3. **Inflammatory response**
   - Periodontal disease may cause a systemic inflammatory response.
     - a. The inflammatory response may affect other parts of the body, including the digestive system.
     - b. Chronic inflammation may be associated with digestive disorders such as irritable bowel syndrome.
4. **Immune function of the body**
   - Periodontal disease can affect the body's immune system.
     - a. Disorders of the immune system may lead to the occurrence or aggravation of digestive disorders.
     - b. Patients with periodontal disease may be more susceptible to gastrointestinal infections.
5. **Common risk factors**
   - Periodontal disease may share risk factors with certain digestive disorders, such as smoking, eating habits and lifestyle.
     - a. Smoking is not only a risk factor for periodontal disease, but also increases the risk of digestive disorders.
     - b. Unhealthy eating habits may exacerbate both periodontal disease and digestive disorders.
6. **nutrient uptake**
   - Periodontal disease can affect nutrient intake and absorption.
     - a. Dental problems may cause patients to choose soft or easy-to-chew foods, which may affect the balance of nutrients.
     - b. Malnutrition may further affect the health of the digestive system.
7. **Psychosocial factors**
   - Periodontal disease can cause pain and cosmetic problems, affecting the mental health of patients.
     - a. Psychological stress and anxiety may affect gastrointestinal function, such as causing excessive stomach acid or intestinal peristalsis disorders.
     - b. Mental health conditions may indirectly affect the health of the digestive system.

To summarize the above key points, although periodontal disease mainly affects oral health, it may indirectly affect the digestive system through a variety of mechanisms. Periodontal disease can have an impact on the digestive system, from decreased chewing efficiency to systemic inflammation and changes in the immune system. In addition, common risk factors and psychosocial factors may also establish a link between periodontal disease and digestive disorders. Therefore, maintaining good oral hygiene is not only essential for dental health, but also an important aspect of maintaining overall health. If you have periodontal disease, it is recommended that you consult a dentist and gastroenterologist for comprehensive health management advice.

1. How is periodontal disease treated?

Periodontal disease refers to a series of diseases that affect the tissues around the teeth (gums, alveolar bone, etc.), and treatment of periodontal disease often requires a comprehensive approach. The following are the key points for the treatment of periodontal disease that have been recombined based on the knowledge I have searched:

1. **Initial assessment and diagnosis**:
   - 1.1. **Comprehensive oral examination**: The treatment of periodontal disease begins with a comprehensive oral examination, including the condition of the teeth, gums, and the presence or absence of calculus.
   - 1.2. **Periodontal examination**: The severity of periodontal disease is assessed by checking the depth of the periodontal pocket with a probe.
   - 1.3. **Imaging tests**: X-rays may be required to assess the loss of alveolar bone.
2. **Oral Hygiene Education**:
   - 2.1. **Brushing Techniques**: Instruct on proper brushing techniques, including brushing angles, pressures, and the use of a soft-bristled toothbrush.
   - 2.2. **Flossing**: Teach how to properly floss to remove food debris and plaque from between your teeth.
   - 2.3. **Other Auxiliary Tools**: Introduce the use of auxiliary tools such as interdental brushes and water flossers.
3. **Non-surgical treatment**:
   - 3.1. **Scaling**: Removes plaque and tartar from the tooth surface and under the gums.
   - 3.2. **Root scaling**: Root scaling is performed deep into the periodontal pocket to remove pathological substances on the root surface.
   - 3.3. **Pharmacotherapy**: Topical antibiotic gel or systemic antibiotic therapy to control infection.
4. **Surgical treatment** (considered if non-surgical treatment is ineffective):
   - 4.1. **Flap surgery**: The root and alveolar bone are surgically exposed in order to remove the tartar and diseased tissue more thoroughly.
   - 4.2. **Bone regeneration surgery**: the use of membrane techniques or bone substitutes to promote the regeneration of damaged alveolar bone.
   - 4.3. **Periodontal plasty**: Reshape or adjust the gums and alveolar bone to improve periodontal health and aesthetics.
   - 4.4. **Periodontal tissue grafting**: Tissue is taken from other sites and transplanted to the damaged area to facilitate periodontal tissue restoration.
5. **Lifestyle Adjustments**:
   - 5.1. **Smoking cessation**: Smoking is an important risk factor for periodontal disease, and smoking cessation is very important for the treatment of periodontal disease.
   - 5.2. **Diet**: Maintain a balanced diet and increase your intake of vitamin C and other nutrients that contribute to gum health.
   - 5.3. **Control diabetes**: People with diabetes are more likely to develop periodontal disease, so it is important to control blood sugar levels.
6. **Regular review and maintenance**:
   - 6.1. **Regular cleaning**: Professional cleaning every 3 to 6 months to prevent recurrence of periodontal disease.
   - 6.2. **Follow-up**: Periodontal condition is reviewed regularly to evaluate the treatment effect and maintain the treatment results.
7. **Holistic Health Management**:
   - 7.1. Stress **management**: Stress can exacerbate periodontal disease, and learning stress management techniques can help with overall health.
   - 7.2. **Disease control**: Other chronic diseases such as heart disease, rheumatoid arthritis, etc. may also be associated with periodontal disease and require comprehensive management.

Each treatment needs to be tailored to the individual's specific situation, so it is recommended to do it under the guidance of a dentist. The treatment of periodontal disease is a long-term process that requires the active cooperation of patients and changes in lifestyle habits to achieve the best treatment results.

1. What should be paid attention to in the daily care of patients with periodontal disease?

Periodontal disease is chronic inflammation of the supporting structures of the teeth, including the gums, alveolar bone, and periodontal ligaments. Routine care is essential to control periodontal disease. Here are the key points of day-to-day care for periodontal patients, reassembled from the knowledge base I searched:

1. **Proper brushing techniques**:
   - Use a soft-bristled toothbrush: Reduces irritation to the gums.
   - Brushing with the Belleville method: Brushing at a 45-degree angle helps remove plaque from the gum margins and between the teeth.
   - Brushing time: at least 2 minutes at a time, at least twice a day.
2. **Use dental floss and auxiliary cleaning tools**:
   - Floss daily: Remove food debris and plaque from between your teeth.
   - Special auxiliary tools for periodontal disease: such as interstitial brushes, periodontal brushes, etc., for periodontal pockets and large interstitial cleaning.
3. **Dietary Adjustments**:
   - Reduce your intake of sugars and refined carbohydrates: These foods tend to cause plaque growth.
   - Increase your intake of vitamin C and vitamin A: Promotes healthy gums.
   - Adequate hydration: Helps clean the mouth and promotes saliva production.
4. **Quit smoking and limit alcohol:**
   - Smoking is a major risk factor for periodontal disease, and quitting smoking can significantly improve periodontal health.
   - Limit alcohol consumption: Excessive alcohol consumption can affect the oral cavity's resistance.
5. **Regular dental check-ups**:
   - Periodontal examination every 6 months to 1 year: timely detection and treatment of periodontal problems.
   - Deep cleaning: Periodontal deep cleaning as recommended by your dentist.
6. **Control of systemic disease**:
   - Manage blood sugar: People with diabetes need to control their blood sugar levels because unstable blood sugar can worsen periodontal disease.
   - Managing stress: Stress can affect the immune system and exacerbate periodontal inflammation.
7. **Use fluoride toothpaste and mouthwash**:
   - Fluoride toothpaste: Can help strengthen teeth and prevent tooth decay.
   - Antibacterial mouthwash: Helps reduce bacteria in the mouth, but should be used under the guidance of a dentist.
8. **Develop good habits**:
   - Avoid opening caps or biting hard objects with your teeth: protect your teeth and periodontal structures from damage.
   - Maintain good sleep quality: Getting enough sleep is good for both overall health and periodontal health.
9. **Supplementation of nutrients and trace elements**:
   - Calcium and phosphorus intake: contributes to the health of teeth and bones.
   - Omega-3 fatty acids: They have been shown to reduce the inflammatory response in periodontal disease.
10. **Pay attention to other problems in the mouth**:
    - Watch for symptoms such as loose teeth, bleeding, or odor, and seek medical attention promptly.
    - Tooth wear or incorrect bite can also aggravate periodontal disease and need to be adjusted promptly.

Day-to-day care for periodontal disease is a comprehensive process that involves personal hygiene habits, lifestyle, eating habits, and regular professional dental care. Patients should follow the above key points, combined with the advice of a professional dentist, to maintain oral health and control or reverse the progression of periodontal disease.

1. **Problems related to oral mucosal diseases**
2. What is oral mucosal disease?

Oral mucosal diseases refer to a series of diseases that occur on the oral mucosa, including various inflammations, infections, immune diseases, tumors, etc. These diseases can be caused by a variety of factors, including bacterial, viral infections, autoimmune reactions, genetic factors, environmental factors, malnutrition, drug reactions, etc. Oral mucosal diseases can manifest in a variety of forms, ranging from mild discomfort to severe pain or dysfunction. Below, I will introduce the key points of oral mucosal disease in detail according to your requirements:

1. **Classification of etiology**:
   - **Infectious diseases**: Infections of the oral mucosa caused by microorganisms such as bacteria, viruses, fungi, etc. Such as:
     - Viral infections, such as oral herpes caused by the herpes simplex virus.
     - Bacterial infections such as gingivitis, periodontitis.
     - Fungal infections, commonly Candida, cause oral candidiasis.
   - **Immune-related diseases**: including autoimmune diseases such as Sjögren's syndrome, systemic lupus erythematosus, glossitis, etc.
   - **Tumors**: Benign and malignant tumors can occur in the oral mucosa, such as squamous cell carcinoma, fibroids, etc.
   - **Hereditary disorders**: Vitiligo, such as vitiligo, is a genetic disorder that manifests as white patches in the mouth.
   - **Trauma and physicochemical factors**, such as tobacco and alcohol use, as well as poor oral hygiene, can cause diseases of the oral mucosa.
2. **Clinical manifestations**:
   - **Pain**: is one of the most common symptoms, especially if there is inflammation or infection.
   - **Ulcers**: Small to medium-sized sores that appear on the oral mucosa with clear edges and can be caused by a variety of causes.
   - **Erythema and swelling**: The inflammatory response often leads to erythema and swelling of the oral mucosa.
   - **Vitiligo**: can be caused by a fungal infection, chronic irritation, or other pathological conditions.
   - **Bleeding**: Bleeding gums are a common symptom in diseases of the oral mucosa, especially in periodontal disease.
3. **Diagnosis**:
   - **Clinical examination**: a detailed history and a thorough oral examination are the basis of diagnosis.
   - **Laboratory tests**: including bacterial culture, viral PCR tests, blood tests, etc.
   - **Biopsy**: for unclear lesions, tissue biopsy is the gold standard for diagnosis.
4. **Treatment**:
   - **Medications**: Depending on the cause, these may include antibiotics, antivirals, antifungals, immunomodulators, etc.
   - **Topical** treatments: such as oral care, topical drug application, laser therapy, etc.
   - **Surgical treatment**: Surgery may be needed for certain types of tumors or lesions that do not heal.
5. **Prevention & Management**:
   - **Good oral hygiene habits**: Brush and floss your teeth regularly and get regular dental check-ups.
   - **Avoid bad lifestyle habits**: Reduce tobacco and alcohol use.
   - **Healthy diet**: Adequate nutrient intake, especially vitamin C and B vitamins, can help maintain oral health.
   - **Timely treatment**: In the face of any oral discomfort, you should seek medical attention in time to avoid aggravation of the condition.

There are various types of oral mucosal diseases and complex influencing factors, so the treatment and management need to take into account individual differences. Regular oral health check-ups can help to detect problems early and treat them in time, thereby maintaining oral health and improving quality of life.

1. What are the common oral mucosal diseases?

Oral mucosal diseases refer to various diseases that occur on the oral mucosa, including infectious diseases, immune diseases, neoplastic diseases, etc. The following are the classifications of some common oral mucosal diseases and their representative diseases:

1. **Infectious Diseases**:
   - **Viral Infections**:
     - **Herpes simplex virus infection**: usually presents as painful blisters that can appear on the lips or in the mouth, often caused by HSV-1 (herpes simplex virus type 1).
     - **Hand, foot and mouth disease**: Commonly seen in children, caused by coxsackievirus, etc., manifested as oral vesicular rash and hand and foot rash.
   - **Fungal** infections:
     - **Candida infection**: causes thrush and is common in immunocompromised individuals, such as infants, the elderly, or patients after antibiotic treatment.
   - **Bacterial infections**:
     - **Syphilis**: caused by Treponema pallidum pallidum and can initially form chancre in the mouth.
     - **Necrotizing ulcerative gingivostomatitis**: Also known as "necrotizing gingivitis", it is common in immunocompromised patients and presents with severe pain, bad breath, and gingival margin necrosis.
2. **Immune Diseases**:
   - **Recurrent aphthous ulcers**: Also known as mouth ulcers, they are a common inflammation of the mucous membranes that manifest as painful sores that occur periodically.
   - **Pemphigus**: A rare autoimmune disease that manifests as blisters and erosions in the mouth.
   - **Lupus erythematosus**: may affect the oral mucosa and manifest as reticular erythema or ulcers.
3. **Neoplastic Diseases**:
   - **Vitiligo**: may be a precursor to oral cancer, appearing as indelible white spots.
   - **Squamous cell carcinoma of the oral** cavity: is the most common malignancy of the oral cavity and may develop from vitiligo.
   - **Fibroids**: benign tumors that appear as bulges or nodules on the mucous membranes.
4. **Other diseases**:
   - **Oral submucosal fibrosis**: associated with betel nut chewing and manifested by limited oral opening and mucosal sclerosis.
   - **Geographic tongue**: a map-like erythematous patch, usually asymptomatic, but sometimes burning or painful.
   - **Black-haired tongue**: The papillae of the tongue are too long, and bacteria or fungi are attached to the surface, appearing black or brown.
5. **Drug-related diseases**:
   - **Drug reactions**: Certain medications may cause oral mucosal reactions, such as fixation drug eruptions, drug-induced ulcers.
   - **Drug-induced allergic reactions to mucous membranes**: such as certain antibiotics, antiepileptic drugs, etc.
6. **Hereditary Diseases**:
   - **White spongiform mouth disease**: an inherited disorder characterized by white, spongiform changes in the oral mucosa.
7. **Nutritional deficiencies**:
   - **Iron deficiency anemia**: may cause glossitis or parakeratosis.
   - **Vitamin B12 deficiency**: may cause tongue pain and stomatitis.

The diagnosis of these diseases usually requires a clinical examination and necessary laboratory tests, including but not limited to tissue biopsies, blood tests, microbial cultures, etc. Treatment varies depending on the condition and can include medications, surgery, laser treatment, lifestyle modifications, and more.

Please note that the above information is for reference only, if you suspect that you have oral mucosal disease, please consult a medical professional for diagnosis and treatment in time.

1. Can oral mucosal disease become malignant?

As a patient, it is important to understand the potential risk of malignant transformation of oral mucosal disease for disease management and prevention. Here are the reassembled answers based on the information I searched in my knowledge base, arranged by key:

1. **Definition and classification of oral mucosal diseases**:
   - Oral mucosal diseases refer to various diseases that occur on the oral mucosa, including infectious diseases, immune-related diseases, neoplastic diseases, etc.
   - These diseases can be benign, such as mouth ulcers, or malignant, such as oral cancer.
2. **Possibility of malignant transformation**:
   - Some oral mucosal diseases have the potential to become malignant, which means that they may develop into malignant tumors.
   - For example, leukoplakia and erythroplakia are considered precancerous lesions of oral cancer.
3. **Risk Factors for Malignant Transformation**:
   - Smoking and alcohol consumption are known risk factors for oral cancer, and they can increase the chance of malignant oral mucosal disease.
   - Human papillomavirus (HPV) infection is also associated with malignant transformation of oral mucosal disease.
   - Long-term chronic irritation, such as ill-fitting denture edges, can also lead to oral mucosal lesions.
4. **Clinical manifestations of malignant transformation**:
   - Changing color, such as unusual white or red spots on the mucous membranes.
   - Ulcers or lumps on mucous membranes, especially those that don't heal.
   - Feeling pain or a foreign body sensation may be an early sign of malignant transformation.
5. **Diagnosis and Monitoring**:
   - Regular dental check-ups are essential for early diagnosis and treatment of oral mucosal diseases.
   - Oral mucosal biopsy is the standard method to confirm the nature of mucosal lesions.
   - High-risk lesions may require more frequent monitoring and retesting.
6. **Prevention & Treatment**:
   - Smoking cessation and alcohol restriction can significantly reduce the risk of malignant oral mucosal disease.
   - Regular oral health care and professional dental check-ups can detect problems early and take action.
   - For precancerous lesions or early-stage cancers that have already been diagnosed, treatment may include surgical removal, radiation therapy, or chemotherapy.
7. **Patient education and self-management**:
   - Patients should be educated about the method of oral self-examination and be aware of any abnormal changes in the oral cavity.
   - Understand your individual's risk factors, such as family history, lifestyle habits, and discuss strategies to reduce your risk with your healthcare provider.
8. **Psychological support and quality of life**:
   - Oral mucosal disease and the risk of malignant transformation can be psychologically taxing.
   - Providing psychological support and appropriate counselling services is an important part of maintaining a patient's quality of life.
9. **Follow-up studies and new treatments**:
   - Scientific research is constantly advancing, and new treatments and drugs are being developed and tested.
   - Patients should maintain communication with their care team to stay up-to-date on treatment options and clinical trials.

To summarize the above key points, although not all oral mucosal diseases will become malignant, there is a certain risk of lesions deteriorating into oral cancer. Understanding these risk factors and significantly reducing them can be achieved through regular dental check-ups, lifestyle changes, and appropriate treatment. At the same time, attention to the patient's mental health and quality of life is also an important aspect of the overall treatment plan. Working closely with medical professionals can help patients better manage their health conditions.

1. Are there any special oral cleaning products for people with oral mucosal disease?

For patients suffering from diseases of the oral mucosa, it is very important to choose the right oral cleaning products. Here's what you've searched and curated based on your current knowledge base to answer your questions:

1. **Hypoallergenic formula**
   - Patients with oral mucosal disease should choose products that are alcohol-free, low-foaming, and fragrance-free, as these ingredients may cause irritation or aggravate symptoms.
2. **Contains soothing ingredients**
   - a. Products may contain natural ingredients such as aloe vera and chamomile, which are believed to have soothing and healing properties.
   - b. Some products may contain vitamin E, which helps to promote the healing of damaged oral mucosa.
3. **Antimicrobial effect**
   - a. Choosing a mouthwash that contains antimicrobial ingredients such as chlorhexidine can help reduce the bacteria in your mouth, which can reduce the risk of infection.
   - b. However, long-term use of strong antimicrobial products that may cause an imbalance in the oral flora should be avoided.
4. **Gentle physical cleaning**
   - a. Using a toothbrush with soft bristles can reduce physical irritation to the lesion.
   - b. The gentle mode of the electric toothbrush is also an option as it can provide a stable and gentle cleaning power.
5. **Consideration of special ingredients**
   - Some products may contain nitrates, which may be helpful in reducing tooth sensitivity, but may not be appropriate for certain oral mucosal conditions.
   - b. Moderate amounts of fluoride can help prevent tooth decay, but excessive amounts can cause problems with the oral mucosa.
6. **Avoid abrasive ingredients**
   - Some toothpastes contain abrasives, ingredients that may have a cleaning effect on healthy teeth, but may cause additional damage in people with oral mucosal disease.
7. **Professional advice**
   - a. Before choosing any product, a dentist or dental hygienist should be consulted, who can recommend suitable products based on the patient's specific condition.
   - b. Regular dental check-ups can help monitor the progression of the disease and adjust the oral care regimen.
8. **Personalization**
   - a. Oral care is not one-size-fits-all, and everyone's situation is different, so it may be necessary to try several products to find the best one for you.
   - b. If discomfort occurs after using a product, discontinue use immediately and consult a physician.
9. **The importance of daily care**
   - a. In addition to choosing suitable cleaning products, patients should also maintain good oral hygiene habits, such as daily brushing and flossing.
   - b. Avoid using any food or drink that may irritate the oral mucosa, such as excessively hot, cold, or excessively spicy foods.
10. **Nutrition and general health**
    - a. Good nutrition is essential for the health of the oral mucosa, and ensuring adequate intake of vitamins and minerals is beneficial for healing and maintaining oral health.
    - b. General health conditions, such as controlling diabetes and avoiding smoking, can also have an impact on oral health.

In summary, patients with oral mucosal diseases should be especially careful when choosing oral cleaning products, avoid ingredients that may cause irritation, and seek professional advice. Maintaining good oral hygiene habits and general health are equally important for improving and maintaining oral health.

1. Do patients with oral mucosal disease need more frequent oral examinations?

Whether patients with diseases of the oral mucosa need more frequent oral examinations, the following are the answers compiled according to relevant medical knowledge:

1. **The importance of disease surveillance**:
   - Oral mucosal lesions may be chronic and recurrent, requiring regular monitoring to assess progression.
   - Regular check-ups can help detect new or worsening lesions, especially the potential risk of cancer.
2. **Disease Management**:
   - Frequent check-ups can help doctors evaluate the effectiveness of treatment and adjust treatment options.
   - Regular dental check-ups can help detect and manage complications related to the disease in a timely manner.
3. **Individualized Examination Plan**:
   - Depending on the severity and changes in your condition, your doctor will recommend different frequencies of tests.
   - For active or severe oral mucosal lesions, more frequent tests may be required.
4. **What to check**:
   - Tests include, but are not limited to, visual examination, palpation, and, if necessary, biopsy.
   - Imaging tests, such as X-rays or MRI scans, may also be included to evaluate lesions in deep structures.
5. **Patient self-monitoring**:
   - Patients should be educated on how to perform a self-oral exam so that they can self-monitor between exams.
   - Self-monitoring involves noticing any new or changing lesions within the mouth.
6. **Lifestyle Adjustments**:
   - Patients may need to adjust their diet to avoid irritating foods and drinks.
   - Smoking cessation and alcohol restriction are essential to reduce the risk of oral mucosal lesions.
7. **Psychological support**:
   - Long-term management of the disease can lead to psychological stress, so psychological support is also very important.
   - Regular doctor-patient communication can help patients better understand the disease and reduce anxiety.
8. **Multidisciplinary collaboration of the medical team**:
   - Dentists, dentists, pathologists, and other specialists as needed should work together.
   - Multidisciplinary teamwork helps to provide patients with a comprehensive treatment and monitoring plan.
9. **Education and Prevention**:
   - Patients should be educated about oral health to reduce the risk of disease recurrence.
   - Preventive measures include proper oral hygiene habits and regular dental cleaning.
10. **Track & Record**:
    - The doctor keeps track of the patient's medical records, including test results and response to treatment.
    - This helps to manage the patient's health in the long term and provides a basis for future treatment decisions.
11. **Arrangement of regular follow-up visits**:
    - Depending on the patient's specific situation, the doctor will arrange an appropriate follow-up period.
    - In stable cases, the interval between follow-up visits may be longer; May be shorter for unstable or treated cases.
12. **Economic considerations**:
    - Patients should consider the financial burden of testing and treatment, and explore possible financial support options with their physician.
    - In some cases, health insurance may cover the cost of these tests.

In summary, patients with oral mucosal lesions may indeed require more frequent oral examinations to ensure that the condition is effectively monitored and managed. The specific frequency and type of regular check-ups should be determined by the physician on a case-by-case basis. Active patient participation and appropriate lifestyle modification are also integral parts of disease management.

1. Are there any special dietary recommendations for people with oral mucosal disease?

Oral mucosal diseases refer to various diseases that occur on the oral mucosa that can cause pain, discomfort, or unbearable symptoms. To alleviate these symptoms, patients can take some special dietary measures. Here are some suggestions based on my knowledge base search and reorganization:

1. **Avoid spicy and irritating foods**:
   - Spicy foods such as chili peppers, mustard, etc. may irritate the oral mucosa and aggravate the symptoms.
   - The temperature of food and drinks should not be overheated to avoid burns.
2. **Reduce your intake of acidic foods and beverages**:
   - Acidic foods such as citrus fruits, tomatoes, carbonated drinks, etc., may irritate damaged oral mucosa.
3. **Avoid hard foods**:
   - Hard foods such as nuts, hard candies, etc., may cause physical damage to the oral mucosa.
   - Choose foods that are soft, easy to chew and swallow.
4. **Don't smoke and limit alcohol intake**:
   - Both smoking and alcohol contribute to oral mucosal disease, and they interfere with normal cell function within the mouth.
   - Long-term smoking can also lead to pigmentation of the oral mucosa.
5. **Maintain adequate hydration intake**:
   - Adequate hydration intake helps keep the mouth moist and reduces dryness and discomfort.
   - Drink plenty of water, or you can hydrate yourself with fruits and vegetables that have a high water content.
6. **Foods rich in vitamins and minerals**:
   - Nutrients such as B vitamins, vitamin C, zinc, and iron are essential for the health of the oral mucosa.
   - These nutrients can be obtained through foods such as whole grains, lean meats, legumes, green leafy vegetables, fruits, etc.
7. **Avoid allergens**:
   - If the patient is allergic to certain foods, these foods should be avoided as allergic reactions may exacerbate inflammation of the oral mucosa.
8. **Maintain oral hygiene**:
   - Good oral hygiene habits can help reduce the buildup of bacteria in the mouth and reduce symptoms.
   - Use a gentle, alcohol-free oral hygiene product.
9. **Regular oral check-ups**:
   - Regular dental check-ups can help detect problems early and treat them accordingly.
10. **Follow your doctor's advice**:
    - If the doctor prescribes a drug for oral mucosal disease, it should be used strictly according to the doctor's instructions.
    - Some medications may require a special combination of diet.

These recommendations can help people with oral mucosal diseases reduce discomfort and promote oral health. However, everyone's specific situation may be different, so it's best to consult a doctor or dietitian before making any changes to your diet. In addition, if symptoms do not improve after dietary modifications, or if there is any doubt, you should seek medical attention promptly.

1. How to avoid recurrence of oral mucosal disease?

The key points to avoid the recurrence of oral mucosal disease can be prevented and controlled from many aspects, and the following are some important measures based on existing medical knowledge:

1. **Basic Oral Hygiene Maintenance**:
   - Clean your teeth: Brush your teeth at least twice a day, use fluoride toothpaste, and floss properly.
   - Regular check-ups: Oral check-ups are conducted every six months to one year to detect and treat oral problems in a timely manner.
   - Professional cleaning: Perform regular dental professional cleanings to remove tartar and tartar.
2. **Healthy Lifestyle Habits**:
   - Diet: Maintain a balanced diet and reduce your intake of sugar and acidic foods to reduce the risk of tooth decay and periodontal disease.
   - Quit smoking and limit alcohol: Tobacco and alcohol are risk factors for oral mucosal disease and should be avoided as much as possible.
   - Hydration: Maintain adequate hydration to keep your mouth moist and reduce problems caused by dry mouth.
3. **Disease Management**:
   - Diabetes control: Diabetic patients should have tight blood sugar control because hyperglycemia increases the risk of oral mucosal disease.
   - Immune status: For immune system disorders, it is important to follow the doctor's instructions to manage the condition and avoid recurrence due to weakened immunity.
4. **Medication Use and Administration**:
   - Avoid long-term use of medications that cause oral mucosal disease, such as certain oral lozenges, sprays, etc.
   - If it must be used, it should be used under the guidance of a physician and possible alternatives should be sought.
5. **Psychological Stress Management**:
   - Stress reduction: Psychological stress is considered one of the triggers of canker sores, so proper stress management is done through exercise, meditation, hobbies, etc.
   - Sleep: Get enough sleep to help your body recover and maintain your immunity.
6. **Nutritional support**:
   - Vitamins: Get enough vitamins C and B complexes, which are essential for maintaining a healthy oral mucosa.
   - Trace elements: Adequate intake of trace elements such as iron and zinc is also an important factor in preventing the recurrence of oral mucosal diseases.
7. **Avoid mechanical stimuli**:
   - Oral hygiene options: Use a soft-bristled toothbrush and alcohol-free mouthwash to reduce irritation of the oral mucosa.
   - Food choices: Avoid foods that are too hard, too hot or too spicy to avoid damaging the oral mucosa.
8. **Prevention in special cases**:
   - Aligner users: Patients who wear aligners should pay more attention to oral hygiene to avoid irritation of the oral mucosa by aligners.
   - Pregnant women: Oral hygiene management should be strengthened as the oral mucosa may be more susceptible during pregnancy due to hormonal changes.
9. **Timely treatment of oral diseases**:
   - Timely treatment: Any oral disease, such as periodontal disease, gingivitis, etc., should be treated as soon as possible to avoid aggravating the damage to the oral mucosa.
10. **Regular review**:
    - Even if symptoms are relieved, regular follow-up tests should be done to detect possible signs of recurrence and prevent worsening.

The implementation of the above measures needs to be adjusted according to the specific situation of the individual and is recommended to be carried out under the guidance of a professional doctor. These measures not only help prevent the recurrence of oral mucosal diseases, but also help improve overall oral health.

1. Do patients with candidiasis infection need special oral protection?

Candidiasis is an infection caused by a fungus of the genus Candida, commonly found in the oral cavity, called oral candidiasis or thrush. The following are recommendations for special oral protection measures for patients with oral candidiasis:

1. **Maintain oral hygiene**:
   - Brush your teeth twice a day and use a toothbrush with soft bristles to reduce irritation of the oral mucosa.
   - Use an alcohol-free mouthwash because alcohol can irritate an already infected oral mucosa.
   - If you use dentures, make sure to clean and disinfect them daily to prevent Candida bacteria from accumulating on their surface.
2. **Dietary modifications**:
   - Reduce your intake of sugars and refined carbohydrates, as these foods provide adequate nutrients for Candida.
   - Add foods rich in prebiotics and probiotics, such as yogurt, to help balance the microbial community in your mouth.
3. **Medications**:
   - Use antifungal medications, such as mouthwashes that contain nutrients or antifungal ingredients, as prescribed.
   - Long-term use of immunosuppressants or broad-spectrum antibiotics should be discussed with your doctor about possible alternatives or adjuvant therapy.
4. **Avoid irritants**:
   - Avoid using oral hygiene products that contain strong fragrances or pigments.
   - Reduce smoking and alcohol abuse, which can damage the oral mucosa and increase the risk of infection.
5. **Maintain good general health**:
   - Make sure you get enough sleep and moderate amount of physical activity to boost the function of your immune system.
   - Control blood sugar levels, especially in diabetic patients, as high blood sugar can promote Candida growth.
6. **Regular check-ups**:
   - Visit your dentist regularly for dental check-ups to detect and treat oral problems early.
   - If symptoms persist or worsen, you should seek medical attention promptly, and you may need to adjust your treatment regimen.
7. **Education & Self-Management**:
   - Learn about candidiasis and how to manage and prevent infections.
   - Monitor changes in symptoms and record the effects of daily care measures so that they can be shared with medical providers.
8. **Precautions in special cases**:
   - Patients with weakened immune systems, such as HIV/AIDS patients or cancer patients undergoing chemotherapy, require more stringent oral hygiene measures and may require more frequent medical monitoring.
9. **Avoid cross-contamination**:
   - Use personal hygiene products such as personal toothbrushes and cups and do not share them with others to prevent cross-infection.
10. **Nutritional supplements**:
    - Consider supplementing with B vitamins and trace elements such as iron and zinc to support mucosal health and immune function.

In summary, patients with oral candidiasis need a series of special oral protection measures to control infections and promote oral health. It is important to follow your doctor's guidance and adjust the above recommendations to your individual's specific situation. If symptoms persist, seek medical attention.

1. How can I relieve canker sores on a daily basis?

Canker sores are a common lesion of the oral mucosa that can be caused by a variety of factors, including but not limited to stress, malnutrition, local trauma, altered immune status, or certain diseases. Here are some ways to relieve canker sores:

1. **Maintain oral hygiene**:
   - Use a toothbrush with soft bristles to reduce irritation of the oral mucosa.
   - Brush your teeth regularly with fluoride toothpaste to prevent bacterial infections.
   - Use a gentle, alcohol-free mouthwash to help reduce bacteria in your mouth.
2. **Avoid irritating foods and drinks**:
   - Reduce your intake of spicy, acidic, excessively hot, or too hard foods to reduce irritation of ulcers.
   - Avoid alcoholic and caffeinated beverages as they may irritate the oral mucosa.
3. **Improving Diet**:
   - Increase foods rich in B vitamins, vitamin C, zinc, and iron, such as lean meats, legumes, nuts, whole grains, and fresh fruits and vegetables.
   - Vitamin and mineral supplements may be considered if necessary, but it is best to do so on the advice of your doctor.
4. **Reduce stress and anxiety**:
   - Relieve stress through deep breathing, yoga, meditation, or other relaxation techniques.
   - Get enough rest and sleep to help your body recover and regulate your immune system.
5. **Use of topical medications**:
   - Topical painkillers, such as gels containing benzocaine, may be used to reduce pain.
   - Apply ointments containing steroids to reduce inflammation, but use as directed.
   - Use an antibacterial mouthwash, such as chlorhexidine mouthwash, to reduce bacterial infections at the ulcer site.
6. **Stay hydrated**:
   - Drinking enough water to maintain your body's water balance helps the oral mucosa repair itself.
7. **Avoid local trauma**:
   - Avoid biting into the cheeks or tongue to reduce additional oral damage.
   - If you wear dentures or braces, make sure they fit well and don't rub against the oral mucosa.
8. **Quit smoking**:
   - Smoking may prolong the healing time of mouth sores and increase the risk of infection.
9. **Regular check-ups**:
   - Have regular oral exams, especially if sores occur frequently or don't heal, to rule out more serious causes.
10. **Pain Management**:
    - Try to avoid touching the ulcer area while eating and drinking to reduce pain.
    - Ice cubes can be used to gently apply ice to the ulcer to reduce pain and inflammation.
11. **Immunomodulation**:
    - In some cases, canker sores are linked to the body's immune response, so regulating the immune system may help.
12. **Follow your doctor's advice**:
    - If canker sores occur frequently or are accompanied by other symptoms, you should seek help from your doctor and follow your doctor's professional treatment advice.

These methods may help relieve the discomfort of canker sores and promote healing. However, if canker sores persist and do not heal, or if they are accompanied by other serious symptoms, such as fever, difficulty swallowing, or persistent pain, you should seek medical attention promptly to rule out other underlying health problems.

1. Are there any ways in your daily life that can help improve the resistance of the oral mucosa?

Improving oral mucosal resistance is a multifaceted issue, involving lifestyle habits, diet, oral hygiene, and certain health care measures. The following is a description of some of the key points on how to improve the resistance of the oral mucosa based on existing medical knowledge and recommendations:

1. **Good oral hygiene**
   - (a) Brush your teeth regularly: Use fluoride toothpaste at least twice a day for at least two minutes at a time.
   - (b) Flossing: Flossing at least once a day to remove food debris and plaque from between your teeth.
   - (c) Regular dental check-ups: Professional dental check-ups and teeth cleaning should be conducted every six months to one year.
2. **Eat wisely**
   - (a) Increase intake of vitamins and minerals: Eat foods rich in vitamin C and vitamin A, such as fresh fruits and vegetables, which are very helpful in maintaining the health of the oral mucosa.
   - (b) Limit the intake of sugars and acidic foods: these foods can easily lead to tooth erosion and oral diseases.
   - (c) Increase calcium intake: such as milk, cheese and other dairy products, which contribute to healthy teeth and bones.
3. **Quit smoking and limit alcohol**
   - (a) The harmful substances in tobacco can damage the oral mucosa and reduce its resistance.
   - (b) Excessive alcohol consumption can interfere with the normal balance of microflora in the mouth and increase the risk of infection.
4. **Proper physical activity**
   - (a) Physical exercise can improve the immunity of the whole body, including the resistance of the oral mucosa.
   - (b) Maintain a regular exercise routine of at least 150 minutes of moderate-intensity exercise per week.
5. **Get enough sleep**
   - (a) Adequate sleep helps the immune system to recover and adjust.
   - (b) Adults should get 7 to 9 hours of sleep per night.
6. **Stress management**
   - (a) Long-term stress can affect the body's immune function, including oral immunity.
   - (b) Stress can be managed through meditation, yoga, or other relaxation techniques.
7. **Supplement with probiotics**
   - (a) Probiotics can help maintain the microbial balance in the mouth and help inhibit the growth of harmful bacteria.
   - (b) Probiotics can be consumed through the consumption of probiotic-rich foods or supplements.
8. **Avoid overuse of alcohol-based mouthwashes**
   - (a) Alcohol can disrupt the natural microbiota in the mouth, and long-term use may reduce the resistance of the oral mucosa.
   - (b) You can choose a non-alcoholic mouthwash or use it in moderation as recommended by your doctor.
9. **Keep your mouth moist**
   - (a) Dry mouth reduces the production of saliva, which is part of the mouth's natural defense system.
   - (b) The mouth can be kept moist by drinking water, using saliva substitutes, or oral health products that stimulate saliva production.

By combining the above points, it can effectively improve the resistance of the oral mucosa and reduce the occurrence of oral diseases. It's important to note that these recommendations are not set in stone and may vary from person to person, so it's best to consult with a doctor or oral health professional before implementing any health plan.

1. **oral related problems in children**
2. Why does the baby delay teething?

There are a number of reasons why a baby may not have teething, but here are some possible explanations based on current medical knowledge:

1. **Genetic factors**:
   - Child: If parents have teething late in their infancy, their babies may also have similar situations. Genetic factors play a role in many aspects of a child's development.
2. **Nutritional status**:
   - Sub-point A: If your baby lacks essential nutrients, such as calcium, phosphorus and vitamin D, it may affect the development of teeth.
   - Sub-point B: Breastfed babies tend to teeth later than formula-fed babies because of the difference in nutrients between breast milk and formula.
3. **Endocrine factors**:
   - Sub-dots: Certain abnormalities of the endocrine system, such as an underactive thyroid gland, may delay tooth growth.
4. **Birth weight**:
   - Small spots: Babies born prematurely or with low birth weight may teething later than babies born at term.
5. **Systemic diseases**:
   - Systemic diseases such as congenital heart disease and Down syndrome may affect your baby's overall development, including tooth growth.
6. **Neuromuscular conditions**:
   - Sub-dots: Disorders that affect muscle and nerve development, such as cerebral palsy, can also cause late teething in infants.
7. **Environmental factors**:
   - Sub-dots: Excessive environmental pollution and exposure to certain harmful chemicals may also affect your baby's development, including tooth growth.
8. **Individual differences in growth and development**:
   - Sub-small points: Every baby's development rate is different, and some babies naturally grow teeth later than others.
9. **Habits of the mother during pregnancy**:
   - Smoking, drinking alcohol or using certain medications during pregnancy can adversely affect the development of your baby's teeth.
10. **Disorders of Dental Development**:
    - Sub-dots: Some children may have a disorder of tooth development, such as tooth germ insufficiency, which requires a professional medical diagnosis.

While considering all of the above possibilities, here are some specific steps and recommendations:

- **Observations and Records**:
  - Keep track of your baby's growth milestones and share them with your doctor for comparison and evaluation.
- **Consult a medical professional**:
  - If you're concerned about your baby's late teething, you should consult a pediatrician or pediatric dentist for evaluation.
- **Carry out the necessary checks**:
  - Your doctor may recommend blood tests to assess nutritional status or X-rays to see how your teeth are under your gums.
- **Maintain good nutrition**:
  - Make sure your baby is getting adequate nutrition, especially calcium, phosphorus and vitamin D, which are essential for tooth development.
- **Avoid exposure to hazardous substances**:
  - Ensure the safety of the environment in which the baby grows during pregnancy and avoid exposure to substances that are harmful to development.
- **Regular medical check-ups**:
  - Take your baby for regular check-ups so that problems that may affect tooth growth can be detected and addressed in a timely manner.

In short, late teething in babies is a multi-factor problem, and it is necessary to comprehensively consider genetics, nutrition, environment, disease and other factors. In most cases, your baby will eventually develop healthy teeth, but if there are any concerns, it is best to consult a medical professional for individualized assessment and advice.

1. How can I brush my baby's teeth?

Brushing a baby's teeth is a job that requires care and patience, and here are a series of steps and points that I have compiled based on my medical knowledge and advice from paediatric dentistry:

1. **Find out when to brush your teeth**
   - Start early: Start cleaning your baby's gums even when their teeth haven't fully erupted.
   - Tooth eruption: Once a baby's first tooth erupts, a toothbrush should be started.
2. **Choose the right tool**:
   - Toothbrush choice: Use a small-headed, soft-bristled toothbrush designed for babies.
   - Choice of toothpaste: Use children's toothpaste with very low or no fluoride content and should not exceed the size of a grain of rice.
3. **Cleaning Method**:
   - Clean the gums: Before the tooth erupts, gently wipe the baby's gums with moistened gauze.
   - Clean your teeth: After your teeth erupt, gently brush your teeth and gums with a soft-bristled toothbrush.
4. **Brushing Steps**:
   - Step 1: Prepare warm water and soak your toothbrush.
   - Step 2: Squeeze a grain of rice-sized toothpaste onto the toothbrush.
   - Step 3: Gently brush the surface of each tooth, including the inner and outer sides and the occlusal surface.
   - Step 4: After brushing teeth, gently wipe the baby's mouth to help remove excess toothpaste.
5. **Frequency and duration of brushing**:
   - Frequency: At least twice a day, preferably in the morning and before bedtime.
   - Duration: Each brushing session lasts approximately 1-2 minutes.
6. **Supervision and education**:
   - Supervision: Always brush your teeth under adult supervision to ensure safe and proper brushing techniques.
   - Education: As babies grow older, teach them how to brush their own teeth, but adults should be involved in the brushing process until they are 6 years old.
7. **Prevention of accidental** swallowing:
   - Pay attention to the amount of toothpaste you use to prevent your baby from swallowing too much toothpaste.
8. **Regular dental check-ups**:
   - First check-up: The baby's first dental check-up should be within 6 months of the first tooth erupting, or before their 1st birthday.
   - Regular check-ups: Routine dental check-ups should be done every 6 months thereafter.
9. **Tackling the Challenge**:
   - If babies don't like brushing, try using a different flavor of toothpaste, or let them listen to music or watch videos while brushing their teeth to distract them.
10. **Encouragement and Rewards**:
    - Create a positive brushing experience: Give your baby positive feedback and appropriate rewards, such as stickers or extra story time, to encourage them to develop good brushing habits.
11. **Pay attention to your diet**:
    - Reduce sugar intake: Avoid giving babies too much sugary foods and drinks, as this may increase the risk of tooth decay.
    - Healthy eating: Encourage your baby to consume foods rich in calcium and phosphorus to promote dental health.

Through the above key points, we can help babies establish good oral hygiene habits, prevent tooth decay, and promote their overall health development. These steps and recommendations are compiled based on current medical knowledge and advice from paediatric dental specialists and are designed to provide a comprehensive guidance protocol for parents or carers.

1. What should I do if a new tooth grows in and the old one doesn't fall out?

If a new tooth erupts and the old tooth does not fall out, this condition is medically known as baby tooth retention, which means that the baby tooth does not fall out on time when the permanent tooth erupts, resulting in the permanent tooth not erupting properly or in the wrong position. Here are some details and recommendations on the situation:

1. **Identify the problem**:
   - **1.1** Know the symptoms: Typically, children will start changing their teeth at about 6 years of age, with baby teeth falling out naturally and permanent teeth subsequently erupting in place.
   - **1.2** Observe eruption: If you find that the permanent teeth are starting to erupt but the baby teeth are not falling out, this may cause the permanent teeth to grow next to the baby teeth, forming a double row of teeth.
2. **Possible Causes**:
   - **2.1** Genetic factors: Sometimes retention of baby teeth is related to genetics.
   - **2.2** Abnormal tooth development: Abnormal development of permanent teeth under baby teeth may lead to non-loss of baby teeth.
   - **2.3** Injury or disease: Tooth damage or certain diseases may also affect the loss of baby teeth.
3. **Potential Issues**:
   - **3.1** Crowded teeth: The coexistence of permanent and baby teeth can lead to crowding of teeth, affecting bite and tooth alignment.
   - **3.2** Difficulty in teeth cleaning: Double rows of teeth can make teeth cleaning difficult, increasing the risk of tooth decay and periodontal disease.
   - **3.3** Psychological effects: For children, dental problems can affect their self-confidence and social interactions.
4. **Treatment Recommendations**:
   - **4.1** Watchful waiting: If there are still signs of loosening of baby teeth, they may fall out naturally, so you can observe them for a period of time.
   - **4.2** Surgical intervention: If the baby tooth is firm and immobile, it may need to be extracted by a dentist.
   - **4.3** Orthodontic treatment: After the removal of baby teeth, orthodontic treatment may be required if the permanent teeth are not in the right position.
5. **Follow-up care**:
   - **5.1** Regular check-ups: Go to the dentist regularly for check-ups to monitor the development of your teeth.
   - **5.2** Oral hygiene: Maintain good oral hygiene habits to prevent tooth decay and periodontal disease.
   - **5.3** Eat right: Avoid excessive intake of sugary foods and beverages to reduce the risk of tooth decay.
6. **Precautionary measures**:
   - **6.1** Early intervention: For children with a genetic predisposition, early intervention may help prevent retention of primary teeth.
   - **6.2** Avoid habitual bites: Educate children to avoid habitual bites to reduce damage to baby teeth.
7. **Awareness of importance**:
   - **7.1** Understand the impact: Recognise the potential impact of retained baby teeth on children's dental development.
   - **7.2** Professional Guidance: Follow the dentist's guidance for proper treatment and care.
8. **The role of the parent**:
   - **8.1** Supervised care: Parents should ensure that their child adheres to good oral hygiene habits.
   - **8.2** Educational awareness: Parents should enhance children's awareness and attention to oral health.

When dealing with the problem of retained baby teeth, it is important to consult with a professional dentist who will provide personalized diagnosis and treatment recommendations based on the child's specific situation. Following a doctor's advice and having regular dental check-ups are essential to ensure healthy growth of a child's teeth.

1. What happens when a newly erupted tooth has white patches or "pits" on the surface?

White patches or "pits" on teeth can be caused by a variety of reasons, but here are some possible explanations, as well as background information and suggestions:

1. **Incomplete mineralization in the teeth**:
   - The inside of the tooth is the outermost layer of hard tissue that covers the surface of the tooth, and its degree of mineralization determines the hardness of the tooth.
   - If the mineralization in the teeth is incomplete, it may lead to white spots or small pits on the surface of the teeth.
   - This condition can be caused by genetic factors, malnutrition, or certain interferences during tooth development, such as disease or drug influences.
2. **Dental fluorosis**:
   - Dental fluorosis is caused by excessive fluoride ingestion in children during tooth development.
   - In this case, the teeth may appear white spots, or even turn brown in severe cases, accompanied by a depression in the tooth surface.
   - Preventive measures include controlling your child's fluoride intake, such as using a moderate amount of fluoride toothpaste.
3. **Early caries (precaries lesions):**
   - White patches may appear when the tooth begins to demineralize, but no visible cavities have yet formed.
   - This is an early sign of caries and is usually caused by poor oral hygiene, excessive sugar intake in the diet, or bacterial activity in the mouth.
   - By improving oral hygiene, regular use of fluoride toothpaste and mouthwash can help reverse this condition.
4. **Dental trauma**:
   - When a tooth is struck by an external force, it may cause damage to the inside of the tooth, forming white spots or pits.
   - This condition requires a professional evaluation by a dentist to determine if treatment is needed.
5. **Nutritional deficiencies**:
   - Deficiencies in specific nutrients, such as calcium, phosphorus, vitamin D, etc., can affect the health of your teeth and lead to underdevelopment of your teeth.
   - Ensuring a balanced diet and proper nutritional supplementation is key to preventing this.
6. **Accumulation of tartar or tartar:**
   - Tartar that has not been removed effectively over a long period of time may calcify into calculus, which can lead to uneven tooth surfaces that look like small pits.
   - Regular scaling and proper brushing habits can prevent the formation of dental calculus.
7. **Hereditary Diseases**:
   - Certain genetic disorders can affect tooth development, leading to incomplete formation in the teeth, resulting in white patches or pits.
   - This condition requires genetic counseling and professional dental treatment.
8. **Oral Hygiene Habits**:
   - Incorrect brushing or the use of a hard toothbrush can cause damage to the tooth surface.
   - It is important to learn proper brushing techniques and choose the right toothbrush.
9. **Environmental factors**:
   - Certain environmental factors, such as chemicals in water sources, may also affect the health of your teeth.
   - Understanding and controlling potential risks in your living environment can be helpful in preventing dental problems.
10. **Regular dental check-ups**:
    - Regular dental check-ups can detect dental problems early and treat them.
    - Dentists can provide professional diagnosis and advice to help maintain dental health.

If you have white patches or small pits on your teeth, it is advisable to make an appointment with your dentist as soon as possible to determine the specific cause and get treatment recommendations. The health of your teeth is very important for the overall physical health, so any possible problems should not be overlooked.

1. How is caries in baby teeth treated?

Tooth decay in deciduous teeth refers to tooth erosion or damage that occurs on a child's deciduous teeth. Prompt treatment of caries in primary teeth is very important because baby teeth provide space for the eruption of permanent teeth and contribute to the child's chewing and language development. Here are the key steps and precautions for treating caries in deciduous teeth:

1. **Preliminary examination**
   - **Symptom confirmation**: Usually includes tooth pain, sensitivity, or a significant hole in the tooth.
   - **Professional diagnosis**: A dentist's examination is required to confirm whether or not there is caries and the extent of caries.
2. **Treatment options**
   - **Mild tooth decay**
     - **Medications**: Initial tooth decay may only require fluoride mouthwash or fluoride coating to strengthen the teeth.
     - **Minimally invasive repair**: Small cavities are filled with resin or other restorative materials.
   - **Moderate tooth decay**
     - **Removal of decayed tissue**: Removal of dead tooth tissue from the decayed part.
     - **Filling treatment**: Fill the cleaned cavity with suitable materials (e.g., amalgam, composite resin, glass ions, etc.).
   - **Severe tooth decay**
     - **Endodontic treatment**: Deep caries may invade the pulp and require endodontic treatment, such as fracture therapy or root canal treatment.
     - **Crown restoration**: Severely damaged teeth may require restorations that cover the entire crown, such as a stainless steel crown.
3. **Precautionary measures**
   - **Daily care**: Make sure your child brushes his teeth twice a day, uses fluoride toothpaste, and flosses properly.
   - **Regular check-ups**: Take your child to the dentist for check-ups and professional cleanings every six months to a year.
   - **Eating habits**: Reduce your child's intake of high-sugar foods and beverages and avoid snacking before bedtime.
4. **Parental guidance**
   - **Educational Importance**: Explain to parents the importance of baby teeth and proper oral hygiene practices.
   - **Assisted care**: Parents should help their children brush their teeth when they are young until they are able to do it properly on their own.
5. **Special Circumstances Handling**
   - **Baby teeth are about to be replaced**: If baby teeth are about to fall out, complex treatment may not be necessary.
   - **Degree of cooperation**: If the child is scared or uncooperative, special management techniques or light sedation may be needed.
6. **Post-treatment attention**
   - **Regular check-ups**: After treatment, you should visit your dentist regularly to check the condition of the restorations and the state of your oral hygiene.
   - **Observe the reaction**: Pay attention to whether your child has a reaction to tooth pain or discomfort and communicate with the dentist in a timely manner.
7. **Psychological support**
   - **Reduce anxiety**: Use gentle language and behavior to help your child reduce fear and anxiety about treatment.
   - **Positive encouragement**: Give your child positive encouragement and praise them for their bravery in the treatment process.

The above steps and precautions are based on the current standard of dental treatment and precautions. The purpose of treating caries in baby teeth is not only to solve the current problem, but also to prevent future oral problems and ensure that the child's oral health is maintained in the long term. Through the detailed description of the above steps, we hope to help parents and children better understand the treatment process of caries in primary teeth, and take appropriate measures to protect their children's oral health.

1. Does local anesthetic injections affect brain development in children?

In response to your question about whether local anesthetic injections in children affect brain development, I will elaborate on the following key points:

1. **Definition and mechanism of action of local anesthetic drugs**:
   - Local anesthetic drugs are drugs used to temporarily eliminate sensation in a certain part of the body, which achieve anesthetic effect by blocking the transmission of nerve signals, so that patients do not feel pain during surgery or other medical procedures.
   - These drugs usually act on peripheral nerves and do not directly affect the central nervous system of the brain.
2. **Sensitivity of children's brain development**:
   - In childhood, especially in newborns and infants, the brain is in a stage of rapid development and is more sensitive to various external factors than adults.
   - At this stage, the brain's synapses are being formed and trimmed, and neural pathways are constantly being built and optimized.
3. **Potential effects of local anesthetic drugs on brain development in children**:
   - Certain studies have shown that local anesthetic drugs may affect the survival and development of nerve cells, especially during critical periods of brain development.
   - Long-term or high-dose local anesthesia may cause some effects on the nervous system, but short-term and moderate use is generally considered safe.
4. **Disagreements and consensus in scientific research**:
   - At present, there is no complete consensus in the scientific community on the effects of local anesthetic drugs on children's brain development.
   - Some animal experiments have shown that local anesthetic drugs may have adverse effects on brain development in animal models, but these results cannot be directly generalized to humans.
   - The long-term effects of clinical studies in children are not sufficient, and more research is needed to determine.
5. **Considerations in clinical practice**:
   - In clinical practice, doctors consider the necessity and potential risks of using local anesthetic drugs.
   - For necessary surgery and treatment, the benefits of avoiding pain may far outweigh the potential risks of local anesthesia.
   - Doctors try to use the smallest effective dose and monitor the patient's response.
6. **Communication between parents and medical professionals**:
   - Parents should communicate adequately with a medical professional when their child undergoes any medical procedure.
   - It is important to understand why local anesthetic drugs are used, the possible risks, and the expected effects.
   - If in doubt, parents should seek a second opinion and consider other possible treatment options.
7. **Summary**:
   - Although there are some studies that suggest that local anesthetic drugs may have a potential effect on brain development in children, the current evidence is insufficient to draw definitive conclusions.
   - The use of local anesthesia when necessary is a routine practice in medical practice and is generally considered safe, especially under the supervision of a medical professional.
   - Parents should discuss all treatment options with their doctor and make a decision after understanding all potential risks and benefits.

Considering the above key points, although the effect of local anesthetic drugs on children's brain development is still an issue that needs further research, in medical practice, the use of such drugs is usually carried out after weighing the pros and cons. It is crucial for parents to be informed and communicate well with their doctors.

1. Does root canal treatment for deciduous teeth affect permanent teeth?

First of all, as a patient, it is very important to understand the impact of root canal treatment on permanent teeth of deciduous teeth. In answering this question, I will provide a detailed explanation based on my existing dental knowledge and clinical experience.

1. **Function and importance of baby teeth**:
   - Baby teeth not only help children chew food, but they also play an important role in pronunciation and maintaining the space for permanent teeth to erupt.
   - The premature loss of baby teeth can cause adjacent teeth to displace, which in turn affects the alignment of permanent teeth.
2. **Purpose of root canal treatment for deciduous teeth**:
   - Designed to clear the infection, the baby tooth is preserved until it falls out naturally, providing space and guidance for the normal eruption of the permanent teeth.
3. **Potential effects of root canal treatment of deciduous teeth on permanent teeth**:
   - Properly done root canal treatment usually does not negatively affect permanent teeth.
   - Mistakes during treatment, such as the overuse of chemical disinfectants, can cause damage to the underlying germ of the permanent tooth.
4. **Steps and precautions for root canal treatment**:
   - Evaluation: Treatment is determined by clinical examination and x-rays.
   - Cleaning: Removes diseased pulp and bacteria.
   - Formation: Shaping the root canal for filling.
   - Perfusion: Fill the root canal with biocompatible material.
   - Closure: Restore the form and function of the crown.
   - Note: Care should be taken to protect the underlying germ of the permanent tooth.
5. **Post-treatment monitoring**:
   - Regular follow-up: monitor the condition of baby teeth and the development of permanent teeth.
   - X-rays: to make sure the germ of the permanent tooth is not damaged.
6. **Success rate and possible risks of root canal treatment of deciduous teeth**:
   - Success rate: Root canal treatment has a high success rate under the operation of a professional dentist.
   - Risks: Improper treatment may lead to pigmentation or dysplasia of permanent teeth.
7. **Precautionary measures**:
   - Prompt treatment: Decay in primary teeth should be treated as soon as possible to reduce the need for root canal treatment.
   - Oral hygiene: Maintain good oral hygiene habits to prevent dental diseases.
8. **Roles of Parents and Patients**:
   - Parents should help their children develop good oral hygiene habits.
   - The child should cooperate with treatment and regular follow-up.

To summarize the above key points, root canal treatment of deciduous teeth is a common dental treatment modality used to deal with pulp lesions in deciduous teeth. When performed correctly, this treatment not only relieves pain, but also preserves the function of baby teeth, creating the conditions for a healthy eruption of permanent teeth. However, there are certain risks associated with any medical procedure, so it is crucial to choose an experienced dentist for treatment and follow their follow-up care recommendations. Regular dental check-ups and good oral hygiene practices can minimize the potential impact on permanent teeth.

1. Can a child's tooth be replanted?

To the question of whether a child's tooth can be replanted after a tooth is knocked out, here are detailed answers based on existing dental knowledge and practice:

1. **Judgment of tooth type**:
   - **The difference between baby teeth and permanent teeth**: The first thing you need to do is determine whether it is a baby tooth or a permanent tooth that is missing. Baby teeth are a child's first set of teeth that usually fall out naturally and are then replaced by permanent teeth. If a baby tooth is lost, it is usually not replanted because the permanent tooth grows naturally.
   - **In the case of permanent teeth**: If a permanent tooth is lost due to trauma or other reasons, reimplantation is possible, but the success rate and treatment effect depend on a variety of factors.
2. **Possibilities and conditions for replantation**:
   - **Tooth preservation**: Lost teeth need to be properly preserved as soon as possible, preferably in milk or moisturized with saline, which will help keep the tooth cells alive.
   - **Time factor**: Replantation should be carried out as soon as possible after tooth loss, preferably within 1 hour, the shorter the time, the higher the likelihood that the tooth will survive.
   - **Degree of damage to the tooth and alveolar bone**: The success rate of reimplantation is higher if the damage to the root of the tooth and alveolar bone is not severe.
3. **Steps and prognosis of replantation surgery**:
   - **Cleaning and treatment**: The doctor will first clean the missing tooth and the person's mouth to evaluate the damage to the tooth and socket.
   - **Immobilization**: The tooth is properly placed back into the socket and a fixation device (such as braces) is used to hold the tooth in place and promote the healing of the tooth and alveolar bone.
   - **Follow-up and treatment**: Regular follow-up examinations are required after replantation, and further treatments such as root canal treatment may be required.
   - **Prognostic assessment**: After successful reimplantation, it is also necessary to observe the stability and function of the tooth for a long time, as well as whether there are complications.
4. **Complications and Risks**:
   - **Tooth replantation failure**: Replanted teeth may fail for various reasons, such as the tooth failing to successfully join the alveolar bone.
   - **Perapical lesions**: Lesions such as apical periodontitis may occur and require further treatment.
   - **Dental stability issues**: Replanted teeth may not be as stable as natural teeth and require special attention.
5. **Long-term maintenance and precautions**:
   - **Oral hygiene**: Maintain good oral hygiene habits and have regular dental check-ups.
   - **Diet and lifestyle habits**: Avoid eating hard foods, do not open caps on teeth, etc., to reduce the adverse effects on replanted teeth.
   - **Regular check-ups**: Regular check-ups with the dentist to ensure the health of the replanted teeth.
6. **Alternatives**:
   - **Dental grafting**: If reimplantation is not successful, dental grafting or denture fitting may be considered.
   - **Dental implants**: Dental implants are an ideal alternative for patients of the right age, but because your child's jaw bone is still growing, it is often not possible to get implants until adulthood.

In summary, whether a child's tooth can be replanted after tooth loss depends on a variety of factors, including the type of tooth, the state of preservation and treatment, and the timeliness of treatment and replantation. It is advisable to contact a dentist for professional assessment and treatment as soon as possible after tooth loss. Throughout the treatment process, close attention to the recovery of the teeth is required, and appropriate oral hygiene measures as well as regular check-ups are required to ensure the best treatment outcome and prognosis.

1. Why do my child have recurrent mouth sores?

Canker sores are a common lesion of the oral mucosa that usually occurs on the lips, tongue, or inside of the mouth, and usually presents as a painful ulcer that is round or oval. There can be a variety of reasons why a child has recurrent mouth sores, but here are the key points after searching and regrouping based on the existing knowledge base:

1. **Nutritional deficiencies**
   - Iron deficiency: Iron is an essential element for the synthesis of hemoglobin, and iron deficiency may lead to the blockage of the metabolism of cells in the oral mucosa, which can lead to ulcers.
   - Deficiency of vitamin B complex: Deficiencies of vitamins B1, B2, B6, and B12 can lead to oral mucosal lesions.
   - Zinc deficiency: Zinc is a component of several enzymes, and zinc deficiency can affect cell repair ability.
2. **Immune system problems**
   - Autoimmune diseases: Autoimmune diseases such as systemic lupus erythematosus can cause recurrent mouth sores.
   - Weakened immunity: When the body's resistance is weakened, the oral mucosa is more susceptible to bacteria and viruses.
3. **oral hygiene**
   - Poor oral hygiene habits: Incorrect brushing or irregular oral hygiene can lead to a build-up of bacteria that can cause ulcers.
   - Toothbrush hardness: Using a hard-bristled toothbrush may scratch the oral mucosa and form ulcers.
4. **Microbial infections**
   - Viral infections: Infections such as herpes simplex virus can cause recurrent mouth sores.
   - Bacterial infections: Certain bacterial infections can also cause mouth sores.
5. **Digestive disorders**
   - Gastrointestinal problems: Gastrointestinal problems such as Helicobacter pylori infection, inflammatory bowel disease, etc., may be associated with mouth sores.
6. **Psychological factors**
   - Stress: Long-term mental stress or anxiety can lead to a decrease in the body's resistance and a tendency to mouth sores.
   - Lack of sleep: Chronic sleep deprivation can affect immune system function and increase the risk of mouth sores.
7. **Genetic factors**
   - Family history: Canker sores are common in some families and may have a genetic predisposition.
8. **Medication side effects**
   - Certain medications: Such as nonsteroidal anti-inflammatory drugs (NSAIDs) and certain antihypertensive drugs can cause mouth sores.
9. **Local irritation**
   - Physical irritation: Long-term friction of the oral mucosa by physical factors such as braces and aligners may lead to ulcers.
   - Chemical irritation: For example, the ingredients in some toothpastes may cause allergic reactions in some people.
10. **Changes in hormone levels**
    - Hormonal changes: Changes in hormone levels during puberty can affect the health of the oral mucosa.
11. **Bad habits**
    - Eating habits: Spicy, excessively hot or hard foods can damage the oral mucosa.
    - Smoking and alcohol consumption: may cause damage to the cells of the oral mucosa and increase the risk of ulcers.

For children with recurrent mouth ulcers, it is recommended that parents take their children to the hospital for a detailed examination to determine the specific cause and carry out targeted treatment based on the doctor's diagnosis and recommendations. At the same time, parents should pay attention to improving their children's dietary structure to ensure adequate nutritional intake; develop good oral hygiene habits; Reduce your child's psychological stress and ensure adequate sleep; Avoid oral hygiene products that may cause irritation; And pay attention to observe whether the child has bad oral habits, such as lip biting, lip licking, etc., and correct them in time.

1. Why can't the baby's tongue stick out or have a "groove" when it sticks out? Do I need surgery?

Regarding the situation where the baby's tongue does not stick out or has a "groove" when it sticks out, the following answers have been reassembled according to the knowledge I have searched, arranged by key points:

1. Tongue-tie shortness (tongue-tie tension)
   - Tongue-tie is a thin piece of tissue that connects the base of the tongue to the floor of the mouth.
   - When tongue-tie is too short or too tight, it restricts the movement of the tongue and may cause the tongue to not stick out or to have a deep middle groove when it sticks out.
   - This condition is medically known as tongue-tie or tongue-tie tension (ankyloglossia).
2. Effects and symptoms
   - Tongue-tie may affect the baby's breastfeeding as the baby may not be able to suck properly.
   - It may cause the baby to gain weight slowly or the mother to feel pain while breastfeeding.
   - As you grow up, it can affect the development of pronunciation or language skills.
3. diagnosis
   - Diagnosis is usually made by a pediatrician, dentist, or speech therapist.
   - Diagnosis is based on the length of the tongue-tie, the range of motion of the tongue, and the baby's breastfeeding.
4. treat
   - If tongue-tie doesn't have a significant effect on your baby's breastfeeding or language development, treatment may not be needed.
   - If the impact is significant, your doctor may recommend minor surgery, called frenotomy or frenuloplasty.
   - Surgery is usually simple and low-risk and can be done on an outpatient basis.
5. Surgical determinants
   - The decision to have surgery depends on your baby's specific condition and the severity of your symptoms.
   - The doctor will take into account the baby's breastfeeding, weight gain, tongue-tie shape and parents' wishes.
6. Recovery after surgery
   - Recovery after surgery is usually rapid, and most babies return to normal breastfeeding behaviour soon after surgery.
   - There may be brief discomfort or bleeding, but these usually subside quickly.
7. Non-surgical treatment
   - Speech therapy may be considered if the risk of surgery is high or if the parent chooses nonsurgical treatment.
   - Speech therapy can help improve tongue movement and speech articulation.
8. Parental support
   - Parents should be properly fed and cared for under the guidance of a doctor.
   - When deciding whether or not to undergo surgery, parents should actively communicate with their doctor about all treatment options and possible outcomes.
9. Track and Trace
   - Regardless of whether surgery is performed or not, your baby should be followed up regularly to ensure that his or her feeding and language development are normal.
   - If any problems arise, you should contact your doctor promptly.
10. Prevention and education
    - There is currently no known preventive measure to avoid tongue-tie shortening.
    - Parents should be educated about the effects of tongue-tie and how to treat it so that they can seek help if their baby has problems.

In summary, the baby's tongue does not stick out or has a "groove" when it sticks out may be caused by a short tongue tie. This condition needs to be diagnosed by a medical professional, and the need for surgery depends on the baby's specific condition and the severity of symptoms. Parents should keep in touch with their doctor about all treatment options and have regular follow-up check-ups to ensure the healthy development of their baby.

1. **questions related to oral prevention**
2. What is Oral Health?

Oral health refers to the absence of diseases and discomforts inside the mouth, including teeth, gums, upper and lower jaws, tongue, lips, oral mucosa, salivary glands, and surrounding tissues. A healthy mouth is not only able to chew, swallow and communicate normally, but it also has an important impact on overall health and quality of life. Here are the key points about oral health:

1. **Dental Health:**
   - A. No caries (tooth decay): Regular cleaning and check-ups, reducing the intake of high-sugar foods and beverages.
   - B. Proper brushing technique: Use fluoride toothpaste and brush your teeth at least twice a day for at least two minutes each time.
   - C. Floss: Floss daily to remove food debris and plaque from between your teeth.
2. **Gum Health:**
   - A. No gingival inflammation: The gums are normal in color and there is no redness, swelling, bleeding or pain.
   - B. Regular cleaning: Prevent periodontal disease by removing tartar and plaque through professional cleaning.
3. **Oral mucosal health:**
   - A. No ulcers on the mucosa: Regularly check the oral mucosa to prevent ulcers and infections.
   - B. No pain or abnormal sensation: Keep the mucous membranes moist and avoid dryness and irritation.
4. **Tongue Health:**
   - A. Tongue Coating Cleaning: To keep your tongue clean, you can use a tongue scraper or toothbrush to gently scrape your tongue.
   - B. No abnormal hyperplasia: Regular self-examination, note the color and surface changes of the tongue.
5. **Saliva and salivary gland health:**
   - A. Normal saliva secretion: Maintain adequate water intake to promote saliva secretion.
   - B. No salivary gland inflammation: avoid eating too hard or irritating foods.
6. **Oral Function:**
   - A. Chewing function: Keep your teeth and gums healthy to maintain normal masticatory function.
   - B. Swallowing function: Regularly exercise the oral muscles to keep the swallowing function normal.
   - C. Language function: Aligned teeth help to articulate clearly.
7. **Oral Cancer Prevention:**
   - A. Avoid tobacco products: Smoking and chewing tobacco are major risk factors for oral cancer.
   - B. Limit alcohol intake: Excessive alcohol consumption also increases the risk of oral cancer.
   - C. Regular oral check-ups: early detection of any abnormal changes such as leukoplakia or erythema.
8. **Regular check-ups and professional care:**
   - A. Regular visits to the dentist: Dental check-ups and cleanings at least once a year.
   - B. Follow your dentist's advice: Take good care of your mouth according to your dentist's instructions.
9. **Diet and lifestyle habits:**
   - A. Healthy Diet: Consume foods rich in calcium and vitamins, such as dairy products, fresh fruits, and vegetables.
   - B. Reduce sugar intake: Cut back on sweets and beverages to reduce the risk of tooth decay.
   - C. Moderate exercise: Regular physical activity contributes to overall health, including oral health.
10. **Children's Oral Health:**
    - A. Breastfeeding: Breastfeeding contributes to the normal development of the child's mouth and maxillofacial area.
    - B. Children's oral hygiene education: cultivate children's correct oral hygiene habits from an early age.

Maintaining oral health is not only essential to an individual's daily life, but is also closely related to overall health. For example, there is an association between periodontal disease and heart disease, diabetes, and other diseases. Therefore, maintaining good oral hygiene habits, regular dental check-ups, and taking preventive measures are essential to maintain overall health.

1. What causes bad breath?

Bad breath, also known as bad breath, is a common symptom that can be caused by a variety of reasons. Here are some key points that can cause bad breath:

1. **Poor oral hygiene**:
   - Food Residues: Food particles remain in the teeth and mouth and can be broken down by bacteria to produce odors.
   - Plaque and tartar: Poor oral hygiene habits can lead to the formation of plaque and tartar, which are breeding grounds for bacteria that can produce foul odors.
   - Tongue coating: The accumulation of bacteria and food debris on the tongue can also cause bad breath.
2. **Diseases of the Oral** Cavity:
   - Periodontal disease: is caused by a bacterial infection around the teeth that can produce bad breath.
   - Tooth decay: Broken teeth can be a place for food residue and bacterial growth, leading to bad breath.
   - Oral infections: such as mouth ulcers, periodontal abscesses, etc.
3. **Dry mouth**:
   - Saliva reduction: Saliva helps clean the mouth and neutralize acids, reducing saliva (for example, due to taking certain medications or having an open mouth while sleeping) can cause bad breath.
4. **Eating habits**:
   - Strong foods: Sulfur-containing foods such as garlic and onions can enter the lungs through the bloodstream after digestion and have a special smell when exhaled.
   - Diet: A high-protein diet, excessive sugar intake, etc. may increase bad breath.
5. **Smoking and drinking**:
   - Tobacco: Smoking causes changes in the oral environment and increases bad breath.
   - Alcohol: Alcohol consumption can cause dry mouth and increase the risk of bad breath.
6. **Systemic diseases**:
   - Diabetes: People with uncontrolled diabetes may have a fruity taste in their breath, which is a sign of ketoacidosis.
   - Liver disease: Hepatic insufficiency can lead to a peculiar sweet smell in the breath.
   - Kidney disease: Kidney failure can cause urine odor in your breath.
   - Gastrointestinal problems: Bad breath can also be caused by gastroesophageal reflux disease (GERD).
7. **Medication side effects**:
   - Certain medications, such as antidepressants and antihistamines, can cause dry mouth or other side effects that can cause bad breath.
8. **Respiratory tract infections**:
   - Such as chronic sinusitis, tonsillitis, etc.
9. **Psychological factors**:
   - Psychological states such as stress and anxiety may also indirectly contribute to bad breath as they may affect saliva production.
10. **Habits**:
    - Lack of sleep: Staying up late for long periods of time and not getting enough sleep can affect the body's metabolism and can lead to bad breath.
    - Excessive dieting: Skipping food for a long time can lead to an increase in bacteria in the mouth, causing bad breath.

All of the above points are the causes that can cause bad breath. If you're experiencing this problem, it's a good idea to start by improving your oral hygiene habits, such as brushing and flossing regularly, and visiting your dentist for regular check-ups and cleanings. At the same time, pay attention to dietary habits and lifestyle modifications, such as quitting smoking and limiting alcohol intake. If bad breath persists, it is advisable to consult a doctor or dentist to determine if there is an underlying health problem.

1. What to do about bad breath?

Bad breath, medically known as halitosis, is a common problem that can take a toll on an individual's social and mental health. Dealing with bad breath often requires a comprehensive approach, and here are some key points and explanations:

1. **Oral Hygiene Improvement**:
   - (a) **Brush your teeth regularly**: use fluoride toothpaste for at least two minutes twice a day.
   - (b) **Flossing**: At least once a day to remove food debris and bacteria from between your teeth.
   - (c) **Tongue cleaning**: Use a tongue scraper or toothbrush to gently scrape the tongue surface to remove the tongue coating.
   - (d) Regular **dental check-ups**: visit the dentist every 6 months to 1 year for professional cleaning and check-ups.
2. **Dietary modifications**:
   - (a) **Reduce irritating foods**: such as garlic, onions, etc., which may cause short-term bad breath.
   - (b) **Avoid dryness**: Drinking plenty of water and keeping your mouth moist can help remove food debris and bacteria from your mouth.
   - (c) Healthy eating: Increase your intake of fruits and vegetables, which contain fibre that can help clean your teeth.
3. **Lifestyle Adjustments**:
   - (a) **Smoking cessation and alcohol restriction**: Both tobacco and alcohol can cause dry mouth and increase the risk of bad breath.
   - (b) **Regular work and rest**: Get enough sleep and reduce stress, which contributes to overall health, including oral health.
4. **Check for physical ailments**:
   - (a) **Gastrointestinal problems**: such as gastroesophageal reflux disease, which may cause bad breath and require medical evaluation and treatment.
   - (b) **Diabetes mellitus**: Uncontrolled diabetes can lead to specific halitosis that requires appropriate medical intervention.
   - (c) **Oral diseases**: Periodontal disease or other oral infections can also cause bad breath and require treatment by a dentist or specialist.
5. **Use Oral Fresheners**:
   - (a) **Mouthwash:** The use of antibacterial or zinc chloride-containing mouthwash can temporarily reduce bad breath.
   - (b) **Chewing gum or mints**: Sugar-free gum or mints can stimulate saliva production and help clean the mouth.
6. **Maintain good digestive health**:
   - (a) **Regular bowel movements**: Keep your bowels open and avoid constipation, which may lead to bad breath.
   - (b) **Intake of probiotics**: Moderate intake of probiotics, such as yogurt, can help maintain the balance of intestinal flora.
7. **Seek professional medical advice**:
   - If none of the above methods solve the problem, it is advisable to seek the help of a doctor to rule out other conditions that may be causing bad breath.

Before taking any action, the first thing to do is to identify the cause of bad breath. Sometimes, bad breath may simply be due to eating certain foods with a strong smell, such as garlic or onions, in which case bad breath is usually temporary. But if bad breath persists, it may be due to poor oral hygiene, certain medical conditions, medication side effects, a dry mouth (xerostomia), or other health problems.

Based on the above information, measures to address bad breath should be comprehensive, taking into account daily oral hygiene habits, dietary modifications, lifestyle changes, physical condition check-ups, and seeking professional medical advice if necessary. If you have tried these methods and bad breath persists, you should consult a dentist or doctor, as this can be a sign of some undiagnosed health problem.

1. How can I prevent tooth decay?

Preventing tooth decay is a multi-step process that involves daily living habits, oral hygiene habits, and regular oral health check-ups. Here is a series of key points and recommendations, organized and described in detail according to your requirements:

1. **Maintain good oral hygiene:**
   - Brush your teeth at least twice a day and use a fluoride toothpaste.
     - Fluoride can help strengthen the tooth surface and make it more resistant to acids.
   - Floss between your teeth at least once a day.
     - Flossing removes food debris and plaque from between your teeth that brushing can't reach.
   - Consider using a fluoride mouthwash.
     - Mouthwashes can further reduce bacteria in your mouth and provide additional protection against fluoride.
2. **Visit your dentist regularly for check-ups and cleanings:**
   - Dental check-ups and professional cleanings every 6 months.
     - Regular check-ups can detect and treat early tooth decay.
   - Follow your dentist's professional advice.
     - Your dentist may recommend additional precautions, such as fissure sealants or fluoride treatment.
3. **Dietary Adjustments:**
   - Reduce sugar and refined carbohydrates.
     - These foods are easily converted into acids by oral bacteria, which can erode tooth enamel.
   - Avoid frequent diets and snacks.
     - Frequent eating provides a constant source of food for bacteria, increasing acid production.
   - Increase your intake of foods containing calcium and phosphorus.
     - These minerals help in tooth remineralization and strengthening.
4. **Lifestyle changes:**
   - Quit smoking and limit alcohol intake.
     - Tobacco and excessive alcohol can reduce the mouth's ability to repair itself.
   - Manage chronic conditions, such as diabetes.
     - Certain diseases can increase the risk of tooth erosion.
5. **Use caries prevention aids:**
   - Consider using an electric toothbrush.
     - An electric toothbrush can remove plaque more effectively.
   - Use an interdental brush or water flosser.
     - These tools can help clean hard-to-reach areas.
6. **Education and Habit Building:**
   - Cultivate oral hygiene habits in children from an early age.
     - Good habits developed in childhood can last a lifetime.
   - Regular oral health education.
     - Learn how to brush and floss properly.
7. **Individualized precautions:**
   - Develop a personalized prevention plan based on the individual's risk factors.
     - For example, if there is a genetic predisposition in the family, more frequent visits to the dentist may be required.
8. **Maintain overall health:**
   - Maintain adequate hydration.
     - Saliva is essential for neutralizing the acidic environment of the oral cavity.
   - Maintain a balanced diet.
     - A balanced diet contributes to overall health, including oral health.

The above points are based on existing dental research and public health recommendations and aim to provide a comprehensive strategy for preventing dental caries. With the combination of these measures, the risk of tooth decay can be greatly reduced and the overall health of the oral cavity can be maintained.

1. What is the correct way to brush my teeth?

Brushing teeth is the foundation of oral hygiene, and proper brushing methods can effectively prevent oral diseases such as tooth decay and periodontal disease. Here are the detailed brushing steps and precautions based on dental expertise and guidance:

1. Choosing the right toothbrush and toothpaste:
   - Use a soft-bristled toothbrush to reduce damage to your gums.
   - Choose a toothpaste that contains fluoride, which helps prevent tooth decay.
2. Preparation before brushing:
   - Floss to remove food debris and plaque from between your teeth.
   - To clean your tongue, you can use the tongue scraper on the back of your toothbrush.
3. Steps to brush your teeth:
   - Place the toothbrush diagonally at the junction of your teeth and gums at an angle of about 45 degrees.
   - Brush your teeth with gentle circular motions and avoid brushing too hard laterally to avoid damaging your gums.
   - Brush your teeth in a certain order to make sure each area is cleaned: a. Start by brushing the outer side, starting with one side of the back teeth and gradually moving towards the opposite side. b. Brush the inner surface again, also starting from the back teeth, and pay attention to the inner surface brushing method to be more detailed. c. Brush the occlusal surface, the top of the tooth, where the food is chewed. d. Brush the inside of the tooth in front of the end of the last way, you can hold the toothbrush upright and make it easier to clean.
4. When and how often you brush your teeth:
   - Brush your teeth for at least 2 minutes each time, making sure each area is adequately cleaned.
   - Brush your teeth at least twice a day, once in the morning and once in the evening, especially before bed.
5. Steps after brushing:
   - After brushing, rinse your mouth with water to remove loose food debris and toothpaste foam from the brushing process.
   - Using a mouthwash can additionally help clean the mouth and eliminate bacteria, but it's not necessary.
6. Care and replacement of toothbrushes:
   - After each use, wash your toothbrush thoroughly and place it in a ventilated and dry place.
   - Replace your toothbrush every 3 to 4 months, or when the bristles start to deform.
7. Periodic Inspections:
   - Even if daily oral hygiene is done well, a dental check-up should be done at least once a year.

The above steps and precautions are compiled based on dental expertise, and following these guidelines can be effective in maintaining oral health. However, everyone's oral condition is different, and the brushing method may need to be adjusted according to the individual situation, such as periodontal patients or orthodontic patients. Therefore, it is advisable to consult with a dentist regularly for personalized oral hygiene guidance.

1. What are the types of toothpaste?

Toothpaste is an indispensable everyday item in oral hygiene, and it comes in many types for different oral concerns and personal preferences. Here's a closer look at the types of toothpaste that I searched and recombined based on my knowledge base:

1. **Fluoride toothpaste**
   - **Prevent tooth decay**: Fluoride toothpaste contains sodium fluoride or tin fluoride, which can help strengthen the enamel on the tooth surface to prevent tooth decay.
   - **Suitable for**: Suitable for most people, especially those who are at risk of tooth decay.
2. **Whitening toothpaste**
   - **Remove surface stains**: Whitening toothpastes often contain abrasives such as calcium carbonate or silicates to help remove stains from the surface of your teeth.
   - **Bleaching ingredients**: Some whitening toothpastes may also contain hydrogen peroxide or urea peroxide, which have a bleaching effect.
   - **Suitable for**: Suitable for people who want to improve the color of their teeth and remove stains.
3. **Anti-allergy toothpaste**
   - **Reduces sensitivity**: Contains ingredients such as potassium nitrate or strontium chloride to help relieve tooth sensitivity.
   - **Long-term effects**: With long-term use, it can help reduce tooth sensitivity to cold, heat, sour, and sweet reactions.
   - **Suitable for**: Suitable for patients with sensitive teeth.
4. **Toothpaste for the control of plaque and periodontal disease**
   - **Antimicrobial** ingredients: Contains antibacterial ingredients such as triclosan and chlorhexidine to help control the formation of plaque.
   - **Prevent periodontal disease**: Helps reduce the occurrence of gingivitis and periodontal disease.
   - **Suitable for**: Suitable for people with periodontal problems or those who want to prevent periodontal disease.
5. **Fluoride children's toothpaste**
   - **Low-fluoride formulations**: Children's toothpastes typically contain lower concentrations of fluoride and are suitable for children.
   - **Flavors**: In order to make children more willing to brush their teeth, there are often various fruit or candy flavors.
   - **Suitable for**: Specially designed for children, suitable for children over the age of 2.
6. **Natural/organic toothpaste**
   - **No chemical additives**: no fluoride, no preservatives, no artificial sweeteners and no colors.
   - **Natural Ingredients**: May contain natural ingredients such as tea tree oil, green tea extract, xylitol, etc.
   - **Suitable for**: Suitable for consumers looking for natural oral care products.
7. **Medicated/prescription toothpaste**
   - **Special Ingredients**: May contain higher concentrations of fluoride or other medicinal ingredients.
   - **Professional guidance**: This type of toothpaste usually needs to be used under the guidance of a dentist.
   - **Suitable for**: Suitable for patients with special oral problems that require professional treatment.
8. **Toothpaste to remove bad breath**
   - **Freshens Breath**: Contains ingredients such as peppermint to help improve breath.
   - **Long-lasting formula**: Some products may contain special ingredients that provide a fresher breath effect for longer.
   - **Suitable for**: Suitable for people who have bad breath problems or want to keep their breath fresh.

When choosing a toothpaste, you should consider your own oral health and personal preferences. For example, if you have tooth sensitivity issues, then anti-sensitivity toothpaste may be a good choice. If you're looking for a whitening effect, whitening toothpaste may be more suitable for you. For children, it is important to choose a low-fluoride toothpaste that is appropriate for their age and taste. If you have specific oral health problems, it is best to choose a toothpaste on the advice of your dentist.

The choice of each toothpaste should be based on knowledge of the ingredients and consideration of the individual's oral health. The efficacy of toothpaste is closely related to an individual's oral hygiene habits, brushing skills, and regular dental check-ups. Proper use of toothpaste, along with good oral hygiene habits, can help maintain oral health.

1. How can pregnant women protect their oral health?

Pregnant women should pay special attention to their oral health during pregnancy, as they are more likely to develop gingivitis and periodontal disease due to changes in hormone levels during this time. Here are the answers based on the information I searched in my knowledge base, regrouped and arranged by key points:

1. **Regular dental check-ups**
   - A comprehensive oral examination should be done before pregnancy to deal with oral problems in a timely manner.
   - It is recommended to have at least one dental check-up during pregnancy to identify and treat potential problems.
   - Inform your dentist about your pregnancy status so that you can take appropriate precautions and treatments.
2. **Good daily oral hygiene habits**
   - Brush your teeth morning and night, and floss between your teeth after each meal.
   - Brush your teeth with a fluoride toothpaste to prevent tooth decay.
   - Choose a soft-bristled toothbrush when brushing your teeth to reduce irritation to sensitive gums.
   - If vomiting occurs, it is recommended to add 1 teaspoon of baking soda to 1 cup of water, rinse your mouth with this solution, and then brush your teeth again to neutralize the erosion of stomach acid on your teeth.
3. **Eat right**
   - Increase your intake of calcium, such as milk, cheese and green leafy vegetables, to enhance the health of your teeth.
   - Avoid foods and drinks high in sugar to reduce the risk of tooth decay.
   - Maintain a balanced diet and ensure adequate intake of vitamins and minerals to support oral health.
4. **Control morning sickness**
   - Morning sickness may cause stomach acid to come into contact with your teeth more often, increasing the risk of corrosion.
   - Try a loose diet and eat smaller, more frequent meals to reduce morning sickness symptoms.
   - If morning sickness is severe, you should consult your doctor for help.
5. **Avoid certain oral hygiene products**
   - Avoid using alcohol-based mouthwashes, as alcohol intake should be reduced during pregnancy.
   - Oral care products with natural ingredients to reduce the potential risk of chemicals to the fetus.
6. **Quit smoking and drinking**
   - Both smoking and drinking alcohol increase the risk of oral diseases and have serious effects on fetal health.
   - Seek professional help to quit bad habits.
7. **Dealing with gum problems during pregnancy**
   - If you experience swollen, painful or bleeding gums, you should consult your dentist promptly.
   - Brush and floss gently to avoid additional damage to your gums.
8. **Avoid oral punctures**
   - Oral piercing should be avoided during pregnancy as it may increase the risk of infection.
9. **Be mindful of drug use**
   - Always consult your doctor or dentist before using any medications, including topical oral medications.
   - Avoid medications that can be harmful to the fetus.
10. **Stay hydrated**
    - Dry mouth is easy to drink during pregnancy, so drinking plenty of water can help keep your mouth moist and reduce bacterial growth.
11. **Education and access to information**
    - Learn about oral health during pregnancy and learn how to prevent and deal with common problems.
    - Attend pregnancy health classes to exchange experiences with other pregnant women.

Through the above measures, pregnant women can effectively protect their oral health and lay a good foundation for the health of the fetus. These recommendations are based on current medical knowledge and best practices, but each individual's situation may be different, so it's best to consult your own dentist or doctor before taking any action.

1. What is Oral Preventive Care?

Preventive oral care is a set of measures and practices aimed at preventing the onset and progression of oral diseases and maintaining and promoting oral health. Here's what I've searched, collated and combined with key information about preventive oral care, based on your requirements:

1. **Daily oral hygiene maintenance**:
   - **Brush your teeth properly**: Use fluoride toothpaste and a toothbrush of appropriate firmness at least twice a day for at least two minutes each time.
     - *Tip note*: The correct brushing method involves brushing the toothbrush at a 45-degree angle to the tooth and cleaning the tooth surface in small circular motions.
   - **Floss**: Floss at least once a day to remove food debris and plaque from between your teeth.
     - *Note*: Flossing correctly can be effective in preventing periodontal disease and tooth decay.
2. **Regular oral check-ups**:
   - **Regular visits to the dentist**: It is advisable to have an oral check-up every six months for early detection and treatment of oral problems.
     - *Note*: Regular check-ups can help detect common diseases such as tooth decay and periodontal disease.
3. **Dietary Adjustments**:
   - **Reduce sugar intake**: High-sugar foods and beverages can easily lead to tooth decay, so you should consume them in moderation and pay attention to a balanced diet.
     - *Tip note*: Sugar is the main food source for bacteria, and when bacteria metabolize sugar, they produce acids that can corrode teeth and cause tooth decay.
   - **Eat a balanced diet**: Make sure you're getting enough vitamins and minerals to help maintain healthy teeth and gums.
     - *Bottom line*: Calcium and phosphorus are important minerals that make up your teeth, and vitamin C helps keep your gums healthy.
4. **Quit smoking and limit alcohol:**
   - **Avoid tobacco products**: Smoking increases the risk of oral cancer, periodontal disease, and tooth discoloration.
     - *Notes*: Harmful substances such as nicotine and tar in tobacco have an irritating and damaging effect on the oral mucosa.
   - **Drink alcohol in moderation**: Excessive alcohol consumption can also increase the risk of oral cancer.
5. **Protective measures**:
   - **Using oral care products**, such as an antibacterial mouthwash, can reduce the buildup of bacteria.
     - *A small note*: Mouthwash can help clean areas that are difficult to clean with a toothbrush and dental floss.
   - **Use a mouth guard while exercising**: Use a mouth guard to protect your teeth from damage when participating in sports activities that can cause oral injuries.
6. **Oral Health Care for Special Populations**:
   - **Children**: Childhood is a critical period for developing good oral hygiene habits, and attention should be paid to children's oral health education.
     - *Note*: Although children's baby teeth will be replaced, the health of baby teeth will affect the health of permanent teeth.
   - **Pregnant women**: Pregnant women are more likely to have their gums become inflamed due to hormonal changes, so oral hygiene should be strengthened.
   - **Elderly**: As we age, oral problems such as xerosporia, periodontal disease, etc., may increase and require special attention.
7. **Education & Advocacy**:
   - **Raise awareness of oral health**: Raise public awareness of oral health through education and awareness campaigns.
     - *Explanation*: Understanding the dangers and prevention of oral diseases is essential to promote oral health for individuals and society.
8. **Early Intervention of the Disease**:
   - **Prompt treatment**: For early oral diseases, such as mild caries and gingivitis, treatment should be done as soon as possible to avoid worsening of the condition.

The above key points outline the main elements of preventive oral care. These measures aim to build a comprehensive oral health care system through daily maintenance, regular check-ups, adjustment of lifestyle habits, special attention to different populations, as well as education and early intervention. By implementing these measures, it is possible to effectively prevent oral diseases, improve the quality of life, and reduce the physical and financial burden caused by oral problems.

1. How to maintain oral hygiene?

Maintaining oral hygiene is essential for maintaining overall health. Here are some of the key points on how to maintain your oral hygiene, based on what I've learned:

1. **Brush your teeth regularly**
   - Use a fluoride toothpaste and brush your teeth at least twice a day for at least two minutes.
     - Fluoride helps prevent tooth decay and can strengthen teeth.
   - Use proper brushing techniques.
     - Aim your gums at a 45-degree angle and gently brush your teeth back and forth over short distances.
   - Choose the toothbrush that's right for you.
     - A small-headed toothbrush makes it easier to clean the back teeth.
2. **Floss**
   - Floss at least once a day to remove food debris and plaque from between your teeth.
     - Proper flossing can prevent periodontal disease and bad breath.
3. **Use mouthwash regularly**
   - Choose a mouthwash that contains antimicrobial ingredients to help reduce bacteria in your mouth.
     - Mouthwash can reach places that are hard to reach for brushing and flossing.
4. **Eating habits**
   - Reduce your intake of sugar and acidic foods.
     - These foods promote the growth of bacteria in the mouth and increase the risk of tooth decay.
   - Eat plenty of fiber-rich foods.
     - Increases saliva production and helps clean teeth naturally.
5. **Quit smoking and limit alcohol**
   - Smoking and excessive alcohol consumption increase the risk of oral cancer and periodontal disease.
6. **Regular check-ups**
   - Dental check-ups and teeth cleanings are done every six months.
     - Problems can be detected early and treated.
7. **Keep your mouth moist**
   - Avoid dry mouth, as saliva helps reduce oral bacteria.
     - Drink water or use saliva substitutes.
8. **Use the right oral hygiene products**
   - Choose oral hygiene products that are right for you, such as toothpaste, toothbrush, and dental floss.
     - A dentist can be consulted for recommendations.
9. **Protect your teeth**
   - Use a dental protector when exercising.
     - Prevents tooth damage caused by impact.
10. **Avoid using your teeth to do things that aren't appropriate**
    - Do not use your teeth to open the cap or bite hard objects.
      - This avoids unnecessary damage to the teeth.
11. **Proper use of oral aids**
    - If you use a toothpick, be gentle to avoid damaging your gums.
      - Use a toothpick to clean between your teeth, but you shouldn't push too hard.
12. **Maintain a healthy lifestyle**
    - Get enough sleep and exercise.
      - This helps to improve the body's overall resistance and reduce oral problems.
13. **Manage chronic conditions**
    - Control chronic diseases such as diabetes, as they may affect oral health.
      - Chronic diseases may weaken the body's antimicrobial function and increase the risk of oral infections.

These key points are based on extensive medical research and best practices in dental hygiene. Maintaining oral hygiene is not only about your teeth, but also about the health of your whole body. Through daily care and regular professional check-ups, oral diseases can be effectively prevented and oral health can be maintained.

1. What is the relationship between oral diseases and systemic diseases?

There are complex associations between oral diseases and systemic diseases, and these relationships are reflected at different levels and mechanisms. Here are the key takeaways based on existing scientific research and medical knowledge:

1. **Cardiovascular disease**:
   - 1.1. Studies have shown that people with periodontal disease may be more likely to develop cardiovascular disease.
   - 1.2. Periodontal disease may lead to impaired vascular endothelial function and atherosclerosis, which increases the risk of heart disease and stroke.
   - 1.3. Inflammation in the mouth can be transmitted through the blood circulation, activating an inflammatory response throughout the body.
2. **Diabetes**:
   - 2.1. There is a two-way relationship between oral diseases, especially periodontal disease, and diabetes.
   - 2.2. People with diabetes are more likely to develop periodontal disease because diabetes weakens the immune system, making the mouth more susceptible to infections.
   - 2.3. Periodontal disease can exacerbate diabetes because it can increase blood sugar levels, making diabetes more difficult to control.
3. **Respiratory diseases**:
   - 3.1. Poor oral health, especially periodontal disease, may increase the risk of pneumonia and chronic obstructive pulmonary disease (COPD).
   - 3.2. Bacteria in the mouth can enter the lungs through inhalation and cause infection.
4. **Pregnancy complications**:
   - 4.1. Periodontal disease in pregnant women may increase the risk of preterm birth and low birth weight babies.
   - 4.2. Periodontal disease is associated with inflammation during pregnancy, which may affect the uterus and fetus.
5. **Digestive Disorders**:
   - 5.1. Oral health has a direct impact on the chewing and digestion of food.
   - 5.2. Tooth loss or periodontal disease can lead to indigestion because food is not chewed adequately.
6. **Immune System Disorders**:
   - 6.1. Periodontal disease may activate or exacerbate autoimmune diseases such as rheumatoid arthritis.
   - 6.2. Inflammation in the mouth can lead to an overactive immune system, causing inflammation in other areas.
7. **Cancer**:
   - 7.1. Periodontal disease is associated with an increased risk of certain types of cancer, such as cancers of the mouth, larynx, esophagus, and pancreas.
   - 7.2. Inflammation and bacteria in the oral cavity may be implicated in cancer development by promoting an inflammatory response or directly damaging cellular DNA.
8. **Osteoporosis**:
   - 8.1. Periodontal disease can lead to the loss of alveolar bone, which is associated with generalized osteoporosis.
   - 8.2. People with osteoporosis may be more susceptible to periodontal disease because osteopenia weakens the supporting structures of the teeth.
9. **Kidney disease**:
   - 9.1. Poor oral health is associated with the development of chronic kidney disease.
   - 9.2. Periodontal disease may affect kidney function through a chronic inflammatory process.
10. **Mental Health**:
    - 10.1. Oral diseases can affect mental health by causing pain, self-esteem problems, and social difficulties.
    - 10.2. Some mental health problems, such as depression and anxiety, can lead to negligence in oral hygiene, which in turn exacerbates oral problems.

Summarizing the above key points, it can be seen that oral health and general health are closely related. Oral diseases not only affect localized teeth and gums, but can also affect overall health through a variety of mechanisms. Therefore, maintaining good oral hygiene habits and having regular dental check-ups are very important for the prevention and management of systemic diseases.

1. **problems related to dental restorations and dental implants**
2. Do I need bone grafting before dental implants?

Whether or not a bone graft is needed before a dental implant is a common question, especially for those who are missing teeth and are considering dental implants. Here is a detailed answer to this question based on your requirements:

1. **The importance of alveolar bone condition**:
   - Dental implants are the process of placing root-shaped implants in the alveolar bone and then fixing dentures on top.
   - Alveolar bone is the bone that supports the tooth, and its health is critical to the success of dental implants.
   - If the alveolar bone is not large or dense enough, the implanted root may not be stable, affecting the success rate and long-term stability of the dental implant.
2. **Causes of bone grafting**:
   - Bone grafting is a surgical procedure to increase the amount of alveolar bone and is often used in cases of alveolar bone degeneration or loss.
   - After a tooth is missing, the alveolar bone may begin to atrophy because there is no stimulation from the tooth to maintain its volume and density.
   - Atrophy of alveolar bone can be the result of prolonged tooth loss, periodontal disease, trauma, or other systemic diseases.
3. **Types of bone grafting**:
   - Autologous bone grafting: The patient's own bone is used, usually from the mandible or ilium.
   - Allogeneic bone grafting: The use of treated human donor bone.
   - Xenotransplantation: The use of treated animal bones.
   - Synthetic materials: Use biologically active or biocompatible synthetic materials.
4. **Assess the need for bone grafting**:
   - Imaging tests (eg, X-rays, CT scans) are used to assess the height, width, and density of alveolar bone.
   - An oral exam to assess periodontal health and other factors that may affect dental implants.
   - A thorough health assessment, including medical history, medication use, and lifestyle habits, as these factors may affect bone healing.
5. **The process of bone grafting surgery**:
   - Bone graft surgery is usually performed under local anesthesia.
   - Surgery may involve cutting the gums to expose areas that need to increase bone mass, then placing bone graft material and closing the gums with sutures.
   - It usually takes several months to heal after a bone graft for the transplanted bone to fuse with its own bone.
6. **Risks and considerations of bone grafting**:
   - Surgical risks such as infection, bleeding, rejection or displacement of bone graft material.
   - The long healing time may delay the entire process of dental implants.
   - Cost and insurance coverage issues, as bone grafting and dental implant surgeries can be quite expensive.
7. **Post-operative recovery and care**:
   - Follow your dentist's instructions, including oral hygiene maintenance, regular check-ups, and taking any necessary medications.
   - During bone healing, it may be necessary to avoid putting pressure on the surgical area or eating hard food.
8. **Long-term prognosis**:
   - After a successful bone graft, the success rate of dental implants is significantly improved, providing similar function and appearance to natural teeth.
   - Regular oral health care and dentist visits help maintain the long-term success of dental implants.

In summary, the need for bone grafting depends on the individual's specific circumstances, including the quality and quantity of alveolar bone, as well as the patient's overall health. If the alveolar bone is not enough to support the implant, bone grafting surgery may be necessary. Bone grafting can improve the success rate of dental implants, but it also comes with additional surgical risks, costs, and recovery time. Therefore, when deciding whether or not to undergo a bone graft, a detailed discussion should be held with a dental professional to ensure that all the risks, benefits, and available alternatives are understood.

1. What are the consequences of missing teeth not being repaired?

Missing teeth can lead to a range of oral health problems and a reduced quality of life. Here are the answers after searching and reassembling them based on my knowledge base, arranged by key points:

1. **Tooth displacement**
   - After missing a tooth, adjacent teeth may tilt or move into the space where the missing tooth is missing, causing the teeth to align out of alignment.
   - This displacement may affect the occlusal relationship, which is how the upper and lower teeth are butted, which in turn can lead to less efficient chewing.
2. **Difficulty chewing**
   - Missing teeth reduce the chewing surface, making it difficult to chew food.
   - This can lead to digestive problems as food is swallowed without being chewed enough.
3. **Voice issues**
   - Teeth play an important role in the pronunciation process, especially the "F" and "S" sounds.
   - Missing teeth may cause unclear pronunciation and interfere with communication.
4. **Jaw atrophy**
   - The jaw bone in the edentulous area does not receive normal chewing stimulation and may gradually atrophy.
   - Jaw atrophy not only alters the facial contour, but may also lead to alveolar bone loss, affecting future dental restoration options.
5. **Changes in facial appearance**
   - Long-term tooth loss can cause facial muscles to relax, causing facial contours to change.
   - This can make the person appear older than they really are.
6. **Psychological effects**
   - Missing teeth can lead to a decrease in self-confidence, especially in social situations.
   - Some people may avoid smiling or talking because they are missing teeth, affecting their mental health and social life.
7. **Increased risk of periodontal disease**
   - Edentulous areas are more likely to accumulate food debris and bacteria, increasing the risk of periodontal disease.
   - Periodontal disease not only affects oral health, but is also associated with systemic diseases such as heart disease and diabetes.
8. **Further tooth loss**
   - Missing teeth can cause additional stress on the remaining teeth, accelerating tooth wear and potential tooth loss.
9. **Inadequate nutrient intake**
   - Because chewing is difficult, it can lead to a preference for soft, easy-to-chew foods, which can reduce nutrient-dense food intake.
   - This can lead to malnutrition, affecting overall health.
10. **Financial burden**
    - In the long run, missing teeth can lead to other dental problems that ultimately require more complex treatment and increase the financial burden.
11. **Treatment becomes more difficult**
    - Over time, if a decision is made to restore a tooth, it may become more difficult and complicated to treat due to jaw atrophy or other complications.

In summary, missing teeth not only affect oral health, but can also have a negative impact on quality of life, mental health, and economic well-being. Therefore, if there is a missing tooth, it is advisable to consult a dentist in time to understand the suitable restoration options to avoid the above problems.

1. Do fixed crowns need to be replaced regularly?

On the question of whether fixed crowns need to be replaced regularly, here are the answers after searching and reassembling according to my knowledge base:

1. **Crown lifespan**:
   - Dental crowns are usually designed to be used for long periods of time, but they are not permanent. Their average lifespan is around 5 to 15 years, but this time span can vary depending on factors such as material, oral hygiene habits, the size and precision of the bite force, and the frequency of regular dental check-ups.
2. **Material Influences**:
   - a. **Metal crowns**: Metal crowns, such as gold or other alloys, are generally more durable but less aesthetically pleasing.
   - b. **Porcelain fused metal crown** (PFM): This type of crown combines the strength of metal with the aesthetics of porcelain, but may need to be replaced due to crackage of porcelain.
   - c. **All-ceramic/all-ceramic crowns**: These crowns are aesthetically superior to metal crowns, but may be slightly inferior in terms of durability.
3. **Oral Hygiene Habits**:
   - Good oral hygiene habits are key to maintaining the longevity of a crown. Regular brushing, flossing, and mouthwash can reduce bacterial buildup around the crown and plaque formation, which can extend the life of the crown.
4. **Bite force and usage habits**:
   - Excessive bite force, such as grinding, clenching, or chewing hard objects with a crown, can cause premature crown damage. Regular visits to the dentist for bite adjustments can help reduce this risk.
5. **Regular inspection and maintenance**:
   - Regular dental check-ups are essential as dentists can monitor the condition of the crown and deal with minor problems in a timely manner to prevent them from developing into major problems, which also helps to prolong the life of the crown.
6. **Possible Problems and Reasons for Replacement**:
   - a. **Loose crown**: If the crown is loose, it may be due to a change in the underlying tooth structure or tooth decay in the tooth underneath the crown.
   - b. **Gum problems**: If the gums are receding, the crown edge may be exposed, which not only affects the aesthetics but can also lead to the accumulation of bacteria on the crown edge.
   - c. **Cosmetic considerations**: Over time, crowns may no longer meet the patient's aesthetic needs due to wear or color change.
   - d. **Structural damage**: If there is a crack or fragment of the crown, it must be replaced to prevent further damage or infection.
7. **Individual patient differences**:
   - Each patient's oral condition and lifestyle habits are different, so the lifespan of the crown will also vary. Some patients may find that their crowns are able to last longer, while others may need to be replaced more frequently.
8. **Summary**:
   - Crowns are not a one-and-done solution, and they can need to be replaced for a number of reasons. To maximize the longevity of the crown, patients should maintain good oral hygiene, avoid excessive bite behavior, and have regular dental check-ups.

From the above key points, we can understand that whether a fixed crown needs to be replaced regularly depends on a variety of factors, including material, oral hygiene habits, bite force, etc. Patients should work closely with their dentist to undergo regular check-ups and necessary maintenance to ensure the best condition and maximum longevity of the crown. Of course, if any problems arise or the patient has new requirements for the appearance or function of the crown, the dentist should also be consulted to determine if the crown needs to be replaced.

1. Is there an age limit for dental implants?

Regarding whether there is an age limit for dental implants, here's what I searched and collated based on my knowledge base:

1. **Consideration of the lower age limit**:
   - **Skeletal maturation**: Dental implants usually require that the patient's jaw development has been completed, which means that teenagers are often not candidates for dental implant surgery because their jaws may still be growing. In general, women need to reach about 18 years of age and men need to reach about 21 years of age.
   - **Tooth development**: Dental implants may affect normal tooth alignment and jaw growth during the developmental stage of the teeth and jaw structure in adolescents.
2. **Consideration of the upper age limit**:
   - **Physical health**: Theoretically, there is no definite upper age limit for dental implants, and the key lies in the overall health of the patient. Dental implants may also be considered for older patients if they are in good health, do not have serious chronic diseases, or are taking medications that may affect surgery.
   - **Bone condition**: As we age, bone may become more fragile, which may affect the stability and success of dental implants. Before getting a dental implant, the doctor will assess the density and quality of the patient's jawbone.
3. **Impact of overall health status**:
   - **Chronic medical conditions**: Patients with certain chronic medical conditions, such as diabetes or heart disease, may need to consider dental implants more cautiously under the guidance of their doctors.
   - **Effects of medications**: Taking certain medications, especially those that affect bone metabolism or blood clotting, may increase the risk of implant surgery.
4. **Importance of preoperative evaluation**:
   - **Oral health**: Before getting a dental implant, it is important to conduct a thorough assessment of the patient's oral health, including the condition of the gums, jaw health, and whether there is periodontal disease.
   - **Lifestyle habits**: Lifestyle habits such as smoking and drinking alcohol may affect the success rate of the surgery and the healing process.
5. **Success rate of dental** implants:
   - **Technological advancement**: With the advancement of medical technology, the success rate of dental implants has been very high, but it is still necessary to consider factors such as the patient's age, health status and lifestyle habits.
   - **Post-operative care**: Post-implant care is equally important, and good oral hygiene habits and regular dental follow-up are essential to ensure the long-term success of dental implants.
6. **Patient's expectations and quality of life**:
   - **Functional restoration**: The main purpose of dental implants is to restore the function and aesthetics of the teeth, which is of great significance for improving the quality of life of patients.
   - **Psychological effects**: Missing teeth can have a negative impact on a patient's self-confidence and social life, and dental implants can help improve these psychological factors.
7. **Economic and practical considerations**:
   - **Cost**: Dental implants are an expensive treatment and patients need to consider their own financial situation.
   - **Long-term investment**: Despite the large initial investment, dental implants are a long-term solution that usually lasts for many years or even a lifetime.

To sum up, there is no strict age limit for dental implants, but whether or not they are suitable for dental implants needs to consider the patient's jaw development, overall health, oral health, lifestyle habits and financial ability. It is advisable to communicate well with a professional dentist when considering dental implants to make the decision that is best for you.

1. What is the difference between removable dentures, porcelain teeth, and dental implants, and how to choose?

Removable dentures, porcelain teeth, and dental implants are the three most common types of dental restoration, each with different indications, advantages and disadvantages, and cost considerations. Here's a detailed comparison and selection suggestion based on my knowledge base search and integration:

1. Removable dentures (partial or full dentures can be removed)
   - Indications: Suitable for multiple teeth missing in the oral cavity, especially when the missing teeth are widely distributed or the whole mouth is missing.
   - Pros: a. The cost is relatively low. b. The manufacturing process is relatively simple and does not require complex surgery. c. It can be removed and cleaned, which is hygienic and convenient.
   - Cons: a. It may take some getting used to, and there may be discomfort at first. b. Periodic adjustments are required. c. Long-term use may result in excessive wear and tear of the remaining teeth or atrophy of the alveolar bone.
   - Suggestion: Suitable for patients with a large number of tooth loss and economic considerations.
2. Porcelain tooth (fixed bridge)
   - Indications: Suitable for one or more missing teeth and healthy adjacent teeth.
   - Pros: a. The appearance is natural and similar to that of natural teeth. b. Fixed in the mouth, comfortable to use, good chewing function. c. It does not need to be removed, and it is easy to clean.
   - Cons: a. Adjacent healthy teeth need to be worn for support. b. If there is a problem with the abutment or bridge, it may need to be remade. c. Medium cost, higher than dentures.
   - Suggestion: Suitable for patients with healthy adjacent teeth and certain aesthetic requirements.
3. Dental implants
   - Indications: For single or multiple tooth loss, oral conditions that allow surgery.
   - Pros: a. High aesthetics, closest to natural teeth. b. No need to wear adjacent teeth, protecting other healthy teeth. c. Strong chewing power and good recovery effect. d. It can effectively prevent alveolar bone atrophy.
   - Disadvantages: a. Surgery is required and the process is relatively complicated.
     b. The recovery period is long, taking a few months to half a year. c. The cost is higher, which is the most expensive of the three ways.
   - Selection suggestion: suitable for patients who have high requirements for aesthetic and functional recovery, and whose economic conditions allow.

When choosing the right dental restoration, you should consider the following key points:

- Oral health: including the health of the alveolar bone, the quantity and quality of remaining teeth, etc.
- Economic factors: Choose the most appropriate treatment option based on your financial situation.
- Aesthetic needs: Choose the most natural way to restore the effect according to your requirements for appearance.
- Comfort of use: Choose the most suitable method for you according to your requirements for comfort when using it.
- Maintenance costs: The expenses that may be incurred for long-term maintenance are also one of the factors to consider when choosing.

Before making a decision, it is recommended that you consult a professional dentist who will provide personalized advice based on your specific situation. In the meantime, you may also want to consider consulting with several different doctors for more information and advice to make the best choice for you.

1. Do I need to get a dental implant immediately after a tooth extraction?

Before answering your question, it's important to be clear that dental implants are a patient-specific procedure, and not everyone has to or can get a new tooth implanted immediately after a tooth extraction. Here are the key points of the answer based on my knowledge base search and recombination:

1. **Timing of dental implants**:
   - Immediate implantation: In some cases, such as if the alveolar bone is of good quality and there is no infection, the implant procedure may be performed immediately after the tooth extraction.
   - Postponement of implantation: In other cases, it may be necessary to wait for a while, such as if the alveolar bone needs to heal or the alveolar bone needs to be added in volume.
2. **Changes in alveolar bone after tooth extraction**:
   - Alveolar bone may shrink after tooth extraction, especially in the first 3 months after tooth extraction, when bone loss is most significant.
   - If alveolar bone atrophy is severe, bone grafting or bone augmentation surgery may be required, which will delay the optimal time for dental implants.
3. **Consideration of the patient's health status**:
   - Patients with chronic diseases such as diabetes and heart disease need to consider dental implants after their condition has stabilized.
   - Dental implants in smokers and alcoholics have a relatively low success rate and need to be considered for implants after quitting smoking and drinking.
4. **Treatment Plan for Dental Implants**:
   - A comprehensive oral examination and assessment is required before dental implants, including alveolar bone quality, oral hygiene status, and the patient's overall health.
   - Based on the results of the assessment, the dentist will develop a personalized implant plan, which may include immediate implants, short-term implants, or long-term planned implants.
5. **Choice of technique for dental implants**:
   - Traditional dental implants are usually performed in stages, including extractions, healing, implant placement, osseointegration and restoration.
   - The immediate loading technique allows the installation of the crown to be carried out immediately or shortly after implant placement, but this is subject to specific clinical conditions.
6. **Cost and Financial Considerations of Dental Implants**:
   - The cost of dental implants is relatively high, and patients need to consider their own financial situation.
   - Some health insurance plans may cover some of the costs, but in most cases, patients will be responsible for most or all of the costs.
7. **Patient's quality of life considerations**:
   - Missing teeth may affect the patient's chewing function, pronunciation, and appearance, and dental implants can restore these functions to a great extent.
   - Patients need to weigh the benefits of dental implants against the cost of waiting for implants.
8. **Postoperative recovery and maintenance**:
   - Dental implant surgery requires a period of recovery, during which there may be symptoms such as pain and swelling.
   - Regular check-ups and good oral hygiene habits are required after dental implants to ensure the long-term stability and longevity of the implants.

Taken together, whether or not you need an immediate dental implant depends on a number of factors, including the condition of the alveolar bone, your overall health, financial considerations, and quality of life needs. It is recommended that you consult a professional dentist for a detailed oral examination and health assessment to determine the best treatment option for you.

1. How often should I get teeth after tooth extraction?

Dental implants after tooth extraction are common dental restorations, but the duration of dental implants depends on several factors, including how well the tooth heals after the tooth extraction, the patient's overall health, the condition of the alveolar bone, etc. Here's a comprehensive tip for when to get a tooth implant after a tooth extraction based on the information in the knowledge base I've searched:

1. **Implantation immediately after tooth extraction**:
   - In some cases, if the alveolar bone is of good quality and there is no infection, dental implant surgery can be performed at the same time as the tooth extraction.
   - The benefit of this method is that it reduces the time of the overall treatment process and helps to preserve the volume and shape of the alveolar bone.
   - However, not all patients are suitable for immediate implants and need to be evaluated and advised by a professional dentist on a case-by-case basis.
2. **Early planting (weeks to months):**
   - If there is a slight infection in the extraction area or it takes time to heal, it may take a few weeks to a few months before the implant can be performed.
   - Usually, the healing period is around 6 to 8 weeks, which allows the soft tissues to heal first.
   - During this time, the dentist may follow up on the patient's healing to make sure no infection has occurred and assess the condition of the alveolar bone.
3. **Delayed planting (3-6 months or more):**
   - If the patient's alveolar bone is of poor quality or has a large infection, it may take longer to heal and prepare, and may even require a bone graft.
   - Deferred implantation usually involves waiting 3 to 6 months, or even longer, to ensure that the alveolar bone has enough mass and volume to support the implant.
   - During this period, there are a number of measures that can be taken to promote alveolar bone regeneration, such as the use of bone regeneration materials.
4. **Assessment of alveolar bone condition**:
   - Before implantation, X-rays, CT scans, etc., are needed to assess the quality and quantity of alveolar bone, and whether bone grafting or bone augmentation surgery is required.
   - The dentist will decide on the timing of the implant based on the results of the evaluation.
5. **Patient's overall health status**:
   - The overall health of the patient, such as diabetes, heart disease, etc., will also affect the timing and success rate of dental implants.
   - Some diseases or habits, such as smoking, may prolong healing or increase the risk of implant failure.
6. **Post-operative care**:
   - During the period between the extraction and the implant, patients need to strictly follow the dentist's care instructions to ensure an optimal healing environment.
   - This includes the maintenance of oral hygiene, regular check-ups, and avoiding excessive pressure on the extraction area.
7. **Patient expectations and quality of life**:
   - The timing of dental implants also needs to take into account the patient's expectations and quality of life.
   - For some patients who wish to regain chewing function and aesthetics as soon as possible, immediate or early implantation may be preferred.

To sum up, the time for dental implants after tooth extraction is not set in stone, and a variety of factors need to be considered. The most important thing is that the patient should have sufficient communication with the dentist, and the professional doctor will develop a personalized treatment plan according to the patient's specific situation. The doctor's professional judgment and advice are crucial throughout the decision-making process.

1. Are dental implants lifelong and how long can they last?

Regarding how long dental implants can be used, this is a question that involves several factors. The lifespan of dental implants can be affected by factors such as the implant technique, the patient's own condition, oral hygiene habits, and regular dental check-ups. Here are the answers based on my knowledge base search and regrouping:

1. **Dental Implant Technique**:
   - Implant materials: Modern dental implants often use titanium or titanium alloys because these materials have good biocompatibility and osseointegration capacity.
   - Implant process: Implant surgery is divided into two stages: implant placement and denture fitting, each of which requires a certain amount of healing time.
   - Technological development: With the advancement of technology, the success rate of dental implants is already very high, but the constant updating of technology also means that different dental implant systems may have different lifespans.
2. **Patient's own condition**:
   - Bone condition: Adequate bone mass and good bone quality are the keys to successful implantation. Insufficient bone mass may require bone grafting.
   - General health: Patients with systemic diseases such as diabetes, heart disease, etc., may affect the success rate and longevity of dental implants.
   - Oral health: A patient's oral health condition, such as periodontal disease, can also affect the stability and longevity of dental implants.
3. **Oral Hygiene Habits**:
   - Daily cleaning: Good oral hygiene habits, such as proper brushing and flossing, are important factors in maintaining the longevity of dental implants.
   - Regular check-ups: Regular check-ups and professional cleanings at the dentist can detect and deal with problems that may affect dental implants in a timely manner.
4. **Regular oral check-ups**:
   - Monitor the health condition around the dental implant, such as checking for signs of periodontitis.
   - Assess the wear and tear of your dentures and adjust or replace them if necessary.
5. **Lifetime statistics**:
   - The average lifespan of dental implants is usually more than 15 years, and some studies have shown that well-maintained dental implants can last for 20 years or more.
   - However, these figures are based on statistical averages, and individual differences may result in actual service life differing from the average.
6. **Other factors that may affect longevity**:
   - Smoking: Smoking is considered a negative factor that affects the longevity of dental implants as it affects blood circulation and healing processes inside the mouth.
   - Eating habits: Hard or sticky foods can put undue pressure on dentures and affect their stability.
   - Tooth wear habits: such as grinding or clenching, these habits can cause additional stress on dental implants.

In summary, although dental implants may last a lifetime, their actual lifespan is affected by a variety of factors. Patients need to work closely with their dentist to maintain dental implants through good oral hygiene habits, regular dental check-ups, and a proper lifestyle to achieve the best results and longest lifespan.

1. How should dental implant food impaction be removed?

Food impaction is a common problem after dental implants and requires proper cleaning methods to maintain oral hygiene and prevent potential oral problems. Here are some suggestions on how to clean up dental implant food impaction:

1. **Regular cleaning**:
   - Daily brushing: Use a soft-bristled toothbrush at least twice a day, especially after eating.
   - Floss: Use dental floss or floss sticks to clean the gap between the dental implant and your natural tooth at least once a day.
2. **Special Tools**:
   - Interdental brushes: Use an appropriately sized interdental brush to clear the larger gaps around the dental implant.
   - Water flosser: Using a water flosser, it uses a high-pressure stream of water to remove food debris and plaque.
3. **Correct Technique**:
   - Brushing method: Brush gently in a circular motion, avoiding horizontal and strong brushing to avoid damaging the soft tissues around the implant.
   - Flossing: Gently slide the dental floss into between the teeth and avoid using too much force to avoid injuring the implant.
4. **Regular check-ups**:
   - Regular dental check-ups: Professional dental check-ups and cleanings every 6 months to 1 year.
   - Dental Implant Maintenance: Professional maintenance of dental implants as recommended by your dentist.
5. **Dietary advice**:
   - Avoid sticky foods: Reduce or avoid foods that tend to stick to your teeth.
   - Eat a balanced diet: Maintaining a balanced diet can help maintain oral health.
6. **Habits**:
   - Quit smoking: Smoking increases the risk of oral diseases and is not good for the health of dental implants.
   - Limit alcohol: Excessive alcohol consumption may affect the healing process of the mouth and the stability of dental implants.
7. **Responding to emergencies**:
   - If you notice gum inflammation or bleeding due to food impaction, you should consult your dentist immediately.
   - If you still feel uncomfortable after cleaning, see a doctor in time to check for loose or infected dental implants.
8. **Use the product**:
   - Consider using fluoride toothpaste and mouthwash, which can help reduce bacterial growth and protect oral health.
9. **Pay attention to oral hygiene**:
   - After each meal, rinse your mouth with water to remove food debris from your mouth.
   - When performing oral cleaning, observe for redness, swelling, bleeding or abnormal sensations and report it to your dentist promptly.
10. **Pro Advice**:
    - It is best to consult a dentist before performing any oral hygiene procedure, especially when choosing a model of interdental brush and water flosser.

To summarize the above key points, cleaning up dental implant food impaction requires a comprehensive consideration of daily oral hygiene habits, the use of appropriate cleaning tools, the adoption of correct cleaning techniques, and regular oral examinations and professional maintenance. Eating habits in life and some harmful habits such as smoking and drinking alcohol also have a direct impact on the health of dental implants. If you encounter any problems, you should consult a professional dentist in time and follow their guidance and advice. Through these measures, the problem of food impaction can be effectively reduced and the health and function of dental implants can be maintained.

1. What should I do if my dental implant is inflamed?

When dental implants become inflamed, immediate action should be taken as this can be a sign of infection that may affect the success rate of the implant and oral health. Here are the answers based on the information I found in my knowledge base and regrouped:

1. **Seek medical attention** promptly:
   - Inflammation around dental implants can be a sign of inflammation around implants, which is an infection caused by bacteria.
   - If left untreated, it can lead to implant failure.
   - Patients should contact their own dentist or dental implant specialist as soon as possible.
2. **Self-management**:
   - a. **Oral hygiene**: Maintaining good oral hygiene is key to preventing and controlling infections.
     - i. Use a soft-bristled toothbrush to brush your teeth gently.
     - ii. Using an alcohol-free mouthwash can help reduce bacteria in your mouth.
   - b. **Cold compresses**: If swelling is present, a cold compress can be applied to the outside of the affected area of the face to reduce the swelling.
   - c. **Avoid irritating foods**: Avoid excessively hot, cold, or irritating foods to reduce irritation to the affected area.
3. **Medications**:
   - a. **Antibiotics**: Your doctor may prescribe antibiotics to control the infection.
   - b. **Anti-inflammatory drugs**: Nonsteroidal anti-inflammatory drugs (NSAIDs) such as ibuprofen may be recommended to reduce pain and inflammation.
4. **Monitor symptoms**:
   - a. **Watch for changes in symptoms**: Record changes in pain, swelling, or redness.
   - b. **Regular check-ups**: Even if your symptoms improve, you should follow your doctor's recommendations for regular check-ups.
5. **Surgical treatment**:
   - If the inflammation is severe and medical and conservative treatments are ineffective, surgery may be needed.
   - Surgery may include cleaning up the infected tissue around the implant and may even require the implant to be removed.
6. **Lifestyle Modifications**:
   - a. **Quit smoking**: Smoking slows down the healing process and increases the risk of infection.
   - b. **Nutrition**: A balanced diet helps the body fight infections and promotes healing.
   - c. **Avoid using toothpicks or other sharp objects**: this may damage the tissues around the dental implant.
7. **Follow your doctor's advice**:
   - a. **Take your medications**: Take all medications prescribed by your doctor on time.
   - b. **Follow-up visits**: Observe the timing of all follow-up appointments so that the doctor can monitor the healing process.
8. **Symptom Warning**:
   - If you notice pus draining, bleeding that doesn't stop, or severe pain, you should seek medical attention right away, as these can be signs of a more serious problem.
9. **Precautionary measures**:
   - a. **Regular** cleanings: Regular cleanings at the dental office can reduce the accumulation of plaque and tartar.
   - b. **Regular check-ups**: Dental implant check-ups should be done regularly, even if there are no symptoms.
10. **Psychological support**:
    - Problems with dental implants can cause anxiety and stress, and psychological support can be sought if necessary.

In conclusion, dental implant inflammation is a problem that needs to be taken seriously and should be addressed with comprehensive measures as soon as possible. Maintaining a positive attitude, following your doctor's instructions, and taking proper precautions are important factors in ensuring the success of dental implants and oral health. In any case, timely professional medical advice and treatment is crucial.

1. **Orthodontics-related problems**
2. Crooked teeth, what conditions are suitable for orthodontics?

Misaligned teeth usually refer to an irregular alignment of teeth, also known as malocclusion. Orthodontic treatment involves the use of braces or other instruments to slowly move teeth to achieve the desired alignment and occlusal relationship. The following are the conditions when orthodontic treatment is appropriate:

1. **Misaligned teeth**
   - (a) Crowding: Overlapping or twisting of teeth due to lack of space.
   - (b) Buckteeth (protruding): Excessive protrusion of the upper anterior teeth, often associated with underdeveloped mandibles.
   - (c) Crossbite (mandibular protrusion): The lower anterior teeth protrude more than the upper anterior teeth, which may be related to under-developed upper jaw or over-developed lower jaw.
2. **Bite problems**
   - (a) Open jaw: The upper and lower teeth cannot be touched during the bite and are commonly found in the front teeth.
   - (b) Deep overlay: The upper teeth cover the lower teeth too much, and sometimes the lower teeth are barely visible.
   - (c) Staggered occlusion: When teeth occlusion, one upper tooth is within the lower tooth and the other side is the opposite, resulting in an unstable bite.
3. **Functional issues**
   - (a) Difficulty chewing: Crooked teeth may lead to inefficient chewing.
   - (b) Pronunciation problems: Tooth alignment affects articulation, orthodontics can help improve speech intelligibility.
   - (c) Tooth wear: Incorrect occlusal can lead to abnormal tooth wear.
4. **Aesthetic needs**
   - (a) Boost self-confidence: Straight teeth can help improve one's self-confidence.
   - (b) Improved smile: Crooked teeth can affect the aesthetics of a smile, and orthodontics can significantly improve the appearance of a smile.
5. **Age factor**
   - (a) Adolescents: Growing up is the best time for orthodontic treatment.
   - (b) Adults: Orthodontics is still effective, although teeth may move more slowly in adulthood.
6. **Periodontal health**
   - (a) Good periodontal condition: Ensure that there is no active periodontal disease before orthodontics.
   - (b) After periodontal treatment: After periodontal disease treatment has stabilized, orthodontics can be performed.
7. **A thorough oral examination**
   - (a) X-ray: to assess the condition of the teeth and jaws.
   - (b) Oral model: understanding tooth alignment and occlusal relationships.
   - (c) Detailed consultation: Discuss the treatment plan and expected results with the orthodontist.
8. **Aligner selection**
   - (a) Metal brackets: traditional and universal aligners.
   - (b) Ceramic brackets: orthoses with high aesthetics.
   - (c) Clear aligners: clear plastic aligners that are aesthetically pleasing and undetectable.
9. **Care during treatment**
   - (a) Cleaning: Special attention needs to be paid to oral hygiene during orthodontics.
   - (b) Regular follow-up: Follow-up visits to the hospital on time to adjust the aligners.
10. **Maintenance after treatment**
    - (a) Use of retainers: Retainers are required to secure the position of the teeth after orthodontic treatment.
    - (b) Regular check-ups: Regular oral health check-ups at the hospital.

Orthodontic treatment is a complex process that requires a treatment plan that needs to be tailored to the individual's specific situation. Before considering orthodontic treatment, you should consult with a professional orthodontist for a comprehensive oral health assessment to ensure that you choose the most suitable treatment for you. Orthodontics is not only about aesthetics, but also about the function and overall health of the oral cavity.

1. Does orthodontics require tooth extraction? Which teeth are extracted? Will tooth extraction affect normal chewing?

In answering your question about whether orthodontics requires tooth extraction, I will provide a detailed explanation based on medical knowledge and the usual procedures of orthodontic treatment.

1. **The relationship between the purpose of orthodontic treatment and tooth extraction**:
   - a. The main objective of orthodontic treatment is to improve tooth alignment and occlusal relationship, as well as to improve aesthetics and functionality.
   - b. Tooth extraction is a means of orthodontic treatment, not all orthodontic treatments require tooth extraction.
2. **The necessity of tooth extraction**:
   - a. The need for tooth extraction depends on the individual's oral condition, such as the degree of crowding, the severity of the misalignment, facial aesthetics, and occlusion.
   - b. Tooth extraction is usually done to get enough space to correct the alignment of the teeth or to improve the occlusal relationship.
3. **Select the tooth to be extracted**:
   - Common tooth extraction options include first or second premolars, and occasionally wisdom teeth or other teeth may also be included.
   - b. Which tooth to extract is decided by the orthodontist based on the treatment plan and the patient's specific situation.
4. **Chewing function after tooth extraction**:
   - a. In the short term, there may be discomfort after tooth extraction, and chewing function may be temporarily affected.
   - b. In the long term, chewing function usually improves after orthodontic treatment is completed, as the teeth are aligned more neatly and the bite relationship is more harmonious.
5. **Side effects and management of tooth extractions**:
   - a. There may be some short-term side effects of tooth extraction, such as pain, swelling, or bleeding, but these usually resolve themselves within a few days.
   - b. The orthodontist will provide appropriate instructions and medications to help the patient manage the discomfort after the tooth extraction.
6. **Holistic Considerations for Tooth Extraction and Orthodontic Treatment**:
   - a. Tooth extraction is based on the overall treatment plan and the expected treatment outcome.
   - b. The orthodontist will take into account the patient's age, dental development, oral health status and other factors.
7. **Oral care after treatment**:
   - a. Good oral hygiene is very important during and after orthodontic treatment, regardless of whether the tooth is extracted or not.
   - b. Proper brushing techniques, flossing and regular dental check-ups can help maintain the results of treatment.
8. **Maintenance of the therapeutic effect**:
   - a. After the treatment is completed, it may be necessary to wear retainers to maintain the position of the teeth.
   - b. Regular follow-up visits can ensure long-term stability of treatment results.

To summarize the above key points, whether or not a tooth extraction is required for orthodontic treatment is based on the specific situation of the individual and is decided by a professional orthodontist based on the patient's oral condition, tooth alignment, and bite problems. After tooth extraction, chewing may be affected in the short term, but as the mouth adapts and recovers, chewing function will gradually improve. In the long term, orthodontic treatment can improve chewing efficiency and oral health. Good oral hygiene and regular follow-up visits are required after treatment to maintain the results of treatment.

1. Can my teeth line up after straightening?

Orthodontics is a complex process that involves several factors, including the current condition of the teeth, the choice of orthodontic method, the duration of treatment, and the degree of cooperation of the patient. Here are the answers organized in detail based on these factors:

1. **Initial condition of the teeth**:
   - a. **Crowded teeth**: If the teeth are crowded and uneven due to lack of space, orthodontics can be adjusted by adjusting the alignment of the teeth to create enough space for the teeth to line up neatly.
   - b. **Gaps between teeth**: If gaps exist, orthodontics can close those gaps by moving the teeth slowly.
   - c. **Occlusal problems**: For occlusal problems such as underfoot or buckteeth, orthodontics can adjust the alignment of the upper and lower teeth to improve the occlusion.
2. **Selection of correction method**:
   - a. **Traditional metal brackets**: This is the most common orthodontic method in which the position of the teeth is gradually adjusted by attaching metal brackets to the teeth and connecting them with wires.
   - b. **Invisalign**, for example, are clear plastic trays that can be replaced at regular intervals to move teeth step by step.
   - c. **Ceramic** brackets: Similar to metal brackets, but similar in color to teeth and more discreet.
   - d. **Lingual aligners**: Fitted on the inside of the tooth, it is almost indistinguishable from the appearance that the correction is being done.
3. **Duration of treatment**:
   - a. **Short-term orthodontics**: Some minor misaligned teeth can be resolved with short-term orthodontics, which may only take a few months.
   - b. **Long-term orthodontics**: For complex bite problems or severe tooth crowding, orthodontics can take several years to complete.
4. **Patient's level of cooperation**:
   - a. **Follow the doctor's instructions**: go to the hospital on time for a follow-up and adjust the aligners regularly.
   - b. **Daily care**: Clean your teeth and aligners properly and avoid foods that may damage your aligners.
   - c. **Wear retainers**: After orthodontic treatment, wear retainers as directed to maintain the position of your teeth.
5. **Possible complications**:
   - a. **Gum problems**: Poor oral hygiene during correction can lead to gum inflammation.
   - b. **Tooth movement limitations**: In some cases, the movement of teeth may be physiologically limited, affecting the effectiveness of orthodontic treatment.
   - c. **Root resorption**: During the orthodontic process, there may be some degree of resorption at the root of the tooth, which is a normal physiological process, but excessive root resorption needs attention.
6. **Maintenance of the therapeutic effect**:
   - a. **Regular follow-up visits**: Regular follow-up visits to the dentist for check-ups are necessary even after the orthodontic treatment is completed.
   - b. **Wear retainers continuously**: Depending on your dentist's recommendations, retainers may need to be worn for long periods of time or intermittently.
7. **Psychological and social factors**:
   - a. **Increased self-confidence**: When teeth are aligned, it often boosts an individual's self-confidence and desire to socialize.
   - b. **Improved quality of life**: Straight teeth help improve speech and chewing function, improving quality of life.

In summary, the results of orthodontic treatment depend on a variety of factors, but in most cases, the teeth can be aligned if the appropriate orthodontic method is chosen and the patient is able to follow the doctor's instructions and complete the entire orthodontic process under the supervision of the doctor. However, each person's situation is unique, and the exact outcome needs to be communicated with a professional dentist and a treatment plan tailored to the individual's specific situation.

1. How much does orthodontics improve the mouth protrusion?

Orthodontic treatment can have a significant effect on improving the protrusion of the mouth. Here's a detailed answer to this question based on what I know:

1. **Overview of orthodontic treatment**
   - Orthodontic treatment is a treatment that improves tooth alignment and occlusion by using braces or other oral devices to gradually move teeth and affect jaw growth.
   - This type of treatment is usually performed by a specialist orthodontist and may include means such as fixed aligners (such as traditional metal brackets), invisible aligners, functional appliances or maxillofacial surgery.
2. **Causes of the mouth protrusion**
   - The protrusion of the mouth can be caused by a variety of factors, including genetic factors, poor oral habits (such as long-term finger sucking), abnormal tooth development, or uncoordinated jaw growth.
   - This condition can lead to cosmetic problems with the face and dysfunction of chewing, pronunciation, etc.
3. **Orthodontic treatment for the improvement of the mouth process**
   - **Tooth alignment adjustment:** Orthodontics can promote the normal growth of the jaw to a certain extent by correcting the alignment of teeth and reducing the abnormal pressure on the jaw.
   - **Jaw growth guidance:** For children and adolescents, the use of functional appliances during the growth and development period can effectively guide the growth direction of the jaw and improve the mouth process.
   - **Maxillofacial Surgery:** For adult patients, orthodontics combined with maxillofacial surgery may be required if the oral protrusion problem is primarily due to jaw dysplasia.
   - **Comprehensive treatment options:** In some complex cases, a combination of orthodontic treatments may be required to achieve the best results.
4. **Therapeutic Efficacy Factors**
   - **Patient age:** Younger patients have a growing jaw bone and are more likely to improve with orthodontic treatment.
   - **Case complexity:** The severity of the oral process and the growth of the jaw bone directly affect the effectiveness of treatment.
   - **Patient cooperation: The**  patient's cooperation throughout the treatment process, such as wearing aligners on time and regular follow-up visits, are important factors that affect the effectiveness of the treatment.
5. **Duration of treatment**
   - Orthodontic treatment usually takes a certain amount of time, with mild oral protrusions taking one to two years, while severe oral protrusions or cases requiring maxillofacial surgery may take longer to treat.
6. **Treatment risks and precautions**
   - Orthodontic treatment is a relatively safe procedure, but there are still risks such as irregular tooth movement speed, root resorption, and loose teeth.
   - Patients should maintain good oral hygiene and avoid eating hard or sticky foods during treatment to reduce damage to aligners and oral health problems.
7. **Follow-up maintenance**
   - After orthodontic treatment, patients usually need to wear retainers to fix the effect of the treatment and prevent tooth retraction.

In summary, orthodontic treatment can improve the problem of mouth protrusion to a large extent, but the specific treatment effect needs to be based on the specific situation of the individual. Patients are advised to consult a professional orthodontist in detail to understand their condition and treatment options before starting treatment.

1. Can orthodontics adjust the occlusion?

Orthodontic treatment is the process of adjusting the position of teeth and jaws through the use of orthodontic appliances or other dental devices to improve occlusion problems and improve oral function and aesthetics. Here are the key points for orthodontic treatment to adjust the occlusion, along with a detailed explanation of these key points:

1. **Diagnose the type of bite problem**:
   - Before orthodontic treatment, the dentist will perform a comprehensive examination, including an oral examination, a model of the tooth, X-rays, and possibly 3D scans, to determine the type of bite problem (e.g., open bite, deep bite, staggered bite, etc.).
   - Small points: Dentists use this information to develop a treatment plan.
2. **Choosing the Right Orthodontic Device**:
   - Depending on the severity and type of bite problem, the doctor may recommend different aligners, such as traditional metal aligners, ceramic aligners, invisible aligners, or maxillofacial surgery.
   - Small points: Each type of aligner has its characteristics, for example, invisible aligners are more popular in terms of aesthetics and comfort.
3. **Move the teeth step by step**:
   - Orthodontic devices gradually move the teeth into the ideal position by applying pressure.
   - Small points: This process can take anywhere from a few months to a few years, during which time you will need to visit your dentist regularly to make adjustments.
4. **Adjust the jaw relationship**:
   - In some cases, occlusal problems are caused by an imbalance in jaw development and may need to be adjusted with orthodontics combined with maxillofacial surgery.
   - Small dots: This treatment is usually more common in adults because their jaws have stopped growing.
5. **Improves chewing function**:
   - Proper occlusal can improve chewing efficiency and reduce abnormal wear and tear on teeth.
   - Small dots: Long-term malocclusion can lead to excessive wear and tear on teeth, jaw joint problems, or muscle pain.
6. **Improves oral hygiene**:
   - Straight teeth are easier to clean and reduce the risk of periodontal disease and tooth decay.
   - Small spots: Misocclusion can lead to areas of teeth that are difficult to clean, increasing the accumulation of plaque and tartar.
7. **Aesthetic effect**:
   - In addition to improving the occlusion, orthodontic treatment can also significantly improve the aesthetics and self-confidence of the patient's smile.
   - Small dots: Aligned teeth can have a positive impact on social and professional life.
8. **Maintenance Therapeutic Effect**:
   - After completing orthodontic treatment, retainers are usually needed to maintain the position of the teeth.
   - Small dots: If you don't wear a retainer, your teeth may return to their previous position.
9. **Regular review and maintenance**:
   - Even after orthodontic treatment is completed, regular follow-up examinations are still required to ensure that the occlusal relationship remains normal.
   - Small points: Regular reviews can also help to identify and deal with minor problems that may arise, such as the adjustment or replacement of retainers.
10. **The Importance of Patient Collaboration**:
    - It is important for the patient to cooperate throughout the treatment process, such as regular follow-up appointments, proper wearing of aligners and retainers, and good oral hygiene.
    - Small spots: The patient's lack of cooperation may result in poor treatment response or prolonged treatment.

Orthodontic treatment is a complex process that involves expertise and techniques in multiple fields. Every patient's situation is unique, so the treatment plan needs to be personalized as well. Nonetheless, the above key points provide a basic framework and necessary steps regarding the adjustment of the occlusion in orthodontic treatment. It is important to work closely with an experienced dentist to ensure the best possible treatment results.

1. Will orthodontics change my face shape?

Orthodontic treatment is usually done to improve the alignment and occlusion of teeth, but it can also have an impact on facial appearance. Here's what I've put together based on my knowledge base:

1. **Improvement in tooth alignment**:
   - Orthodontic treatment is able to align the teeth, which directly affects the contour of the face.
   - When teeth are misaligned or there is a bite problem, the face shape may appear uncoordinated.
   - The supporting effect of teeth on the face shape may be enhanced after orthodontics, making the face shape look more harmonious.
2. **Adjustment of the occlusal relationship**:
   - Orthodontics can correct occlusal problems such as protrusion, opening and closing, deep coverage, etc., and these improvements in occlusion may have an impact on the face shape.
   - For example, correction of protrusion (commonly known as "ground wrapping") may make the side contour of the face straighter.
3. **Considerations for jaw growth**:
   - For adolescent patients, orthodontic treatment can guide the growth of the jaw to a certain extent, which in turn affects the face shape.
   - Jaw growth in adults is almost complete, and orthodontics has a relatively limited impact on the face shape.
4. **Soft tissue adaptability**:
   - Soft tissues (such as lips, cheeks, etc.) adapt to changes in teeth and jaws, and may change with them.
   - Moving the teeth forward may make the lips fuller, while moving the teeth back may make the lips appear flatter.
5. **Long-term effects**:
   - After orthodontic treatment, the changes in face shape are usually gradual and may take a while to become fully apparent.
   - The post-treatment retention period is also very important to ensure that the changes in teeth and face shape are stable.
6. **Individual differences**:
   - Everyone's reaction is different, and the degree to which orthodontics affects the shape of the face varies from person to person.
   - Some people may find the change noticeable, while others may feel that the change is not significant.
7. **Professional Assessment**:
   - A thorough evaluation of the face and teeth should be performed by a professional orthodontist before orthodontic treatment is considered.
   - An orthodontist can provide a professional opinion on what to expect from your treatment, including the possible effects on your face shape.
8. **The importance of a treatment plan**:
   - A well-designed treatment plan will take into account the potential impact of the treatment on the face shape.
   - The treatment plan should be tailored to the individual's specific situation to achieve optimal functional and aesthetic results.
9. **Psychological and social factors**:
   - Orthodontic treatment may improve an individual's self-confidence and social interactions, as improvements in tooth alignment tend to make people look more attractive.
   - Psychological research has shown that improvements in appearance can have a positive impact on an individual's mental health and social life.

In summary, orthodontic treatment may have an impact on the shape of the face, depending on a variety of factors, including the specific circumstances of the treatment, the physiological characteristics of the individual, and the adaptability of the soft tissues. If you are considering orthodontic treatment, it is advisable to consult a professional orthodontist for a more detailed and personalized assessment and advice.

1. Are there any precautions to be taken during correction?

There are quite a few things to look out for during the correction period, involving many aspects of daily life. Here are some key takeaways from what I know:

1. **Oral hygiene maintenance**
   - Clean your teeth: Brush your teeth at least twice a day, use a soft-bristled toothbrush and fluoride toothpaste, and clean your teeth and aligners thoroughly.
   - Floss: Floss specifically designed for aligners and clean food debris between your teeth and under your aligners at least once a day.
   - Mouthwash: Use an antibacterial mouthwash to help reduce bacteria and prevent gingivitis and mouth sores.
2. **Dietary changes**
   - Avoid hard foods: Avoid foods such as hard nuts and hard candies that may cause damage to the aligners.
   - Bypass sticky foods: Avoid sticky foods such as milk candy to avoid sticking to the aligners and making them difficult to clean.
   - Reduce sugar intake: Reduce sugar intake to avoid increasing the risk of tooth decay.
   - Divide the food: Cut the food into small pieces to reduce the pressure on the aligners.
3. **Protection of aligners**
   - Avoid bad habits: Don't bite hard objects with your teeth, such as pens, nails, etc., to prevent damage to aligners.
   - Sports protection: When participating in sports, use oral protectors to protect your teeth and aligners.
4. **Review regularly**
   - Follow-up appointments: Regular follow-up appointments allow your doctor to check the condition of your aligners and the movement of your teeth.
   - Timely feedback: If the aligners are loose or damaged, you should contact your doctor immediately for treatment.
5. **Pain management**
   - Coping with pain: There may be discomfort at the beginning of the correction process, which can be relieved by applying cold compresses or taking over-the-counter pain relievers recommended by your doctor.
   - Avoid irritating foods: such as spicy, excessively hot or cold foods, which may exacerbate oral discomfort.
6. **Respond to emergencies**
   - Damaged aligners: If the aligner parts are damaged, you should contact your doctor as soon as possible and do not try to repair them yourself.
   - Canker sores: If the aligners are causing canker sores, topical anti-inflammatory creams can be used and spicy and irritating foods should be avoided.
7. **Mental preparation**
   - Be patient: Orthodontics is a long process that requires a lot of patience.
   - Positive Attitude: Maintain a positive attitude and believe that you will have a better smile after correction.
8. **Adjustment of lifestyle habits**
   - Speaking and swallowing: In the early stages of correction, you need to adapt to the different sensations you feel when speaking and swallowing.
   - Musical instruments: People who play musical instruments need to adapt to the changes brought about by the aligners, which may take a while to adjust.
9. **Wear a night retainer**
   - If recommended by your doctor, you will need to wear a night retainer to keep your teeth in place after the orthodontic treatment is over.
10. **Communication with your doctor**
    - Maintain communication: Communicate regularly with your doctor about the progress of the correction and any things to look out for.

The above are some of the key points to be aware of during the correction period. Everyone's situation is different and the specific precautions may vary, so it's important to follow your orthodontist's instructions. In conclusion, maintaining good oral hygiene habits, following the doctor's instructions, regular follow-up visits, and patiently waiting for the results of the correction are all areas that need special attention during the correction period.

1. How can I ensure the effectiveness of clear aligners?

The key points to ensure the effectiveness of the treatment of clear aligners are as follows:

1. **Choosing the right orthodontic plan and doctor**:
   - **Professional Qualifications**: Choose a qualified orthodontist and ensure that they have a proven track record and extensive experience.
   - **Personalized treatment plan**: Make sure that your doctor develops a personalized treatment plan based on your specific situation, including the type of aligners, the duration of the treatment, and the expected results.
2. **Observe the wearing time regulations**:
   - **Daily wear time**: Wear the appliance for at least 22 hours a day to ensure that the appliance has enough time to apply pressure to the teeth.
   - **Change the aligners on time**: Continue the treatment process by replacing the appliances regularly according to your doctor's instructions.
3. **Maintain oral hygiene**:
   - **Clean the aligners**: Clean the clear aligners daily to prevent bacterial growth and tartar buildup.
   - **Teeth cleaning**: Use dental floss and a toothbrush to clean your teeth to reduce the risk of tooth decay and periodontal disease.
   - **Regular check-ups**: Visit your dentist regularly for check-ups and cleanings to ensure your oral health.
4. **Dietary Adjustments**:
   - **Avoid hard and sticky foods**: Avoid chewing hard or sticky foods to prevent the appliance from being deformed or damaged.
   - **Remove the appliance when not drinking water**: Remove the appliance when eating to prevent staining and damage.
5. **Regular follow-up visits**:
   - **Follow up the progress of treatment**: Regular follow-up visits for doctors to check the progress of orthodontic treatment and adjust the treatment plan in time.
   - **Solve the problem**: If there is any discomfort or damage to the aligner, you should contact the doctor in time to solve the problem.
6. **Follow your doctor's instructions**:
   - **Use aids**: If your doctor recommends any aids, such as rubber bands, make sure they are used correctly.
   - **Avoid self-adjustment**: Do not try to adjust the appliance yourself, so as not to affect the treatment effect.
7. **Wait patiently for the effect**:
   - **Understand the process**: Recognize that orthodontics is a gradual process and it takes time to see results.
   - **Mental preparation**: There may be some discomfort, but it usually gradually lessens over time.
8. **Wear retainers**:
   - **Post-orthodontic maintenance**: After the orthodontic treatment is completed, put on the retainer according to the doctor's instructions to maintain the new position of the teeth.
9. **Lifestyle Adjustments**:
   - **Avoid bad habits**: Avoid smoking and excessive alcohol consumption, which may affect oral health and corrective effects.
   - **Maintain a healthy lifestyle**: Good lifestyle habits contribute to overall health and are also beneficial to the results of corrections.

Through the implementation of the above key points, the therapeutic effect of the clear aligner can be maximized. It is important to maintain good communication with the doctor and provide timely feedback on any problems during the treatment process so that the doctor can adjust the treatment plan to ensure that the treatment goes smoothly. At the same time, the patient's own cooperative attitude and adjustment of lifestyle habits are also important factors for successful correction.

1. What is the process of lingual correction?

Lingual orthodontic treatment is a type of orthodontic treatment that, unlike the common frontal bracket correction, has orthodontic instruments such as brackets and coils that are attached to the lingual side of the tooth, so they are almost invisible in appearance. The following is the general process of lingual correction:

1. Initial consultation
   - The patient goes to the orthodontist for a consultation for the first time.
   - The doctor will check the health of the person's mouth and take a medical history.
   - Discuss the patient's corrective needs and desired outcomes.
2. Check in detail
   - Perform a thorough examination of the mouth, including teeth, gums, and jaw structures.
   - X-rays, tooth models and oral photographs are taken to analyze the alignment of the teeth in detail.
3. Develop a treatment plan
   - Based on the results of the examination, the doctor will develop a personalized treatment plan.
   - Discuss the course of treatment, time, cost, and possible risks and complications.
4. Make personalized brackets
   - Use the patient's tooth model to create your own lingual bracket.
   - This step may take a while because the brackets are custom-made.
5. Install the brackets
   - After the brackets are made, the patient needs to return to the doctor, and the doctor attaches the brackets to the lingual side of the tooth.
   - Adjust the brackets to ensure comfort and no harm to the tongue.
6. Regular follow-up adjustments
   - Regular follow-up visits are required during the orthodontic process so that the doctor can adjust the pressure on the brackets and promote tooth movement.
   - The frequency of follow-up visits is usually every 4 to 6 weeks.
7. Oral hygiene guidance
   - Your doctor will instruct you on how to clean the lingual brackets and maintain oral hygiene.
   - Special toothbrushes and cleaning tools may be required.
8. Monitoring of the treatment process
   - Throughout the orthodontic process, the doctor will monitor the movement and progress of the teeth.
   - If necessary, make adjustments to ensure optimal correction.
9. End of correction and maintenance phase
   - After the scheduled correction time has been completed, the brackets are removed.
   - To maintain the position of the teeth, it may be necessary to wear retainers for a period of time.
10. Follow-up tests
    - Even after the orthodontic treatment is over, regular oral health check-ups are required.
    - Make sure that the position of the teeth is stable and that possible problems are dealt with in a timely manner.

The advantage of lingual correction is that it is hidden, but it can also be accompanied by certain discomforts, such as irritation of the tongue and difficulty in cleaning. In addition, the cost of treatment is usually higher than that of traditional orthodontics. Patients should fully understand the treatment process, possible discomfort and follow-up maintenance requirements before choosing lingual correction, and fully communicate with the doctor to ensure the success of the treatment and satisfactory results.

1. Are there any complications of orthodontic treatment?

Orthodontic treatment, commonly known as orthodontic treatment, is safe and effective in many cases, but like all medical procedures, it can come with some complications. Here are the answers that were searched based on the information found in my knowledge base and regrouped and arranged according to your requirements:

1. **Pain and discomfort caused by tooth movement**
   - In the early stages of orthodontic treatment, patients may experience pain or discomfort as their teeth begin to move.
   - Usually this pain is most noticeable within a few days of the start of treatment and then gradually decreases.
   - Pain can usually be controlled with regular over-the-counter pain relievers.
2. **Periodontal problems**
   - During orthodontic treatment, if the oral hygiene is not properly maintained, it can lead to gingivitis or periodontal disease.
   - Periodontal disease can lead to loose teeth and, in severe cases, tooth loss.
   - Regular oral hygiene and good daily oral hygiene habits are essential to prevent periodontal problems.
3. **Tooth demineralization and white spots**
   - Orthodontic instruments can hinder the cleaning of teeth, leading to the accumulation of bacteria and food debris.
   - This can lead to demineralization of the tooth surface and the formation of white spots, known as orthodontic leukoplakia.
   - Preventive measures include using fluoride wash water and avoiding foods high in sugar.
4. **Root resorption**
   - Some patients may experience root resorption, which is a shortening of the root length during treatment.
   - Mild root resorption may not cause problems, but severe root resorption may affect the stability of the tooth.
   - Regular radiological examinations help to monitor the condition of the tooth roots.
5. **Permanent teeth do not erupt or are abnormally positioned**
   - Orthodontic treatment may affect the location of permanent teeth that have not erupted.
   - Sometimes it is necessary to work with an oral surgeon to perform tooth extractions or other surgeries to assist in orthodontic treatment.
6. **Occlusal instability and regression after treatment**
   - After completing orthodontic treatment, the tooth may gradually return to its pre-treatment position without the use of retainers.
   - It may take time to stabilize the bite and use the retainer strictly as directed.
7. **Tooth and periodontal tissue damage**
   - Orthodontic devices can cause soft tissue injuries such as gums, cheeks, and tongue.
   - Orthodontic devices that have not been adjusted for a long time can irritate or damage periodontal tissue.
8. **anaphylaxis**
   - Rarely, patients may have an allergic reaction to certain orthodontic materials (e.g., metal, rubber).
   - If you experience allergic symptoms, you should contact your orthodontist immediately.
9. **Tooth damage**
   - Orthodontic instruments exert pressure on the teeth, which can lead to tooth damage if not administered properly.
   - These include fractured, cracked, or enamel damage.
10. **Oral habits**
    - Certain oral habits, such as using the tongue to top teeth or biting lips, may affect the effectiveness of orthodontic treatment.
    - Professional guidance is needed to improve these habits to ensure treatment results.

In conclusion, although there is a certain risk of complications in orthodontic treatment, most complications can be prevented or controlled by choosing an experienced orthodontist, following professional guidance, and maintaining good oral hygiene habits. If there are any problems or discomfort during the treatment, you should communicate with your orthodontist in a timely manner.

**Querying GPT-4 with Standard Method (Open-ended Questions)**

1. **Endodontics-related problems**
2. What are the common symptoms of endodontic disease?

Endodonticosis is a common disease of the tooth that mainly involves the hard tissue of the tooth and the pulp (i.e., the soft tissue inside the tooth). Common symptoms of this condition include:

1. **Toothache**: This is the most common symptom and may present as persistent or paroxysmal pain. Pain may be triggered by cold, hot, sweet, or sour foods and beverages, or it may worsen with biting or touching.
2. **Increased sensitivity**: Teeth respond more to cold, hot, sweet, or sour stimuli.
3. **Swollen or bleeding gums**: This may be a sign of pulpitis or apical periodontitis.
4. **Color change**: The tooth is discolored and may turn gray or black, which is usually a sign of pulp death.
5. **Discomfort when chewing**: Feeling discomfort or pain in your teeth when eating.
6. **Loose teeth**: Severe endodontic disease can lead to loose teeth.
7. **Nocturnal pain**: Toothache that worsens at night or when lying down.
8. **Swelling of the face or jaw**: This may be a sign that the infection has spread to the tissues around the teeth.

If one or more of these symptoms are present, it is advisable to seek medical attention promptly so that proper diagnosis and treatment can be made. If left untreated, endodontic disease can lead to further complications such as pulp necrosis, apical periodontitis, and even tooth loss.

1. How can I relieve toothache caused by endodontics?

There are several ways to relieve toothache caused by endodontic disease:

1. **Oral hygiene maintenance**: Maintaining good oral hygiene, brushing and flossing regularly, can reduce the occurrence of pain.
2. **Cold compress**: Gently apply a cold pack to the area of the face corresponding to the painful tooth to reduce pain and swelling.
3. **Avoid irritating foods**: Avoid foods that are too hot, too cold, or too hard, as well as foods that are high in sugar, which can worsen toothache.
4. **Medication relief**: Use over-the-counter pain relievers, such as ibuprofen or acetaminophen, to reduce pain. However, care should be taken not to rely on or overuse it for a long time.
5. **Salt water gargle**: Rinsing your mouth with warm salt water can temporarily reduce toothache, and salt water has an anti-inflammatory effect.
6. **Visit your dentist**: If the toothache persists or is very severe, you should make an appointment with your dentist as soon as possible. Endodontic disease may require specialized treatment, such as fillings, root canal treatments, etc.

Keep in mind that the above methods only provide temporary pain relief and do not treat endodontic disease itself. The fundamental solution to toothache is to treat its cause, so it is very important to seek help from a dentist. At the same time, it is best to consult a doctor or pharmacist before using any medication.

1. Do endodontic patients have to have root canal treatment?

Whether or not a person with endodontic disease needs root canal treatment depends on the specific condition. Endodontic disease refers to diseases that occur in the hard tissues of the teeth (enamel and dentin) as well as the pulp (the soft tissues inside the teeth). These diseases are usually caused by tooth decay, tooth damage or other factors.

Root canal treatment, also known as endocant treatment, is mainly used to treat pulpitis or apical periodontology, which is when the pulp is infected or inflamed. The treatment involves removing the infected pulp tissue, cleaning and disinfecting the root canal system, and finally filling the root canal to prevent future infections.

But not all endodontics have to undergo root canal treatment. Minor dental injuries or early endodontic lesions can sometimes be managed with other treatments, such as fillings, endodontic care or pulpectomy. The decision to proceed with root canal treatment usually depends on the following factors:

1. **Condition of the pulp**: If the pulp has died or there is irreversible inflammation, root canal treatment may be necessary.
2. **Degree of infection**: Deep caries or severe tooth damage can lead to an infection of the pulp, in which case root canal treatment is usually required.
3. **Symptoms**: The patient's symptoms, such as severe pain or the formation of an abscess, are also important factors in deciding whether root canal treatment is needed.

It is recommended that the dentist evaluate and advise on a case-by-case basis. The dentist will evaluate the health of the pulp through clinical examinations and X-rays and recommend appropriate treatment options.

1. What are the symptoms of pulpitis?

Pulpitis, also known as inflammation of the pulp, usually has the following symptoms:

1. **Toothache**: This is the most common symptom and can be intermittent or continuous. The pain may worsen at night, and sometimes eating hot or cold foods or sweets can trigger or worsen the pain.
2. **Increased sensitivity**: Teeth are sensitive to hot, cold, sweet or sour foods and drinks.
3. **Discomfort or pain when chewing**: You may feel pain or discomfort in your teeth when chewing food.
4. **Swelling or tenderness**: Swelling or tenderness may occur on the gums around the teeth.
5. **Pain that gets worse at night**: Some patients may find that their toothache gets worse at night.
6. **Headache or jaw pain**: Sometimes pulpitis can cause headache or jaw pain.
7. **Tooth discoloration**: Pulpitis can cause gray or black discoloration of teeth.

If one or more of these symptoms occur, prompt medical attention is recommended. If left untreated, pulpitis can lead to more serious problems such as tooth loss or spreading infection.

1. What is Root Canal Treatment?

Root canal treatment, also known as root canal filling treatment, is a dental treatment used to treat infection or inflammation inside the tooth (pulp). This condition is usually caused by tooth decay, a laceration of a tooth, or a severe blow to the tooth. The steps of root canal treatment are roughly as follows:

1. **Visit for diagnosis**: The dentist will examine the affected tooth and may include X-rays to determine if root canal treatment is needed.
2. **Local anesthesia**: Before treatment begins, the dentist usually uses a local anesthetic to numb the affected tooth area and reduce pain.
3. **Opening**: The dentist drills a small hole in the affected tooth so that it can access the inside of the tooth.
4. **Cleaning the infection**: Through this hole, the dentist will remove the infected or inflamed pulp tissue and clean the root canal inside the tooth.
5. **Root canal formation**: After cleaning, the dentist will shape the root canal in preparation for the filling material.
6. **Filling the root canal**: After the root canal has been cleaned and shaped, the dentist fills it with a special material to prevent the infection from recurring.
7. **Closure**: Finally, the dentist will close the hole of the opening and may cover a filling or crown to restore the shape and function of the tooth.

Root canal treatment can save an infected tooth, prevent the spread of infection, and help patients maintain their natural teeth. After treatment, the tooth may require further restorative work, such as placing a crown to improve the strength and durability of the tooth.

1. What do I need to pay attention to after root canal treatment?

After root canal treatment, there are a few things to keep in mind to ensure the effectiveness of the treatment and avoid potential complications:

1. **Avoid chewing hard foods**: Avoid chewing hard foods on your treated teeth for a few days after treatment to prevent cracking.
2. **Maintain oral hygiene**: Brush and floss regularly to clean all teeth, especially those undergoing root canal treatment.
3. **Regular follow-up visits**: Follow your dentist's recommendations for follow-up appointments to ensure that the root canal-treated tooth returns to normal.
4. **Follow your dentist's instructions**: If your dentist recommends specific medications or special oral care, follow those instructions.
5. **Watch out for pain and discomfort**: There may be mild discomfort or pain after treatment, which is usually normal. However, if the pain worsens or persists, you should contact your dentist.
6. **Avoid smoking and alcohol**: these substances may affect the healing process of the mouth.
7. **Pay attention to tooth discoloration**: After root canal treatment, teeth may become discolored. If visible discoloration occurs, consult a dentist.

It is important to follow the specific instructions of your dentist, as everyone's situation may be different. If you have any questions or concerns, please consult your dentist.

1. What should I do if my root canal treatment fails?

Root canal treatment failures, while uncommon, do happen. If root canal treatment fails, there are several ways to deal with it:

1. Redo the root canal: If the root canal failure is due to technical problems or the complexity of the root canal system, the root canal treatment may be redone. This involves reopening the tooth, removing the original filling, cleaning and disinfecting the root canal again, and then refilling and sealing.
2. Apicoectomy: If redoing root canal treatment is not appropriate or fails, apical surgery may be considered. This surgery involves removing a small part of the root tip of the tooth as well as the surrounding inflamed tissue and sealing it at the root tip.
3. Tooth extraction: In some cases, tooth extraction may need to be considered if the tooth is severely damaged or the prognosis for treatment is poor. Dental implants, fixed bridges or removable dentures can then be used to restore chewing function and aesthetics.
4. Conservative treatment or observation: In some cases, if root canal treatment fails but symptoms are mild, conservative treatment or regular observation may be an option.

Importantly, each case is unique and the most appropriate treatment option needs to be determined based on the individual's specific situation and the condition of the teeth. If you experience this condition, it is advisable to consult a professional dentist for professional advice and treatment specific to your individual situation.

1. What are the options for dental fillings?

There are several different options for dental fillings, each with its own specific benefits and uses. Here are some common dental fillings:

1. **Amalgam (silver filling):** This is a traditional filling material made of mercury, silver, tin and copper. Amalgam is durable and affordable, but it has been used less and less in recent years due to its color and mercury content.
2. **Composite resin (white filling):** This material is similar in color to natural teeth, has a high aesthetic value, and is suitable for the restoration of anterior teeth or visible areas. Composite resins bond strongly to teeth, but may not be as durable as metal materials.
3. **Porcelain**: The porcelain filling material is also very natural and beautiful, with good abrasion resistance and a high degree of color matching to the teeth. Porcelain fills are generally more durable than composite resins, but they are more costly.
4. **Gold**: Gold fillings are very durable and react less to other materials in the mouth. However, the cost of gold filling is higher, and the appearance is not as natural as composite resin or porcelain.
5. **Glass ions**: This material releases fluoride, which helps protect teeth from corrosion. It is commonly used for temporary fillings of children's teeth and non-weight-bearing teeth.

Each material has its specific indications and limitations. The choice of filling material often depends on the location of the prosthetic site, the health of the oral cavity, the patient's aesthetic needs, and budget. Based on these factors, the dentist will recommend the most suitable filling material for the patient.

1. What do I need to pay attention to after a tooth filling?

There are a few things to keep in mind after a tooth filling (filling) to ensure the durability of the filling and oral health:

1. **Avoid hard** foods: Chewing hard foods should be avoided for the first few hours after filling to prevent damage to the new filling.
2. **Pay attention to sensitivity**: Filled teeth may have transient sensitivity, especially to hot and cold foods. If sensitivity persists or intensifies, consult your dentist.
3. **Maintain oral** hygiene: Continue to practice good oral hygiene, including brushing and flossing regularly, to prevent new cavities from occurring.
4. **Avoid biting your lip or tongue**: If you have a local anesthetic, you need to be careful not to bite your tongue or lip until the anesthesia wears off.
5. **Regular check-ups**: Visit your dentist regularly to make sure the fillings are in good condition and to address any issues promptly.
6. **Follow your dentist's instructions**: Depending on your specific situation, your dentist may provide more specific guidance on your care.

If you experience persistent discomfort, pain, or other problems after filling, contact your dentist promptly.

1. What is the effect of diet on tooth decay?

The effect of diet on tooth decay (commonly known as tooth decay) is significant. Here are some of the main impacts:

1. **Sugary foods and drinks**: Intake of foods and beverages high in sugar is one of the main causes of tooth decay. When you eat sugary foods, bacteria in your mouth break down the sugar into acids, which can erode the enamel of your teeth, which can lead to tooth decay.
2. **Sticky foods**: Sticky foods such as dried fruits and gummies tend to attach to your teeth and are not easily washed off by saliva, increasing the time for acid to erode your teeth.
3. **Acidic foods and drinks**: Acidic foods and drinks (e.g., citrus fruits, sodas, sports drinks) can also damage tooth enamel.
4. **Frequent eating and snacking**: Frequent eating or snacking, especially sugary foods, can provide bacteria with a constant supply of sugar, which can increase acid production and lead to an increased risk of tooth decay.
5. **Unhealthy eating habits**: Unhealthy eating habits, such as not eating vegetables and fruits regularly, can lead to oral health problems, including tooth decay.

To prevent tooth decay, it is recommended to reduce the intake of high-sugar foods and beverages, maintain a healthy diet, and practice regular oral hygiene.

1. **Periodontal disease**
2. What is Periodontal Disease?

Periodontal disease is a disease of the tissues surrounding the teeth that mainly affects the gums and bones that support the teeth. This condition is usually caused by plaque (a thin film of bacteria that adheres to the surface of your teeth), especially when these bacteria accumulate and form tartar. The main features of periodontal disease include red, swollen gums, bleeding, receding gums, and can eventually lead to loose and lost teeth.

Periodontal disease is divided into two main stages:

1. Gingivitis: This is the early stage of periodontal disease, when the gums may become red, swollen, and bleed easily, especially when brushing or flossing. Gingivitis can often be reversed with good oral hygiene habits.
2. Periodontitis: If gingivitis is left untreated, it can develop into periodontitis. This is a more serious condition in which deep pocket-like structures form between the gums and teeth, and bacteria build up in these pockets, leading to further inflammation, gum recession, and damage to the tooth's supporting structures.

The key to preventing periodontal disease is to maintain good oral hygiene, including regular brushing, flossing, and regular visits to the dental office for professional cleaning. If periodontal disease has already developed, you should seek the help of a dentist for treatment, which may include professional cleaning, medication, and even surgery in some cases.

1. What are the causes of periodontal disease?

Periodontal disease, also known as periodontitis, is a common condition that affects the tissues around the teeth. The reason for it usually involves several factors, including:

1. **Plaque and tartar accumulation**: Plaque is a sticky substance made up of bacteria that accumulate on the surface of your teeth over time and do not remove it. These bacteria can irritate the gums, leading to inflammation.
2. **Poor oral hygiene**: Incorrect or irregular brushing and flossing can lead to the accumulation of plaque and tartar, increasing the risk of periodontal disease.
3. **Smoking**: Smokers have a higher risk of periodontal disease than non-smokers because smoking reduces blood flow in the mouth, affecting the health of gum tissue.
4. **Diabetes**: People with diabetes are more likely to develop periodontal disease because diabetes affects the body's ability to fight infections.
5. **Genetic factors**: Some people may be more susceptible to periodontal disease because of genetic factors.
6. **Hormonal changes**: Hormonal changes during pregnancy, menstrual cycle, menopause, etc., can make gums more sensitive and prone to inflammation.
7. **Medication effects**: Certain medications, such as heart medications and anti-epileptic medications, can cause abnormal growth of gum tissue.
8. **Immune system disorders**: Diseases such as HIV/AIDS and cancer and their treatment can affect oral health.
9. **Stress**: Long-term stress can affect the body's immunity, which can increase the risk of periodontal disease.
10. **Poor eating habits**: A diet deficient in vitamin C and other nutrients may affect the health of your gums.

Understanding these factors can help you take preventive measures to maintain good oral health. If you have symptoms of periodontal disease, such as bleeding gums, swollen or loose teeth, you should seek medical attention.

1. What are the symptoms of periodontal disease?

Periodontal disease is a common oral health problem that mainly affects the tissues around the teeth. Its symptoms include:

1. **Red, swollen and bleeding gums**: This is often the earliest sign of periodontal disease. When you brush or floss your teeth, your gums may bleed.
2. **Bad breath**: Periodontal disease can cause persistent bad breath or bad breath.
3. **Gum recession**: The gums gradually recede down or upward, making the teeth appear longer.
4. **Loose teeth**: As periodontal disease progresses, the tissues that support the teeth are destroyed and the teeth may become loose or displaced.
5. **Difficulty or pain in chewing**: You may feel pain or discomfort when eating because your gums and other tissues are infected and inflamed.
6. **Deep pockets form in the** gums: Deep pockets may form between the gums and teeth, where bacteria and food debris can accumulate, exacerbating infection and inflammation.

If you suspect that you have symptoms of periodontal disease, you should consult a dentist for professional diagnosis and treatment. If left untreated, periodontal disease can lead to more serious oral health problems, including tooth loss.

1. Is periodontal disease related to genetics?

There is indeed a link between periodontal disease and genetics. Genetic factors can influence an individual's susceptibility to periodontal disease. According to research, if someone in the family has periodontal disease, other family members may be at increased risk of developing the disease. This is because certain genetic factors may affect the body's immune response, inflammatory response, as well as the oral environment, which can affect periodontal health.

However, genetic factors are only one of the many factors that contribute to periodontal disease. Lifestyle habits, oral hygiene habits, smoking, diabetes, and other systemic health conditions also play an important role in the development of periodontal disease. Therefore, even if there is a genetic predisposition, good oral hygiene habits and regular dental check-ups can help prevent the development of periodontal disease.

1. How is diabetes related to periodontal disease?

Diabetes and periodontal disease have a complex bidirectional relationship, where each condition has the potential to influence the other.

1. **Impact of Diabetes on Periodontal Disease**:
   - **Higher Risk of Infection**: Individuals with diabetes, particularly when uncontrolled, have a higher susceptibility to infections due to a compromised immune system. This makes them more vulnerable to periodontal disease, which is essentially a chronic infection of the gums and the structures supporting the teeth.
   - **Blood Vessel Changes**: Diabetes causes thickening of the blood vessels, which can reduce the flow of nutrients and removal of waste products from body tissues, including the gums. This weakened tissue becomes more susceptible to infection and less capable of healing, thereby exacerbating the severity of periodontal disease.
2. **Impact of Periodontal Disease on Diabetes**:
   - **Glycemic Control**: Periodontal disease can make it more difficult to control blood sugar. The inflammation originating from periodontal disease may increase insulin resistance, which can disrupt blood sugar control, thus forming a vicious cycle between diabetes and periodontal complications.
   - **Systemic Inflammation**: The inflammation associated with periodontal disease may also contribute to the overall inflammatory burden of the body, further complicating diabetes management.

Effective management of one condition can have positive effects on the other. For instance, controlling blood sugar levels in diabetic patients can help reduce the risk of periodontal disease and potentially lessen its severity, while treating periodontal disease may help improve blood glucose control in diabetic patients, thereby reducing the risk of diabetic complications.

1. How is periodontal disease related to pregnancy?

There are some associations between periodontal disease and pregnancy. Changes in hormone levels in a woman's body during pregnancy can affect oral health, especially on the gums. Here are some of the main points of connection:

1. **Hormonal changes**: During pregnancy, levels of hormones such as estrogen and progesterone rise, which can make the gums more susceptible to inflammation, leading to inflammation, redness, and bleeding. This condition is sometimes referred to as "gingivitis gemisata."
2. **Exacerbation of periodontal disease**: If a pregnant woman had periodontal disease before she became pregnant, the condition may worsen during pregnancy. Periodontal disease is a chronic inflammatory disease of the tooth's supporting tissues that can lead to loosening or loss of teeth.
3. **Effects on the fetus**: Studies have shown that severe periodontal disease may be associated with an increased risk of preterm birth and low birth weight babies. Although the exact mechanism is not fully understood, it is thought that periodontal disease may affect pregnancy outcomes by affecting the level of inflammation in the body.
4. **Importance of oral hygiene**: Maintaining good oral hygiene during pregnancy is essential to prevent periodontal disease. This includes brushing your teeth regularly, flossing between your teeth, and getting regular dental check-ups.
5. **Nutrition and lifestyle**: Proper nutrition and a healthy lifestyle during pregnancy are also important to prevent periodontal disease. For example, adequate intake of vitamin C and vitamin D can help maintain gum health.

In summary, periodontal health during pregnancy is a concern, and pregnant women should work with their dentist to ensure that good oral health is maintained throughout pregnancy.

1. Does periodontal disease increase the risk of heart disease?

Yes, periodontal disease may increase the risk of heart disease. While the exact mechanism of association between periodontal disease and heart disease is not fully understood, studies suggest a certain link between the two.

Periodontal disease is a serious oral health problem that involves the tissues around the teeth, such as gums and bones. If left untreated, periodontal disease can lead to loose teeth or even loss of teeth. Periodontal disease causes a chronic inflammatory response that can affect other parts of the body, including the heart.

There are several mechanisms that may explain the link between periodontal disease and an increased risk of heart disease:

1. **Systemic inflammation**: Periodontal disease can lead to chronic inflammation within the mouth, which can spread throughout the body, including the cardiovascular system.
2. **Spread of bacteria**: Periodontal disease can cause harmful bacteria to enter the blood circulation, which can reach the heart and lead to heart disease.
3. **Atherosclerosis**: Periodontal disease may contribute to the development of atherosclerosis, a major cause of heart disease.

While the exact link between periodontal disease and heart disease is still being studied, maintaining good oral hygiene and regular dental check-ups is very important for overall health. If periodontal disease is present, it should be treated promptly to reduce the risk that may be associated with heart disease.

1. Does periodontal disease affect digestive disorders?

Periodontal disease can have an impact on the overall health of the body, including the digestive system. Periodontal disease is an inflammatory disease of the teeth and gums (tissues surrounding the teeth). This inflammation can lead to loose teeth and even tooth loss.

The effects of periodontal disease on the digestive system are mainly indirect. First of all, the teeth are the first step in the digestive process and are responsible for the chewing of food. Periodontal disease can cause painful or loose teeth, which in turn can affect chewing efficiency, which can lead to indigestion. Secondly, oral health is closely related to the health of the rest of the body. Periodontal disease can cause bacteria and inflammatory factors to enter the blood circulation, which can affect other parts of the body, including the digestive system.

While current research has not been able to directly prove a direct link between periodontal disease and specific digestive disorders, maintaining good oral hygiene is very important for overall health. Regular dental check-ups and cleanings, as well as good daily oral hygiene habits, can help prevent the onset and progression of periodontal disease.

1. How is periodontal disease treated?

Treatment of periodontal disease usually involves the following steps:

1. **Initial treatment and oral hygiene education**: This is the basic part of the treatment and includes professional teeth cleaning (e.g. scaling) to remove plaque and tartar from the teeth and gum margins. At the same time, the dentist will teach the proper way to brush and floss.
2. **Medications**: In some cases, antibiotics or other medications may be needed to help control infection and inflammation.
3. **Deep cleaning (periodontal scaling and root planing):** This is a more in-depth cleaning than regular scaling to remove plaque and tartar from the periodontal pockets and smooth the root surface to reduce bacterial attachment.
4. **Surgical treatment**: In more severe cases, periodontal surgery such as periodontal flap surgery, bone regeneration surgery, or periodontal plastic surgery may be needed to restore damaged periodontal tissue.
5. **Maintenance treatment**: Periodontal disease requires regular check-ups and professional cleaning after treatment to maintain oral health.

It is important to note that the treatment of periodontal disease needs to be individualized, and the specific treatment plan should be developed by the dentist according to the specific situation of the patient. At the same time, the patient's own oral hygiene habits and lifestyle modification are also key to the success of the treatment.

1. What should be paid attention to in the daily care of patients with periodontal disease?

Routine care for patients with periodontal disease is important to help manage the condition and prevent it from getting worse. Here are some key considerations:

1. **Brush your teeth correctly**: Use a toothbrush with soft bristles and use proper brushing techniques to avoid gum injuries caused by too hard brushing.
2. **Floss or gap brush**: Use a floss or gap brush daily to clean the gaps between your teeth, which can help remove food debris and plaque that is difficult to reach by brushing.
3. **Use mouthwash regularly**: Using an antibacterial mouthwash can help reduce bacteria in your mouth and reduce plaque formation.
4. **Regular check-ups and cleanings**: Visit your dental office regularly for dental check-ups and professional cleanings to remove tartar and plaque that is difficult to remove on your own.
5. **Maintain a good diet**: Avoid excessive intake of sugary foods and acidic beverages, which may worsen periodontal disease symptoms.
6. **Smoking cessation**: Smoking is one of the main causes of periodontal disease, and quitting smoking is very important to improve periodontal condition.
7. **Manage chronic conditions**: Chronic conditions, such as diabetes, can affect periodontal health, so these conditions should be managed properly.
8. **Reduced stress**: Long-term stress can affect the immune system and may worsen periodontal disease symptoms.
9. **Use proper toothpaste**: Use a toothpaste that contains fluoride or is specifically designed for periodontal disease to help protect your teeth and gums.
10. **Maintain overall oral hygiene**: Ensure hygiene throughout the oral cavity, including the cleanliness of the tongue and soft tissues of the mouth.

The above measures are general recommendations, and a dental professional should be consulted for personalized guidance and treatment recommendations.

1. **Problems related to oral mucosal diseases**
2. What is oral mucosal disease?

Oral mucosal diseases refer to a series of diseases that affect the oral mucosa. The oral mucosa is a moist layer of tissue that covers the inside of the mouth, including the lips, tongue, gums, floor of the mouth, and the inside of the cheeks and mouth. These diseases can be inflammatory, infectious, systemic, or even cancerous. Common oral mucosal diseases include:

1. **Canker sores**: This is one of the most common diseases of the oral mucosa and usually manifests as small painful sores.
2. **Oral candidiasis**: caused by infection with Candida (a fungus) and is common in individuals with weakened immune systems.
3. **Oral leukoplakia**: Appears as white patches in the mouth, sometimes precancerous.
4. **Oral submucosal fibrosis**: This is a chronic, irreversible condition that is often associated with betel nut chewing.
5. **Oral lichen planus**: This is a chronic inflammatory disease that appears as wavy or reticular white plaques in the mouth.
6. **Oral cancer**: a malignant tumor of the oral mucosa associated with smoking, alcohol consumption, and HPV infection.

Treatment for these conditions depends on the type and severity and may include medications, surgery, or other specialized treatments. If there are abnormal symptoms of the oral mucosa, a dental or dentist should be consulted promptly for diagnosis and treatment.

1. What are the common oral mucosal diseases?

Oral mucosal diseases refer to various diseases that occur on the oral mucosa, and there are many types of such diseases that can be classified according to their nature and manifestations. Here are some common oral mucosal diseases:

1. **Recurrent canker sores**: Also known as recurrent aphthous ulcers, they are one of the most common ulcerative diseases of the mouth and present as painful sores on the oral mucosa that usually heal on their own.
2. **Oral leukoplakia**: presents as a white plaque on the oral mucosa that cannot be erased, and its etiology is complex and can sometimes turn malignant.
3. **Oral lichen planus**: is a chronic inflammatory disease characterized by reticulated, annular or patchy white lines on the oral mucosa, which may be painful.
4. **Oral candidiasis**: caused by a Candida infection, it appears as white or red spots or plaques in the mouth that can be wiped off, and the patient may feel a pain in the mouth or a burning sensation.
5. **Oral herpes**: Caused by the herpes virus, it appears as small blisters on the mucous membranes of the mouth that may rupture to form painful sores.
6. **Oral submucosal fibrosis**: This is a condition associated with betel nut chewing that is characterized by hardening of the oral mucosa and limited opening.
7. **Dry mouth** (Sjogren's syndrome): Characterized by persistent dryness of the mouth, often caused by decreased saliva production, which can lead to difficulty swallowing, mouth sores and infection.
8. **Oral cancer**: including squamous cell carcinoma, it is the most serious type of oral mucosal disease, which is manifested as ulcers, lumps or hard spots on the oral mucosa, which do not heal and may be accompanied by pain.

Diagnosis and treatment of these disorders need to be determined by doctors based on medical history, clinical manifestations, and necessary laboratory tests. If you suspect that you have a disease of the oral mucosa, you should seek medical attention as soon as possible so that you can get proper diagnosis and treatment.

1. Can oral mucosal disease become malignant?

Some types of oral mucosal disease do carry a risk of malignant transformation, that is, transition from a benign state to a malignant neoplasm. This risk depends on the specific type of oral mucosal disease. The following are several cases of oral mucosa that may have the potential for malignant transformation:

1. **Leukoplakia**: This is a white patch that appears on the mucosa of the mouth and cannot be simply wiped off. Although most vitiligo is benign, some of them can become squamous cell carcinoma of the mouth. The rate of malignancy of vitiligo depends on a variety of factors, including the size, location, and appearance of the plaque, as well as whether it is accompanied by redness (erythemoplakia).
2. **Erythroplakia: Erythroplakia** is a red patch that appears on the oral mucosa and has a higher risk of malignant transformation than leukoplakia. The surface of erythema may be smooth, granular, or ulcerate, and in most cases erythema is considered to have a high potential for malignant transformation.
3. **Oral lichen planus**: Most oral lichen planus is benign, but some research suggests that erosive or ulcerative oral lichen planus, in particular, may be at some risk of malignant transformation. However, the exact risk of this malignant transformation remains controversial.
4. **Oral submucous fibrosis in Asians**: This is a chronic condition associated with betel nut chewing that can cause the oral mucosa to harden and restrict the opening of the mouth. This disease has a high risk of malignant transformation.

It is important to note that the malignant transformation of oral mucosal disease is not always predictable, therefore, regular monitoring and evaluation should be performed for any type of oral mucosal abnormality. If any new or persistent oral symptoms are found, a dentist or dental specialist should be consulted for evaluation. Early detection and treatment are key to preventing malignant transformation of oral mucosal diseases.

1. Are there any special oral cleaning products for people with oral mucosal disease?

For patients with oral mucosal disease, it is very important to choose the right oral cleaning products. These patients may have a more sensitive mouth and are susceptible to irritation. Here are some special oral cleaning products that are suitable for patients with oral mucosal diseases:

1. **Gentle toothpaste**: Choose a toothpaste that doesn't contain scrubs, strong fragrances, or other harsh ingredients. Some toothpastes are specifically designed for sensitive teeth or special oral conditions.
2. **Low-alcohol or alcohol-free mouthwashes**: Alcohol may irritate or cauterize sensitive oral mucosa. Choosing a gentle, alcohol-free mouthwash can help reduce irritation.
3. **Soft-bristled toothbrushes**: Using soft-bristled toothbrushes can reduce irritation of sensitive or damaged oral mucosa.
4. **Special dental floss**: For gums that bleed easily or are sensitive, a wax-coated or softer dental floss may be more appropriate.
5. **Oral** sprays: Some oral spray products contain ingredients that can help soothe the oral lining and reduce inflammation.
6. **Special products for oral diseases**: There are some special products on the market that are specifically designed for conditions such as canker sores, gingivitis, etc.

It is advisable to consult with a dentist or doctor before using these products to ensure that the product you choose is appropriate for your specific situation. If you have a more severe condition of your oral mucosa, you may need more specialized medical products or treatments.

1. Do patients with oral mucosal disease need more frequent oral examinations?

Oral mucosal disease is a class of diseases that affect the oral mucosa and may involve a variety of different pathological conditions, such as ulcers, inflammation, infection, immune-related diseases, etc. For patients with oral mucosal disease, it is true that more frequent oral examinations may be required for several reasons:

1. **Monitor changes in the condition**: Oral mucosal disease may have periodic episodes and remissions, so regular check-ups can help monitor changes in the condition and adjust treatment plans in a timely manner.
2. **Detect and manage complications promptly**: Some oral mucosal diseases can cause complications, such as infection or increased pain, and regular check-ups can help detect and manage these problems early.
3. **Prevention of exacerbations**: For some types of oral mucosal diseases, such as vitiligo, there is a risk of progression to oral cancer. Regular check-ups can help detect potentially malignant changes early.
4. **Evaluate the effectiveness of treatment**: Regular checkups can help doctors evaluate the effectiveness of their current treatment options and make adjustments if necessary.
5. **Maintain oral health**: Oral mucosal diseases can affect a patient's daily oral health care. Regular check-ups can help guide patients to proper oral hygiene management and prevent other oral problems.

The frequency of examinations should be determined by the dentist based on the specific condition and needs of the patient. Some patients may need to be checked every few months, while others may need more frequent check-ups. It is important to follow your doctor's advice to ensure oral health and timely treatment.

1. Are there any special dietary recommendations for people with oral mucosal disease?

Dietary recommendations for patients with oral mucosal disease are often aimed at reducing oral irritation, helping with symptom relief, and promoting oral health. Here are some general dietary recommendations:

1. **Avoid spicy, excessively hot, or excessively cold foods and drinks**: these foods may irritate or damage the already sensitive oral mucosa.
2. **Avoid acidic foods and beverages**: such as citrus fruits, carbonated beverages, etc., as they may irritate the lesion area.
3. **Avoid hard, coarse foods**, such as nuts, hard bread, etc., to reduce physical damage to the oral mucosa.
4. **Choose foods that are soft, easy to chew and swallow**: such as cooked vegetables, pureed fruits, tofu, etc.
5. **Adequate hydration**: Keeping your mouth moist can help relieve discomfort.
6. **Nutritionally balanced**: Make sure you get enough vitamins and minerals, especially B vitamins, vitamin C, zinc and iron, which are important for oral health.
7. **Avoid alcohol and tobacco**: these substances can irritate the oral mucosa and may worsen symptoms.
8. **Be aware of food allergies**: Some people may have allergic reactions to specific foods that should be avoided.
9. **Maintain oral hygiene**: While not a straightforward dietary recommendation, maintaining oral hygiene is very important for reducing inflammation and promoting healing.

Please note that these recommendations may vary depending on the individual situation, and if there is a specific oral mucosal disease, it is best to consult a doctor or dietitian for personalized dietary advice.

1. How to avoid recurrence of oral mucosal disease?

To avoid recurrence of oral mucosal disease, the following measures can be taken:

1. **Good oral hygiene habits**: Regular brushing, flossing, and regular use of mouthwash can help reduce bacteria and viruses in your mouth, which can reduce the risk of infection.
2. **Regular dental check-ups**: Visit your dentist or dentist regularly for oral health check-ups so that problems can be detected and treated early.
3. **Avoid irritating foods and drinks**: Spicy, excessively hot, or excessively cold foods and drinks may irritate the oral mucosa and should be avoided as much as possible.
4. **Quit smoking and limit alcohol consumption**: Both tobacco and alcohol are common causes of oral mucosal disease, and reducing or quitting these habits can help prevent relapse.
5. **Control chronic diseases**: Chronic diseases such as diabetes and autoimmune diseases can affect oral health and should be properly controlled.
6. **Avoid using a toothbrush that is too harsh**: Use a soft-bristled toothbrush and avoid overbrushing or pushing too hard to avoid damaging the oral mucosa.
7. **Managing stress**: Long-term psychological stress may affect the body's immune system and increase the risk of oral diseases.
8. **Supplementation**: Make sure you get enough vitamins and minerals, especially B vitamins, vitamin C, and zinc, which are important for maintaining oral health.

Keep in mind that these are only general recommendations, and for specific oral mucosal diseases, it is best to consult a professional dentist or dentist for personalized treatment and prevention advice.

1. Do patients with candidiasis infection need special oral protection?

Candidiasis is an infection caused by the fungus Candida, a fungus, that is commonly found in the mouth but can also affect other parts of the body. Patients with candidiasis do need special oral protection measures aimed at reducing the severity of the infection, preventing the spread of the infection, and promoting a quick recovery. These include:

1. **Good oral hygiene**: Brushing and flossing regularly can reduce the number of Candida in your mouth.
2. **Use of antifungal medications**: Your doctor may prescribe an antifungal cream or liquid to apply directly to the infected area of your mouth.
3. **Keep your mouth dry**: Candida grows faster in humid environments, so it's important to keep your mouth dry.
4. **Avoid irritating foods and drinks**: Spicy, acidic, or hard foods may irritate the infected mouth and should be avoided as much as possible.
5. **Change your toothbrush regularly**: Replace your toothbrush during and after recovery from infection to prevent reinfection.
6. **Oral saline gargle**: Gargling with warm salt water can help reduce oral discomfort and help disinfect.
7. **Control blood sugar and avoid smoking**: It is very important for people with diabetes to control their blood sugar levels, as high blood sugar promotes the growth of Candida. Smoking is also a risk factor and should be avoided.
8. **Regular check-ups**: Report changes in symptoms to your doctor regularly and follow their recommendations.
9. **Patients with dentures**: Make sure the dentures fit well and clean and disinfect them every night.

If you or someone you know is experiencing a Candida infection, it is advisable to consult a doctor or dentist for specific advice and treatment options specific to the individual situation.

1. How can I relieve canker sores on a daily basis?

There are many daily ways to relieve canker sores, and here are some effective suggestions:

1. **Maintain oral hygiene**: Brushing and flossing regularly can reduce the buildup of bacteria in your mouth, which can reduce the risk of ulcers.
2. **Avoid irritating foods**: Spicy, excessively hot, too hard foods may irritate pre-existing ulcers and should be avoided as much as possible.
3. **Supplementation with vitamins and minerals**: Nutrients such as B vitamins, vitamin C, zinc, and iron are important for oral health. If these nutrients are lacking in the diet, consider supplementing them with foods or supplements.
4. **Reduce stress**: Long-term mental stress can cause or exacerbate mouth sores. Engaging in light physical activity, meditation, or other relaxation techniques can help relieve stress.
5. **Use topical pain medications**: There are many types of canker sore gels or sprays on the market that can temporarily reduce pain.
6. **Get enough rest**: Getting enough sleep also contributes to the proper functioning of the immune system, which promotes the healing of ulcers.
7. **Avoid alcohol and tobacco**: Both alcohol and tobacco can irritate mouth sores and should be avoided as much as possible.
8. **Drink plenty of water**: Maintaining your body's water balance helps maintain oral health.

If canker sores occur frequently or for a long time, it is advisable to consult a medical professional for further examination and treatment.

1. Are there any ways in your daily life that can help improve the resistance of the oral mucosa?

There are several ways in daily life that can help improve the resistance of the oral mucosa, which is very important to prevent oral diseases and infections. Here are some tips for the health of your oral mucosa:

1. **Good oral hygiene**: Regular brushing and flossing can remove plaque and reduce bacterial growth, which is important for maintaining a healthy oral mucosa.
2. **Eat a balanced diet**: Make sure your diet contains enough vitamins and minerals, especially vitamin C, vitamin A, and zinc, which are especially important for mucosal health.
3. **Avoid tobacco and alcohol**: Both smoking and excessive alcohol consumption can damage the oral mucosa, reducing its resistance.
4. **Proper hydration**: Maintaining proper water intake can help keep your mouth moist and avoid dryness, which can make your mouth more susceptible to infections.
5. **Regular dental check-ups**: Regular visits to the dentist for dental check-ups and professional cleanings can detect and treat oral problems early.
6. **Stress reduction**: Long-term stress can affect the body's immune system, including oral resistance. Practicing stress-reducing techniques, such as deep breathing, meditation or yoga, can help maintain overall health.
7. **Avoid excessively hot or cold foods and drinks**: Excessively hot or cold foods and beverages may irritate or damage the oral mucosa.
8. **Maintain good overall health**: Chronic diseases such as diabetes can affect oral health, so managing these conditions is also important to keep the oral mucosa healthy.

The above recommendations can help maintain the health and resistance of the oral mucosa. If you have specific oral health concerns, it's best to consult with a dentist or other oral health professional.

1. **oral related problems in children**
2. Why does the baby delay teething?

There could be a few reasons why your baby is slow to teeth. Typically, babies begin to develop their first baby teeth around 6 months of age, but this time point can vary greatly from person to person. Here are some of the reasons why your baby may not be teething:

1. **Genetic factors**: If there is a history of late teething in the family, then the baby may also have teething later.
2. **Nutritional factors**: Malnutrition or deficiencies in certain nutrients, such as calcium, phosphorus, and vitamin D, may affect tooth growth.
3. **Endocrine problems**: Certain endocrine problems, such as an underactive thyroid, can cause delayed growth and development, including tooth growth.
4. **Low birth weight**: Babies born prematurely or born with a low birth weight may teething later than children of the same age.
5. **Specific health conditions**: Certain rare genetic diseases or health problems can also cause stunted tooth growth.

If you have any concerns about your baby's tooth growth, it is advisable to consult a pediatrician or pediatric dentist. They can provide professional advice and, if necessary, carry out further examinations. Usually, if your baby's overall health is good, teething a little later is not necessarily a sign of a problem.

1. How can I brush my baby's teeth?

Brushing your baby's teeth is an important hygiene habit that can be cultivated from soon after birth. Here are some tips for brushing your little one's teeth:

1. **Start time**: When the baby's first teeth start to erupt (usually around 6 months), it's time to start brushing.
2. **Choose the right tool**:
   - Use a toothbrush designed for babies that has a small head and soft bristles.
   - Baby toothpaste can be used, and the fluoride content should be lower than that of adult toothpaste. The amount used should be very small, no more than the size of a grain of rice.
3. **Brushing method**:
   - Gently brush each tooth and gum, taking care to clean all sides of your teeth.
   - Use gentle circular motions to ensure that the front, back and sides of your teeth are cleaned.
   - Do not push too hard to avoid injuring the baby's gums.
4. **Make it a habit** to brush your teeth at least twice a day, especially in the morning and before bedtime.
5. **Supervision and demonstration**: Parents need supervision and assistance throughout the entire process when babies are young. As children grow, they can gradually be taught to brush their own teeth, but they still need adult help and supervision until they are six years old.
6. **Regular check-ups**: Take your baby to the dentist regularly for oral health check-ups.
7. **Create fun**: The brushing process can be made fun by singing, telling stories, or using a fun toothbrush, which can help children build positive brushing habits.

Remember, a baby's dental health is very important to their overall health, so it's crucial to develop good oral hygiene habits from an early age.

1. What should I do if a new tooth grows in and the old one doesn't fall out?

If the old tooth is not lost when the new tooth comes in, this usually happens during the child's tooth replacement. This condition is known as "baby tooth retention". Normally, baby teeth loosen naturally and eventually fall out when permanent teeth erupt. But sometimes baby teeth don't fall out, causing permanent teeth to erupt in abnormal places.

For this case, the best course of action is to consult a dentist. Your dentist may recommend the following treatments:

1. Watchful waiting: If baby teeth start to loosen a little, your doctor may recommend watchful waiting to see if it falls out naturally.
2. Tooth extraction: If the baby teeth are completely unshaken, your doctor may recommend the removal of the baby teeth to provide enough space for the permanent teeth.
3. Orthodontic treatment: If a permanent tooth is misaligned and affects the bite or aesthetics, orthodontic treatment may be needed to correct it.

It is important not to attempt to remove baby teeth on your own, as this can lead to infection or other complications. It is important to have a professional dentist take care of this issue. At the same time, regular dental check-ups can help monitor the development of your child's teeth and deal with similar problems in a timely manner.

1. What happens when a newly erupted tooth has white patches or "pits" on the surface?

White patches or "pits" on the surface of newly erupted teeth can be caused by several conditions:

1. **Dental fluorosis**: This is caused by excessive intake of fluoride during tooth formation. Dental fluorosis is characterized by white or brown spots or streaks on the surface of the tooth, which may be accompanied by slight surface irregularities. While dental fluorosis can be cosmeticly problematic, it usually does not affect the function of your teeth.
2. **Enamel hypoplasia**: This is a problem with the development of teeth that can be caused by genetic factors, premature birth, malnutrition, or certain diseases such as adenovirus infection. Aenamel hypoplasia can lead to a decrease in tooth surface hardness, prone to spots and pits.
3. **Early signs of tooth decay (caries):** While tooth decay is often associated with black or brown spots, in the early stages, tooth decay may appear as white spots, especially in areas where the teeth come into contact with food and drink.
4. **Oral hygiene problems**: Sometimes, poor oral hygiene habits, such as improper brushing or infrequent flossing, can also lead to white patches on the surface of your teeth.
5. **Other factors** such as excessive intake of sugary foods and beverages, insufficient intake of minerals (such as calcium and phosphorus), medication side effects, etc., may also affect the health of your teeth.

If such changes are seen on newly erupted teeth, it is advisable to consult a dentist as early as possible to determine the specific cause and get appropriate treatment or advice. Remember, early diagnosis and intervention can be effective in preventing potential dental problems from progressing further.

1. How is caries in baby teeth treated?

Treatment of caries in deciduous teeth depends on the severity of the caries. Here are a few common treatments:

1. **Superficial caries treatment**: If the caries is shallow and confined to the enamel, remineralization of the enamel can usually be promoted with medication. This may include the use of fluoride toothpaste or other fluoride products.
2. **Moderate caries**: If the caries has invaded the dentin but has not yet reached the pulp (dental nerve), a filling is usually needed. The dentist will remove the decayed part and fill it with a suitable material such as composite resin or amalgam.
3. **Deep caries treatment**: If the tooth decay has invaded the pulp, root canal treatment may be required. This process involves removing the infected pulp tissue, cleaning and sterilizing the root canal, and then filling and sealing.
4. **Prevention and maintenance**: In addition to treating existing caries, it is also important to prevent new caries. This may include proper oral hygiene habits, regular dental check-ups, and the use of fluoride.
5. **Crown restoration**: In some cases, crowns may be needed to restore the shape and function of baby teeth if they are severely damaged.
6. **Extraction**: In rare cases, baby teeth may need to be removed if they have severe decay and cannot be repaired. However, because baby teeth are essential for the health and proper development of a child's mouth, extractions are usually avoided as much as possible.

Each treatment has its indications and limitations, so it is up to a professional dentist to decide which treatment option to choose on an individual basis. Regular dental check-ups are essential for the prevention and early detection of caries in deciduous teeth.

1. Does local anesthetic injections affect brain development in children?

Whether a child's receipt of local anesthetic medication affects brain development is a complex question that involves many factors. First, different anesthetic drugs have different components and mechanisms of action, and their potential effects on children's brains may also be different.

Current research suggests that local anesthesia is generally considered safe, especially when used in a short period of time and in moderation. Local anesthetic drugs act primarily on nerve endings at the site of application, reducing or blocking the transmission of pain signals, and usually do not have a systemic effect on the brain or central nervous system.

However, for children, especially infants, the brains are in a stage of rapid development, so the sensitivity to drugs may be different from that of adults. Some studies have shown that certain general anesthetic drugs may adversely affect brain development in infants and young children, especially if used for long periods of time or with repeated use. But these studies have focused mainly on general anesthetic drugs rather than local anesthetic drugs.

Overall, local anesthesia is generally considered safe if administered by an experienced medical professional when necessary and appropriately, with little risk of effects on brain development. However, every situation is unique and it is best to consult a relevant medical professional if there are any concerns or need detailed medical advice.

1. Does root canal treatment for deciduous teeth affect permanent teeth?

Root canal treatment for deciduous teeth (also known as root canal treatment for children) is a dental restoration procedure performed when an infection or damage occurs in a deciduous tooth. The purpose of this treatment is to remove infected or injured pulp tissue, clean and disinfect the root canal system, and then fill and seal the root canal to preserve the structure and function of the baby tooth until it falls out naturally, making way for the permanent tooth.

The impact of root canal treatment on permanent teeth is usually positive as it helps to keep baby teeth healthy until they are ready to erupt. By maintaining the position and health of baby teeth, permanent teeth can enter the mouth more correctly, helping to maintain occlusion and prevent tooth alignment problems.

However, if the root canal of a deciduous tooth is not treated properly, for example, if the infection is not fully controlled after root canal treatment, or if there is damage to the surrounding tissues during treatment, it may theoretically have a negative impact on the development of permanent teeth. These effects may include a change in the location of the permanent tooth eruption or a slight change in the color and shape of the permanent tooth.

Overall, primary tooth root canal treatment is an important dental treatment designed to maintain children's oral health and support the normal process of tooth development. As long as it is treated properly, it usually does not negatively affect permanent teeth. However, every case is unique, and if you have questions or concerns, it's best to consult with a dental professional for advice tailored to your individual situation.

1. Can a child's tooth be replanted?

If a child's tooth is a baby tooth (i.e. a newborn tooth from childhood), it is usually not replanted after it is knocked out because the baby tooth is eventually replaced by a permanent tooth. Baby tooth loss is a normal physiological process, and as the child grows, the baby teeth will fall out naturally, and the permanent teeth will then erupt.

This is not the case if the child is missing a permanent tooth. The loss of a permanent tooth is usually due to trauma or other reasons, and in this case, it is sometimes possible to try to replant the tooth back in place. The likelihood of a successful reimplant depends on a number of factors, such as how the tooth is treated after it has been lost, the time factor (the shorter the time the tooth has been out of the body, the more likely the reimplant is to be successful), and the condition of the injured tooth and gums.

If your child's permanent tooth is missing, it is advisable to take the following steps as soon as possible:

1. Try not to touch the root of the tooth and only hold the crown (i.e. the upper part of the tooth).
2. If the tooth is dirty, gently wash it with milk or saline, avoiding direct rinsing with tap water.
3. Place the tooth back into the socket as much as possible and have your child gently bite on a clean piece of cloth to secure the tooth.
4. If you can't put the socket back in right away, keep the tooth in milk or your child's saliva.
5. Contact a dentist immediately for emergency treatment.

It is important that such conditions should be managed and guided by a professional dentist as soon as possible.

1. Why do my child have recurrent mouth sores?

There are several reasons why your child has recurrent mouth sores:

1. **Malnutrition or vitamin deficiencies**: Deficiencies in B vitamins (especially B12 and B6), iron, zinc, and other nutrients can lead to mouth sores.
2. **Oral hygiene problems**: Poor oral hygiene habits can lead to a buildup of bacteria, which can trigger mouth sores.
3. **Immune system problems**: Certain immune system problems can make your child more susceptible to mouth sores.
4. **Stress or anxiety**: Mental stress and anxiety are also thought to trigger canker sores.
5. **Genetic factors**: Studies have shown a genetic predisposition to canker sores in some families.
6. **External irritants**: For example, spicy food, toothbrush irritation, or small wounds in the mouth can trigger ulcers.
7. **Other medical conditions**: For example, gastrointestinal problems, hormonal changes or certain medical conditions, such as herpes, can cause mouth sores.

If your child has frequent mouth sores, it is advisable to consult a doctor to determine the specific cause and get advice on appropriate treatment. At the same time, maintaining good oral hygiene, eating a balanced diet, and reducing stress may all help reduce the occurrence of mouth sores.

1. Why can't the baby's tongue stick out or have a "groove" when it sticks out? Do I need surgery?

Your baby's tongue may not stick out or have a "groove" when it sticks out, probably because the tongue-tie is a piece of tissue that connects the base of the tongue to the floor of the mouth. This condition is medically known as tongue-tie or tongue-tie abnormality.

1. **Identify tongue-tie that is too short**: Tongue-tie that is too short can make it difficult for your baby to stick out their tongue, or a groove may appear in the middle when they stick out their tongue. This can affect your baby's feeding, swallowing and pronunciation.
2. **When intervention is needed**: not all tongue-tie requires treatment. If your baby is able to feed and swallow normally and has no pronunciation problems, intervention is generally not necessary. However, if tongue-tie is too short to interfere with your baby's feeding or may affect speech later in life, your doctor may recommend surgery.
3. **Surgical treatment**: The surgical treatment of tongue-tie is usually a simple procedure called tongue-tie or tongue-tie release. Surgery is usually quick and low-risk.
4. **Observation and evaluation**: Before deciding whether to proceed with surgery, the doctor will evaluate your baby's feeding status, tongue range of motion, and other relevant factors.
5. **Seek professional advice**: If you are concerned about your baby's condition, it is best to consult a pediatrician or a specialist speech therapist who can provide professional assessment and advice.

It is important to note that each baby's situation is unique and needs to be assessed and managed individually. Consult a medical professional before making any decisions.

1. **questions related to oral prevention**
2. What is Oral Health?

Oral health refers to the health of the inside of the mouth, including the teeth, gums, tongue, and other soft and hard tissues. A healthy mouth is not just free of tooth decay or periodontal disease, but also includes the following aspects:

1. **Dental health**: no tooth decay (caries), no tooth damage, teeth aligned, good bite relationship.
2. **Healthy gums**: There is no inflammation of the gums, no redness, swelling or bleeding, and the gums fit snugly around the teeth.
3. **Healthy oral mucosa**: The oral mucosa (e.g., tongue, soft tissues inside the mouth) is free of ulcers, inflammation, or other abnormal changes.
4. **No bad breath**: The mouth is fresh and there are no persistent halitosis.
5. **Normal function**: Chewing, swallowing, speech, etc. are functioning normally, and are not affected by oral problems.

Maintaining oral health is not only good for your digestive system and overall health, but it also has a positive impact on your social interactions and mental health. Daily measures to maintain oral health include regular brushing, flossing between teeth, regular dental check-ups and cleaning, etc.

1. What causes bad breath?

Bad breath, also known as bad breath, can be caused by a variety of causes, including:

1. **Poor oral hygiene**: Infrequent brushing or flossing can lead to a buildup of food debris in the mouth, which can cause odors when bacteria break down these debris.
2. **Gum disease**: Such as periodontal disease, which is caused by bacteria building up between the teeth and gums, leading to inflammation and infection of the gums.
3. **Dry mouth**: Saliva helps clean the mouth and neutralize bacteria, and dry mouth, or xerostomia, can cause bad breath.
4. **Food and drink**: Certain foods and drinks, such as garlic, onions, and coffee, may temporarily cause bad breath.
5. **Smoking and alcohol**: These can lead to changes in the oral environment and cause bad breath.
6. **Certain diseases and conditions**, such as diabetes, gastroesophageal reflux, liver or kidney disease, etc., can also cause bad breath.
7. **Infections in the mouth**: such as pulpitis, mouth ulcers, etc.
8. **Medications**: Some medications can cause dry mouth or release odorous chemicals during decomposition.

If bad breath persists, it is advisable to consult a doctor or dentist to determine the cause and get treatment recommendations.

1. What to do about bad breath?

Bad breath, also known as bad breath, can be caused by a variety of causes, including poor oral hygiene, gum disease, dry mouth, eating habits, certain foods, smoking, and certain health conditions. To solve bad breath, here are a few things you can try:

1. **Practice good oral hygiene**: Brush your teeth at least twice a day, floss to remove food debris and bacteria from between your teeth, and use a fluoride mouthwash regularly.
2. **See your dentist regularly**: Get teeth cleaned and checked every 6 months to prevent periodontal disease and other oral health problems.
3. **Keep your mouth moist:** Dry mouth can lead to bad breath. Drinking plenty of water can help keep your mouth moist. Sometimes, using saliva substitutes may also help.
4. **Avoid or reduce your intake of certain foods and beverages**: such as garlic, onions, and coffee, which may cause bad breath.
5. **Quit smoking and avoid tobacco products**: Smoking is not only harmful to health, but it can also lead to worsening of bad breath.
6. **Check your diet**: Certain low-carb diets can cause "keto breathing," a special type of bad breath that occurs due to the body burning fat instead of sugar.
7. **Consult a doctor**: If you have taken the above steps and bad breath persists, you may want to consult a doctor, as it may be a sign of a deeper health problem, such as stomach problems, diabetes, or kidney disease.

Keep in mind that if bad breath persists, it's best to consult a doctor or dentist to determine if there is an underlying health problem.

1. How can I prevent tooth decay?

Preventing tooth decay can be achieved in several ways:

1. **Good oral hygiene**: Brush your teeth at least twice a day, especially in the morning and before going to bed at night. Using fluoride toothpaste can help prevent tooth decay.
2. **Flossing**: Flossing daily to remove food debris and plaque from between your teeth is important to prevent both periodontal disease and tooth decay.
3. **Visit your dentist regularly**: Visiting a dental clinic every six months or a year for a professional dental check-up and cleaning can detect and treat tooth decay early.
4. **Dietary control**: Reduce the consumption of foods and drinks that are high in sugar, as sugar is one of the main causes of tooth decay. Improving your diet and eating more calcium- and phosphorus-rich foods, such as dairy, can help strengthen your teeth.
5. **Oral protection**: For athletes, the use of oral protectors can prevent dental injuries caused by exercise.
6. **Oral health education**: Understanding the importance of oral health and proper oral hygiene habits is also very important to prevent tooth decay.
7. **Proper use of fluoride**: Using fluoride under the guidance of a dentist can help strengthen teeth and prevent tooth decay.

Not only do these methods help prevent tooth decay, but they also have a positive impact on overall oral health. However, the specifics may vary from person to person, and it is advisable to consult your dentist regularly for personalized advice and treatment.

1. What is the correct way to brush my teeth?

The proper way to brush your teeth mainly includes the following steps:

1. **Choose the right toothbrush and toothpaste**: Use a soft-bristled toothbrush and fluoride toothpaste.
2. **Hold the toothbrush correctly**: Hold the toothbrush in a comfortable way with your fingers resting on the handle of the toothbrush.
3. **Toothbrush placement**: The toothbrush should be at a 45-degree angle to the junction of the teeth and gums.
4. **Brushing action**:
   - **Brush gently**: Brush your teeth with a gentle and smooth motion, avoiding excessive force.
   - **Brushing sequence**: You can brush the inside first, then the outside, and finally the occlusal surface.
   - **Brushing time**: Brush your teeth for at least 2 minutes at a time, making sure each area is cleaned.
5. **Tongue brushing**: Gently scrub the surface of the tongue to remove bacteria and food debris.
6. **Gargle**: Rinse your mouth thoroughly with water after brushing your teeth to remove loose food debris and bacteria.
7. **Replace your toothbrush regularly**: It is recommended to replace your toothbrush every three months, or when the bristles are worn out.
8. **Regular dental check-ups**: It is recommended to have dental check-ups every six months to a year.

These steps can help effectively remove plaque and food debris from your teeth, prevent tooth decay and gum disease, and maintain oral health. Of course, everyone's oral condition is different, and the specific brushing method may need to be adjusted according to the dentist's recommendations.

1. What are the types of toothpaste?

There are many types of toothpastes that can be classified according to their main functions and ingredients. Here are some common types of toothpaste:

1. **Anti-cavity toothpaste**: Contains fluoride and is mainly used to prevent tooth decay.
2. **Whitening toothpaste**: Contains bleach or scrub ingredients to help remove stains from the surface of your teeth.
3. **Anti-allergy toothpaste**: contains ingredients such as potassium ions to reduce tooth sensitivity.
4. **Gum Care Toothpaste**: Contains anti-inflammatory or circulation-boosting ingredients to improve gum health.
5. **Children's toothpaste**: It generally contains a lower concentration of fluoride and is mildly formulated and suitable for children.
6. **Herbal/Natural Toothpaste**: Mainly use natural ingredients such as herbal essential oils and avoid chemical additives.
7. **All-round protection toothpaste**: comprehensive multi-effect, such as anti-cavity, whitening, antibacterial, etc.
8. **Bad breath toothpaste**: Contains ingredients that freshen your breath, such as peppermint.

The specific ingredients and efficacy of each toothpaste may vary, so it is recommended to choose the right toothpaste according to your needs and dental condition. At the same time, visit your dentist regularly for check-ups to ensure your oral health.

1. How can pregnant women protect their oral health?

It is important for pregnant women to protect their oral health, as hormonal changes during pregnancy can affect oral health and increase the risk of gingivitis and dental problems. Here are some tips to help pregnant women maintain their oral health:

1. **Brush and floss regularly**: Pregnant women should brush their teeth at least twice a day and floss between their teeth to reduce the accumulation of plaque and tartar.
2. **Use fluoride toothpaste**: Using fluoride toothpaste can help prevent tooth decay.
3. **Regular dental check-ups**: It's important to see your dentist regularly for check-ups and cleanings during pregnancy.
4. **Dietary control**: Reduce your intake of high-sugar foods and beverages to reduce the risk of tooth decay.
5. **Increase your intake of calcium and vitamin D**: This is essential for the health of your teeth and gums.
6. **Oral hygiene education**: Learn about the importance of oral health during pregnancy and how to protect it.
7. **Avoid alcohol and tobacco**: Both tobacco and alcohol can harm oral health.
8. **Dealing with nausea and vomiting**: Morning sickness can cause damage to your teeth. After spitting, rinsing your mouth with water can help reduce the erosion of your teeth by stomach acid.

If you have any oral problems during pregnancy, such as bleeding gums, toothache or other discomfort, you should consult your dentist promptly. Remember, maintaining oral health during pregnancy is not only good for the mother herself, but also has a positive impact on the health of the fetus.

1. What is Oral Preventive Care?

Preventive oral care is a set of measures and habits aimed at preventing the onset and progression of oral diseases, as well as maintaining oral health. It includes the following main aspects:

1. **Daily oral hygiene maintenance**: Includes proper brushing techniques and flossing to remove food debris and plaque near the teeth and gum line. This helps prevent tooth decay and periodontal disease.
2. **Regular dental check-ups**: Regular visits to the dentist for check-ups and professional cleanings. This helps in the early detection and treatment of oral problems and prevents the disease from getting worse.
3. **Dietary control**: Reduce your intake of sugary foods and beverages, as sugar is a major contributor to tooth decay. At the same time, increasing foods rich in calcium and phosphorus, such as dairy products, can help strengthen teeth.
4. **Quit smoking and limit alcohol**: Smoking and excessive alcohol consumption have been linked to oral cancer and other oral health problems.
5. **Use a toothpaste that contains fluoride**: Fluoride can help strengthen teeth and prevent tooth decay.
6. **Children's oral health care**: This includes the use of appropriate levels of fluoride and prompt treatment of decay in baby teeth, as well as supervision of children's brushing habits.
7. **Oral protection**: Wear appropriate oral protective equipment when participating in activities that may cause facial injuries, such as certain sports.

Through these measures, the risk of oral diseases can be effectively reduced and overall oral health can be maintained.

1. How to maintain oral hygiene?

Maintaining oral hygiene is very important not only to help keep your breath fresh, but also to prevent tooth and gum disease. Here are some basic oral health care recommendations:

1. **Brush your teeth regularly**: Brush your teeth at least twice a day and use a fluoride toothpaste. Make sure to clean every surface of your teeth when brushing.
2. **Floss:** Floss at least once a day to remove food debris and plaque between your teeth.
3. **Use mouthwash regularly**: Using mouthwash can help kill bacteria in your mouth and reduce plaque and bad breath.
4. **Regular dental check-ups**: Regular check-ups and cleanings at the dentist every six months can detect and treat oral problems early.
5. **Reduce your intake of sugar and acidic foods**: These foods can increase the risk of tooth decay and tooth erosion.
6. **Don't smoke**: Smoking increases the risk of oral cancer and periodontal disease.
7. **Maintain a healthy diet**: Eat foods rich in vitamins and minerals, such as fruits and vegetables, which are good for dental health.
8. **Use a dental protector:** If you participate in physical activity, especially those with a risk of impact, using a dental protector can avoid damage to your teeth.

These steps can help you maintain your oral health and reduce your risk of dental and gum problems. Remember, regular visits to the dentist and following their recommendations are essential for maintaining good oral hygiene.

1. What is the relationship between oral diseases and systemic diseases?

There is an interrelated relationship between oral diseases and systemic diseases. These relationships are mainly manifested in the following aspects:

1. **Oral diseases may be indicators of systemic disease**: Certain systemic diseases, such as diabetes, hematologic disorders, heart disease, certain autoimmune diseases, etc., may show early symptoms in the oral cavity. For example, bleeding gums, mouth sores, loose teeth, etc. can be early signs of poor general health.
2. **Oral diseases can worsen health problems throughout the body**: Poor oral health, such as severe periodontal disease, may increase the risk of other diseases. For example, periodontal disease is associated with an increased risk of heart disease and stroke. Bacteria can enter the bloodstream from the mouth and affect other parts of the body.
3. **Systemic diseases may affect oral health**: Certain systemic diseases or their treatments can negatively affect oral health. For example, people with diabetes are more likely to develop periodontal disease, and anti-cancer treatments can cause mouth sores or dryness.
4. **Medication side effects**: Some medications used to treat systemic diseases can have side effects on the mouth. For example, certain medications can cause dry mouth, which increases the risk of tooth decay and gum disease.
5. **Lifestyle factors**: Certain lifestyle factors, such as diet, smoking, and drinking habits, can affect both general and oral health.

Therefore, maintaining good oral hygiene is not only important for the prevention of oral diseases themselves, but also an important aspect of maintaining overall health. At the same time, regular oral examinations can help detect signs of systemic diseases early.

1. **problems related to dental restorations and dental implants**
2. Do I need bone grafting before dental implants?

Whether or not bone grafting is needed prior to dental implantation (i.e. dental implant surgery) depends on the individual's specific situation. Key considerations include:

1. **Bone mass and quality**: If your alveolar bone (the bone that supports your teeth) is low in volume or quality, bone grafting surgery may be needed. This is because dental implants require sufficient bone mass to support and stabilize.
2. **Missing teeth**: If the tooth is missing for a long time, the alveolar bone may gradually shrink and bone grafting may be needed.
3. **Special case of the implant site**: Some areas of the mouth may have lower bone mass or quality due to anatomy or previous dental disease.
4. **Overall health**: People with certain diseases or conditions like diabetes, osteoporosis, etc., may need additional considerations and treatment steps.

Before deciding whether or not to proceed with bone grafting surgery, the dentist will usually perform a detailed oral examination and may use X-rays or CT scans to assess bone mass and quality. Bone grafting surgery can use autologous bone (bone taken from another part of the patient's own body), allogeneic bone (from a donor), xenogeneic bone (bone of animal origin), or synthetic bone as a substitute material.

It is advisable to consult a professional dentist for professional advice and advice tailored to your individual situation.

1. What are the consequences of missing teeth not being repaired?

Missing teeth without restoration can lead to a range of oral health problems and other consequences:

1. **Displacement of adjacent teeth**: Missing teeth may cause adjacent teeth to tilt or move towards openings, which can affect the occlusal relationship and cause the teeth to be misaligned.
2. **Overgrowth of the opposite tooth**: When a tooth is missing, the corresponding upper or lower teeth may overgrow due to the lack of counterbites, which can also affect the bite.
3. **Decreased chewing efficiency**: Missing teeth can lead to decreased chewing efficiency, which in turn can affect the health of the digestive system.
4. **Jaw atrophy**: Long-term tooth loss can lead to gradual recession of the alveolar bone, which not only affects the appearance of the face, but also makes future dental restoration more difficult.
5. **Pronunciation problems**: Teeth play an important role in pronunciation, and missing teeth can lead to slurred speech.
6. **Decreased self-confidence**: Missing teeth can affect an individual's appearance, which can affect self-confidence and social interactions.
7. **Increased risk of periodontal disease**: Edentulous areas are more likely to accumulate food debris and bacteria, increasing the risk of periodontal disease.
8. **Other teeth damaged**: Other teeth may be subjected to additional stress due to the redistribution of chewing force, increasing the risk of wear and damage.

Therefore, if there is a missing tooth, it is advisable to consult a dentist for proper treatment recommendations. Depending on the situation, possible restoration options include bridges, dentures, or dental implants, among others.

1. Do fixed crowns need to be replaced regularly?

Fixed crowns usually do not need to be replaced regularly, but their longevity can be affected by a variety of factors. In general, the average lifespan of a fixed crown is between 5 and 15 years, or even longer. It depends on several factors:

1. **Type of material**: Crowns can be made from a variety of materials, such as tiles, metal, or a combination of tiles and metal. The durability and longevity of different materials may vary.
2. **Oral hygiene habits**: Good oral hygiene habits, including regular brushing and flossing, can help prolong the life of crowns.
3. **Occlusion and wear**: The bite force and degree of wear on a crown can affect its longevity. Habits such as habitual chewing on hard objects or grinding teeth may accelerate crown wear.
4. **Regular dental check-ups**: Regular dental check-ups can help detect and address problems that may affect the longevity of a crown.
5. **Installation quality of dental crowns**: The quality of the manufacture and installation of dental crowns is also an important factor affecting its longevity.

If the crown is loose, cracked, or has gum problems, you should consult a dentist promptly. In some cases, the crown may need to be replaced. However, not all fixed crowns need to be replaced regularly, and in many cases, they can be used normally for a long time.

1. Is there an age limit for dental implants?

Dental implants are usually age-restricted. This is mainly because dental implants require the patient to have good alveolar bone condition, as well as overall health.

1. **Lower age limit**: Dental implant surgery is generally not recommended for minors because their jaws may still be growing and developing. Dental implants in minors may affect the normal development of the jaws. Usually, dental implants are not considered until around the age of 18, when the jaw development is basically completed.
2. **Upper age limit**: For older patients, there is no absolute upper age limit, the key is the patient's overall health and the quality of the alveolar bone. Older patients may also be considered for dental implant surgery if they have sufficient alveolar bone quality and do not have serious systemic diseases (e.g., severe heart disease, diabetes, etc.).

Therefore, whether dental implants are suitable or not needs to be decided according to the individual's specific situation, including age, alveolar bone condition, overall health, etc. Before considering dental implants, it is advisable to consult a professional dentist for a thorough evaluation.

1. What is the difference between removable dentures, porcelain teeth, and dental implants, and how to choose?

Removable dentures, porcelain teeth, and dental implants are the three common methods of restoring missing teeth, each with its own characteristics and indications:

1. **Removable dentures (removable dentures):**
   - **Features**: Removable dentures are removable dentures that are suitable for people who are missing some or all of their teeth. These dentures can be removed for cleaning, which is more convenient.
   - **Indications**: It is suitable for patients who have a large number of missing teeth, or who are not physically suitable for porcelain teeth and dental implant surgery.
   - **Advantages**: relatively low cost, quick to make, less invasive to the oral cavity.
   - **Disadvantages**: It doesn't feel as good as fixed dentures, it may take a while to get used to, and the chewing effect is not as good as porcelain teeth or dental implants.
2. **Porcelain tooth (fixed bridge)**:
   - **Features**: A porcelain tooth is a type of denture that is fixed to the patient's original tooth and requires grinding of adjacent healthy teeth for support.
   - **Availability**: Applies if a single or a few teeth are missing and the surrounding teeth are healthy.
   - **Advantages**: Beautiful, good chewing, comfortable to wear, feels close to real teeth.
   - **Disadvantages**: Healthy teeth need to be grinded, irreversible; If there is a problem with the supporting teeth, the entire bridge may need to be redone.
3. **Dental Implants**:
   - **Features**: A titanium "tooth root" is implanted into the alveolar bone of the edentulous area, on which a denture crown is attached.
   - **Indications**: Suitable for patients with single or multiple missing teeth and good alveolar bone condition.
   - **Advantages**: The function and aesthetics are closest to natural teeth, no need to grind adjacent teeth, and simple maintenance.
   - **Disadvantages**: high cost, long treatment cycle, relatively high surgical risk.

When choosing the right dentures, the following factors should be considered:

- **Degree of tooth loss**: The number and location of missing teeth can affect the choice.
- **Oral health**: the health of the alveolar bone, the condition of the adjacent teeth, etc.
- **Age and health**: Age and overall health may affect the risk of surgery and the ability to recover.
- **Economic** considerations: The cost of different treatment options varies widely.
- **Personal preferences**: including requirements for aesthetics, comfort, etc.

It is advisable to consult a professional dentist before making a decision to choose the most suitable treatment plan according to your specific situation and the doctor's advice.

1. Do I need to get a dental implant immediately after a tooth extraction?

Whether or not dental implants are needed immediately after a tooth extraction depends on a variety of factors, including the condition of the individual's teeth, overall health, and specific treatment plan. Here are some considerations:

1. **Healing process after tooth extraction**: After tooth extraction, the alveolar bone (the bone where the tooth sits) will begin to heal. In some cases, your dentist may recommend waiting a period of time after the tooth extraction to allow the alveolar bone to heal sufficiently before proceeding with the implant procedure.
2. **Immediate implants**: In some cases, your dentist may recommend an implant procedure on the same day as the tooth extraction. This is usually done if the alveolar bone is in good condition and there are no other complications.
3. **Patient's health**: The overall health of an individual can also influence the decision to get dental implants. For example, diabetics or severely ill smokers may require special consideration.
4. **Dentist's advice**: The most important thing is to follow the professional advice of your dentist or oral surgeon. They will suggest the most suitable treatment plan for your specific situation.

If you're considering dental implants, it's a good idea to discuss your specific situation and treatment options with your dentist in detail.

1. How often should I get teeth after tooth extraction?

The time interval between dental implants after tooth extraction depends on several factors, including the healing process after tooth extraction, the overall health of the patient, and the specifics of the dental implant. In general, here are some common timelines:

1. **Immediate implants**: In some cases, teeth can be implanted on the same day as the tooth extraction. This is usually done if there is no significant bone defect after tooth extraction.
2. **Early implantation**: This is usually done about 2-3 months after the tooth extraction, when the soft tissue has healed but the bone tissue has not been completely rebuilt.
3. **Delayed implantation**: Performed 4-6 months after tooth extraction, allowing the bone and soft tissues to heal adequately.
4. **Late implantation**: In some cases, it may take longer, such as more than 6 months, especially when additional bone grafting or other complex procedures are required.

It is important to consult with a dental professional to find out which timeline fits into your specific situation. Everyone's situation is different, so the best time to grow should be decided based on the individual's healing and overall health.

1. Are dental implants lifelong and how long can they last?

Dental implants are often designed as long-term solutions that can even last a lifetime. However, their actual lifespan is affected by a variety of factors, including the patient's oral hygiene habits, overall health, the quality of the dental implants, and the skills of the doctor performing the implant surgery.

1. **Oral hygiene**: Good oral hygiene is key to keeping dental implants stable for a long time. This includes brushing your teeth regularly, flossing, and seeing your dentist regularly.
2. **Overall health**: Certain health conditions, such as diabetes or osteoporosis, can affect the stability and longevity of dental implants.
3. **Quality of dental implants**: Dental implants that use high-quality materials and advanced technology are generally more durable.
4. **Doctor's Technique**: The experience and skill of the dentist performing the implant procedure is essential to the success and durability of the implant.

In the best case, dental implants can last for 20 years or more. However, they may require maintenance and regular inspections to ensure that they are kept in top condition. Some patients may need to replace the crown (the upper structure of the dental implant) after a period of time, even if the implant (the part of the implanted bone) is still secure. In conclusion, dental implants are a long-lasting solution, but require the patient, dentist, and regular care to ensure their maximum effectiveness and longevity.

1. How should dental implant food impaction be removed?

Food impaction after dental implants is a common problem and should be cleaned with the following points:

1. **Use a soft-bristled toothbrush and gentle brushing**: Use a soft-bristled toothbrush and brush your teeth gently to avoid unnecessary stress or damage to the implant.
2. **Flossing and interdental brushes**: Flossing or interdental brushes can help remove food debris around and between dental implants. For dental implants, it is best to choose dental floss or an interdental brush that is specifically designed for dental implants, which will allow for better cleaning and less damage to the implant.
3. **Water flosser**: A water flosser is a gentle and effective cleaning tool that uses a high-pressure stream of water to remove food debris near the teeth and gum line.
4. **Regular dental check-ups**: Regular visits to the dental office for professional dental check-ups and cleanings can help maintain dental implant hygiene and prevent possible problems.
5. **Avoid using hard or sharp objects for cleanup**: Do not use toothpicks or other sharp objects to forcibly remove impacted food, which may damage dental implants or gums.
6. **Follow your dentist's advice**: Follow your dentist's specific recommendations for daily care, as everyone's oral condition and the specifics of dental implants may vary.

If you notice unusual pain, swelling or bleeding near your dental implants, you should consult your dentist promptly. Proper and regular cleaning can help maintain the health of dental implants and prolong their lifespan.

1. What should I do if my dental implant is inflamed?

If your dental implant is inflamed, there are a few steps you should take to deal with it:

1. **Seek medical attention promptly**: First, you should contact your dentist or dental implant specialist as soon as possible so that they can assess your condition and provide professional treatment advice.
2. **Oral hygiene**: It is very important to maintain good oral hygiene. Brush and floss regularly and correctly as directed by your doctor. Using a mild mouthwash may help reduce inflammation and prevent infection.
3. **Avoid irritating foods**: During treatment, avoid eating hard foods or foods that are too hot/too cold that may irritate or injure the dental implant area.
4. **Take medications**: If your doctor prescribes antibiotics or other medications, be sure to take them as directed to help control infections and reduce inflammation.
5. **Regular follow-ups**: Even if symptoms improve, follow your doctor's instructions for follow-up check-ups to ensure that inflammation is fully controlled and to prevent future recurrences.
6. **Rest and de-stress**: Maintaining adequate rest and reducing stress can also help the body recover.

Keep in mind that these are general recommendations and need to be handled based on your specific condition and your doctor's guidance. If the inflammation is severe or accompanied by other symptoms, such as fever, severe pain, etc., you should seek medical attention immediately.

| accuracy | completeness | structure | Professionalism | Overall level |
| --- | --- | --- | --- | --- |
|  |  |  |  |  |

1. **Orthodontics-related problems**
2. Crooked teeth, what conditions are suitable for orthodontics?

Crooked teeth can be improved with orthodontic treatment. Conditions that are suitable for orthodontics typically include:

1. **Crowded teeth**: Teeth are not aligned due to lack of space.
2. **Dental gap**: There is an abnormal gap between the teeth.
3. **Occlusal problems**: including crossbite (maxillary teeth behind the mandibular teeth), deep coverage (maxillary anterior teeth overcovering the mandibular anterior teeth), open jaw (vertical space between the upper and lower teeth), etc.
4. **Jaw dysplasia**: such as mismatch between the length of the upper and lower jaws.
5. **Abnormal tooth development**: such as teeth that are not of the same size or shape.

When deciding whether or not to undergo orthodontic treatment, it is best to consult a professional dentist. The doctor will determine the most appropriate treatment plan based on the individual's specific situation, including dental condition, age, oral health status and other factors. Orthodontic treatment is not only for aesthetics, but also to help improve occlusal function and oral hygiene.

1. Does orthodontics require tooth extraction? Which teeth are extracted? Will tooth extraction affect normal chewing?

Whether or not orthodontics requires tooth extraction and which teeth to extract depends mainly on the individual's oral condition and the specific needs of orthodontic treatment. Here are some basic guidelines:

1. **Whether or not a tooth extraction is required**: Not all orthodontic treatments require a tooth extraction. Tooth extractions are usually done to get enough space to correct tooth alignment, improve occlusion, or relieve crowding. Tooth extraction may be necessary if the patient's teeth are too crowded or if the occlusal relationship needs to be adjusted drastically.
2. **Which teeth are extracted**: The most commonly extracted teeth are the first or second premolars, depending on the amount of space needed and the specific alignment of the teeth. In some cases, wisdom teeth or other teeth may also be removed. The choice of tooth extraction is determined by the orthodontist based on the patient's oral health and treatment goals.
3. **Effect of tooth extraction on chewing**: After tooth extraction, there may be some impact on chewing function for a period of time, especially if the molars used for chewing are removed. However, chewing function usually improves as the teeth gradually move into the desired position through orthodontic treatment. Proper tooth alignment and bite relationships are beneficial for long-term oral health and function.
4. **Note**: When considering tooth extraction as part of orthodontic treatment, it is important to have adequate communication with the orthodontist to discuss the treatment plan, possible risks, and expected treatment outcomes. The orthodontist will provide professional advice based on the patient's specific situation.

If you are considering orthodontic treatment and are concerned about tooth extraction, it is recommended to consult a professional orthodontist for a detailed oral examination and personalized treatment plan.

1. Can my teeth line up after straightening?

Straightening teeth often allows for a straighter alignment of teeth. Orthodontic treatment aims to address various oral problems such as misaligned teeth, crowded teeth, teeth with too much gap, bite problems, etc. Treatments include traditional metal braces, clear aligners such as Invisalign, and more. The effectiveness of the treatment depends on the individual's specific situation, such as the current condition of the teeth, the type of orthodontic treatment, and the degree to which the patient cooperates with the treatment.

Before starting treatment, the dentist conducts a thorough assessment, including taking X-rays, a model of the mouth, and a detailed oral examination. Based on this information, the dentist will develop a personalized treatment plan.

It is important to note that each person's situation is unique, so treatment outcomes may vary from person to person. The course of treatment can take a while, usually from a few months to several years, and the retainer needs to be worn continuously after the treatment to maintain the results.

If you are considering orthodontics, it is advisable to consult a professional dentist for more accurate and personalized advice.

1. How much does orthodontics improve the mouth protrusion?

Orthodontic treatment can have a significant improvement effect on the protrusion of the mouth (commonly known as "ground wrapping"), but the exact effect depends on a variety of factors. The mouth protrusion is a condition in which the jaw bone is unbalanced, resulting in abnormal tooth alignment and/or protruding mandibles. Orthodontic treatment mainly involves the use of braces, aligners, or other orthodontic devices to adjust the position of the teeth and the relationship between the jaws.

The effectiveness of treatment depends on several factors:

1. **Severity of the case**: Mild cases of oral protrusion can be well improved with orthodontic treatment, but more severe cases may require a combination of maxillofacial surgery.
2. **Patient's age**: Children and adolescents often respond more aggressively to orthodontic treatment because their jaws are still developing. In adulthood, the jaw is fixed, and treatment can be more complicated.
3. **Individual differences**: Oral structure, tooth alignment, and jaw development are different for each person, and these factors can affect the effectiveness of orthodontic treatment.
4. **Treatment options**: Different orthodontic methods and equipment, such as traditional metal braces, invisible appliances or maxillofacial correction surgery, are suitable for different degrees of oral protrusion.
5. **Patient cooperation**: The patient's level of cooperation during treatment, including regular follow-up visits, adherence to doctor's instructions, and good oral hygiene habits, has a significant impact on the outcome of the treatment.

It is recommended to consult a professional orthodontist to develop the most suitable treatment plan for you through a comprehensive oral examination and individualized assessment. Orthodontic treatment is a long-term process that requires patience and persistence from the patient.

1. Can orthodontics adjust the occlusion?

Yes, orthodontic treatment can be used to adjust the occlusion. Occlusal problems are when the upper and lower teeth are not properly aligned when closed, which can lead to a variety of problems such as difficulty chewing, problems speaking, tooth wear, and maxillofacial pain.

Orthodontic treatment uses devices such as braces, aligners, clear aligners, etc., to gradually move the teeth and jaws to improve the alignment of the teeth and the alignment of the upper and lower jaws. This treatment can correct a variety of occlusal problems such as open jaw (the space between the upper and lower teeth), deep coverage (too much upper teeth covering the lower teeth), crossbite (the lower teeth are located before the upper teeth), etc.

It is important to note that the effectiveness of orthodontic treatment depends on the individual's specific situation, such as age, type and severity of bite problems, etc. Therefore, an orthodontist should be consulted to determine the most suitable treatment option before proceeding with orthodontic treatment.

1. Will orthodontics change my face shape?

Orthodontic treatment may have an impact on the shape of your face. Orthodontic treatment is mainly about adjusting the position of the teeth and the occlusal relationship, but due to the close relationship between the teeth and the jawbone, this adjustment may indirectly affect the facial contour.

1. **Tooth alignment** adjustments: Changes in tooth alignment may make the face shape look more harmonious. For example, if your teeth are crowded or uneven, aligning your teeth more neatly after orthodontic treatment may make your face look more defined.
2. **Improvement in occlusion**: Orthodontic treatment can also improve the occlusal relationship of the upper and lower jaws. The effect of this change on the shape of the face varies from person to person, but in some cases it can be quite significant, especially if your bite problem involves a change in the position of the jawbone.
3. **Jaw Position Adjustment**: In some complex cases, orthognathic surgery may be required to assist in orthodontic treatment, in which case the impact on the face shape will be more pronounced.

It is important to note that everyone's situation is different, and the results of orthodontic treatment also vary from person to person. If you are considering orthodontic treatment and have questions or concerns about possible facial changes, it is advisable to consult a professional orthodontist. They can provide a more detailed consultation and an explanation of what to expect based on your specific situation.

1. Are there any precautions to be taken during correction?

During orthodontic work, I assume that you are referring to orthodontics. During the orthodontic process, there are several important considerations to ensure the effectiveness of the orthodontic process and maintain oral health:

1. **Maintain oral hygiene**: Orthodontic devices can make it more difficult to clean your teeth. Brush your teeth at least twice a day and floss to clean the gaps between your teeth and aligners.
2. **Avoid certain** foods: Hard, sticky, or large pieces of food may damage the aligners. Such as nuts, hard candy, caramel, chewing gum, etc.
3. **Regular check-ups**: Follow your dentist's recommendations for regular follow-up appointments to ensure a smooth correction process.
4. **Use wax sheets**: If the aligners cause discomfort inside the mouth, special wax sheets can be used to cover the sharp edges.
5. **Avoid any actions that may cause damage to the instrument**: such as biting a pen, biting a nail, etc.
6. **Wear a protector**: If you participate in physical activity, especially contact sports, you should wear an oral protector.
7. **Follow your dentist's instructions**: Your dentist may give special instructions, such as wearing rubber bands or other assistive devices.
8. **Patience**: Orthodontics is a lengthy process that requires patience and constant attention.

If you have any doubts or discomfort, you should consult your dentist promptly. Everyone's situation is different, so your dentist will provide personalized advice that works best for you.

1. How can I ensure the effectiveness of clear aligners?

The key to ensuring the effectiveness of clear aligners lies in the following aspects:

1. **Choosing the right clear aligners**: Not all dental problems are suitable for clear aligners. Work with a dental professional to ensure that clear aligners are the best option for your specific situation.
2. **Follow your dentist's instructions**: Clear aligners need to be replaced according to a specific schedule, usually every two weeks. It is important to strictly follow the dentist's instructions and schedule.
3. **Maintain adequate wearing time**: For best results, it is generally recommended to wear clear aligners for 22 hours a day. Remove only when eating and cleaning your teeth.
4. **Good oral hygiene**: Maintaining good oral hygiene is essential while using clear aligners. After each meal, you should brush your teeth and clean your appliances to prevent bacterial buildup and tooth erosion.
5. **Regular follow-up visits**: Visit your dentist regularly for check-ups to ensure that your treatment is going well. Your dentist may need to adjust your treatment plan to accommodate your progress.
6. **Adherence to the treatment plan**: The entire treatment process can take anywhere from a few months to several years. It is important to be patient and stick to the whole orthodontic process.
7. **Wear retainers**: After the orthodontic treatment is over, retainers are usually needed to maintain the new position of the teeth. Follow your dentist's advice and wear retainers to prevent the tooth from returning to its original position.

Remember, everyone's situation is unique, so it's crucial to work closely with your dentist and follow a personalized treatment plan.

1. What is the process of lingual correction?

Lingual orthodontics, also known as lingual invisible orthodontics, is a method of orthodontics in which aligners are mounted on the lingual side of the tooth so that they are barely visible in appearance. The basic process of lingual correction usually consists of the following steps:

1. **Initial consultation**: First, the patient needs to have an initial consultation. At this stage, the orthodontist will perform a comprehensive examination of the patient's oral health, including the alignment of the teeth, the occlusal relationship, etc.
2. **Develop a treatment plan**: Based on the results of the initial consultation, the doctor will develop a personalized treatment plan. This usually includes x-rays of the teeth, oral models, and a detailed treatment plan.
3. **Impressions and custom aligners**: The doctor takes precise measurements of the patient's teeth and takes impressions. These models will be used to customize lingual aligners that are specifically tailored to the patient's teeth.
4. **Fitting aligners**: Once the custom-made aligners are ready, the doctor will install them on the lingual side of the patient's teeth.
5. **Regular follow-up visits**: After the aligners are fitted, the patient needs to return for regular visits so that the doctor can adjust the aligners, monitor the progress of treatment, and make necessary adjustments.
6. **Post-treatment**: As the treatment progresses, the teeth gradually move to their intended position. The entire correction process can take anywhere from a few months to several years, depending on the complexity of the case.
7. **Removal of aligners**: The aligners are removed once the teeth have been moved to the desired position.
8. **Wear retainers**: After the treatment is complete, retainers are usually needed to maintain the new position of the teeth and prevent them from returning to their original positions.

It is important to note that the specific process of lingual correction may vary depending on the individual situation and the clinic chosen. Before choosing to undergo lingual correction, it is advisable to consult a professional orthodontist for more personalized and professional advice.

1. Are there any complications of orthodontic treatment?

Yes, there may be some complications from orthodontic treatment. Orthodontic treatment is mainly through the use of braces, retainers, or other appliances to correct the position of the teeth and jaws. While this treatment is safe and effective in many cases, it can also come with some risks and complications, including:

1. **Risk of tooth loss**: In rare cases, orthodontic treatment may result in tooth root resorption, which can make the tooth unstable or even fall out.
2. **Gum problems**: Braces and other orthodontic devices can make oral hygiene more difficult, increasing the risk of gingivitis and periodontal disease.
3. **Tooth decay**: If good oral hygiene is not maintained during treatment, food debris and bacteria may build up around braces or other appliances, increasing the risk of tooth decay.
4. **Discomfort and pain**: Repositioning your teeth may cause temporary discomfort or pain.
5. **Effect on occlusion**: Incorrect treatment can lead to occlusal problems, such as jaw joint disorders.
6. **Tooth re-movement**: After treatment, if you don't wear the retainer as directed, the tooth may return to its previous position.

It is important to note that the risk of complications varies from individual to individual and can significantly reduce these risks by following the orthodontist's guidance closely. If you are considering orthodontic treatment, it is advisable to discuss the possible risks and expected results in detail with a professional orthodontist.

**Querying GPT-4 with Standard Method (Multiple-Choice Questions)**

**Prosthodontics**

**Restoration of Tooth Defects**

1.2010U4A1059 gold porcelain crown can be used for ( )

A. Anterior teeth misaligned

B. Conical teeth

C. Fixed bridge retain

D. Tetracycline teeth

E. All of the above

2.2004U4A2082 A patient, the upper right 4 near the mid-cheek large area of mercury filling, dead pulp tooth, the patient has high aesthetic requirements, the best restoration design is ( )

A. Gold alloy full crown

B. Resin inlays

C. Metal porcelain crown

D. Plastic full crown

E. 3/4 crown

3.2010U4A1060 The role of opaque porcelain, except ( )

A. Increase the brightness of the porcelain layer

B. Cover up metallic colors

C. Chemical bonding of gold porcelain

D. Forming the base color of the gold porcelain crown

E. All of the above

4.2013U4A1005 The performance requirements for porcelain alloys are incorrect ( )

A. The lowest elastic mold

B. Good mechanical strength

C. Good casting performance

D. Small shrinkage deformation

E. Good wettability

5.2011U4A1026 The requirements for porcelain alloy are incorrect ( )

A. The modulus of elasticity is small

B. Good wettability

C. Small shrinkage deformation

D. Good casting performance

E. Not easy to deform and wear

6.2003U4A1012 The most important binding force in gold-porcelain bonding is ( )

A. Mechanical mating

B. van der Waals

C. Inverted retention

D. Chemical binding

E. Pressure bonding

7.2010U4A1061 For the reinforcement of gold alloy porcelain gold bonding, the method that can be adopted, except ( )

A. Sandblasting

B. Pre-oxidation

C. Ultrasonic cleaning

D. Degassing

E. Electrolytic etching

8.2010U4B1140 The cut end of the anterior gold porcelain crown should be at least ground ( )

A. 1mm

B. 1.5mm

C. 3.5m

D. 2.5mm

E. 3mm

9.2010U4A1062 The thickness of the lip porcelain layer in the anterior tooth gold porcelain crown is generally ( )

A. 0.5mm

B. 1mm

C. 2mm

D. 2.5mm

E. 3mm

10.2003U4A1013 The gingival margin of the lip of the gold porcelain crown is generally ( )

A. 0.5mm shoulder

B. 1mm shoulder

C. 1.2mm shoulder unit

D. 1.5mm shoulder

E. None of the above

11.2003U4A1014 Which of the following descriptions of the base crown of gold porcelain crown is wrong ( )

A. Good fit with the preparation

B. Support porcelain layer

C. The junction of gold porcelain is blade-shaped

D. Avoid the occlusal zone at the gold-porcelain junction

E. The lip surface is a porcelain layer with a gap of 0.85~1.2mm

12.2007U4A1005 PFM full crown shoulder width is generally ( )

A. 0.5mm

B. 1.0mm

C. 1.5mm

D. 2mm

E. 2.5mm

13.2000U4A1005 The production of metal porcelain crowns, the wrong statement is ( )

A. Full crown tongue side and neck margin are all metal

B. The gold-porcelain junction should avoid the occlusal functional area

C. The gold-porcelain junction is beveled and overlapped

D. The thickness of the crown on the bottom layer of the porcelain covering area is at least 0.5mm

E. The porcelain layer space in the porcelain coverage area shall not exceed 2mm

14.2004U4A2083 A patient, after the restoration of the metal porcelain crown of the right upper central incisor. Inspection: the metal porcelain crown is good in color and shape, not loose, the probe on the edge of the neck can be probed, the dental floss at the adjacent point can barely pass, and the lower right 1 insect end bites on the porcelain surface of the metal porcelain crown when the median tooth is occluded, and this crown is ( )

A. Qualified restorations

B. The adjacency is too tight

C. Adjacent over-loose

D. Improper design of the gold-porcelain bonding area

E. Misfit of the edges

15.2003U4A1015 The least likely cause of porcelain layer damage after fixed denture restoration is ( )

A. Early occlusal contact

B. Prepare 1 mm space on the lingual side of the anterior teeth

C. The metal material does not match the porcelain powder

D. Contamination of the surface of the metal substrate during production

E. 0.5mm space left on the occlusal surface of the fixed bridge base bridge

16.2011U4A1027 Excessive axonal process of the restoration can cause ( )

A. Gingival hyperplasia

B. Gum recession

C. Gingival epithelium attachment disruption

D. Gingivitis

E. Food impaction

17.2011U4A1028 Tooth defect restoration as a treatment device for ( )

A. Prevent the further development of dental lesions

B. Restoration of normal physiological functions

C. Prevent the occurrence of dental and periodontal support tissue lesions

D. Maintain the health of the oral and maxillary system and coordinate with all departments

E. All of the above are their functions

18.2011U4A1029 The requirements for tooth preparation are ( )

A. Remove the diseased tissue and stop the lesion from developing

B. Eliminate the shaft wall indentation and obtain a good positioning channel

C. Open up the space occupied by the restoration, and ensure that the restoration has a certain strength, thickness and beauty

D. Prepare good retention and resistance shapes, abrasion of long teeth, misaligned teeth, abnormal jaw teeth and adjacent teeth, and make appropriate preventive extensions

E. All of the above requirements should be met

19.2011U4A1030 Significance of close contact between the adjacent surface of the crown restoration and the adjacent tooth does not include ( )

A. Prevent food impaction

B. Maintain the stability of the tooth position and arch shape

C. Maintain mutual support with adjacent teeth and disperse the tooth resultant force

D. Helps each tooth maintain its own physiological movement while chewing

E. May increase chewing power

20.2010U4A1063 Which of the following statements about pulp protection in dental preparation is false ( )

A. Tooth preparation is done in stages

B. Light pressure grinding

C. Minimize teeth grinding

D. Prepare for a short time break, short time grinding

E. Water mist cooling

21.2011U4A1031 Which of the following methods can be taken in order to reduce the damage to the pulp when preparing the abutment tooth ( )

A. Water mist cooling

B. Intermittent cutting

C. Short time cutting

D. Light pressure grinding

E. All of the above are true

22.2008U4A1001 Restoration of the affected tooth, in order to prevent the occurrence of secondary caries, to do ( )

A. The prosthesis should correctly restore the anatomical morphology of the affected tooth

B. The restoration should be able to restore the function of the affected tooth

C. The restoration is as thick as possible to prevent bacterial intrusion

D. The edge line of the restoration should be located in the self-cleaning zone

E. All of the above

23.2009U4A1005 The disadvantages of the superior edge of the whole crown gingiva are ( )

A. Predisposition to secondary caries

B. Easy to cause plaque adhesion

C. The edges are not easy to seal

D. Unsightly in the anterior area

E. Easy to form shoulders

24.2010U4A1064 Factors that do not need to be considered when determining the position of the gingival margin of a fixed restoration are ( )

A. Tooth resultant force

B. Periodontal health status

C. Oral hygiene status

D. Aesthetics

E. Age

25.2010U4B1141 Usually the best choice for the gingival edge of the front teeth metal porcelain crown lip is ( )

A. Supragingival concave edge

B. Subgingival bladed edge

C. Supragingival shoulder margin

D. Subgingival concave edge

E. Gingival margin

26.2010U4B1142 Usually the best choice for posterior cast metal full crown gingival margin is ( )

A. Supragingival concave edge

B. Infragingival shoulder margin

C. Supragingival shoulder margin

D. Subgingival concave edge

E. Gingival margin

27.2010U4A1065 The gingival margin of the crown and the tongue of the gold porcelain is best ( )

A. 1mm shoulder

B. 0.5mm concave

C. Bladed

D. Chiseled

E. None of the above

28.2010U4B1143 The worst of the following edge types is ( )

A. Bladed

B. 90° shoulder

C. 90° shoulder with bevel

D. Concave

E. 135° shoulder table

29.2010U4B1144 The worst fit of the following edge types is ( )

A. Bladed

B. 90° shoulder

C. 90° shoulder with bevel

D. Concave

E. 135° shoulder table

30.2010U4A1066 Factors unrelated to the morphological design of the occlusal surface of the whole crown are ( )

A. Retention factors

B. Factors of Resistance

C. Periodontal status

D. Conjunct surface morphology

E. Whether the contralateral crown is intact

31.2010U4A1067 Which of the following is not a measure to increase the resistance of the restoration in the restoration of tooth defects is ( )

A. Use good materials

B. Avoid stress concentrations

C. Increase cusp inclination

D. Avoid casting defects

E. The gold porcelain articulation area of the gold porcelain crown is located away from the occlusal contact point

32.2011U4A1032 Which is wrong to increase the resistance of the prosthetic body ( )

A. Reduce the volume and thickness of the restoration so that more tooth tissue is preserved

B. Reasonably control the shape of the restoration, and avoid sharp, thin and sharp structural forms

C. According to the conditions and design requirements of the affected tooth, select the restoration materials with excellent physical and chemical properties

D. Ensure the quality of the restoration

E. Control the occlusal surface morphology and force direction

33.2011U4A1033 All-ceramic crown belongs to ( )

A. Metal full crown

B. Non-metallic full crown

C. Mixed full crown

D. Nuclear crown

E. Pile crown

34.2013U4A1006 The advantages of porcelain crown do not include which of the following ( )

A. The color is stable and natural

B. Low thermal conductivity

C. Good biocompatibility

D. Non-conductive

E. Low brittleness and high strength

35.2011U4A1034 The situation that should not be made porcelain full crown is ( )

A. Large anterior tooth proximal defect or multiple crown defects

B. Anterior teeth, fluorosis, discolored teeth, tetracycline-stained teeth, and enamel hypoplasia affect the appearance

C. Anterior teeth that are aesthetically pleasing due to dysplasia or dysplasia

D. Defects in primary teeth and juvenile permanent teeth with pulp affecting aesthetics

E. Misaligned and twisted teeth are not suitable for orthodontic treatment

36.2011U4A1035 The indication for casting the crown is ( )

A. Retainers for fixed dentures

B. Modify the shape of the tooth and restore the occlusion

C. Severe defect of the posterior tooth

D. Severe dentin hypersensitivity with tooth defect

E. All of the above

37.2006U4A1002 The abutment tooth morphology is normal, and the retainer with the largest retention force is ( )

A. Inlays

B. Full crown

C. Intrarhizous retain

D. Partial crown

E. Pile core crown

38.2000U4A2082 female, 40 years old, longitudinal fissure of the right maxillary first molar with pulp involvement, short clinical crown, tight occlusion, root canal treatment completed. The optimal prosthetic design for this case is ( )

A. Hammer the crown

B. Casting full crowns

C. Orthodontic inlays

D. Porcelain crown

E. Inlays

39.2010U4A1068 Which of the following is not an indication for casting a full crown ( )

A. Retainers for fixed dentures

B. Modify the shape of the tooth

C. Restore the occlusion

D. Dentin hypersensitivity

E. Stump

40.2011U4A1036 When the cast metal full crown tooth is prepared, the occlusal surface gap should be prepared ( )

A. 0.5mm

B. 0.8mm

C. 1.0mm

D. 2.0mm

E. None of the above

41.2011U4A1037 The occlusal surface clearance provided by the preparation of casting full crown teeth is generally ( )

A. 1～2mm

B. 0.8～1.5mm

C. 2.5mm

D. 1．5mm

E. 2.5～3mm

42.2013U4A1007 When preparing the inlay tooth, the wrong way to do it is ( )

A. Remove the decay of the lesion

B. The maximum circumference of the axial surface is lowered to the gingival margin

C. Provide good retention and resistance shapes

D. Proper abrasion of abnormal counter-jaw teeth

E. Preventive expansion

43.2011U4A1038 The casting temperature of the cast gold alloy is ( )

A. Above 1100°C

B. 850～1000℃

C. 500～1000℃

D. 850～300℃

E. 500～300℃

44.2011U4A1039 Which of the following statements about full crown retention is false ( )

A. The full crown retention force mainly comes from friction and adhesion

B. Friction is the most important retention force

C. The amount of friction is related to the fit of the restoration to the preparation

D. The occlusal surface morphology of the full crown is related to retention

E. Buccal-lingual diameter of the preparation is independent of retention

45.2010U4A1069 Which of the following measures is not conducive to increasing the friction of artificial crowns ( )

A. Increase the polymerization angle when preparing teeth

B. The contact surface is appropriately rough

C. The artificial crown is tightly integrated with the affected tooth

D. Try to keep the axial planes of the crown as parallel as possible

E. Point angles and line angles should be clear

46.2014U4A1023 Which is not a factor affecting the total crown retention ( )

A. Crown height

B. Crown diameter

C. Type of alloy used

D. Defects

E. Dental resultancy

47.2010U4B1145 The depth of the nail hole retention form is generally ( )

A. 1mm

B. 1.5mm

C. 3.5mm

D. 2.5mm

E. 3mm

48.2010U4A1070 Which of the following statements about the nail hole retention is wrong ( )

A. The amount of the nailing force depends mainly on the depth of the nail hole

B. The diameter of the retaining nail is about 1mm

C. Retaining nails can be divided into cemented type, threaded type, and wedge-in type

D. Wedge-in retention force is the best

E. The nail hole should be located in the solid tooth nature

49.2011U4A1040 The retainer of the tooth preparation of the restoration does not include ( )

A. Box retention

B. Dovetail retention

C. Inverted retention

D. Nail hole retention

E. Groove retention

50.2010U4A1071 Which of the following statements about the groove is false ( )

A. The depth is generally 1mm

B. Resistant to horizontal dislocation and occlusal dislocation

C. Consistent with neck 1/3 orientation

D. Reinforcement of restorations

E. Align with the direction of the inauguration path

51.2010U4A1072 Which of the following statements about the dovetail retainer is false ( )

A. Can increase the retention force of the restoration

B. Prevents horizontal dislocation of the restoration

C. The width of the Hatomi Gorge is about 1/2 of the width of the occlusal surface

D. The dovetail gorge should be located between the triangular crests of the occlusal surface

E. The preparation of the dove tail has an effect on the resistance of the preparation

52.2004U4A1036 The inclination of the slope of the cave edge is ( )

A. 30°

B. 45°

C. 60°

D. 70°

E. 25°

53.2013U4A1008 The following are not double-sided inlays ( )

A. Distal occlusal inlay

B. Buccal occlusal inlay

C. Medial occlusionDistal inlay

D. Tongue-occlusal inlays

E. Median occlusal inlay

54.2007U4A1006 The material that should not be used for inlay repair is ( )

A. Composite resins

B. Self-coagulating resins

C. Porcelain

D. Cast ceramics

E. Metals

55.2014U4A1024 Comparison with amalgam filling: The advantages of casting inlays are ( )

A. Excellent mechanical properties

B. Good retention

C. Short marginal lines

D. Less tooth incision

E. Easy to make

56.2010U4A1073 Indications for inlays, except ( )

A. Adolescent permanent teeth

B. Severe wear of the occlusal surface requires occlusal reconstruction

C. Occlusal brackets should be placed on the occlusal cavity

D. Tooth defect with broken proximal contact

E. None of the above

57.2000U4A1006 The purpose of preparing the inlay edge bevel does not include ( )

A. Increase the edge fit of the inlay

B. Increase the strength of the inlay

C. Reduction of micro-leakage

D. Prevent enamel breakage

E. Increase the closure of the inlay and the edges

58.2010U4A1076 Which of the following requirements for the 3/4 crown cut bevel of the anterior teeth is incorrect ( )

A. At a 90° angle to the long axis of the tooth

B. Prepare a gap of 0.7mm or more

C. The fangs should form two bevels, the mesiodistal and distal

D. The upper anterior teeth are obliquely from the labial side to the lingual side

E. The lower anterior teeth are obliquely from the lingual side to the labial side

59.2010U4A1077 The main function of the 3/4 crown sulcus of the anterior teeth is ( )

A. Prevent occlusion dislocation

B. Prevent lingual dislocation

C. Prevent mesiodistal and distal dislocations

D. Prevent labial dislocation

E. Prevent gingival displacement

60.2014U4A1025 Which of the following requirements for the front teeth 3/4 crown cut bevel is wrong ( )

A. At a 90-degree angle to the long axis of the tooth

B. Prepare a gap of 0.7mm or more

C. The fangs should form two bevels, the mesiodistal and distal

D. The cutting end groove can be prepared in the cutting bevel

E. Lower anterior teeth are prone to metal exposure

61.2010U4A1078 3/4 crown disadvantage ( )

A. Easy to get in place

B. It cannot be used as a fixed bridge retainer

C. Molars

D. Long outline line, not conducive to caries prevention

E. None of the above

62.2010U4A1079 The direction of the 3/4 crown sulcus of the upper anterior teeth is ( )

A. Parallel to the long axis of the tooth

B. Parallel to the labial cut 2/3

C. Parallel 1/3 to the labial face

D. Parallel to the labial gingiva 1/3

E. Parallel to the lingual carina

63.2010U4A1080 The direction of the 3/4 crown sulcus of the upper posterior teeth is ( )

A. Parallel to the buccal occlusion 2/3

B. Parallel to buccal gingiva 1/3

C. Parallel to the shaft wall

D. Parallel to the lateral wall of the tongue

E. None of the above

64.2010U4A1081 The position of the 3/4 crown sulcus of the upper posterior teeth is ( )

A. Adjacent cheek 1/3

B. Junction of the adjacent cheek 1/3 and the middle 1/3

C. 1/3 of the adjacent surfaces

D. The 1/3 of the adjacent surface is bordered by the 1/3 of the tongue

E. 1/3 of the adjacent tongue

65.2000U4A1007 Artificial crown fully in place sign, not included ( )

A. The bite is basically appropriate

B. No warping

C. Edge sealing

D. The edge reaches the design position

E. Proper tightness of the contact points

66.2014U4A2080 Male, 20 years old. Traumatic defect of upper anterior teeth for 7 days. No symptoms. Oral examination: right upper 1 mesial incision angle defect, gingival diameter greater than 2/3 of the crown, no penetration of the pulp cavity, normal temperature test, percussion (-), no loosening. X-ray shows that there is no root fold, and the most appropriate treatment is ( )

A. Nail-retained amalgam repair

B. Nail-retained glass ion repair

C. Nail-retained composite resin restoration

D. Nail-retained zinc phosphate cementing powder repair

E. Endodontic treatment followed by porcelain crown restoration

67.2003U4A1016 Which of the following statements about the possible causes of spontaneous pain after full crown bonding is false ( )

A. Pulpitis

B. Metallic microcurrent stimulation

C. Apical periodontitis

D. Gingivitis

E. All of the above is wrong

68.2003U4A2078 Spontaneous pain on the second day after cementation of mandibular bicuspids, pulp, and full crown restoration, the most likely cause is ( )

A. Pulpitis

B. Occlusal trauma

C. Apical periodontitis

D. Pulp congestion

E. None of the above

69.2003U4A1017 Gingivitis after full crown bonding, its possible causes, except ( )

A. Poor axial wall protrusion

B. The crown margin is too long

C. Crown margins are not sealed

D. Cementant residue in the sulcus

E. Early occlusal contact

70.2011U4A1041 Which of the following is not included in the causes of gingivitis after full crown repair ( )

A. Food impaction

B. Occlusal imbalance

C. The edges are too long

D. Misfitting edges

E. Poor finish with rough edges

71.2003U4AN123 47-year-old male. The maxillary anterior teeth have been fixed and restored for 3 years, and now they are loose and have painful bite. Oral examination: the upper left 2 is missing, the upper left 123 porcelain fixed bridge is repaired, and the upper left 13 is the porcelain full crown retainer, and the edges are not sealed. Deep caries can be detected on the distal proximal surfaces of the upper left 1 and upper left 3, and the upper left 3 percussion (++) shows red and swollen gingiva without periodontal pockets. The remaining teeth are healthy and the bite is normal. The reason for the loosening of the original fixed prosthesis is ( )

A. Breakage of the original fixed restoration

B. The edges of the original fixed prosthesis are not sealed

C. Gingivitis

D. Periodontitis

E. Poor abutment preparation

72.2003U4A1018 Short-term occlusal pain after full crown bonding, the main reason is ( )

A. Pulp hyperemia

B. Loose crown

C. Periodontitis

D. Traumatic occlusion

E. None of the above

73.2011U4A1042 Causes of Bond Repair Failure Do Not include ( )

A. Removal of the marrow protection material from the cave wall

B. The adhesive is too thick

C. The tooth surface is not thoroughly cleaned

D. Bonded restorations were moved before curing

E. Poor moisture insulation

74.2004U4A2084 patient, 2 months after the repair of the upper left 7 casting crown, loosened and fell off, examination: the preparation was good, and the occlusal examination revealed that there was a significant early contact point at the tip of the mesial tongue. The correct treatment is ( )

A. Align the jaw cusp

B. Alignment of the jaw early contact point

C. Use a better quality adhesive for bonding

D. Adjust the early contact points on the crown and re-glue after polishing

E. Redo the full crown

75.2011U4A1043 The most ideal restoration for the treatment of stumps is ( )

A. Inlays

B. Pile crown

C. Metal crown

D. Porcelain crown

E. 3/4 crown

76.2010U4A1082 According to the degree of tooth defect from small to large, the largest degree of tooth defect should be selected ( )

A. Full crown

B. Inlays

C. Pile crown

D. Partial crown

E. Onlays

77.2013U4A1009 Eligible for the indications for pile crown are ( )

A. Lateral puncture of the root canal wall

B. Root canal treatment has been performed, and the fistula orifice is not closed

C. Crown stump for fixed denture abutments

D. The anterior teeth are obliquely folded up to 1/3 of the roots

E. The root canal is slightly curved

78.2004U4A2085 A patient, 3/4 of the crown of the right upper central incisor is defective, no percussion pain, no loosening, no redness and swelling of the gums, X-ray shows that the tooth has been treated with a perfect root canal, and there is no shadow at the root tip, the most suitable treatment plan is ( )

A. Inlays

B. 3/4 crown

C. Open crown

D. Pile core + full crown

E. Full crown

79.2004U4A2086 A patient with 6 large areas of amalgam filling in the upper right requires repair treatment. Examination: a large area of mercury filling on the occlusal surface, less remaining hard tissue in the crown, only the thin wall of the buccal tongue remains, no loosening, no percussion pain, and the root canal treatment has been perfected. Best treatment plan ( )

A. Inlays

B. Onlays

C. Cast metal full crown

D. Porcelain full crown

E. Pile nucleus + full crown

80.2004U4A1037 Which item is wrong ( )

A. Preserve as much residual crown tissue as possible to increase the length of the crown pile

B. The root canal is prepared into an oval shape to reduce the taper of the root canal wall

C. Use the length of the root canal as much as possible, and the crown pile can reach the apex if needed

D. Shoulder preparation at the root and neck to increase the stability of the crown

E. Use cast crown piles to increase the tightness of the crown piles and the root canal wall

81.2004U4A2087 A patient, a large part of the crown of the anterior tooth is defected, and the length of the root canal preparation should reach the root length ( )

A. 1/5

B. 1/4

C. 2/5

D. 2/3

E. 4/5

82.2010U4A1083 The following method of increasing the crown pile retention, the incorrect is ( )

A. Prepare a small shoulder table at the root canal orifice

B. Increase the tightness of the pile and the pipe wall

C. Choose excellent binders

D. Preserve the residual crown hard tissue as much as possible

E. Remove the residual crown hard tissue as much as possible

83.2010U4A1084 Enhancement of pile core retention method, except ( )

A. Preserve the residual crown hard tissue as much as possible

B. Make use of root canal length as much as possible

C. Increase the tightness of the pile and the pipe wall

D. The root canal is prepared into a cylindrical shape

E. Proper use of binders

84.2010U4A1085 Which of the following statements about crown retention is false ( )

A. Adhesion is the most important retention force

B. The pile should be tightly sealed with the root canal wall

C. The longer the pile, the better the retention

D. The pile diameter is related to the retention

E. Pile morphology affects retention

85.2010U4A1086 Which of the following statements about the pile crown is false ( )

A. The longer the pile, the better the retention

B. The retention force depends on the friction and adhesion force

C. After the pile crown is repaired, the occurrence of apical disease is increased

D. Perfect root filling must be performed before repair

E. Retainer that can be used as a fixed bridge

86.2010U4A1087 In the repair of pile core crown, the error handling of the repaired residual crown is ( )

A. Cut along the top line of the papillae

B. Removal of decay

C. Remove the thin walls

D. Removal of baseless glaze

E. Preserve the residual crown hard tissue as much as possible

87.2010U4A1088 The following description of the shape of the pile crown, the correct one is ( )

A. The cross-section is circular

B. The transverse diameter is 1/2 of the root diameter

C. Seal with the root canal wall

D. Consistent with root length

E. Cylindrical from root canal mouth to root tip

88.2010U4A1089 The following incorrect operation in the preparation of the pile crown root canal is ( )

A. Look at the dental film first

B. Determine the pile length

C. Remove the root charge with a round drill

D. Use a crack drill to remove the root filling and trim the pipe wall for 1 time

E. Correct the drilling direction at any time when drilling

89.2011U4A1044 Temporary crown is not intended for ( )

A. Avoid re-irritation of the pulp

B. Maintain the tooth position of the affected tooth

C. Avoid wear on the occlusal surface

D. Maintain the mesial and distal space

E. Facilitation of the wearing of crowns

90.2000U4A2083 Male, 40 years old, dead pulp tooth of right lower first molar, restored with PFM full crown after root canal treatment, what kind of treatment is still needed before the full crown is first worn after tooth preparation and impression taking?

A. No processing is required

B. Temporary protective repair with a plastic crown

C. Protective repair with a metal crown

D. Make removable dentures to maintain clearance

E. Make a gap holder

91.2004U4A2088 Patient, 6 dead pulp teeth in the lower right, restored with a metal porcelain crown after root canal treatment, between the preparation of the tooth and the first wearing of the metal porcelain crown, ( )

A. Protective repair with a metal crown

B. Protective repair with a plastic crown

C. Make a gap protector

D. No Processing

E. Protect your gums with a gum protector

92.2003U4AN131 patient, a 30-year-old female, with 7 residual crowns in the upper left, has undergone root canal treatment and is now planned to be protected by crown repair. The most common material for the top 7 temporary crown on the left is self-coagulation resin. The cements commonly used for bonding temporary crowns are ( )

A. Zinc polycarboxylate cement

B. Zinc phosphate cement

C. Vitreous ionic cement

D. Calcium hydroxide cement

E. Zinc oxide cement

93.2003U4A1019 The most common causes of tooth defects are ( )

A. Caries disease

B. Trauma

C. Abrasion

D. Wedge defect

E. Developmental malformations

94.2011U4A1045 Definition of tooth defect ( )

A. Various tooth tissues with varying degrees of texture damage or abnormality

B. Damage or abnormality of various tooth tissues with varying degrees of physiological anatomy

C. Damage or abnormality of various tooth tissues with varying degrees of texture and physiological anatomical shape

D. Damage or abnormality of various tooth hard tissues with varying degrees of texture and physiological anatomy

E. Damage or abnormality of the soft tissues of the tooth to varying degrees

95.2010U4A1090 Which of the following factors is not related to the cause of tooth defect ( )

A. Wedge defect

B. Severe abrasion

C. Periodontal disease

D. Dental trauma

E. Caries disease

96.2010U4A1091 Which of the following causes of tooth defects is wrong ( )

A. Acid erosion

B. Plaque enamel

C. Tetracycline teeth

D. Night grinding

E. Wear occlusal pads

**Fixed partial denture restoration of dentition defects**

1.1999U4A1009 Which of the following is the most important support base for fixed bridges ( )

A. Periodontal ligament

B. Alveolar bone

C. Gums

D. Binding epithelium

E. Mucous membranes

2.2011U4A1048 Among the following oral conditions, fixed restoration can be used ( )

A. Absence of the distal free end of the dentition

B. The number of tooth missing teeth is large and the gap span is large

C. Anterior region with severe jaw defect

D. Anterior teeth are missing, and the abutments on both sides are stable

E. All of the above conditions are suitable for fixed restoration

3.2014U4A1026 Which of the following is not an indication for fixed bridges ( )

A. Single anterior tooth is missing

B. The occlusal relationship is normal

C. Periodontal tissue health of abutments

D. Anterior tooth loss with massive bone defects

E. Orthomaxillary teeth are normal

4.2000U4A1009 Need to consider increasing the number of abutment teeth for the purpose of ( )

A. The abutment tooth is a single tooth

B. Slight tilt of the abutment

C. Abutment periodontal ligament widening

D. Abutments without countermaxillary function

E. Alveolar bone resorption of more than 1/3

5.2008U4A1002 as the abutment of the fixed bridge, must be ( )

A. Pulp tooth

B. The tooth tissue is healthy and intact

C. Periodontal tissue health

D. The abutment teeth are aligned normally without inclination

E. The abutment teeth should not be bitten too tightly

6.2008U4A1003 Which item is the least important when choosing a fixed abutment tooth ( )

A. Ability to retain abutments

B. Common alignment of the abutments

C. The abutment tooth must be a pulp tooth

D. Periodontal tissue health of the abutments

E. Abutment support capacity

7.2011U4A2090 Fixed dentures can only be made if the alveolar ridge in the missing area is well healed, and the fixed bridge should be made after tooth extraction ( )

A. Half Moon

B. January

C. March

D. June

E. 1 year

8.2014U4A1027 The best time for fixed bridge repair after tooth extraction is ( )

A. 1 month

B. 2 months

C. 3 months

D. 4 months

E. 6 months

9.2010U4A1094 What kind of auxiliary examination should be done first if there is occlusal pain after a period of fixed bridge repair?

A. Test the pulp viability of the abutment teeth

B. Check tooth looseness

C. Take an X-ray

D. Inspect the right teeth

E. CT scan of the brain

10.2007U4A1007 Persistent pain in the affected tooth after cementing the pulp restoration, most likely ( )

A. Has developed pulpitis

B. Presence of gingivitis or periodontitis

C. Has developed apical periodontitis

D. Dentin hypersensitivity

E. Has secondary caries but has not developed pulpitis

11.2013U4A2079 male, 30 years old. The right lower 6 metal crown fell off after 1 week of cementation, and the bite often had instantaneous pain. Oral examination showed that the patient had a tight bite, a short crown, an amalgam filling on the opposite surface of the jaw, and a complete crown that had fallen off. The most likely cause of momentary pain in the bite is ( )

A. Pulpitis

B. Apical periodontitis

C. Metal microcurrent stimulation

D. Periodontitis

E. Gingival marginitis

12.2011U4A1049 The advantages of the fixed bridge do not include ( )

A. Stable, retained, well supported, able to fully restore part of the chewing function lost due to missing teeth

B. Similar in size and shape to the tooth of the same name in the original dentition, there is no foreign body sensation, and it does not affect the functional activity of the tongue

C. There are many tooth tissues to be removed, and the scope of application is small

D. High aesthetics

E. The positive force of the teeth is shared by the abutment teeth

13.2011U4A1050 The selection of fixed abutment conditions is not important ( )

A. Abutment support capacity

B. Ability to retain abutments

C. Common alignment of the abutments

D. Periodontal tissue health of the abutments

E. The abutment tooth must be a living pulp

14.2010U4A1095 When using fixed bridge restoration, the influencing factors to determine the number of abutment teeth are ( )

A. Root condition of adjacent teeth

B. Alveolar bone condition of adjacent teeth

C. Periodontal ligament condition of adjacent teeth

D. Occlusion

E. All of the above

15.2011U4A1051 When selecting a fixed abutment, the minimum clinical crown-root ratio is ( )

A. 1:1

B. 1:2

C. 2:3

D. 3:2

E. 3:4

16.2010U4A1096 The conditions that the retainer should have are ( )

A. The retained body should have a good retained shape and resistance shape

B. The retainers should be able to obtain the common alignment channels required to secure the bridge

C. The material used in the retainer should achieve a certain mechanical strength

D. The retainer should be able to protect the tooth tissue

E. All of the above

17. 2011U4A1052 Which retainer design is wrong ( )

A. Inlays have the worst retention effect

B. The longer the span of the bridge, the more curved, and the greater the tooth force, the smaller the retention force of the retaining body

C. The retention force of the retained body at both ends of the abutment teeth should be basically equal

D. There must be a common path of inposition between the retainers

E. Abutment cusp fracture should be prevented

18.2014U4A1028 The following statement about the double-ended fixed bridge retainer is wrong ( )

A. There must be a common way to be in place

B. The retention force of the retainer at both ends should be substantially equal

C. The retention force of the retainer should be adapted to the size of the tooth resultant force

D. The co-alignment tract should be parallel to the long axis of the tooth

E. The retention force of the retainer should be adapted to the span of the bridge

19.2011U4A1053 The occlusal surface of the bridge body should be made ( )

A. The limbic crest morphology should be restored correctly

B. The occlusal surface of the bridge body should form the buccal and lingual grooves

C. A certain abduction gap and adjacent gap should be formed between the bridge and the retained body

D. Occlusal surface: Functional, cusp contact with countermaxillary teeth should be even

E. All of the above should be done

20.2003U4A1020 There is at least 3mm gap between the gingival surface and the mucosa of the suspended bridge, mainly because ( )

A. It is easy for food to pass through and has a self-cleaning effect

B. Does not irritate and compress gum tissue

C. Simple to make

D. The patient feels comfortable

E. Convenient food massage on the gums

21.2008U4A1004 The degree of bending of the bridge body is the same as ( ) when the fixed bridge is stressed.

A. The cube of the bridge thickness is inversely proportional

B. The cube of the length of the bridge is proportional

C. Bridge width is inversely proportional

D. The hardness of the bridge is related

E. All of the above

22.2014U4A1029 Under the same conditions, if the thickness of the fixed bridge body is halved, its deflection deformation becomes ( )

A. Doubled the original size

B. Increase to 4 times

C. Increase to a factor of 6

D. Increase to 8 times

E. Increase to a factor of nine

23.2014U4A1030 The following statement about semi-fixed bridges is wrong ( )

A. Tilt the abutments to obtain a common alignment tract

B. Protect the abutment tooth with weak support on the side of the gap

C. Multi-unit fixed bridge with intermediate abutment to protect intermediate abutment

D. The bolt of the movable connector is located on the retainer

E. Movable connectors are generally bolted attachments

24.2003U4AN125 47-year-old male. The maxillary anterior teeth have been fixed and restored for 3 years, and now they are loose and have painful bite. Oral examination: the upper left 2 is missing, the upper left 123 porcelain fixed bridge is repaired, and the upper left 13 is the porcelain full crown retainer, and the edges are not sealed. Deep caries can be detected on the distal proximal surfaces of the upper left 1 and upper left 3, and the upper left 3 percussion (++) shows red and swollen gingiva without periodontal pockets. The remaining teeth are healthy and the bite is normal. The best restoration method for the upper left 13 after correct tooth treatment is ( )

A. Porcelain full-crown fixed bridge restoration

B. Removable denture restoration

C. Cast metal fixed bridge repair

D. Full mucosal support removable denture restoration

E. Retained fixed denture restoration with 3/4 crown

25.2000U4A2084 Female, 55 years old. Due to the loss of 67 in the upper right position, the 4567 fixed denture in the upper right was repaired, and the abutment teeth had bite pain and loosening one year later. The main reasons for this are ( )

A. Unreasonable design

B. Insufficient retention force

C. Low number of abutments

D. End side sinking

E. Early occlusal contact

26.2011U4A1054 Reasons for poor retention of double-ended fixed bridge are ( )

A. Improper design of the retainer and insufficient retention

B. The number of abutments at both ends is unequal

C. When the abutment tooth is prepared, the axial surface polymerization is too small

D. Insufficient bridge strength

E. The retention force of one end of the retainer is slightly higher than that of the other end

27.2011U4A1055 Which is not a component of a fixed denture ( )

A. Retainers

B. Abutments

C. Bridge

D. Connectomes

E. All of the above components are fixed dentures

28.2011U4A1056 Which of the following fixed bridges belongs to special structure fixed bridges ( )

A. Double-ended fixed bridge

B. Single-ended fixed bridge

C. Semi-fixed bridges

D. Composite fixed bridges

E. Implant abutment fixed bridge

29.2010U4A1097 The main advantages of designing a semi-fixed bridge are ( )

A. Stress buffering

B. In order to attain a common path of inauguration

C. Aesthetics

D. Easy to make

E. Inexpensive

30.2011U4A1057 Which of the following statements is wrong about composite fixed bridges ( )

A. Generally includes 4 or more dentals

B. It is more difficult to achieve a common path

C. The abutment teeth are evenly stressed and are not easy to loosen and fall off

D. Composed of a combination of 2 or more simple fixed bridges

31.2007U4A1008 In the narrative about composite fixed bridges, the correct one is ( )

A. Contains four or more dental units

B. Contains more than two abutment teeth

C. Consists of a combination of two or three basic types of fixed bridges

D. The number of abutments is large and scattered, and it is not easy to obtain a common alignment tract

E. All of the above

32.2004U4A1038 The resultant force of the teeth is completely borne by one side of the abutment, which fixed bridge ( )

A. Double-ended fixed bridge

B. Single-ended fixed bridge

C. Semi-fixed bridges

D. Composite fixed bridges

E. Spring Bridge

33.2011U4A1058 The upper right 2 is missing, and the condition for designing a single-ended fixed bridge with the upper right 3 as the abutment is ( )

A. The upper right 2 has a large gap

B. Upper right 3 alveolar bone resorption exceeds 1/3 of the root length

C. The gap in the upper right 2 is small and the force is not large

D. The patient's tooth force is too large

E. The upper right 3 crowns are short

34.2004U4A2089 40-year-old female, 67 missing lower left, 8 lower left not erupting, and the remaining teeth of the oral cavity are healthy. The occlusion is normal. Pre-fixed denture restoration, the correct design is ( )

A. The lower left 45 was used as an abutment for a single-ended fixed denture

B. Single-ended fixed denture restoration for abutment teeth in lower left 345

C. Single-ended fixed denture restoration for the abutment tooth in the lower left 2345

D. Single-ended fixed denture restoration for the abutment tooth in the lower left 5

E. Single-ended fixed denture restoration is not recommended

35.2004U4A2095 Patient, female, 26 years old, lost an anterior tooth due to trauma 2 months ago, and now requires porcelain restoration. Oral examination: the left upper incisor is missing, the space is normal, and the alveolar ridge has no obvious absorption. 1/2 of the crown of the left upper central incisor is defective, the pulp has been exposed, the probe is slightly sensitive, the percussion is negative, and there is no loosening. The crown of the left upper canine is good, negative to percussion, and there is no loosening. There was no abnormality in the remaining teeth. The most suitable treatment options are ( )

A. Pile crown partial dentures

B. Overdentures

C. Pile core and double-ended fixed bridge

D. Intraroot retainer fixed bridge

E. None of the above

36.2004U4A2096 45-year-old male, the lower right 6 is missing, the lower right 7 is stable, the lower right 5 periodontal condition is poor, if it is repaired according to the double-ended fixed bridge, it should be designed as ( )

A. The lower right 5 is best designed with a full crown retainer

B. Lower right 5 is best designed with intracoronal retainers

C. Fixed denture restoration with the lower right 457 as the abutment

D. Reduce the tip slope of the bridge surface

E. Appropriately reduce the buccal-lingual diameter of the pontine body

37.2004U4A1044 The quality standards that should be met by the restorations are ( )

A. Sufficient retention force, correct shape, adjacency and occlusion

B. The edge is close to the affected tooth

C. The bite force is coordinated with the periodontal tissues to restore the function of the oral and maxillary system

D. It should be in place smoothly, the color is natural and beautiful, the surface is smooth and clean, the self-cleaning effect is good, and there is no adverse stimulation

E. All of the above

38.2014U4A1038 The best material used for the gingival surface of the bridge body is ( )

A. Ceramics

B. Medium-melt alloys

C. High-melt alloys

D. Thermoset plastics

E. Self-corositing plastics

39.2010U4A2133 If one end of the double-ended fixed bridge is designed as a 3/4 crown retainer, and the other end is a full crown retainer, which of the following methods should be used when making the wax type of the fixed bridge?

A. Made directly in the mouth

B. Fabrication on a model

C. Make it on the model first, then finish it in the mouth

D. First make it in the mouth and then finish it on the model

E. None of the above is appropriate

**Chapter 5 Removable partial denture restoration of dentition defects**

1.2014U4A1031 The reason for the difficulty in placing a removable partial denture cannot be ( )

A. The ring arm is too tight

B. The snap ring body enters the abutment tooth inverted

C. The base enters the inverted

D. High occlusion of occlusal support

E. Deformation of dentures

2.2011U4A1059 The reason for the deformation of the bracket is the most unlikely ( )

A. The impression material is not of good quality

B. Improper design

C. The casting channel is not set up reasonably

D. Model defects

E. The coefficient of thermal expansion of the high-temperature embedding material is insufficient

3.2006U4A1003 What does not make it difficult to remove partial dentures is ( )

A. The base enters the tissue inverted

B. The snap ring arm is too tight

C. Inposition in the wrong direction

D. The tip of the arm enters the inverted hole too deeply

E. The base does not fit into the mucosal tissue

4.2008U4A1005 Kennedy Type 1 Dentition Defect ( )

A. Unilateral free deletion

B. Bilateral free deletions

C. Non-dissociative absence

D. Absence of space

E. Anterior tooth loss

5.2008U4A1006 According to Kennedy's taxonomy, 8721|1278 deletions belong to ( )

Category I, subcategory I

B. Category II, subcategory I

C. Category II, subcategory II

D. Category III and subcategory II

E. Category IV and subcategory II

6.2008U4A1007 According to the Kennedy taxonomy, 8765278 deletions belong to ( )

Category I, subcategory I

B. Category I and subcategory II

C. Category II, subcategory I

D. Category II, subcategory II

E. Category III, subcategory I

7.2008U4A1008 According to the Kennedy classification of dentition defects, those with 8743211278 defects belong to ( )

A. Category I, subcategory II

B. Category I, subclass I

C. Category II, subcategory I

D. Category IV and subcategory II

E. Category IV

8. 2011U4A1060 Bilateral free end deletion belongs to Kennedy classification ( )

A. Category I

Category II

C. Category III

Category IV

E. Category I. Classification

9.2011U4A1061 Dentition defect Kennedy classification class 1 ( )

A. Unilateral free end is missing

B. Bilateral free end deletion

C. Unilateral non-free end deletion

D. Bilateral non-free end deletions

E. Anterior tooth loss

10.2008U4A1009 According to the Kennedy taxonomy, 8765246 deletions belong to ( )

A. Category I, subcategory II

B. Category I, subcategory III

C. Category II, subcategory II

D. Category II and sub-categories

E. Category III and subcategory

11.2008U4A1010 According to the Kennedy classification of dentition defects, those with 652112346 defects belong to ( )

A. Category III and subcategory II

B. Category III, subcategory I

C. Category II, subcategory II

D. Category IV and subcategory II

E. Category IV

12.2008U4A1011 According to the Kennedy classification of dentition defects, those with 432112 defects belong to ( )

A. Category III and subcategory II

B. Category III, subcategory I

C. Category III

D. Category IV and subcategory II

E. Category IV

13.2004U4A2090 Maxillary Left 1234 Right 123456 Missing Category Which category does Kennedy classification belong to ( )

A. Category III and subcategory II

B. Category III and subcategory III

C. Category III

Category IV, subcategory I

E. Category IV

14.1999U4A2080 A patient has a loss of lower right 6, and the lower right 5 and lower right 7 are healthy, and the gap is normal. The fulcrum line of a removable partial denture can be designed as ( )

A. Slash

B. Straight

C. Horizontal line

D. Longitudinal type

E. Flat type

15.2000U4A2085 Male, 60 years old, wearing dentures for 2 days, felt pain when moving the upper lip downward, and difficulty in removing the dentures. Check: 12 partial dentures can be removed from the upper right 1234567 upper left, 37 single-arm snap rings on the upper left, the snap rings fit with the abutments, the base of the upper anterior arch area is too long, and the resistance to remove the dentures is greater. The rest is unusual. Causes of pain and difficulty in removing dentures are other than ( )

A. The snap ring is too tight

B. The base fits snugly against the tooth surface

C. The base enters the undercut area

D. The denture base area is large

E. The patient has not mastered the method of removing the denture

16.2004U4A1039 Causes of poor retention of removable partial dentures include, in addition to ( )

A. Abutment crowns are small

B. The base does not fit well with the tissue, and the edges are not well sealed

C. The snap ring does not fit the abutment tooth

D. Improper placement of artificial teeth

E. The base cushioning of the undercut area is insufficient

17.2003U4AN142 A patient has missing 5678 left and 678 right mandible, left lower 4 is not loose, no caries, and the alveolar ridge is full. Cast bracket denture lower left 4 lower right 5 distal middle occlusion support, three-arm card retainer, tongue rod large connector. After wearing the denture for 1 week, he complained of base tenderness and abutment bite pain. Intraoral examination found that there was a small ulcer at the base of the tongue frenulum, the mucosa of the left mandibular convex area was red and swollen, the lower left 4 percussion pain (+), the dentures were tightly sealed, and the bite was not high. The cause of ulceration at the base of the tongue-tie is that the tongue shaft is too low. The treatment of left mandibular bulge tenderness is ( )

A. Denture base edge grinding off

B. Buffering at the corresponding point of the tissue surface of the denture base

C. Relining of the denture base tissue surface

D. Adjust the occlusion

E. Adjust the retention force of the lower left 4 snap rings

18.2003U4AN146 A patient has missing 5678 in the right mandible, and the adhesive denture is repaired, with 4 three-arm cards in the lower right and 47 gaps in the lower left. One week after wearing the denture, he complained of tenderness at the free end of the denture and discomfort to chew. Intraoral examination found that all parts of the denture were tightly sealed, the retention and stability were good, there was no high point in the occlusion, the base extension did not affect the soft tissue activity, and there was no obvious tenderness, redness, swelling or ulceration of the missing mucosa. The cause of denture tenderness is ( )

A. Poor clamp ring retention force

B. Kito sinking

C. Pitch of the base

D. The alveolar ridge is overburdened

E. Improper Use

19.2011U4A1062 The oral examination that needs to be carried out before the start of the restoration is ( )

A. External oral examination

B. Examination in the mouth

C. Preparation of model checks

D. X-rays

E. All of the above tests are required

20.2014U4A2081 When designing the gap card to prepare the tooth, it cannot be prepared into a wedge, nor can it destroy the contact point of two adjacent teeth, the reason for this is ( )

A. Make the most of natural gaps

B. Prevent interabutment food impaction

C. Minimal tooth tissue is removed

D. Prevent decay of the abutments

E. Avoid the formation of a wedge force that displaces the abutment

21.2011U4A1063 What kind of impression must be trimmed when taking ( )

A. Anatomical impressions

B. Impressions of tooth-supported dentures

C. Impressions of mucosal support dentures

D. Impressions of hybrid support dentures

E. Individual pallet impressions

22.2004U4A1040 The purpose of taking a functional impression is ( )

A. Accurately reflects the position and morphology of the perioral muscles during functional activity

B. Evenly compress the oral tissues

C. Round the edge of the impression

D. See anatomical landmarks clearly

E. Margins and mucosal transitions are clear

23.2004U4A2091 A patient has 67 upper right maxillary remainder and 67 lower left lower right mandib, which method should be used in order to establish the correct occlusal relationship on the model ( )

A. Recording the occlusal relationship of the remaining teeth by wax biting

B. Record vertical distances with wax embankments

C. Record the median relationship with a wax embankment

D. Record the vertical distance with the median relationship with the wax embankment

E. Determine the vertical distance and median relationship directly with the model occlusion

24.2013U4AN125 When wearing the full denture for the first time, it was found that the lower total denture was warped left and right, and the patient felt pain during afterburner. Possible causes of warping are not included ( )

A. The kito is stretched too long

B. Inaccurate impressions

C. The base is not buffered into the undercut area

D. Kito deformation

E. The base tissue plane corresponding to the hard zone is not buffered

25.2006U4A1004 The purpose of the removable partial denture is that the width of the buccal and lingual diameter of the artificial posterior teeth is smaller than that of the natural tooth ( )

A. Improve chewing efficiency

B. Balance

C. Prevent cheek biting

D. Reduce the load on the support organization

E. Enhanced retention

26.2014U4A1032 According to the direction and degree of abutment tooth inclination, the wire can be divided into three types, the correct one is ( )

A. The wire drawn by the abutment tooth tilted to the gap side is the I. type wire

B. Ι-type wire is close to the occlusal surface on the near gap side of the abutment

C. The wire drawn by the abutment tooth tilted in the opposite direction of the gap is a type II wire

D. Type II wire is far from the occlusal surface on the distal gap side

E. When there is no obvious inverted indentation on the near and distal gap side of the abutment tooth or the abutment tooth is inclined to the mesiodistal and middle of the abutment, the wire drawn is a type III wire

27. 2010U4A1098 I. type observation line of the undercut is mainly in ( )

A. The proximal notch side of the abutment tooth is inverted very smallly

B. Distal notched side of the abutment, the proximal notch side is very small

C. There are large inverted concaves on the near and distal gap sides of the abutments

D. There is no large inverted depression on the near and distal gap sides of the abutment

E. Adjacent faces near the gap

28.2011U4A1064 Type II. The undercut area of the abutment of the lateral line is in ( )

A. Near the gap side

B. Distal gap side

C. Buccal side

D. Lingual

E. The inverted concave area of the near and far gap sides is large

29.2004U4A1041 Which of the following is NOT a component of a removable partial denture ( )

A. Retainers

B. Abutments

C. Artificial teeth

D. Kitto

E. Connectors

30.2004U4A1042 The width and length of the molar casting occlusal support are ( )

A. The width is 1/3 of the buccal tongue diameter and the length is 1/4 of the mesiodistal middle diameter

B. The width is 1/4 of the buccal-tongue diameter and the length is 1/4 of the mesiodistal middle diameter

C. The width is 1/4 of the buccal-tongue diameter and the length is 1/3 of the mesiodistal middle diameter

D. The width is 1/3 of the buccal-tongue diameter and the length is 1/3 of the mesiodistal middle diameter

E. The width is 1/2 of the buccal tongue diameter and the length is 1/2 of the mesiodistal diameter

31.2011U4A1065 Which of the following descriptions of occlusal support function is incorrect ( )

A. Prevent gingival displacement of dentures

B. Maintain the position of the snap ring on the abutment

C. Prevent food impaction

D. Prevent lateral displacement of dentures and occlusion

E. Restoration of occlusal relationship

32.2010U4A1100 The angle between the concave bottom of the occlusal support and the long axis of the abutment is ( )

A. 90°

B. 10°

C. 20°

D. 25°

E. 30°

33.2010U4A1100 The angle between the concave bottom of the occlusal support and the long axis of the abutment on the abutment is ( )

A. 20°

B. 50°

C. 60°

D. 75°

E. 90°

34.2010U4A1101 Which of the following dentition defects requires indirect retainer ( )

A. Anterior teeth are missing

B. Most posterior teeth are missing

C. Dissociative deletion at the end

D. Missing gaps

E. Most anterior teeth are missing

35.2008U4A1012 The function of the indirect retainer is ( )

A. Enhance the retention, stability and strength of the denture

B. Enhance the retention, stability and aesthetics of the denture

C. Enhance denture retention, support, and strength

D. Enhance denture retention, support, and stability

E. Enhance denture retention, support, and comfort

36.2011U4A1066 Indirect retainer not included ( )

A. Canine support

B. Interproximal hook of anterior teeth

C. Snap ring

D. Metal tongue plate

E. Metal palatal plate

37.2011U4A1067 The retention force of the buccal and lingual snap arm of the posterior teeth should be equal, mainly for ( )

A. Strengthen retention

B. Strengthen the role of enveloping

C. Prevent denture warping

D. Prevent dentures from wobbling

E. Prevent denture damage

38.2003U4AN143 A patient has missing 5678 left and 678 right in the lower jaw, and the lower left 4 is not loose and caries, and the alveolar ridge is full. Cast bracket denture lower left 4 lower right 5 distal middle occlusion support, three-arm card retainer, tongue rod large connector. After wearing the denture for 1 week, he complained of base tenderness and abutment bite pain. Intraoral examination found that there was a small ulcer at the base of the tongue frenulum, the mucosa of the left mandibular convex area was red and swollen, the lower left 4 percussion pain (+), the dentures were tightly sealed, and the bite was not high. In order to reduce the torsional force on the lower left 4, the following measures can be taken, except for ( )

A. Add indirect retainers

B. Use RPI snap rings instead

C. Use a pull-back ring instead

D. Reduction of free end bases

E. Reduction of the number and diameter of artificial teeth

39.2014U4A1033 The role of the snap ring body ( )

A. It plays a role in retaining the position and prevents the denture teeth from moving in the closed direction

B. Stabilizes and prevents the denture from moving laterally

C. Acts as a supporter to prevent the denture from moving in the gingival direction

D. Acts as retention and stabilization to prevent dentures from moving in the closed and lateral directions

E. Stabilizes and supports the lateral and gingival movement of the denture

40.2014U4A1034 Description of casting snap rings, inappropriate is ( )

A. The ring arm is wide and thick at the beginning and narrower towards the tip

B. The tip of the snap ring arm has the functions of retention, support and stability

C. There are three commonly used types of snap ring arms: round, semicircular and flat

D. The snap ring body is located in the non-chamfered area of the abutment

E. The tip of the snap ring arm is located in the abutment inverted area

41.2007U4A1009 The tip of the snap ring retained arm should be located in the abutment ( )

A. Contour high point line

B. Conformal high-point line occlusion

C. Shape of the high point line gingival square

D. Occlusal of the wire

E. Gingival side of the wire

42.2011U4A1068 Compared with cast snap rings, the characteristics of forging wire snap rings are ( )

A. It is not easy to adjust

B. Fit snugly with the tooth surface of the abutment

C. Good elasticity

D. Good interaction and confrontation

E. Good support

43.2008U4A1013 Half snap ring for ( )

A. Excessive teething

B. Abutments with poor crown shape

C. Distal solitary molars with cheek or tongue slanted

D. Isolated bicuspids, molars

E. Loose abutments

44.2000U4A2086 Male, 50 years old, 567 missing lower right, 6 missing lower left, 8 anterior lower right, poor contact with the opposite teeth, normal residual teeth, how to design the upper snap ring of the lower right 8 when designing a dental support denture ( )

A. Three-armed snap ring

B. Three-arm snap and enlarge the support to restore its occlusion

C. Ring snap ring

D. Ring the ring and enlarge the support to restore its occlusion

E. Barb the ring, and enlarge the support to restore its occlusion

45.2007U4A1011 is ( )

A. Gaps

B. Upper reverse snap ring

C. Ring snap ring

D. Three-armed snap ring

E. Pull-back snap ring

46.2008U4A1014 PULL BACK SNAP RING FOR ( )

A. Anterior teeth are missing

B. Absence of spacers

C. Posterior tooth free loss

D. Posterior teeth are not free and missing

E. Individual tooth loss

47.2008U4A1015 Pull Back Snap Ring( )

A. The tip of the arm is located in the inverted area of the labial and buccal surface of the abutment, bypassing the mesial surface of the abutment and connecting with the brace and the mesial lingual side of the abutment

B. The tip of the arm is located in the inverted area of the labial and buccal surface of the abutment, bypassing the mesial surface of the abutment and connecting with the connector on the distal lingual side

C. The tip of the arm is located in the inverted area of the labial and buccal surface of the abutment, bypassing the distal surface of the abutment and connecting with the brace, and connecting with the connector on the mesial lingual side

D. The tip of the arm is located in the inverted area of the labial and buccal surface of the abutment, bypassing the distal surface of the abutment and connecting with the brace and the distal lingual side of the abutment

E. The tip of the arm is located in the inverted area of the labial and buccal surface of the abutment, bypassing the mesial surface of the abutment and connecting with the brace, and connecting with the connector on the mesial midbuccal side

48.2008U4A1016 Pull back snap ring has stress interrupting effect ( )

A. Because the distal occidental support is connected to the base, the positive force is transmitted to the abutment through the support, which reduces the burden on the alveolar ridge

B. Because the distal occisidal support is connected to the base, the positive force is transmitted to the alveolar ridge through the artificial tooth and the base, reducing the burden on the abutment

C. Because the distal occlusal support is not connected to the base, the resultant force is transmitted to the abutment through the support, reducing the burden on the alveolar ridge

D. Because the distal occisidal support is not connected to the base, the positive force is transmitted to the alveolar ridge through the artificial tooth and the base, which reduces the burden on the abutment

E. Because the distal occlusal support is not connected to the base, the positive force is transmitted to the alveolar ridge through the artificial tooth and the base, reducing the burden on the alveolar ridge

49.2003U4A1021 What is effective in preventing food impaction is ( )

A. RPI snap ring

B. Pull back snap ring

C. Joint snap ring

D. Half snap rings

E. Rod snap rings

50.2010U4A1102 The snap ring with stress interrupting effect is ( )

A. Three-armed snap ring

B. Ring snap ring

C. Pull back snap ring

D. Half snap rings

E. Extension snap rings

51.2011U4A2091 Kennedy Class 1 dentition defects, when the hybrid support denture design is adopted, the main purpose of using the stress interrupting snap ring for the terminal abutment is ( )

A. Increase denture retention force

B. Enhanced denture stabilization

C. Facilitates a more uniform distribution of the load over the abutment and alveolar ridge

D. Prevent alveolar ridge mucosal tenderness

E. Reduce the burden on the alveolar ridge

52.2011U4A1069 PRI snap ring is composed of ( )

A. Distal occlusal support, mesial proximal panel, and lingual I-bar

B. Medial occlusal support, distal proximal panel, and buccal I-bar

C. Medial occlusal support, distal proximal panel, and buccal T-ring

D. Distal occissal support, mesial proximal panel, and lingual T-bar

E. None of the above

53.2007U4A1012 The main disadvantage of the rod ring and the ring ring is ( )

A. Poor retention

B. Poor stabilization

C. Poor support

D. Poor elasticity

E. Great damage to the abutment

54.2008U4A1017 Rod-shaped snap ring ( )

A. Good elasticity, small contact area with abutment teeth, weak push-type retention, less damage to abutment teeth, and better stability than annular snap rings

B. Good elasticity, small contact area with abutment, strong push-type retention, small damage to abutment, and better stability than annular snap ring

C. Good elasticity, small contact area with abutment, weak push-and-hang retention, little damage to abutment, and less stable effect than annular ring

D. Good elasticity, small contact area with the abutment, strong push-type retention, great damage to the abutment, and the stabilization effect is not as good as the annular snap ring

E. Good elasticity, small contact area with abutment, strong push-type retention, small damage to abutment, and less stable effect than annular ring

55.2008U4A1018 Comparison with distal occlusal support ( )

A. Medial occlusion reduces the torsional force on the abutments and reduces the burden on the alveolar ridge

B. Medial occlusion minimally reduces the torsional force on the abutments but increases the burden on the alveolar ridge

C. The mesial occlusion supports increase the torsional force on the abutments and also increase the burden on the alveolar ridge

D. Medial occlusion supports increase the torsional force on the abutments, but reduce the burden on the alveolar ridge

E. Medial occlusal support does not reduce the torsion on the abutments or the burden on the alveolar ridge

56.2008U4A1019 The main function of the RPI snap ring adjacent panel is ( )

A. Prevent the base from sinking

B. Reduce alveolar ridge stress

C. Aesthetics

D. Retention of enhanced dentures

E. Prevent food impaction

57.2003U4A2079 Patients with missing 5678 left and right mandibles. If the remaining alveolar ridge is full, 1/3 of the left and right lower 4 alveolar bones are resorbed. Left and right lower 4 retainers should be used ( )

A. Three-armed snap ring

B. Gaps

C. RPI snap ring

D. Extension snap ring

E. Ring snap ring

58.2000U4A1010 Type III snap ring (suitable for type 3 observation line) is characterized by ( )

A. Good retention and stabilization, medium support

B. Good retention, stability and support

C. Poor retention, stability and support

D. Good retention, good support, medium stability

E. Good stability and support, poor retention

59.2010U4A1103 Compared with the posterior palatal bar, the anterior palatal bar is characterized by ( )

A. Thick and wide, thickness about 1.5~2mm, width about 3.5mm

B. Thin and wide, thickness about 1~1.5mm, width about 3~3.5mm

C. Thin and wide, thickness about 1mm, width about 8mm

D. Thin and wide, thickness about 0.5mm, width about 5mm

E. Thick and wide, thickness about 2mm, width about 5mm

60.2008U4A1020 The relationship between the lateral palatal bar and the gingival margin is ( )

A. Contact with the gingival margin

B. Leave 1~3mm

C. Leave 4~6mm

D. Leave 7~10mm

E. Leave 11~15mm

61.2008U4A1021 The two ends of the posterior palatal bar are bent forward to ( )

A. Second pair of cuspids

B. Between the second bicuspid and the first molar

C. First molars

D. Between the first and second molars

E. Second molars

62.2008U4A1022 The posterior palate bar is located in ( )

A. Before the maxillary hard area

B. Maxillary hard area

C. After the maxillary hard area, before the flutter line

D. Vibrator line

E. After the flutter line

63.2008U4A1023 ANTERIOR PALATAL BAR SHOULD BE LOCATED ( )

A. Palatal folds

B. After the palatal fold, before the hard area of the upper palate

C. Hard area of the palate

D. After the hard area of the palate

E. Before the flutter line

64.2011U4A1070 The following are small connectors ( )

A. Palate bar

B. Tongue bar

C. Lip rod

D. Tongue plate

E. Booster Network

65.2008U4A1024 The tongue bar should be away from the gingival margin and should have a certain width. When using the tongue bar, the floor of the mouth should be deep enough, and the distance from the gingival margin of the lingual side of the lower anterior teeth to the floor of the mouth should be greater than ( )

A. 4mm

B. 8mm

C. 10mm

D. 15mm

E. 20mm

66.2011U4A2092 Left and right lower 56 missing, design tongue bar, alveolar ridge is inverted concave placement position is ( )

A. Gentle contact with mucous membranes

B. Depends on the type of denture design

C. Only 0.5 mm gap from the mucosa

D. Located in a non-inverted area

E. Located in the undercut area

67.2000U4A2087 Male, 55 years old, wore a mandibular brace removable partial denture for 3 days, and felt severe pain. Check: the lower right 67 and the lower left 67 can be removed partial dentures, the tongue rod is connected, the anterior alveolar ridge lingual side is sloped, and each part of the denture fits well with the tissue. Mucosal ulceration at the lower edge of the tongue shaft, which does not affect the soft tissue activity of the floor of the mouth. The cause of the pain is the tongue bar ( )

A. Compression of the mucosa

B. The edges are not smooth

C. Improper location

D. Inelasticity

E. Excessively thick

68.2008U4A1025 In which case, the tongue rod needs to leave the mucosa 0.3~0.4mm( )

A. The alveolar bone morphology on the lingual side of the mandibular is vertical and non-free missing

B. The alveolar bone morphology of the mandibular lingual side is vertical and free and absent

C. The alveolar bone morphology of the mandibular lingual side is sloped and not free missing

D. The alveolar bone morphology of the mandibular lingual side is sloped and free is missing

E. The alveolar bone morphology of the lingual side of the mandible is inverted

69.2010U4A1104 If the alveolar ridge on the lingual side of the mandible is sloped, the tongue bar should be ( )

A. Parallel to the mucosa, leave the mucosa a little or lightly touched

B. Parallel to the mucosa, without contact, 3~4mm away from the mucosa

C. Parallel to the mucous membranes and in close contact

D. Perpendicular to the mucous membrane and leave a little

E. None of the above

70.2000U4A1011 Removable partial denture base does not have the function of ( )

A. Bear and concentrate the tooth resultant force

B. Protect mucosa and alveolar bone

C. Connect the dentures into a single unit

D. Enhanced denture retention and stabilization

E. Repair of defective soft and hard tissues

71.2000U4A2088 Male, 70 years old. I wore a mandibular removable denture for half a year, and it broke off when I bit something yesterday. Check: lower right 24567 lower left 24567 mucosal support removable partial denture. 3 lingual bases folded longitudinally. The two broken ends are about 1.5mm thick, and the occlusal contact is good. The cause of the breakage of the base is ( )

A. The base is too thin

B. Biting hard food

C. Habitual unilateral chewing

D. Incorrect denture removal

E. Alveolar ridge resorption, the base is not well attached to the tissue

72.2011U4A1071 The thickness of the plastic base is generally ( )

A. 0.5mm

B. 1.0mm

C. 1.5mm

D. 2.0mm

E. 3.0mm

73.2011U4A1072 Which is wrong in the following description of the extension range of the tooth support RPD base ( )

A. Keep the base as small as possible

B. Posterior edge of the free end of the maxillary posterior teeth extended to the pterygomaxillary notch

C. Distal midbuccal aspect of the posterior edge of the maxillary jaw covers the maxillary tubercle

D. The edge protrudes into the inverted area of the tissue

E. The posterior edge of the mandible covers the front 1/2~2/3 of the posterior molars pad

74.2011U4A1073 The parts of the RPD base tissue surface that do not need to be buffered are ( )

A. Buccal side of maxillary tubercles

B. Maxillary hard area

C. Mandibular bulge

D. Post-molar pads

E. Medial oblique crest

75.2011U4A1074 The tip inclination of semi-anatomical teeth in removable partial dentures is ( )

A. 0°

B. 10°

C. 20°

D. 30°

E. 40°

76.2014U4A1035 The reason why hard plastic teeth or porcelain teeth should be chosen for artificial posterior teeth is ( )

A. Good anatomy

B. Easy to grind or adjust occlusion

C. It is not easy to fall off

D. High hardness and wear resistance

E. All of the above are true

77.2007U4A1013 The part of the removable partial denture that plays the role of connection, stability and retention is ( )

A. Retainers

B. Artificial teeth

C. Kitto

D. Large connectors

E. Small connectomes

78.2003U4A1022 In addition to the retention effect, the extension snap ring also has ( )

A. Stress interrupting

B. Prevent food impaction

C. Splint fixation

D. Anti-caries effect

E. Self-cleaning

79.2008U4A1026 Factors related to the functional restoration of removable partial dentures are ( )

A. Location and number of missing teeth

B. Abutment conditions

C. Mucosa and alveolar bone

D. Occlusal relationship of the remaining teeth

E. All of the above

80.2007U4A1014 Methods for reducing denture net force in removable partial dentures, excluding ( )

A. Reduce the buccal-lingual diameter of the artificial tooth

B. Reduced cusp inclination

C. Use plastic teeth

D. Reduce occlusal contact of artificial teeth

E. The number of artificial teeth can be reduced in free end denture restoration

81.2011U4A1075 Removable partial denture artificial posterior teeth occlusion cheek tongue diameter under what circumstances should the diameter of the cheek tongue be narrower than the natural tooth ( )

A. In any case

B. The opposite jaw is a natural tooth

C. Severe resorption of the maxilloalveolar ridge

D. There is more absorption of the alveolar ridge in the edentulous area

E. The number of missing teeth is low

82.2007U4A1015 The conditions that must be met by the removable partial denture retainer do not include ( )

A. No foreign body sensation

B. No corrective displacement of abutments

C. It is not easy to accumulate food

D. Avoid using different types of metals in the mouth

E. When taking and wearing, there is no lateral pressure on the abutment

83.2008U4A1027 The relationship between denture retention force and the depth of the ring arm entering the abutment indentation and the slope of the indentation ( )

A. Enter the inverted deep depth, the inverted concave slope is large, and the retention force is strong

B. Enter the inverted deep depth, the inverted concave slope is small, and the retention force is strong

C. Entering the inverted concave shallow, the inverted concave slope is large, and the retention force is strong

D. Entering the inverted concave shallow, the inverted concave slope is small, and the retention force is strong

E. The retention force and the depth of the undercut are independent of the slope

84.2000U4A1012 The measures to adjust the retention force of removable partial dentures are as follows, except ( )

A. Adjust the denture in place

B. Regulate the dispersion between the abutments

C. Increase or decrease the number of direct retainers

D. If you need to increase the transverse retention force, you should choose a casting snap ring

E. Adjust the depth and slope of the snap ring arm into the undercut area

85.2011U4A1076 Concave In-place Channel Refers to ( )

A. The angular bisector of the long axis extension of the abutment teeth on both sides is the in-place channel

B. By the inclination of the model, the undercut is concentrated on one side, and the direction of the tooth force is in place

C. The inverted concave is concentrated on one side by the inclination of the model, and the direction of the tooth force is not in the same direction

D. The positioning tract is consistent with the long axis of the abutment

E. The alignment of the tract and the direction of the tooth resultant force

86.2007U4A1016 In the principle of selecting removable partial denture abutments, the wrong one is ( )

A. Choose a healthy tooth for the abutment

B. Have a dental disease but have been treated

C. Periodontal disease is under control

D. The closer the tooth is to the gap for abutment retention, the better the support effect

E. When using multiple abutments, the more abutments and tighter the better

87.2014U4A1036 In order to eliminate the instability of removable partial dentures, the following method is wrong ( )

A. Increase the adversarial equilibrium retainer

B. Strive to design mucosal support dentures

C. Equilibrium is applied on the opposite side of the fulcrum or fulcrum line

D. Eliminate the fulcrum formed by occlusal support and snap ring on the remaining teeth

E. Eliminate the fulcrum formed by the base and the tissue

88.2007U4A1017 In order to eliminate the instability of removable partial dentures, the wrong method is ( )

A. Increase the adversarial equilibrium retainer

B. Try to design mucosal support dentures to avoid fulcrums

C. Equilibrium is applied on the opposite side of the fulcrum or fulcrum line

D. Eliminate the fulcrum formed by occlusal support and snap ring on the remaining teeth

E. Eliminate the fulcrum formed by the base and the tissue

**Chapter 7 Implant Dentures**

1.2011U4A1008 Which of the following does not generally cause fracture of the base of the full denture ( )

A. Imbalance of tooth resultant force

B. The base is thinner

C. Alveolar bone continues resorption

D. The base does not fit the mucosa

E. Insufficient recovery of vertical distance

2.2010U4A1031 The following situations require denture relining, except for ( )

A. The denture does not fit well when it is first worn and needs to be relined when it is not properly retained

B. Poor retention of full dentures due to alveolar ridge resorption after a period of wear

C. Broken denture resulting in malfitting after repair

D. Patients with a bladed alveolar ridge with generalized tenderness after full denture restoration

E. Wrong jaw position

3.2011U4A1009 According to the characteristics of edentulous jaw tissue, the zygomatic process belongs to ( )

A. Main support area

B. Sub-support area

C. Buffers

D. Rear embankment area

E. Marginal Closure Zone

4.2011U4A1010 The area where the edge of the denture can not be stretched more is ( )

A. Alveolar ridge

B. Distal mid-buccal angle region

C. Buccal flange area

D. Maxillary tubercles

E. zygomatic process

5.2004U4A1003 The incisor mastoids are an anatomical landmark of the aligned upper central incisors because ( )

A. The incisor mastoid process is located at the anterior end of the maxillary midpalatal suture

B. There is a more stable relationship between the incisor mastoid process and the maxillary central incisors

C. The incisor hole is below the incisor mastoid process, and it is necessary to prevent compression here when arranging the teeth

D. Less variation in the position of the incisor mastoid process

E. The boundary line of the two upper central incisors should be based on the incisor mastoid

6.2004U4A1004 The function of the incisor mastoids is ( )

A. Reference markers for aligning maxillary central incisors

B. Determine the landmarks of the occlusal plane

C. Signs to determine the extent of the base extension

D. Determine the landmarks of the alveolar crest

E. Identification of the rear embankment area

7.2010U4A1032 The purpose of simulating palatal fold in the anterior palatal area of maxillary base is ( )

A. Fabrication of artificial dentures

B. Aesthetics

C. Helps with pronunciation

D. Pleasant taste

E. Increase base strength

8. 2004U4A1005 Anatomical landmarks unrelated to the posterior edge of a full denture are ( )

A. Palate fovea

B. Palatal lobe

C. Posterior fibrillation line

D. Pterygomaxillary notch

E. Post-molar pads

9.2004U4A1006 Palate fovea located in ( )

A. Anterior incisor mastoid process

B. Between the palatal fold and the incisor mastoid process

C. Posterior palatal fold

D. Posterior edge of the maxillary alveolar ridge

E. Junction of hard and soft palate, flanked by midpalatal suture

10.2004U4A1007 The role of the rear embankment area is ( )

A. Positioning of the trailing edge of the base

B. Edge closure

C. Supporting role

D. Tooth Row Marker

E. Buffering

11.2004U4A1008 Maxillary denture posterior embankment area is located in ( )

A. Post-molar pads

B. Anterior palatal fovea

C. Before the anterior fibrillation line

D. Between anterior and posterior flutter lines

E. After the posterior fibrillation line

12.2007U4A1002 The area between the alveolar ridge and the hard area of the maxillary common denture is ( )

A. Main support area

B. Sub-support area

C. Buffers

D. Edge stretch area

E. Marginal Closure Zone

13.2010U4A1033 Which of the following descriptions of edentulous sub-support areas is incorrect ( )

A. Sub-supporting area refers to the labial, buccal, and lingual sides of the maxillary alveolar ridge (excluding the hard area)

B. There is no clear boundary between the secondary and main support areas

C. The support of the sub-supporting area is poor and cannot withstand large chewing pressure

D. There are mucosa, submucosa, and muscle attachment points on the bone surface in this area

E. The mucosa in this area is closely attached to the bone surface, and the mucosa is stable and immovable, which is conducive to denture retention

14.2004U4A1009 According to the characteristics of the tissue structure of each part of edentulous jaw and its relationship with full dentures, edentulous jaw is divided into the following four areas, except for ( )

A. Main support area

B. Sub-support area

C. Marginal Closure Zone

D. Rear embankment area

E. Buffers

15.2004U4A1011 belongs to the edge of the closed area ( )

A. Maxillary alveolar crest

B. Post-molar pads

C. Incisor mastoids

D. Buccal vestibule

E. Mandibular carina

16.2004U4A1012 Belongs to buffer ( )

A. Maxillary alveolar crest

B. Post-molar pads

C. Incisor mastoids

D. Buccal vestibule

E. Buccal frenulum

17.2011U4A1011 According to the characteristics of edentulous jaw tissue structure, the mandibular hyoid crest belongs to ( )

A. Main support area

B. Sub-support area

C. Marginal Closure Zone

D. Buffers

E. Embankment area

18.2011U4A1012 Which of the following is NOT the purpose of the base as a buffer ( )

A. Prevent tenderness

B. Prevent pronunciation from being impaired

C. Prevent compression of gingival tissue

D. Prevent crushing of mucosal tissue

E. Prevent the base from warping

19.2004U4A1013 The part that belongs to the buffer zone is ( )

A. Incisor mastoid area

B. Posterior molar cushion area

C. Embankment area

D. Alveolar crest region

E. Buccal flange region

20.2004U4A1014 The surface most closely related to the action of the labial and buccal muscles of the full denture is ( )

A. Tooth plane

B. Polished surface

C. Occlusal surface

D. Organizational surface

E. Adsorption surface

21.2010U4A2129 When the full denture is first worn, if there is an open anterior tooth, the possible causes are, except for ( )

A. Error in intraoral jaw removal

B. Error in the process of transferring the jaw position

C. Early contact in the distal middle of the maxillary base

D. High occlusion in the posterior region

E. Patient will not use dentures

22.2011U4A1013 The lower dental arch is found to be significantly receding when the full denture is first worn, and the reason may be ( )

A. Improper alignment of artificial teeth

B. The patient's mandible is too receding

C. Occlusion moves back and forth

D. Extension of the lower jaw when determining the horizontal jaw position

E. The vertical distance is too low

23.2010U4A2130 When the denture is first worn, the reason for the need to grind and adjust the occlusion, except for ( )

A. Any occlusion does not fully correspond to the patient's actual situation

B. Improper operation during the denture making process

C. The shape of the artificial tooth cusp is not standard

D. A simple occlusal frame was used for the fabrication

E. The vertical distance is usually predetermined to be higher when taking the jaw relationship

24.2010U4A2131 Full denture first wearing, lateral occlusion and grinding, usually should be adjusted ( )

A. Upper canine

B. Upper anterior teeth

C. Inferior canine

D. Lower anterior teeth

E. Upper posterior teeth

25. 2010U4A1034 When the full denture is first worn, the wrong way to select and grind is ( )

A. In the case of early contact between the median occlusion, the mesiodistal marginal crest and fossa corresponding to the early contact support tip are mainly selected

B. When selecting and grinding lateral occlusal interference, select and grind a small number of occlusal interference points on the non-supporting cusp

C. When the lateral occlusion interference is selected, if it is a canine, the interference is usually dominated by the upper canine

D. When the anterior occlusion does not interfere, such as when the anterior teeth are in contact but the posterior teeth are not in contact, the lower anterior lip bevel is mainly selected

E. When the anterior occlusion is not disturbed, such as when the posterior teeth are in contact but the anterior teeth are not, the distal oblique plane of the upper cusp or the mesial oblique plane of the lower cusp should be grinded

26.2011U4A1014 The median occlusion is found to be normal when the full denture is first worn, and the anterior occlusion and anterior teeth should be sharpened ( )

A. Upper incisor incisor tongue bevel

B. Upper incisor incisal lip bevel

C. Upper incisor lingual fossa

D. Incisor margin of the lower incisor

E. Lower incisor tongue bevel

27.2010U4A1035 When the full denture is first worn, the wrong thing in the patient's medical order is ( )

A. Initial foreign body sensation, nausea, and slurred pronunciation, but gradually improves

B. Dentures should be cleaned after each meal

C. Do not remove your dentures while sleeping

D. If you have pain, you should go to the hospital for follow-up in time

E. Soak in cold water when not wearing

28.2010U4A1036 Full dentures are difficult to place for ( )

A. The inverted indentation is too large

B. The vertical distance is too high

C. The edge of the base is too long

D. Small nodules on the tissue plane

E. None of the above

29.2004U4A1015 The base tissue surface needs to be partially buffered is ( )

A. Misplaced relationship

B. Poor denture retention

C. Poor occlusion function of the anterior teeth

D. Tenderness at the mandibular carina

E. Denture loss when speaking and opening widely

30.2004U4A1016 When the denture is first worn, the wrong thing in the doctor's order given to the patient is ( )

A. If you have a foreign body sensation or slurred pronunciation, you should wear it consistently

B. Eat small pieces of soft food first

C. Avoid biting hard objects

D. There may be pain, so keep wearing it

E. Try to chew with both back teeth at the same time

31.2010U4A1037 Causes of pain in dentures at the first time, except ( )

A. Small nodules in the tissue plane

B. Early contact with individual teeth

C. Impression deformation

D. Plaster model damage

E. The vertical distance is too short

32.2004U4AN124 A patient who wore a full denture for one week complained of laborious chewing, mucosal tenderness, and facial soreness. The solution is ( )

A. Wear it consistently and get used to it gradually

B. Occlusion

C. Base tissue surface buffering

D. Reproduction

E. Relining

33.2003U4AN133 Patients complained that the dentures were easy to loosen after wearing full dentures for 1 week. When taking a medical history, it is important to ask about ( )

A. How to loosen

B. How loose it is

C. When the denture is loose

D. Have you worn dentures in the past

E. Ability to eat

34.2003U4AN137 Patients complained that the dentures were easy to loosen after wearing full dentures for 1 week. When to reliner ( )

A. Unstable dentures

B. Warping of the denture on both sides

C. Unbalanced bite of dentures

D. Poor or poor attachment of denture edges

E. Insufficient cushioning of the maxillary carina

35.2003U4AN134 The patient complained that the denture was easy to loosen after wearing the full denture for 1 week. Check the diagnostics are ( )

A. Check that the bite is stable

B. Let the patient eat something first

C. Check the retention force first

D. Start by opening the patient's mouth wide

E. Make the patient yawn first

36.2003U4AN147 A patient has missing 5678 in the right mandible, and the adhesive denture is repaired, with 4 three-arm cards in the lower right and 47 gaps in the lower left. One week after wearing the denture, he complained of tenderness at the free end of the denture and discomfort to chew. Intraoral examination found that all parts of the denture were tightly sealed, the retention and stability were good, there was no high point in the occlusion, the base extension did not affect the soft tissue activity, and there was no obvious tenderness, redness, swelling or ulceration of the missing mucosa. The cause of denture tenderness is the overloading of the alveolar ridge. Solution( )

A. The kito tissue is heavily lined

B. Diameter reduction or reduction of artificial teeth

C. Add snap rings and supports in the lower left 6

D. A, B and C

E. B and C

37.2003U4AN140 A 59-year-old male patient with missing upper dentition, healthy lower right 8, mesial lingual slope, full alveolar ridge, maxillary scattered at the bony apex, and normal intermaxillary distance. The most closely related factor to denture retention is whether the impression is accurate. After wearing a denture for a week, it is necessary to have a follow-up consultation to adjust the occlusion, and the reason for the denture is sinking. The patient reported nausea from wearing teeth at the follow-up visit, what could be the reason ( )

A. Early posterior tooth contact

B. The posterior margin of the maxillary is too long

C. Poor denture retention

D. Mandibular posterior teeth are aligned too far to the lingual side

E. Yes

38.2013U4A1003 What can cause nausea and increased saliva after wearing maxillary dentures is ( )

A. The posterior edge of the denture base is under-sealed

B. At the buccal frenulum, the base is not cushioned enough

C. Poor shape of the polished surface

D. Posterior teeth aligned on the hemibuccal side

E. The posterior edge of the denture base is too short

39.2003U4AN135 Patients complain that the dentures are easy to loosen after wearing full dentures for 1 week. If the patient states that the denture is easy to fall off only when yawning, what should be checked except ( )

A. Denture edge extension

B. Denture polished surface morphology

C. Suitability of the rear embankment area

D. Whether the straps are out of the way

E. Stability of the denture

40.2003U4AN136 Patients complained that the dentures were easy to loosen after wearing full dentures for 1 week. If the patient states that the denture is prone to fall out only when eating, what should be the focus of the check-up ( )

A. Check the balance occlusion of the dentures

B. Check the base edge extension

C. Check the arrangement of the artificial teeth

D. Check how the embankment area is

E. Check that the lacing is out of the way

41.2003U4AN128 A 65-year-old female with most teeth missing in the whole mouth, only the upper left 78 remained, and the looseness was III.°, the gingiva was red and swollen, the alveolar ridge was of moderate height, and the intermaxillary distance was normal. The work that should be done before repair is to remove the upper left 78. The proposed restoration method is full denture restoration. If the correct method is followed, the impression is made in the following way: ( )

A. Functional impressions were taken on both the upper and lower jaws

B. Anatomical impressions were taken on both the upper and lower jaws

C. Take a functional impression of the upper jaw and an anatomical impression of the lower jaw

D. Anatomical impressions were taken on the upper jaw and functional impressions were taken on the lower jaw

E. Either of the above

42.2004U4AN123 A patient who wore a full denture for one week complained of frequent bite of the left back tooth and no other discomfort. The most likely reason is ( )

A. Left posterior tooth coverage is too large

B. Left posterior tooth coverage is too small

C. Left posterior occlusion interference on the working side

D. Left posterior tooth balance side occlusion interference

E. Interference of occlusion on the balance side of the right posterior teeth

43.2011U4A1015 Cheek biting phenomenon with full dentures, need to be sharpened ( )

A. Upper posterior teeth lingual apical lingual bevel and lower posterior teeth lingual apical buccal bevel

B. Upper posterior teeth lingual apical buccal bevel and lower posterior lingual apical lingual bevel

C. Upper posterior buccal bevel and lower posterior buccal bevel

D. Upper posterior buccal aperical tongue bevel and lower posterior blucial acupuncture bevel

E. Upper posterior blucial aical lingual bevel and lower posterior pacual apical buccal bevel

44.2003U4AN141 A 59-year-old male patient with missing upper dentition, healthy lower right 8, mesial lingual slope, full alveolar ridge, maxillary scattered at the bony apex, and normal intermaxillary distance. The most closely related factor to denture retention is whether the impression is accurate. After wearing a denture for a week, it is necessary to have a follow-up consultation to adjust the occlusion, and the reason for the denture is sinking. The patient reported nausea from tooth wearing at the follow-up visit, which may be the cause of the long posterior edge of the maxillary. The patient bites his lip and cheek while eating due to ( )

A. Insufficient extension of the denture margin

B. The vertical distance is too high

C. The median relationship is incorrect

D. The arrangement of artificial teeth covers too little

E. All of the above are true

45.2004U4A2078 A 65-year-old female patient underwent full denture restoration after extraction due to periodontal disease 3 years ago, and the effect was satisfactory. Recently, he had pain in his bite. Possible causes of pain ( )

A. The alveolar ridge has a bony tip

B. Excessive bite force

C. The edge of the base is too long after alveolar ridge resorption

D. Inflammation of the mucous membranes

E. Unbalanced occlusion

46.2004U4A1017 The retention of full dentures refers to ( )

A. Dentures do not fall out when chewing

B. The denture does not fall out when the mouth is wide open

C. The denture does not fall out when speaking

D. The denture does not fall out when the tongue is raised

E. Resistance when removing dentures from the mouth

47.2007U4A1003 In the restoration of total dentures, the force acting between saliva and the base should be called ( )

A. Adhesive strength

B. Attractiveness

C. Adhesion

D. Adhesion

E. Adhesion

48.2011U4A1016 Full denture retention mainly depends on ( )

A. Adhesion

B. Adhesion

C. Cohesion

D. Tongue and buccal muscle pressure

E. Adsorption

49.2014U4A1018 The factors most closely related to adsorption are ( )

A. The degree of tightness of the base tissue surface to the mucosa

B. Whether or not you have worn an old denture

C. Amount of saliva

D. Whether or not to use soft lining materials

E. Thickness of the base

50.2000U4A2080 A patient, female, 82 years old, with missing dentition, alveolar ridge stenosis, and low chewing efficiency after full denture restoration, the reason for which could not be ( )

A. Being too old

B. Alveolar ridge narrowing with poor retention

C. The vertical distance is too low

D. Fewer occlusal contact points

E. The artificial tooth model is too small

51.2011U4A1017 Factors not related to atmospheric pressure are ( )

A. Contact area of the base with the mucosa

B. Tightness of the base to the mucosa

C. Denture edge closure effect

D. Polishing degree of polishing surface of the base

E. All of the above are true

52.2014U4A1019 What has nothing to do with full denture retention is ( )

A. Base area

B. Alveolar ridge morphology

C. Mucosal properties

D. Saliva properties

E. Occlusal balance

53.2010U4A1038 Factors related to full denture retentionWhich of the following is false ( )

A. Anatomical morphology of the jaw

B. Nature of the oral mucosa

C. Alveolar bone density

D. Stretching of the base

E. Quality and quantity of saliva

54.2004U4A1018 The mucosal condition that is favorable for the retention and support of the full denture is ( )

A. Thick and fluffy

B. Thick and tough

C. Thin

D. Thin and dry

E. B or D

55.2013U4AN124 When wearing the full denture for the first time, it was found that the maxillary common denture was tilted from side to side, and the patient felt pain during afterburner. Among the following causes of denture warpage, the first reason to consider should be ( )

A. The kito is stretched too long

B. Kito deformation

C. Inaccurate impressions

D. Inaccurate model

E. The base tissue plane corresponding to the hard zone is not buffered

56.2010U4A1039 Full denture suitable concave polished surface form can be ( )

A. Reduced chewing efficiency

B. Make the pronunciation clear

C. Help with denture retention

D. Avoid biting your cheeks and tongue

E. Increases facial fullness

57.2011U4A1018 Edentulous oral special examination content does not include ( )

A. Size, morphology, and location of the maxillary arches and alveolar ridges

B. Alveolar ridge resorption

C. Examination of the oral mucosa

D. The size, shape, and functional status of the tongue

E. Saliva pH

58.2011U4A1019 Surgical excision is generally not advocated before repair ( )

A. Sharp bony protrusions, bony apex, and bony ridges

B. Formation of a large inverted mandibular carina underneath

C. The labial and buccal folds are too shallow

D. Laborobuccal frenulum attachment close to the alveolar ridge

E. Soft alveolar ridge

59.2004U4A1019 Surgical excision is generally advocated before repair ( )

A. Inverted maxillary tubercles

B. Sharp bony apex, bony procession, and bony ridge

C. Hyperplasia of mucosal tissue

D. Lalabial and buccal frenulum attachment points close to the alveolar ridge

E. All of the above

60.2010U4A1040 Alveolar bone trimming is not included in the parts that are commonly required before denture restoration.

A. Maxillary nodules

B. Maxillary labial side

C. Post-molar pads

D. Lingual side of mandibular bicuspids

E. Extraction wound site

61.2004U4A2079 A patient with edentulous jaw had a normal relationship between the upper and lower mandibular arches, a plump alveolar ridge, and a sharp bone spur on the labial side of the alveolar ridge at the upper jaw equivalent to the left canine position. This patient will need to undergo ( )

A. Deepening of labial-buccal folds

B. Maxillary tubercle trimming

C. Alveolar bone trimming

D. Lip tie trimming

E. Buccal frenulum trimming

62.2011U4A1020 The method of increasing the relative height of the alveolar ridge is ( )

A. Lip-tongue frenulum correction

B. Alveolar ridge repair

C. Bony carina

D. Vestibular sulcus deepening

E. Alveolar ridge reconstruction

63.2003U4AN138 A 59-year-old male patient with missing upper dentition, healthy lower right 8, mesial lingual slanting, full alveolar ridge, maxillary scattered at the bony apex, and normal intermaxillary distance. The factors most closely related to denture retention are ( )

A. Accuracy of the impression

B. Whether the jaw position is recorded correctly

C. Whether the position of the tooth row is correct

D. Whether the edge of the base is stretched correctly

E. Whether the tooth selection is suitable

64.2004U4A1020 What does not meet the requirements of edentulous impressions is ( )

A. The impression is detailed and clear

B. Stretch the edges as far as possible

C. The edges should be round and blunt, with a certain thickness

D. Tissue compression is uniform

E. Take a functional impression

65.2011U4A1021 Which is wrong about the full denture model description ( )

A. Shows signs of muscle function trimming

B. The width of the edge of the model should be 3~5mm

C. The thinnest part of the model should not be less than 5mm

D. The posterior edge of the model should be no less than 2 mm behind the palatal fovea

E. Mandibular model with posterior molars pad not less than 10 mm from the anterior edge

66.2007U4A1004 When selecting an antulous maxillary tray, its trailing edge length should be ( )

A. On the line between the pterygomaxillary notch and the palatal fovea

B. 0.5 mm above the flutter line

C. 1 mm above the flutter line

D. Flush with the fibrillation line

E. 3~4mm over the flutter line

67.2006U4A1001 When recording the maxillary position relationship of full dentures, the erroneous statement about occlusion is ( )

A. The occlusal tray is composed of a base and a wax bank

B. There are two types of base, temporary base and Henderson base

C. A base made of base wax tablets is called a temporary base

D. A base made of self-coagulation plastic is called a henderson

E. Temporary base is finally replaced by thermoformed plastic

68.2004U4A1021 Clinically used to record the relationship between the jaw ( )

A. Kitto

B. Temporary Basis

C. Occlusal embankment

D. Occlusal brace

E. Wax base

69.2010U4A1041 Determination of the maxillary relationship of full dentures refers to ( )

A. Restore the appropriate height of the lower 1/3 of the face and the physiological posterior position of the condyles on both sides

B. Restore facial physiology

C. Determine the correct intermaxillary distance

D. Correction of habitual jaw misalignment

E. Determine the vertical distance of the face

70.2014U4A1020 Determining the jaw relationship refers to ( )

A. Restore facial physiology

B. Restore the appropriate vertical distance of the face

C. Determine the correct intermaxillary distance

D. Restore the physiological posterior position of the condyle and the lower 1/3 height of the face

E. Correction of habitual protrusion of the jaw

71.2004U4A1022 When making the rear embankment area on a plaster cast, the width at the widest point is ( )

A. 1mm

B. 3mm

C. 5mm

D. 8mm

E. 10mm

72.2011U4A1022 The main purpose of using occlusion in the production of full dentures is ( )

A. Easy tooth arrangement

B. Restore vertical distance

C. Maxillary recording

D. Restore facial shape

E. Determine the occlusal plane

73.2010U4A1042 Which of the following descriptions of the occlusal surface of the full crown is wrong ( )

A. The original occlusal surface size and shape must be restored

B. It should be in harmony with the morphology of the adjacent occlusal surface

C. It should be in harmony with the morphology of the occlusal surface

D. The direction of the positive force is close to the long axis of the tooth

E. No early contact

74.2004U4A1023 The occlusal embankment plane of the posterior area should be parallel to ( )

A. Upper lip

B. Pupil connection

C. Bottom of the nose

D. Alar tragus line

E. Orbital auricular plane

75.2004U4A1024 The relationship between the embankment plane and the lower edge of the upper lip is ( )

A. 2 mm above the lip

B. Lip level

C. 2 mm below the lip

D. 3 mm below the lip

E. 4 mm below the lip

76.2014U4A1021 Occlusal vertical distance refers to ( )

A. The distance between the pupil line and the ostom

B. The distance from the base of the nose to the base of the chin when the upper and lower teeth of the natural dentition come into contact

C. The distance between the maxillary and maxillary alveolar crests

D. The distance from the base of the nose to the base of the mind when the natural dentition is in the midmaxillary position

E. Distance between the upper and lower jaws of edentulous jaws

77.2004U4A1025 The distance between the alveolar crest of the upper and lower mandibular crest when the lower jaw is in the median position in edentulous patients is called ( )

A. A. Attancy

B. Occlusal space

C. Vertical distance

D. Intermaxillary distance

E. Overbite

78.2011U4A1023 The method of determining the vertical distance by using the jaw position is ( )

A. The distance from the base of the nose to the base of the chin is measured in the jaw position, which is the vertical distance

B. When measuring the jaw position, the distance from the bottom of the nose to the base of the chin is subtracted by 2~3mm, which is the vertical distance

C. The distance from the corner of the mouth to the base of the chin is the vertical distance when measuring the rest and jaw position

D. The distance from the corner of the mouth to the base of the chin is subtracted by 2~3mm when measuring the jaw position, which is the vertical distance

E. None of the above methods are correct

No Thought Chain GPT-4 answers:

These questions appear to be multiple-choice questions about prosthodontics, involving the design and material selection of gold porcelain crowns, dental preparations, and restorations. I will answer these questions based on the basic principles of prosthodontics:

1. E. All of the above
2. C. Metal porcelain crown
3. E. All of the above
4. A. The lowest elastic mold
5. A. The modulus of elasticity is small
6. D. Chemical binding
7. C. Ultrasonic cleaning
8. B. 1.5mm
9. B. 1mm
10. E. None of the above
11. D. Avoid the occlusal zone at the gold-porcelain junction
12. B. 1.0mm
13. B. The gold-porcelain junction should avoid the occlusal functional area
14. E. Misfit of the edges
15. B. Prepare 1 mm space on the lingual side of the anterior teeth
16. A. Gingival hyperplasia
17. E. All of the above are their functions
18. E. All of the above requirements should be met
19. E. May increase chewing power
20. A. Tooth preparation is done in stages
21. E. All of the above are true
22. E. All of the above
23. D. Unsightly in the anterior area
24. E. Age
25. C. Supragingival shoulder margin
26. B. Infragingival shoulder margin
27. E. None of the above
28. A. Bladed
29. A. Bladed
30. E. Whether the contralateral crown is intact
31. E. The gold porcelain articulation area of the gold porcelain crown is located away from the occlusal contact point
32. A. Reduce the volume and thickness of the restoration so that more tooth tissue is preserved

These answers are based on common knowledge and practice principles in prosthodontics. However, it is also advisable to consult the relevant dental materials and technical guidelines to ensure accuracy.

Here are the answers to the questions based on dental prosthodontics and restoration:

33. B. All-ceramic crown
34. This is incorrect because ceramic crowns are known for being brittle and not high in strength
35. D. Not suitable for all-ceramic crowns due to the presence of vital pulp in primary and young permanent teeth with aesthetic concerns
36. E. All the options are indications for cast full crowns
37. B. Full crown provides the greatest retention
38. B. Cast full crown is most appropriate for a short clinical crown with a vertical crack affecting the pulp
39. E. Residual roots are not an indication for cast full crowns
40. C. 1.0mm (The occlusal clearance for cast metal full crown preparation is typically 1.0mm)
41. B. 0.8～1.5mm (The interocclusal space for cast full crowns is generally 0.8 to 1.5mm)
42. E. Prophylactic extension is incorrect for inlay preparation
43. B. 850～1000℃ (The casting temperature for gold alloys is usually between 850 to 1000°C)
44. This is incorrect as the buccolingual diameter of the prepared tooth does relate to retention
45. A. Increasing the convergence angle during tooth preparation is not beneficial for increasing friction of the crown
46. E. Occlusal forces do not affect the retention of full crowns directly
47. B. 1.5mm (The depth of a pinhole for retention is generally 1.5mm)
48. B. The diameter of the retaining nail is about 1mm (This is incorrect; the diameter of retention pins can vary and is not specifically around 1mm)
49. D. Pinhole retention is not a form of retention preparation for restorations
50. E. Groove retention does not need to be in line with the path of placement
51. E. The preparation of dovetails has an effect on the resistance of the preparation (This is incorrect; the preparation of a dovetail does affect the resistance form of the preparation)
52. B. 45° (The bevel angle of a cavity margin is typically 45°)
53. D. Lingual occlusal inlay is not a type of onlay
54. E. Metal (Metal is suitable for inlay restorations, so it's not the incorrect material)
55. A. Compared to amalgam fillings, cast inlays have superior mechanical properties
56. E. All provided options are indications for inlays
57. E. Increasing the seal between the inlay and the margin is not a purpose of the bevel on the cavity margin
58. A. 90° angle to the long axis of the tooth (It is incorrect; the bevel should not be at a 90-degree angle to the long axis of the tooth)
59. A. The primary role of the groove in a 3/4 crown is not to prevent occlusal displacement
60. A. 90 degrees angle to the long axis of the tooth (It is incorrect; the bevel of a 3/4 crown should not be at a 90-degree angle to the long axis of the tooth)
61. B. 3/4 crowns can be used as abutments for fixed bridges, so this is a disadvantage
62. D. The direction of the groove in an upper anterior 3/4 crown
63. C. Parallel to the shaft wall (The direction of the groove in an upper posterior 3/4 crown)
64. B. The location of the groove in an upper posterior 3/4 crown

These questions look like exam questions in dentistry or dentistry. I will give the answers one by one:

65. E. Proper tightness of the contact points
66. C. Nail-retained composite resin restoration
67. E. All of the above is wrong
68. A. Pulpitis
69. E. Early occlusal contact
70. E. Poor finish with rough edges
71. B. The edges of the original fixed prosthesis are not sealed
72. D. Traumatic occlusion
73. A. Removal of the marrow protection material from the cave wall
74. D. Adjust the early contact points on the crown and re-glue after polishing
75. B. Pile crown
76. A. Full crown
77. C. Crown stump for fixed denture abutments
78. D. Pile core + full crown
79. C. Cast metal full crown
80. E. None of the above (usually preserving residual crown tissue is the correct way to increase the length of the crown pile)
81. D. 2/3
82. E. Remove the residual crown hard tissue as much as possible
83. D. The root canal is prepared into a cylindrical shape
84. C. The longer the pile, the better it is to retain (false, because too long a pile may cause too much pressure on the root, increasing the risk of fractures)
85. C. Increased occurrence of apical disease after pile crown repair (error, correct operation should reduce the risk of lesions)
86. E. Preserve the residual crown hard tissue as much as possible
87. C. Seal with the root canal wall
88. D. Use a crack drill to remove the root filling and trim the pipe wall for 1 time
89. E. Facilitation of the wearing of crowns
90. B. Temporary protective repair with a plastic crown
91. B. Protective repair with a plastic crown
92. A. Zinc polycarboxylate cement
93. A. Caries disease
94. C. Damage or abnormality of various tooth tissues with varying degrees of texture and physiological anatomical shape
95. C. Periodontal disease
96. E. Wear occlusal pads

These answers are based on general knowledge of dentistry. In practice, the handling of specific cases may need to be judged according to the specific situation and clinical experience of the patient.

1. B. Alveolar bone
2. D. Anterior teeth are missing, and the abutments on both sides are stable
3. D. Anterior tooth loss with massive bone defects
4. E. Alveolar bone resorption of more than 1/3
5. C. Periodontal tissue health
6. C. The abutment tooth must be a pulp tooth
7. D. June
8. E. 6 months
9. A. Test the pulp viability of the abutment teeth
10. C. Has developed apical periodontitis
11. C. Metal microcurrent stimulation
12. C. There are many tooth tissues to be removed, and the scope of application is small
13. E. The abutment tooth must be a living pulp
14. E. All of the above
15. B. 1:2
16. E. All of the above
17. B. The longer the span of the bridge, the more curved, and the greater the tooth force, the smaller the retention force of the retaining body
18. B. The retention force of the retainer at both ends should be substantially equal
19. E. All of the above should be done
20. A. It is easy for food to pass through and has a self-cleaning effect
21. E. All of the above
22. D. Increase to 8 times
23. E. Movable connectors are generally bolted attachments
24. C. Cast metal fixed bridge repair
25. B. Insufficient retention force
26. E. The retention force of one end of the retainer is slightly higher than that of the other end
27. E. All of the above components are fixed dentures
28. C. Semi-fixed bridges
29. A. Stress buffering
30. B. It is more difficult to achieve a common path
31. E. All of the above
32. B. Single-ended fixed bridge
33. E. The upper right 3 crowns are short
34. A. The lower left 45 was used as an abutment for a single-ended fixed denture
35. C. Pile core and double-ended fixed bridge
36. A. The lower right 5 is best designed with a full crown retainer
37. E. All of the above
38. A. Ceramics
39. B. Fabrication on a model
1. C. The base enters the inverted
2. A. The impression material is not of good quality
3. E. The base does not fit into the mucosal tissue
4. B. Bilateral free deletions
5. B. Category II, subcategory I
6. Category I, subcategory I
7. E. Category IV
8. A. Category I
9. B. Bilateral free end deletion
10. C. Category II, subcategory II
11. E. Category IV
12. E. Category IV
13. C. Category III
14. B. Straight
15. D. The denture base area is large
16. E. The base cushioning of the undercut area is insufficient
17. B. Buffering at the corresponding point of the tissue surface of the denture base
18. D. The alveolar ridge is overburdened
19. E. All of the above tests are required
20. A. Make the most of natural gaps
21. A. Anatomical impressions
22. A. Accurately reflects the position and morphology of the perioral muscles during functional activity
23. A. Recording the occlusal relationship of the remaining teeth by wax biting
24. E. The base tissue plane corresponding to the hard zone is not buffered
25. D. Reduce the load on the support organization
26. C. The wire drawn by the abutment tooth tilted in the opposite direction of the gap is a type II wire
27. A. The proximal notch side of the abutment tooth is inverted very smallly
28. B. Distal gap side
29. B. Abutments
30. A. The width is 1/3 of the buccal tongue diameter and the length is 1/4 of the mesiodistal middle diameter
31. E. Restoration of occlusal relationship
32. C. 20°
33. A. 20°
34. C. Dissociative deletion at the end
35. D. Enhance denture retention, support, and stability
36. D. Metal tongue plate
37. A. Strengthen retention
38. E. Reduction of the number and diameter of artificial teeth
39. D. Acts as retention and stabilization to prevent dentures from moving in the closed and lateral directions
40. E. The tip of the snap ring arm is located in the abutment inverted area
41. B. Conformal high-point line occlusion
42. C. Good elasticity
43. C. Distal solitary molars with cheek or tongue slanted
44. D. Ring the ring and enlarge the support to restore its occlusion
45. D. Three-armed snap ring
46. C. Posterior tooth free loss
47. B. The tip of the arm is located in the inverted area of the labial and buccal surface of the abutment, bypassing the mesial surface of the abutment and connecting with the connector on the distal lingual side
48. E. Because the distal occlusal support is not connected to the base, the positive force is transmitted to the alveolar ridge through the artificial tooth and the base, reducing the burden on the alveolar ridge
49. A. RPI snap ring
50. C. Pull back snap ring
51. E. Reduce the burden on the alveolar ridge
52. A. Distal occlusal support, mesial proximal panel, and lingual I-bar
53. E. Great damage to the abutment
54. A. Good elasticity, small contact area with abutment teeth, weak push-type retention, less damage to abutment teeth, and better stability than annular snap rings
55. A. Medial occlusion reduces the torsional force on the abutments and reduces the burden on the alveolar ridge
56. E. Prevent food impaction
57. C. RPI snap ring
58. D. Good retention, good support, medium stability

These questions appear to be from a dental prosthetics or removable partial dentures (RPD) examination, focusing on the design, function, and clinical considerations of RPDs. Here are the answers based on dental prosthetics principles:

59. B. Thin and wide, thickness about 1~1.5mm, width about 3~3.5mm
60. B. Leave 1~3mm
61. B. Between the second bicuspid and the first molar
62. C. After the maxillary hard area, before the flutter line
63. B. After the palatal fold, before the hard area of the upper palate
64. A. Palate bar
65. B. 8mm
66. E. Located in the undercut area
67. A. Compression of the mucosa
68. D. The alveolar bone morphology of the mandibular lingual side is sloped and free is missing
69. A. Parallel to the mucosa, leave the mucosa a little or lightly touched
70. E. Repair of defective soft and hard tissues
71. A. The base is too thin
72. D. 2.0mm
73. A. Keep the base as small as possible
74. B. Maxillary hard area
75. B. 10°
76. D. High hardness and wear resistance
77. D. Large connectors
78. A. Stress interrupting
79. E. All of the above
80. C. Use plastic teeth
81. D. There is more absorption of the alveolar ridge in the edentulous area
82. D. Avoid using different types of metals in the mouth
83. B. Enter the inverted deep depth, the inverted concave slope is small, and the retention force is strong
84. A. Adjust the denture in place
85. B. By the inclination of the model, the undercut is concentrated on one side, and the direction of the tooth force is in place
86. E. When using multiple abutments, the more abutments and tighter the better
87. B. Strive to design mucosal support dentures
88. B. Try to design mucosal support dentures to avoid fulcrums

These answers are based on general knowledge of removable partial dentures in dental prosthetics and may vary depending on specific academic or clinical guidelines.

These questions appear to be professional dental exam questions about full dentures (full dentures). Let me provide the answer to each question:

1. D. The base does not fit the mucosa
2. E. Wrong jaw position
3. B. Sub-support area
4. E. zygomatic process
5. D. Less variation in the position of the incisor mastoid process
6. A. Reference markers for aligning maxillary central incisors
7. C. Helps with pronunciation
8. D. Pterygomaxillary notch
9. E. Junction of hard and soft palate, flanked by midpalatal suture
10. A. Positioning of the trailing edge of the base
11. D. Between anterior and posterior flutter lines
12. A. Main support area
13. E. The mucosa in this area is closely attached to the bone surface, and the mucosa is stable and immovable, which is conducive to denture retention
14. E. Buffers
15. D. Buccal vestibule
16. E. Buccal frenulum
17. B. Sub-support area
18. E. Prevent the base from warping
19. E. Buccal flange region
20. B. Polished surface
21. E. Patient will not use dentures
22. E. The vertical distance is too low
23. E. The vertical distance is usually predetermined to be higher when taking the jaw relationship
24. C. Inferior canine
25. D. When the anterior occlusion does not interfere, such as when the anterior teeth are in contact but the posterior teeth are not in contact, the lower anterior lip bevel is mainly selected
26. B. Upper incisor incisal lip bevel
27. C. Do not remove your dentures while sleeping
28. E. None of the above
29. D. Tenderness at the mandibular carina
30. C. Avoid biting hard objects
31. E. The vertical distance is too short
32. B. Occlusion
33. A. How to loosen
34. A. Unstable dentures
35. A. Check that the bite is stable
36. D. A, B and C
37. E. Yes
38. A. The posterior edge of the denture base is under-sealed
39. A. Denture edge extension

These answers reflect an understanding of the design, adjustment, function, and adaptability of a full denture.

These questions appear to be from a dental examination, specifically focusing on prosthodontics, the field of dentistry related to the design, manufacture, and fitting of artificial replacements for teeth and other parts of the mouth. Here are the answers based on dental prosthodontic principles:

40. B. Check the extension of the denture base
41. A. Take functional impressions for both upper and lower jaws
42. C. Interference on the working side of the left posterior teeth
43. D. Upper posterior teeth buccal cusp lingual incline and lower posterior teeth buccal cusp buccal incline
44. B. Excessive vertical dimension
45. E. Occlusal imbalance
46. A. The denture does not fall out while chewing
47. D. Adhesion
48. A. Adhesion
49. A. The degree of fit between the denture base tissue surface and the mucosa
50. A. Old age
51. E. All of the above
52. E. Occlusal balance
53. E. The quality and amount of saliva
54. B. Thick and resilient
55. C. Inaccurate impression
56. C. Help with denture retention
57. E. Saliva pH value
58. C. Too shallow labial-buccal sulcus
59. E. All of the above
60. E. Extraction sites
61. C. Alveolar bone adjustment
62. E. Alveolar ridge augmentation
63. A. Whether the impression is accurate
64. E. Taking a functional impression
65. The mandibular model should not be less than 10mm from the anterior edge to the posterior pad
66. B. Exceed the vibrating line by 0.5mm
67. E. Temporary base is finally replaced by heat-cured plastic
68. D. Occlusal splint
69. C. Determine the correct inter-jaw distance
70. B. Restore the appropriate facial vertical distance
71. B. 3mm
72. C. Jaw position recording
73. A. Must restore the original size and shape of the occlusal surface
74. D. Ala-tragus line
75. C. 2mm below the lip
76. D. Distance from the base of the nose to the base of the chin when the natural teeth are in the central jaw position
77. C. Vertical distance
78. A. Measure the distance from the base of the nose to the base of the chin at the rest position, which is the vertical distance

Endodontics

**Chapter 1 Introduction to Caries Diseases**

1.2012U2A1093 Which of the following diseases is not associated with genetic factors ( )

A. Congenital edentulism

B. Periodontal disease

C. Dental caries

D. Malocclusion

E. Opalescent dentin

2.2009U2A1086 The first good tooth surface for caries is ( )

A. Labrobuccal surface

B. Tongue and palatine surface

C. Near-median surface

D. Distal plane

E. Occlusal surface

3.2012U2A1054 The distribution characteristics of dental caries are ( )

A. More neck than occlusal surface

B. More lower jaw than upper jaw

C. There are more anterior teeth than posterior teeth

D. More proximal surfaces than occlusal surfaces

E. More buccal surface than occlusal surface

4. 2012U2A1055 The most common site of caries is ( )

A. Molar occlusal surface pit and fissure

B. Posterior tooth adjudication surface

C. Anterior tooth neck

D. Pits and fissures, adjacency surfaces, and tooth necks

E. Neck of posterior teeth

5.2004U2A1075 Caries is caused by the hard tissue of the tooth ( )

A. Acute progressive destruction

B. Acute intermittent destruction

C. Chronic progressive destruction

D. Chronic intermittent disruption

E. All of the above types of destruction exist

6.2004U2A1076 Caries can also be referred to as hard tissue of teeth ( )

A. Bacterial infectious diseases

B. Viral infectious diseases

C. Immunoreactive diseases

D. Allergic diseases

E. Chemical irritant diseases

7.2012U2A1084 Caries is more common in teeth ( )

A. Self-cleaning area

B. Marginal crest

C. Detention area

D. Tongue surface

E. Cusp

8.2013U2AN148 Among the 300 students in the sixth grade (12-year-old age group) of a school, 180 had caries, lost or filled permanent teeth, 328 untreated caries, 3 teeth lost due to caries, and 69 filled teeth due to caries. The caries rate of students in this grade was ( )

A. 0.64

B. 0.62

C. 0.6

D. 0.58

E. 0.56

GPT-4

1. Diseases not related to genetic factors: C. Dental caries
2. The first good tooth surface for caries: E. Occlusal surface
3. Characteristics of dental position distribution in caries: D. There are more proximal surfaces than occlusal surfaces
4. Preferred site of caries: A. Molars occlusal surface pit and fissure
5. Caries occurs in the hard tissues of the teeth: C. Chronic progressive destruction
6. Caries can also be referred to as the hard tissue of the tooth: A. Bacterial infectious diseases
7. Caries that predisposes to the teeth: C. Detention area
8. Calculation of caries rate: A. 0.64 (180/300)

**Chapter 2 Etiology and Pathogenesis of Caries**

1.2016U2A1031 Foods that are particularly cariogenic are ( )

A. Vegetables

B. Milk

C. Peanuts

D. Cookies

E. Eggs

2.2016U2A1048 The correct statement about plaque is ( )

A. There was no significant difference in plaque structure among the sites

B. There was no significant difference in bacteria within the same oral plaque

C. Plaque consists of a sticky matrix and bacteria embedded in it

D. The main components of the matrix are salivary glycoproteins, the intracellular polymers of bacteria

E. In the plaque-dental interdental layer, the bacteria are scalloped and attach directly to the enamel

3. 2009U2A1093 It is currently believed that the main cariogenic bacteria in humans are ( )

A. Lactobacillus

B. Streptococcus salivarius

C. Hemostreptococcus

D. Streptococcus mutans

E. light streptococcus

4.2009U2A1096 The correct order of supragingival plaque formation process is ( )

A. Acquired membrane formation → initial aggregation→ bacteria grow and multiply rapidly→ plaque matures

B. Initial aggregation → acquired membrane formation→ bacteria grow rapidly and multiply→ plaque matures

C. Initial aggregation→ rapid bacterial growth and multiplication→ acquired membrane formation→ plaque maturation

D. Acquired membrane formation→ bacteria grow rapidly and multiply→ initial aggregation → plaque maturation

E. Initial aggregation→ bacteria grow rapidly → plaque matures

5.2009U2A1103 Food is a cause of caries, which of the following is wrong ( )

A. Retention of food on the tooth surface can cause caries

B. The amount of sugar eaten is more important than the number of times you eat sugar for the occurrence of caries

C. Phosphorus content in food is associated with the occurrence of caries

D. Physical properties of food are closely related to the occurrence of caries

E. Sucrose must be bacteria to cause caries

6.2008U2A1047 The following minerals or elements with anti-caries effect are ( )

A. Fluorine

B. Carbonates

C. Selenium

D. Zinc

E. Iron

7.2008U2A1058 Time required for the formation of an acquired film on a clean tooth surface ( )

A. Minutes

B. 1~2 hours

C. 2~10 hours

D. 10~24 hours

E. 24~48 hours

8. 2010U2A1064 The enzyme that plays a major role in the adhesion of Streptococcus mutans to the tooth surface is ( )

A. Hyaluronidase

B. Glucosyltransferase

C. Lactate dehydrogenase

D. Glucose polymerase

9.2010U2A1068 Streptococcus mutans was divided into 8 serotypes A~H according to the bacterial myrosaccharide antigen, among which the serotype with the highest detection rate in humans was ( )

A. acf

B. def

C. cef

D. dce

E. gef

10.2010U2A1070 Which of the following is wrong about the acquired membrane ( )

A. Its number is inversely correlated with the occurrence of caries

B. Acts as a diffusion barrier to repair and protect the enamel surface

C. Affects the adhesion of bacteria on the tooth surface

D. as a substrate and source of nutrients for bacterial metabolism

11.2012U2A1053 The bacteria that are currently thought to be most closely associated with caries are ( )

A. Lactobacillus

B. Actinomycetes

C. Hemostreptococcus

D. Streptococcus mutans

12.2012U2A1056 Antibodies most closely associated with the onset of caries in human saliva ( )

A. IgA

B. SIgA

C. IgM

D. IgM and IgA

GPT-4

1. Foods that are particularly cariogenic : D. biscuits
2. The correct statement about plaque is: C. Plaque is made up of a sticky matrix and bacteria embedded in it
3. Major cariogenic bacteria: D. Streptococcus mutans
4. The correct sequence of the supragingival plaque formation process: A. Acquired membrane formation → initial aggregation→ bacteria grow rapidly and multiply→ plaque matures
5. Misconceptions that food causes caries: B. The amount of sugar eaten is more important than the number of times you eat sugar for the occurrence of caries
6. Anti-caries minerals or elements: A. fluorine
7. Time required for acquired film formation: B. 1~2 hours
8. The enzyme that plays a major role in the adhesion of Streptococcus mutans to the tooth surface: B. mutans Glucosyltransferase
9. The serotype with the highest detection rate in humans: A. acf
10. On the terms of acquired membrane errors: A. Its number is inversely correlated with the occurrence of caries
11. The bacteria most closely associated with caries: D. Streptococcus mutans
12. Antibodies most closely associated with the onset of caries: B. SigA

**Chapter 3 Clinical Manifestations and Diagnosis**

1.2003U2A1007 Transillumination method, which needs to be carried out in a dimly lit room, can be used to inspect ( )

A. Adjacent caries

B. Whether the pulp is necrotic

C. Cracked teeth

D. Root canal orifice

E. Either of the above

2.2016U2A2079 Some occlusal surfaces (pits and fissures) caries are tapered, with the bottom facing the dentin and the tip pointing to the enamel surface.

A. Wet caries

B. Violent caries

C. Stealth caries

D. Resting caries

E. Linear enamel caries

3. 2008U2A1036 The most commonly used routine diagnostic method for superficial caries is ( )

A. Temperature stimulation test

B. Inspection method

C. Transillumination

D. X-rays

E. Percussion

4.2008U2A1051 When caries in dentin, the changes produced by the dentin pulp complex are ( )

A. Pulp hyperemia

B. Chronic inflammation of the pulp

C. Pulp degeneration

D. Secondary dentin formation

E. Restorative dentin formation

5. 2010U2A1067 The pain of deep caries is manifested as ( )

A. Provoke pain

B. Spontaneous severe pain

C. Both

D. Neither

6. 2003U2A1017 Caries that is more common in patients with Sjogren's syndrome is called ( )

A. Occult caries

B. Resting caries

C. Rampant caries

D. Root caries

E. Annular caries

7.2013U2A1023 Which of the following methods can be used to help diagnose secondary caries that are not easy to diagnose in clinical practice ( )

A. Visitation

B. Temperature test

C. X-ray

D. D. Dyeing

E. Anesthesia

8. 2012U2A1081 The clinical manifestations of deep caries are ( )

A. Less severe pain in the presence of cold, heat and chemical stimuli

B. Cavities form, painful and sometimes spontaneous in response to cold, heat, and chemical stimuli

C. There may be release pain, which cannot be localized

D. Cavities are formed, pain is caused by cold, heat and chemical stimuli, and the symptoms disappear immediately after the stimulation is removed

E. Cavities form, pain in the presence of cold, heat, and chemical stimuli, and pain persists for a longer time after the stimulus is removed

9.2011U2A1019 Which type of caries classification is most suitable for clinical practice ( )

A. Classification according to the incidence of the disease

B. Classification by rate of progress

C. Classification by anatomical site of injury

D. Classification by depth of lesion

E. Other classifications

10.2012U2A2125 Male, 35 years old. The pain in the right upper posterior tooth is evident when eating, and there is no spontaneous pain. The right upper posterior tooth has been treated with a "filling". Oral examination: there is an amalgam filling on the upper right 6 occlusal surfaces, caries lesions around the filling, percussion (-), probing (-), no loosening. Temperature stimulation test (+), symptoms disappear immediately after the stimulation is removed. Can be diagnosed as ( )

A. Resting caries

B. Secondary caries

C. Violent caries

D. Sneaking caries

E. Root caries

11.2014U2A2126 Female, 18 years old. The upper anterior teeth are dotted with blackness between the teeth. There is no spontaneous pain and pain of cold and heat irritation. Oral examination: punctate dark spots on the upper left and right 1 proximal and mid-adjacent surfaces, rough surface, percussion (-), probing (-). The most likely diagnosis is ( )

A. Superficial caries

B. Medium caries

C. Deep caries

D. Resting caries

E. Secondary caries

12. 2014U2A2127 patient, female, 20 years old. Present to the doctor because of the discomfort of sweet food in the left back tooth. Examination: cavities on the lower left 6 occlusal surfaces, medium depth, soft and slightly sensitive, slightly sensitive after cold stimulation into the cavity, and the hot and cold test was the same as that of the control teeth. The diagnosis of the affected tooth may be ( )

A. Superficial caries

B. Medium caries

C. Deep caries

D. Pulp congestion

E. Chronic pulpitis

13. 2014U2A2128 patient female, 20 years old. Present with pain in impacted food when eating in the left back tooth. Examination: caries cavity on the occlusal surface of the lower left 6, sensitive to the bottom of the cave, percussion (-), pain after cold stimulation into the hole, immediate disappearance of the removal of the stimulus, and the same as the control tooth for thermal measurement. The dental diagnosis may be ( )

A. Superficial caries

B. Medium caries

C. Deep caries

D. Chronic pulpitis

E. Acute pulpitis

14.2014U2A2130 The patient sought medical attention with sweet tooth pain in the right lower back teeth. Examination: lower right 6 distal median caries, sensitive, hot and cold measurement with control teeth. The pigmentation of the lower right 7 cheeks can be jammed, and the cold test is the same as that of the control tooth, and the diagnosis is ( )

A. Superficial caries in the lower right 7 buccal surfaces, and distal median caries in the lower right 6 faces

B. Distal median surface in the lower right 6 and middle buccal surface in the lower right 7

C. Middle caries in the lower right 7 buccal faces, and deep caries in the distal face in the lower right 6

D. Superficial caries on the lower right 7 buccal surfaces, and deep caries on the distal and median surfaces in the lower right 6 below

None of the above diagnoses are correct

GPT-4

1. Transillumination can be used to examine: E. All of the above
2. Caries on the occlusal surface (pit and fissure) with a base towards the dentin: C. Sneaking caries
3. The most commonly used conventional diagnostic methods for superficial caries: B. Inspection method
4. Changes in caries in dentin: E. Restorative dentin formation
5. Painful manifestations of deep caries: C. Both
6. Caries in patients with Sjogren syndrome: C. Rampant caries
7. Secondary caries that is not easy to diagnose can be used: C. X-rays
8. Clinical manifestations of deep caries: B. Caries forms and is painful and sometimes spontaneous in response to cold, heat, and chemical stimuli
9. The classification of caries is best applied to the clinic: B. Categorized by rate of progress
10. Diagnosis of secondary caries: B. Secondary caries
11. Diagnosis of punctate blackening between the two teeth of the upper anterior teeth: A. Superficial caries
12. Diagnosis of left posterior tooth discomfort with sweet food: B. Medium caries
13. Diagnosis of impacted food pain when eating in the left back teeth: B. Medium caries
14. Diagnosis of sweet tooth pain in the right lower back teeth: A. The lower right 7 cheeks have shallow caries, and the lower right 6 have distal median caries

**Chapter 4 Treatment Plan for Caries Disease**

1.2009U2A1101 Treatment of dental caries ( )

A. Pharmacotherapy

B. Pharmacotherapy + abrasion

C. Filling

D. Filling + drug therapy + abrasion

E. Medication + Filling + Extraction

2.2012U2A1029 When filling the cavity with composite resin, which of the following disinfectants should be used ( )

A. 50% vanillol ethyl musk alcohol solution

B. Camphor phenol mixture

C. 75% ethanol

D. Chlorophenol camphor mint

3.2010U2A1075 When the composite resin is filled, the cavity disinfectant used is ( )

A. 50% thymol

B. Camphrol

C. Clove oil

D. Wood grenade oil

E. 75% ethanol

4. 2000U2AN148 female, 35 years old, came to the doctor because the left lower back tooth was sensitive to hot and cold stimuli for 1 week. Examination found that the occlusal surface of the left lower first molar was deeply caries, and the bottom of the cave was sore, and the cold test reaction was transiently sensitive, and percussion pain (-). There is no abnormality in the gums. The diagnosis of the tooth is reducible pulpitis and is managed by ( )

A. Palliative treatment

B. The base of zinc phosphate adhesive powder is permanently filled

C. The base of zinc polycarboxylate cemented powder is permanently filled

D. Zinc oxide clove oil cementing powder pad is permanently filled

E. Direct permanent filling

5. 2015U2AN150 patient, a 25-year-old male, had pain due to eating hot and cold food in the right lower back tooth for 1 week, and had no history of spontaneous pain or nocturnal pain. Examination: cavities on the lower right 7 cheeks, slightly sensitive, percussion (-), loose (-), gingiva (-), cold and hot measurements with control teeth. Cavities on the lower right 6 cheeks, sensitive, percussion (-), loose (-), gingival (-), pain caused by hot and cold measurements, disappears immediately after removal. There is no abnormality in the remaining teeth, the best way to deal with it ( )

A. Amalgam filling on the lower right 7 cheeks, zinc oxide clove oil paste on the lower right 6 cheeks soothing

B. Silver-mercury filling of the lower right 7 cheeks

C. Right lower 67 buccal zinc oxide clove oil paste to soothe

D. Amalgam filling of the lower right 7 buccal surfaces, and endodontic treatment of the lower right 6 buccal faces

E. None of the above is optimal

6. 2010U2A1088 Drug treatment of caries is not suitable for ( )

A. Resting caries

B. Rampant caries

C. Superficial dentin caries

D. Shallow caries that is not holed

E. Deciduous tooth caries

7.2014U2A1033 The purpose of drug treatment for dental caries cannot be achieved ( )

A. Maintain intact dentition

B. Restore the appearance

C. Inhibition of caries progression

D. Avoid complications

E. Protect the pulp

8.2005U2A1042 Over time, once a cavity is formed, the tooth ( )

A. There is a possibility of self-repair

B. After antimicrobial treatment, there is a possibility of self-repair

C. After being treated by a special induction method, there is a possibility of self-repair

D. There is no possibility of self-repair

E. Rarely self-repairing

GPT-4

Treatment of dental caries: D. Filling + drug therapy + abrasion

Disinfectant to be used when filling cavities with composite resin: C. 75% ethanol

Cavity disinfectant used in composite resin filling: E. 75% ethanol

Treatment of reducible pulpitis: D. Zinc oxide clove oil cementing powder pad is permanently filled

The best way to manage pain from eating hot and cold food in the right lower back teeth: A. The lower right 7 cheeks are filled with silver mercury, and the lower right 6 cheeks are soothed with zinc oxide clove oil paste

Medical treatment of caries is not suitable for: C. Superficial dentin caries

Objectives that cannot be achieved by drug treatment of dental caries: B. Restore the shape

Possibility of self-restoration of the tooth after cavity formation: D. There is no possibility of self-repair

**Chapter 5 Abnormal Dental Development and Stained Teeth**

1. 2014U2A1038 Clinical manifestations of deep caries, incorrectly described ( )

A. The caries lesion has reached deep into the dentin

B. Most have hot and cold triggering pain

C. Sensitive to sweet and sour foods

D. Spontaneous pain may also occur occasionally

E. Caries embedded in food is painful

2. 2000U2A1033 is inconsistent with the clinical manifestations of deep caries ( )

A. The bottom of the cave is deep, close to the medullary cavity

B. Food embedding pain

C. Occasional dull pain at night

D. Sweet and sour irritation

E. Pulp viability test normal

3.2003U2A1009 The pathogenesis and pathology of enamel tooth are incorrect ( )

A. When fluorine concentration is too high, alkaline phosphatase activity decreases

B. The surface enamel is porous

C. Poor interstitial mineralization of the glaze columns, over-mineralization of the glaze columns

D. Fluoride has a greater effect on dentin than on enamel

E. Fluoroplamine is caused by the adsorption of foreign pigments

4.2004U2A1072 Structural abnormalities of teeth include ( )

A. Enamel hypoplasia

B. Opalescent dentin

C. Dental fluorosis

D. Tetracycline teeth

E. All of the above

5.2016U2A1034 Deformed central cusp is commonly found in ( )

A. Left and right down 5

B. Left and right on 5

C. Left and right lower 4

D. Left and right on 4

E. Left and right up 45

6.2016U2A1037 Enamel hypoplasia at the incision margin of maxillary lateral incisors indicates that the causative factor has been extended to ( )

A. Before birth

B. Under 1 year of age

C. Year 2 after birth

D. After 2~3 years old

E. After the age of 4

7.2009U2A1097 Which is wrong about the distinguishing point between dental fluorosis and enamel hypoplasia ( )

A. Dental fluorosis is more common in permanent teeth, and tooth enamel hypoplasia is prone to occur in both baby teeth and permanent teeth

B. Enamel hypoplasia with well-defined chalky spots and less defined dental fluorosis

C. Enamel hypoplasia can occur in a single tooth or a group of teeth, whereas dental fluorosis is more common in many teeth

D. Patients with dental fluorosis may have a history of living in areas with high fluoride

E. Enamel hypoplasia: Chalky lines coincide parallel to the enamel growth line, whereas dental fluorosis does not coincide

8. 2009U2A1104 Drugs that can cause tetracycline teeth are ( )

A. Pioneermycin and tetracycline

B. Oxytetracycline and norchlortetracycline

C. Penicillin and tetracycline

D. Streptomycin and chloramphenicol

E. Jethromycin and gentamicin

9. 2008U2A2082 patient, 11 years old, found that the newly erupted left lower 5 with a pointed and long deformed central cusp, the correct treatment is ( )

A. Regular observations

B. Adjust the opposite teeth appropriately, and at the same time grind the tip several times in small quantities

C. Under anesthesia and strict disinfection, the tip is ground off once and the medulla is routinely treated

D. B and C

E. Apical induction

10. 2011U2A1011 Deformed central cusp is most likely to occur in the crown of the tooth ( )

A. Buccal crest

B. Fovea

C. Near median fossa

D. Distal fossa

E. Tongue crest

11.2011U2A1015 glaze beads are often located in ( )

A. Within or near the root fork

B. Tooth nature

C. On tooth enamel

D. Apex

E. 1/3 of the root

12.2000U2A1018 Turner (Turer) tooth is due to ( )

A. Caused by taking tetracycline

B. Caused by excessive fluoride content in drinking water

C. Severe febrile illness in the child

D. Congenital developmental defects

E. Severe periapical infection of deciduous teeth

13.2015U2A2091 The patient was 30 years old, due to the treatment of other decayed teeth, it was found that there was a round blunt conical protrusion in the center of the occlusal surface of the lower left 5, the occlusal relationship between the upper left 45 and the lower right 45 was normal, percussion (-), the cold and heat measurement was the same as the control tooth, how should the tooth be treated ( )

A. No treatment is necessary, regular observation

B. Silver-mercury filling of the preparation hole

C. Filling of glass ions in the preparation hole

D. Directly capping the medulla

E. Cutting of the living myel

14.1999U2A2079 The patient requested aesthetic treatment of the anterior teeth, and felt that there were spots on the posterior tooth surface from the eruption of the teeth, and the surrounding adjacent surfaces also had similar manifestations. Check: scattered yellow-brown and chalky spots of varying degrees can be seen in the whole mouth of the dentition. The tooth is diagnosed with ( )

A. Enamel hypoplasia

B. Dental fluorosis

C. Tetracycline teeth

D. Superficial caries

E. Hereditary opalescent dentin

15. 2012U2A2113 The 30-year-old patient presented with recurrent swelling and pain in the left lower back tooth. Check: no caries in the lower left 5, central round black ring, dentin axis visible on occlusal surface, probe unresponsive, percussion (+), X-ray: apical lesion, apical foramen is flare-shaped. The tooth may be caused by ( )

A. Abrasion

B. Cracked teeth

C. Deformed central cusp

D. Occlusal trauma

E. Enamel hypoplasia

16.2012U2A2114 The patient requested treatment due to white spots on the upper anterior teeth. Examination: chalky spots on the left and right upper 1 labial surfaces, smooth and hard. Yu (-). The tooth is diagnosed with ( )

A. Superficial caries

B. Enamel hypoplasia

C. Dental fluorosis

D. Tetracycline teeth

E. All of the above diseases are possible

17.2003U2A1026 Fluoridation of water source is an effective means of preventing caries, and the standard fluorine concentration of water quality in China is ( )

A. 0.05ppm or more ~ 0.1ppm or less

B. 0.1ppm or more ~ 0.5ppm or less

C. 0.5ppm or more ~ 1ppm or less

D. More than 1ppm ~ less than 5ppm

E. More than 5ppm ~ less than 10ppm

18.2004U2A1080 The following correct statement about the abnormal number of teeth is ( )

A. Manifested by insufficient and excessive numbers

B. Insufficient number of teeth, including congenital and acquired missing teeth

C. Congenital edentulism is a manifestation of mesodermal hypoplasia

D. Wisdom teeth are the most common type of extra teeth

E. Congenital edentulism does not occur in baby teeth

19.2012U2A1072 Among the following diseases, the second bicuspid of the mandibular is more common ( )

A. Deformed central cusp

B. Deformed lingual fossa

C. Deformed root sulcus

D. Deformed lingual tip

E. Glazed beads

20.2012U2A1082 Turner tooth is due to ( )

A. Caused by chickenpox or scarlet fever in children

B. Caused by severe dyspepsia in children

C. Severe periapical infection of primary teeth in children affects the inheritance of permanent teeth

D. Pregnant women with rubella or toxemia

E. Caused by decreased parathyroid function

GPT-4

The clinical manifestations of deep caries, which are incorrectly described: E. Caries embedded in food is painful

Inconsistent with the clinical manifestations of deep caries are: E. Pulp viability test is normal

The incorrect claims about the pathogenesis and pathology of enamel are: D. Fluoride has a greater effect on dentin than on enamel

Structural abnormalities of the tooth include: E. All of the above

Deformed central cusps are commonly found in: A. Left and right down 5

The presence of enamel hypoplasia at the incisal margin of the maxillary lateral incisor indicates that the causative factor has been extended to: C. 2nd year after birth

The wrong points for distinguishing dental fluorosis from enamel hypoplasia are: D. People with dental fluorosis may have a history of living in areas with high fluoride

Drugs that can cause tetracycline teeth are: A. Pioneer mycin and tetracycline

The correct way to deal with the deformed central cusp: D. B and C

The malformed central cusp is most likely to occur in the crown of the tooth: B. Central fovea

Glaze beads are often located at: A. Root fork within or near it

Turner tooth is due to: E. Turer Caused by severe periapical infection of deciduous teeth

Methods for dealing with the deformed central cusp: A. No treatment is necessary, regular observation

Diagnosis of patients treated with anterior aesthetic therapy: C. Tetracycline teeth

Causes of recurrent swelling and pain in the left lower back tooth: B. Cracked teeth

Diagnosis of white plaque on the upper anterior teeth: B. Enamel hypoplasia

The standard fluorine concentration of water quality in China is: C. More than 0.5ppm ~ less than 1ppm

Correct statement about the abnormal number of teeth: A. It is manifested by insufficient numbers and excessive numbers

Diseases that predispose to the second bicuspid of the mandible: E. Glaze beads

Causes of Turner teeth: C. Severe periapical infection of primary teeth in children affects the inheritance of permanent teeth

**Chapter 6 Dental Trauma**

1.2003U2A1001 Tooth fracture often occurs in the following tooth positions ( )

A. Upper front teeth

B. Lower anterior teeth

C. Single cuspid

D. Premolars

E. Teeth grinding

2.2016U2A1045 root fold often occurs in ( )

A. 1/3 of the root apex of mature permanent teeth

B. 1/3 of the roots of young permanent teeth

C. 1/3 of the roots of mature permanent teeth

D. 1/3 of the root neck

E. 1/3 of the root apex of young permanent teeth

3.2000U2A1017 The description of tooth concussion is correct ( )

A. Pulp viability test was performed on the affected tooth with tooth convulsion, and the response was mixed

B. The pulp of a young permanent tooth will not lose its vitality after concussion

C. Young permanent teeth lose their vitality quickly after shock

D. Teeth are usually displaced after concussion

E. Tooth concussion is usually accompanied by a defect in the tooth tissue

4. 2015U2A2092 The patient, 20 years old, had a traumatic injury 1 hour ago, and his upper anterior teeth became shorter and required treatment. Check: the upper left 1 crown is 3mm shorter than the adjacent tooth, the crown is intact, percussion (+), the gingiva is mildly red and swollen, X-ray: periapical periapical membrane disappears. The dental treatment is ( )

A. Wait for it to emerge naturally and observe it regularly

B. Post-reduction fixation, regular observation

C. Reduction and fixation after endodontic treatment

D. Endodontic treatment after 4 weeks of reduction and fixation

E. Uprooting

5. 2015U2A2093 Patient 20 years old, traumatic 10 minutes ago, upper anterior teeth loss, requiring treatment. Check: 1 complete dislocation on the left, 2 on the left and 1 on the right with a complete crown, percussion (±), loose (-), what should the patient do ( )

A. Immediate replantation fixation with regular observation

B. After endodontic treatment, reduction and replantation are fixed

C. 4 weeks after reduction and fixation, root canal treatment

D. Ask the patient to repair the wound after healing

E. Immediate Fix

6. 2015U2A2095 The patient suffered a bruise on his upper anterior teeth during exercise one year ago, and had bite pain at that time, but no other discomfort and was not treated. Discoloration of the crown is found later, which may be due to ( )

A. Pigmentation

B. Pulp necrosis

C. Pulp degeneration

D. Dentin degeneration

E. None of the above

7.2015U2A2098 Trauma to the upper anterior teeth in adults, 1/3 of the crown fold, exposing the pulp, percussion (-). The principle of treatment is ( )

A. Vital myeloidectomy

B. Directly capping the medulla

C. Dry pulp surgery

D. Root canal treatment

E. Plastination therapy

8.2013U2A2124 Patient with upper anterior tooth trauma, present. Check: the upper left 1 crown is loose III. degree, gingival bleeding, percussion (++), X-ray shows that the root fold is located at 1/3 of the neck, and the remaining root is about 15mm long.

A. Fixation

B. Extraction of the affected tooth

C. Remove the crown and restore it directly

D. The crown of the tooth is extracted and the root is treated with a root canal

E. Direct photoadhesive repair

9.2013U2A2130 The patient was 16 years old, male, 1 hour ago, upper anterior tooth trauma, checked: upper left 1 lip dislocation, loose II. degree, percussion (±), mild redness and swelling of the gums, upper right 1 upper left 2 percussion (-), loose (-), pulp viability measurement was the same as that of the control tooth, no root fracture was found on X-ray, the patient was treated as ( )

A. Self-healing

B. Occlusion observation

C. Reduction fixation, observation

D. Reduction and fixation after endodontic treatment

E. Uprooting

10.2012U2A1068 Loss of pulp vitality of young permanent teeth can be done after injury at the latest ( )

A. 4 months

B. 6 months

C. 8 months

D. 10 months

E. More than 1 year

· Fracture usually occurs in **A. upper anterior teeth**

· Root fracture usually occurs in **A. 1/3 of the root apex of mature permanent teeth**

· The correct description of tooth concussion is **A. Pulp viability test was performed on the tooth affected by the concussion, and the response was mixed**

· The patient was 20 years old with traumatic injury to the upper anterior teeth, and the treatment was **D. 4 weeks after reduction and fixation, endodontic treatment**

· Loss of upper anterior teeth, what to do A **. Immediate replantation fixation, regular observation**

· Discoloration of the crown, which may be caused by **B. pulp necrosis**

· For upper anterior tooth trauma in adults, the principle of treatment is **D. root canal treatment**

· Trauma to the upper anterior tooth, treated as **B. Extraction of the affected tooth**

· A 16-year-old male with trauma to his upper anterior teeth, managed for **C. Reduction and fixation, observation**

· Loss of pulp viability in young permanent teeth can occur up to **more than 1 year after injury**

**Chapter 7 Chronic Dental Injury**

1.2003U2A1013 Wedge defects often occur in ( )

A. Upper front teeth

B. Lower anterior teeth

C. Upper and lower mandibular bicuspids

D. Maxillary molars

E. Mandibular molars

2.2016U2A1032 The main symptoms of cracked teeth are ( )

A. Pulp hyperemia

B. Acute pulpitis

C. Acute apical inflammation

D. Chronic pulpitis

E. Any of the above symptoms may occur depending on the location and degree of the fissure

3.2016U2A2078 Superficial fissure, no obvious symptoms, and normal pulp viability, the treatment should be the first choice ( )

A. Open marrow inactivation

B. Full crown restoration

C. Fill the hole

D. Occlusion therapy

E. Extraction of the affected tooth

4.2016U2A1049 The following are not chronic dental injuries ( )

A. Abrasion

B. Wedge defect

C. Acid etching

D. Crown folding

E. Cracked teeth

5.2009U2A1084 Cracked tooth may be accompanied by symptoms ( )

A. Temperature irritation pain

B. Painful bite

C. Spontaneous pain

D. No obvious symptoms

E. All of the above are possible

6. 2008U2A2084 The patient was 72 years old with spontaneous labor pain in the lower anterior teeth. The anterior teeth were obviously worn, the left and right lower 1 were cold (++), percussion (-), and the X-ray showed that the periodontal condition was normal. The route of infection of the pulp is ( )

A. Periodontal infection

B. Bloodborne infections

C. C. Cies

D. Cracked teeth

E. Abrasion

7. 2008U2A1053 The following description of root longitudinal fissure is wrong ( )

A. Most of the patients are middle-aged and elderly

B. Maxillary first molars have the highest incidence

C. X-rays are important for diagnosing root longitudinal cracks

D. The absence of a pulp tooth is one of the important factors

E. For longitudinal root fissures with obvious looseness and wide and deep periodontal pockets, which are ineffective by conservative treatment, they should be extracted

8.2000U2A1015 The main causes of temporomandibular joint disorder caused by severe occlusal wear are ( )

A. The vertical distance between the jaws is too short, causing joint damage

B. Defects of the marginal ridge and developmental sulcus, resulting in incomplete occlusal surfaces

C. Uneven wear leaves high and steep cusps, resulting in occlusal trauma

D. Dentin hypersensitivity, resulting in insufficient dental force and damage to the joints

E. Long-term chewing concentrates the stress of the tooth resultant force and damages the joint

9. 2015U2A2096 The patient requested treatment for the right posterior tooth occlusal pain, hot and cold pain for half a year. Check: no caries in the upper right 6, suspicious fissure in the near and middle, pain caused by hot and cold measurements, after the stimulus is removed, it lasts for a few seconds, and percussion (±). The right posterior tooth is worn to varying degrees, sensitized. The patient's chief complaint is that the principles of dental treatment are ( )

A. No treatment

B. Occlusion

C. Desensitization

D. Full crown restoration after endodontic treatment

E. Fill the hole

10. 2012U2A2110 The patient's left upper back tooth has been biting unwell for half a year, and occasionally has sharp pain when biting a certain place. Examination: no caries and periodontal pocket were found in the upper left 4567, suspicious cracks in the proximal occlusal surface of the upper left 6, cold sensitivity, and no abnormalities were found on X-ray. Which of the following diseases is most likely to suffer from in this tooth ( )

A. Wedge defect

B. Wear and tear

C. Hidden fissures

D. Bruxism

E. Chronic pulpitis

· Wedge defects often occur in **D. maxillary molars**

· The main symptoms of a cracked tooth are **E. Depending on the location and extent of the crack, any of the above symptoms may occur**

· For superficial fissures, treatment should be preferred **C. Cave filling**

· What is not a chronic injury to the tooth is **D. crown fracture**

· Cracked teeth may be accompanied by symptoms **E. All of the above may be possible**

· A 72-year-old patient suffers from spontaneous labor pain, and the infection route of the pulp is **E. abrasion**

· The incorrect description of root longitudinal fissures is **C. X-rays are important in diagnosing root longitudinal fissures**

· The main cause of temporomandibular joint disorder caused by severe occlusal surface wear is **A. The vertical distance between the jaws is too short, causing joint damage**

· The patient had pain in the bite of the right posterior tooth for half a year, and the main complaint was **that the principle of dental treatment was D. full crown restoration after endodontic treatment**

· The left upper posterior tooth has been biting uncomfortably for half a year, and the disease is most likely **C. Occult crack**

**Chapter 9 Prosthodontic Treatment**

1.2015U2A2102 The patient requested treatment due to a hole in the upper anterior teeth. Check: left and right upper 1 near the middle surface caries, the ink-soaked edge crest is not destroyed, the cold test is the same as the control tooth, and the diagnosis is medium caries. During treatment, the superficial layer of dentin is removed. The tooth can be selected after one treatment ( )

A. Glass ionomer cement, composite resin filling

B. Composite resin filling

C. Vitreous ionic cement filling

D. Zinc phosphate cement cushion bottom, composite resin filling

E. Zinc oxide clove oil paste at the bottom, composite resin filling

2.2012U2A1094 Which of the following tooth defects cannot be treated by filling ( )

A. Posterior occlusal cavity

B. Anterior incision defect

C. The bite needs to be elevated

D. Posterior occlusal cavity

E. Wedge defects

3.2017U2A1001 The following are not contraindications for composite resin repair ( )

A. Those who cannot effectively isolate the treatment area

B. When the restoration extends to the root plane

C. Restoration of V. cavities in anterior teeth

D. Patients with deep abrasion or bruxism

E. If all occlusions are on the restoration

**Chapter 10 Tooth Preparation and Amalgam Filling**

1.2016U2A1041 Which of the following is not the resistance form of the cavity ( )

A. Inverted

B. Depth of the hole

C. Box cavity

D. Ladders

E. The shape of the cavity

2.2016U2A1056 The adjacent part of the occlusal part of the occlusal cavity is larger than the occlusal side because ( )

A. Prevent lateral dislocation of the restoration

B. Prevent vertical dislocation of the restoration

C. Affected by the direction of the glazed columns

D. Affected by the orientation of the dentinal tubules

E. Saving filling materials

3.2009U2A1089 The purpose of preparing dovetails is ( )

A. Obtain a good retention

B. Obtain a good resistance shape

C. To facilitate the complete removal of decayed tooth tissue

D. To make the cave shape aesthetically pleasing

E. To facilitate filling

4.2010U2A1054 The main purpose of making dove buckles is ( )

A. Avoid accidental puncture

B. Maximize the preservation of natural tooth tissue

C. Facilitates the restoration of adjacency

D. Prevent the filling from falling off horizontally during chewing

5.2010U2A1055 Standard cavity needs to have ( )

A. Cave walls, corners, edges, fixed shapes and resistance shapes

B. Cave walls, corners, edges and resistance shapes

C. Retained shape and cave walls, corners, and edges

D. Retained and resistant

6.2010U2A2105 15 distal adjacent occlusal surface cavities, according to the Black cavity classification method, should be ( )

A. Class I Cave

B. Class II Caves

C. Class V. Holes

D. Class IV Caves

7.2012U2A1031 Which of the following holes belongs to the category BlackII. cave ( )

A. 43 Adjacent Cave

B. 14 adjacent to the cave

C. 15 buccal side neck hole

D. 36 buccal occlusal cavity

8.2012U2A1032 Which of the following is not conducive to resistance in order to achieve a good resistance shape ( )

A. The cavity should have a certain depth in the dentin

B. The cavity is required to be box-shaped

C. Removal of empty suspension enamel

D. The entrance is small and the bottom is large

9. 2012U2A1033 The definition of resistance shape is ( )

A. Enable the filling to withstand chewing force

B. Make the filling and teeth able to withstand chewing force

C. Enabling the teeth to withstand chewing force

D. Keep the filling from loosening and falling off

10.2012U2A1034 During the filling operation, the purpose of the pad is ( )

A. Isolate irritation and protect the pulp

B. To strengthen the resistance

C. To enhance disinfection

D. To strengthen retention

11. The basic structure of the 2012U2A1037 hole consists of ( )

A. Cave walls and corners

B. Cave walls and cave edges

C. Cave walls and corners, cave edges

D. Walls and corners, edges, bottoms

12.2012U2A2100 47 buccal occlusal cavities, according to the Black cavity classification should be ( )

A. Class II Caves

B. Class I Cave

C. Class III Caves

D. Class IV Caves

13.2012U2A1041 Black Taxonomy is based on ( )

A. Extent of decay

B. Tooth surface where the cavity is located

C. The location of the caries and the characteristics of caverning

D. None of the above

14.2012U2A1042 Distal neck caries of maxillary posterior teeth should be classified as Black ( )

A. Class I Cave

B. Class II Caves

C. Class IV Caves

D. Class V Cave

15.2012U2A1044 Occlusal surface box-shaped hole, generally should penetrate deep into the dentin ( )

A. 0.2～0.5mm

B. 0.5～1mm

C. 1.0～1.5mm

D. 1.5～2.0mm

16.2010U2A1069 The basic characteristics of the box-shaped hole, which of the following is wrong ( )

A. The bottom of the cave should be flat

B. The corners of the line should be round and blunt

C. There should be a certain amount of depth

D. Inward inclination of the cave wall

17.2010U2A1057 Which of the following is wrong in order to achieve a good resistance shape ( )

A. Removal of baseless enamel

B. The cavity has a certain depth in the dentin

C. The bottom angle of the hole is clear

D. Removal of thin-walled weak tips

18.2010U2A1061 Neck hole in the mesial face of the posterior teeth is classified by Black ( )

A. Class II Caves

B. Class III Caves

C. Class I Cave

D. Class V Cave

19.2010U2A1065 According to the classification of black holes, the class IV holes are ( )

A. Begins in the hole of the pit and fissure

B. Posterior tooth proximal cavity

C. Anterior tooth proximal cavity does not include chamfers

D. Anterior tooth adjacent surface holes including climacto

E. Cavities in the third of the teeth of the labial, buccal, or lingual neck

20.2012U2A1048 Posterior occlusal hole application ( )

A. Sidewall retention

B. Subtentive retention

C. Dovetail retention

D. Nail retention

21. The basic characteristics of the box-shaped hole, which of the following is wrong ( )

A. The bottom of the cave should be flat

B. The corners of the line should be round and blunt

C. There should be a certain amount of depth

D. Dovetail buckles should be prepared

22.2010U2A1074 Preferred material for temporary sealing of cavities ( )

A. Zinc oxide, clove oil, cement

B. Zinc phosphate cement

C. Calcium hydroxide

D. Teether

E. Cave protection paint

23.2010U2A1076 According to the Black Cave Classification Method Class III. Cave is ( )

A. Begins in the hole of the pit and fissure

B. Posterior tooth proximal cavity

C. Anterior tooth proximal cavity does not include chamfers

D. Anterior tooth adjacent surface holes including climacto

E. Cavities in the third of the teeth of the labial, buccal, or lingual neck

24. Posterior tooth adjacent occlusal hole application ( )

A. Sidewall retention

B. Subtentive retention

C. Dovetail retention

D. Stent retention

E. Nailing

25.2012U2A1052 The treatment principle of the empty hanging glazed column is ( )

A. Removal

B. Retention in its entirety

C. Lower the height

D. None of the above

26.2015U2A2090 A 20-year-old female presents with a decayed tooth. Check: left 7 buccal occlusal surface dotted caries, soft probe, card probe, cold and hot measurement with the control tooth, multiple teeth in the mouth due to caries filling treatment, the best treatment of the affected tooth is ( )

A. Amercury fills the area involved in decay

B. Vitreous ion-filled parts of the caries involvement

C. Preventive extension, amalgam filling

D. Preventive expansion, glass ion filling

E. Precautionary expansion, composite resin filling

27.2012U2A1061 II. type cavity type occlusal surface dovetail shape design according to ( )

A. Depth of caries on the adjacent surface

B. Location of adjacent holes

C. Tooth position of the adjacent caries

D. Types of proximal caries

E. Size of the adjacent defect

28.2013U2A1024 According to G · V · Black's cavity classification method, which of the following sentences is correct ( )

Class A. I holes are all single-sided holes

B. Class II holes are all double-faced holes

C. Class I cavity is a cavity prepared by posterior pit and fissure caries

D. Type III cavity is a cavity prepared by caries of the adjacent surface of the anterior tooth without damaging the incision

E. V. type hole is a hole prepared by the destruction of the posterior labial (buccal) and lingual (palatal) surfaces near the gingiva

29.2003U2A1020 The medullary wall of the staircase and ( )

A. Gingival walls are parallel

B. Gingival wall vertical

C. Shaft walls are parallel

D. Parallel to the mesial wall

E. Distal wall parallel

30.2003U2A1021 Which of the following measures is wrong when preparing for deep caries ( )

A. The bottom of the cave is flat, the side walls are straight, and the two are perpendicular

B. Remove the decay

C. Protect the pulp

D. The edge of the cave is round and blunt

E. Preserve healthy tooth tissue as much as possible

31.2003U2A1022 When preparing the posterior tooth class II. hole, the width of the dovetail gorge is ( )

A. 1/4 of the buccal and lingual apical spacing

B. 1/4~1/3 of the buccal and lingual apical spacing

C. 1/3~1/2 of the buccal and lingual cusp spacing

D. 1/2~2/3 of the buccal and lingual apical spacing

E. 2/3~3/4 of buccal-lingual cusp spacing

32.2003U2A1023 The posterior tooth II type cavity type of dove tail gorge is located in ( )

A. The angle of the medullary line of the axis of the cave floor is close to the midline

B. At the angle of the medullary line of the axis of the cave bottom

C. The axis of the bottom of the cave is close to the edge crest

D. Dispose of as appropriate according to the caries

E. Any part of the buccal tongue cusp

33.2003U2A1024 trapezoidal retention can be used for ( )

A. Occlusal cavity

B. Adjacent (labial) buccal cavity

C. Adjacent tongue (palatal) facial hole

D. Posterior buccal occlusal cavity

E. All of the above:

34.2012U2A2121 Female, 47 years old. Food impaction in the left lower back tooth was uncomfortable for 3 months. No spontaneous pain. Oral examination: there is a cavity on the adjacent occlusal surface of the lower left 7, percussion (-), probing (±), no loosening. According to the classification method, the cave shape should belong to ( )

A. Cave I type

B. Class II Caves

C. Class III Caves

D. Class IV Caves

E. Class VI Cave

35.2015U2A2101 The patient requested treatment due to a hole in the lower left posterior tooth. Examination: deep cavities in the lower left 6 proximal occlusal surfaces, sensitization, percussion (-), normal cold measurement, transient sensitivity of ice water into the cavity, diagnosis of deep caries. Processed as ( )

A. Amalgam filling

B. Zinc polycarboxylate cement-filling

C. Zinc polycarboxylate cement, silver amalgam filling

D. Vitreous ionic cement filling

E. Endodontic treatment

36.2013U2A1024 According to G · V · Black's cavity classification method, which of the following sentences is correct ( )

Class A. I holes are all single-sided holes

B. Class II holes are all double-faced holes

C. Class I cavity is a cavity prepared by posterior pit and fissure caries

D. Type III cavity is a cavity prepared by caries of the adjacent surface of the anterior tooth without damaging the incision

E. V. type hole is a hole prepared by the destruction of the posterior labial (buccal) and lingual (palatal) surfaces near the gingiva

37.2003U2A1029 Which of the following items is not required by the resistance form ( )

A. The cavity should be flat at the bottom, straight in the wall, clear and round and blunt

B. The depth of the cavity should reach 0.5 mm below the enamel dentin boundary

C. The shape of the hole is rounded and gentle

D. Use root canal piles or dentin nails

E. The angle of the dove tail gorge and the axis medullary line should not be on the same plane

38.2012U2A1089 The following is ( )

A. Dovetail

B. Inverted

C. Sidewall retention

D. Trapezoidal

E. All of the above

39. 2011U2A1021 In the preparation of the cavity, the following does not belong to the resistance design is ( )

A. Removal of baseless glaze

B. Removal of thin-walled weak tips

C. Steps for the preparation of the occlusal cavity

D. Preparation of trapezoidal shape for the occlusal cavity of the adjacent occlusal surface

E. The shape of the cavity is a gentle curve

40.2011U2A1022 The function of the compound hole to make a ladder does not include ( )

A. Dispersion of the tooth resultant

B. Less incision of tooth tissue

C. Conducive to filling

D. Protect the pulp

E. Resistance

41.2011U2A1023 The width of the dovetail gorge in the posterior teeth is the distance between the buccal tip of the tongue ( )

A. 2/3～1/2

B. 1/2

C. 1/3～1/2

D. 1/4～1/3

E. 1/5～1/4

1. Not the resistance of the cavity B. The depth of the hole
2. The adjacent part of the occlusal cavity is larger than the occlusal part because A. prevents lateral dislocation of the restoration
3. The purpose of preparing dovetails is to obtain a good retention
4. The main purpose of making a dove buckle D. Prevent the filling from falling off horizontally during chewing
5. Standard cavities need to have A. walls, corners, edges, retainers and resistances
6. 15 The distal adjacent occlusal cavity, according to the Black cavity classification, should be a B. II type cavity
7. Belonging to the category Black II cave is A. 43 adjacent face cave
8. Not conducive to the resistance is D. a small entrance and a large hole at the bottom
9. Resistance is defined as B. making the filling and teeth able to withstand chewing force
10. The purpose of the pad is to A. isolate irritants and protect the pulp
11. The basic structure of the cave includes D. The walls and corners of the cave, the edge of the cave, and the bottom of the cave
12. 47 buccal occlusal cavities, according to the Black cavity classification should be A. II cavities
13. The Black classification is based on C. the location of caries and the characteristics of cavity
14. Distal neck caries of the maxillary posterior teeth should be a D.V. class hole according to the Black classification
15. The box-shaped hole of the occlusal surface should generally penetrate deep into the dentin C. 1.0~1.5mm
16. The basic characteristic of the box-shaped hole, the mistake is that D. the wall of the cave is tilted inward
17. In order to achieve a good resistance shape, the wrong thing is that C. hole bottom angle is clear
18. The proximal neck hole of the posterior teeth is a D.V. class hole classified by Black
19. According to the classification of black cavities, the IV. type holes are: D. Anterior proximal cavities, including incisions
20. C. dovetail retention should be applied to the occlusal hole of the posterior teeth
21. Basic characteristics of the error: D. Dovetail buckles should be prepared
22. Preferred material for temporary sealing of cavities: C. calcium hydroxide
23. The Type III caves of the Black Cavity Classification are: C. The anterior proximal cavity does not include the chamfer
24. Posterior orthodental occlusal cavity application: C. Dovetail retention
25. The treatment principle of the empty suspended glazed column is: A. Remove it
26. The best treatment for the affected tooth is: D. Preventive extension, glass ion filling
27. II. The dovetail design of the occlusal surface of the cavity type is based on: B. The location of the adjacent hole
28. According to G · V · Black's cavity classification method: B. Class II holes are all compound holes
29. The medullary wall of the staircase during the preparation of the compound cave is the same as: C. The shaft walls are parallel
30. Wrong measures when preparing for deep caries: E. Try to preserve healthy tooth tissue
31. When the posterior tooth class II hole is prepared, the width of the dovetail gap is: B. 1/4~1/3 of the buccal and lingual apical spacing
32. The posterior tooth class II. cavity type dovetail gorge is located at: A. The axis of the bottom of the cave is close to the midline
33. Trapezoidal retaining can be used for: E. All of the above include:
34. According to the classification, the cave shapes should belong to: B. Class II. Holes
35. Processed as: C. Zinc polycarboxylate cement, amalgam filling
36. According to G · V · Black's cavity classification method: B. Class II holes are all compound holes
37. Not required by the form of resistance: D. Use root canal piles or dentin nails
38. The following belong to the retained shape: E. All of the above
39. Those that do not belong to the resistance type design are: A. Remove the baseless glaze
40. The role of the duplex cave as a staircase does not include: E. Resistance shape
41. The width of the dovetail gorge in the hind teeth is: C. 1/3～1/2

**Chapter 11 Application Basis of Dental Prosthetic Materials and Bonding Materials**

1.2010U2A1001 Bonding Mechanism of Etching Composite Resin ( )

A. Chelation

B. Chemical bonds

C. van der Waals

D. Mechanical bonding

E. Binding copolymerization

2.2010U2A1002 Full etching system refers to ( )

A. Preparations that can acidify enamel

B. Preparations that can acidify dentin

C. Preparations that can corrode cementum

D. Preparations that etch both enamel and dentin

E. Preparations that etch both dentin and cementum

3.2016U2A1040 Which term is wrong to describe acid etching ( )

A. Removes the surface layer of tarnished enamel and acts as a mechanical retention

B. Increase enamel surface free energy

C. It can enhance the chemical binding ability of enamel and filling material

D. Increase tooth surface area and roughness

E. Activation of the enamel surface

4.2012U2A1049 The main reason why dentin wet bonding can improve bond strength is ( )

A. Able to bond with intertubular dentin

B. Able to bond with peritubular dentin

C. Formation of resin processes chimeric with dentinal tubules

D. Forms a hybrid layer with the bulging collagen fiber network in dentin

5.2011U2A1016 The retention effect of the fourth-generation adhesive on dentin is produced by ( )

A. Micromechanical chimerism

B. Bonding

C. Chelation

D. Hydrogen bonding

E. van der Waals

6.2012U2A1062 Glass ionomer cement, as a filling material, is characterized by ( )

A. Tooth-colored material

B. Good abrasion resistance

C. Releasing fluorine

D. Limited operating time

E. Chemical binding to tooth tissue

7.2012U2A1085 The effect of acid etching does not include ( )

A. Remove the surface layer of tarnished enamel

B. Reduction of surface roughness

C. Increase enamel surface free energy

D. Activation of the enamel surface

E. Increase tooth surface area

8.2010U2A1086 Acid etching can not play a role ( )

A. Increase the surface area of the teeth

B. Mechanical cleaning action

C. Protect pulp viability

D. Expose clean and fresh enamel

E. A+B+D

9.2003U2A1047 The bonding method of dentin adhesive is mainly ( )

A. Mechanical bonding

B. Chemical bonding

C. Mechanical and chemical bonding

D. Retained cavity

E. None of the above

10.2003U2A1048 Bonding method between composite resin and enamel surface ( )

A. Mechanical locking bonding

B. Chemical bonding

C. Mechanical and chemical bonding

D. Retained cavity

E. Chelation

1. Bonding mechanism of etching composite resin: D. Mechanical bonding
2. The full etching system refers to: D. Preparations that etch both enamel and dentin
3. Wrong description of acid etching: B. Increases enamel surface free energy
4. The main reasons why wet dentinal bonding can improve bond strength: C. Forming a resinous process that chimes with the dentinal tubules
5. The retention of dentin by the fourth-generation adhesive is caused by: A. Micromechanical chimerism
6. Characteristics of glass ionic cement as a filling material: C. Fluoride can be released
7. The effects of acid etching do not include: B. Reduced surface roughness
8. The effects that acid etching cannot do are: Protects pulp vitality
9. Bonding method of dentin adhesive: C. Mechanical and chemical bonding
10. Bonding methods of composite resin to the surface of tooth enamel: A. Mechanically locking bonding

**Chapter 12 Dental Bonding Restoration**

1.2011U2A1005 The following statement about the sandwich technique is correct ( )

A. The glass ions are mechanically mated to the hard tissue of the tooth

B. The ionic surface of the glass should be acid-etched

C. The binding mode of glass ions to composite resin is chemical bonding

D. Reduced the adhesion of the composite resin to the cave wall

E. All of the above are true

2.2003U2A1015 Which of the following is not the purpose of making an inclined plane of the cave wall when the composite resin is filled ( )

A. Increase the etching area

B. Increased Resistance

C. Increased retention

D. Reduce enamel cracks caused by polymerization shrinkage of composite resins

E. More aesthetically pleasing

3.2010U2A1059 When preparing the occlusal surface hole during resin filling, the hole edge should be formed ( )

A. Right angles

B. 45-degree incline

C. Acute angles

D. 60-degree inclined plane

4.2011U2A1040 Composite resin filling class V hole, the following design is correct ( )

A. Small and medium defects should be designed as conservatively as possible

B. Bevel only on the enamel wall

C. Defects with a large range should be prepared for retention

D. The design of the retained shape can be a retained groove

E. All of the above are true

5.2005U2A1053 The composite resin filling body is to widen the width of the enamel acid etching band, and the enamel wall of the hole edge should be made ( )

A. 30° angle short bevel

B. 45° angle short bevel

C. 60° angle short bevel

D. Inclined plane within a 135° angle

E. 120° angle within the inclined plane

6.2003U2A1046 Causes of Adhesive Restorations of Composite Resin Filling Materials, Except ( )

A. The tooth surface is not thoroughly cleaned

B. Acid-etched tooth surface contact with saliva

C. The composite resin filler is thinner

D. The tooth surface is not dry

E. Retainers were prepared

7.2002U2A1088 Notes should be paid to the preparation of composite resin filling hole shape ( )

A. The point line angle should be round and blunt, the enamel wall of the cave edge should be beveled, and the inverted concave should be arc-shaped

B. The bottom should be flat, the wall of the cave should be straight, and the shape of the cave must be deep

C. To remove the baseless enamel and increase the resistance shape

D. In order to obtain a good retention, normal tooth tissue must be removed

E. There is no need to remove baseless enamel or prepare short bevels at the edge corners

8.2003U2A1050 The reasons for impaction after filling are as follows, except for ( )

A. Touchpoints are not restored

B. Decay of adjacent teeth

C. Small abduction gap

D. Overhang of the filling body

E. Forming sheets are used for filling

1. About the Sandwich Technique: C. The combination of glass ions and composite resins is chemically combined
2. It is not the purpose of making an inclined surface of the cave wall when the composite resin is filled: E. More aesthetically pleasing
3. When preparing the occlusal surface hole during resin filling, the hole edge should be: B. 45 degree bevel
4. Composite resin fills Class V holes, the following design is correct: E. All of the above are true
5. The composite resin backfill is to widen the width of the enamel acid etching band, and the enamel wall of the cave margin should be made of: B. 45° angle short bevel
6. Causes of detachment of adhesive restorations of composite resin fillers, except: E. Retains were prepared
7. When preparing composite resin filling cavities, attention should be paid to: A. The point line angle should be round and blunt, the enamel wall of the cave edge should be made into an inclined plane, and the inverted concave should be arc-shaped
8. Causes of impaction after filling, except: E. Forming sheets are used for filling

**Chapter 13 Deep Caries Treatment and Pulp Capping**

1. 2013U2A2135 The 10-year-old child had cold irritation and pain in the front teeth for several days. Examination: the right upper central incisor is near the middle and deep cavity, there is no abnormality on percussion, no loosening, and transient pain is measured coldly. Treatment: decomposing clean, very close to the pith, sensitive. What kind of treatment to choose ( )

A. Secondary derotting

B. Zinc oxide clove oil paste to soothe

C. Calcium hydroxide indirectly caps the pulp

D. Directly capping the medulla

E. Cutting of the living myel

2.2003U2A1025 In the process of hole preparation and filling, the measures to protect the pulp are ( )

A. High-speed drilling rigs must have cooling measures

B. Keep the pit dry when the slow mobile phone is derotted

C. Intermittent abrasion should be used to cut the tooth tissue

D. The bottom of the deep hole should be filled

E. Includes the above

3. 1999U2A2078 Patient female with pain and discomfort in her lower left lower back tooth when eating sweet and sour food. Diagnosis of deep caries. The preparation hole is sensitive, the caries can be exhausted, and the pulp is not penetrated, and ( ) should be selected during treatment

A. Indirect capping

B. Direct capping

C. Palliative treatment

D. Underfilling

E. Endodontic treatment

4.2012U2A2115 Patients with deep caries have more severe excitation pain, and the soft caries at the bottom of the cave can be completely cleaned, and the treatment method should be selected ( )

A. Double layer of bottom, complete the filling treatment at one time

B. Pulp opening and inactivation after local anesthesia, endodontic treatment

C. Tranquilization therapy is done first, and after the symptoms are eliminated at the follow-up visit in one to two weeks, then fill with a double layer

D. Perform a myelinotomy

E. Indirect capping of the medulla and double layer of the bottom to complete the filling treatment at one time

5.2015U2A2103 The patient requested treatment due to a hole in the upper anterior teeth, and there was no spontaneous pain. Check: left and right upper 1 near mid caries, sensitization, cold measurement of transient pain, removal of irritation can be relieved, heat measurement with control teeth. Diagnosis of deep caries and the best treatment of the tooth on the day is ( )

A. Zinc oxide clove oil paste to soothe

B. Ca(OH)₂ preparation for marrow protection, photosensitive resin filling

C. Photosensitive resin filling

D. Vitreous ionic cement filling

E. Endodontic treatment

6. The child was 10 years old, and the anterior teeth were cold and irritated and painful for several days. Examination: the right upper central incisor is near the middle and deep cavity, there is no abnormality on percussion, no loosening, and transient pain is measured coldly. Treatment: decomposing clean, very close to the pith, sensitive. What kind of treatment to choose ( )

A. Secondary derotting

B. Zinc oxide clove oil paste to soothe

C. Calcium hydroxide indirectly caps the pulp

D. Directly capping the medulla

E. Cutting of the living myel

7. 2013U2A2127 The patient was 18 years old, and during the treatment of other teeth, he found that the upper right 2 deformed lingual fossa, deep, cochin probe, and the temperature measurement was the same as that of the control tooth. The treatment of this tooth should be ( )

A. No treatment

B. Precautionary filling

C. Directly cap the medulla

D. Vital myelinotomy

E. Root canal treatment

8.2016U2A1027 pH of calcium hydroxide preparations used to cover the pulp ( )

A. 6.5～7.5

B. 8～9

C. 9～12

D. 12～14

E. 14

9.2016U2A2080 Reducible pulpitis of young permanent teeth, the preferred treatment for patients with deputy and no pulp exposed is ( )

A. Pulp opening and drainage under local anesthesia

B. Palliative treatment

C. Indirect capping of the medulla

D. Vital myelinotomy

E. Local anesthesia extraction

10.2004U2A1079 Pathological changes of the pulp after pulpectomy ( )

A. Calcification of the pulp

B. Intra-root canal absorption

C. Pulp viability is normal

D. Root pulp fixation fibrosis

E. Root canal orifice forms a hard tissue bridge

11.2012U2A1071 Reducible pulpitis caused by deep caries, when indirect pulp capping is used, the commonly used pulp capping agent is ( )

A. Calcium hydroxide

B. Zinc phosphate cohesive powder

C. Glass ionic cementing powder

D. Zinc oxide clove oil cement powder

E. Iodoform paste

12.2010U2A1085 For the tooth with accidental pulp penetration, what kind of pulp capping agent is generally used ( )

A. Calcium hydroxide

B. Zinc phosphate cement

C. Zinc oxide clove oil paste

D. Arsenic anhydride

E. Iodoform zinc oxide

1. The 10-year-old child had cold irritation and pain in his anterior teeth for several days. Calcium hydroxide indirectly covers the medulla
2. Measures to protect the pulp during hole preparation and filling: E. Include all of the above
3. Deep caries sensitive, not pierced medulla, treatment should be selected: A. Indirect capping
4. Patients with deep caries have more severe provocative pain, and the treatment method is selected: C. Plaliative therapy is done first, and after the symptoms are eliminated at the follow-up visit in one to two weeks, then fill with a double layer
5. Deep caries of the upper anterior tooth, the best treatment of the tooth of the day: B. Ca(OH)₂ preparation for marrow protection, photosensitive resin filling
6. Considering the child's age, dental symptoms, and clinical findings, the most appropriate treatment is C. Calcium hydroxide indirectly covers the medulla.
7. Found right upper 2 deformities lingual fossa, deep, treatment options: B. Preventive filling
8. pH of calcium hydroxide preparations used to cover the pulp: C. 9～12
9. Reducible pulpitis of young permanent teeth, depleting of decay without pulp, preferred treatment: C. Indirect capping of the medulla
10. Pathological changes of the pulp after pulpectomy: D. Root pulp fixation of fibrosis
11. For reducible pulpitis caused by deep caries, pulp capping agents commonly used in indirect pulp capping: A. calcium hydroxide
12. For the affected tooth with accidental pulp perforation, the pulp capping agent generally used: A. calcium hydroxide

**Chapter 15 Etiology and Pathogenesis of Pulp Apical Disease**

1.2009U2A1092 Harmful substances produced by bacteria entering the pulp do not include ( )

A. Endotoxin

B. Collagenase

C. Amylase

D. Hyaluronidase

Chondroitinase sulfate

2. 2009U2A1100 The most common causes of pulp infection are ( )

A. Trauma

B. Periodontitis

C. Bloodborne infections

D. Caries disease

E. Dental malformations

3.2008U2A1048 If periapical lesions continue to worsen while receiving root canal treatment, the possibility of bacterial infection around the apical should be considered ( )

A. Porphyria

B. Prevotella

C. Actinomycetes

D. Eubacterium

E. Viron cocci

4. 2011U2A1012 Root canal and periapical infection is ( )

A. Isolated aerobic infection

B. Simple anaerobic infection

C. Co-infections predominantly aerobic infections

D. Co-infections with predominantly anaerobic infections

E. Co-infections of aerobic and anaerobic bacteria are equally important

5.2011U2A1014 Endodontic disease is caused by the infection route ( )

A. Retrograde infection of deep caries and periodontal disease

B. Periodontal disease retrograde infection, deep caries, and chemical irritation

C. Electrical stimulation, occlusal trauma, systemic chronic wasting systemic disease

D. Bloodborne infection, retrograde infection of periodontal disease, deep caries, or dental injury exposing the pulp

E. Trauma, developmental malformations, and accidental perforation

6.2012U2A1073 Physical factors causing endodontic diseases, except ( )

A. Cavity preparation process

B. Occlusal trauma

C. Cavity disinfection

D. Metal material filling

E. Dental trauma

7. 2010U2A1080 The endemic pathogen of pulp infection that occurs only in the infected root canal is ( )

A. Porphyria pulp

B. Porphyria gingivals

C. Clostridium

D. Digestive streptococcus

E. Viron cocci

8. 2011U2A1030 The main causes of pulpitis are ( )

A. Occlusal trauma

B. Chemical irritation

C. Physical stimuli

D. Bacterial infection

E. Allergic reactions

9.2002U2A1071 During the treatment of the root canal of infection, there is often a sudden outbreak of pain within minutes to hours after the enclosure of a certain drug, which may be due to ( )

A. Acute onset of apical outbreak infection

B. Root canal instrument damage to periapical tissues

C. Root canal instruments push bacteria around the apical to cause acute infection

D. Encapsulated drug acts as a hapten to elicit an acute immunoallergic reaction

E. Pulpitis occurs in the residual pulp

10.2006U2A1027 Causes of periapical tissue disease do not include ( )

A. Trauma

B. Chemical irritation

C. Infection

D. Electrical stimulation

E. Immune factors

11.2006U2A1028 The most important route of periapical infection is ( )

A. Deep periodontal pockets

B. Wedge defect

C. Abrasion

D. Accessory root canal

E. Deep caries

12.2000U2A1024 Factors leading to pulp necrosis of the affected tooth due to occlusal trauma are ( )

A. Physical factors

B. Chemical factors

C. Local infection

D. Immunity

E. Lesion infection

1.C. Amylase

2.D. Caries disease

3.A. Porphyria

4.D. Co-infections with anaerobic bacteria predominantly

5.D. Bloodborne infection, retrograde infection of periodontal disease, deep caries or tooth injury exposing the pulp

6.C. Cavity disinfection

7.C. Clostridium

8.D. Bacterial infections

9.C. Root canal instruments push bacteria around the apical area to cause acute infection

10.D. Electrical stimulation

11.E. Deep caries

12.A. Physical Factors

**Chapter 16 Clinical manifestations and diagnosis of endodontic diseases**

1.2009U2A1069 The clinical manifestations of pulp necrosis are ( )

A. Usually asymptomatic, but x-rays show intradental resorption

B. Teeth are dark, discolored, opaque, and have no response to temperature and electrical viability tests

C. Tooth discoloration, rot and odor in the pulp cavity, slow response to temperature and electrical viability test, lower reading than control tooth

D. No obvious spontaneous pain, which can be caused by temperature changes

E. Usually asymptomatic, x-ray shows uneven enlargement of the root canal

2.2003U2A1010 Which of the following is inappropriately described in the clinical classification of endodontics ( )

A. Pulp hyperemia

B. Nonreducible pulpitis

C. Pulp necrosis

D. Calcification of the pulp

E. Intradental resorption

3.2016U2A1030 Which item is not a characteristic of acute pulpitis pain ( )

A. Pain is worse at night than during the day

B. Cannot be located

C. Pain worsening with temperature stimulation

D. Spontaneous pain, paroxysmal exacerbation

E. The sitting position is heavier than the lying position

4.2016U2A1047 Pulpitis based on X-ray results as an important diagnostic basis is ( )

A. Reducible pulpitis

B. Acute pulpitis

C. Chronic pulpitis

D. Calcification of the pulp

E. Pulp necrosis

5. 2016U2A1050 The key points of diagnosis of reducible pulpitis are ( )

A. Complaints of transient sensitivity to temperature stimuli

B. No history of spontaneous pain

C. Dental lesions or periodontal tissue lesions that can cause pulp lesions

D. Decreased response domain values to pulp viability tests

E. All of the above

6.2016U2A1051 Acute pulpitis is characterized by pain ( )

A. Spontaneous pain, exacerbation of heat stimulation pain, reduction of cold stimulation pain, pain localization, percussion pain

B. Hot and cold stimuli, short duration of pain, no spontaneous pain

C. Spontaneous, sharp pain, paroxysmal episodes, intensifying pain with cold or hot stimuli, pronounced nocturnal pain, referred pain

D. Food impaction pain is severe, temperature irritation pain is obvious, and there is no obvious spontaneous pain

E. Persistent dull pain, bite pain, radiating pain

7. 2016U2A1053 The common clinical manifestations of chronic pulpitis at presentation are as follows, except for ( )

A. Bite pain, light percussion pain

B. Severe spontaneous pain

C. Pain of food embedding

D. Hot and cold irritation pain

E. Caries cavities can be probed to perforators

8.2009U2A1083 Reducible pulpitis is distinguished from the serous phase of acute pulpitis by ( )

A. No temperature irritation

B. No chemical irritation pain

C. No spontaneous pain

D. No probing pain

E. No percussion

9.2009U2A1102 Pain characteristics of acute pulpitis do not include ( )

A. Spontaneous, paroxysmal pain

B. Pain exacerbated by temperature stimulation

C. Nocturnal pain

D. Pain cannot be localized

E. Pain is loose or referential, sometimes to the contralateral area of the affected tooth

10.2008U2A1040 The following types of diseases can be pointed out in the pain area, except ( )

A. Acute apical abscess

B. Acute papillitis gingivals

C. Acute pulpitis

D. Dentin sensitivity

Acute periodontal abscess

11.2008U2A1043 Pain characteristics of chronic pulpitis ( )

A. Severe spontaneous pain

B. Severe nocturnal pain

C. Referred pain

D. Mild spontaneous dull pain

E. A+B+C

12.2008U2A1044 The most valuable diagnostic indication for residual myelitis is ( )

A. X-rays

B. Temperature Stimulation Test

C. Electrical test

D. Remove the original supplement to probe for sensation or pain deep at the root of the affected tooth

E. Percussion

13.2008U2A1049 The following are not reducible pulpitis manifestations ( )

A. Food embedding pain

B. Hot and cold irritation pain

C. Sweet and sour irritation

D. Tooth defects close to the pulp tissue

E. Spontaneous pain

14.2008U2A2079 A 32-year-old patient presents with pain in his left lower posterior tooth. The lower left 6 occlusal surfaces showed mercury filling, mild pain on percussion, flaccid pain with cold stimulation, X-ray showed that the filling had reached the pulp cavity, no root filling image, and the apex was normal. Diagnosed as ( )

A. Chronic apical periodontitis

B. Reducible pulpitis

C. Myelitis remnant

D. Deep caries

E. Acute pulpitis

1. B. Teeth are dark, discolored, opaque, and have no response to temperature and electrical viability tests
2. A. Pulp hyperemia
3. E. The sitting position is heavier than the lying position
4. D. Calcification of the pulp
5. E. All of the above
6. C. Spontaneous, sharp pain, paroxysmal episodes, intensifying pain with cold or hot stimuli, pronounced nocturnal pain, referred pain
7. B. Severe spontaneous pain
8. C. No spontaneous pain
9. D. Pain cannot be localized
10. Acute periodontal abscess
11. D. Mild spontaneous dull pain
12. D. Remove the original supplement to probe for sensation or pain deep at the root of the affected tooth
13. E. Spontaneous pain
14. B. Reducible pulpitis

**Chapter 17 Clinical manifestations and diagnosis of periapical disease**

1.2015U2A1012 Diseases that must be diagnosed by X-ray examination are ( )

A. Occlusal caries

B. Acute pulpitis

C. Chronic pulpitis

D. Acute apical periodontitis

E. Chronic apical periodontitis

2. When the periapical transmission area of the 2013U2A1018 X-ray film includes several teeth, the main basis for confirming the pathogenic tooth is the affected tooth ( )

A. Whether there is periodontal disease

B. Presence or absence of sinus tracts

C. Pulp viability

D. Presence or absence of cavities

E. Presence or absence of percussion

3.2016U2A1036 What stage of acute apical periodontitis in clinical practice has the highest pain ( )

A. Acute serous apical periodontitis

B. Acute periapical abscess

C. Acute subperiosteal abscess

D. Acute submucosal abscess

E. Osteitis compact

4.2016U2A1038 According to the typical natural development process of acute purulent apical periodontitis, the clinically visible purulent drainage route does not include ( )

A. Drainage of pus from the crown defect through the root canal from the apical foramen

B. Perforate the bone wall and break through the mucosa to drain pus

C. Perforate the bone wall and break through the skin to drain pus

D. Drainage of pus through the maxillary sinus wall

E. Breakthrough of the nasal floor mucosa to drain pus

5.2016U2A1039 The best auxiliary examination method for chronic apical periodontitis is ( )

A. Percussion

B. Electro-test of pulp viability

C. X-rays

D. Bite diagnosis

E. Transillumination

6.2016U2A1043 What kind of apical periodontitis is common in clinical practice ( )

A. Serous phase of acute apical periodontitis

B. Acute apical periodontitis suppurative phase

C. Apical abscess

D. Acute apical periodontitis

E. Chronic apical periodontitis

7.2008U2A1055 Which is the most common method of purulent drainage in the typical purulent route of acute purulent apical periodontitis ( )

A. Perforating the bone wall and breaking through the mucosa

B. Penetrate the bone wall and break through the skin

C. Drainage through the apical foramen through the root canal

D. Drainage of pus through the maxillary sinus wall

E. Breakthrough of the nasal floor mucosa to drain pus

8. Typical local symptoms of patients with acute subperiosteal abscess in patients with 2000U2A1019 are ( )

A. Degree III loosening, percussion pain (+++) of the affected tooth

B. Red and swollen gingiva at the apex, red and swollen gingiva at the root tip, and painful gingiva

C. Regional lymphadenopathy is swollen and tender

D. Migration groove becomes shallower and deep fluctuations

E. Corresponding facial soft tissue reactive edema

9.2000U2AN134 male, 32 years old. On the 2nd, I had a toothache in the lower right back and could not bite anything. Half a year ago, I found that there was a small bag in the lower right posterior gum, and there was no obvious discomfort at ordinary times. Examination showed deep caries in the distal and median face of the upper right 6, deep caries in the upper left 1, no sensation in the perforation of the medullary hole, percussion (±), there was a fistula opening in the gingiva at the apex of the lower right 8 and 7 lower right sides, and a little pus was squeezed. The lower right 8 anterior leaning impaction, the gingiva is red and swollen obviously, and there is pus in the deep part of the gingival pocket. X-ray showed that the X-ray transmission area around the distal apical area of the upper right 6 was irregular and the boundary was blurred. The diagnosis of the tooth causing the presenting symptom is acute wisdom tooth pericoronitis, which is the source tooth of the gingival fistula and should be done ( )

A. Fistula probing

B. Periodontal pocket probing

C. X-rays

D. Fistula diagnostic silk x-ray

E. Periodontal pocket diagnostic silk X-ray

10.2000U2AN136 male, 14 years old, maxillary gums often bleed pus for many days. Deep caries was examined, no perforation of the medullary hole was detected, loose II. degree, percussion (±), temperature measurement was unresponsive, and there was a fistula at the apex of the labial side of the affected tooth. To confirm the diagnosis, the tests that need to be done are ( )

A. Electrical vitality test

B. Smear test

C. Fistula examination

D. X-rays

E. Puncture

11.2000U2AN143 male, 36 years old. 2 weeks ago, I found that there was a small bag in the gums of the right lower back teeth, and there was no obvious discomfort at ordinary times. Examination showed deep caries on the occlusal surface of the right lower first molar, no sensation through the medullary foramen, percussion (±), and a fistula opening in the gingiva at the root apex of the right lower second molar, and a little pus was squeezed out. X-rays showed that the transmission area of the proximal apical apex of the right lower first molar was irregular and the boundary was blurred. The presenting tooth should be diagnosed as ( )

A. Chronic pulpitis

B. Chronic alveolar abscess

C. Apical cyst

D. Apical granulomas

E. Chronic periodontitis

12.2000U2AN144 male, 36 years old. 2 weeks ago, I found that there was a small bag in the gums of the right lower back teeth, and there was no obvious discomfort at ordinary times. Examination showed deep caries on the occlusal surface of the right lower first molar, no sensation through the medullary foramen, percussion (±), and a fistula opening in the gingiva at the root apex of the right lower second molar, and a little pus was squeezed out. X-rays showed that the transmission area of the proximal apical apex of the right lower first molar was irregular and the boundary was blurred. To confirm the diagnosis of a tooth with gingival fistula, ( )

A. Fistula probing

B. X-rays

C. Periodontal pocket probing

D. Fistula diagnostic silk x-ray

E. Periodontal pocket diagnostic silk X-ray

13.2012U2A1064 The following diseases need to be diagnosed by X-ray examination, but excluding ( )

A. Acute apical periodontitis

B. Adjacent caries and secondary caries

C. Intradental and external resorption

D. Chronic apical periodontitis

E. Myeloliths and diffuse calcification

14.2013U2AN145 male, 35 years old. Due to the swelling and pain of the lower right back tooth for 5 days, I came to the doctor today with general discomfort. The patient's painful face was examined, and the right cheek was swollen more obviously. The distal neck caries of the lower right first premolar is deeply perforated with medulla, no probing pain, third-degree loosening, percussion pain (+++), gingival redness and swelling are obvious, and the transition groove is flattened. Tests that should also be done for the patient are as follows, with the exception of ( )

A. Take your temperature

B. Pulp temperature test

C. Check the white blood cell count

D. X-rays

E. Palpation of submandibular lymph nodes

1. E. Chronic apical periodontitis
2. C. Pulp viability
3. B. Acute periapical abscess
4. E. Breakthrough of the nasal floor mucosa to drain pus
5. C. X-rays
6. E. Chronic apical periodontitis
7. C. Perforating the bone wall and breaking through the mucosa
8. C. Regional lymphadenopathy is swollen and tender
9. C. X-rays
10. D. Fistula diagnostic silk x-ray
11. B. Chronic alveolar abscess
12. B. X-rays
13. B. Adjacent caries and secondary caries
14. C. Check the white blood cell count

**Chapter 18 Treatment plan for periapical endodontic disease**

1.2015U2A2127 Female, 60 years old. Left upper back teeth. Severe spontaneous pain for 2 days. There is release pain. Percussion (+), probing of deep cavities, perforation of medullary holes, probing pain (+++). No loosening, periodontal examination (-). The best emergency management is ( )

A. Palliative treatment

B. Taking anti-inflammatory painkillers

C. Pulp opening and drainage

D. Acupuncture analgesia

E. Incision to drain pus

2. 2015U2AN139 A 55-year-old male patient presented with a painful face, complaining of severe pain in the right upper back tooth and not daring to bite and touch the pain. It was found that the gingiva in the upper right 6 was swollen relative to the apical area, the transitional sulcus became shallow, and there was a deep fluctuation sensation on palpation. The upper right 6 percussion (+++) is loose III. degree, which can probe the pulp cavity, and there is no response, the upper right 7 teeth are intact, and the buccal side can detect the root bifurcation, which cannot be penetrated, and percussion (++) is loose III. degree. The right cheek is swollen and tender to the touch. then it should be dealt with first as ( )

A. Intravenous antibiotics

B. Incision to drain pus

C. Removal of decayed tissue

D. Adjust the occlusion

E. A+B

3. 2013U2A2068 patient, male, 24 years old, complained of cold and hot irritation pain in the right lower back tooth for 3 days at night. On examination, the lower right third molar is impacted in the near middle and low position, there is more food impaction between the lower right 78, the lower right 8 does not detect caries, and the distal blind pocket is slightly red and swollen, and it is painful to touch. Cold (-). Detection of caries in the distal neck of the lower right 7 is profound, deep, soft, and cold stimulation causes severe pain. The best treatment for the first visit is ( )

A. Remove the lower right 8

B. Circumcoronal irrigation of the lower right 8 crowns

C. Lower right 7 open and drainage

D. Lower right 7 after derotting ZOE appeased

E. Intravenous broad-spectrum antibiotics

4.2003U2A1011 Seal deactivator should be noted ( )

A. Do not push the inactivator when temporarily sealing the cavity

B. Inactivator is placed in the perforamen

C. Cavities located under the gums can be sealed in layers

D. Attention should be paid to the dryness of the cavity environment before sealing the medicine near the gums

E. Attention should be paid to all of the above

5.2004U2A1073 Pulp inactivation is generally used ( )

A. Paraformaldehyde inactivator

B. Metal arsenic inactivators

C. Arsenite inactivator

D. A or B

E. A, B, C are acceptable

6.2016U2A1026 Which of the following is NOT the principle of emergency treatment of acute apical periodontitis ( )

A. Anti-inflammatory and analgesic

B. Palliative treatment

C. Adjust the teeth and relieve pain

D. Extraction of the affected tooth

E. Establish drainage

7.2016U2A1042 The most effective emergency treatment for acute pulpitis is ( )

A. Anti-inflammatory and analgesic

B. Palliative treatment

C. Pulp opening and drainage

D. Adjustment and abrasion

E. Acupuncture analgesia

8.2016U2A1052 Emergency treatment of acute apical periodontitis is correct ( )

A. Under local anesthesia

B. Open the medullary cavity and drain the root canal

C. Perforation through the apical hole

D. Incision and drainage of abscess if there is an abscess

E. All of the above

9.2009U2A1088 In the treatment of acute pulpitis, the first question that should be considered is ( )

A. Anti-inflammatory

B. Destroy pathogenic bacteria

C. Painless or minimized

D. Prevent the spread of infection

E. Protect the living pulp

10.2009U2A1091 The most effective treatment for submucosal abscess in acute apical inflammation is ( )

A. Take anti-inflammatory painkillers

B. Drainage

C. Incision to drain pus

D. Adjustment and abrasion

E. Acupuncture for pain relief

11. 2008U2A1052 Which point is wrong about the treatment of endodontic disease and periapical disease ( )

A. During root canal treatment, it is not advisable to seal the medicine repeatedly

B. Acute pulpitis with severe pain Oral painkillers are difficult to work

C. Calcium hydroxide preparations can also be used for root canal disinfection

D. There is no need to wait until all symptoms are gone before doing a root canal filling

E. Endodontic disease and periapical disease are mostly caused by bacterial infection, so aseptic procedures can be de-emphasized in treatment

12.2000U2AN142 male, 40 years old. In the past six months, the left upper back tooth has encountered hot and cold pain, but there is no spontaneous pain, and in the past 1 month, in addition to cold and heat allergies, there is a dull pain. Spontaneous pain in the past 1 week, worsening paroxysmal episodes, nocturnal pain. Examination revealed that the left upper second molar had deep caries on the mesial occlusal surface, and the pain was evident and percussion (±). The effective treatment of the presenting tooth is ( )

A. Anti-inflammatory and analgesic

B. Topical application

C. Caries cavity filling

D. Endodontic treatment

Periodontal treatment

13.2013U2A2131 A 25-year-old patient with poor oral hygiene, the first permanent molar of the left lower left had a pulp-periodontal joint lesion, and the pain was severe, but the affected tooth was not loosened. The first measures to be taken to eliminate the pain and restore health ( )

A. Early filling

B. Open and drain to stop the spread of inflammation

C. Anti-inflammatory and analgesic

D. Promote periodontal soft tissue health

Use of fluoride

14.2012U2A1066 Pulp Plasticization Treatment, the requirements for pulp extraction are ( )

A. Retain 1/3 of the pulp at the apex

B. Extract the pulp as much as possible

C. Puncture the apical hole

D. The pulp can be removed without extraction

E. Retain the pulp at the apical 2/3

1. C. Pulp opening and drainage
2. B. Incision to drain pus
3. C. Lower right 7 open and drainage
4. E. Attention should be paid to all of the above
5. E. A, B, C are acceptable
6. D. Extraction of the affected tooth
7. C. Pulp opening and drainage
8. E. All of the above
9. B. Destroy pathogenic bacteria
10. B. Drainage
11. E. Endodontic disease and periapical disease are mostly caused by bacterial infection, so aseptic procedures can be de-emphasized in treatment
12. D. Endodontic treatment
13. B. Open and drain to stop the spread of inflammation
14. B. Extract the pulp as much as possible

**Chapter 19 Root Canal Therapy**

1.2017U2A1015 The following statements about root canal treatment are incorrect ( )

A. X-rays were taken before surgery to understand periapical lesions and root resorption

B. Remove the entire pulp under local anesthesia or after the pulp is inactivated

C. It is suitable for the affected tooth whose pulp inflammation involves the root pulp and is not suitable for pulpectomy

D. Remove the necrotic pulp tissue in the pulp chamber and root canal, and file the root canal appropriately

E. Rinse and sterilize the root canal thoroughly, using 5% hydrogen peroxide solution

2.2016U2A1055 Which treatment is best for juvenile apical periodontitis ( )

A. Dry myeloid surgery

B. Root canal treatment

C. Plastinization therapy

D. ATC therapy

E. Palliative treatment

3. 2000U2AN137 male, 14 years old, maxillary gums often bleed pus for many days. Deep caries was examined, no perforation of the medullary hole was detected, pine II.°, percussion (±), temperature measurement was unresponsive, and there was a fistula at the root apex of the labial side of the affected tooth. Clinical treatment should be used ( )

A. Cutting of the living myel

B. Plastination therapy

C. Open drainage

D. Root canal treatment

E. Dry myeloid surgery

4. 2000U2AN140 male, 60 years old, has a right upper posterior toothache for one year, which mostly occurs in the evening and involves the right lower orbit and temporal region. The patient had a history of headache and runny nose several years ago. Check: deep caries on the upper right 5 distal occlusal surfaces and 6 upper right occlusal surfaces, percussion pain (+), palpital pain (-), upper right 7 and upper right 4 crowns intact, percussion pain (-), upper right 8 stubs. The most important thing in the treatment design of the chief complaint disease is ( )

A. Bottom filling

B. Root canal treatment

C. Endomedulla therapy

D. Root uprooting

E. ENT visits

5. 2000U2AN145 male, 36 years old. 2 weeks ago, I found that there was a small bag in the gums of the right lower back teeth, and there was no obvious discomfort at ordinary times. Examination showed deep caries on the occlusal surface of the right lower first molar, no sensation through the medullary foramen, percussion (±), and a fistula opening in the gingiva at the root apex of the right lower second molar, and a little pus was squeezed out. X-rays showed that the transmission area of the proximal apical apex of the right lower first molar was irregular and the boundary was blurred. The treatment of the complaining tooth is ( )

A. Filling therapy

B. Plastination therapy

C. Root canal treatment

D. Apical surgery

E. Uprooting

6.2013U2A2126 The patient did not dare to bite due to wedge-shaped defect, after being filled with composite resin, the cold and heat stimulated the pain. Check: 456 buccal composite resin filling on the upper left, 46 cold pain on the upper left, which can be relieved after removal, 5 percussions (+) on the upper left, obvious pain in hot and cold measurement, and lasting for a period of time after removal. The principle of handling the upper left 5 should be ( )

A. No treatment, observation

B. Desensitization therapy

C. Remove the old backfill and refill it

D. Remove the old filler, soothe with zinc oxide clove oil paste

E. Endodontic treatment

7. 2013U2A2129 The patient presented with cold and hot pain in the left tooth. Check: 6 severe uneven wear in the lower left, no perforation of the pulp hole, pain caused by thermal measurement and lasted for 2 minutes, percussion (-), the treatment principle of the affected tooth is ( )

A. Ask the patient to eat a small hard diet

B. Desensitization

C. Adjust the occlusion

D. Endodontic treatment

E. Full crown restoration

8.2014U2A1043 Which of the indications for root canal treatment is not exact ( )

A. Pulpitis of all kinds

B. Pulp necrosis

C. Various types of apical periodontitis

D. Apical periodontitis of deciduous and permanent teeth

E. Pulp periodontal disease

9.2014U2A1044 Observation time of postoperative efficacy evaluation of root canal therapy is ( )

A. Half a year

B. 1 year

C. 2 years

D. 3~5 years

E. More than 5 years

10.2003U2A1012 In root canal treatment, the instruments used for rotation should not be rotated more than ( )

A. 45°

B. 90°

C. 180°

D. 270°

E. 360°

11.2011U2A2081 Root canal treatment but periapical lesions are worsening, which of the following bacteria should be considered ( )

A. Actinomycetes

B. Fusobacterium

C. Peptic streptococcus

D. Melanin-producing Pruvotea

E. eubacterium

1. E. Rinse and sterilize the root canal thoroughly, using 5% hydrogen peroxide solution
2. B. Root canal treatment
3. D. Root canal treatment
4. B. Root canal treatment
5. C. Root canal treatment
6. D. Remove the old filler, soothe with zinc oxide clove oil paste
7. E. Full crown restoration
8. E. Pulp periodontal disease
9. C. 2 years
10. E. 360°
11. A. Actinomycetes

**Chapter 21 Root Canal Preparation and Disinfection**

1.2010U2A1087 The statement about calcium hydroxide preparations is incorrect ( )

A. It is strongly alkaline

B. Has anti-inflammatory and antibacterial properties

C. Promote restorative dentin formation

D. Conducive to the recovery of pulp function

E. Insoluble in saliva, the solubility is the smallest among the substrate materials

2.2003U2A1014 Which of the following statements is incorrect about H file ( )

A. The cross-section is comma-shaped

B. Suitable for loose root canal preparation

C. The cutting edge is nearly perpendicular to the root canal wall

D. Rotation and lifting movements can be used during operation

E. Easier to break

3.2016U2A1046 In general, the main file should be larger than the primary file ( )

A. 1 device number

B. 3 device numbers

C. 4 instrument numbers

D. 5 instrument numbers

E. Arbitrary

4.2004U2A1078 Root surface chemical treatment, the most commonly used drug is ( )

A. 37% phosphoric acid

B. 10% citrate

C. 20% phosphoric acid

D. 10% acetic acid

E. 10% hydrochloric acid

5. 2010U2A1078 Which is wrong about root canal preparation ( )

A. Use reaming files and reaming drills, from fine to coarse, in order of serial number

B. The root canal mouth can be enlarged by ball drill, large reamer drill or crack drill

C. Root canal stenosis, calcification, or foreign bodies in the root canal can be treated with the chelating agent ethylenediaminetetraacetic acid

D. All instruments used for rotation should not be rotated more than half a turn

E. A+C+D

6. 2011U2A1034 The following statements about root canal irrigation fluid are incorrect ( )

A. The alternating use of 5.25% sodium hypochlorite and 3% hydrogen peroxide can produce new ecological oxygen and have foaming effect

B. Sodium hypochlorite is a good solvent for necrotic tissue

C. EDTA is effective for enlarging, hardening, or bending root canals

D. Fluids can provide lubrication

E. Sodium hypochlorite solution is selected for root canal irrigation with root hypoplasia

7. 2011U2A1035 The most commonly used root canal disinfection method in clinical practice is ( )

A. Laser treatment

B. Microwave therapy

C. High-frequency therapy

D. Electrolysis therapy

E. Drug disinfection

8.2002U2A1079 The depth of root canal preparation should stop at the cementum boundary of dentin, usually about ( ) from the apex

A. 0.5mm

B. 1mm

C. 1.5mm

D. 2mm

E. 1/3 of the apex

9.2006U2A1043 With the increase of age, the working length of the root canal continues ( )

A. Reduction

B. Additions

C. No change

D. Can be reduced, can be increased

E. All of the above are possible

10.2000U2A1030 The purpose of mechanical preparation of root canal is as follows, except ( )

A. Removal of infection in the main root canal

B. Removal of infection from the root canal wall

C. Enlarge the apical foramen to facilitate drainage

D. Reduce the curvature of the bent root canal

E. Prepare root canal morphology to facilitate filling

1. **Statement about calcium hydroxide preparation**: The correct statement is that calcium hydroxide is strongly alkaline, has anti-inflammatory and antibacterial properties, can promote the formation of restorative dentin, and is conducive to the recovery of pulp function. Calcium hydroxide is insoluble in saliva, but its solubility is not the smallest of the base materials, so option **E** is incorrect.
2. **Statement about H file**: H file has a comma cross-section and is suitable for the preparation of loose root canals, with the cutting edge nearly perpendicular to the root canal wall. Although the H file can be operated with rotating and lifting movements, it is easier to break. Therefore, **option D**, "Rotate and lift while operating" is not entirely correct, because while it is technically possible to rotate and lift, it increases the risk of breakage.
3. **In general, the main file should be larger than the primary file**: the main file is usually 1 device size larger than the primary file, so the correct answer is **A**.
4. **The most commonly used drug for root surface chemical treatment**: The most commonly used drug is 37% phosphoric acid, so the correct answer is **A**.
5. **Statement about root canal preparation**: The wrong statement may be **E**, because A, B, and C are all correct methods of root canal preparation, and the rotation mentioned by D is also a safety tip.
6. **What is said about root canal irrigation fluid**: EDTA is correct for enlarging hardened or bending root canals, but not all liquids provide lubrication, so **option D** "All liquids provide lubrication" is incorrect.
7. **The most commonly used method of root canal disinfection in clinical** practice: the most commonly used is drug disinfection, so the correct answer is **E**.
8. **The depth of root canal preparation**: It should stop at the cementum boundary of dentin, usually about 0.5 mm to 1 mm from the root apex, so the answer may be **A** or **B**, depending on the literature or textbook recommendation.
9. **Root canal working length as it ages**: As it ages, root canals may become shorter due to tooth wear, so the answer is **A** decreases.
10. **Purpose of mechanical preparation of root canals**: Reducing the curvature of curved root canals is not the purpose of mechanical preparation of root canals, so **D** is an exception.

**Chapter 27 Medical History Collection and Clinical Examination**

1.2013U2A2116 Male, 50 years old, 1 week old right back tooth bite discomfort, cold water caused pain. In the past 4 days, night pain has affected sleep and caused pain in half of the head and face, and the pain cannot be located. On examination, it was found that both the upper and lower first molars on the right side had occlusal cavities. If the jaw position of the affected tooth cannot be determined after examination, ( )

A. Bite

B. Anesthetic test

C. Temperature test

D. Periodontal pocket probing

E. X-rays

2.2016U2A1029 The temperature of the thermal stimulus should be higher than ( )

A. 20℃

B. 40℃

C. 30℃

D. 60℃

E. 50℃

3. 2009U2A1082 When diagnosis of caries, the result of the normal tooth surface temperature test is ( )

A. Normal

B. Transient sensitivity

C. Sensitive

D. Sluggishness

E. No Response

4.2009U2A1090 The most reliable examination method for judging pulp viability is ( )

A. Temperature test

B. Electrical test

C. Selective anesthesia

D. Experimental cave preparation

E. X-rays

5.2008U2A1038 The following contraindications to the electroviability test of the pulp are ( )

A. Hyperthyroidism

B. Coronary heart disease

C. High blood pressure

D. Pacemakers

Epilepsy

6.2008U2A1045 Causes of false negative reaction of electron pulp viability test, excluding ( )

A. Use of anesthetic prior to testing

B. Newly erupted tooth whose root tip is not fully developed

C. Teeth that have undergone pulptomy

D. Teeth with excessive calcification in the root canal

E. Teeth that have just been traumatized

7.2008U2A1057 Electro-endodontic viability test is commonly used in diagnosis ( )

A. Calcification of the pulp

B. Inflammation of the pulp

C. Acute apical periodontitis

D. Pulp necrosis

E. Chronic apical periodontitis

8.2012U2A1063 Precautions for tooth temperature test are as follows, except ( )

A. Measure the affected tooth first

B. Thermal temperature is above 60°C

C. Isolate saliva

D. Xiaoice sticks for cold measurement

E. Test in the middle 1/3 of the labial (buccal) or tongue (palatal) surface of the teeth

9. 2012U2A1078 The cause of the false positive reaction of the electrododontic viability test is correct ( )

A. The probe is in contact with a large area of metal restoration

B. The test tooth is not adequately insulated from moisture or dryness

C. The pulp has liquefaction necrosis

D. Patient is overly nervous and anxious

E. All of the above

10.2013U2A1022 False reaction may occur in the electroviability test of the pulp, and the cause of the false positive reaction does not include which of the following ( )

A. The probe or electrode touches a large area of metal restoration or gum, allowing the current to flow to the periodontal tissue.

B. Inadequate moisture isolation or drying of the test tooth to leak into the periodontal tissue.

C. Liquefied necrotic pulp has the potential to conduct an electric current around the apex, and the patient may have a slight reaction when the current is adjusted to the maximum scale

D. The patient is so nervous and anxious that he or she responds when the probe first touches the tooth surface or when asked about his feelings.

E. The patient has used analgesics, anesthetics, or alcoholic beverages beforehand and cannot perceive electrical stimuli normally.

These questions look like specialized test questions in the field of dentistry that involve knowledge of dental health and pulp viability testing. Here's the answer to each question:

1. If the jaw position of the affected tooth cannot be determined after examination, E. X-rays. X-rays can help determine the exact problem and location of the teeth.
2. The temperature of the thermal stimulus should be higher than that of B. 40℃。 This is because higher temperatures are more likely to cause tooth sensitivity or painful reactions, which are used to test pulp reactions.
3. When tooth decay is diagnosed, the results of the normal tooth surface temperature test are A. Normal. This is because tooth decay usually responds normally to heat and cold, unless the caries has affected the pulp.
4. The most reliable test to determine pulp viability is B. Electrical quiz. The electro-test can accurately assess the health status of the pulp.
5. The following are contraindications to the electrododontic viability test**: D. Pacemakers**. Electrical testing can interfere with the function of the pacemaker.
6. Causes of false negative electron reaction on pulp viability test do not include **E. Teeth that have just undergone trauma.** Trauma does not directly affect the results of the electrical test.
7. Electro-pulp viability testing is often used to diagnose **D. pulp necrosis**. An electrical test can help determine if the pulp is still viable.
8. The precautions for the tooth temperature test are as follows, except B. The thermal temperature is above 60°C. Such a high temperature is not usually used for temperature tests to avoid injury.
9. The cause of the false positive reaction on the electron viability test of the pulp is correct**: E.** All of these factors can lead to false positives.
10. False reactions may occur in pulp viability tests, and the cause of false positive reactions does not include any of the followingE**. The patient has used analgesics, anesthetics, or alcoholic beverages beforehand and cannot perceive electrical stimuli normally**. This condition is more likely to lead to a false-negative reaction.

**Chapter 29 Endodontics Internship Course**

1.2020U1A1058 The materials commonly used in non-traumatic repair therapy are ( )

A. Glass ions

B. Composite resins

C. New composite resins

D. Amalgam

E. Pit and fissure sealants

2.2015U2A2107 Yao, male, 2 years old and 7 months, parents found that the child's upper anterior teeth were "blackened" and asked for treatment, and the left and right upper I. proximal and median adjacent surface were examined for superficial caries. Treatment is generally used ( )

A. Medication

B. Filling therapy

C. Restorative treatment

D. Root canal treatment

E. Observation and follow-up

3.2008U2A1035 Clinical manifestations of periodontal lesions caused by apical periodontitis ( )

A. Pulp no viability or abnormal viability

B. Periodontal lesions are confined to one tooth

C. X-ray shows that the periapical destruction zone is flask-like

D. Often accompanied by dental lesions

E. All of the above

4.2008U2A1037 Which of the following pulp lesions may cause periodontal disease ( )

A. Acute serous pulpitis

B. Acute suppurative pulpitis

C. Chronic pulpitis

D. Acute periapical abscess

E. Residual myelitis

5. The pathological changes of 2018U2A1007 acute serous apical periodontitis are ( )

A. Minimal neutrophil infiltration

B. Alveolar bone hyperplasia at the apex

C. Squamous epithelial cord of hyperplasia within the lesion

D. Formation of a large amount of granulation tissue in the lesion

E. Apical cementum resorption

6.2017U3A1004 The total daily saliva of normal people is 1000~1500ml, of which the secretion of parotid gland and submandibular gland accounts for ( )

A. 50%

B. 60%

C. 70%

D. 80%

E. 90%

7. 2014U3A2074 Hypertensive patient, male 65 years old, maxillary right 67 stump, no history of heart disease, need to extract teeth, the nerve that should be anesthetized during extraction is ( )

A. Infraorbital nerve + posterior palatine nerve

B. Superior antalveolar nerve + superior postalveolar nerve + anterior palatine nerve

C. Superior posterior alveolar nerve + superior middle alveolar nerve + nasopalatine nerve

D. Superior posterior alveolar nerve + superior middle alveolar nerve + anterior palatine nerve

E. Superior postalveolar nerve + anterior palatine nerve

8. 2014U3AN143 female, 22 years old, requested extraction due to low ambush impaction of right lower wisdom tooth. In order to reduce bleeding and maintain a clear surgical field, epinephrine is often added to the anesthetic, and the nerve that should be anesthetized to extract the tooth is the inferior alveolar nerve + buccal nerve + lingual nerve on the same side.

A. Anesthesia of the inferior alveolar nerve

B. Anesthetized masticatory nerve

C. Buccal nerve anesthetized

D. Anesthetized the facial nerve

E. Oral hormones

9.2019U3A1003 The nerve anesthetized for maxillary first molars extraction is ( )

A. Nasopalatine nerve, anterior palatine nerve, posterior palatine nerve

B. Superior postalveolar nerve, superior alveolar middle nerve, anterior palatine nerve

C. Superior posterior alveolar nerve, superior middle alveolar nerve, nasopalatine nerve

D. Superior postalveolar nerve, superior alveolar middle nerve, superior antalveolar nerve

E. Superior alveolar middle nerve, nasopalatine nerve, anterior palatine nerve

10.2018U3A2111 Male, 2 years old. For cleft lip repair, an infraorbital nerve block anesthesia is added, and the needle insertion point is ( )

A. 1.5 cm below the midpoint of the infraorbital margin, the midpoint of the line from the alar of the nose to the tragus

B. 0.5 cm below the midpoint of the inferior orbital margin, from the tip of the nose to the midpoint of the tragus

C. 1.5 cm below the midpoint of the inferior orbital margin, the midpoint of the nasal alar to the lateral eyelid line

D. 0.5 cm below the midpoint of the inferior orbital margin, from the tip of the nose to the midpoint of the lateral eyelid line

E. 0.5 cm below the midpoint of the inferior orbital margin, the midpoint of the nasal alar to the lateral eyelid line

11.2003U3AN103 "Oral Interpretation" male, 45 years old. He complained of discomfort in his left mandibular posterior teeth for 6 months. Oral examination: 56 residual crown in the lower left, percussion (±), loose (-), endodontic treatment has been done, the gums are slightly red, can not be repaired, and are to be extracted. There were no obvious abnormalities in the other teeth in the mouth. X-rays showed that there was a shadow at the apex of the distal root in the lower left 6, which was close to the mandibular neural canal. The lower left 56 removed nerves that do not require anesthesia are ( )

A. Inferior alveolar nerve

B. Tongue nerve

C. Masseter nerve

D. Buccal nerve

E. All of the above nerves require anesthesia

12. 2013U3A2109 patient, a 24-year-old male, complained of hot and cold irritation pain in the right lower back tooth for 3 days at night. On examination, the lower right third molar is impacted in the near middle and low position, there is more food impaction between the lower right 78, the lower right 8 does not detect caries, and the distal blind pocket is slightly red and swollen, and it is painful to touch. Cold (-). Detection of caries in the distal neck of the lower right 7 is profound, deep, soft, and cold stimulation causes severe pain. Probable diagnosis of presenting tooth ( )

A. Hypo8 coronitis in the lower right

B. Acute apical inflammation in the lower right 7

C. Right lower 78 periodontitis

D. Acute pulpitis in the lower right 7

E. Near-mid-low impaction in the lower right 8

1. B. Composite resins
2. B. Filling therapy
3. E. All of the above
4. B. Acute suppurative pulpitis
5. A. Minimal neutrophil infiltration
6. B. 60%
7. D. Superior posterior alveolar nerve + superior middle alveolar nerve + anterior palatine nerve
8. D. Anesthetized the facial nerve
9. B. Superior postalveolar nerve, superior alveolar middle nerve, anterior palatine nerve
10. E. 0.5 cm below the midpoint of the inferior orbital margin, the midpoint of the nasal alar to the lateral eyelid line
11. E. All of the above nerves require anesthesia
12. D. Acute pulpitis in the lower right 7

**Periodontology**

**Chapter 2 Applied Anatomy and Physiology of Periodontal Tissue**

1.2008U2A1022 The epithelium of the inner wall of the gingival sulcus is ( )

A. Non-keratinized stratified squamous epithelium

B. Keratinized stratified squamous epithelium

C. Infully keratinized stratified epithelium

D. Laminated columnar epithelium

E. Laminated epithelium without spikes

2.2008U2A1025 The distance from the normal alveolar crest to the enamel cementum boundary is ( )

A. 1~2mm, no more than 2.5mm

B. 1~1.5mm, not more than 2mm

C. 1.5~2mm, not more than 3mm

D. 2~3mm, not more than 3mm

E. 0.5~2.5mm, not more than 3mm

3.2004U2A1057 Factors to keep the tooth position normal and undisplaced include ( )

A. Adequate periodontal support tissue

B. Dental force and labial-buccal-tongue muscle strength

C. Integrity of dentition

D. Normal tooth morphology and adjacency

E. All of the above

4.2005U2A1017 The tooth position that is least prone to periodontal destruction is ( )

A. Maxillary incisors and mandibular molars

B. Mandibular incisors and maxillary molars

C. Maxillary canines and mandibular premolars

D. Mandibular canines and maxillary premolars

E. Maxillary and mandibular premolars

5.2013U2A1008 How many mm is the distance from the alveolar crest to the enamel cementum boundary on X-ray that exceeds the alveolar bone resorption ( )

A. 1mm

B. 2mm

C. 3mm

D. 4mm

E. 5mm

6.2013U2A1011 How much gingival sulcus depth exceeds should be recorded ( )

A. 1mm

B. 2mm

C. 2.5mm

D. 3mm

E. 3.5mm

7. 2011U2A1007 The width of the periodontal ligament is generally ( )

A. 0．5～1mm

B. 0.15～0.38mm

C. 1．2～2mm

D. 0.5～0.1mm

E. 1～2mm

8. 2007U2A1001 Gull of the gum is the first site of periodontal disease because ( )

A. Loose intercellular connections in the epithelium

B. The epithelium is keratinized and non-self-cleaning

C. The epithelium is awholly keratinized and the epithelial thickness is thin

D. There is no connective tissue in the subepithelial lamina propria at the site

E. The area is prone to food impaction, resulting in bacterial retention and multiplication

9.2007U2A1002 Which of the following structures is the biological width composed of ( )

A. Connective tissue combining the width of the crown root of the epithelium + the crown square of the bony crest

B. Width of the crown-roots direction of the combined epithelium + depth of the gingival sulcus

C. Connective tissue combining epithelial width + gingival sulcus depth + bony crest crown square

D. Depth of probing + connective tissue of the crown of the bony crest

E. Probing depth minus gingival sulcus depth

10.2005U2A1019 The thickness of the periodontal ligament is different in different people and different teeth, generally ( )

A. 0.01～0.38mm

B. 0.15～0.38mm

C. 0.05～0.38mm

D. 0.10～0.38mm

E. 0.20～0.38mm

1. Type of epithelial lining of the gingival sulcus
   - Answer: A. Non-keratinized stratified squamous epithelium
2. The distance from the normal alveolar crest to the cementum boundary of the enamel
   - Answer: A. 1~2mm, no more than 2.5mm
3. Factors that keep the tooth position normal and unshifted
   - Answer: E. All of the above
4. Teeth that are least susceptible to periodontal destruction
   - Answer: C. Maxillary canines and mandibular premolars
5. The distance from the alveolar crest to the enamel cementum boundary on x-rays is considered to be the standard for alveolar bone resorption
   - Answer: B. 2mm
6. How much more than the gingival sulcus depth should be recorded
   - Answer: C. 2.5mm
7. The width of the periodontal ligament is generally as follows:
   - Answer: E. 1~2mm
8. The gingival valley is the reason why periodontal disease originates
   - Answer: B. The epithelium is keratinized and non-self-cleaning
9. What structures are made up of biological widths
   - Answer: A. Connective tissue combining the width of the crown root of the epithelium + the crown square of the bony crest
10. The thickness of the periodontal ligament is generally as follows:
    - Answer: B. 0.15~0.38mm

**Chapter 4 Microbiology of Periodontal Disease**

1.2009U2A1051 Correlation between bacteria in plaque ( )

A. Symbiosis

B. Antagonism

C. Competition

D. Symbiosis, antagonism, and competitive coexistence

E. Varies from person to person

2.2009U2A1068 The following table shows the characteristics of subgingival plaque, pointing out the wrong one of the attached plaques and non-attached plaques ( )

A. Does not extend to the bound epithelium, extends to the bound epithelium

B. No contact with the sulcus epithelium, contact with the sulcus epithelium

C. Permeable epithelium and connective and may be connected to supragingival plaque

D. G+ bacteria are dominant, and Gˉ active bacteria are dominant

E. Associated with calculus formation root caries, related to periodontal tissue inflammation

3. 2009U2A1076 Supragingival plaque is correctly defined ( )

A. Unmineralized bacterial sediments, firmly adhered to tooth surfaces and restoration surfaces, composed of bacteria

B. Unmineralized bacterial sediment, firmly adhered to the tooth surface and the surface of the restoration, consisting of a sticky matrix and bacteria embedded in it

C. Partially mineralized bacterial sediment, firmly adhered to the tooth surface and the surface of the restoration, consisting of a sticky matrix and bacteria embedded in it

D. Mineralized bacterial sediments, firmly adhered to tooth surfaces and restorations, consisting of a sticky matrix and bacteria embedded in them

E. None of the above

4.2009U2A1078 Periodontal bacteria lead to the occurrence of periodontal destruction, and the mechanism is ( )

A. Direct invasion of bacteria into the periodontal tissues

B. The bacteria directly invade the periodontal tissues, causing an immune response

C. Bacteria multiply in the body

D. Inhibition and evasion of the host's defense function

E. All of the above

5. 2009U2A1080 The most important bacteria that cause gingivitis are ( )

A. Gram-positive bacilli

B. Gram-negative bacilli

C. Spirochetes

D. Chlamydia

E. Actinomycetes viscosus

6. 2016U2A1020 Microorganisms implicated in the occurrence of periodontal disease, the most well-documented are ( )

A. With Actinobacillus actinomyceter, Prevotella intermediates, and Fusobacterium nucleata

B. Porphyromonas gingivals, Prevotella intermediates, Trephelum donticula

C. With Actinobacterium, Porphyromonas gingivalis and Pseudobacteroides fersa

D. Pruzewala intermediates, Pseudobacteroides fersa, Treponebacterium dentensis

E. with Actinobacillus, Pseudobacteroides fersia, Fusobacterium nucleatus

7. 2016U2A1023 The bacterial components that are not related to periodontal destruction are ( )

A. Bacterial surface material

B. Gliase and hyaluronidase secreted by bacteria

C. Bacterial toxins

D. Bacterial metabolites

E. Bacterial size and morphology

8.2008U2A1023 Which of the following descriptions is incorrect about non-adherent subgingival plaque ( )

A. Under certain conditions, the amount of non-adherent subgingival plaque is greatly increased

B. Its composition is dominated by anaerobic bacteria

C. Closely related to the destruction of alveolar bone

D. Causes root caries

E. Loose structure, direct contact with the pouch epithelium and gingival sulcus

9.2013U2A1014 Which of the following suspected periodontal pathogens is associated with recurrence or further exacerbation of periodontitis after treatment ( )

A. Meprevotesa

B. Fersaibacter

C. Fusobacterium nucleatus

D. Porphyromonas gingivals

E. Actinobacillus actinomyceter

10.2011U2A1008 Which of the following bacterial surface substances is the most toxic to periodontal tissues ( )

A. Lipoteichoic acid

B. Outer membrane proteins

C. Endotoxin

D. Ciliary proteins

E. Membrane vesicles

11. 2010U2A1049 The main basis of the non-specific plaque theory ( )

A. Plaque extract can cause subcutaneous abscess in animals

B. If there is a lot of plaque and tartar, the gum inflammation is more severe

C. Removal of plaque and tartar can reduce inflammation

D. Plaque overall microbial co-pathogenicity

E. All of the above

12.2007U2A1003 Subgingival non-attached plaque is mainly composed of the following bacteria ( )

A. Gram-positive cocci, bacilli, and filamentous bacteria

B. Gram-negative anaerobes, actine bacteria

C. Gram-positive facultative and anaerobic bacteria

D. Gram-positive aerobic and facultative bacteria

E. Gram-positive aerobic and gram-negative anaerobes

13.2007U2A1004 Main points of the specific plaque theory ( )

A. Periodontal disease occurs as a result of mixed infection of the oral flora

B. Periodontal destruction is due to an increase in the number of bacteria and increased virulence

C. Periodontal destruction of different types and sites is associated with specific bacteria

D. Periodontal destruction is due to low defenses at the lesion site

E. All of the above

Chapter 4 is titled with a focus on periodontal microbiology:

1. Interrelationships between bacteria within plaque
   - Answer: D. Symbiosis, antagonism, and competitive coexistence
2. One of the wrong features of subgingival plaque
   - Answer: D. G+ bacteria are predominant, Gˉ actans are dominant (this description seems to be a mixture of the characteristics of attached and non-attached plaques)
3. Correct definition of supragingival plaque
   - Answer: B. Unmineralized bacterial sediment, firmly adhered to the tooth surface and the surface of the restoration, consisting of a sticky matrix and bacteria embedded in it
4. The mechanism by which periodontal bacteria cause periodontal destruction
   - Answer: E. All of the above
5. The most important bacteria that cause gingivitis
   - Answer: A. Gram-positive bacilli
6. The microorganisms involved in the development of periodontal disease, the most well-documented:
   - Answer: B. Porphyromonas gingivals, Pruchonella intermediates, Trephela donticula
7. Bacterial components not associated with periodontal destruction
   - Answer: E. Bacterial body size and morphology
8. Incorrect description of non-attached subgingival plaque
   - Answer: D. Causes root caries
9. Periodontitis suspected of causing bacteria that recur after treatment or continue to worsen the disease
   - Answer: D. Porphyromonas gingivals
10. The most toxic bacterial surface substance to periodontal tissues
    - Answer: C. Endotoxin
11. The main basis for the non-specific plaque theory
    - Answer: E. All of the above
12. Subgingival non-attached plaque is mainly composed of the following bacteria:
    - Answer: B. Gram-negative anaerobes, acterium
13. The main points of the specific plaque theory
    - Answer: E. All of the above
14. Which of the following is related to the formation of subgingival tartar?
    - Answer: B. Attached subgingival plaque

**Chapter 6 Immune inflammatory responses and promoters in periodontal disease hosts**

1.2008U2A1020 The main components of gingival crevicular fluid are derived from ( )

A. Blood

B. Serum

C. Plasma

D. Adjacent periodontal tissue

E. Bacteria

2.2004U2A1059 Effect of smoking on periodontal tissue, excl. ( )

A. Reduction of local oxygen tension

B. Inhibition of fibroblast growth

C. Inhibition of neutrophil function

D. Not conducive to oral hygiene maintenance

E. Inhibition of collagenase activity

3.2004U2A1063 Effect of gingival crevicular fluid ( )

A. Flush the sulcus with foreign material

B. Has antimicrobial effect

C. Rich in plasma proteins

D. Provide the inorganic components of tartar mineralization

E. All of the above

4. 2010U2A1040 Factors related to the severity of periodontal destruction in diabetic patients are ( )

A. Types of diabetes

B. Degree of metabolic control

C. The length of the duration of diabetes

D. Presence or absence of systemic complications

E. All of the above

5.2015U2A1007 Saliva function ( )

A. Mechanical cleaning

B. Lubrication and protection

C. Buffering

D. Antimicrobial

E. All of the above

6. 2010U2A1042 The possible relationship between diabetes mellitus and periodontal disease is ( )

A. Diabetes mellitus is one of the risk factors for periodontal disease

B. Periodontal disease does not affect diabetes

C. There is a two-way relationship between diabetes and periodontal disease

D. A+B

E. A+C

7.2005U2A1036 Type I diabetic periodontitis is more common in ( )

A. Maxillary incisors

B. Mandibular incisors

C. Upper and mandibular incisors and bicuspids

D. Upper and mandibular incisors and first molars

E. Upper and mandibular incisors and second molars

8.2015U2A1021 Systemic contributing factors for periodontitis include ( )

A. Gastric ulcers

B. Dental deformities

C. Mental stress

D. Hepatitis B

E. Pyelonephritis

9.2017U2A1021 What is incorrect in the narrative about gingival crevicular fluid is ( )

A. Very small amounts of gingival crevicular fluid in healthy gums

B. Significant increase in gingival crevicular fluid during inflammation

C. The liquid component is mainly derived from serum

D. Immunoglobulins in the gingival crevicular fluid have the effect of anti-specific pathogenic bacteria

E. There are no defense cells such as leukocytes in the gingival sulcus fluid

Chapter 6 examines the immune inflammatory response and contributing factors in periodontal disease hosts:

1. The main source of gingival sulcus fluid
   - Answer: A. Blood
2. The effects of smoking on periodontal tissues are not included
   - Answer: E. Inhibition of collagenase activity
3. The role of gingival sulcus fluid
   - Answer: E. All of the above
4. Factors related to the severity of periodontal destruction in diabetic patients
   - Answer: E. All of the above
5. The function of saliva
   - Answer: E. All of the above
6. Possible relationship between diabetes mellitus and periodontal disease
   - Answer: E. A+C
7. Type I: Periodontitis in patients with type I. Tooth position
   - Answer: E. Upper and lower incisors and second molars
8. Systemic contributing factors to periodontitis
   - Answer: C. Mental stress
9. Incorrect statements about crevicular fluid
   - Answer: E. There are no defense cells such as white blood cells in the gingival sulcus fluid

**Chapter 8 Examination and Diagnosis of Periodontal Disease**

1.2009U2A1042 In order to obtain a clinically accurate periodontal pocket depth or attachment level, the key points that should be mastered are ( )

A. Use standardized probes

B. Master the correct probing power

C. Probing direction parallel to the long axis of the tooth

D. Sequential walk-in probing process and stable fulcrum

E. All of the above

2.2009U2A1048 Occlusion examination, wax bite tablets are often used to check ( )

A. Median occlusion

B. Anterior occlusion

C. Lateral occlusion

D. Crossbite

E. All of the above

3.2016U2A1007 Special instruments for clinical examination of periodontal tissue( )

A. Oral scope, probe, forceps

B. Periodontal probe, electrovinometer, thin wax sheet

C. Periodontal probe, teether stick, dental floss, stain

D. Periodontal probe, dental floss, occlusal paper, thin wax sheet

E. Periodontal probes, bite papers, thin wax films, X-rays

4.2016U2A1012 II. Degree loose teeth refer to ( )

A. Vertical loosening

B. Slightly greater than physiological mobility within 1 mm

C. Shake from the normal position to the buccal tongue or mesiodistal direction with a movement greater than 2 mm

D. Shake from the normal position to the buccal tongue or mesiodistal direction, with a movement of 1~2mm

E. Shake from the normal position to the buccal tongue or mesiodistal direction with a movement greater than 3 mm

5.2016U2A1021 Inspection method for accurately judging the presence or absence of periodontal pockets ( )

A. Inspect for swelling of the gums

B. Inspection and inspection of the gum color

C. Occlusion examination

D. Periodontal probe examination

E. X-rays

6.2013U2A1003 Which item is incorrect about the present periodontal history ( )

A. The timing, course and possible triggers of the onset

B. How the disease was treated

C. Bleeding gums and periodontal abscess

D. Whether the parents have periodontitis

E. Oral hygiene

7.2008U2A1009 Methods used to check early contact points do not include ( )

A. Inspection

B. Examination

C. Occlusion paper method

D. Wax bite tablet method

E. Floss

8. 2008U2A1013 II. degree bone resorption describes the bone destruction ( )

A. Alveolar bone resorption is within 1/3 of the root neck

B. Alveolar bone resorption exceeds 1/3 of the root length, but is within 2/3 of the root length or up to 1/2 of the root length

C. Alveolar bone resorption accounts for more than 2/3 of the root length

D. Alveolar bone resorption accounts for more than 1/2 and less than 3/4 of the root length

E. Alveolar bone resorption within 1/2 of the root neck

9.II. Degree of tooth loosening refers to ( )

A. The tooth loosening amplitude is less than 1mm

B. The tooth loosening amplitude is within 0.5~lmm

C. The tooth loosening amplitude is within 2~3mm

D. The tooth loosening amplitude is within 1~3mm

E. The tooth loosening amplitude is within 1~2mm

10.2008U2A1017 Which of the following describes occlusion interference ( )

A. When the anterior extension is occluded, there is extensive contact between the anterior teeth

B. Non-working cusp contact during lateral occlusion

C. When the anterior extension teeth are occlusal, the anterior teeth are in single-point contact

D. When the lateral occlusion, the working side cusps are widely contacted

E. Deep overbite

11. 2008U2A1019 III. degree bone resorption describes the bone destruction as ( )

A. Alveolar bone resorption is within 1/3 of the root neck

B. Alveolar bone resorption exceeds 1/3 of the root length, but is within 2/3 of the root length or up to 1/2 of the root length

C. Alveolar bone resorption accounts for more than 3/4 of the root length

D. Alveolar bone resorption accounts for more than 1/2 and less than 3/4 of the root length

E. Alveolar bone resorption greater than 2/3 of the root length

12.2008U2A1027 The best non-invasive method for judging alveolar bone dynamics is ( )

A. Periodontal probing with pressure-sensitive probes

B. Dental mobility test

C. Dental force force test

D. X-ray digital subtraction technology

E. Gingival crevicular fluid examination

13.2008U2A1031 The force used in periodontal pocket probing is ( )

A. 10～20g

B. 20～25g

C. 25～30g

D. 30～35g

E. 35～40g

14.2013U2A1004 Measure the adhesion level, the appropriate distance is ( )

A. Gingival margin-enamel cementum boundary

B. Tooth neck-pouch bottom

C. Gingival margin - bottom of the bag

D. Enamel bony boundary-pouch bottom

E. Tooth neck-enamel cementum boundary

15.2013U2A1005 Periodontitis bone resorption on X-ray as alveolar bone ( )

A. Height reduction

B. The morphology remains unchanged

C. Increased density

D. Thickening of bony plates

E. Narrowing of the periodontal ligament

16.2015U2A2071 Female, 29 years old, healthy. Complaints: Bleeding gums in the past 3 months and bleeding gums when chewing hard foods. The following tests should be done, with the exception of ( )

A. Oral hygiene status

B. Determination of tooth resultant force

C. Inflammation of the gums

D. Loss of attachment

E. Depth of probing

17.2004U2A1055 Measurement of periodontal pocket depth, to detect ( )

A. Six sites on the buccal lingual side: mesial, central, and distal

B. Near-median and distal loci on the buccal and lingual side

C. Four sites on the buccal side: mesial, central, distal, and lingual center

D. Four loci on the lingual side: mesial, central, distal, and buccal center

E. Two central sites on the buccal lingual side

18.2013U2A1015 Which method cannot be used for the examination of occlusal trauma ( )

A. Examination

B. Inspection

C. Wax bite tablet method

D. Articulation paper test

E. Periodontal probing

19.2002U2A1035 In addition to measuring the depth of the bag, periodontal probing should also be explored ( )

A. Whether the gums are bleeding

B. Amount and distribution of subgingival calculus

C. Whether the root fork is involved

D. Location of the gingival margin

E. All of the above

20.2002U2A1036 Factors that cause loose teeth in periodontitis do not include ( )

A. Alveolar bone resorption

B. Occlusal trauma

C. Acute inflammation of the periodontal ligament

D. Periodontal pocket formation

E. A+B+C+D

21.2012U2A1024 The following should be checked during occlusion examination, except for ( )

A. Whether the dentition is intact

B. Whether there is crowding, dislocation, overbite, and overlay

C. Whether the overbite and coverage relationship are normal

D. Type of occlusion

E. Night grinding habits

22.2012U2A1025 occlusal trauma can be manifested as ( )

A. Horizontal resorption of alveolar bone

B. Asymmetric gingival recession and clefts

C. Formation of pseudoperiodontal pockets

D. Severe inflammation of the gums

E. Redness of the gums during occlusal movements

GPT-4

E. All of the above

E. All of the above

B. Periodontal probe, electrovinometer, thin wax sheet

D. Shake from the normal position to the buccal tongue or mesiodistal direction, with a movement of 1~2mm

D. Periodontal probe examination

D. Whether the parents have periodontitis

E. Floss

B. Alveolar bone resorption exceeds 1/3 of the root length, but is within 2/3 of the root length or up to 1/2 of the root length

E. The tooth loosening amplitude is within 1~2mm

C. When the anterior extension teeth are occlusal, the anterior teeth are in single-point contact

C. Alveolar bone resorption accounts for more than 3/4 of the root length

D. X-ray digital subtraction technology

B. 20～25g

C. Gingival margin - bottom of the bag

A. Height reduction

B. Determination of tooth resultant force

A. Six sites on the buccal lingual side: mesial, central, and distal

E. Periodontal probing

E. All of the above

E. A+B+C+D

E. Night grinding habits

A. Horizontal resorption of alveolar bone

**Chapter 9 Gum Disease**

1.2009U2A1044 Gingival tumor during pregnancy that interferes with eating, if surgery is necessary, optional ( )

A. Surgical excision at 4~6 months of pregnancy

B. Surgical excision at 3~8 months of pregnancy

C. Only plaque control is done, not excision

D. Resolves spontaneously as the gestation months increase

E. All of the above

2.2009U2A1045 Which is wrong to describe the lesions of mild chronic gingivitis ( )

A. Involvement of free gingiva

B. Involvement of the gingival papillae

C. Involvement of attached gingiva

D. Changes in gum color

E. Stippling can disappear

3.2009U2A1046 The most common gum disease is ( )

A. Multiple gingival abscesses

B. Single gingival abscess

C. Gingival papillitis

D. Chronic gingivitis

E. Gingival hyperplasia

4.2009U2A1050 In acute gingival papillitis, the clinical examination shows that the affected tooth has mild percussion pain ( )

A. Periodontal ligament below the gingival papilla is inflamed and edematous

B. Inflammation around the apex

C. Have a periodontal abscess

D. Periodontal trauma

E. Have acute pulpitis

5. The clinical manifestations of 2009U2A1074 proliferative gingivitis are ( )

A. Deep overbite of anterior teeth

B. Hypertrophy of the gingival margin, bulbous hyperplasia of the gingival papillary, and bleeding on probing

C. Periodontal pocket formation

D. Significant halitosis and pain

E. All of the above

6.2016U2A1006 Clinical manifestations of gingivitis ()

A. Significant pain

B. Spontaneous bleeding

C. Periodontal pocket formation

D. Crowded dental arrangement

E. There are often tartar and soft plaque deposits on the neck of teeth

7.2009U2A2108 male, 25 years old, the upper and lower anterior gingival papillae in the oral cavity disappeared and sunken, showing an anti-wavy shape, the buccal and lingual sides of the intergingival papillae were separated, which could be turned over from the tooth surface, and there was tartar and tartar below, and there was no gingival necrosis. The possible causes are ( )

A. Herpetic gingivostomatitis

B. Neutrophil deficiency causes gingival necrosis

C. Chronic gingivitis

D. Chronic necrotizing gingivitis

E. Gingival papillitis

8.2016U2A1022 The most common areas of gingivitis in adolescence are ( )

A. Maxillary molars buccal gingival margin

B. Palatarian gingival margin of maxillary molars

C. Anterior teeth, labial gingival margin, and gingival papillae

D. Maxillary bicuspid buccal gingival margin

E. Mandibular bicuspid interdental papilla

9.2008U2A1005 What are the main causative factors of gingivitis in adolescence ( )

A. Poor restorations

B. Food impaction

C. Tartar

D. Plaque

E. Occlusal trauma

10.2008U2A1011 The suspected pathogen of gingivitis in pregnancy is ( )

A. Actinomycetes viscosus

B. Prevotella intermediates

C. Endomycetes

D. Microdigestive streptococcus

E. Gingival carbon dioxide fibrophage

11.2008U2A1012 Among the plaque in women with gingivitis during pregnancy, the bacteria with the highest detection rate were ( )

A. intermediates

B. Porphyrimonas gingivals

C. Nuclusionella

D. Pseudobacterium fersa

E. Ekenbacterium

12.2013U2A2094 male, 21 years old, short upper lip, clinical examination: hypertrophy of the gingival margin and gingival papilla on the labial side of the upper anterior teeth, covering 1/3 of the crown, and the texture is tough. The most likely diagnosis is ( )

A. Proliferative gingivitis

B. Marginal gingivitis

C. Acute necrotizing gingivitis

D. Drug-induced gingival hyperplasia

E. Gingival fibromatosis

13.2010U2A1034 Gingival tumors during pregnancy are more common in ( )

A. Maxillary bicuspid buccal papilla

B. Mandibular bicuspid buccal alcrual papilla

C. Maxillary molars, buccal gingival papillae

D. Lower anterior labial gingival papillae

E. Mandibular molars, buccal gingival papillae

14.2000U2AN127 male, 27 years old, with sore gums, automatic bleeding for 3 days. Examination: putrefactive halitosis, loss and flattening of the tips of the gingival papillae of multiple teeth, and eroded necrosis of the gingival margins of the lower incisors covered with a grayish-white membrane. The most likely diagnosis of the disease is acute necrotizing gingivitis, and the most helpful ancillary tests are ( )

A. Smear Gram stain

B. Check for white blood cells

C. Photograph curved tomographs

D. Occlusal examination

E. Electromyography

15.2000U2AN129 male, 27 years old, with sore gums and spontaneous bleeding for 3 days. Examination: putrefactive halitosis, loss and flattening of the tips of the gingival papillae of multiple teeth, and eroded necrosis of the gingival margins of the lower incisors covered with a grayish-white membrane. The most likely diagnosis of the disease is acute necrotizing gingivitis, and the main causative organism that causes the disease is ( )

A. Actinobacillus actinobacillus

B. Porphyromonas gingivals

C. Staphylococcus

D. Clostridium and spirochetes

E. Actinomycetes

16.2000U2AN130 male, 27 years old, with sore gums and automatic bleeding for 3 days. Examination: putrefactive halitosis, loss and flattening of the tips of the gingival papillae of multiple teeth, and eroded necrosis of the gingival margins of the lower incisors covered with a grayish-white membrane. The most likely diagnosis of the disease is acute necrotizing gingivitis, and if the diagnosis is clear, and it is considered that systemic drugs should be used in treatment, the best choice of drug is ( )

A. Penicillin

B. Amoxicillin

C. Anti-inflammatory

D. Metronidazole

E. Synergistic bisulfonate tablets

17.2013U2A2095 female, 28 years old, complained: a tumor on the lingual side of the lower anterior teeth grew for 3 months, and slowly enlarged, affecting feeding. If the patient is diagnosed with a gestational tumor and needs to be resected, the following factors should be noted, except ( )

A. Inflammation of the gums

B. Timing of pregnancy

C. Whether or not to take medication during pregnancy

D. Oral hygiene

E. Selection of surgical method (laser, etc.)

18.2010U2A1035 For gestational gingival tumors that require surgical resection, the timing of surgery should be selected in ( )

A. Within 1 month of pregnancy

B. Pregnancy period of 2~3 months

C. Pregnancy period 4~6 months

D. Pregnancy period of 6~8 months

E. 9 months of gestation

19.2004U2A1050 Oral manifestations of leukemia patients do not include ( )

A. The gums are pale or dark red and inelastic

B. The gums have a tendency to bleed and are not easy to stop bleeding

C. Gingival margin is often necrosis

D. Loose and displaced teeth are common

E. Swollen gums that spread to all teeth

20.2013U2AN140 female, 28 years old, in the past 4 months, the gums of the whole mouth have gradually enlarged, the gums are easy to bleed when brushing teeth, and there is a history of occasional automatic bleeding of the gums. If the patient is diagnosed with gevicitis during pregnancy at 6 months of pregnancy, the clinical manifestations are most likely to be ( )

A. Sore, foul-smelling gums

B. Loose teeth arrangement

C. The gums are fibrous and enlarged

D. The gums are bright red and shiny

E. Gingival necrosis

21.2013U2AN141 female, 28 years old, has gradually enlarged gums in the past 4 months, gums are easy to bleed when brushing teeth, and occasionally there is a history of automatic bleeding of gums. If the patient is not pregnant, the oral manifestations of leukemia are suspected, and the diagnosis is confirmed by ( )

A. Biopsy

B. Exfoliated cell smear

C. Leukocyte phagocytosis

D. Leukocyte chemotaxis

E. Blood check

22.2013U2AN142 female, 28 years old, has gradually swollen gums in the past 4 months, gums are easy to bleed when brushing teeth, and occasionally have a history of automatic gingival bleeding. If leukemia is diagnosed, it is also manifested in the oral cavity ( )

A. The gums are pale, fluffy and fragile

B. The gums are bright red, shiny and soft

C. Fluctuating gums

D. Pseudomembrane on the surface of the gums

E. Herpes on the surface of the gums

23.2008U2A1032 The principles of treatment of gingival lesions in leukemia are ( )

A. Remove plaque and tartar immediately and thoroughly

B. Conservative treatment, hemostasis, and anti-infection

C. Removal of hypertrophied gums

D. Root surface leveling

E. Periodontal stoppers are used to prevent bleeding gums

24.2015U2A1001 The anaerobic microorganisms with the largest increase in the number of lesions in acute necrotizing gingivitis are ( )

A. Actinomycetes viscosus with spirochetes

B. With Actinobacillus

C. Fusobacterium and spirochetes

D. Clostridium and Porphyromonas gingivals

E. Porphyrimonospora gingivals

25.2015U2AN135 Female, 27 years old. Complained of swollen gums, bleeding and significant pain for 3 days. Oral examination: full-mouth gingiva swelling affecting the interdental papilla, marginal gingiva and attached gingiva, pale in color, necrotic ulcer at the gingival margin, and local bleeding. Hemorrhage spots may be seen on the oral mucosa, and the ancillary tests that should be done to further confirm the diagnosis are ( )

A. Probing the periodontal pocket

B. Percussion

C. X-rays

D. Perform routine blood tests in a timely manner

E. Perform bacteriological examinations

26.2015U2A1003 The most sensitive antibacterial drugs for the treatment of acute necrotizing ulcerative gingivitis are ( )

A. Tetracycline

B. Chlortetracycline

C. Sulfonamides

D. Metronidazole

E. Penicillin

27.2015U2A2072 Female, 29 years old, complaining: gums bleed easily after brushing teeth in the past 3 months, and gums bleeding when chewing hard food. If the patient is diagnosed with gingivitis of pregnancy, the most likely finding on clinical examination is ( )

A. Gingival papillae flattening

B. Sore, bleeding, foul-smelling gums

C. Bleeding gums and loose teeth

D. Hyperplasia of the gingiva covering 2/3 of the tooth surface

E. The gingival margin and papillae are bright red, soft and shiny

28.2004U2A1051 Swollen gums in leukemia are due to ( )

A. Gingival fibrous connective tissue hyperplasia

B. Gingival epithelial hyperplasia

C. Fibroblast proliferation

D. Massive lymphocyte infiltration

E. Massive infiltration and aggregation of immature, non-functioning leukocytes

29.2004U2A1052 The main pathological manifestations of gingival lesions in leukemia are ( )

A. The gums are filled with a large number of naïve white blood cells

B. There is a large number of capillary hyperplasia in the gums

C. There is a large amount of collagen fiber growth in the gums

D. Gingival epithelial hyperplasia

E. All of the above

30.2015U2AN136 Female, 27 years old. Complained of swollen gums, bleeding and significant pain for 3 days. Oral examination: full-mouth gingiva swelling affecting the interdental papilla, marginal gingiva and attached gingiva, pale in color, necrotic ulcer at the gingival margin, and local bleeding. Hemorrhagic spots may be seen on the oral mucosa. Routine blood count: abnormally increased white blood cell count and immature white blood cells. The initial diagnosis was: gingival manifestations of acute leukemia. Which of the following processing is wrong ( )

A. Cooperate with the physician in the treatment in a timely manner

B. Oral treatment should be conservative

C. Surgery or biopsy histopathology if needed

D. Bleeding can be stopped with local compression or medications

Rinse topically with 3% hydrogen peroxide solution gently

31.2015U2A2073 Female, 27 years old, examination: full mouth gingival hyperplasia covering 1/3~1/2 of the crown. Diagnosis of drug-induced gingival hyperplasia, which one is incorrect about the cause of gingival hyperplasia ( )

A. Increased division activity of fibroblasts

B. Increased secretion of inactive collagen-lytic enzymes

C. Not related to pre-existing gingival inflammation

D. Synthesis is greater than degradation

E. Increased ability to synthesize collagen

32.2008U2A1033 Clinical manifestations of drug-induced gingival hyperplasia ( )

A. Affects all oral teeth, and the upper and lower anterior teeth are heavier

B. The growth can range from marginal gingiva to attached gingiva

C. The gum texture is tough and elastic

D. The gingiva are spherical and nodal hyperplasia

E. All of the above

33.2004U2A1053 What is the obvious relationship between the degree of drug-induced gingival hyperplasia ( )

A. Oral hygiene status

B. Duration of medication

C. Dosage

D. Age

E. Gender

34.2010U2A1036 Drugs that can cause drug-induced gingival hyperplasia are ( )

A. Phenytoin

B. Phenytoin + nifedipine

C. Phenytoin + cyclosporine

D. Nifedipine + cyclosporine

E. Phenytoin + nifedipine + cyclosporine

35.2015U2A1004 Drugs that can cause gingival hyperplasia are ( )

A. Cyclosporine

B. Penicillin

C. Metronidazole

D. Tetracycline

E. Ibuprofen

36.2012U2A2107 female, 55 years old, with a history of hypertension for many years. Clinical: congestion and edema of the gingival margin and papilla, and the whole mouth gingival hyperplasia covers 1/3~1/2 of the crown.

A. Dalontinoids

B. Cyclosporine

C. Nifepyridine

D. Eliminate heartache

E. Reserpine

37.2005U2A1030 The causes of gingivitis in pregnancy are ( )

A. Endocrine changes

B. Occlusal trauma

C. Pregnancy itself

D. Malnutrition

E. All of the above

38.2010U2A1039 Which of the following is NOT a direct cause of gingivitis during pregnancy ( )

A. Plaque

B. Tartar

C. Food impaction

D. Poor restorations

E. Pregnancy

39.2015U2A1008 The drugs that can cause gingival hyperplasia are ( )

A. Nifepyridine

B. Metronidazole

C. Spiramycin

D. Dimethylaminetetracycline

E. Penicillin

40.2013U2A2096 Female, 55 years old, with a history of hypertension for many years, diagnosed with drug-induced gingival hyperplasia. Which of the patient's treatment options is incorrect ( )

A. Periodontal basic treatment

B. Systemic antibiotics

C. Oral health education

D. It is better to switch to another class of blood pressure medication

E. Periodontal surgery may be performed if necessary

41.2013U2A1016 Gingival fibromatosis is characterized by ( )

A. Soft gums

B. Never occurs in baby teeth

C. Gingival hyperplasia covers 2/3 of the crown

D. No involvement of attached gingiva

E. Normal gum color

42.2013U2A2097 Male, 30 years old, complains: automatic bleeding of the gingiva. Pain for more than 3 days with low-grade fever, the most likely diagnosis in this patient is ( )

A. Interdental papillitis

B. Hypertrophic gingivitis

C. Acute necrotizing gingivitis

D. Herpetic gingivostomatitis

E. Juvenile periodontitis

43.2013U2A2105 Male, 28 years old, red and swollen gums in the anterior teeth area for 1 year, bulbous hypertrophy of the gingival papilla, mouth breathing habits, tartar (+), good general condition. The most likely diagnosis in this patient is ( )

A. Adolescent gingivitis

B. Ulcerative gingivitis

C. Juvenile periodontitis

D. Proliferative gingivitis

E. Drug-induced gingival hyperplasia

44.2013U2A2106 female, 23 years old, with gum pain and easy bleeding in the past 1 week, abnormal bad breath, and necrosis of the papilla on the labial side of the mandibular anterior teeth, the most likely diagnosis and pathogenic bacteria are ( )

A. Gingivitis of pregnancy, Prevotella intermediates

B. Acute necrotizing ulcerative gingivitis, spirochetes and clostridium

C. Chronic gingivitis, Actinomyces viscosus

D. Adolescent gingivitis, Porphyrimonas gingivals

E. Proliferative gingivitis, Actinomyces nepheri

45.2003U2A2083 A 29-year-old male patient is in good health. Complained of spontaneous bleeding from the gums for 1 month, and after staying up late for 3 days, the bleeding was severe and accompanied by gingival pain and halitosis, accompanied by low-grade fever and general malaise. The most likely diagnosis is ( )

A. Marginal gingivitis

B. Herpetic gingivostomatitis

C. Juvenile periodontitis

D. Interdental papillitis

E. Acute necrotizing ulcerative gingivitis

46.2013U2A2108 Male, 55 years old. Severe pain and bleeding in the anterior mandibular teeth for 3 days. Bad taste in the mouth. The patient smokes a lot. On oral examination 43211234 gingival margin on the left and right lower lip sides is like an insect erosion, covered with a gray-brown pseudofilm, which can be wiped off to leave a bleeding surface. The gingival papillae and marginal gingiva are in a straight knife shape. No loosening, no periodontal pockets. There is a peculiar putrid odor in the mouth. Submandibular lymphadenopathy is swollen and tender. Possible diagnoses are ( )

A. Gingival manifestations of acute leukemia

B. Acute papillitis gingivals

C. Acute necrotic ulcerative gingivitis

D. Acute necrotic ulcerative periodontitis

E. Acute herpetic gingivitis

47.2013U2A2109 Male, 30 years old. Spontaneous swelling and pain in the lower left back tooth, bleeding easily when sucking. He complained of picking his teeth with a toothpick 5 days ago. Oral examination: the left 56 interdental papillae are red and swollen with obvious probing pain and easy bleeding. There is no caries and non-caries disease in the tooth. Percussion (+), no loosening, no periodontal pockets probed. Possible diagnoses are ( )

A. Acute pulpitis

B. Acute periodontitis

C. Acute apical periodontitis

D. Acute papillitis gingival

E. Acute necrotizing ulcerative gingivitis

48.2000U2AN128 Male, 27 years old, with sore gums, spontaneous bleeding for 3 days. Examination: putrefactive halitosis, loss and flattening of the tips of the gingival papillae of multiple teeth, and eroded necrosis of the gingival margins of the lower incisors covered with a grayish-white membrane. The most likely diagnosis of the disease is ( )

A. Marginal gingivitis

B. Proliferative gingivitis

C. Acute necrotizing gingivitis

D. Chronic periodontitis

E. Rapidly progressive periodontitis

49.2012U2A1015 The following statements about the main clinical manifestations of ANUG are incorrect ( )

A. The main characteristic lesion is necrosis of the gingival papilla and marginal gingiva

B. The course of the disease is short, generally a few days to 1~2 weeks

C. Lesions tend to involve only the attached gingiva

D. Significant pain

E. Corrupt bad breath

50.2013U2A2111 16-year-old girl, anterior teeth are irregular, crowded, anterior teeth lip side of the papillae are bulbous protrusions, soft and shiny texture, a small amount of plaque, etc. The most likely diagnosis in this patient is ( )

A. Gingival fibromatosis

B. Gingivitis of pregnancy

C. Drug-induced gingival hyperplasia

D. Adolescent gingivitis

E. Leukemia

51.2012U2A1016 ANUG patients who are not treated in a timely and appropriate manner, which bacteria can be co-infected with which bacteria can cause chancre ( )

A. aeruginosa aeruginosa

B. Staphylococcus aureus perfringens

C. Aeroperfringens

D. Escherichia coli

E. mutans

52.2000U2AN131 female, 50 years old. Gingival hyperplasia affects chewing for a year. Examination reveals hypertrophy of the whole mouth gingival hypertrophy, covering about 1/2 of the tooth surface, nodular shape. There was bleeding during probing, and some teeth were displaced in areas where gingival hyperplasia was severe. To aid in the diagnosis, the most important history to ask is ( )

A. Menstrual status

B. Children's situation

C. Dietary status

D. Medication history

E. Epidemiological history

53.2000U2AN132 female, 50 years old. Gingival hyperplasia affects chewing for a year. Examination reveals hypertrophy of the whole mouth gingival hypertrophy, covering about 1/2 of the tooth surface, nodular shape. There was bleeding during probing, and some teeth were displaced in areas where gingival hyperplasia was severe. To aid in the diagnosis, the most important history to ask is a history of medication, and if the patient has had a kidney transplant and has been treated for a year, the most likely diagnosis is ( )

A. Drug-induced gingival hyperplasia

B. Gingival fibromatosis

C. Necrotizing gingivitis

D. Rapidly progressive periodontitis

E. Gum lesions in leukemia

54.2010U2A1046 Manifestations of chronic gingivitis are ( )

A. Significant pain

B. Periodontal pocket formation

C. The teeth are not loose

D. X-rays show horizontal resorption of alveolar bone

E. Tooth displacement

55.2000U2AN133 female, 50 years old. Gingival hyperplasia affects chewing for a year. Examination reveals hypertrophy of the whole mouth gingival hypertrophy, covering about 1/2 of the tooth surface, nodular shape. There was bleeding during probing, and some teeth were displaced in areas where gingival hyperplasia was severe. In order to aid in the diagnosis, the most important medical history to ask is a history of medication, and if the patient has had a kidney transplant and has been treated for a year, the most likely diagnosis is drug-induced gingival hyperplasia, the main cause of which is ( )

A. Immunosuppressant cyclosporine

B. Genetic factors

C. Spirochetes

D. Massive leukocyte infiltration

E. High blood pressure

56.2007U2A1005 Which description is incorrect about ANUG ( )

A. It is more common in young and middle-aged men

B. Grayish-yellow necrotic tissue on the gingival margin

C. Bad breath, easy bleeding or spontaneous bleeding

D. Improper treatment can turn into chronic recurrent attacks

E. X-ray showing alveolar bone resorption

57.2007U2A1006 Causes of acute papillitis gingival do not include ( )

A. Mechanical stimulation

B. Chemical irritation

C. Plaque irritation

D. Poor prosthetic irritation

E. Food impaction stimulation

58.2002U2A1037 The earliest gum disease that can occur in early childhood is ( )

A. Chronic gingivitis

B. Proliferative gingivitis

C. Hereditary gingival fibromatosis

D. Gingival tumors

E. Drug-induced gingival hyperplasia

59.2002U2A1038 Which of the following gum diseases is most common ( )

A. Acute necrotic ulcerative gingivitis

B. Acute papillitis gingivals

C. Chronic gingivitis

D. Gingivitis of pregnancy

E. Gingival tumors

60.2002U2A1039 The clinical manifestations of chronic gingivitis are ( )

A. Periodontal pocket formation

B. Alveolar bone resorption

C. Loose teeth

D. Tooth displacement

E. Probing for bleeding gums

61.2014U2A2096 Female, 25 years old, bleeding from tooth brushing for 3 weeks, examination: gingival papilla mild congestion, edema, tartar (+), most of the probing depth is 2-3mm, and there is no loss of attachment. This patient is most likely to be ( )

A. Interdental papillitis

B. Marginal gingivitis

C. Adolescent gingivitis

D. Juvenile periodontitis

E. Acute necrotizing gingivitis

62.2014U2A1015 Causes of bleeding gums do not include ( )

A. Inflammation

B. Thrombocytopenic purpura

C. Acute leukemia

D. Aplastic anemia

E. Addison's illness

63.2014U2A2098 18-year-old patient complains of bleeding from tooth brushing for several months, found loose teeth (-), red and swollen gums, periodontal probing depth ≤3mm, tartar (++), bleeding on probing, best treatment plan ( )

A. Taking amoxicillin and metronidazole

B. Oral hygiene education and supragagingival hygiene

C. Oral hygiene education, supragingival cleaning, root planing

D. Take periodontine tablets and use gargles

E. Perform gingivectomy

64.2014U2A2099 male, 58 years old, history of hypertension for many years, clinical: congestion and edema of the gingival margin and gingival papilla of the whole mouth, gingival hyperplasia covering 1/2 of the crown, and loose displacement of the lower anterior teeth. The most likely diagnosis is ( )

A. Hypertrophic gingivitis

B. Gingival fibromatosis

C. Rapidly progressive periodontitis

D. Drug-induced gingival hyperplasia

E. Interdental papillitis

65.2014U2A2100 female, 57 years old, clinical: gingival margin and gingival papilla congestion and edema, gingival hyperplasia covering 1/3~1/2 of the crown, and loose displacement of the lower anterior teeth. To determine the diagnosis, it is most important to ask ( )

A. Family history

B. History of menopause

C. History of hypertension

D. History of oral hygiene

E. Mouth breathing habits

66.2014U2A2101 female, 11 years old, bleeding from tooth brushing for 1 month, no obvious pain symptoms, oral antibiotics no obvious relief. Examination: An oval growth was found in the 34 buccal papillae on the lower left side, and the bleeding index was 4, but the subgingival calculus was not reached. X-ray showed that the alveolar bone mineral density of the lower left 34 cells was reduced. The most likely diagnosis is ( )

A. Chronic gingivitis

B. Gingival tumors

C. Drug-induced gingival hyperplasia

D. Gingival papillitis

E. Adolescent gingivitis

67.2014U2A2102 Male, 42 years old, bleeding and painful gums for 3 days, bad breath, examination: individual gingival papillae have grayish-white necrosis. If necrotizing gingivitis is diagnosed, the patient may present with the following signs, except (

A. Bad breath in the mouth

B. Low-grade fever

C. Decreased physical resistance

D. Submandibular lymphadenopathy

E. Presence of protoblasts in peripheral blood

68.2014U2A2103 Female, 27 years old, examination: gingival hyperplasia of the whole mouth covering 1/2 of the crown. History that should be emphasized, except ( )

A. History of pregnancy

B. History of acute hepatitis

C. History of epilepsy

D. Family history

E. Current medical condition

69.2014U2A2104 Male, 25 years old. Lower anterior teeth, swollen, painful, bleeding, foul odor in the mouth for 2 days. Oral examination reveals gingival margin necrosis. The initial diagnosis was: acute necrotizing ulcerative gingivitis. The most appropriate emergency response is ( )

A. Gently remove the necrotic tissue, and preliminarily scrape off the large pieces of tartar, rinse with 3% hydrogen peroxide solution

B. Systemic drug therapy

C. Subgingival scaling

D. Scraping of the inner wall of the bag

E. A+B

70.2014U2A2105 Female, 20 years old. Spontaneous swelling and pain in the left maxillary posterior tooth, bleeding for 3 days while sucking. History of fish bone stab wounds 4 days ago. On oral examination, the pain of probing between the teeth in the upper left 67 was obvious, the gingival papilla was red and swollen, percussion (+), there was no loosening, and the periodontal pocket was not explored. The following emergency measures are appropriate ( )

A. Removal of irritants such as tartar from the affected tooth

B. Irrigation of the interdental papilla with 3% hydrogen peroxide solution and normal saline solution alternately

C. Topical application of 2% iodine glycerin

D. Gargle with 0.2% chlorhexidine solution

E. All of the above

71.2010U2A1053 Clinical features of gingival hypertrophy ( )

A. Gums are dark red or dark red

B. The gums are soft and inflamed with a smooth surface

C. Gingival margin hypertrophy

D. The gingival papilla is globular and often covers 1/3 or more of the crown near the neck, and is prone to bleeding

E. All of the above are true

72. The causes of gingivitis in pregnancy are ( )

A. Endocrine changes

B. Occlusal trauma

C. Pregnancy itself

D. Malnutrition

E. All of the above

73.2005U2A1031 Which hormone level in blood is consistent with the clinical changes in gingivitis in pregnancy ( )

A. Androgens

B. Glucocorticoids

C. Progesterone

D. Thyroxine

E. Parathyroid hormone

74.2005U2A1032 Which is correct about gingivitis in pregnancy ( )

A. Pregnancy itself can cause gingivitis

B. Without local irritants and plaque, gingivitis gestationum can also occur

C. Can occur in individual teeth or whole mouth gingiva, with the posterior area being heavier than the anterior region

D. Changes in sex hormone levels during pregnancy can exacerbate or alter the characteristics of pre-existing chronic gingivitis

E. If the gestational tumor is large and obstructs eating, surgical resection between 7~9 months of pregnancy can be selected as much as possible

75.2005U2A1033 In addition to pregnant women, what other groups of women will experience gingivitis symptoms during pregnancy ( )

A. Women of childbearing age

B. Women on long-term oral contraceptives

C. Women with dysmenorrhea

D. Menopausal women

E. Women who have undergone sterilization

76.2013U2A2121 Female, 27 years old, chief complaint: In the past 2 months, it was found that the whole mouth gingiva was generally hyperplistic. Clinical: Gingival hyperplasia of the whole mouth, covering 1/2 or more of the tooth surface, medium texture, grayish-white color. When taking a medical history, the medical history that should be taken is the focus of the examination, except for ( )

A. History of automatic bleeding from the gums

B. History of epilepsy

C. History of hemichewing

D. Family history

E. Recent general condition

77.2003U2A2084 patient, male, 35 years old, had bleeding from gum brushing for more than 4 years. Bleeding is also caused by touching while eating, which can be stopped by gargling. Examination: gingival redness and swelling, probing depth ≤3mm, X-ray: no alveolar crest resorption. The most likely diagnosis in this patient is ( )

A. Adolescent gingivitis

B. Chronic periodontitis

C. Chronic gingivitis

D. Necrotizing gingivitis

E. Aggressive periodontitis

78.2003U2AN135 A 29-year-old male patient is in good health. Complained of spontaneous bleeding from the gums for 1 month, and after staying up late for 3 days, the bleeding was severe and accompanied by gingival pain and halitosis, accompanied by low-grade fever and general malaise. The most likely diagnosis is acute necrotizing ulcerative gingivitis. If acute necrotizing ulcerative gingivitis is diagnosed, the most valuable adjunct is a plaque smear. If acute necrotizing ulcerative gingivitis is diagnosed, its causative bacteria are mainly ( )

A. Actinomycetes and spirochetes

B. Actinomycetes and Clostridium

C. Fusobacterium and streptococcus

D. Clostridium and spirochetes

E. Spirochetes and streptococcus

79.2005U2A1034 Acute gingival abscess without any symptoms ( )

A. Redness, swelling and throbbing pain in most interdental papillae

B. Both the affected tooth and the adjacent tooth are sensitive to percussion

C. Elevated peripheral white blood cell count

D. The oral mucosa is often generally red and swollen, with ulcers and pseudomembranes

E. Elevated body temperature, regional lymphadenopathy

80.2003U2AN134 A 29-year-old male patient is in good health. Complained of spontaneous bleeding from the gums for 1 month, and after staying up late for 3 days, the bleeding was severe and accompanied by gingival pain and halitosis, accompanied by low-grade fever and general malaise. The most likely diagnosis is acute necrotizing ulcerative gingivitis. If acute necrotizing ulcerative gingivitis is diagnosed, the most valuable ancillary tests are ( )

A. Plaque smear

B. X-rays

C. Histopathology

D. Immunoassays

E. Routine blood examination

81.2003U2A1005 In addition to clinical features, commonly used auxiliary diagnostic methods in the diagnosis of acute necrotizing gingivitis ( )

A. Biopsy

B. Bacterial culture

C. Necrosis and gingival tissue culture

D. Bacteriological smear of the lesion area

E. Exfoliated cell examination

82.2003U2AN136 A 29-year-old male patient is in good health. Complained of spontaneous bleeding from the gums for 1 month, and after staying up late for 3 days, the bleeding was severe and accompanied by gingival pain and halitosis, accompanied by low-grade fever and general malaise. The best initial treatment for this patient is ( )

A. Oral antibiotics

B. Conservative treatment, with various auxiliary examinations to confirm the diagnosis

C. Remove tartar and irritants as much as possible and rinse with 3% hydrogen peroxide

D. Oral hygiene education, rinse with 1% hydrogen peroxide for 3 days

E. Remove large pieces of tartar and necrosis and rinse with 3% hydrogen peroxide

83.2002U2A1056 Clinical features of acute necrotizing ulcerative gingivitis ( )

A. Red and swollen gums, loose teeth, periodontal pus

B. Cut-like chipping of the gingival papilla, painful bleeding, putrefactive halitosis

C. Extensive vesicles of the gingival mucosa

D. Milky white, slightly convex dots or plaques on the gingival mucosa

E. White papules of the gingival mucosa are connected in lines or reticulations

84.2000U2A1011 Characteristics of chronic gingivitis do not include ( )

A. In severe cases, the attached gingiva may be affected

B. Gingival pockets can be more than 3mm

C. There is a slight loss of attachment

D. Red, swollen and fluffy gums

E. Bleeding easily when touched

85.1999U2A1011 The best treatment for acute multiple gingival abscess is ( )

A. Topical therapy with antibiotic therapy

B. Chlorhexidine solution gargling for 1 week with topical treatment

C. Gargle with 2% H2O2 for 1 week, then apply topical treatment

D. Topical treatment combined with integrative medicine

E. Topical treatment with supportive care

86.2014U2A1020 Which of the following is NOT the main basis for differentiating gingival hyperplastic disease ( )

A. Bone marrow image

B. Systemic medical history

C. X-rays

D. Complaints and clinical manifestations

E. Medication history

87.2000U2A2080 female, 28 years old, has gradually enlarged gums in the past four months, gums are easy to bleed when brushing teeth, and occasionally have a history of automatic bleeding gums. If the patient is diagnosed with gevicitis during pregnancy at 6 months of pregnancy, the clinical manifestations are most likely to be ( )

A. Sore, foul-smelling gums

B. Loose teeth

C. The gums are fibrous and enlarged

D. The gums are bright red and shiny

E. Gingival necrosis

88.2015U2A1023 The factors most closely related to the occurrence of gingivitis in pregnancy are ( )

A. Age

B. Genetic factors

C. History of smoking

D. Changes in sex hormones in the body

E. Mental stress

89.2018U2A1009 Local irritants causing chronic gingivitis are as follows, with the exception of ( )

A. High point of occlusal filling

B. Food impaction

C. Misaligned dentition

D. Tartar

E. Poor restorations

90.2019U2A1001 Medications associated with drug-induced gingival hypertrophy are ( )

A. Cyclosporine A

B. Spiramycin

C. Doxycycline

D. Amoxicillin

E. Metronidazole

91.2017U2A1029 Gingival hypertrophy with drug hyperplasia begins in ( )

A. Lingual gingival papillae

B. Palatal lateral gingival papillae

C. Near midgingival papillae

D. Distal gingival papillae

E. Labial-buccal lateral gingival papillae

92.2017U2A1026 Which of the following drugs does not cause drug-induced gingival hyperplasia ( )

A. Phenytoin

B. Verapamil

C. Nifedipine

D. Cyclosporine

E. Morphine

93.2017U2A1025 The clinical manifestations of gingival lesions in leukemia are unlikely to occur ( )

A. Swollen gums are often localized

B. The tissue is soft and fragile or of medium hardness, and the surface is shiny

C. Color: dark redness, cyanosis or pale

D. Significant gingival inflammation

E. Swollen gums can cover part of the tooth surface

94.2017U2A1028 Pathogenesis of acute necrotizing ulcerative gingivitis does not include ( )

A. It usually occurs in young adults

B. Onset is acute, often associated with stress

C. At the beginning, the gingival papilla is congested and edematous, and some ulcers appear

D. Rapid progression, necrosis of the interdental papillae and marginal gingiva

E. Long course of disease, bleeding gums, loose teeth

95.2017U2A1027 Gingival hyperplasia caused by cyclosporine is different from that caused by phenytoin.

A. Cyclosporine-induced hyperplasia has more components of blood vessels and chronic inflammatory cells in the tissues

B. Phenytoin-induced hyperplasia has more components of blood vessels and chronic inflammatory cells in the tissues

C. Cyclosporine causes a significantly higher number of hyperplastic tissues than phenytoin

D. Phenytoin causes significantly more hyperplastic tissue than cyclosporine

E. Cyclosporine-induced hyperplasia is significantly larger in size than phenytoin

96.2017U2A1024 The following description of the pathological changes in gingival lesions in leukemia is incorrect ( )

A. The gingival epithelium and connective tissue are filled with dense naïve white blood cells

B. Normal neutrophils may be seen

C. Focal infiltrates of lymphocytes and plasma cells may be seen

D. Collagen fibers are replaced by naïve blood cells

E. Capillary constriction, tissue necrosis is seen

97.2017U2A1023 Chlorhexidine gargle, incorrect item ( )

A. Broad-spectrum antimicrobials

B. Long-term gargling stains the surface of the tooth and the dorsum of the tongue

C. Drug-resistant strains that are susceptible to bacteria

D. Gargle twice a day for 1 minute

E. It has a bitter taste and causes a temporary change in taste

98.2020U1A1059 A better indicator of gingivitis activity is ( )

A. Plaque index

B. Gingival index

C. Gingival sulcus bleeding index

D. Simplification of the Oral Hygiene Index

E. Community periodontal index

GPT-4

1. Surgical excision at 4~6 months of pregnancy
2. Involvement of attached gingiva
3. Chronic gingivitis
4. B. Inflammation around the apex
5. All of the above
6. B. Spontaneous bleeding and E. There are often tartar and soft plaque deposits on the neck of teeth are the most accurate clinical manifestations of gingivitis.
7. C. Anterior teeth, labial gingival margin, and gingival papillae
8. D. Plaque
9. D. Microdigestive streptococcus
10. Pruskiella intermediates
11. D. Drug-induced gingival hyperplasia
12. D. Lower anterior labial gingival papillae
13. There is a periodontal abscess
14. Clostridium and spirochetes
15. Metronidazole
16. Choice of surgical method (laser, etc.)
17. C. Pregnancy period 4~6 months
18. D. Loose and displaced teeth are common
19. D. The gums are bright red and shiny
20. Check the blood
21. B. The gums are bright red, shiny and soft
22. B. Conservative treatment, hemostasis, and anti-infection
23. C. Fusobacterium and spirochetes
24. D. Perform routine blood tests in a timely manner
25. D. Metronidazole
26. The gingival margin and papillae are bright red, soft and shiny
27. E. Massive infiltration and aggregation of immature, non-functioning leukocytes
28. A. The gums are filled with a large number of naïve white blood cells
29. Rinse topically with 3% hydrogen peroxide solution gently
30. B. Increased secretion of inactive collagen-lytic enzymes
31. E. All of the above
32. A. Oral hygiene status
33. E. Phenytoin + nifedipine + cyclosporine
34. A. Cyclosporine
35. B. Cyclosporine
36. E. All of the above
37. E. Pregnancy
38. A. Nifepyridine
39. B. Systemic antibiotics
40. E. Normal gum color
41. C. Acute necrotizing gingivitis
42. A. Adolescent gingivitis
43. B. Acute necrotizing ulcerative gingivitis, spirochetes and clostridium
44. E. Acute necrotizing ulcerative gingivitis
45. C. Acute necrotic ulcerative gingivitis
46. D. Acute papillitis gingival
47. C. Acute necrotizing gingivitis
48. C. Lesions tend to involve only the attached gingiva
49. D. Adolescent gingivitis
50. E. mutans
51. D. Medication history
52. A. Drug-induced gingival hyperplasia
53. C. The teeth are not loose
54. A. Immunosuppressant cyclosporine
55. E. X-ray showing alveolar bone resorption
56. B. Chemical irritation
57. A. Chronic gingivitis
58. C. Chronic gingivitis
59. E. Probing for bleeding gums
60. B. Marginal gingivitis
61. E. Addison's illness
62. B. Oral hygiene education and supragagingival hygiene
63. D. Drug-induced gingival hyperplasia
64. C. History of hypertension
65. B. Gingival tumors
66. E. Presence of protoblasts in peripheral blood
67. B. History of acute hepatitis
68. E. A+B
69. E. All of the above
70. E. All of the above are true
71. E. All of the above
72. C. Progesterone
73. D. Changes in sex hormone levels during pregnancy can exacerbate or alter the characteristics of pre-existing chronic gingivitis
74. B. Women on long-term oral contraceptives
75. A. History of automatic bleeding from the gums
76. C. Chronic gingivitis
77. D. Clostridium and spirochetes
78. D. The oral mucosa is often generally red and swollen, with ulcers and pseudomembranes
79. E. Routine blood examination
80. D. Bacteriological smear of the lesion area
81. C. Remove tartar and irritants as much as possible and rinse with 3% hydrogen peroxide
82. B. Cut-like chipping of the gingival papilla, painful bleeding, putrefactive halitosis
83. E. Bleeding easily when touched
84. A. Topical therapy with antibiotic therapy
85. A. Bone marrow image
86. D. The gums are bright red and shiny
87. D. Changes in sex hormones in the body
88. E. Poor restorations
89. A. Cyclosporine A
90. E. Labial-buccal lateral gingival papillae
91. E. Morphine
92. E. Swollen gums can cover part of the tooth surface
93. E. Long course of disease, bleeding gums, loose teeth
94. A. Cyclosporine-induced hyperplasia has more components of blood vessels and chronic inflammatory cells in the tissues
95. B. Normal neutrophils may be seen
96. C. Drug-resistant strains that are susceptible to bacteria
97. C. Gingival sulcus bleeding index

**Chapter 10 Periodontitis**

1. 2009U2A1054 Which is wrong to describe rapidly progressive periodontitis ( )

A. Onset is between adolescence and 35 years

B. Lesions are diffuse and involve most teeth

C. Severe and rapid bone destruction that continues to develop

D. Most patients have neutrophil dysfunction

E. Sometimes accompanied by systemic symptoms

2.2009U2A1056 Palmoplantar keratosis-periodontal destruction syndrome is characterized by ( )

A. There is significant chronic inflammation of the wall of the periodontal pocket

B. Osteoclast activity is evident and osteogenic activity is low

C. Predominantly plasma cell infiltration

D. The cementum at the root of the affected tooth is very thin

E. All of the above

3.2009U2A1073 Main causes of periodontitis in adolescents ( )

A. There are special microorganisms in the plaque

B. Defective neutrophil chemotaxis

C. Familial hereditary

D. Neutrophil phagocytosis defect

E. All of the above

4.2009U2A1075 Clinical manifestations of juvenile periodontitis exclude ( )

A. The age of onset is more common from adolescence to 25 years of age

B. Tartar irritation and gum inflammation are obvious

C. Significant tooth displacement

D. Significant loosening of teeth

E. Deeper periodontal pockets on the adjacent surface

5. 2016U2A1001 Palmoplantar Keratosis-Periodontal Destruction Syndrome belongs to ( )

A. Autosomal recessive inheritance

B. Autosomal dominant inheritance

C. Sex chromosome recessive inheritance

D. Sex chromosome dominant inheritance

E. Genetic disorders are not associated with plaque

6.2016U2A1015 Which is wrong in the description of the clinical manifestations of palmoplantar keratosis-periodontal destruction syndrome ( )

A. Skin and periodontal lesions usually co-occur before the age of 4 years

B. The child is intellectually disabled but has normal physical development

C. Invasion of both primary and permanent teeth

D. There are deep periodontal pockets with rapid alveolar bone resorption

E. This disease is rare, and the prevalence in the population is about 1/1 million ~ 4/1 million

7.2016U2A1018 Which of the following is false about the narrative of juvenile periodontitis ( )

A. It occurs mainly in young people from adolescence to 25 years of age, and is more common in women than in men

B. In early patients, plaque and tartar are scarce, and the degree of periodontal tissue destruction is not proportional to the amount of local irritants

C. X-ray showed that the first molars were absorbed horizontally in the near and distal directions, and the incisors were mostly in an arc of absorption

D. The course of the disease progresses rapidly

E. There may be a family history of hereditary history

8.2016U2A1025 Juvenile periodontitis, tooth displacement direction is ( )

A. Upper incisor labial displacement

B. Labial displacement of the lower incisor

C. Distal displacement of the upper incisors

D. Distal displacement of the lower incisors

E. Distal displacement of the labial side of the upper and lower incisors

9. 2008U2A1007 Characteristic manifestations of early juvenile periodontitis do not include ( )

A. Bleeding gums

B. It is more common in adolescents between adolescence and 25 years of age

C. The amount of plaque and tartar is small, and the gingival inflammation is mild, but there are deep periodontal pockets

D. X-rays showing typical "arc absorption" of the first molars

E. Early appearance of loose and displaced teeth

10. 2008U2A1016 Closely related to the occurrence of localized juvenile periodontitis is ( )

A. Aggregator actinomyces

B. Actinomycetes viscosus

C. prawela intermediates

D. Porphyrimonas gingivals

E. Pseudobacterium fersa

11.2000U2A1008 Periodontitis dominated by alveolar bone resorption is ( )

A. Compound periodontitis

B. Advanced adult periodontitis

C. Juvenile periodontitis

D. Rapidly progressive periodontitis

E. Periodontal abscess

12.2015U2A1002 The most obvious loosening and displacement of teeth in juvenile periodontitis is ( )

A. Mandibular premolars

B. Mandibular molars

C. Mandibular anterior teeth

D. Maxillary molars

E. Maxillary anterior teeth

13.2015U2AN137 male, 33 years old, chief complaint: In the past 1~2 years, he felt that his whole mouth teeth were loose, his chewing strength was weakened, and his front teeth were displaced. The most likely initial impression is ( )

A. Chronic periodontitis

B. Rapidly progressive periodontitis

C. Juvenile periodontitis

D. Acute multiple periodontal abscess

E. Acute necrotizing gingivitis

14.2015U2A2074 female, 33 years old, chief complaint: In the past 1~2 years, most of the teeth in the whole mouth have been loosened and the chewing ability has weakened. If rapidly progressive periodontitis is diagnosed, the most likely findings on clinical examination are ( )

A. Most teeth are displaced

B. Multiple periodontal abscesses

C. Molar root bifurcation lesion 3rd degree

D. Most alveolar bones are moderately and severely absorbed

E. Most gingival recession is significant

15.2015U2A2075 female, 28 years old, X-ray: alveolar bone destruction of 6 and 12 above and below left and right, but the alveolar bone of other teeth was also damaged, and the degree varied. The most likely diagnosis for this patient is ( )

A. Chronic periodontitis

B. Rapidly progressive periodontitis

C. Juvenile periodontitis

D. Acute necrotizing gingivitis

E. Multiple periodontal abscesses

16.2015U2AN138 male, 34 years old, complained: In the past 1~2 years, he felt that his teeth were loose and his chewing strength was weakened. If the patient is diagnosed with rapidly progressive periodontitis, the most common cause of loose teeth in this patient is ( )

A. Inflammation of the gums

B. Resorption of alveolar bone

C. Primary occlusal trauma

D. Secondary occlusal trauma

E. Periodontal ligament inflammation

17.2010U2A1038 Which of the following patients with periodontal disease is more likely to have peripheral blood neutrophil dysfunction ( )

A. Chronic gingivitis

B. Juvenile periodontitis

C. Periodontitis in adults

D. Acute necrotic ulcerative gingivitis

E. Periodontal atrophy

18.2015U2A1005 Periodontal maintenance interval for juvenile periodontitis, initial should be ( )

A. 1~2 months

B. 4~6 months

C. 6~8 months

D. 8~10 months

E. 6~12 months

19.2015U2A1009 The most characteristic marker that distinguishes early periodontitis from gingivitis is ( )

A. Visiting ≥ 3 dishes

B. Bleeding on probing

C. Loose gums

D. Weakness in chewing

E. Whether the enamel cementum boundary can be explored

20.2013U2A1007 Compound Periodontitis ( )

A. Both localized and systemic damage

B. Both supra-bony and subbony pockets

C. Gingivitis and periodontitis are present at the same time

D. Inflammatory damage coexists with trauma

E. None of the above

21.2013U2A2100 female, 21 years old, distal displacement of upper incisors, widening of the gap, sought treatment. Examination: periodontal probing of the upper incisors was 6mm, the first permanent molars were loosened I degree, periodontal probing was 6mm, and X-rays showed that the alveolar bone in the upper anterior area was horizontally resorpted, and the first molars were arc-shaped resorption. Diagnosed as ( )

A. Rapidly progressive periodontitis

B. Chronic periodontitis

C. Localized juvenile periodontitis

D. Preadolescent periodontitis

E. Diffuse juvenile periodontitis

22. 2013U2A2102 patient, female, 23 years old. Complaints of chewing weakness. oral examination; The periodontal pocket of the upper and lower first molars was 6 mm, loose I.°, plaque index and gingival index 1, and the preliminary diagnosis was juvenile periodontitis. The main pathogens of the disease are ( )

A. Actinobacillus actinobacillus

B. Prevotella intermediates

C. Nuclusionella

D. Porphyromonas gingivals

E. Campylobacter rectum

23.2013U2A2104 Male, 17 years old, bleeding from tooth brushing, loose and displaced front teeth for 1 year, sometimes swollen and painful gums. If you want to diagnose juvenile periodontitis, you also need to check ( )

A. Attachment level

B. Periodontal pocket depth

C. X-rays

D. Occlusal relationship

E. All of the above examinations are required

24.2010U2A1047 The microorganisms that play an important role in AIDS-related periodontal disease are ( )

A. Streptococcus

B. Candida albicans

C. Viruses

D. Chlamydia

E. Mycoplasma

25.2007U2A1007 Periodontitis is now recognized as ( )

A. Infectious diseases with multifactorial pathogenesis

B. Geneticly related diseases

C. Tissue degeneration with bacterial infection

D. Occlusal trauma is one of the main causative factors-

E. Diseases caused by bacteria and their products

26.2002U2A1040 The main clinical symptoms of early adult periodontitis patients are ( )

A. Tooth displacement

B. Food impaction

C. Bleeding gums when brushing or eating

D. Gum recession

E. Loose teeth

27.2002U2A1041 Factors to be excluded in the diagnosis of rapidly progressive periodontitis do not include ( )

A. Whether there is a serious malocclusion

B. Whether the age is between puberty and 25 years

C. Whether or not incorrect orthodontic treatment has been received

D. Presence of significant local contributing factors, such as food impaction

E. Whether there is a concomitant systemic disease such as diabetes mellitus

28.2002U2A1042 Clinical features of juvenile periodontitis include ( )

A. It is more common in adolescents between adolescence and 25 years of age

B. Prediscriminate in the first permanent molars and upper and lower incisors

C. The lesion progresses rapidly, and the tooth is loose and displaced in the early stage

D. X-ray showing arc bone resorption of the first permanent molar

E. All of the above

29.2002U2A1043 Localized juvenile periodontitis is ( )

A. Maxillary incisors

B. Mandibular incisors

C. Premolars and upper and lower incisors

D. First molars and upper and lower incisors

E. Second molars and upper and lower incisors

30.2014U2A2097 Female 20 years old, complaining of masticatory weakness, periodontal pocket 6mm on the left and right upper 6 buccal sides of oral examination, loose II.°, general oral hygiene, calculus I°, probable diagnosis ( )

A. Periodontitis in adolescence

B. Chronic gingivitis

C. Simple periodontitis

D. Compound periodontitis

E. None of the above

31.2014U2A2106 Male, 43 years old, bleeding from tooth brushing for 2 years, soreness from hot and cold stimuli, no spontaneous pain, nocturnal pain, and weak occlusion of lower anterior teeth for half a year. Examination: the tartar index is 3, the gingiva is congested and swollen, the lower anterior teeth are loose II., the gingival recession is 2mm, the periodontal probing depth of the upper and lower molars is 4~5mm, and the diagnosis should be ( )

A. Gingivitis

B. Chronic periodontitis

C. Rapidly progressive periodontitis

D. Pulpitis

E. Diffuse juvenile periodontitis

32.2014U2A2107 Male, 38 years old. Bleeding from the whole mouth, weakness to chew. And have just completed thorough periodontal system treatment. No significant systemic disease. Oral examination: the depth of the periodontal pocket was increased compared with that before treatment, and the X-ray showed that alveolar bone resorption had developed significantly. Good oral hygiene. Initial diagnosis is ( )

A. Juvenile periodontitis

B. Periodontitis associated with diabetes

C. Periodontitis in adults

D. Rapidly progressive periodontitis

E. Refractory periodontitis

33.2014U2A2108 Male, 17 years old. complained of tooth bleeding and chewing weakness for more than 1 month. Oral examination: incisors and first molars are loosened I.°, incisors are displaced labially, periodontal pockets are 5~6mm, periodontal pockets of first molars are 6mm, plaque index and gingival index are 1, and gingival bleeding is probed. Initial diagnosis is ( )

A. Periodontitis in adults

B. Juvenile periodontitis

C. Rapidly progressive periodontitis

D. Adolescent gingivitis

E. Prepubertal periodontitis

34.2014U2A1016 Juvenile periodontitis is characterized by ( )

A. More common in adolescent girls

B. Lots of tartar

C. Severe gum inflammation

D. It is more common to have fangs

E. Alveolar bone is mostly absorbed horizontally

35.2014U2A1017 The perceived symptoms of patients in the early stage of juvenile periodontitis are often ( )

A. Weakness in tooth chewing

B. Significant bleeding from the gums

C. Significant redness and swelling of the gums

D. Bad breath

E. Periodontal pocket discharge

GPT-4

1. D. Most patients have neutrophil dysfunction
2. E. All of the above
3. E. All of the above
4. E. Deeper periodontal pockets on the adjacent surface
5. B. Autosomal dominant inheritance
6. B. The child is intellectually disabled but has normal physical development
7. B. In early patients, plaque and tartar are scarce, and the degree of periodontal tissue destruction is not proportional to the amount of local irritants
8. A. Upper incisor labial displacement
9. E. Early appearance of loose and displaced teeth
10. A. Aggregator actinomyces
11. C. Juvenile periodontitis
12. E. Maxillary anterior teeth
13. B. Rapidly progressive periodontitis
14. D. Most alveolar bones are moderately and severely absorbed
15. B. Rapidly progressive periodontitis
16. B. Resorption of alveolar bone
17. B. Juvenile periodontitis
18. A. 1~2 months
19. A. Visiting ≥ 3 dishes
20. A. Both localized and systemic damage
21. C. Localized juvenile periodontitis
22. D. Porphyromonas gingivals
23. E. All of the above examinations are required
24. B. Candida albicans
25. A. Infectious diseases with multifactorial pathogenesis
26. C. Bleeding gums when brushing or eating
27. E. Whether there is a concomitant systemic disease such as diabetes mellitus
28. E. All of the above
29. D. First molars and upper and lower incisors
30. A. Periodontitis in adolescence
31. B. Chronic periodontitis
32. D. Rapidly progressive periodontitis
33. B. Juvenile periodontitis
34. A. More common in adolescent girls
35. B. Significant bleeding from the gums

**Chapter 11 Associated Lesions of Periodontitis**

1.2009U2A1053 Pathogenesis factors of periodontal abscess do not include ( )

A. Purulent inflammation of the wall of the periodontal pocket develops deeply, and pus cannot drain out of the pocket

B. The periodontal pocket is tortuous and involves multiple tooth surfaces, and the purulent exudate cannot be drained smoothly

C. Scaling, scaling is rough, pushing the tartar fragments deep into the periodontal pocket

D. The foreign body penetrates the interdental papilla, causing purulent inflammation

E. Perforation of the medullary floor or root canal wall during root canal treatment, causing purulent inflammation

2.2009U2A1067 Pathogenesis associated with root bifurcation lesions includes ( )

A. Plaque and tartar

B. Occlusal trauma

C. Anatomical morphology of the tooth root

D. Spread of pulp infection

E. All of the above

3.2016U2A1010 Key points for differentiating periodontal abscess from alveolar abscess do not include ( )

A. Presence or absence of deep periodontal pockets

B. Pulp viability

C. Percussion

D. X-ray findings

E. Degree of gum inflammation

4.2008U2A1010 Main causative factors of root bifurcation lesions ( )

A. Anatomical morphology of the tooth root

B. Trauma

C. Plaque

D. Enamel protrusion in the neck of the tooth

E. Pulp infection of molars spreads through the accessory root canal at the base of the pulp chamber to the root bifurcation zone

5.2000U2A1007 The following periodontal diseases are caused by local irritants, except ( )

A. Simple gingivitis

B. Periodontitis in adults

C. Multiple gingival abscesses

D. Gingival papillitis

E. hypertrophic gingivitis

6.2000U2AN125 female, 60 years old, gum swelling and pain for 2 weeks, had swelling and pain 1 year ago, but no treatment. Examination: The gingiva on the lower left 6 buccal sides is swollen, there is a fistula, the fistula points to the apical direction, and there are periodontal pockets of 5~6mm in the center of the buccal side, distal middle and lingual side. An important test to confirm the diagnosis is ( )

A. Bleeding on probing

B. Tooth looseness

C. X-rays

D. Probing of root forks

E. Probe the subgingival calculus

7.2000U2AN126 female, 60 years old, gum swelling and pain for 2 weeks, had swelling and pain 1 year ago, but no treatment. Examination: The gingiva on the lower left 6 buccal sides is swollen, there is a fistula, the fistula points to the apical direction, and there are periodontal pockets of 5~6mm in the center of the buccal side, distal middle and lingual side. An important test to confirm the diagnosis is to take x-rays, and the most likely diagnosis of the tooth is ( )

A. Pulpitis

B. Chronic periodontitis

C. Marginal gingivitis

D. Root bifurcation lesions

E. Combined periodontal-pulp lesions

8. 2015U2A2070 A 25-year-old patient with poor oral hygiene, the first permanent molars in the left lower left had a pulp-periodontal joint lesion, severe pain, but no loosening of the affected tooth. In order to eliminate the pain and restore health, the first measure taken is to open the marrow and drain the marrow to stop the inflammation from spreading. The above treatment measures are tertiary prevention. At the same time, it should also be taken ( )

A. Desensitization of the first permanent molar in the lower left

B. Filling of the lower left first permanent molar

C. Periodontal treatment of the lower left first permanent molar

D. Occlusion of the first permanent molar in the lower left

E. Extraction of the first permanent molar in the lower left

9.2015U2A2077 male, 45 years old, after treatment by experienced doctors, bleeding was still detected at the root bifurcation of the right upper 6 cheeks. Causes affecting the prognosis of root bifurcation lesion treatment, except ( )

A. The root fork entry is too small

B. Enamel process

C. Patient is not flossing

D. Incomplete removal of plaque tartar

E. Most molars have root bifurcation close to the cementum of the enamel tooth

10.2015U2A2078 A patient, complaining: swollen and painful gingiva in the right upper posterior tooth for 2 days. Examination: oval swelling of the gingiva on the right upper 6 cheeks, fluctuating sensation (+) PD: 7mm, pus leakage in the probing bag, and normal electrical viability measurement. The most likely diagnosis is ( )

A. Acute alveolar abscess

B. Acute multiple periodontal abscess

C. Retrograde pulpitis

D. Acute periodontal abscess

E. Root bifurcation lesions

11.2004U2A1064 The local reasons affecting the treatment effect of root bifurcation lesions are mainly ( )

A. Anatomical features

B. Bacteria are more virulent

C. High number of bacteria

D. Occlusal surface morphology

E. The number of roots is high

12.2010U2A1037 The following methods of treatment of acute periodontal abscess are inappropriate ( )

A. Abscess incision

B. Gingival flap surgery

C. Anti-inflammatory and analgesic

D. Adjust the occlusion

Topical irrigation of periodontal pockets

13.2013U2A1006 IV. Root bifurcation lesions, which treatment is not needed ( )

A. Gingivectomy

B. Periodontal flap surgery

C. Cleansing

D. Curettage

E. Root planing

14.2013U2A2099 Adult male, diagnosed with retrograde pulpitis, whether the affected tooth can be preserved mainly depends on ( )

A. Patient's financial status

B. Efficacy of endodontic therapy

C. Patient's general condition

D. Oral hygiene habits

E. Extent of periodontal lesions and prognosis

15.2013U2A2101 female, 47 years old, has been diagnosed with the third degree root bifurcation lesion of the left lower first molar, loose (-), generally not used ( )

A. Guided tissue regeneration

B. Root splitting

C. Tunneling

D. Pocket wall resection

E. Root reduction flap surgery

· Factors for the development of periodontal abscess do not include (E)

· Pathogenesis factors associated with root bifurcation lesions include (E)

· Points to distinguish periodontal abscess from alveolar abscess do not include (E)

· Main causative factors of root bifurcation lesions (E)

· The following periodontal diseases are caused by local irritants, with the exception of (E)

· An important check to be made is (C)

· The most likely diagnosis of this tooth is (E)

· At the same time, it should also be taken (C)

· Causes affecting the prognosis of root bifurcation lesion treatment, with the exception of (E)

· The most likely diagnosis is (D)

· The main local reasons affecting the therapeutic effect of root bifurcation lesions are (A)

· The following methods for the management of acute periodontal abscess are inappropriate (B)

· IV. Root bifurcation lesions, which treatment is least needed (E)

· The retention of the affected tooth depends mainly on (B)

· Generally not used (E)

**Chapter 15 Basic Periodontal Treatment**

1.2009U2A1040 The purpose of root surface planarization is ( )

A. Removal of root surface infected cementum

B. Removal of residual tartar

C. Remove granulation tissue from the inner wall of the bag

D. Remove subgingival plaque

E. All of the above

2.2009U2A1041 No need to do after cleaning ( )

A. Use a pointed probe to check whether the tartar has been removed

B. Rinse the periodontal pocket with 3% hydrogen peroxide solution

C. Intragingival sulcus application

D. Immediate root desensitization

E. Check the gums for damage

3.2009U2A1043 The focus of the basic treatment of periodontal disease is ( )

A. Medication

B. Occlusal adjustment

C. Plaque control

D. Loose tooth fixation

E. Treatment of food impaction

4.2009U2A1049 Selective grinding method in food impaction treatment is suitable for ( )

A. All vertical food impaction

B. Partial vertical food impaction

C. All horizontal food impaction

D. Partial horizontal food impaction

E. B+C

5.2009U2A1057 Methods that do not belong to self-control plaque are ( )

A. Brush your teeth

B. Gargle

C. Use of dental floss

D. Cleanliness

E. Use of toothpicks

6.2009U2A1059 Wrong grinding method ( )

A. Generally, the early contact area in the center is ground first, and the functional cusp is not easily polished

B. The grinding of the side should take into account both the working side and the non-working side

C. The grinding work should be completed at one time to reduce the number of patient visits

D. At the end of grinding, the tooth surface must be polished with a hard rubber wheel to reduce the accumulation of plaque

E. For those who have allergic symptoms due to the exposure of dentin by grinding, desensitization treatment should be performed

7.2009U2A1060 Treatment that can eliminate periodontal pocket inflammation and increase the level of attachment ( )

A. Oral health education

B. Supragingival cleansing

C. Cleansing and root leveling

D. Periodontal pocket medication

Periodontal irrigation

8.2009U2A1061 Plaque control index, if it can be achieved, it is basically controlled ( )

A. Less than 10 per cent

B. Less than 15 per cent

C. Less than 20 per cent

D. Less than 25 per cent

E. Less than 30 per cent

9.2009U2A1066 Which is correct for the description of the grinding method ( )

A. Ground the median early contact point first

B. Don't change too much at once

C. Loose teeth with inflammation at the root tip should be reground after the inflammation is eliminated

D. Restore the spherical shape of the tooth as much as possible

E. All of the above

10.2009U2A1077 What factors should be considered first in the etiology of periodontitis to guide treatment ( )

A. Genetic factors

B. Endocrine factors

C. Immunological factors

D. Plaque factor

E. Nutritional factors

11.2016U2A1005 When there is early contact between median occlusion and non-median occlusion, it should be abrasional ( )

A. Bevel of the buccal tip of maxillary molars

B. Early contact area of the lingual fossa or fossa

C. Early contact cusps

D. Incisal margin of the inferior anterior teeth of early contact

E. C+D

12.2016U2A1009 Which of the following brushing methods is most effective in removing plaque near the gingival sulcus and in the adjacent space ( )

A. Light brushing

B. Horizontal fibrillation method

C. Vertical rotation method

D. Re-brushing method

E. Horizontal brushing method

13.2009U2A1079 Temporary splint fixation precautions are wrong ( )

A. The original position of the teeth should be maintained without traction

B. Suitable for those with intact dentition

C. After ligation and fixation, adequate occlusion should be performed with or without occlusal trauma

D. Strengthen oral hygiene guidance and strictly control bacteria

E. No need to fix your teeth to bite hard objects

14.2016U2A1014 Commonly used plaque display agent ( )

A. Magenta solution

B. Gentian violet liquid

C. Iodized oil

D. Methylene blue

E. Sodium fluorescein

15.2016U2A1016 Periodontal system treatment is ( )

A. Adjust the occlusion

B. Upper and lower gingival cleansing

C. Root surface leveling

D. Plaque control

E. Administered therapy

16.2008U2A2078 Anterior teeth have early contact in median occlusion, anterior occlusion coordination, should be abrasional modification ( )

A. Upper cusps

B. Lower cusps

C. Upper lingual fossa

D. Labial surface of the lower teeth

E. Upper tongue bevel

17.2008U2A1026 The gargle that can color the mucous membrane is ( )

A. Dobell′s

B. 1％NaHCO3

C. Saline

D. Chlorhexidine

E. 3% hydrogen peroxide (diluted 2-fold)

18.2004U2A1062 When cleaning with hand instruments, attention should be paid to ( )

A. The suitability of the instrument to the tooth surface and soft tissue

B. The working angle between the working end and the tooth surface

C. Use the push and pull of the wrist

D. The direction of cleaning is vertical, horizontal, and oblique

E. All of the above

19.2004U2A1070 Commonly used plaque display agent ( )

A. Basic magenta

B. Gentian violet

C. Red mercury

D. Congo red

E. Iodine tincture

· The purpose of root planarization is (E)

· Not required after cleansing (D)

· The focus of basic treatment for periodontal disease is (C)

· Selective grinding in the treatment of food impaction is suitable for (E)

· Methods that do not belong to self-control plaque are (D)

· Errors in the grinding method (C)

· Treatment that eliminates periodontal pocket inflammation and increases the level of attachment (C)

· Plaque control indicators, if any, are basically controlled (B)

· Which is correct for describing the grinding method (E)

· What factors should be considered first in the etiology of periodontitis to guide treatment (D)

· When there is early contact between median and non-median occlusions, abrasion should be modified (E)

· Which of the following brushing methods is most effective at removing plaque near and adjacent gingival sulcus (C)

· Precautions for temporary splint fixation are wrong (D)

· Commonly used plaque display agents are (E)

· Throughout the treatment of the periodontal system, (D)

· Should be abrasion(D)

· The gargle that stains the mucous membranes is (D)

· When cleaning with manual instruments, attention should be paid to (E)

· Commonly used plaque display agents (B)

**Chapter 17 Surgical Treatment of Periodontal Disease**

1.2009U2A1062 It is strictly forbidden to touch the tooth surface and root surface when using electric knife to cut the gingiva

A. Can cause tissue loss

B. Can cause osteonecrosis

C. Can cause soft tissue necrosis

D. Can irritate the pulp and cause pulp lesions

E. Can cause alveolar osteonecrosis

2. The concentration of citrate used for root treatment in 2009U2A1072 GTR surgery is ( )

A. 10% saturated citrate

B. 20% saturated citrate

C. 30% saturated citrate

D. 50% saturated citrate

E. 70% saturated citrate

3.2016U2A1004 Which of the following is not an absorbable GTR biocompatible membrane material ( )

A. Polymerized collagen membrane

B. PTFE

C. Polylactic acid film

D. Lactic acid-acetic acid copolymer membrane

E. All of the above

4.2016U2A1024 The best site for vertical incision in periodontal surgery should be selected in ( )

A. Midpoint of buccal/lingual gingiva adjacent to a healthy tooth

B. Near and distal axial angles of adjacent healthy teeth

C. Adjacent to the gingival papilla of a healthy tooth

D. The most prominent root of the adjacent healthy tooth

E. Bone margins in the lesion area

5.2008U2A1003 The main effect of the plugging agent does not include ( )

A. Protective

B. Bactericidal effect

C. Haemostasis

D. Analgesic

E. Immobilization

6.2000U2A1009 The incision that can completely remove the epithelium and infected tissue on the inner wall of the bag during periodontal flap surgery is ( )

A. Interdental incisions

B. Intrasulcus incision

C. External bevel incision

D. Internal bevel incision

E. Longitudinal incision

7.2004U2A1058 Purpose of pre-gingivectomy bag ( )

A. Determine the direction of the incision

B. Understand periodontal pocket depth

C. Know the gum thickness

D. Determine the incision location

E. All of the above

8.2004U2A1061 The ideal way for tissue to heal after periodontal surgery is ( )

A. New Attachment

B. Re-attachment

C. Long epithelial union

D. General Repairs

E. Bony union

9.2004U2A1065 Bone resorption of distal buccal root of maxillary first molar is more than 2/3, bone resorption of mesial buccal root and palatal root is about 1/3, distal and buccal root bifurcation lesion III. degree, selectable ( )

A. Amputation of the meso-buccal root

B. Truncation of the distal buccal root

C. Hemitomy

D. Guided tissue regeneration

E. Alveolar osteoplasty

10.2004U2A1067 During the healing process of periodontal surgery, the cells that preferentially occupy the root surface are usually ( )

A. Alveolar bone marrow cells

B. Gingival connective tissue cells

C. Gingival epithelial cells

D. Periodontal ligament cells

E. Odontoblasts

11.2004U2A1069 The advantages of internal bevel incision are ( )

A. Direct excision of the epithelial and inflammatory tissue on the inner wall of the bag

B. Preservation of attached gingiva on the surface of the periodontal pocket

C. Less postoperative gingival recession

D. The gingival shape is better after healing

E. All of the above

12.2013U2A1010 During periodontal surgery, if the buccal and lingual gingival tissues are of equal height and tension, and the suture needle can pass through both gingival flaps at the same time, the method used is ( )

A. Interdental intermittent sutures

B. Interdental "figure 8" suture

C. Suspension suture

D. Horizontal mattress suture

E. Anchor suture

13. 2012U2A1009 Indications for guided tissue regeneration, except ( )

A. II. Root bifurcation lesions

B. IV. Root bifurcation lesions

C. Hypopalasal pouch

D. Three-wall sub-bone pocket

E. Angular bone resorption

14.2012U2A1011 Which of the following should not be treated for gingivectomy ( )

A. Hyperplasia of gingival tissue

B. Chronic abscess in suprabony pockets

C. Posterior teeth attach to moderately deep supraosseous pockets where the gingiva is too narrow

D. Wisdom tooth pericrown blind pocket formation

E. Gingival tumors

1. It is strictly forbidden to touch the tooth surface and root surface when using an electric knife to cut the gingiva for (B)
2. The concentration of citrate used for root treatment in GTR surgery is (C)
3. Which of the following is NOT an absorbable GTR biocompatible membrane material (B)
4. The best site for the vertical incision of periodontal surgery should be selected in (B)
5. The main effects of the stoppers do not include (C)
6. The incision that can completely remove the epithelium and infected tissue in the inner wall of the pocket during periodontal flap surgery is (E)
7. Purpose of pre-gingivectomy bag fixing (E)
8. The ideal way for tissue to heal after periodontal surgery is (B)
9. Bone resorption of more than 2/3 of the distal buccal root of maxillary first molar, bone resorption of about 1/3 of the mesial buccal root and palatal root, and III. degree of distal and buccal root bifurcation lesions, can be selected (A)
10. During the healing process of periodontal surgery, the cells that preferentially occupy the root surface are usually (C)
11. The advantages of the internal bevel incision are (E)
12. During periodontal surgery, if the buccal and lingual gingival tissues are of equal height, the tension is the same, and the needles can pass through both gingival flaps at the same time.
13. Indications for guided tissue regeneration, with the exception of (B)
14. Which of the following should not be treated for gingivectomy (C)

**Oral mucosal diseases**

**Chapter 2 Infectious Diseases of the Oral Mucosa**

1. 2001U1A1014 Acyclovir antiviral ability in the correct order is ( )

A. HSV-2, HSV-1, varicella-zoster virus, Epstein-Barr virus

B. HSV-1, HSV-2, varicella-zoster virus, Epstein-Barr virus

C. HSV-1, HSV-2, Epstein-Barr virus, varicella-zoster virus

D. HSV-2, HSV-1, Epstein-Barr virus, varicella-zoster virus

E. HSV-1, Epstein-Barr virus, HSV-2, varicella-zoster virus

2. 2010U2A2104 female, 50 years old, white patches of buccal mucosa near the corners of the mouth for nearly l years, can not be wiped off. Histology shows epithelial hyperplasia with neutrophil infiltration and scattered microabscesses, PAS-positive hyphae perpendicular to the epithelium in the keratotic layer, and chronic inflammatory cell infiltration in connective tissue. The most likely pathological diagnosis is ( )

A. Vitiligo

B. Erythema

C. Oral tuberculitis

D. Candidiasis

E. Chronic discoid lupus erythematosus

3.2009U2A1022 Typical clinical manifestations of herpes zoster in the face and mouth ( )

A. Clusters of erythema, blisters are symmetrically distributed along the trigeminal nerve and are painful

B. Clusters of erythema, blisters along one side of the trigeminal nerve, severe pain

C. Clusters of erythema and blisters are distributed along one side of the trigeminal nerve, and in severe cases, they can be distributed beyond the midline to the contralateral trigeminal nerve, and the pain is severe

D. Erythema appears on the face, along one side of the trigeminal nerve, followed by blisters, secondary infection and pustules, and finally crusting, leaving scars after scabbing off

E. None of the above

4.2001U3A1018 Oral tuberculosis anti-tuberculosis chemotherapy regimen medium and long-term (standard) chemotherapy drugs do not include ( )

A. Isoniazid

B. Streptomycin

C. Rifampicin

D. Ethambutol

E. p-Aminosalicylic acid

5. 2009U2A1027 is not an infectious disease ( )

A. Herpes zoster

B. Recurrent herpetic stomatitis

C. Oral tuberculosis

D. Snow mouth disease

E. Bullous pemphigoid

6.2009U2A1033 Herpes simplex virus can be transmitted by the following routes, except ( )

A. Skin

B. Cornea

C. Mucous membranes

D. Digestive tract

E. Respiratory

7.2009U2A1038 Candida angular cheilitis in elderly patients is more often related to which of the following ( )

A. Older people are susceptible to systemic diseases

B. Older people have lower resistance

C. Long-term use of various medications, especially antibiotics

D. The vertical distance of the occlusion is shortened, and saliva is easy to overflow from the corners of the mouth and is in a long-term wet state

E. None of the above

8.2009U2A1039 Primary herpetic gingivostomatitis has a course of ( )

A. 2~5 days

B. 4~6 days

C. 7~10 days

D. 9~11 days

E. 9~13 days

9.2000U2A1003 Oral mucosa and skin manifestations of dense herpes with unilateral distribution along the nerve are ( )

A. Recurrent herpetic stomatitis

B. Acute herpetic gingivostomatitis

C. Herpangina

D. Herpetiform aphthous ulcers

E. Shingles

10.2000U2A1004 Oral candidiasis often occurs in the following situations, except ( )

A. Long-term use of broad-spectrum antibiotics

B. Long-term use of immunosuppressants

C. Chronic wasting disease

D. Chronic stress

E. Candida albicans itself is more virulent

11.2000U2A1006 Herpetic gingivostomatitis is more common in ( )

A. Infants and toddlers aged 6 months ~ 2 years old

B. Preschoolers

C. Adolescents

D. Middle-aged

E. Older persons

12.2000U2AN122 female, 2.5 years old. High fever for 3 days, canker sores for 2 days. Crying, salivating, refusing to eat. Physical examination found that the child's gingiva was red and swollen all over the mouth, and dense needle-sized transparent blisters could be seen on the palate mucosa, some of which had broken into superficial ulcers, and the surrounding mucosa was congested and edema was extensive. The most likely diagnosis in this case is ( )

A. Thrush

B. Foot-and-mouth disease

C. Mouth sores in Belleson

D. Stomatitic aphthous ulcers

E. Herpetic gingivostomatitis

13.2000U2AN123 female, 2.5 years old. High fever for 3 days, canker sores for 2 days. Crying, salivating, refusing to eat. Physical examination found that the child's gingiva was red and swollen all over the mouth, and dense needle-sized transparent blisters could be seen on the palate mucosa, some of which had broken into superficial ulcers, and the surrounding mucosa was congested and edema was extensive. The most likely diagnosis in this case is herpetic gingivostomatitis, and depending on the clinical presentation of the disease, the preferred ancillary tests are ( )

A. Urinalysis

B. Routine blood test and differential examination

C. Biopsy

D. T cell subset assay

E. Liver and kidney function

14.2000U2AN124 female, 2.5 years old. High fever for 3 days, canker sores for 2 days. Crying, salivating, refusing to eat. Physical examination found that the child's gingiva was red and swollen all over the mouth, and dense needle-sized transparent blisters could be seen on the palate mucosa, some of which had broken into superficial ulcers, and the surrounding mucosa was congested and edema was extensive. The most likely diagnosis of this case is herpetic gingivostomatitis, and the measures that are not suitable for treatment in this case are ( )

A. Take breaks to strengthen your nutrition

B. Systemic use of antiviral drugs

C. Nebulized inhalation with topical corticosteroids

D. Supplement with plenty of vitamins

E. Traditional Chinese Medicine

15. 2012U2A1001 Which of the following diseases generally does not leave scars after healing ( )

A. Periglandular aphthous ulcers

B. Herpes zoster

C. Fibrosis of the oral mucosa

D. Genital ulcers in Behcet's disease

E. Pressure ulcers

16.2012U2A1002 The following statements about candidal cheilitis are incorrect ( )

A. Candidal angular cheilitis is usually bilateral

B. Characterized by wet white erosions

C. Angular cheilitis in older patients is often associated with shortened vertical occlusal distance

D. It often occurs in children during the cold winter months

E. Often complicated by glossitis, cheilitis, scrotitis

17.2004U2A1019 Regarding varicella-zoster virus, the following correct statement is ( )

A. Attack T lymphocytes

B. Violation of the trigeminal nerve

C. Causes submucosal fibrosis

D. Low serum antibody titers after infection

E. Increase the chance of infection with other viruses

18.2004U2A1027 The following diseases can cause papillae atrophy, except ( )

A. Lichen planus

B. Sjögren's syndrome

C. Discoid lupus erythematosus

D. B vitamin deficiency

E. Oral Candida infection

19.2004U2A1037 The clinical features of chronic proliferative candidiasis are ( )

A. White papules with cross-reticular streaks on the oral mucosa, and hyperemia of the basal mucosa

B. White curd-like spots or patches on the oral mucosa without obvious symptoms

C. Well-defined erythema can be seen in the oral mucosa, and tongue papillae atrophy with erosion and chapping of the mucosa of the oral corners

D. Dense white plaques appear on the mucosa on the medial side of the mouth corners, with a rough surface, accompanied by bilateral redness and chapping of the corners of the mouth

E. A diamond-shaped, filamentous papillary atrophy zone appears in the posterior central mucosa of the dorsal tongue, and nodular hyperplasia may appear on the surface, which is hard on examination but soft at the base

20.2010U2A1012 Varicella-zoster virus can cause in adults and the elderly ( )

A. Chickenpox

B. Hand-foot-mouth disease

C. Herpangina

D. Herpes zoster

E. A+D

21.2010U2A1016 ( )

A. Lupus vulgaris

B. Snow mouth sores

C. Herpes zoster

D. Pemphigus vulgaris

E. Hand-foot-mouth disease

22.2010U2A1017 Antivirals that are highly selective against herpes simplex virus types I and II are ( )

A. Ribavirin

B. Interferon

C. Polymyocytes

D. Acyclovir

E. Thymosin

23.2010U2A1023 An important item used to distinguish between herpetiform aphthous ulcers and herpetic stomatitis is ( )

A. Good or bad prognosis

B. Lesion size

C. Pain level

D. Presence or absence of skin lesions

E. Acupuncture reaction

24.2010U2A1024 Which of the following drugs should not be used in patients with herpes zoster with serious complications ( )

A. Acyclovir

B. Interferon

C. Prednisone

D. Cimetidine

E. Transfer factors

25. The common sites of recurrent infection with herpes simplex virus type I in 2010 are ( )

A. Buccal mucosa

B. Mucosa dorsal of the tongue

C. Mucosa of the tongue and lateral border of the tongue

D. Labial mucosa

E. Lip redness and peripheral skin

26.2012U2A2102 Female, 40 years old, erythema around the lips 1 week ago, burning itching, clusters of needle-tip blisters appeared one day later, scabbed after the blisters broke and erosion one day later, there was a history of cold before the illness, probable diagnosis ( )

A. Angular cheilitis

B. Discoid lupus erythematosus

C. Cold sores

D. lichen planus

E. Thrush

27.2010U2A1028 Membranous stomatitis is caused by ( )

A. Fusophilia

B. Caused by mycobacteria

C. Coxsackievirus M

D. Herpes simplex virus type I

E. Staphylococcus aureus and hemolytic streptococcus

28.2010U2A1029 Oral herpes simplex virus infection route ( )

A. Droplets

B. Saliva

C. Herpes fluid

D. Birth canal

E. All of the above

29.2010U2A1030 The cause of primary herpetic stomatitis is infection ( )

A. HSVI

B. HSVⅡ

C. HIVI

D. HIVⅡ

E. None of the above

30.2002U2A1012 Herpetic recurrent aphthous ulcer is characterized by ( )

A. Generally, there are 1~5 ulcers with a diameter of 5~10mm, which mostly occur in the lip and cheek mucosa

B. It is mostly a single large ulcer, with a diameter of more than 1 cm, and mostly occurs in the cheeks, soft palate, etc

C. The number and diameter vary greatly, and obvious local stimuli can be seen

D. The ulcer is single, long-lasting, and excavating

E. Multiple ulcers can reach dozens, with a diameter of 1~2mm, and can also be fused into patches, mostly seen in the mucosa of the floor of the mouth and tongue and abdomen

31.2002U2A1013 The incidence of neonatal snow mouth disease is ( )

A. 0.02

B. 0.03

C. 0.04

D. 0.05

E. 0.06

32.2002U2A1014 Among the following infectious oral mucosal diseases, the clinical characteristics of pseudomembrane lesions are ( )

A. Herpes simplex

B. Herpes zoster

C. Hand-foot-mouth disease

D. Coccal stomatitis

E. Oral tuberculosis

33.2002U2A1017 The following ulcers will leave scars after healing ( )

Aphthous ulcers

B. Severe aphthous ulcers

C. Herpetiform aphthous ulcer

D. Behcet's disease canker sores

E. All of the above

34.2012U2A2103 A 40-year-old female who had clusters of pinhead-sized blisters on her lower lip and around her lips after a cold, crusted over after bursting, and was locally itching and painful. The patient's possible illness is ( )

A. Cold sores

B. Chronic cheilitis

C. Herpetic stomatitis

D. Fixed drug eruption

E. Recurrent aphthous ulcers

35.2012U2A2104 Xie, female, 34 years old, 2 days after fever, felt itching and burning pain in the left side of the maxillary gum, and soon found that the left maxillary gum was red and swollen, and clusters of transparent blisters the size of millet grains appeared on the surface, which became small ulcers after bursting. Which of the following treatments is the most important ( )

A. Keep your mouth clean

B. Intramuscular injection of penicillin

C. Oral antipyretic analgesics

D. Oral administration of acyclic guanosine or radix of banyan root

E. Bed rest and multivitamin supplementation

36.2012U2A2105 Baby male, 5 days old, crying and refusing to eat, the oral mucosa appears slightly convex soft white dots, wiped off to reveal the bleeding surface, probable diagnosis ( )

A. Early eruption of baby teeth

B. Vitiligo

C. Infantile snow mouth disease

D. Keratinized epithelial beads

E. None of the above

37.2012U2A2106 Infant, female, 7 days old, crying and refusing to eat. Slightly convex soft white dots appear in the oral mucosa, which can reveal bleeding spots after wiping, and the probable diagnosis is ( )

A. Vitiligo

B. Early deciduous teeth

C. Candidiasis albicans

D. Keratinized epithelial beads

E. Herpetic stomatitis

38.2013U2A2079 Baby girl, 7 days old. The mother complained of irritability, crying, and refusal to eat. Oral examination showed scattered snow-white soft spots on the left cheek and tip of the tongue, and obvious basal mucosal hyperemia. With a little force, it can be wiped off, leaving a bleeding erosion. Initial diagnosis is ( )

A. Acute pseudomembranous candidal stomatitis

B. Acute erythematous candidal stomatitis

C. Chronic hypertrophic candidal stomatitis

D. Candida chronic erythematous stomatitis

E. Candidal cheilitis

39.2013U2A2080 Infant girl, 2 months old, white curd-like spots and plaques on the oral mucosa that can be wiped, redness, crying, and difficulty in breastfeeding of the basal mucosa, should be suspected as ( )

A. Thrush

B. Recurrent canker sores

C. Herpetic gingivostomatitis

D. Coccal stomatitis

E. Crohn disease

40.2013U2A2081 Male, 60 years old, suffering from diabetes for 10 years. In the past 2 weeks, the mouth has been dry, the thirst has worsened, the corners of the mouth have been cracked, bleeding, and there is a significant burning sensation when eating. Bilateral redness of the corners of the mouth with fissure, mandibular and mandibular restoration, and redness of the mucosa in the denture support area, which is consistent with the denture. A large number of hyphae can be seen on the surface smear of the denture. Which method should be selected for clinical treatment is the most effective ( )

A. Gargle with 3%~5% sodium bicarbonate solution every day

B. Maintain oral hygiene and pay attention to the isolation and disinfection of tableware

C. Replacement or relining of dentures

D. Topical application of nystatin or dacnine cream in the mouth

E. Supplement with iron, vitamin B12 and folic acid

41.2013U2A2082 Male, 10 years old. The left tongue margin ulcerated for more than 3 months. The earliest ulceration was a small nodule that was painless, and the left submandibular lymph node was painful. Oral examination: 1 ulcer with a diameter of 1 cm can be seen on the left tongue margin, there is induration around it, there is a little purulent discharge at the base, dark red mulberry-like granuloma can be seen in the removal of exudate, and the edge of the ulcer is rat-like.

A. Tuberculin test

B. Erythrocyte sedimentation rate

C. Concentrated culture of collected bacteria

D. Biopsy histopathological examination

E. All of the above

42.2013U2A2083 Male, 35 years old. Pain in tongue ulceration for 1 week. 10 days ago, he had a "cold and fever" accompanied by multiple erosions in the mouth. Oral examination: an erosion surface with a diameter of 2cm can be seen in the center of the dorsum of the tongue, with obvious hyperemia and edema, a yellow-brown pseudomembrane on the surface, which is easy to wipe off, leaving a bloody erosion surface, obvious pain, and non-specific halitosis. Probable( )

A. Oral lichen planus

B. Acute pseudomembranous candidal stomatitis

C. Recurrent herpetic stomatitis

D. Recurrent severe aphthous ulcers

E. Coccal stomatitis

43.2013U2A2084 A 22-year-old female presents with swollen and painful gums with spontaneous bleeding. The patient was examined for poor oral hygiene, scaltar grade III, and obvious halitosis. The gingiva of the whole mouth is red and swollen, the gingival papillae on the left and right lower 12 lips are knife-shaped, the surface is covered with gray-white pseudo-membrane, the necrotic membrane is removed, and the base is a fresh wound that is easy to bleed. The case is intended to be diagnosed as ( )

A. Acute infectious stomatitis

B. Radiation stomatitis

C. Necrotizing gingivostomatitis

D. Herpetic gingivostomatitis

E. Leukemia

44.2013U2A2085 An 8-year-old male presented with 1 week of necrosis and ulceration of the gums and oral mucosa with high fever for 10 days. The initial diagnosis was necrotizing gingivostomatitis. Which of the following drugs is not suitable for topical oral treatment ( )

A. 3% hydrogen peroxide

B. 1% gentian violet

C. 10% silver nitrate

D. Periodontal plug preparations

E. Nourishing Yin and Shengjisan or Ice Boron

45.2010U2A2104 Female, 50 years old, white patches of buccal mucosa near the corners of the mouth for nearly l years, which cannot be erased. Histology shows epithelial hyperplasia with neutrophil infiltration and scattered microabscesses, PAS-positive hyphae perpendicular to the epithelium in the keratotic layer, and chronic inflammatory cell infiltration in connective tissue. The most likely pathological diagnosis is ( )

A. Vitiligo

B. Erythema

C. Oral tuberculitis

D. Candidiasis

E. Chronic discoid lupus erythematosus

46.2005U2A1011 The cause of herpetic gingivostomatitis is ( )

A. Bacteria

B. Viruses

C. Fungi

D. Chlamydia

E. Rickettsia

47.2005U2A1012 The cause of gangrenous stomatitis may be ( )

A. Poor oral hygiene

B. Vitamin deficiencies

C. Local trauma

D. Viral infection

E. Bacterial infections

48.2004U2A1038 Recurrent herpetic stomatitis is generally common ( )

A. Lips

B. Buccal mucosa

C. Back of the tongue

D. Gingiva

E. Palatal mucosa

49.2004U2A1039 Oral tuberculosis pathogenic microorganisms are ( )

A. Viruses

B. Bacteria

C. Fungi

D. Chlamydia

E. Mycoplasma

50.2004U2A1020 The preferred drug for gangrenous stomatitis is ( )

A. Penicillin

B. Acyclic guanosine

C. Corticosteroids

D. nystatin

E. Postsamycin plus metronidazole

51.2002U2A1022 Systemic drug of choice for the treatment of acute herpetic gingivostomatitis ( )

A. Stomatitis powder

B. Acyclic guanosine

C. B vitamins

D. Corticosteroids

E. Broad-spectrum antibiotics

52.1999U2AN128 A 45-year-old female presents with redness and blistering of the skin on the left cheek and mucosa on the left tongue for 3 days, accompanied by severe pain. On physical examination, the body temperature was 38.5°C, dense patches of miliary transparent blisters were seen on the left facial skin and the left dorsum of the tongue and buccal mucosa, and congestive erythema was seen on the surrounding skin and mucosa. Assays: WBC 7.8×19/L, neutral 62%, lymphatic 34%. Diagnosis of herpes zoster. The main lesion sites in this case are ( )

A. Trigeminal nerve 1

B. Trigeminal nerve 2nd branch

C. 3rd branch of the trigeminal nerve

D. Facial nerve

E. Cervical nerve

53.1999U2AN129 A 45-year-old female presents with redness and blistering of the skin on the left cheek and mucosa on the left tongue for 3 days, accompanied by severe pain. On physical examination, the body temperature was 38.5°C, dense patches of miliary transparent blisters were seen on the left facial skin and the left dorsum of the tongue and buccal mucosa, and congestive erythema was seen on the surrounding skin and mucosa. Assays: WBC 7.8×19/L, neutral 62%, lymphatic 34%. Diagnosis of herpes zoster. The main differentiators between herpes zoster and herpes simplex are ( )

A. The etiology is different

B. Different prodromal symptoms

C. The duration of the disease varies

D. Clinical manifestations are different

E. Systemic symptoms vary

54.1999U2AN130 A 45-year-old female presents with redness and blistering of the skin on the left cheek and mucosa on the left tongue for 3 days, accompanied by severe pain. On physical examination, the body temperature was 38.5°C, dense patches of miliary transparent blisters were seen on the left facial skin and the left dorsum of the tongue and buccal mucosa, and congestive erythema was seen on the surrounding skin and mucosa. Assays: WBC 7.8×19/L, neutral 62%, lymphatic 34%. Diagnosis of herpes zoster. The most serious complications that can occur in this case are ( )

A. Pneumonia

B. Encephalitis

C. Conjunctivitis

D. Keratitis

E. Facial paralysis

55.1999U2AN132 A 45-year-old female presents with redness and blistering of the skin on the left cheek and mucosa on the left tongue for 3 days, accompanied by severe pain. On physical examination, the body temperature was 38.5°C, dense patches of miliary transparent blisters were seen on the left facial skin and the left dorsum of the tongue and buccal mucosa, and congestive erythema was seen on the surrounding skin and mucosa. Assays: WBC 7.8×19/L, neutral 62%, lymphatic 34%. Diagnosis of herpes zoster. Which of the various treatment measures you think is unreasonable at this point ( )

A. Oral antiviral drugs such as acyclic guanosine, etc

B. Injection of polymyocytes

C. Vitamin B₁₂ 500μg + vitamin B₁ 100mg intramuscularly once every other day

D. Skin lesions are coated with calamine lotion

E. Oral partial closure of dexamethasone + procaine

56.1999U2AN131 A 45-year-old female presents with redness and blistering of the skin on the left cheek and mucosa on the left tongue for 3 days, accompanied by severe pain. On physical examination, the body temperature was 38.5°C, dense patches of miliary transparent blisters were seen on the left facial skin and the left dorsum of the tongue and buccal mucosa, and congestive erythema was seen on the surrounding skin and mucosa. Assays: WBC 7.8×19/L, neutral 62%, lymphatic 34%. Diagnosis of herpes zoster. The patient was given intramuscular penicillin injection for 3 days, laser irradiation of local lesions and oral multivitamins and other measures in the hospital, and the symptoms improved, but did not disappear completely, especially the pain symptoms were still obvious. The reasons for poor efficacy are ( )

A. No supportive care was given

B. No injection of polymyocytes or transfer factors

C. Incorrect diagnosis

D. Topical anti-inflammatory gargle is not used

E. Carbamazepine or intramuscular vitamin B₁+ vitamin B₁₂ was not given

57.2004U2A1048 Herpes simplex virus susceptible sites do not include ( )

A. Oral cavity

B. Skin

C. Eye

D. Nervous system

E. Digestive tract

58.2016U2A1060 Diseases characterized by secondary ulcers of the oral mucosa are ( )

A. Chronic cheilitis

B. Vitamin A deficiency

C. Angular cheilitis

D. Herpes simplex

E. Map tongue

59.2017U2A1055 Herpes simplex virus nucleocapsid is made up of ( )

A. Composition of 160 shell particles

B. Composition of 161 shell particles

C. Composition of 162 shell particles

D. Composition of 163 shell particles

E. Composition of 164 shell particles

60.2017U2A1054 The following is wrong about the mode of transmission of oral herpes simplex virus ( )

A. Respiratory

B. Oral cavity

C. Nose

D. Conjunctiva of the eye

E. Digestive tract

61.2017U2A1053 The clinical manifestations of acute erythematous candida stomatitis are ( )

A. The mucosa of the lesion is congested with small soft spots as white as snow

B. The mucosa of the lesion appears as nodular or granular hyperplasia, or as a tightly fixed white keratin plaque

C. The mucosa in the lesion area is bright red edema, or a mottled pseudomembrane is seen

D. The mucosa in the lesion area is congested and erosive, the papillae on the back of the tongue are clumps and atrophied, and the surrounding tongue coating is thickened

E. Pearlescent white reticulation with hyperemia on the mucosa of the lesion

62.2017U2A1052 For elderly patients with chronic hypertrophic type in candidal stomatitis, the preferred diagnostic method is ( )

A. Saliva culture

B. Saliva and serum Candida antibody determination

C. Biopsy

D. Direct smear microscopy

E. Blood CEA assay

63.2020U1A1006 Typical clinical manifestations of acute pseudomembranous oral candidiasis are ( )

A. The dorsal mucosa of the tongue is bright red with tongue papilla atrophy

B. The mucosa in the denture support area is bright red

C. Medial triangle of the mouth with granular hyperplasia

D. The oral mucosa is congested with curd-like patches

E. The mucosa in the corner of the mouth is wet and white, erosive, and cracked

64.2020U1A1007 The typical clinical manifestations of chronic proliferative oral candidiasis are ( )

A. The dorsal mucosa of the tongue is bright red with tongue papilla atrophy

B. The mucosa in the denture support area is bright red

C. Congestion of the angular symphysis with granular hyperplasia

D. The oral mucosa is congested with curd-like patches

E. The mucosa in the corner of the mouth is wet and white, erosive, and cracked

The questions appear to come from exams in the field of dentistry and deal with the diagnosis, treatment, and etiology of oral diseases. Here are the answers to each question:

1. B. HSV-1, HSV-2, varicella-zoster virus, Epstein-Barr virus
2. D. Candidiasis
3. B. Clusters of erythema, blisters along one side of the trigeminal nerve, severe pain
4. B. Streptomycin
5. E. Bullous pemphigoid
6. E. Respiratory
7. C. Long-term use of various medications, especially antibiotics
8. C. 7~10 days
9. E. Shingles
10. E. Candida albicans itself is more virulent
11. A. Infants and toddlers aged 6 months ~ 2 years old
12. E. Herpetic gingivostomatitis
13. B. Routine blood test and differential examination
14. C. Nebulized inhalation with topical corticosteroids
15. A. Periglandular aphthous ulcers
16. D. It often occurs in children during the cold winter months
17. B. Violation of the trigeminal nerve
18. E. Oral Candida infection
19. C. Well-defined erythema can be seen in the oral mucosa, and tongue papillae atrophy with erosion and chapping of the mucosa of the oral corners
20. E. A+D
21. D. Pemphigus vulgaris
22. D. Acyclovir
23. D. Presence or absence of skin lesions
24. C. Prednisone
25. D. Labial mucosa
26. C. Cold sores
27. A. Fusophilia
28. E. All of the above
29. A. HSVI
30. E. Multiple ulcers can reach dozens, with a diameter of 1~2mm, and can also be fused into patches, mostly seen in the mucosa of the floor of the mouth and tongue and abdomen
31. B. 0.03
32. E. Oral tuberculosis
33. D. Behcet's disease canker sores
34. A. Cold sores
35. A. Keep your mouth clean
36. C. Infantile snow mouth disease
37. C. Candidiasis albicans
38. A. Acute pseudomembranous candidal stomatitis
39. A. Thrush
40. C. Replacement or relining of dentures
41. D. Biopsy histopathological examination
42. B. Acute pseudomembranous candidal stomatitis
43. C. Necrotizing gingivostomatitis
44. E. Nourishing Yin and Shengjisan or Ice Boron
45. D. Candidiasis
46. B. Viruses
47. E. Bacterial infections
48. A. Lips
49. B. Bacteria
50. A. Penicillin
51. The answer is D. corticosteroids.
52. The answer is B. trigeminal nerve branch 2.
53. The answer is D. clinical presentations are different.
54. The answer is B. encephalitis.
55. The answer is B. injection.
56. The answer is C. The diagnosis is incorrect.
57. The answer is E. digestive tract.
58. The answer is C. angular cheilitis
59. The answer is B. 161 shell particles.
60. The answer is E. digestive tract.
61. The answer is C. Bright red edema in the lesion area, or mottled pseudomembrane.
62. The answer is C. biopsy.
63. The answer is D. The oral mucosa is hyperemic, with curd-like patches.
64. The answer is D. The oral mucosa is hyperemic, with curd-like patches.

These answers are based on the understanding and analysis of knowledge in dentistry.

**Chapter 3 Allergic Diseases of the Oral Mucosa**

1.2009U2A1028 Erythema multiforme complicated by skin lesions is characterized by ( )

A. Erosion

B. Ulcers

C. Exudation is excessive, forming a grayish-white pseudofilm

D. Significant pain

E. Erythema iridis

2. 2009U2A1036 Erythema multiforme oral lesions, the correct clinical manifestations are ( )

A. The most common lesion is a large erosion with a large amount of fibrinous exudate forming a thick pseudomembrane on the surface

B. Thick, black crusts often form on the lips

C. Significant pain that interferes with eating

D. Submandibular lymph nodes are swollen and tender

E. All of the above

3. 2010U2A1010 may belong to allergic diseases ( )

A. Glyphic tongue

B. Erythema multiforme exudative erythema

C. Map tongue

D. Glandular cheilitis

E. Angular cheilitis

4.2010U2A1011 Which of the following may be the trigger for the onset of angioedema ( )

A. Bacterial infection

B. Food

C. Autoimmunity

D. Trauma

E. Viruses

5. 2010U2A1015 Toxic epidermal necrolysis clinical manifestations are correct ( )

A. Epidermal lysis, positive Nikolsl sign, lesions appear only on the skin

B. The oral mucosa is red and swollen, bullae appear, and the Nikols sign is negative

C. Blistering erythema on the skin, redness and swelling of the oral mucosa, bullae without epidermal release

D. Positive Nikolsz sign only on the oral mucosa

E. Skin epidermal lysis, positive Nikol's sign, redness and swelling of the oral mucosa, bullae, large erosions, and easy bleeding

6. 2010U2A1027 Which of the following descriptions is wrong about erythema multiforme exudative ( )

A. is an allergic disease

B. Oral mucosa exhibits extensive erosions

C. Skin lesions are erythematous, blisters, and papules

D. Ocular lesions are iridocyclitis and empyema

E. May be accompanied by obvious systemic reactions, such as high fever, headache, etc

7.2002U2A1015 Which of the following allergic diseases is related to the season ( )

A. Erythema multiforme

B. Drug-allergic stomatitis

C. Allergic contact stomatitis

D. Angioedema

E. Toxic epidermal necrolysis

8. 2013U2A2086 A 38-year-old female patient, Xiao, had unexplained ulceration of the lips and mouth for 2 weeks, severe pain, and difficulty in speaking and eating. The patient had a similar illness in the last year. Examination: erosion of the lips, easy bleeding, bloody crusts. There are large erosions on the dorsum of the tongue, the floor of the mouth, and the right cheek, covered with a yellow pseudomembrane. Both palms have round or oval erythema. The possible diagnosis for this patient is ( )

A. Pemphigus

B. Drug-induced stomatitis

C. Discoid lupus erythematosus

D. Erythema multiforme exudative erythema

E. erosive lichen planus

9.2004U2A1030 The following clinical features of drug allergic stomatitis are correctly described as ( )

A. Skin bullae, white scars of the oral mucosa

B. Purple papules on the skin with white streaks in the mouth

C. Erythema and blisters on the skin, blisters and erosions of different sizes in the oral cavity

D. Skin blisters or maculopapular rashes, small clear blisters that merge with each other in the mouth

E. Erythema nodosum of the skin, recurrent ulcers in the mouth

10.2004U2A1041 Angioneuroedema occurs in which part of the most dangerous part ( )

A. Lips

B. Tongue

C. Palate

D. Epiglottitis

E. Eyelids

11.2004U2A1042 Serious complications that may occur with angioedema are ( )

A. Asthma

B. Swollen eyelids

C. Esophageal stricture

D. Restricted tongue movement

E. Laryngeal edema, suffocation

12.2004U2A1043 Which of the following locations does not generally occur angioneurotic edema ( )

A. Tongue

B. Lips

C. Eyelids

D. Palate

E. Throat

13.2002U2A1023 Which of the following descriptions is consistent with the clinical features of erythema multiforme ( )

A. The onset is seasonal, and it is more common in autumn and winter

B. Generally not accompanied by systemic reactions

C. The onset of the disease is slow, often prolonged, and not self-limited

D. Lesions are characterized by erythema, edema, bullae, erosions, etc

E. Lesions may occur in the oral cavity, but not on the skin, eyes, or genitals

14.2009U2A1070 The antibody produced by the body during the drug allergic stomatitis is ( )

A. IgA

B. IgG

C. IgM

D. IgD

E. IgE

1. The answer is E. iris erythema. The characteristic clinical manifestation of erythema multiforme is erythema iridis.
2. The answer is C. The pain is significant and interferes with eating. This is typical of oral lesions in erythema multiforme.
3. The answer is B. erythema multiforme exudative. It is an allergic disease.
4. The answer is E. virus. Viral infections are a common cause of angioedema.
5. The answer is E. Skin epidermal lysis, positive Nikols sign, redness and swelling of the oral mucosa, bullae, large erosions, and easy bleeding. This describes the clinical manifestations of toxic epidermal necrolysis.
6. The answer is D. Ocular lesions are iridocyclitis and empyema. This is not characteristic of erythema multiforme exudative.
7. The answer is D. angioedema. It is not directly related to seasonal changes.
8. The answer is D. erythema multiforme exudative. The patient's symptoms are consistent with features of erythema multiforme exudative.
9. The answer is C. erythema, blisters on the skin, blisters of varying sizes in the mouth, erosions. This describes the clinical features of drug-allergic stomatitis.
10. The answer is D. epiglottis. Epiglottic edema can cause severe breathing difficulties and even choking.
11. The answer is E. laryngeal edema, suffocation. This is a serious complication that can occur with angioedema.
12. The answer is D. palate. Angioedema generally does not occur in the palate.
13. The answer is D. Lesions are characterized by erythema, edema, bullae, erosions, etc. This description is consistent with the clinical features of erythema multiforme.
14. The answer is E. IgE. Antibodies produced in the body during drug-allergic stomatitis are usually IgE.

**Chapter 4 Oral Mucosal Ulcer Diseases**

1.2004U2A1017 The following factors may cause traumatic ulcers of the oral mucosa, except ( )

A. Eat hot noodles

B. Mechanical friction

C. Orthodontic wire

D. Chew gum

E. Sharp cusp irritation

2.2009U2A1030 Which of the following is a non-self-injurious ulcer ( )

A. Pressure ulcers

B. Self-injurious ulcers

C. Chemical burn ulcers

D. Heat-injured ulcers

E. All of the above

3.2009U2A1037 Among the following diseases, the main occurrencies on the oral mucosa are ( )

A. Recurrent aphthous ulcers

B. lichen planus

C. Erythema multiforme

D. Behcet's disease

E. Lupus erythematosus

4.2000U2A1005 Among the treatment measures for recurrent oral ulcers, the best short-term efficacy is ( )

A. Local oral anti-inflammatory, analgesic and healing

B. Surgical excision

C. Injection of transfer factor or oral levamisole

D. Treatment of systemic and local factors associated with the onset of the disease

E. Nutritional supplementation

5.2004U2A1018 Intensive use of corticosteroids may cause the following adverse consequences, except ( )

A. Diabetes mellitus

B. Decreased blood pressure

C. Decreased serum calcium

D. Candida infection

E. Peptic ulcers

6.2004U2A1028 Which of the following is NOT an oral allergic disease ( )

A. Riga's disease

B. Contact stomatitis

C. Drug-induced stomatitis

D. Sjohn's syndrome

E. Angioneurotic edema

7. 2004U2A1034 Clinical features of recurrent oral ulcers do not include ( )

A. It is more common in teenagers

B. The course of the disease is 7~14 days, and it is self-limited

C. It is more common in non-keratinized mucous membranes such as lips, cheeks, and tongue

D. Recurrent ulcers are round or oval in shape with a yellow pseudomembrane on the surface and red around them

E. Lesions can affect the perioral skin

8. 2012U2A2101 Male, 8 months old, tongue-tie ulceration and mass for 1 month, affecting eating and tongue activity, sought treatment. Examination: the deciduous incisors erupted, the edges were sharp, and ulcers of 1.0 cm × 0.8 cm in size could be seen at the tongue frenulum, the ulcers proliferated, the edges were raised and turned out, and there was a gray-white pseudomembrane on the surface, which was tough to the touch. This child may be diagnosed with ( )

A. Tumors

B. Riga's disease

C. Bednar aphthous ulcers

D. Periglandular aphthous ulcers

E. Pressure ulcers

9.2013U2A2076 Male, 33 years old. There are many ulcers in the mouth, the pain can not eat, the mucous membrane of the cheeks and the back of the tongue, more than ten ulcers of millet grains can be seen, scattered and distributed, and the surrounding mucosa is widely congested and swollen. History of multiple previous episodes of mild aphthous ulcers. Should be diagnosed as ( )

A. Herpetic gingivostomatitis

B. Herpangina

C. Herpetiform aphthous ulcers

D. Herpes zoster

E. Behcet's disease

10.2013U2A2078 Male, 8 months old, tongue-tie ulceration in January, mandibular breast middle incisors erupted, 1.0 cm × 0.8 cm ulcers were visible on both sides of tongue frenulum with ulcer hyperplasia, elevated margins and eversion valgus, and gray-white pseudomembranes. Palpation is firm. This child may be diagnosed with ( )

A. Lee-Freund's disease

B. Mouth sores in Pei's mouth

C. Periglandular aphthous ulcers

D. Decubitus ulcers

E. Thrush

11. 2002U2A1016 The clinical manifestations of mild aphthous ulcer are wrong ( )

A. The ulcer is depressed in the middle and the base is not hard

B. There is a redness band of hyperemia about 1 mm around the perimeter

C. The surface is covered with a light yellow pseudofilm

D. Burning sensation is obvious

E. Healing is not until treatment

12.2013U2A2087 Female, 35 years old. He complained of pain and ulceration on the left side of the lower lip for 4 days, affecting eating. The same history of 3~6 attacks per year. Oral examination: a 4mm diameter ulcer can be seen on the mucosa on the left side of the lower lip, the periphery is red, there is a yellow pseudomembrane on the surface of the ulcer, and the bottom of the ulcer is slightly depressed, and the tenderness is obvious. Clinically diagnosable as ( )

A. Recurrent mild aphthous ulcers

B. Recurrent herpetiform stomatitis

C. Recurrent severe aphthous ulcers

D. Erythema multiforme

E. Herpetic stomatitis

13.2013U2A2088 A 30-year-old female complained of moderate pain in the lower lip for 2 days, which was aggravated by eating irritating food; Oral examination: a 5 mm diameter ulcer was found on the mucosa of the lower left 1 labial side, covered with pseudomembrane, and the ulcer base was congested and tender. The chief complainant has a history of similar diseases for about 3 years, and has 4~6 attacks per year. The ulcer heals on its own. Probable( )

A. Discoid lupus erythematosus

B. Herpetiform stomatitis

C. Mild aphthous ulcers

D. Severe aphthous ulcers

E. Erythema multiforme

14.2013U2A2089 female, 46 years old, mouth ulcers recurrent for 3 years, with an interval of 1~2 months, mostly before menstruation, ranging from 1~3 each time, mainly located in the lower lip and tongue and other parts, the pain is obvious, and it heals in about 7~10 days. In order to achieve the desired therapeutic effect, it is advisable to take ( )

A. Local symptomatic treatment of the oral cavity

B. Systemic antibiotics

C. Multivitamin supplementation

D. Oral estrogen + oral local symptomatic therapy

E. Injection of transfer factors

15.2013U2A1029 The following description of the clinical manifestations of severe aphthous ulcer is incorrect ( )

A. The edge of the ulcer is neat, crater-like

B. The edge of the ulcer is uneven, rat-like, and has granulation tissue at the bottom

C. The ulcer is regular-shaped, red, swollen and raised

D. The diameter of the ulcer can reach 10~30mm

E. Ulcers deep into the submucosa up to the muscular layer

16. 2013U2A2091 patient, male, 50 years old. Oral and palate ulceration pain for more than 1 month. Has a history of recurrent seizures. Oral examination: 1 large and deep ulcer on the soft palate, 1.5cm in diameter, like a crater, red, swollen and bulging around but neat, slightly concave at the bottom, with a pseudomembrane on the surface, and a defect in the palatal lobe. Initial diagnosis is ( )

A. Cancerous ulcers

B. Tuberculous ulcers

C. Traumatic ulcers

D. Recurrent severe aphthous ulcers

E. Necrotizing salivary gland metaplasia

17.2013U2A2092 Jiang, female, 36 years old, with multiple oral ulcers for 1 week, pain and inability to eat. Examination: More than 10 ulcers of millet grains were found on the mucosa of both cheeks and tongue, scattered and distributed, and the surrounding mucosa was congested and red. The patient has a history of multiple similar seizures in the past. The patient should be diagnosed with ( )

A. Behcet's disease

B. Herpes zoster

C. Herpetiform aphthous ulcers

D. Herpangina

E. Herpetic gingivostomatitis

18.2013U2A2093 Male 40 years old, found that the palate was swollen and ulcerated for more than 2 months. Investigation: A deep ulcer was seen at the junction of the soft and hard palate, which could reach the bone surface, with a diameter of 1.3cm, irregular shape, clear boundaries, and a raised ulcer edge. Biopsy revealed tissue necrosis and squamous metaplasia of the salivary ducts and acinar epithelium. Probable( )

A. Palatal squamous cell carcinoma

B. Oral tuberculosis

C. Periglandular aphthous ulcers

D. Necrotizing salivary gland metaplasia

E. Palatal adenocarcinoma

19.2014U2A2086 A 30-year-old male patient, Duan, visited the hospital for two years due to repeated ulceration of the oral mucosa, and was examined: there were 2 small ulcers on the right lingual margin, the ulcers were slightly concave, showing yellow pseudomembrane, and the tenderness was obvious. Ocular conjunctivitis, facial boils, and ulcers of the external genitalia should be considered for diagnosis ( )

A. Gonorrhea

B. Behcet's disease

C. Herpes zoster

D. Herpetic stomatitis

E. Recurrent mouth ulcers

20.2014U2A2087 Male, 69 years old. Pain at the base of the left tongue for 3 days, affecting eating. Oral examination: only the lower left 6 remained, the crown remained. The missing tooth has been restored with a denture. An ulcer with a diameter of 1 cm was seen at the base of the left tongue, which was tender to the touch. The appropriate treatment is ( )

A. Ulcer excision

B. Remove the lower left 6

C. Chemotherapy

D. Sharpen off the lower left 6 sharp edges

E. Prednisone plus lidocaine ulcer basal infiltration injection

21.2014U2A2088 Male, 69 years old, only the lower left 7 remains, and the rest are removable dentures. complained of accidentally biting a hard object two weeks ago, a small piece of the crown of the tooth collapsed, because the pain was not obvious and was not paid attention to, recently there was pain at the root of the left tongue, and the oral examination showed that there was an ulcer at the root of the left tongue, and the more appropriate treatment was ( )

A. Remove the lower left 7

B. Excision of the ulcer on the left edge of the tongue

C. Radiotherapy

D. Adjust the sharp edge of the lower left 7 sharp, followed by observation and follow-up

E. Prednisone plus lidocaine infiltrated into the base of the ulcer 10 times

22.2014U2A2089 Hu, male, 66 years old, nasopharyngeal carcinoma was subjected to radiotherapy after surgery, and the oral mucosa ulcerated in a large area after two radiotherapy treatments, and the pain was obvious. Seek treatment. The possible diagnosis of this patient is ( )

A. Allergic stomatitis

B. Contact stomatitis

C. Radiation stomatitis

D. Candida albicans infection

E. Acute infectious stomatitis

23.2014U2A2090 male, 60 years old, nasopharyngeal carcinoma after postoperative radiotherapy, after two radiotherapy treatments, the oral mucosa ulcerated in a large area, and the pain was obvious. The possible diagnosis of this patient is ( )

A. Candida albicans infection

B. Radiation stomatitis

C. Acute infectious stomatitis

D. Allergic stomatitis

E. Contact stomatitis

24.2003U2A1002 Clinical manifestations of herpetiform ulcer ( )

A. Blisters and neuralgia in unilateral banded clusters

B. Sudden onset of acute inflammation with a history of medication prior to onset

C. History of trauma, ulcer morphology often coincident with mechanical stimuli

D. In the early stage, the mucosal of the affected area is congested, scattered with small spots as white as snow, which can soon merge into white patches and continue to expand and spread

E. Ulcers are small and numerous, scattered in any part of the mucosa, less than 2 mm in diameter, and similar ulcers can coalesce into patches

25.2002U2A1024 The following ulcers have recurrence ( )

Aphthous ulcers of severe aphthous ulcers

B. Cancerous ulcers

C. Tuberculous ulcers

D. Traumatic ulcers

E. Necrotizing salivary gland metaplasia

26.2002U2A1025 Adrenocorticosteroids can be injected locally in ( )

A. Tuberculous ulcers

B. Snow mouth disease

C. Periglandular aphthous ulcers

D. Herpetic stomatitis

E. None of the above

27.1999U2A1005 Which of the treatment measures for recurrent oral ulcers has the best effect ( )

A. Local oral anti-inflammatory, analgesic and healing-promoting oral cavity

B. Surgical excision

C. Injection of transfer factor or oral levamisole

D. Whenever possible, identify and treat systemic and local factors associated with the onset of the disease

E. Nutritional supplementation

28.2015U2A1018 The most commonly used method for the treatment of severe recurrent aphthous ulcers that do not heal for a long time is ( )

A. Oral antibiotics

B. Localized cautery of 10% silver nitrate solution

C. Triamcinolone acetonide suspension for local injection

D. Surgical excision of the lesion

E. Fluconazole paste is applied topically

29.2015U2A2133 Ulceration of the mucosa at the junction of the hard and soft palate of the infant due to artificial feeding with a hard rubber nipple, called ( )

A. Bednar ulcer

B. Riga-Fede ulcer

C. Periglandular aphthous ulcers

D. Traumatic ulcers

E. Tuberculous ulcers

30.2015U2A2134 Ulcers formed by the corresponding mucosa caused by the stimulation of the sharp edges of the stump and the residual crown are called ( )

A. Bednar ulcer

B. Riga-Fede ulcer

C. Periglandular aphthous ulcers

D. Traumatic ulcers

E. Tuberculous ulcers

31.2019U2A1004 Infants sucking their thumbs or using too hard rubber nipples cause ulcers on the mucosal surface of the bilateral wing hooks, which is called ( )

A. Traumatic ulcers

B. Pressure ulcers

C. Bednar ulcer

D. Riga-Fede ulcer

E. Recurrent aphthous ulcers

32.2017U2A2079 In the systemic treatment of recurrent aphthous ulcers, the correct method of using adrenocorticosteroids is ( )

A. Prednisone 10~30mg/d, divided into 3 oral doses, and gradually reduce the dose after ulcer control

B. Prednisone 60mg/d, divided into 3 oral doses, reduce the dose after controlling the disease

C. Prednisone 60~80mg/d, divided into 3 oral doses, reduce the dose after controlling the disease

D. Prednisone 80~100mg/d, divided into 3 oral doses, reduce the dose after controlling the disease

E. Prednisone 100~120mg/d, divided into 3 oral doses, reduce the dose after controlling the disease

33.2017U2A1056 The description of the characteristics of recurrent oral ulcers is correct ( )

A. Specific, recurrent, and self-limited

B. Contagious, cyclical and self-limiting

C. Specific, infectious, and recurrent

D. Periodic, recurrent, and self-limited

E. Clustered, specific, and cyclical

The answer is E. sharp cusp irritation. This usually does not cause a traumatic ulcer of the oral mucosa.

The answer is A. decubitus ulcers. This is an example of a non-self-injurious ulcer.

The answer is A. recurrent aphthous ulcers. This is a disease that occurs mainly on the mucous membranes of the mouth.

The answer is C. injection of transfer factor or oral levamisole. These treatments are more effective in the near term for recurrent mouth ulcers.

The answer is B. Decreased blood pressure. Heavy use of corticosteroids usually causes blood pressure to rise, not decrease.

The answer is D. Sjohn's syndrome. This is not an oral allergic disease.

The answer is E. lesions can affect the perioral skin. Recurrent mouth sores usually don't affect the skin around the mouth.

The answer is C. Bednar aphthous ulcers. This is a specific type of ulcer caused by mechanical irritation in infants.

The answer is A. herpetic gingivostomatitis. Considering the patient's history of multiple minor aphthous flares, herpetic gingivostomatitis may be the correct diagnosis.

The answer is B. burner's aphthous ulcer. This is because of ulcers caused when baby teeth erupt.

The answer is E. healing after treatment. Mild aphthous ulcers usually heal on their own and don't have to be treated.

The answer is A. recurrent minor aphthous ulcers. History and clinical presentation consistent with this type of ulcer.

The answer is C. mild aphthous ulcers. Mild sores that are frequent usually heal on their own.

The answer is: D. oral estrogen + local symptomatic treatment of the oral cavity. This treatment may be more effective for recurrent canker sores.

The answer is B. ulcers with uneven edges, rat-like appearance, and granulation tissue at the bottom. This is characteristic of severe aphthous ulcers, not mild ones.

The answer is D. recurrent severe aphthous ulcers. Large, deep ulcers with a history of recurrent recurrent sores are consistent with this type of ulcer.

The answer is C. herpetiformis. The patient's symptoms and history of multiple episodes are consistent with the diagnosis of herpetiform aphthous ulcers.

The answer is D. necrotizing salivary gland metaplasia. Tissue necrosis and squamous metaplasia of the salivary ducts and acinar epithelium are consistent with this diagnosis.

The answer is B. Behcet's disease. A combination of oral mucosal ulcers, ocular conjunctivitis, and external genital ulcers suggests the diagnosis.

The answer is D. Sharpen off the lower left 6 sharp edges. This is the appropriate treatment to address the cause of the ulcer and reduce irritation by smoothing out the sharp edges.

· D. Adjust the sharp edge of the lower left 7 sharp, followed by observation and follow-up

· C. Radiation stomatitis

· B. Radiation stomatitis

· A. Blisters and neuralgia in unilateral banded clusters

· Aphthous ulcers of severe aphthous ulcers

· E. None of the above

· D. Whenever possible, identify and treat systemic and local factors associated with the onset of the disease

· C. Triamcinolone acetonide suspension for local injection

· B. Riga-Fede ulcer

· D. Traumatic ulcers

· D. Riga-Fede ulcer

· A. Prednisone 10~30mg/d, divided into 3 oral doses, and gradually reduce the dose after ulcer control

· D. Periodic, recurrent, and self-limited

**Chapter 5 Oral Mucosal Bullous Diseases**

1. 2004U2A1014 Pemphigoid can have the following clinical manifestations, except ( )

A. Skin blisters

B. Microstoma

C. Positive Nikol's sign

D. Exfoliative gingivitis

E. Bulbar conjunctival adhesions

2. 2004U2A1015 The most common site of lesions in the oral cavity of cicatricial pemphigoid is ( )

A. Gums

B. Lower lip

C. Soft palate

D. Palate

E. Buccal mucosa

3.2009U2A1021 Among the following diseases, spontaneous remission is ( )

A. Pemphigus vulgaris

B. Cicatricial pemphigoid

C. Erosive lichen planus

D. Bullous pemphigoid

E. Epidermolysis bullosa

4.2009U2A1023 Blisters can be quickly formed by gently pushing the skin or mucous membranes with a finger on the normal appearance, which is called ( )

A. Positive peeling test

B. Epidermolysis

C. Positive Nikol's sign

D. Positive OT test

E. Positive penicillin test

5.2009U2A1026 Among the following bullous diseases, which can be life-threatening, are ( )

A. Cicatricial pemphigoid

B. Pemphigus vulgaris

C. Bullous pemphigoid

D. Pemphigoid multiforme

E. Bullous lichen planus

6. 2009U2A1031 Among the various types of pemphigus, the least damage to the oral mucosa is ( )

A. Pemphigus vulgaris

B. Proliferative pemphigus

C. Pemphigus foliaceus

D. Pemphigus erythematosus

E. The probability of occurrence of the above types is approximately the same

7. 2009U2A1034 Hormone therapy for pemphigus is characterized by ( )

A. The condition generally does not recur after stopping the drug

B. Emphasis on large volume, speed, and rapid control of inflammation

C. Emphasize gradual, impatient, and slowly increase from low amounts

D. Can be replaced by traditional Chinese medicine to reduce the adverse effects of hormones

E. It can be dynamically divided into four stages: initiation, control, consolidation and maintenance

8.2009U2A1035 Among the following diseases, the loss of connections between epithelial cells is an autoimmune disease.

A. Behcet's disease

B. Pemphigus

C. Pemphigoid

D. Lupus erythematosus

E. erythema multisexual

9. 2012U2A1004 The most accurate description of the characteristics of hormone therapy for pemphigus is ( )

A. Quantity is large and fast

B. Increment from low amount

C. Initiation, control, consolidation, and maintenance

D. Can be replaced by traditional Chinese medicine

E. No recurrence of the condition after discontinuation of the drug

10. 2004U2A1029 Benign mucous membrane pemphigoid also known as ( )

A. Bullous pemphigoid

B. Erythematous pemphigoid

C. Proliferative pemphigoid

D. Scarring pemphigoid

E. Deciduous pemphigoid

11.2004U2A1033 The following diseases are autoimmune diseases ( )

A. Erythema multiforme, pemphigus, lupus erythematosus

B. Pemphigus, pemphigoid, lupus erythematosus

C. Pemphigoid, erythema multiforme, lupus erythematosus

D. Behcet's disease, lupus erythematosus, angioedema

E. Lupus erythematosus, angioedema, erythema multiforme

12. 2010U2A1013 What type of pemphigus usually presents with oral lesions ( )

A. Proliferative pemphigus

B. Pemphigus vulgaris

C. Pemphigus foliaceus

D. Bullous pemphigoid

E. Pemphigus erythematoform

13.2010U2A1031 Which of the following lesions will form a scar after healing ( )

A. Pemphigus vulgaris

B. Bullous pemphigoid

C. Cicatricial pemphigoid

D. Pemphigoid multiforme

E. B+C

14.2002U2A1018 Cicatricial pemphigoid can occur anywhere in the oral cavity, and the highest incidence is ( )

A. Gums

B. Soft palate

C. Hard palate

D. Tongue

E. Buccal mucosa

15.2014U2A2091 Male, 68 years old, oral ulcer 3 months after visiting the clinic, physical examination found that the buccal mucosa was a large area of erosion, with marginal expansion, and the surface of the erosion surface was covered with a thick gray-white pseudofilm, which was patchy. The diagnosis of pemphigus was probable by exfoliative cytology. The blood test routine and classification of white blood cells were 1.6×10⁹/L, and neutrophils accounted for 82%. The most realistic treatment of choice for this case is ( )

A. 1%~2% procaine solution gargle to relieve pain

B. Topical application of corticosteroid ointment

C. Systemic antibiotics and oral chlortetracycline 0.25% gargle

D. Nystatin in the mouth, alkaline mouthwash topical in the mouth

E. Maintain oral hygiene and remove irritants

16.2014U2A1009 Exfoliative gingival lesions may be a sign of the following diseases, but do not include ( )

A. Pemphigus

B. Pemphigoid

C. Vitiligo

D. Lichen planus

E. Lupus erythematosus

17.2014U2A1010 The following diseases do not usually manifest as lip erosions are ( )

A. Chronic cheilitis

B. Discoid lupus erythematosus

C. Erythema multiforme

D. Proliferative pemphigus

E. Bullous pemphigoid

18.2005U2A1013 Pemphigus belongs to ( )

A. Allergic diseases

B. Autoimmune diseases

C. Immunodeficiency disorders

D. Infectious diseases

E. Communicable diseases

19.2004U2A1044 Which of the following statements related to pemphigoid is false ( )

A. Have autocirculating antibodies

B. Anti-basement membrane antibodies

C. Anti-cellular-cell antibodies

D. Direct immunofluorescence can be used

E. Anti-basement membrane zone antibodies are mainly IgG

20.2002U2A1026 Which of the following is NOT a clinical manifestation of cicatricial pemphigoid ( )

A. Skin blisters

B. Microstoma

C. Exfoliative gingivitis

D. Positive Nikolsz sign

E. Bulb conjugated membrane adhesions

1. **E. Bulbar conjugatory membrane adhesion pemphigoid mainly affects the skin and mucous membranes and atypically affects the bulbar conjugated membrane.**
2. **E. Buccal mucosal cicatricial pemphigoid is commonly found in the buccal mucosa in the oral cavity.**
3. **C. Erosive lichen planus erosive lichen planus may resolve spontaneously, but usually requires treatment.**
4. **C. Positive Nikols sign is a positive Nikols sign that can lead to blister formation by gently scraping the skin or mucous membranes.**
5. **C. Bullous pemphigoidBullous pemphigoid is a potentially life-threatening condition because it can lead to extensive skin shedding and other serious complications.**
6. **Each of the above types has approximately the same probability of occurrence, and each type of pemphigus may cause damage to the oral mucosa with no obvious minimum frequency.**
7. **B. Hormone therapy that emphasizes high-volume, speedy, and rapid control of inflammation in pemphigus usually requires rapid, high-dose control of inflammation.**
8. **B. Pemphigus Pemphigus is an autoimmune disease in which there is a loss of connectivity between epithelial cells.**
9. **A. Hormone therapy for high-volume, pemphigus requires rapid and high doses to control the disease.**
10. **E. Deciduous pemphigoidBenign mucous membrane pemphigoid, also known as deciduous pemphigoid.**
11. **B. Pemphigus, pemphigoid, lupus erythematosus are all autoimmune diseases.**
12. **B. Pemphigus vulgarisPemphigus vulgaris usually affects the oral mucosa.**
13. **C. Cicatricial pemphigoid: Cicatricial pemphigoid scarring occurs after healing.**
14. **E. Buccal mucosa cicatricial pemphigoid occurs most frequently in the oral cavity in the buccal mucosa.**
15. **B. Topical corticosteroid ointmentFor pemphigus, topical corticosteroids are a common treatment.**
16. **C. Leukoplakia exfoliative gingival lesions are commonly associated with pemphigus, pemphigoid, lichen planus, and lupus erythematosus, but not leukoplakia.**
17. **D. Proliferative pemphigusProliferative pemphigus does not usually manifest as lip erosions.**
18. **B. Autoimmune diseasePemphigus is an autoimmune disease.**
19. **B. Pemphigoid with anti-basement membrane antibodies is often associated with anti-cell-to-cell antibodies, rather than anti-basement membrane antibodies.**
20. **D. A positive Nikols sign is not a typical clinical manifestation of cicatricial pemphigoid.**

**Chapter 6 Oral Markings Diseases**

1.2004U2A1016 Discoid lupus erythematosus is the most common site in the oral mucosa ( )

A. Cheeks

B. Soft palate

C. Back of the tongue

D. Upper lip redness

E. Lower lip redness

2.2009U2A1016 Diseases that do not undergo malignant transformation are ( )

A. Vitiligo

B. Chronic discoid lupus erythematosus

C. lichen planus

D. Oral erythema

E. Recurrent aphthous ulcers

3.2009U2A1029 The most effective treatment for oral leukatosis is ( )

A. Surgical excision

B. Oral vitamin A acid

C. Topical application of vitamin A acid preparation

D. Removal of local irritants

Local injection of 2.5% prednisone suspension

4.2000U2A1002 Oral mucosal leukoplakia may have the following clinical manifestations, except for ( )

A. White or off-white homogeneous plaques or crumpled paper-like plaques

B. White irregular bulges with papillary protrusions

C. Granular red-white interlesions

D. Ulceration formation on the basis of white plaques

E. White curd-like pseudomembrane

5. 2012U2A1003 Oral erythema also has the following names, in addition to ( )

A. Red spots

B. Red proliferative lesions

C. Erythema multiforme

D. Erythema proliferative

E. Quillette erythema

6.2004U2A1031 Which of the following statements is true about the pathogenic factors of vitiligo ( )

A. Quitting smoking can lead to vitiligo

B. Chewing betel nut can cause white patches

C. Plaque irritation can lead to white patches

D. Vitiligo has a familial predisposition to hereditary properties

E. Immunocompromised can cause vitiligo

7. 2004U2A1035 Adverse reactions of quinoline chloride include the following, except ( )

A. Visual impairment

B. Decreased blood pressure

C. Nausea, vomiting

D. Liver impairment

E. Decreased white blood cell count

8. 2010U2A1014 Granular leukoplakia is more common in ( )

A. Mucosa in the corner of the mouth

B. Soft palatal pharynx

C. Dorsopharyngeal part of the tongue

D. Floor of the mouth and tongue

E. Gums

9. 2010U2A1018 is an autosomal dominant disease ( )

A. Vitiligo

B. Gingivitis

C. White wrinkle

D. Adolescent periodontitis

E. Color blindness

10.2010U2A1020 Epidemiological statistics, females are more likely to occur than males ( )

A. Vitiligo

B. Discoid lupus erythematosus

C. C. Cies

D. Map tongue

E. Gum cancer

11.2010U2A1021 The following are not the most common sites of oral leukratosis( )

A. Hard palate

B. Lip redness

C. Floor of the mouth

D. Dorsum of the tongue

E. Buccal mucosa

12.2010U2A1022 Skin lichen planus lesions are mainly manifested as ( )

A. Reticulated type

B. Flat papules

C. Annular type

D. Plaque type

E. Streaked type

13.2010U2A1025 Which of the following chronic discoid lupus erythematosus laboratory test results is incorrect ( )

A. Reduction of OKT4/OKT8 ratio

B. Accelerated erythrocyte sedimentation rate

C. Elevated globulin

D. Rheumatoid factor positive

E. Positive for antinuclear antibodies

14.2011U2A1006 Histopathological manifestations of OLP ( )

A. Hypogoniosis or dysplasia

B. Epithelial hyperkeratosis

C. Hypoangulation

D. Liquefaction and degeneration of basal cells

E. Dense bands of lymphocyte infiltration in the connective tissue of the lamina propria

15.2013U2A2077 Male, 22 years old, white lesions on the tongue for 5 years, aggravated for half a year. Examination: On the left and right sides of the dorsal part of the tongue, two white plaques of about 1.1~1.3 cm can be seen respectively, with irregular edges, and striped white lines on the mucosa on the inner side of the lower lip. The clinical diagnosis should be ( )

A. Lichen planus

B. Candidiasis albicans

C. Herpes simplex

D. Cancerous ulcers

E. Coccal stomatitis

16.2010U2A1033 The best parts of oral lichen planus are ( )

A. Gums

B. Soft palate

C. Hard palate

D. Tongue

E. Buccal mucosa

17. 2002U2A1019 Diseases that may be related to light exposure are ( )

A. Erythema multiforme

B. Angioedema

C. Pemphigoid

D. Discoid lupus erythematosus

E. Angular cheilitis

18.2004U2A1040 Which cell-mediated inflammatory disease of oral lichen planus ( )

A. NK cells

B. B lymphocytes

C. T lymphocytes

D. Neutrophils

E. Macrophages

19.2014U2A1011 Oral manifestations of lichen planus excl. ( )

A. Erosion

B. Blisters

C. Herpes

D. Plaque

E. Chapped

20.2014U2A2092 Male, 55 years old. A white patch appeared on the red part of the right lip for 1 year. No symptoms. The patient has a habit of smoking and drinking. Oral examination: a white patch can be seen on the red part of the right lip, which is homogeneous and cannot be wiped off, and no spores or hyphae were found in 3 microscopic examinations of the smear. Improper treatment is ( )

A. Stop smoking

B. Oral vitamin A

C. Topical application of 0.2% tretinoin

D. Silver nitrate topical treatment of white plaques

E. Regular follow-up, if there is an indication of malignant transformation, it should be excised immediately

21.2014U2A2093 Male, 58 years old, a white plaque appeared on the mucosa of the right cheek, homogeneous, could not be wiped off, no obvious pain on palpation, no spores or hyphae were found in the microscopic examination of the smear three times, the lower right 3456 was a poor restoration, no history of smoking and alcoholism, and the treatment measures were inappropriate ( )

A. Removal of defective restorations

B. Oral vitamin A

C. Topical application of 0.2% tretinoin

D. Silver nitrate is treated topically with white plaques to promote plaque regression

E. Regular follow-up, early surgical resection may be considered if there are indications of malignant transformation

22.2008U2A1001 The following measures can be used for vitiligo treatment, except for ( )

A. Quit smoking

B. Surgical excision

C. Cod liver oil

D. Silver nitrate cautery

E. Oral vitamin A

23.2005U2A1014 Among the precancerous lesions, the highest chance of carcinogenesis is ( )

A. Lichen planus

B. Vitiligo

C. Erythema

D. Warts

E. Chronic ulcers

24.2005U2A1015 Which of the following statements is false about the clinical classification of vitiligo ( )

A. Divided into homogeneous and non-homogeneous types

B. The heterogeneous type is further divided into verrucous type, granular type, and ulcer type

C. The homogeneous type manifests as homogeneous plaques or wrinkles on the surface

D. The verrucous type is heterogeneous

E. Heterogeneous types include verrucous, granular, and atrophic

25.2004U2A1045 There are ( ) that are not related to the pathogenesis of OLP

A. Neuropsychiatric factors

B. Viral infection

C. Excessive smoking and drinking

D. Immunological factors

E. Certain chemical drugs, possible genetic factors

26.2004U2A1046 Which of the following is a stimulus for oral leukatosis ( )

A. Smoking

B. Alcoholism

C. Likes spicy food

D. Prefer to eat too hot food

E. Prefer crunchy, hard foods

27.2004U2A1047 Chronic discoid lupus erythematosus skin lesions are characterized by ( )

A. Erythema

B. Hair follicle keratin plugs, scales, keratin plugs

C. Hyperpigmentation and/or hypopigmentation

D. Telangiectasia, atrophy, and scarring

E. All of the above

28.2003U2A1003 Treatment of discoid lupus erythematosus can be used ( )

A. Ribavirin

B. Chloroquine

C. Tretinoin

D. Fluconazole

E. Chlorpheniramine

29.2002U2A1027 Epidemiological statistics show that women are more likely to occur than men ( )

A. Chronic discoid lupus erythematosus

B. Chronic cheilitis

C. Gum cancer

D. Glyph tongue

E. Caries disease

30.1999U2A1006 Which of the following is NOT a pathological manifestation of lichen planus ( )

A. Incomplete keratinization of the epithelium

B. Liquefaction and denaturation of basal cells

C. Banded infiltrates of lymphocytes in the lamina propria

D. Presence of colloidal bodies

E. Collagen fibrillary degeneration

31.1999U2A1007 The following items can be used to distinguish white edema from vitiligo, except for ( )

A. White edema with soft texture

B. White edema with unclear borders

C. White edema with a smooth surface

D. White edema without epithelial keratinization

E. White edema, swelling of acanthocytes

32.2015U2A1019 The auxiliary examination to confirm the carcinogenesis of oral leukoplakia is ( )

A. Potassium hydroxide smear

B. PAS staining

C. Histopathological examination

D. Exfoliated cell examination

E. Toluidine blue staining

33.2018U2A1012 The oral mucosal diseases most closely related to smoking and drinking are ( )

A. Oral lichen planus

B. Mucous membrane pemphigoid

C. Submucosal fibrosis

D. Oral leukoplakia

E. White spongy nevus

34.2017U2A1059 Diagnosis of vitiligo can be based on ( )

A. Can be made based on clinical examination

B. Available from blood tests

C. Can be confirmed based on family history

D. Must be confirmed by pathological examination

E. Must be made by indirect immunofluorescence

35.2017U2A1058 Which of the following measures should not be used for the treatment of vitiligo ( )

A. Oral vitamin A

B. Silver nitrate cautery

C. Quit smoking

D. Surgical excision

E. Topical cod liver oil

36.2017U2A1057 The pathogenesis of oral lichen planus may be related to the following factors: ( )

A. Smoking, trauma, psychological factors

B. Genetics, smoking, alcoholism, bacterial infections

C. Psychological factors, smoking, poor restoration

D. Endocrine factors, viral infections, autoimmunity

E. Psychological factors, poor prosthetics, endocrine factors

37.2017U2A2080 In the epithelial lamina propria of oral lichen planus (OLP), a large number of lymphocytes are infiltrated in a dense band, which of the following factors is related to ( )

A. Psychological factors

B. Endocrine factors

C. Microcirculatory disorder factors

D. Immune factors

E. Heredity

38.2020U1A1001 The pathological characteristics of oral lichen planus are ( )

A. Excessive positive angulation of the surface

B. Clear basement membrane

C. Basal cell keratinization

D. Lamina propria lymphocyte cell infiltration zone

E. Abnormal hyperplasia of the spinous layer

· A. Cheeks

· E. Recurrent aphthous ulcers

· A. Surgical excision

· E. White curd-like pseudomembrane

· E. Quillette erythema

· B. Chewing betel nut can cause white patches

· B. Decreased blood pressure

· D. Floor of the mouth and tongue

· A. Vitiligo

· B. Discoid lupus erythematosus

· A. Hard palate

· B. Flat papules

· E. Positive for antinuclear antibodies

· B. Epithelial hyperkeratosis

· A. Lichen planus

· E. Buccal mucosa

· D. Discoid lupus erythematosus

· C. T lymphocytes

· A. Erosion

· D. Silver nitrate topical treatment of white plaques

· D. Silver nitrate is treated topically with white plaques to promote plaque regression

· D. Silver nitrate cautery

· B. Vitiligo

· E. Heterogeneous types include verrucous, granular, and atrophic

· B. Viral infection

· A. Smoking

· E. All of the above

· B. Chloroquine

· A. Chronic discoid lupus erythematosus

· E. Collagen fibrillary degeneration

· D. White edema without epithelial keratinization

· C. Histopathological examination

· D. Oral leukoplakia

· D. Must be confirmed by pathological examination

· B. Silver nitrate cautery

· C. Psychological factors, smoking, poor restoration

· D. Immune factors

· D. Lamina propria lymphocyte cell infiltration zone

**Chapter 8 Diseases of the Lips and Tongue**

1. The clinical manifestations of 2009U2A1019 May-Roche syndrome are ( )

A. Cracked tongue, angular cheilitis, glandular cheilitis

B. Cracked tongue, facial paralysis, granulomatous cheilitis

C. Geographic tongue, angular cheilitis, anemia

D. Geographic tongue, facial paralysis, anemia

E. Tongue papillitis, facial paralysis, granulomatous cheilitis

2.2009U2A1025 Which of the following is NOT a clinical feature of the geographic tongue ( )

A. It is more common in adults

B. It is more common on the dorsum of the tongue, the tip of the tongue and the margin of the tongue

C. Filiform papillae atrophy of the tongue

D. Diurnal "wandering" conjugation features

E. Generally no pain or other adverse sensations

3. 2004U2A1021 Which of the following is NOT a clinical manifestation of the geographic tongue ( )

A. The red zone can be eroded

B. The exfoliation zone is slightly depressed

C. Filiform papillae flakes

D. Fungal papillae are clearly visible

E. The dorsum of the tongue forms a red smooth area

4. The clinical manifestations of 2004U2A1025 Mero syndrome are ( )

A. Glyphical tongue, facial paralysis, angular cheilitis

B. Glyphical tongue, facial paralysis, glandular cheilitis

C. Glyphical tongue, facial paralysis, granulomatous cheilitis

D. Geographic tongue, facial paralysis, angular cheilitis

E. Geographic tongue, angular cheilitis, granulomatous cheilitis

5.2004U2A1032 The following factors can cause angular cheilitis, except ( )

A. Malnutrition

B. Cold stimulation

C. Bacterial infection

D. Fungal infection

E. Traumatic factors

6.2002U2A1020 Factors unrelated to angular erosion ( )

A. Deficiency of B vitamins, leading to malnutrition

B. Bacterial infection

C. Congenital inheritance

D. Fungal infection

E. Older edentulous patients with shorter spacing

7.2014U2A2094 male, 38 years old. General malaise, loss of appetite for 2 days, accompanied by blistering and crusting of the red mucosa and surrounding skin on the left side of the upper lip. Which of the following disorders is least likely to be considered for diagnosis ( )

A. Herpetic stomatitis

B. Cold sores

C. Herpes zoster

D. Hand, foot and mouth disease

E. Chronic cheilitis

8. 2010U3A1003 The following are not common causes of angular cheilitis ( )

A. Vitamin B₂ deficiency

B. Candida albicans infection

C. Excessive sun exposure

D. Exposure to allergens

E. Stretching time is too long during oral treatment

9.2004U2A1049 Glyph tongue with giant lips and facial paralysis ( )

A. May-Roe syndrome

B. Lytle syndrome

C. Behcet's syndrome

D. Burning mouth syndrome

E. Sjögren's syndrome

10.1999U2A1008 The cause of chronic cheilitis is ( )

A. Dry climate

B. Bad habit of licking and biting lips

C. Habits of smoking, drinking, and hot food

D. Chemical and mechanical stimuli

E. Unknown etiology

11.2002U2A1029 Which B vitamin deficiency is prone to cause dystrophic cheilitis ( )

A. Vitamin B₁

B. Vitamin B₂

C. Vitamin B₁₂

D. Vitamin B₆

E. Niacin

12.1999U2A1008 The cause of chronic cheilitis is ( )

A. Bad habit of licking lips

B. Trauma

C. Caused by a cold

D. Indigestion

E. Stump and crown stimulation

13. 1999U2A1009 Possible symptoms of a patient with geographic tongue are ( )

A. Spontaneous pain

B. Dry mouth

C. Hot and cold pain

D. Burning, irritation, pain

E. Paroxysmal pain

14.2015U2A1020 The most common cause of tongue papilla atrophy is ( )

A. Drug allergies

B. Viral infection

C. Local trauma

D. Chronic pharyngitis

E. Vitamin B₁₂ deficiency

15.2016U2A1061 The most common cause of atrophic glossitis is ( )

A. Vitamin C deficiency

B. Spirochetal infection

C. Viral infection

D. Bacterial infection

E. Iron deficiency anemia

16.2019U2A1005 Common causes of atrophic glossitis are ( )

A. Poor denture irritation

B. Developmental malformations

C. Poor oral environment

D. Anemia

E. Mental factors

17.2019U2A2079 Female, 6 years old. There is an indefinite circular line spot on the back of the tongue, and the examination shows that there is a round filamentous papilla peeling area on the left side of the middle of the back of the tongue, the color is red and slightly depressed, there is no pain, the diameter is about 10mm, the edge is whitish, slightly convex, and the tongue is moving normally. No abnormalities were found throughout the body. Should be diagnosed as ( )

A. Drug-allergic stomatitis

B. Coccal stomatitis

C. Contact stomatitis

D. Lichen planus

E. Map tongue

· B. Cracked tongue, facial paralysis, granulomatous cheilitis

· D. Diurnal "wandering" conjugation features

· A. The red zone can be eroded

· B. Glyphical tongue, facial paralysis, glandular cheilitis

· E. Traumatic factors

· C. Congenital inheritance

· E. Chronic cheilitis

· E. Stretching time is too long during oral treatment

· A. May-Roe syndrome

· B. Bad habit of licking and biting lips

· B. Vitamin B₂

· A. Bad habit of licking lips

· D. Burning, irritation, pain

· E. Vitamin B₁₂ deficiency

· E. Iron deficiency anemia

· D. Anemia

· E. Map tongue

**Chapter 9 Oral Characterization of Sexually Transmitted Diseases**

1. 2004U2A1013 Syphilis mucosal plaques are manifested as ( )

A. Reddish-brown smooth patches

B. Brownish-yellow bright patches

C. Grayish-white shiny patches

D. Grayish-white keratinized plaques

E. Yellowish-white grayish plaques

2.2009U2A1032 Syphilis gum swelling as a kind ( )

A. Connective tissue

B. Lymphoid tissue

C. Mesenchymal tissue

D. Granulation tissue

E. Vascular tissue

3.2000U2AN119 male, 50 years old. White patches in the mouth. Examination: grayish-white, smooth, slightly raised plaques on the left mucosa and palate of the oral cavity, and scattered rosé-red macules on both forearms. Low-grade fever, headache. The patient reports an ulcer on the penis, which has since healed. The initial impression of the disease is ( )

A. Discoid lupus erythematosus

B. Erythema multiforme

C. lichen planus

D. Oral leukoplakia

E. Syphilis

4.2000U2AN120 male, 50 years old. White patches in the mouth. Examination: grayish-white, smooth, slightly raised plaques on the left mucosa and palate of the oral cavity, and scattered rosé-red macules on both forearms. Low-grade fever, headache. The patient reports an ulcer on the penis, which has since healed. The initial impression of the disease is syphilis, and the items that need to be tested for further diagnosis are ( )

A. Rapid Hemagglutination Response (RPR)

B. Tzack cell test

C. Rheumatoid factor

D. Tuberculin test

E. HIV antibody test

5.2000U2AN121 male, 50 years old. White patches in the mouth. Examination: grayish-white, smooth, slightly raised plaques on the left mucosa and palate of the oral cavity, and scattered rosé-red macules on both forearms. Low-grade fever, headache. The patient reports an ulcer on the penis, which has since healed. The initial impression of the disease is syphilis, and the preferred treatment is ( )

A. nystatin

B. Kanamycin

C. Streptomycin

D. Penicillin

E. Erythromycin

6. 2012U2A1005 Diagnostic criteria for secondary syphilis, one of the incorrect descriptions is ( )

A. The course of the disease is more than two years

B. History of unclean sexual intercourse or chancre

C. Multiple rashes with generalized lymphadenopathy

D. Treponema pallidum detected

E. Positive serotest for syphilis

7.2004U2A1022 Stillbirth or preterm birth is more common in ( )

A. Primary syphilis

B. Secondary syphilis

C. Stage 3 syphilis

D. Early congenital syphilis

E. Late congenital syphilis

8.2004U2A1023 When can syphilis chancre heal spontaneously ( )

A. 3~8 weeks after infection

B. 4~9 weeks after infection

C. 5~10 weeks after infection

D. 6~11 weeks after infection

E. 7~12 weeks after infection

9.2004U2A1024 Which of the following descriptions of gonorrhea is wrong ( )

A. Women are more susceptible to infection than men

B. Transmission is mainly through sexual intercourse

C. is an acute or chronic infectious disease

D. About 60% of women are asymptomatic after infection

E. Diagnosis is primarily based on biopsy

10.2004U2A1026 The erroneous statement about syphilis chancre is ( )

A. Obvious tenderness

B. Covered with a thin brownish-yellow crust

C. Lesions contain pathogens

D. Touching like a coin or cartilage

E. Round or oval single ulcer

11. 2010U2A1019 Non-specific tests for the diagnosis of syphilis are ( )

A. Spirochetal examination

B. Fahrenheit test

C. Conn's test

D. Flocculation slide method

E. Peripheral blood routine white blood cell count

12.2013U2A1002 Syphilis Yes ( )

A. Metabolic diseases

B. Endocrine diseases

C. Allergic diseases

D. Immunodeficiency disorders

E. Communicable diseases

· C. Grayish-white shiny patches

· D. Granulation tissue

· E. Syphilis

· A. Rapid hemagglutination reaction (RPR).

· D. Penicillin

· A. The course of the disease is more than two years

· E. Late congenital syphilis

· A. 3~8 weeks after infection

· E. Diagnosis is primarily based on biopsy

· A. Obvious tenderness

· E. Peripheral blood routine white blood cell count

· E. Communicable diseases

**Chapter 10 AIDS**

1.2009U2A1017 The most common oral manifestations in HIV-infected patients ( )

A. Herpes simplex

B. Candidiasis

C. Herpes zoster

D. Hairy leukoplakia

E. Kaposi's sarcoma

2.2009U2A1020 Which of the following is NOT an AIDS preventive measure ( )

A. Avoid sexual disorders

B. Strict quarantine to prevent importation

C. Strengthen surveillance of high-risk populations

Avoid daily contact with people living with HIV/AIDS

E. Strict screening of blood donors and blood products

3.2004U2A1036 Which of the following is NOT an oral manifestation of HIV infection ( )

A. Pemphigus

B. Herpes simplex

C. Candidiasis

D. Kaposi's sarcoma

E. Necrotizing gingivitis

4. 2010U2A1032 The following is a description of the clinical manifestations of hairy leukoplakia, except for ( )

A. It is usually bilateral

B. There are hairy protrusions on the surface

C. Can be wiped off with a cotton swab

D. Generally asymptomatic and asymptomatic

E. Mainly on the edge of the tongue and the abdomen of the tongue

5.2002U2A1021 Infection-related lesions in the oral cavity of AIDS patients include ( )

A. Oral candidiasis

B. Oral hairy leukoplakia

C. Kaposi's sarcoma

D. AIDS-related periodontitis

E. All of the above

6. 2014U2A1013 HIV is characterized by ( )

A. High temperature resistance

B. Not resistant to high temperatures

C. High concentration in saliva

D. Easy to survive outside the body

E. Only in blood and semen

7.2014U2A1014 AIDS patients may have the following oral manifestations in the oral cavity, but excluding ( )

A. Kaposi's sarcoma

B. Candidiasis

C. Necrotizing gingivitis

D. Wegne's granuloma

E. Non-Hodgkin lymphoma

8. 2014U2A2095 patient, male, 20 years old. AIDS due to drug use, HIV (+). The examination found that there were ulcers on the surface of the gingival margin and buccal mucosa of the lower anterior teeth, the surface was covered with gray-white filthy pseudomembrane, the gingival papilla disappeared, and the halitosis was obvious. Should be diagnosed as ( )

A. Rapidly progressive periodontitis

B. Necrotizing stomatitis

C. Leukemia

D. Exfoliative gingivitis

E. Agranulocytopenia

· B. Candidiasis

· Avoid daily contact with people living with HIV/AIDS

· A. Pemphigus

· C. Can be wiped off with a cotton swab

· E. All of the above

· B. Not resistant to high temperatures

· D. Wegne's granuloma

· A. Fast forward

**Pediatric Dentistry**

**Chapter 3 Anatomical and Structural Characteristics of Deciduous Teeth and Young Permanent Teeth**

1.2014U2A1005 Which of the following does not cause pathological root resorption ( )

A. Pulpitis of deciduous teeth

B. Periapical inflammation of deciduous teeth

C. Dental trauma

D. Vital myelinotomy

E. Tooth eruption

2.2010U2A1006 Root resorption of deciduous teeth in the replacement stage belongs to ( )

A. Physiological absorption

B. Pathologic absorption

C. Physiological destruction

D. Pathological destruction

E. None of the above

3.2010U2A1007 Purpose of treatment of deciduous teeth ( )

A. Preserve the function of the tooth as much as possible and avoid premature tooth extraction

B. Maintain endodontic life as much as possible

C. Promote root tip formation as much as possible

D. Prevent premature spontaneous replacement of baby teeth as much as possible

E. All of the above

4.2005U2A1005 Which of the following descriptions of baby teeth is correct ( )

A. Intact deciduous dentition aids in digestion and absorption in children

B. Healthy baby teeth stimulate the normal development of the jaw

C. Premature loss of baby teeth is one of the causes of dentition deformity

D. Properly positioned and healthy baby teeth can guide the normal eruption of permanent teeth

E. All of the above are true

5.2005U2A1006 The time required for most permanent teeth to reach the occlusal plane after eruption is about ( )

A. 2~3 months

B. 4~6 months

C. 7~12 months

D. About 1 year

About 2 years

6.2005U2A1007 Teeth generally begin to erupt when the root is formed ( )

A. About 1/4

B. About 1/3

C. About 1/2

D. About 2/3

E. All formed

7.2009U2A1007 After tooth eruption, the root continues to develop until the root is fully formed.

A. Within half a year

B. 1 year

C. 1~2 years

D. 2~3 years

E. 3~5 years

8.2009U2A1008 Young permanent teeth refer to ( )

A. Newly erupted permanent teeth

B. Permanent teeth that have not yet erupted

C. Permanent teeth in young people

D. Permanent teeth that have erupted, but the root of the tooth has not yet fully developed

E. None of the above

9.2002U2A1007 The hard tissue of the crown of young permanent teeth is thinner than that of adult permanent teeth, mainly due to ( )

A. Low degree of calcification

B. Thick dentinal tubules

C. No secondary dentin

D. Dentin is not yet fully formed

E. Thin enamel

10.2010U2A1008 Endodontic characteristics of young permanent teeth are ( )

A. Loose tissue

B. Abundant blood vessels

C. Strong defense and repair mechanism

D. Strong osmotic effect

E. Hyperplasia is significant

11.2005U2A1008 Pulp characteristics of young permanent teeth ( )

A. Strong defense and repair response, and the phenomenon of proliferation is significant

B. Loose tissues and abundant blood vessels

C. Strong osmotic effect

D. A and B

E. A, B, C

12.2011U2A1004 When there is inflammation or necrosis in the pulp chamber of a young permanent tooth, part of the pulp tissue or root canal pulp in the pulp may still be alive because ( )

A. The pulp tissue of young permanent teeth is loose and the blood vessels are abundant

B. More myeloid cells and undifferentiated mesenchymal cells

C. Strong defense and repair functions

D. The exudative effect is weak, and the hyperplasia phenomenon is more significant

E. A+B+C+D

13.2006U2A1001 The hard tissue of the crown of young permanent teeth is thinner than that of mature permanent teeth, the main reason is ( )

A. Low degree of calcification

B. Thin enamel

C. Tooth enamel is not fully developed

D. No secondary dentin

E. Dentin is not yet fully formed

14.2014U2A2080 Female, 8 years old. The right buccal mucosa was ruptured for 2 weeks. Oral examination showed that the lower right V. residual crown, loose I.°, the apex was exposed from the attached gingiva, a 1 cm diameter ulcer was visible on the right cheek mucosa, the periphery was red, and the lower right 6 had erupted. The best treatment is ( )

A. Dry myeloid surgery

B. Root canal treatment

C. Extraction

D. Upper space maintainer after extraction, wound healing

E. Oral antibiotics with 2% iodine glycerol topically for ulcers

15.2009U2A1009 The main reasons for bone resorption at the root bifurcation caused by pulp infection of deciduous teeth are ( )

A. The pulp floor of deciduous teeth is thin and prone to breakage

B. Poor calcification of deciduous teeth, the floor of the pulp chamber is susceptible to caries perforation

C. Deciduous tooth resorption begins at the root bifurcation

D. Multiple root canals at the floor of the pulp of the deciduous tooth leading to the root bifurcation

E. All of the above

16.2016U2A1058 During physiological resorption of the root of deciduous teeth, the crown pulp is still normal, and the pulp cells of the root canal are degenerated, and the period of disappearance is ( )

A. When the root of the tooth begins to resorb

B. Root resorption: 1/4

C. Root resorption 1/2

D. Root resorption3/4

E. Loss of baby teeth

17.2017U2A1041 In general, the eruption of young permanent teeth is the beginning of root formation ( )

A. 1/4

B. 1/3

C. 2/3

D. 3/4

E. 2/5

· E. Tooth eruption

· A. Physiological absorption

· E. All of the above

· E. All of the above are true

· B. 4~6 months

· B. About 1/3

· C. 1~2 years

· D. Permanent teeth that have erupted, but the root of the tooth has not yet fully developed

· E. Thin enamel

· D. Strong osmotic effect

· D. A and B

· E. A+B+C+D

· B. Thin enamel

· C. Extraction

· E. All of the above

· D. Root resorption3/4

· B. 1/3

**Chapter 4 Growth and Development of Children's Craniofacial and Dentition**

1.2002U2A1011 The eruption time of the second permanent molars is ( )

A. 2~3 years old

B. 6~7 years old

C. 12~13 years old

D. 18~21 years old

E. 5~6 years old

2.2005U2A1009 Common eruption order of baby teeth ( )

A. A→B→C→D→E

B. A→B→D→C→E

C. A→B→C→E→D

D. A→B→D→E→C

E. A→C→B→D→E

3.2014U2A1007 The order of eruption of baby teeth is ( )

A. Ⅴ→Ⅰ→Ⅱ→Ⅲ→Ⅳ

B. Ⅰ→Ⅱ→Ⅴ→Ⅳ→Ⅲ

C. Ⅰ→Ⅱ→Ⅳ→Ⅲ→Ⅴ

D. Ⅰ→Ⅱ→Ⅲ→Ⅴ→Ⅳ

E. Ⅰ→Ⅱ→Ⅴ→Ⅲ→Ⅳ

4.2009U2A1011 Which of the following is NOT a sign of a deciduous tooth approaching the replacement stage ( )

A. Significant damage to the crown

B. Clinical III.° loosening

C. The root of the permanent tooth germ is half formed

D. The position of the permanent tooth germ is close to the root bifurcation of the deciduous tooth

E. Root resorption of primary teeth has reached 1/2

5.2014U2A1008 Tooth eruption without replacement is ( )

A. Permanent incisors

B. Permanent caninees

C. Constant premolars

D. Permanent molars

E. Constant bicuspids

6.2009U2A1012 ( )

A. Babies born at 4 months of age

B. Infants born at 6 months of age

C. Infants 8 months of age

D. Babies born at 10 months of age

E. Babies born 12 months of age

7.2020U1A1016 Incorrect Occlusal Features About Mixed Teeth ( )

A. The upper lip frenulum is too low

B. Interdental space of the central incisors

C. Temporary crowding of anterior teeth

D. Transient mesial occlusion

E. Temporary overbite

· C. 12~13 years old

· D. A→B→D→E→C

· C. Ⅰ→Ⅱ→Ⅳ→Ⅲ→Ⅴ

· E. Root resorption of primary teeth has reached 1/2

· D. Permanent molars

· E. Babies born 12 months of age

· E. Temporary overbite

**Chapter 5 Abnormal Dental Development**

1.2006U2A1004 Abnormal tooth morphology excl. ( )

A. Deformed central cusp

B. Fusion teeth

C. Dental fluorosis

D. Too small teeth

E. Curved teeth

2.2014U2A2085 A 12-year-old female suddenly developed pain in the bite of the left lower tooth, spontaneous pain for 1 day without obvious cause, and worsened at night. Oral examination: permanent teeth, teeth aligned, good oral hygiene, soft scale (-), tartar (-), lower left 5 percussion pain (++), not loose, the most likely cause of pain is ( )

A. Occult caries

B. Cracked teeth

C. Deformed central cusp

D. Acute infection of jaw cysts

E. Amelocytoma complicated with infection

3.2009U2A1013 The following are not dental structural abnormalities ( )

A. Enamel hypoplasia

B. Inverted teeth

C. Dentin hypoplasia

D. Congenital syphilitis teeth

E. Dental fluorosis

4.2006U2A1005 The following statements about the eruption of permanent canines are correct ( )

A. The most common is the ectopic labial eruption of the mandibular canine

B. The location of ectopic eruption of permanent canines is variable

C. Apical periodonymology of the first deciduous molars does not affect the normal eruption of the permanent canine

D. None of the above

E. All of the above are true

5.2006U2A1006 The following incorrect statement about low deciduous teeth is ( )

A. Low baby teeth cannot be replaced properly

B. Low primary teeth are more likely to occur in maxillary second primary molars

C. Trauma can induce the formation of low baby teeth

D. Low deciduous teeth, also known as deciduous adhesions

E. Low primary teeth should be extracted promptly

6. 1999U2A1004 Which of the following is wrong ( )

A. Solid teeth can be 1~3mm lower than the occlusal surface

B. Permanent teeth that do not erupt in their normal position are called ectopic eruptions

C. Eruption of baby teeth more than 1 year later than the average eruption time is called late eruption

D. Birth teeth are extra teeth

E. The root of the permanent tooth is not fully developed when it erupts

7. 2010U2A2103 male, 7 years old. Complaints of loosening of mandibular anterior teeth. Oral examination showed that the right lower deciduous incisor was loose III.°, the crown had a deep cavity, and the lower right 1 had erupted from the lingual side, and the diagnosis ( )

A. Retention of baby teeth

B. Premature loss of baby teeth

C. Apical periodontitis of deciduous teeth

D. Periodontitis of deciduous teeth

E. Trauma to deciduous teeth

8.2009U2A1014 Congenital Missing Teeth can be divided into ( )

A. Individual, partial, and total edentulous missing

B. Edentulism, Edentulism, Edentulism

C. Missing teeth in the upper jaw, missing teeth in the lower jaw, and missing teeth in the whole mouth

D. Both A and B are right

E. Both A and C are right

9. 2009U2A1015 The description of congenital edentulism is wrong ( )

A. Mostly dominantly inherited

B. Genetic disease

C. Permanent and baby teeth can be affected

D. Often a manifestation of ectodermal hypoplasia

E. Having few or no teeth in the mouth

10.2019U2A2080 Child, female, 2 years old, maxillary papillary central incisors are missing, and deciduous tooth germs can be seen on X-ray, which should be diagnosed as ( )

A. Delayed eruption of baby teeth

B. Dental fixation

C. Retention of baby teeth

D. Ectopic eruption

E. Impacted permanent teeth

11.2018U2A2079 Inherited permanent teeth do not erupt for a long time, the deciduous teeth remain in the permanent dentition, and the permanent tooth germ is not seen on X-ray, which should be diagnosed as ( )

A. Delayed eruption of permanent teeth

B. Dental fixation

C. Retention of baby teeth

D. Ectopic eruption

E. Missing permanent teeth

12.2017U2A1043 The position of early eruption of deciduous teeth is more common in ( )

A. Maxillary mammary central incisors

B. Mandibular mammary central incisors

C. Maxillary mastoid incisors

D. Mandibular deciduous canines

E. First deciduous molars

13.2017U2A1042 Regarding the identification of premature eruption of primary teeth and "epithelial beads", the following statement is correct ( )

A. Epithelial beads, like premature eruptions, have the basic structure of the teeth

B. Epithelial beads are formed by the remnants of the dental plate, which can also differentiate to form premature eruptions

C. The epithelial beads, like the early eruption of teeth, can be removed automatically

D. Epithelial beads are supported by alveolar bone, whereas early eruption teeth are not supported by alveolar bone

E. Ethelial beads, like early eruptions, have a crown shape, but the epithelial beads are grayish-white

· C. Dental fluorosis

· A. Occult caries

· E. Dental fluorosis

· E. All of the above are true

· E. Low primary teeth should be extracted promptly

· E. The root of the permanent tooth is not fully developed when it erupts

· E. Trauma to deciduous teeth

· D. Both A and B are right

· A. Mostly dominantly inherited

· A. Delayed eruption of baby teeth

· E. Missing permanent teeth

· A. Maxillary mammary central incisors

· D. Epithelial beads are supported by alveolar bone, whereas early eruption teeth are not supported by alveolar bone

**Chapter VII Caries in Children**

1.2014U2A2078 Dai, male, 8 years old, 2 months old, left upper back tooth food impaction, hot and cold stimulation pain. No history of spontaneous pain, untreated. Examination: there is a large amount of soft caries in the cavity of the occlusal surface, probing (++), percussion (-), and not piercing the pulp. X-rays show deep caries near the medulla, and the apex is not fully formed. The first treatment that should be considered is ( )

A. Root canal treatment

B. Apical inductionplasty

C. Endomedulla therapy

D. Deep caries remineralization therapy

E. Myelotomy

2. 2004U2A1012 The time of remineralization of softened dentin in deep caries treatment of young permanent teeth is approximately ( )

A. 2~4 weeks

B. 10~12 weeks

C. 1~2 months

D. 4~6 months

About 2 years

3. 2014U2A1002 The main objectives of permanent tooth filling and restoration treatment in mixed dentition stage are ( )

A. Restore dental contact points

B. Restoration of tooth anatomy

C. Restore tooth height

D. A+B

E. A+C

4.2011U2A1017 The following are not suitable for plasticization therapy ( )

A. Baby teeth

B. Young permanent teeth

C. Mature permanent anterior teeth

D. Mature permanent posterior teeth

E. A, B, C

5.2014U2A1003 Earliest age of caries in deciduous teeth ( )

A. 6 months after birth

B. 1 year old

C. 3 years old

D. 6 years old

E. 7~8 years old

6. 2009U2A1003 The age at which the prevalence of caries in children peaked was ( )

A. 2~5 years old

B. 5~8 years old

C. 6~12 years old

D. 15~26 years old

7.2011U2A1002 The tooth surface of maxillary deciduous teeth that is most susceptible to caries is ( )

A. Primary incisors mesial and labial surfaces

B. Labial surface of deciduous caninees

C. Distal and mesial surfaces of deciduous caninees

D. First deciduous molar occlusal and distal median surface

E. Second deciduous molar occlusal and distal median surface

8.2005U2A1001 Characteristics of caries in deciduous teeth at different ages ( )

A. 1~2 years old, mainly occurs on the labial and adjacent surfaces of maxillary deciduous anterior teeth

B. 3~4 years old, mostly on the adjacent surface of deciduous molars

C. 4~5 years old, more likely to occur in the pits and fissures of the occlusal surface of the deciduous molars

D. Around 6 years of age, it tends to occur in the pits and fissures and adjacent surfaces of the occlusal surface of the deciduous molars

E. All of the above are true

9.2005U2A1002 Breastfeeding young children who are bottle-fed are sometimes susceptible ( )

A. Acute caries

B. Chronic caries

C. Bottle caries

D. Resting caries

E. Rampant caries

10. 2011U2A1003 Baby teeth often produce caries along the neck of the tooth around the crown ( )

A. Bottle caries

B. Resting caries

C. Rampant caries

D. Dry caries

E. Annular caries

11.2005U2A1003 Effects of caries in deciduous teeth on the oral cavity include ( )

A. Affects chewing function

B. Infection of lesions in other parts of the body caused by lesions as lesions

C. It is easy to cause caries of new permanent teeth

D. Can form a clinically seen Turner tooth

E. All of the above

12.2005U2A1004 Predisposing factors for caries in deciduous teeth ( )

A. Thin enamel and dentin

B. Low salinity and weak acid resistance

C. Children's food is mostly soft, sticky and high in sugar

D. Poor self-cleaning and cleaning ability of children

E. All of the above

13.2002U2A1003 Which is wrong in the description of the clinical manifestations of caries in deciduous teeth ( )

A. High caries rate and early onset

B. Tooth decay is frequent and the extent of caries is extensive

C. Decay develops rapidly

D. Inactive formation of restorative dentin

E. Symptoms are not obvious

14.2009U2A1004 Which of the following drugs will make the tooth surface black after rubbing, and cannot be used for permanent anterior teeth ( )

A. 2% sodium fluoride

B. 8% stannous fluoride

C. Acidic fluorophosphoric acid

D. 10% silver ammonia fluoride

E. 10% ammonium molybdate fluoride

15.2014U2A1004 Principles for the treatment of caries in deciduous teeth do not include ( )

A. Reduce occlusal height

B. Removal of diseased tissue

C. Restore the shape of the tooth

D. Improves chewing function

E. Conducive to the formation of permanent dentition

16.2009U2A2106 Extensive superficial caries on the proximal surface of primary anterior teeth and extensive superficial caries on the occlusal surface of deciduous molars, the treatment to be used by permanent tooth replacement within 1 year is ( )

A. Chemotherapy

B. Remineralization

C. Pit and fissure sealing

D. Preventive filling

E. Extraction

17.2002U2A1004 Drugs that cannot be used in particularly uncooperative children are ( )

A. 2% sodium fluoride

B. 8% stannous fluoride

C. Sodium acidic fluorophosphate

D. 38% ammonia fluoride silver

E. 10% ammonium molybdate fluoride

18.2009U2A1005 Drugs used in the treatment of extensive superficial caries of deciduous teeth do not include ( )

A. 2% sodium fluoride

B. 8% stannous fluoride

C. Silver nitrate

D. 38% ammonia fluoride silver

E. 30% hydrogen peroxide solution

19.2002U2A1005 Multiple Tooth Crowns of Deciduous Teeth Are Seriously Damaged, Special Attention Should Be Paid to Filling Treatment ( )

A. Restore touchpoints

B. Restoration of cusp mosaicism

C. Restore occlusal height

D. Prevent the cracks of the cheek and tongue walls

E. All of the above

20.2009U2A1006 Baby tooth II. type hole under what circumstances can be prepared into a stepless compound hole ( )

A. When the contact point of the deciduous teeth is close to the occlusal surface

B. When the gingival margin of the deciduous tooth is close to the occlusal surface

C. When the decay of the deciduous teeth is closer to the occlusal surface

D. When the deciduous teeth are eroded and the gingival margin is close to the occlusal surface

E. When the contact point of deciduous teeth, gingival margin or caries is close to the occlusal surface

21.2010U2A2101 The child has multiple decayed teeth in the oral cavity.

A. Amalgam filling

B. Removable retainer

C. Energetic activity retainers

D. Metal crowns to restore teeth

E. Fixed gap holders

22.2010U2A1005 The tooth that is not suitable for the treatment of tooth defects by prosthetic restoration method is ( )

A. Maxillary permanent incisors

B. Mandibular permanent incisors

C. Constant bicuspids

D. Permanent molars

E. Baby teeth

23.2014U2A2079 Male, 10 years old. Pain in the left upper back tooth when drinking ice water. No spontaneous pain. Oral examination: left upper V. occlusal surface with extensive caries, hard cavity at the bottom of the cavity. Percussion (-), probing (-), no loosening. The most appropriate way to deal with it is ( )

A. Chemotherapy

B. Remineralization

C. Pit and fissure sealing

D. Restorative treatment

E. Uprooting

24.2002U2A1006 Baby bottle caries is good in ( )

A. Smooth surface of maxillary decidal incisors and occlusal surface of first deciduous molars

B. Smooth surface of mandibular deciduous incisors and occlusal surface of first deciduous molars

C. Smooth surface of maxillary anterior teeth and occlusal surface of molars

D. Smooth surface of mandibular anterior teeth and occlusal surface of molars

E. All baby teeth

25.2006U2A1020 Young permanent teeth in mixed dentition stage should be ( )

A. Pay attention to restoring occlusal height

B. Metal crowns cannot be used for repair

C. Use glass ionic cementing powder for permanent filling, and pay attention to restoring the contact points between teeth

D. For the purpose of restoring the anatomical morphology of the crown, no emphasis is placed on restoring the contact points between the teeth

E. Pay attention to the complete removal of the diseased tissue, and restore the shape of the tooth and the occlusal relationship

26.2009U2A1081 Purpose of Filling Restoration Treatment of Permanent Teeth in Mixed Dentition Stage ( )

A. Restore dental proximal contacts

B. Restoration of crown anatomy

C. Restore tooth height

D. A+B

E. A+C

27.2018U2A1001 Caries is more common in children at the age of 4~5 years ( )

A. Occlusal surface of deciduous molars

B. Adjacent surfaces of deciduous molars

C. Adjacent surface of maxillary deciduous anterior teeth

D. Labial surface of maxillary deciduous anterior teeth

E. Buccal surface of deciduous molars

28.2017U2A1047 Baby Tooth Restoration Treatment Which of the following descriptions is wrong ( )

A. Extensive caries can also be repaired with resin

B. Calcium hydroxide can be used to protect the marrow near the medulla

C. Must be double-layered

D. Glass ions are an ideal filling material

E. Both anterior and posterior teeth can be filled with composite resin

29.2017U2A1046 The base material that cannot be used in the filling treatment of deciduous tooth caries is ( )

A. Zinc polycarboxylate cohesive powder

B. Zinc phosphate cement

C. Vitreous ionic cement

D. Clove oil cement powder

E. Hard calcium hydroxide

30.2017U2A1044 Common types of caries in deciduous teeth ( )

A. Acute caries, dry caries, annular caries

B. Chronic caries, resting caries, bottle caries

C. Acute caries, annular caries, bottle caries

D. Bottle caries, dry caries, annular caries

E. Wet caries, annular caries, chronic caries

31.2017U2A1045 Bottle caries is most common in ( )

A. Maxillary deciduous incisors

B. Mandibular deciduous incisors

C. Maxillary deciduous molars

D. Mandibular deciduous molars

E. All baby teeth

· C. Endomedulla therapy

· B. 10~12 weeks

· D. A+B

· E. A, B, C

· B. 1 year old

· B. 5~8 years old

· D. First deciduous molar occlusal and distal median surface

· E. All of the above are true

· C. Bottle caries

· E. Annular caries

· E. All of the above

· E. All of the above

· D. Inactive formation of restorative dentin

· D. 10% silver ammonia fluoride

· A. Reduce occlusal height

· C. Pit and fissure sealing

· D. 38% ammonia fluoride silver

· A. 2% sodium fluoride

· E. All of the above

· E. When the contact point of deciduous teeth, gingival margin or caries is close to the occlusal surface

· **D. Metal crowns to restore teeth**

· **E. Baby teeth**

· **D. Restorative treatment**

· **A. Smooth surface of maxillary decidal incisors and occlusal surface of first deciduous molars**

· **C. Use glass ionic cementing powder for permanent filling, and pay attention to restoring the contact points between teeth**

· **D. A+B**

· **A. Occlusal surface of deciduous molars**

· **B. Calcium hydroxide can be used to protect the marrow near the medulla**

· **D. Clove oil cement powder**

· **B. Chronic caries, resting caries, bottle caries**

· **A. Maxillary deciduous incisors**

**Chapter 8 Endodontic and Apical Diseases in Children**

1.2014U2A1001 The main causes of periapical disease of deciduous teeth are ( )

A. Trauma to primary teeth

B. Severe abrasion

C. Intramedullary infection

D. Chemical burns

E. Congenital malformations

2.2004U2A1001 Regarding the description of the gingival fistula tract of the deciduous teeth, the following is correct ( )

A. Chronic apical periodontitis of deciduous teeth has fistula foramen near the affected tooth

B. The fistula foramen may appear on the gingival mucosa on both sides of the periroot of the affected tooth, or it may appear at the site of the root divergence of the affected tooth

C. The fistula hole is constantly leaking pus, and the fistula does not close after it appears

D. The presence of a fistula indicates complete necrosis of the pulp of the affected tooth

E. Endodontictomy can be performed on a trial basis for the tooth with fistula

3.2000U2AN115 male, 7 years old. Pain in the right lower back tooth for 1 week and swollen face for 3 days. Examination: large area of caries, percussion pain (+++), shallow gingival and buccal groove, palpable fluctuation, palpatory pain (+++), deep cavity on the mesial occlusal surface, percussion (-), temperature test as control tooth. X-ray showed that IV. Root divergence and large area of low-density shadow, distal root resorption 2/3, permanent tooth embryo sac discontinuous, and the upper bone plate was blurred. Caries proximal medulla, bony hard plate continuous. The initial treatment is to remove the primer, and the diagnosis is ( )

A. Medium caries

B. Deep caries

C. Reducible pulpitis

D. Pulp congestion

E. Chronic apical periodontitis

4.2004U2A1002 The treatment of choice for deciduous teeth with deep caries proximal pulp is ( )

A. Fill directly after the bottom pad

B. Endodonticotomy

C. Dry pulp surgery

D. Indirect capping

E. B or C

5.1999U2A1003 Which is true about the description of the indications for myelotoplasty ( )

A. Reducible pulpitis caused by deep caries

B. Accidental puncture of the pulp during cavity preparation for tooth decay

C. Deep caries exposes the marrow after removing the putus

D. Acute reducible pulpitis of permanent teeth in young people

E. All of the above are correct and correct

6.2009U2A1002 Deep caries of deciduous teeth, after removing caries and accidentally exposing the pulp, it is best to use ( )

A. Dry myeloid surgery

B. Direct capping

C. Indirect medullary capping

D. Pulpectomy

E. Root canal treatment

7. 2004U2A1003 When performing FC myelotomy in 6-year-old children, the time for FC small cotton balls to be placed is generally ( )

A. 1 minute

B. 2 minutes

C. 3 min

D. 5 minutes

E. 10 minutes

8.2013U2A1001 Root canal filling material for deciduous teeth is often used ( )

A. Calcium hydroxide preparations

B. Zinc oxide clove oil paste + teether tip

C. Calcium hydroxide preparation + teether tip

D. Calcium hydroxide preparation + silver tip

E. Zinc oxide clove oil paste + silver tip

9.2004U2A1004 The effect of formaldehyde cresol on the pulp tissue of the pulp tissue in the FC pulp detotomy of deciduous teeth is ( )

A. Promote calcification of pulp tissue

B. Promote the repair of pulp tissue

C. Protect the pulp from bacterial infection

D. Pulp necrosis caused by pulp vascularization

E. Coagulative necrosis occurs in the pulp cross-sectional contact zone

10.2004U2A1005 In FC pulp detoxification of deciduous teeth, the paste covering the pulp section is ( )

A. Pure zinc oxide paste

B. Equal amount of FC and iodoform, calcium hydroxide paste

C. Equal parts of zinc oxide paste mixed with FC and clove oil

D. 3 parts clove oil + 1 part FC zinc oxide paste

E. 3 parts FC + 1 part zinc oxide paste mixed with clove oil

11. 2013U2AN138 An 8-year-old patient who fell and caused the right upper 1 crown to bend obliquely, and immediately came to the hospital for treatment. Oral examination reveals a large perforation and percussion (+). The treatment of choice is myelinotomy, and the key to success in this treatment is ( )

A. Selection of capping agent

B. Aseptic procedure

C. Correct marrow opening

D. Removal of slightly more healthy coronary medulla

E. Correct selection of temporary sealant

12. 2013U2A2136 The 8-year-old child had a distal and deep cavity in the right upper central incisor, and there was no abnormality on percussion, and the gingiva was normal. Do not loosen. Treatment: decomposing clean, deep, close to the medulla. Which treatment is better ( )

A. Zinc oxide clove oil paste indirectly caps the pith

B. Secondary derotting

C. Cutting of the living medulla

D. Apical inductionplasty

E. Indirect capping of the medulla

13.2010U2A1003 The following are not indications for endodonticotomy ( )

A. Traumatic crown exposes the pulp of young permanent teeth

B. Deep caries to remove decay and expose the pulp foramen ≤ 0.5 mm of young permanent teeth

C. Mature permanent teeth with localized pulpitis

D. Accidentally exposed young permanent teeth

E. None of the above

14. 2013U2A2072 A 9-year-old child presents with discolored teeth of the left upper incisor. Examination: crown fracture dentin exposed, tooth discoloration. No response to hot and cold measurements, X-ray shows apical flare, bone plate discontinuity, which of the following items is the most important ( )

A. Pull out the marrow, do not come out of the apical hole

B. Completely remove the infected material in the root canal, eliminate inflammation, and protect the tooth papilla

C. Do not seal FC and other irritating drugs in the root canal

D. Fill with calcium hydroxide paste, do not overfill

E. Regularly review and change paste

15.2010U2A1004 Whether the apical closure of young permanent teeth whose roots cannot continue to develop after treatment depends entirely on ( )

A. Condition of the medulla remnant at the apex

B. Periapical tissue health

C. Role of calcium hydroxide

D. Control of inflammation within the root canal

E. All of the above

16.2000U2AN116 The child was 10 years old, and the upper anterior teeth and gums often had pus for more than 1 month. Check the left upper 1 distal tongue surface deep caries, probe no response, no perforation, tooth loosening I. degree, percussion (+), cold measurement unresponsive, labial gingiva near the apex of a sinus tract. To confirm the diagnosis, the tests that need to be done clinically are ( )

A. Electrical vitality test

B. Puncture

C. Local anesthesia

D. X-rays

E. Smear

17.2000U2AN117 The 10-year-old child had pus from the upper anterior teeth and gums for more than 1 month. Check the left upper 1 distal tongue surface deep caries, probe no response, no perforation, tooth loosening I. degree, percussion (+), cold measurement unresponsive, labial gingiva near the apex of a sinus tract. Clinically probable diagnosis of ( )

A. Acute pulpitis

B. Chronic pulpitis

C. Acute apical periodontitis

D. Chronic apical periodontitis

E. Periodontal abscess

18.2000U2AN118 The child was 10 years old, and the upper anterior teeth and gums often had pus for more than 1 month. Check the left upper 1 distal tongue surface deep caries, probe no response, no perforation, tooth loosening I. degree, percussion (+), cold measurement unresponsive, labial gingiva near the apex of a sinus tract. Clinical treatment should be selected ( )

A. Dry myeloid surgery

B. Vital myelinotomy

C. Root canal therapy

D. Uprooting

E. Plastination therapy

19.2004U2A1006 Periapical tissue disease of deciduous teeth( )

A. It will affect the normal absorption of the root of the deciduous tooth

B. Preventing or delaying the normal replacement of baby teeth

C. Affects the direction of development of inherited permanent tooth germs

D. Affects the normal development of inherited permanent tooth germs

E. All of the above

20.2004U2A1007 Which treatment is best for juvenile periapical disease ( )

A. Dry myeloid surgery

B. Root filling

C. Plasticizing therapy

D. Palliative therapy

E. Air Traffic Tube Therapy

21.2004U2A1008 Description of pain characteristics of endodontic disease and periapical disease of deciduous teeth, the most correct is ( )

A. Pulpitis of primary teeth is generally very responsive to pain caused by various stimuli

B. Acute apical periodontitis is painful with occlusal pain and does not indicate the location of the affected tooth

C. Pulpitis of deciduous teeth has different responses to pain caused by various stimuli, and some have no obvious response to stimuli

D. Patients with pre-existing pulp lesions or pulp necrosis must have had or have had painful symptoms

E. A, B, C ARE ALL RIGHT

22. 2004U2A1009 Observable contents in X-rays of deciduous teeth are ( )

A. Condition and extent of lesions in the periapical tissues of deciduous teeth

B. Calcification of the pulp cavity of deciduous teeth and the number of root canals

C. Pulp status and viability of deciduous teeth

D. Germ development and three-dimensional position of permanent teeth

E. All of the above

23.2004U2A1011 Purpose of pre-scribing X-ray dental film for endodontic treatment of deciduous teeth and young permanent teeth ( )

A. Look at the relative depth of decay and the presence or absence of restorative dentin

B. Whether there is a lesion in the interrhizosphere and the degree of root development of the young permanent teeth

C. Degree of root resorption of deciduous teeth, presence or absence of permanent tooth germs

D. Whether there is calcification in the pulp cavity and whether there is internal absorption

E. All of the above

24.2018U2A1002 Clinically, it is best to use ( ) for the treatment of deep caries in the proximal pulp of deciduous teeth ( )

A. Directly capping the medulla

B. Indirect capping of the medulla

C. Coronary myelectomy

D. Dry marrow surgery

E. Root canal treatment

25.2017U2A1049 The following statements about the characteristics of the clinical manifestations of pulpitis in deciduous teeth are incorrect ( )

A. Deep caries infection is the only cause of endodontic disease in deciduous teeth

B. Pulp disease of deciduous teeth is predominantly characterized by chronic inflammation

C. Deep cavities are often seen clinically

D. Decay that has not been removed from the pulp is often an indication that the pulp is infected

E. Pulp infection of deciduous teeth can be accompanied by root resorption

26.2017U2A1048 The principle of treatment for pulpitis of young permanent teeth is ( )

A. Root canal treatment

B. Apical surgery

C. Apical inductionplasty

D. Treat with myelopreservation as much as possible

E. Uprooting

· **C. Intramedullary infection**

· **B. The fistula foramen may appear on the gingival mucosa on both sides of the periroot of the affected tooth, or it may appear at the site of the root divergence of the affected tooth**

· **B. Deep caries**

· **B. Endodonticotomy**

· **E. All of the above are correct**

· **D. Pulpectomy**

· **C. 3 min**

· **A. Calcium hydroxide preparations**

· **D. Pulp necrosis caused by pulp vascularization**

· **C. Equal parts of zinc oxide paste mixed with FC and clove oil**

· **B. Aseptic procedure**

· **B. Secondary derotting**

· **A. Traumatic crown exposes the pulp of young permanent teeth**

· **B. Completely remove the infected material in the root canal, eliminate inflammation, and protect the tooth papilla**

· **D. Control of inflammation within the root canal**

· **D. X-rays**

· **D. Chronic apical periodontitis**

· **C. Root canal therapy**

· **E. All of the above**

· **B. Root filling**

· **E. A, B, C ARE ALL RIGHT**

· **E. All of the above**

· **E. All of the above**

· **B. Indirect capping of the medulla**

· **A. Deep caries infection is the only cause of endodontic disease in deciduous teeth**

· **D. Treat with myelopreservation as much as possible**

**Chapter 9 Dental Trauma in Children**

1. 2013U2AN139 An 8-year-old patient who fell and caused the right upper 1 crown to be obliquely broken, and immediately came to the hospital for treatment. Oral examination reveals a large perforation and percussion (+). The treatment of choice is ( )

A. Vital myeloidectomy

B. Apical inductionplasty

C. Dry pulp surgery

D. Direct capping

E. Root canal therapy

2. 2009U2A2105 patient 6 years old, left and right upper 1 embedded dislocation, the correct treatment is ( )

A. Root canal treatment was performed 2 weeks after reduction and fixation

B. Root canal treatment was performed 2 days after reduction and fixation

C. Observe and wait for its natural eruption (within half a year) to regularly check the vitality of the pulp (1 year)

D. Root canal treatment: Observe and wait for its natural eruption

E. None of the above

3.2019U2A1008 The most common causes of dental trauma are ( )

A. Falls, collisions

B. Traffic accidents

C. Sports Injuries

D. Violence

E. Use your teeth as tools

· **A. Vital myeloidectomy**

· **C. Observe and wait for its natural eruption (within half a year) to regularly check the vitality of the pulp (1 year).**

· **A. Falls, collisions**

**Chapter 10 Periodontal Tissue Diseases and Common Mucosal Diseases in Children**

1.2003U2AN128 Zhao, female, 3 years old. High fever 4 days ago, fever subsided 2 days ago, but secondary mouth ulcers for 2 days, crying, prolongation, refusal to eat. Physical examination found that the child's gingiva was red and swollen all over the mouth, and clusters of pinhead-sized transparent blisters could be seen on the palate mucosa, some of which had broken into superficial ulcers and were widely distributed. The most likely diagnosis in this case is ( )

A. Hand-foot-mouth disease

B. Herpetiform aphthous ulcers

C. Herpetic gingivostomatitis

D. Thrush

E. Acute necrotizing gingivostomatitis

2.2003U2AN129 Zhao, female, 3 years old. High fever 4 days ago, fever subsided 2 days ago, but secondary mouth ulcers for 2 days, crying, prolongation, refusal to eat. Physical examination found that the child's gingiva was red and swollen throughout the mouth, and clusters of pinhead-sized transparent blisters could be seen on the palate mucosa, and some of them had broken into superficial ulcers and were extensive. The most likely diagnosis in this case is herpetic gingivostomatitis. The causative agent of infection in this case may be ( )

A. Coxsackievirus

B. Candida albicans

C. Streptococcus pneumoniae

D. Staphylococcus aureus

E. herpes simplex virus type I

3. 2003U2A2082 Male, 4 years old. Fever for 2 days with sore throat. Physical examination: pharyngeal congestion, 2 mm to 4 mm large and small blisters in the pharyngeal palatine arch, uvula, and soft palate. The most likely pathogens are ( )

A. Coxsackie group A virus

B. Respiratory syncytial virus

C. Adenovirus

D. Rhinovirus

E. Coronavirus

4.2003U2AN130 Zhao, female, 3 years old. High fever 4 days ago, fever subsided 2 days ago, but secondary mouth ulcers for 2 days, crying, prolongation, refusal to eat. Physical examination found that the child's gingiva was red and swollen throughout the mouth, and clusters of pinhead-sized transparent blisters could be seen on the palate mucosa, and some of them had broken into superficial ulcers and were extensive. The most likely diagnosis in this case is herpetic gingivostomatitis. The causative agent of this case may be herpes simplex virus type I. Which of the treatment measures in this case is not appropriate ( )

A. Take breaks to strengthen your nutrition

B. Systemic antibiotics

C. Supplement with plenty of vitamins

D. Traditional Chinese Medicine

E. Topical application of corticosteroids nebulized inhalation

5. 2002U2A1010 Contraindicated corticosteroids are ( )

A. Lichen planus

B. Discoid lupus erythematosus

C. Simple and herpes zoster

D. Recurrent canker sores

E. Erythema multiforme

6.2000U2A1001 The best site for infantile traumatic ulcer is ( )

A. Tongue margin

B. Buccal mucosa

C. Near the tongue-tie

D. Vestibular sulcus mucosa

E. Dorsum of tongue

· The most likely diagnosis is C. herpetic gingivostomatitis

· The causative agent of infection may be E. simplex type I virus

· The most likely causative agent is A. coxsackie's group A virus

· Inappropriate treatment is B. Systemic antibiotics

· The diseases in which corticosteroids are contraindicated are C. simplex and herpes zoster

· The best site for infantile traumatic ulcers is A. tongue

**Chapter 11 Bite Induction**

1. 2013U2A2074 6-year-old girl complains of crooked new teeth. Check: 2/3 of the two upper central incisors erupted, inclined distal to the middle, with a gap of 2mm in the middle. Which of the following may be the cause of the gap is wrong ( )

A. Lateral incisor germ squeezes the root of the central incisor to form a gap

B. The presence of extra teeth, odontomatas leading to gaps

C. The labial frenulum is attached too low to form a gap

D. Congenital absence of lateral incisors creates a gap in the central incisors

E. Finger sucking habits can cause scattered gaps in the anterior teeth

2. 2013U2A2075 The child was 3 years old, timid and shy. The anterior teeth are open and closed, and there are scattered gaps. The child has a bad habit of finger sucking. The best treatment at this stage is ( )

A. Do not do processing, observe

B. Finger application of medication to get rid of bad habits

C. Fixed appliance orthodontics

D. Adopt appropriate nursing and psychological counseling methods

E. Strict discipline and no bad habits

3.2000U2AN114 male, 7 years old. Pain in the right lower back tooth for 1 week and swollen face for 3 days. Examination: large caries of the first deciduous molar in the lower right, percussion pain (+++), shallow gingival and buccal groove, palpable fluctuation sensation, palpatory pain (+++), deep cavity on the mesial occlusal surface, percussion (-), temperature test as the control tooth. X-ray showed that IV. Root divergence and large area of low-density shadow, distal root resorption 2/3, permanent tooth embryo sac discontinuous, and the upper bone plate was blurred. Caries proximal medulla, bony hard plate continuous. The initial treatment is to remove the drainage, and after the initial treatment, ( ) should also be selected

A. Make a gap holder

B. Improve root canal treatment

C. Remove and make a gap holder

D. Observe the gap

E. Calcium hydroxide fills the root canal and temporarily performs its function

4.2006U2A1002 Premature loss of baby teeth causes gap changes, the following items are wrong ( )

A. Deciduous canines are lost early, and the gap is easy to become smaller

B. The younger the age, the faster the gap changes

C. Premature loss of deciduous incisors with less variation in the gap

D. Premature loss of primary molars, if the first permanent molars are erupting, the gap is likely to become smaller

E. Before the eruption of the first permanent molar, the first deciduous molars are lost prematurely, and the gap rarely changes

5.2002U2A1008 Gap maintainer can not be used in which of the following cases of premature loss of deciduous teeth ( )

A. Inherit the congenital loss of permanent teeth, and decide to close the gap based on the dentition situation

B. X-rays show that the alveolar bone covered by the inherited permanent tooth germ is thicker

C. If the first permanent molars have not erupted and the first primary molars are lost

D. If the first permanent molars have erupted and the first primary molars have been removed

E. Premature loss of deciduous caninees

6.2002U2A1009 Describe which is wrong about the factors affecting the change of the gap after premature loss of deciduous teeth ( )

A. Premature loss of primary incisors, less of which the gap becomes smaller or disappears

B. Premature loss of primary canines and the gap is very small

C. The younger the age at which baby teeth are lost, the more chances there are of adjacent teeth tilting

D. When a baby tooth is close to falling out, the more chance there is of tilting of the adjacent tooth

E. When the dentition is severely crowded, the teeth move quickly towards the missing tooth space

7.2014U2A1006 Whether to make a functional retainer after deciduous tooth extraction should be considered ( )

A. Whether the permanent tooth is congenitally missing

B. Thickness of the bone plate above the permanent tooth germ

C. Sequence of adjacent tooth eruption

D. Crowding of teeth

E. All of the above are true

8.2010U2A2102 The second deciduous molars are lost early, and the first permanent molars have not yet erupted or have not erupted enough.

A. Distal guide plate retainer

B. Loop retainer

C. Functional activity retainers

D. Tongue arch retainer

E. Gap Extension Device

9. 2003U2A2081 The child was 7 years old, 6 Meng 2/3, E was lost early. Which retainer should be chosen better ( )

A. Ring retainer with ring

B. Full crown neaiter retainer

C. Distal guide plate holder with ring

D. Full crown distal guide plate retainer

E. Movable functional retainers

10.2014U2A2081 Male, 9 years old. There is a hole in the left maxillary back tooth for 2 months to be filled. Oral examination: left upper V. residual crown, upper left V. irreparable. 6 have sprouted. Which gap holder should be put on after removal ( )

A. Distal guide plate retainer

B. Loop retainer

C. Functional activity retainers

D. Tongue arch retainer

E. Gap Extension Device

11.2014U2A2082 Lu, male, 5 years old, recurrent pain and swelling of the left upper back tooth for half a year, without systematic specialist treatment. Examination: left upper V. residual crown, no pain, percussion (+), no response to cold measurement, loose (++), buccal fistula discharge left upper 6 did not erupt. X-ray showed that about 1/3 of the upper left V. periroot shadow tooth root was absorbed, and the upper left 6 had erupted to the subgingival area, and about 2/3 of the tooth root was formed.

A. Root canal treatment + inlay repair

B. Root canal treatment + metal finished crown restoration

C. Tooth extraction + loop space retainer

D. Tooth extraction + distal guide gap retainer

E. Palliative care, medullary dressing

12.2014U2A2083 A 9-year-old male with multiple teeth in the upper jaw was damaged and lost and required treatment. Examination: poor oral hygiene, left and right upper IV. missing, V. stump, exposed root tip, no caries on left and right upper 6. Gap management is best done with ( )

A. Distal guide gap holder

B. Loop type gap holder

C. Fillable gap keeper

D. Nance palatal arch space retainer

E. Tongue arch space maintainer

13. 2006U2A1003 Functional activity maintainer indications for ( )

A. Premature loss of second deciduous molars. The first permanent molars are insufficient

B. Premature loss of first deciduous molars

C. Missing 2 or more deciduous molars or missing or accompanied by missing deciduous anterior teeth

D. Second deciduous molars or first permanent molars on both sides, and the inherited permanent teeth are about to erupt in the near future

E. Narrowing or disappearance of the gap due to caries or premature loss of baby teeth

14.2014U2A2084 Female, 10 years old. During the oral examination, the child found that the upper right IV. was missing, and the gap between the missing teeth had been significantly reduced.

A. Distal guide plate retainer

B. Loop retainer

C. Functional activity retainers

D. Tongue arch retainer

E. Gap Extension Device

15.2019U2B1108 A 4-year-old child with IV deficiency in the right lower breast and a gap maintainer was used after root canal therapy for breast V due to periapical disease.

A. Far guide plate clearance holder

B. Tongue arch space maintainer

C. Loop type gap holder with ring

D. Nance palatal arch space retainer

E. Detachable gap keeper

16.2019U2B1107 Male, nine and a half years old, bilateral breast IV and V loss, the space maintainer that can be used is ( )

A. Far guide plate clearance holder

B. Tongue arch space maintainer

C. Loop type gap holder with ring

D. Nance palatal arch space retainer

E. Detachable gap keeper

17.2018U2B1108 Premature loss of multiple primary molars on both sides of the mandible, the first choice when the first permanent molars erupt incompletely ( )

A. Far guide plate clearance holder

B. Tongue arch space maintainer

C. Loop type gap holder with ring

D. Nance palatal arch space retainer

E. Detachable gap keeper

18.2018U2B1107 Unilateral first deciduous molars are the first choice for premature loss ( )

A. Far guide plate clearance holder

B. Tongue arch space maintainer

C. Loop type gap holder with ring

D. Nance palatal arch space retainer

E. Detachable gap keeper

19.2017U2A1051 Causes of premature loss of baby teeth, the correct description is ( )

A. Severe caries, endodontic disease and periapical disease

B. Ectopic eruption of permanent teeth, premature resorption of primary tooth roots

C. Trauma

D. Congenital tooth loss

E. All of the above are correct

20.2017U2A1050 Common causes of premature loss of primary molars ( )

A. Trauma

B. Congenital absence

C. Severe periapical lesions

D. Localized pulpitis

E. Crowded dentition

· E. Finger sucking habits can cause scattered gaps in the anterior teeth

· D. Adopt appropriate nursing and psychological counseling methods

· B. Improve root canal treatment

· E. Before the eruption of the first permanent molar, the first deciduous molars are lost prematurely, and the gap rarely changes

· A. Inherit the congenital loss of permanent teeth, and decide to close the gap based on the dentition situation

· D. The more chance there is of tilt of the adjacent teeth when the baby tooth is close to being lost, the more likely it is that the adjacent teeth will tilt (this description seems to be wrong, as the removal of a baby tooth that is about to fall out usually does not increase the risk of the adjacent teeth being tilted).

· E. All of the above are true

· A. Distal guide plate retainer

· A. Ring retainer with ring

· A. Distal guide plate retainer

· C. Tooth extraction + loop space retainer

· B. Loop type gap holder

· C. Missing 2 or more deciduous molars or missing or accompanied by missing deciduous anterior teeth

· E. Gap Extension Device

· C. Loop type gap holder with ring

· D. Nance palatal arch space retainer

· D. Nance palatal arch space retainer

· C. Loop type gap holder with ring

· E. All of the above are correct

· C. Severe periapical lesions

**Chapter 12 Oral Surgery for Children**

1.2000U2AN113 male, 7 years old. Pain in the right lower back tooth for 1 week and swollen face for 3 days. Examination: large area of caries, percussion pain (+++), shallow gingival and buccal groove, palpable fluctuation, palpatory pain (+++), deep cavity on the mesial occlusal surface, percussion (-), temperature test as control tooth. X-ray showed that IV. Root divergence and large area of low-density shadow, distal root resorption 2/3, permanent tooth embryo sac discontinuous, and the upper bone plate was blurred. Caries proximal medulla, bony hard plate continuous. The initial treatment is ( )

A. Removal after medication to control inflammation

B. Open the marrow and take anti-inflammatory drugs orally

C. Root canal open, oral anti-inflammatory drugs

D. The root canal is open and the abscess is incised

E. Abscess incision

2.2010U2A1009 Which baby tooth should not be extracted ( )

A. Baby teeth whose crowns are so badly damaged that they cannot be repaired

B. Periapical inflammation has been involved in inheriting the germ of the permanent tooth

C. Baby teeth have pulp inflammation that can be treated, but the time for replacement is very close

D. Pushed by the eruption force of the inherited permanent teeth, the root tip is exposed beyond the gingiva, often causing local mucosal traumatic ulcers

E. There are signs of infection but can be completely cured

3.2009U2A1010 Contraindications for deciduous tooth extraction ( )

A. Blood disorders

B. Endocrine diseases

C. Severe cardiac insufficiency

D. Acute inflammation around the apex

E. All of the above

· The initial treatment is D. root canal opening and abscess incision

· Baby teeth that should not be extracted are E. have signs of infection but can be completely cured

· Contraindications to the extraction of deciduous teeth are E. all of the above

**Preventive Oral Medicine**

**Chapter 1 Introduction**

1.2005U4A1002 The establishment of the National Dental Disease Prevention and Treatment Steering Group in China was established in ( )

A. 1984

B. 1986

C. 1988

D. 1990

E. 1992

2. 1999U4A1006 The time when the development of preventive oral medicine began was ( )

A. End of the 18th century

B. Mid-20th century

C. Early 19th century

D. Late 19th century

E. Early 20th century

3. 2003U4A1005 Primary prevention measures for oral cancer are ( )

A. Early detection

B. Early diagnosis

C. Early treatment

D. Etiological prophylaxis

E. Prevention of recurrence

4.2017U4A1002 Which of the following is NOT an essential element of preventive oral medicine research ( )

A. Prevalence of oral diseases in the population

B. Crowd prevention measures

C. Individual prevention methods

Regional epidemiological status

E. Personal health care approach

5.2017U4A1003 The following are not included in the primary prevention of preventive dentistry.

A. Oral health education

B. Oral Hygiene Instructions

C. Plaque control

D. Fixed Repairs

E. Elimination of causative factors

These questions look like exam questions in dentistry or oral epidemiology. Let's answer these questions:

1. The National Steering Group for the Prevention and Treatment of Dental Diseases was established in 1984. **Answer: A. 1984**
2. Preventive dentistry began to develop in the mid-20th century. **Answer: B. mid-20th century**
3. The primary prevention of oral cancer is the prevention of the cause. **Answer: D. Etiological prevention**
4. Which of the following is NOT an essential element of preventive oral medicine research is a personal health care approach. **Answer: E. Personal health care approach**
5. The following are not considered primary prevention in preventive dentistry, and fixed restorations are the following. **Answer: D. Fixed fix**

**Chapter 2 Oral Epidemiology**

1. 2014U4A1001 Cross-sectional survey of one of the epidemiological research methods is also called ( )

A. Case-control study

B. Status Survey

C. Field trials

D. Cohort studies

E. Routine data analysis

2.1999U4A1001 Oral health surveys in the community are not conducted for the purpose of ( )

A. Obtain baseline information on oral health

B. Comparisons can be made in future evaluations of prevention projects

C. Understand the oral disease status of the population

The need to design prevention projects

E. The need for computerized statistics

3. 2014U4A1002 One of the oral health survey methods is ( )

A. Access to Surveys

B. Random Surveys

C. Sample surveys

D. Fixed-point surveys

E. Household surveys

4.2004U4A1001 Children's tooth brushing should be used ( )

A. Horizontal brushing method

B. Vertical brushing method

C. Circular brushing

D. Brushing method

E. Horizontal fibrillation method

5.1999U4A1002 Caries data collation is generally not grouped by which item ( )

A. Age

B. Educational level

C. Gender

Urban and rural

E. Nations

6.2003U4A1003 In order to compare the prevalence of dental caries in different countries and regions, WH0 defines the level of dental caries prevalence as measured by what age children have dental caries ( )

A. 5~8 years old

B. 12 years old

C. 12~15 years old

D. 15 years old

E. 25 years old

7.2006U4A1054 The peak age group of caries in deciduous teeth is ( )

A. 2~3 years old

B. 3~4 years old

C. 5~8 years old

D. 6~9 years old

E. 7~10 years old

8.2014U4A1003 Population distribution factors influencing the prevalence of caries are not included ( )

A. Age

B. Diet

C. Gender

Urban and rural

E. Nations

9. 2009U4A1001 Factors influencing the prevalence of caries do not include (). ( )

A. Sugar intake

B. Frequency of sugar intake

C. Calcium intake

D. Fluoride intake

E. Eating habits

10.2003U4A1004 Factors affecting patients with caries do not include ( )

A. Eating habits

B. Regional differences

C. Gender and ethnic differences in the population

Climatic conditions

E. Age differences

11.2014U4A2079 There are 230 children aged 5~6 years old in a community, according to the 2000 oral health target set by the WHO, the caries-free children in this age group have reached at least ( )

A. 92

B. 138 persons

C. 115 persons

D. 69 persons

E. 161 persons

12.1999U4A1012 The similarity between the plaque index and the scale index is ( )

A. Only the thickness of the plaque at the gingival margin is considered

B. The area of plaque on the tooth surface is not estimated

C. Rinse your mouth before the examination

D. Inspect the tooth surface after blow-drying

Use a sickle probe

13.2010U4A1001 Among the following indices, the following indices are used to judge oral hygiene status ( )

A. Gingival index

B. Plaque index

C. Gingival sulcus bleeding index

D. Bleeding on probing

Periodontal index

14.2010U4A1002 The index recommended by the World Health Organization for epidemiological investigation of periodontal disease is ( )

A. SBI

B. PD

C. CPITN

D. AL

E. PLI

15.2005U4A1001 Oral epidemiology is not ( )

A. A branch of epidemiology

B. To study the population distribution of oral diseases

C. Fundamentals of Preventive Dentistry

D. Foundations of Oral Molecular Biology

E. Study the factors that influence the distribution of the disease

16.2013U4AN130 Among the 300 students in the sixth grade (12-year-old age group) of a school, 180 of them had caries, lost or filled permanent teeth, 328 untreated caries, 3 teeth lost due to caries, and 69 filled teeth due to caries. According to the WHO evaluation criteria, the prevalence of caries in this group belongs to ( )

A. Very low

B. Low

C. Medium

D. High

E. Very high

17.2013U4AN131 Among the 300 students in the sixth grade (12-year-old age group) of a school, 180 of them had caries, lost or filled permanent teeth, 328 untreated caries, 3 lost teeth due to caries, and 69 filled teeth due to caries. If students in this grade were randomly divided into two groups, one group rinsed with fluoride water and the other group with tap water, and the effect of fluoride water gargle was observed, the study was called ( )

A. Routine data analysis

B. Cross-sectional surveys

C. Case-control studies

D. Cohort studies

E. Epidemiological experiments

18. The similarity between the plaque index and the scale index is ( )

A. Only the thickness of the plaque at the gingival margin is considered

B. The area of plaque on the tooth surface is not estimated

C. Rinse your mouth before the examination

D. Inspect the tooth surface after blow-drying

Use a sickle probe

19.2019U4A2084 The health administrative department of a city plans to conduct an oral health survey on the elderly over 65 years old in the city, and formulate a future oral disease control plan based on the survey results. After the standard conformance test, the Kappa value of a certain inspector is 0.61, indicating that the reliability is ( )

A. Unreliable

B. Inconformity

C. Medium

D. Excellent

E. Completely reliable

20.2018U4A1015 Equidistant sampling or mechanical sampling method is also known as ( )

A. Simple sampling

B. Systematic sampling

C. Stratified sampling

D. Cluster sampling

E. Multi-stage sampling

21.2018U4A1016 The main source of fluoride in the human body is ( )

A. Air

B. Drinking water

C. Plant-based foods

D. Animal foods

Topical application of fluoride

22.2018U4A1014 Periodontal condition is commonly described in the following indices ( )

A. DMFT

B. dmfs

C. CPI

D. Dean Index

E. DNFS

23.2019U4A1013 The description of the oral epidemiological role, which is incorrect ( )

A. Research and evaluation of disease prevention measures

B. Monitoring epidemiological trends in oral diseases

C. Understand the distribution of oral health and disease in the population

D. Provide guarantees for the implementation of oral health promotion

E. Study the etiology of oral diseases

24.2020U1A1003 Gingival Index score of 2 Yes ( )

A. Advanced periodontal disease, periodontal pocket depth of 6mm or more

B. Moderate inflammation of the gums, red color, bright edema, bleeding on probing

C. The gums have color changes, significant swelling, bleeding on probing or bleeding automatically

D. Early periodontal disease, the depth of the periodontal pocket is 4~5mm

E. Mild inflammation of the gums, mild changes in gum color and mild edema

Chapter 2 Oral Epidemiology

1. A cross-sectional survey is also known as a current survey. **Answer: B. Current Survey**
2. Oral health surveys in the community are not conducted for the purpose of computer statistics. **Answer: E. The need for computer statistics**
3. One of the methods of oral health surveys is sample surveys. **Answer: C. Sample survey**
4. Children should brush their teeth in a circular arc. **Answer: C. Circular brushing**
5. The compilation of caries data is generally not grouped by ethnic group. **Answer: E. Ethnicity**
6. WH0 defines the level of caries prevalence as mean caries in children aged 12 years. **Answer: B. 12 years old**
7. The peak age of caries in deciduous teeth is 3~4 years old. **Answer: B. 3~4 years old**
8. Ethnicity is not included in the population distribution factors influencing the prevalence of caries. **Answer: E. Ethnicity**
9. Factors influencing the prevalence of caries do not include fluoride intake. **Answer: D. Intake of fluoride**
10. Factors affecting patients with caries do not include gender and ethnic differences in the population. **Answer: C. Gender and ethnic differences in the population**
11. According to the 2000 oral health target set by the WHO, there are at least 92 caries-free children aged 5~6 years. **Answer: A. 92 people**
12. The plaque index is the same as the scale index after blow-drying. **Answer: D. Inspect after blow-drying the tooth face**
13. The plaque index is used to determine the state of oral hygiene. **Answer: B. Plaque index**
14. The index recommended by the World Health Organization for epidemiological investigations of periodontal disease is CPITN. **Answer: C. CPITN**
15. Oral epidemiology is not the basis of oral molecular biology. **Answer: D. Fundamentals of Oral Molecular Biology**
16. The prevalence of caries in this group is high. **Answer: D. High**

17. Studies that observe the effects of fluoride gargles are called cluster studies. **Answer: D. Cohort study**

18. The similarity between the plaque index and the scale index is the inspection after blow-drying. **Answer: D. Inspect after blow-drying the tooth face**

19. A Kappa value of 0.61 for one examiner indicates that reliability is excellent. **Answer: D. Excellent**
20. Equidistant sampling or mechanical sampling is also known as systematic sampling. **Answer: B. Systematic sampling**
21. The main source of fluoride in the human body is drinking water. **Answer: B. Drink water**
22. The index commonly used to describe periodontal condition is the CPI. **Answer: C. CPI**
23. Regarding the description of the epidemiological role of the oral cavity, it is incorrect to study the etiology of oral diseases. **Answer: E. Study the causes of oral diseases**
24. The gingival index score of 2 is moderate inflammation of the gums, red color, bright edema, and bleeding on probing. **Answer: B. Moderate inflammation of the gums, red color, bright edema, bleeding on probing**

**Chapter 4 Prevention of caries**

1.2014U4A1012 Primary prevention of caries includes ( )

A. Root canal treatment, health education

B. Early diagnosis and early treatment

C. Pit and fissure sealing, fluoride caries prevention

D. Health education, filling therapy

E. Early diagnosis, fluoride caries prevention

2.2002U4A1002 Principles for the prevention and treatment of caries in deciduous teeth and young permanent teeth include ( )

A. Analyze the causes

B. Treatment of active caries

C. Oral health education

D. Early detection, early diagnosis, and early treatment

E. A+B+C+D

3. 2013U4AN122 25-year-old patient, poor oral hygiene, left lower first permanent molar, pulp-periodontal lesions, severe pain, but no loosening of the affected tooth. In order to eliminate the pain and restore health, the first measure to be taken is to open the marrow and drain it to stop the inflammation from spreading. The above treatment measures fall under ( )

A. Oral health education

B. Special protective measures

C. Primary prevention

Secondary prevention

E. Tertiary prevention

4. 2013U4AN123 A 25-year-old patient with poor oral hygiene and a left lower first permanent molar had pulp-periodontal joint lesions, severe pain, but no loosening of the affected tooth. In order to eliminate the pain and restore health, the first measure taken is to open the marrow and drain the marrow to stop the inflammation from spreading. The above treatment measures are tertiary prevention. Periodontal treatment of the lower left first permanent molar should also be performed. In addition, patients should also be instructed to do a good job of oral health care, in addition to ( )

A. Learn about oral health

B. Practice good hygiene

C. Rational use of fluoride

D. Regular oral check-ups

E. Frequent self-examination for caries activity

5.2014U4A1013 The main function is to agglutinate cariogenic bacteria, reduce plaque formation, release adhered plaque, and prevent oral pH from dropping

A. Safflower

B. Tea polyphenols

C. Chitin

D. Chlorhexidine

E. Enzymes

6.2014U4A1014 Which of the following is not a characteristic of the drug that controls plaque ( )

A. It can kill specific pathogenic bacteria

B. Unaffected by other components in the oral cavity and plaque

C. Antimicrobial resistance that is not easy to cause bacteria

D. No toxic side effects on the oral cavity and the whole body

E. Can stay in the mouth for a long time

7.2010U4A1003 The appropriate intake of sucrose for children aged 5~9 years old is ( )

A. 15～20g

B. 20～25g

C. 25～30g

D. 30～45g

E. 50～65g

8.2016U4B1100 The caries activity test used to detect the acid-producing ability of acid-producing bacteria in plaque on the tooth surface is ( )

A. Dentobuff Strip Trial

B. Cariostat test

C. Dentocult LB trial

D. Dentocult SM trial

E. Resazurin paper method

9.2016U4B1101 To understand the caries activity test of salivary buffering capacity is ( )

A. Dentobuff Strip Trial

B. Cariostat test

C. Dentocult LB trial

D. Dentocult SM trial

E. Resazurin paper method

10.2019U4A2086 50 15-year-old students in a certain class, including 10 patients with caries, the number of caries fillings was: D=70, M=2, F=8. The number of caries filling surfaces was D=210, M=10, F=15, and the caries surfaces of the students in this class were all ( )

A. 0.8

B. 1.4

C. 1.6

D. 4.2

E. 4.7

11.2018U4A1017 The most suitable age for pit and fissure sealing of primary molars is ( )

A. 2~3 years old

B. 3~4 years old

C. 5~6 years old

D. 7~8 years old

E. 9~10 years old

12.2018U4A1019 Among the following options, which belong to oral primary prevention, is ( )

A. Denture restoration

B. Filling therapy

C. Pit and fissure sealing

D. Deep periodontal scraping

E. Orthodontic treatment

13.2018U4A1020 The key links in the prevention of caries are ( )

A. Plaque control

B. Control sugar intake

C. Application of fluoride

D. Enhance the body's immunity

E. Enhance the caries resistance of teeth

14.2019U4A1014 In general, the frequency of fluorine coating application is ( )

A. 1 time/year

B. 2 times/year

C. 3 times/year

D. 4 times/year

E. 5 times/year

15.2019U4A2085 The average caries of 12-year-old children in China is 6.8, and the prevalence of caries in the WHO assessment is ( )

A. Very low

B. Low

C. Medium

D. High

E. Very high

16.2018U4A1018 The appropriate and safe intake of fluoride per person (mg/d) for children aged 1~3 years is ( )

A. 0.1～0.4

B. 0.5～1.5

C. 1.6～2.6

D. 2.7～3.7

E. 3.8～4.8

17.2017U4A1005 For the prevention of dental caries, pit and fissure sealing in childhood can play a very good role. Regarding the age limit for pit and fissure sealing, the following statement is correct ( )

A. It can be performed within 4 years after tooth eruption

B. Deciduous molars are generally performed around 5~6 years old

C. The first permanent molars are performed at around 8 years of age

D. Second permanent molars are performed around 10 years of age

E. Deciduous molars are performed around 7 years of age

18.2017U4A1004 Regarding the operation of pit and fissure sealing in the prevention of caries in children, the following statement is wrong ( )

A. The time of permanent tooth etching is 20~30 seconds

B. The time for acid etching of deciduous teeth is 60 seconds

C. Dry tooth surface with compressed air for 10~15 seconds

D. Self-coagulating sealant cures 1~2 minutes after coating

E. The irradiation time of the light-curing sealant is generally 60 seconds

19.2020U1A1054 Cariostat test detects that the color of the culture medium is yellow, indicating ( )

A. Good oral hygiene

B. Low caries activity

C. Moderate caries activity

D. Dangerous caries activity

E. Significant caries activity

20.2020U1A1055 The primary prevention of caries is ( )

A. Pit and fissure sealing

B. X-ray examination

C. Removable denture restoration

D. Fixed denture restoration

E. Timely filling of early caries

21.2020U1A1056 Fixed denture restoration belongs to caries ( )

A. Zero-level prevention

Primary prevention

C. Secondary prevention

D. Tertiary prevention

E. Not classified as tertiary prevention of caries

Chapter 4 Prevention of caries

1. Primary prevention of caries includes health education and fluoride to prevent caries. **Answer: E. Early diagnosis, fluoride caries prevention**
2. The principles of prevention and treatment of caries in deciduous teeth and young permanent teeth include analysis of causes, treatment of active caries, oral hygiene education, early detection, early diagnosis, and early treatment. **Answer: E. A+B+C+D**
3. Treatment in patients presenting at the age of 25 is secondary prevention. **Answer: D. Secondary prevention**
4. Patients should also be instructed to take good oral health care in addition to frequent self-examination for caries activity. **Answer: E. Frequent self-examination for caries activity**
5. The main effect is to agglutinate cariogenic bacteria, reduce plaque formation, release adhered plaque, and prevent oral pH from dropping the substance is tea polyphenols. **Answer: B. Tea polyphenols**
6. Which of the following is not a plaque control drug, which is characterized by its ability to stay in the mouth for a long time. **Answer: E. It can stay in the mouth for a long time to function**
7. The appropriate intake of sucrose for children aged 5~9 years is 15~20g. **Answer: A. 15~20g**
8. The caries activity test used to detect the acid-producing ability of acid-producing bacteria in plaque on the tooth surface is the Dentocult SM test. **Answer: D. Dentocult SM trial**
9. The caries activity test to understand the capacity of saliva buffering is the Dentobuff Strip test. **Answer: A. Dentobuff Strip trial**
10. There were 50 15-year-old students in a class, and all of them had 4.2 caries. **Answer: D. 4.2**
11. · C. 5~6 years old
12. · C. Pit and fissure sealing
13. · A. Plaque control
14. · B. 2 times/year
15. · B. Low
16. · B. 0.5～1.5
17. · C. The first permanent molars are performed at around 8 years of age
18. · B. The time for acid etching of deciduous teeth is 60 seconds
19. · C. Moderate caries activity
20. · A. Pit and fissure sealing
21. · C. Secondary prevention

**Chapter 5 Fluoride and caries prevention**

1.1999U4A1005 Which of the following fluoride is not used in fluoride toothpaste ( )

A. Calcium fluoride

B. Sodium fluoride

C. Stannous fluoride

D. Amine fluoride

E. Sodium monofluorophosphate

2.2000U4A1004 Children's fluoride water gargle to prevent caries, the correct way to use it is ( )

A. Over 6 years old, rinse with 15ml for 1 minute

B. Over 6 years old, rinse with 10ml for 1 minute each time

C. Over 6 years old, rinse with 10ml for 2 minutes each time

D. 6 years old and younger, gargle with 10ml for 1 minute each time

E. 6 years of age and younger, gargle with 1Oml for 2 minutes each time

3.2020U1A1061 The number of bacteria in saliva that can be adsorbed to the tooth surface can be reduced is ( )

A. Chlorhexidine

B. Phenolic compounds

C. Quaternary ammonium compounds

D. Stannous fluoride

E. Trichlorohydroxyphenyl ether

4. 2020U1A1062 The endotoxin that can remove plaque is ( )

A. Chlorhexidine

B. Phenolic compounds

C. Quaternary ammonium compounds

D. Stannous fluoride

E. Trichlorohydroxyphenyl ether

5.2020U1A1057 The following statement is correct ( )

A. Children aged 3~6 years old should use a "pea"-sized fluoride toothpaste each time under the supervision and guidance of their parents.

B. In areas with high fluoride, fluoride toothpaste is not recommended for children under 12 years of age.

C. For children over 6 years old, brushing teeth twice a day with a toothpaste with a fluoride concentration higher than 1500mg/kg can effectively prevent caries.

D. Excessive swallowing of fluoride toothpaste in children generally does not pose a risk of dental fluorosis.

E. Adults use about 2g of fluoride toothpaste each time.

· A. Calcium fluoride

· B. Over 6 years old, rinse with 10ml for 1 minute each time

· A. Chlorhexidine

· A. Chlorhexidine

· A. Children aged 3~6 years old should use a "pea"-sized fluoride toothpaste each time under the supervision and guidance of their parents.

**Chapter 6 Clinical Oral Preventive Techniques**

1.2000U4A1002 Preventive resin filling without one of the following operations ( )

A. Removal of diseased tooth enamel or dentin from pits and fissures

B. Adopt a preventive extension of the hole preparation method

C. Acid etching technology is adopted

D. Fill with resin material

E. Apply a layer of sealant to the occlusal surface

2.2014U4A1004 The most effective prevention method for punctate pit and fissure caries is ( )

A. Educate parents about pits and fissures

B. Regular check-ups, early detection and early treatment

C. Control your diet and brush your teeth before bedtime

D. Use fluoride, fluoride toothpaste, and fluoride water to rinse your mouth

E. Punctate pit and fissure sealant

3.2000U4A1003 The most suitable age for pit and fissure sealing of primary molars is ( )

A. 1~2 years old

B. 3~4 years old

C. 5~6 years old

D. 7~8 years old

E. 9~10 years old

4.2014U4A1005 The correct view about pit and fissure sealing is ( )

A. Baby molars do not do pit and fissure sealing

B. Pit and fissure closure of the first permanent molar

C. Pit and fissure sealant can be done as long as there are pit and fissure

D. Adults do not need to have pit and fissure sealants

E. Anterior teeth with malformed tongue tip do not require pit and fissure sealing

5.2010U4B1136 N-N dihydroxyethyl-p-toluidine is ( )

A. Fissure sealant diluent

B. Pit and fissure sealants, visible light-curing initiators

C. Etching agent prepared with pit and fissure sealant

D. Pit and fissure sealants, self-coagulation and curing initiators

E. Pit and fissure sealant resin matrix

6.2010U4B1137 Bisphenol A-glycidyl methacrylate is ( )

A. Fissure sealant diluent

B. Pit and fissure sealants, visible light-curing initiators

C. Etching agent prepared with pit and fissure sealant

D. Pit and fissure sealants, self-coagulation and curing initiators

E. Pit and fissure sealant resin matrix

7.2010U4B1138 Camphor ester is ( )

A. Fissure sealant diluent

B. Pit and fissure sealants, visible light-curing initiators

C. Etching agent prepared with pit and fissure sealant

D. Pit and fissure sealants, self-coagulation and curing initiators

E. Pit and fissure sealant resin matrix

8.2010U4B1139 Methacrylate is ( )

A. Fissure sealant diluent

B. Pit and fissure sealants, visible light-curing initiators

C. Etching agent prepared with pit and fissure sealant

D. Pit and fissure sealants, self-coagulation and curing initiators

E. Pit and fissure sealant resin matrix

· E. Apply a layer of sealant to the occlusal surface

· E. Punctate pit and fissure sealant

· C. 5~6 years old

· C. Pit and fissure sealant can be done as long as there are pit and fissure

· B. Pit and fissure sealants, visible light-curing initiators

· E. Pit and fissure sealant resin matrix

· B. Pit and fissure sealants, visible light-curing initiators

· E. Pit and fissure sealant resin matrix

**Chapter 7 Prevention of Periodontal Disease**

1.2009U4A1002 Commonly used plaque stain is ( )

A. Gentian violet

B. Iodine tincture

C. Neutral red

D. Tetraiodofluorescein sodium

E. C+D

2.2014U4A1006 Which drug is not suitable for long-term use as a method to control plaque and prevent periodontal disease ( )

A. Chlorhexidine

B. Antibiotics

C. Sanguinarine

D. Phenolic compounds

E. Quaternary ammonium compounds

3. The main role of 2014U4A1007 quaternary ammonium compound in plaque control is ( )

A. Interfere with plaque attachment

B. Disruption of plaque matrix

C. Removal of plaque endotoxins

D. Altering the permeability of bacterial cell membranes

E. Control plaque reproduction

4.2016U4A1012 Chlorhexidine antibacterial plaque main effect bacteria ( )

A. Nucleus

B. Cytoplasm

C. Cell membrane

D. Cytoplasm

E. Cell wall

5.2019U4A1025 Adult toothbrush handle length is ( )

A. 120～130mm

B. 140～150mm

C. 155～160mm

D. 160～180mm

E. 180～200mm

6.2019U4A1018 Brushing methods that are easy to learn and master for young children are ( )

A. BASS BRUSHING METHOD

B. Horizontal fluttering brushing method

C. Fones brushing method

D. Smith brushing method

E. Modified Smith brushing method

7.2019U4A1017 The following are not toothpaste ingredients ( )

A. Friction agent

B. Wetting agents

C. Coagulants

D. Adhesives

E. Preservatives

8.2019U4A1016 When chlorhexidine and stannous fluoride are used in combination for plaque control, the correct way to use it is ( )

A. 15-20 minutes after chlorhexidine is applied before stannous fluoride

B. Use stannous fluoride 90 minutes after chlorhexidine application

C. Use stannous fluoride 5 minutes after application of chlorhexidine

D. 6-10 minutes after chlorhexidine is applied before stannous fluoride

E. 30-60 minutes after application of chlorhexidine followed by stannous fluoride

9.2019U4A1015 Simple brushing can remove plaque on average ( )

A. About 40 per cent

B. About 50%.

C. About 60%.

D. About 70%.

E. About 80 per cent

10.2018U4A1021 2010 Global Oral Health Goals: The rate of edentulism at 35~44 years old is ( )

A. Not more than 2 per cent

B. Not more than 4 per cent

C. Not more than 8 per cent

D. Not more than 10 per cent

E. Not more than 12 per cent

11.2020U1A1060 Belonging to the tertiary prevention of periodontal disease is ( )

A. Oral health education and mentoring

B. Oral health education

C. Supragingival cleansing

D. X-rays

Periodontal surgery

· D. Tetraiodofluorescein sodium

· B. Antibiotics

· D. Altering the permeability of bacterial cell membranes

· C. Cell membrane

· B. 140～150mm

· C. Fones brushing method

· A. Friction agent

· A. 15-20 minutes after chlorhexidine is applied before stannous fluoride

· B. About 50%.

· B. Not more than 4 per cent

· Periodontal surgery

**Chapter 8 Self-Oral Health Care Methods**

1.2003U4A1001 The advantages of nylon wire bristles do not include which item ( )

A. The hair ends are rounded, which has a good cleaning effect on teeth and massage on teeth

B. Good elasticity

C. Abrasion resistance

D. Poor water absorption

E. Not easy to curl at high temperatures

2. 2003U4A1002 Aromatics in toothpaste are ( )

A. Peppermint

B. Glycerol

C. Benzoates

D. Silica

E. Sodium lauryl sulfate

3.2002U4A1001 Which brushing method is best ( )

A. Brush lightly

B. Rebrush

C. Brush up and down

D. Brush up and down

E. Horizontal brushing

4.2000U4A1001 Cannot be effectively achieved by brushing teeth ( )

A. Remove tartar and plaque from the neck of the teeth

B. Remove tartar and plaque from the gingival sulcus

C. Remove food debris from the occlusal surface

D. Remove tartar and plaque from the adjacent surfaces of the teeth

E. Massage the gums to enhance the resistance of periodontal tissues

5. 2016U4B1102 Sodium laurate sulfate in toothpaste is ( )

A. Adhesives

B. Wetting agents

C. Preservatives

D. Friction agents

E. Detergents

6.2016U4B1103 Silica in toothpaste is ( )

A. Adhesives

B. Wetting agents

C. Preservatives

D. Friction agents

E. Detergents

· E. Not easy to curl at high temperatures

· A. Peppermint

· C. Brush up and down

· B. Remove tartar and plaque from the gingival sulcus

· E. Detergents

· E. Detergents

**Chapter 9 Prevention of Other Oral Diseases**

1.2016U4A1011 The daily smoking volume with a risk of oral cancer of 6.0 is ( )

A. 10~19 sticks

B. 40~49 pcs

C. 1~9 pcs

D. 20~29 pcs

E. 30~39 pcs

2. 2019U4A1019 Optical radiation is the main cause of skin cancer, and the wavelength is more common ( )

A. 200～300nm

B. 320～400nm

C. 320～450nm

D. 350～450nm

E. 350～550nm

3.2019U4A1020 The following epidemiological characteristics of oral cancer, the correct one is ( )

A. The South-East Asia region has the highest prevalence

B. Onset is not temporal

C. There is a tendency to have a lower age of illness

D. The male-to-female ratio is close to 1:1

E. Incidence is similar across ethnicities

4.2019U4A1021 Prevention methods for dental etching are ( )

A. Eat more fruits

B. Chewing acidic medications

C. Eat acidic foods

D. Refusal of chewing gum

E. Reduce the consumption of carbonated beverages

5. 2020U1A1064 is not part of the category of raising public awareness of oral cancer warning signs ( )

A. There are sores in the mouth that have not healed for more than 2 weeks

B. White patches in the mouth

C. Unexplained swelling and swollen lymph nodes in the mouth and neck

D. Unexplained recurrent bleeding in the mouth

E. Odor in the mouth

6.2020U1A1071 Actively prevent malocclusion ( )

A. Infants

B. Young children

C. School-age children

D. Young adults

E. Older persons

· C. 1~9 pcs

· B. 320～400nm

· C. There is a tendency to have a lower age of illness

· E. Reduce the consumption of carbonated beverages

· E. Odor in the mouth

· C. School-age children

**Chapter 10 Oral Health Care for Specific Populations**

1.2019U4B1111 The suitable population for pit and fissure sealing of the first permanent molars is ( )

A. Infants

B. Young children

C. Older persons

D. Middle-aged

E. Preschool

2. 2019U4B1112 The population susceptible to root caries is ( )

A. Infants

B. Young children

C. Older persons

D. Middle-aged

E. Preschool

3.2019U4B1113 The first oral examination should be done in ( )

A. Infants

B. Young children

C. Older persons

D. Middle-aged

E. Preschool

4.2020U1A1065 People exposed to acidic working environments are susceptible ( )

A. Acid erosion

B. Caries disease

C. Osteoporosis

D. Periodontal disease

E. Dental fluorosis

5.2020U1A1070 Before going to bed, the mother's fingers should be wrapped with clean gauze or the gums and palate should be scrubbed with latex finger sleeves ( )

A. Infants

B. Young children

C. School-age children

D. Young adults

E. Older persons

6.2020U1A1072 THE ROLE OF THE MOUTHGUARD ( )

A. Protect the teeth and other tissues in the mouth

B. Prevent jaw fractures

C. Reduce the likelihood of concussion

D. Enhance athletes' sense of safety

E. All of the above

7.2020U1A1075 The appropriate period for the treatment of oral diseases in pregnant women is ( )

A. 1~3 months

B. 3~6 months

C. 4~6 months

D. 5~7 months

E. 3~5 months

· E. Preschool

· C. Older persons

· E. Preschool

· A. Acid erosion

· A. Infants

· E. All of the above

· C. 4~6 months

**Chapter 11 Oral Health Promotion**

1.2013U4A1001 The decisive role in oral health promotion is ( )

A. Public

B. Dental Defense Guidance Group

C. Health administrative leadership

D. Dental health workers

E. Faculty of Stomatology

2.2014U4A1011 The methods of oral health education are generally ( )

A. Individual conversations, online exchanges, mass communication channels, organization of community events

B. Individual conversations, small seminars, mass communication channels, organization of community events

C. Individual conversations, small seminars, school lessons, and community activities

D. Expert consultations, mini-seminars, mass communication channels, organization of community events

E. Expert consultation, networking, mass communication channels, organization of community events

3.2020U1A1073 The core components of oral health promotion are ( )

A. Oral health education

B. Oral Health Protection

C. Oral disease prevention

D. Primary prevention of oral diseases

E. Community oral health care

4.2020U1A1074 Oral health education methods with large coverage and quick ability to attract public attention and focus on oral health problems to be solved are ( )

Mass media

B. Community activities

C. Mini-seminars

D. Individual conversations

E. Expert seminars

· D. Dental health workers

· B. Individual conversations, small seminars, mass communication channels, organization of community events

· A. Oral health education

· Mass media

**Chapter 14 Evidence-Based Dentistry and Its Applications**

1.2014U4A1008 Vinyl gloves are often used under what circumstances ( )

A. Surgery

B. Inspection

C. Hand allergies

D. Cleaning the instrument

E. Teeth cleaning

2.1999U4A1004 Mobile phone disinfection can not use which of the following methods ( )

A. Formaldehyde vapor fumigation

B. Autoclaving

C. Ultraviolet disinfection

D. Wipe with 2% iodine tincture and then wipe with 75% alcohol

E. Soak in 0.1% Xinjieer solution

3.2014U4A1009 The ideal immersion solution for pre-cleaning is ( )

A. 1:16 glutaroxonol solution

B. 8%~10% sodium hypochlorite solution

C. 1:213 iodophor

D. 75% ethanol

E. 1:32 synthetic phenol solution

4.2014U4A1010 The first step of instrument pre-cleaning is ( )

A. Rinse

B. Chemical immersion

C. Wipe

D. Drying

E. Docking

5. The time required to kill HBV at 95°C at 2001U4B1099 is ( )

A. 1 minute

B. 2 minutes

C. 3 min

D. 4 minutes

E. 5 minutes

6. 2001U4A1001 The diameter of the droplet when propagating is ( )

A. >2μm

B. >5μm

C. >10μm

D. >15μm

E. >20μm

7. 2001U4B1100 HBV is a heat-resistant virus that takes several minutes to kill at 95°C ( )

A. 1 minute

B. 2 minutes

C. 5 min

D. 10 minutes

E. 15 minutes

8.2019U4A1024 In oral health care, the main microorganisms transmitted by contact are ( )

A. Hepatitis C virus

B. Chickenpox virus

C. Measles virus

D. Rubella virus

E. Mumps virus

9.2019U4A1023 Diseases that are mainly transmitted by air are ( )

A. Adenovirus

B. AIDS

C. Syphilis

D. Viral hepatitis

E. Herpes

10.2019U4A1022 Gingival drain belongs to ( )

A. High-Risk Devices

B. Intermediate-risk devices

C. Low-risk devices

D. Medium-level sterilized instruments

E. High-level sterilization of instruments

11.2018U4A1022 Diseases transmitted mainly by contact are ( )

A. Herpes

B. Tuberculosis

C. Measles

D. Chickenpox

E. Rubella

· C. Hand allergies

· A. Formaldehyde vapor fumigation

· A. 1:16 glutaroxonol solution

· A. Rinse

· B. 2 minutes

· A. > 2 μm

· C. 5 min

· A. Hepatitis C virus

A. Adenovirus

A. High-Risk Devices

A. Herpes

## 1.2 Querying GPT-4 with Chain of Thought (Multiple-Choice Questions)

Prosthodontics

Restoration of Tooth Defects

1.2010U4A1059 gold porcelain crown can be used for ( )

A. Anterior teeth misaligned

B. Conical teeth

C. Fixed bridge retain

D. Tetracycline teeth

E. All of the above

2.2004U4A2082 A patient, the upper right 4 near the mid-cheek large area of mercury filling, dead pulp tooth, the patient has high aesthetic requirements, the best restoration design is ( )

A. Gold alloy full crown

B. Resin inlays

C. Metal porcelain crown

D. Plastic full crown

E. 3/4 crown

3.2010U4A1060 The role of opaque porcelain, except ( )

A. Increase the brightness of the porcelain layer

B. Cover up metallic colors

C. Chemical bonding of gold porcelain

D. Forming the base color of the gold porcelain crown

E. All of the above

4.2013U4A1005 The performance requirements for porcelain alloys are incorrect ( )

A. The lowest elastic mold

B. Good mechanical strength

C. Good casting performance

D. Small shrinkage deformation

E. Good wettability

5.2011U4A1026 The requirements for porcelain alloy are incorrect ( )

A. The modulus of elasticity is small

B. Good wettability

C. Small shrinkage deformation

D. Good casting performance

E. Not easy to deform and wear

6.2003U4A1012 The most important binding force in gold-porcelain bonding is ( )

A. Mechanical mating

B. van der Waals

C. Inverted retention

D. Chemical binding

E. Pressure bonding

7.2010U4A1061 For the reinforcement of gold alloy porcelain gold bonding, the method that can be adopted, except ( )

A. Sandblasting

B. Pre-oxidation

C. Ultrasonic cleaning

D. Degassing

E. Electrolytic etching

8.2010U4B1140 The cut end of the anterior gold porcelain crown should be at least ground ( )

A. 1mm

B. 1.5mm

C. 3.5m

D. 2.5mm

E. 3mm

9.2010U4A1062 The thickness of the lip porcelain layer in the anterior tooth gold porcelain crown is generally ( )

A. 0.5mm

B. 1mm

C. 2mm

D. 2.5mm

E. 3mm

10.2003U4A1013 The gingival margin of the lip of the gold porcelain crown is generally ( )

A. 0.5mm shoulder

B. 1mm shoulder

C. 1.2mm shoulder unit

D. 1.5mm shoulder

E. None of the above

11.2003U4A1014 Which of the following descriptions of the base crown of gold porcelain crown is wrong ( )

A. Good fit with the preparation

B. Support porcelain layer

C. The junction of gold porcelain is blade-shaped

D. Avoid the occlusal zone at the gold-porcelain junction

E. The lip surface is a porcelain layer with a gap of 0.85~1.2mm

12.2007U4A1005 PFM full crown shoulder width is generally ( )

A. 0.5mm

B. 1.0mm

C. 1.5mm

D. 2mm

E. 2.5mm

13.2000U4A1005 The production of metal porcelain crowns, the wrong statement is ( )

A. Full crown tongue side and neck margin are all metal

B. The gold-porcelain junction should avoid the occlusal functional area

C. The gold-porcelain junction is beveled and overlapped

D. The thickness of the crown on the bottom layer of the porcelain covering area is at least 0.5mm

E. The porcelain layer space in the porcelain coverage area shall not exceed 2mm

14.2004U4A2083 A patient, after the restoration of the metal porcelain crown of the right upper central incisor. Inspection: the metal porcelain crown is good in color and shape, not loose, the probe on the edge of the neck can be probed, the dental floss at the adjacent point can barely pass, and the lower right 1 insect end bites on the porcelain surface of the metal porcelain crown when the median tooth is occluded, and this crown is ( )

A. Qualified restorations

B. The adjacency is too tight

C. Adjacent over-loose

D. Improper design of the gold-porcelain bonding area

E. Misfit of the edges

15.2003U4A1015 The least likely cause of porcelain layer damage after fixed denture restoration is ( )

A. Early occlusal contact

B. Prepare 1 mm space on the lingual side of the anterior teeth

C. The metal material does not match the porcelain powder

D. Contamination of the surface of the metal substrate during production

E. 0.5mm space left on the occlusal surface of the fixed bridge base bridge

16.2011U4A1027 Excessive axonal process of the restoration can cause ( )

A. Gingival hyperplasia

B. Gum recession

C. Gingival epithelium attachment disruption

D. Gingivitis

E. Food impaction

17.2011U4A1028 Tooth defect restoration as a treatment device for ( )

A. Prevent the further development of dental lesions

B. Restoration of normal physiological functions

C. Prevent the occurrence of dental and periodontal support tissue lesions

D. Maintain the health of the oral and maxillary system and coordinate with all departments

E. All of the above are their functions

18.2011U4A1029 The requirements for tooth preparation are ( )

A. Remove the diseased tissue and stop the lesion from developing

B. Eliminate the shaft wall indentation and obtain a good positioning channel

C. Open up the space occupied by the restoration, and ensure that the restoration has a certain strength, thickness and beauty

D. Prepare good retention and resistance shapes, abrasion of long teeth, misaligned teeth, abnormal jaw teeth and adjacent teeth, and make appropriate preventive extensions

E. All of the above requirements should be met

19.2011U4A1030 Significance of close contact between the adjacent surface of the crown restoration and the adjacent tooth does not include ( )

A. Prevent food impaction

B. Maintain the stability of the tooth position and arch shape

C. Maintain mutual support with adjacent teeth and disperse the tooth resultant force

D. Helps each tooth maintain its own physiological movement while chewing

E. May increase chewing power

20.2010U4A1063 Which of the following statements about pulp protection in dental preparation is false ( )

A. Tooth preparation is done in stages

B. Light pressure grinding

C. Minimize teeth grinding

D. Prepare for a short time break, short time grinding

E. Water mist cooling

21.2011U4A1031 Which of the following methods can be taken in order to reduce the damage to the pulp when preparing the abutment tooth ( )

A. Water mist cooling

B. Intermittent cutting

C. Short time cutting

D. Light pressure grinding

E. All of the above are true

22.2008U4A1001 Restoration of the affected tooth, in order to prevent the occurrence of secondary caries, to do ( )

A. The prosthesis should correctly restore the anatomical morphology of the affected tooth

B. The restoration should be able to restore the function of the affected tooth

C. The restoration is as thick as possible to prevent bacterial intrusion

D. The edge line of the restoration should be located in the self-cleaning zone

E. All of the above

23.2009U4A1005 The disadvantages of the superior edge of the whole crown gingiva are ( )

A. Predisposition to secondary caries

B. Easy to cause plaque adhesion

C. The edges are not easy to seal

D. Unsightly in the anterior area

E. Easy to form shoulders

24.2010U4A1064 Factors that do not need to be considered when determining the position of the gingival margin of a fixed restoration are ( )

A. Tooth resultant force

B. Periodontal health status

C. Oral hygiene status

D. Aesthetics

E. Age

25.2010U4B1141 Usually the best choice for the gingival edge of the front teeth metal porcelain crown lip is ( )

A. Supragingival concave edge

B. Subgingival bladed edge

C. Supragingival shoulder margin

D. Subgingival concave edge

E. Gingival margin

26.2010U4B1142 Usually the best choice for posterior cast metal full crown gingival margin is ( )

A. Supragingival concave edge

B. Infragingival shoulder margin

C. Supragingival shoulder margin

D. Subgingival concave edge

E. Gingival margin

27.2010U4A1065 The gingival margin of the crown and the tongue of the gold porcelain is best ( )

A. 1mm shoulder

B. 0.5mm concave

C. Bladed

D. Chiseled

E. None of the above

28.2010U4B1143 The worst of the following edge types is ( )

A. Bladed

B. 90° shoulder

C. 90° shoulder with bevel

D. Concave

E. 135° shoulder table

29.2010U4B1144 The worst fit of the following edge types is ( )

A. Bladed

B. 90° shoulder

C. 90° shoulder with bevel

D. Concave

E. 135° shoulder table

30.2010U4A1066 Factors unrelated to the morphological design of the occlusal surface of the whole crown are ( )

A. Retention factors

B. Factors of Resistance

C. Periodontal status

D. Conjunct surface morphology

E. Whether the contralateral crown is intact

31.2010U4A1067 Which of the following is not a measure to increase the resistance of the restoration in the restoration of tooth defects is ( )

A. Use good materials

B. Avoid stress concentrations

C. Increase cusp inclination

D. Avoid casting defects

E. The gold porcelain articulation area of the gold porcelain crown is located away from the occlusal contact point

32.2011U4A1032 Which is wrong to increase the resistance of the prosthetic body ( )

A. Reduce the volume and thickness of the restoration so that more tooth tissue is preserved

B. Reasonably control the shape of the restoration, and avoid sharp, thin and sharp structural forms

C. According to the conditions and design requirements of the affected tooth, select the restoration materials with excellent physical and chemical properties

D. Ensure the quality of the restoration

E. Control the occlusal surface morphology and force direction

33.2011U4A1033 All-ceramic crown belongs to ( )

A. Metal full crown

B. Non-metallic full crown

C. Mixed full crown

D. Nuclear crown

E. Pile crown

34.2013U4A1006 The advantages of porcelain crown do not include which of the following ( )

A. The color is stable and natural

B. Low thermal conductivity

C. Good biocompatibility

D. Non-conductive

E. Low brittleness and high strength

35.2011U4A1034 The situation that should not be made porcelain full crown is ( )

A. Large anterior tooth proximal defect or multiple crown defects

B. Anterior teeth, fluorosis, discolored teeth, tetracycline-stained teeth, and enamel hypoplasia affect the appearance

C. Anterior teeth that are aesthetically pleasing due to dysplasia or dysplasia

D. Defects in primary teeth and juvenile permanent teeth with pulp affecting aesthetics

E. Misaligned and twisted teeth are not suitable for orthodontic treatment

36.2011U4A1035 The indication for casting the crown is ( )

A. Retainers for fixed dentures

B. Modify the shape of the tooth and restore the occlusion

C. Severe defect of the posterior tooth

D. Severe dentin hypersensitivity with tooth defect

E. All of the above

37.2006U4A1002 The abutment tooth morphology is normal, and the retainer with the largest retention force is ( )

A. Inlays

B. Full crown

C. Intrarhizous retain

D. Partial crown

E. Pile core crown

38.2000U4A2082 female, 40 years old, longitudinal fissure of the right maxillary first molar with pulp involvement, short clinical crown, tight occlusion, root canal treatment completed. The optimal prosthetic design for this case is ( )

A. Hammer the crown

B. Casting full crowns

C. Orthodontic inlays

D. Porcelain crown

E. Inlays

39.2010U4A1068 Which of the following is not an indication for casting a full crown ( )

A. Retainers for fixed dentures

B. Modify the shape of the tooth

C. Restore the occlusion

D. Dentin hypersensitivity

E. Stump

40.2011U4A1036 When the cast metal full crown tooth is prepared, the occlusal surface gap should be prepared ( )

A. 0.5mm

B. 0.8mm

C. 1.0mm

D. 2.0mm

E. None of the above

41.2011U4A1037 The occlusal surface clearance provided by the preparation of casting full crown teeth is generally ( )

A. 1～2mm

B. 0.8～1.5mm

C. 2.5mm

D. 1．5mm

E. 2.5～3mm

42.2013U4A1007 When preparing the inlay tooth, the wrong way to do it is ( )

A. Remove the decay of the lesion

B. The maximum circumference of the axial surface is lowered to the gingival margin

C. Provide good retention and resistance shapes

D. Proper abrasion of abnormal counter-jaw teeth

E. Preventive expansion

43.2011U4A1038 The casting temperature of the cast gold alloy is ( )

A. Above 1100°C

B. 850～1000℃

C. 500～1000℃

D. 850～300℃

E. 500～300℃

44.2011U4A1039 Which of the following statements about full crown retention is false ( )

A. The full crown retention force mainly comes from friction and adhesion

B. Friction is the most important retention force

C. The amount of friction is related to the fit of the restoration to the preparation

D. The occlusal surface morphology of the full crown is related to retention

E. Buccal-lingual diameter of the preparation is independent of retention

45.2010U4A1069 Which of the following measures is not conducive to increasing the friction of artificial crowns ( )

A. Increase the polymerization angle when preparing teeth

B. The contact surface is appropriately rough

C. The artificial crown is tightly integrated with the affected tooth

D. Try to keep the axial planes of the crown as parallel as possible

E. Point angles and line angles should be clear

46.2014U4A1023 Which is not a factor affecting the total crown retention ( )

A. Crown height

B. Crown diameter

C. Type of alloy used

D. Defects

E. Dental resultancy

47.2010U4B1145 The depth of the nail hole retention form is generally ( )

A. 1mm

B. 1.5mm

C. 3.5mm

D. 2.5mm

E. 3mm

48.2010U4A1070 Which of the following statements about the nail hole retention is wrong ( )

A. The amount of the nailing force depends mainly on the depth of the nail hole

B. The diameter of the retaining nail is about 1mm

C. Retaining nails can be divided into cemented type, threaded type, and wedge-in type

D. Wedge-in retention force is the best

E. The nail hole should be located in the solid tooth nature

49.2011U4A1040 The retainer of the tooth preparation of the restoration does not include ( )

A. Box retention

B. Dovetail retention

C. Inverted retention

D. Nail hole retention

E. Groove retention

50.2010U4A1071 Which of the following statements about the groove is false ( )

A. The depth is generally 1mm

B. Resistant to horizontal dislocation and occlusal dislocation

C. Consistent with neck 1/3 orientation

D. Reinforcement of restorations

E. Align with the direction of the inauguration path

51.2010U4A1072 Which of the following statements about the dovetail retainer is false ( )

A. Can increase the retention force of the restoration

B. Prevents horizontal dislocation of the restoration

C. The width of the Hatomi Gorge is about 1/2 of the width of the occlusal surface

D. The dovetail gorge should be located between the triangular crests of the occlusal surface

E. The preparation of the dove tail has an effect on the resistance of the preparation

52.2004U4A1036 The inclination of the slope of the cave edge is ( )

A. 30°

B. 45°

C. 60°

D. 70°

E. 25°

53.2013U4A1008 The following are not double-sided inlays ( )

A. Distal occlusal inlay

B. Buccal occlusal inlay

C. Medial occlusionDistal inlay

D. Tongue-occlusal inlays

E. Median occlusal inlay

54.2007U4A1006 The material that should not be used for inlay repair is ( )

A. Composite resins

B. Self-coagulating resins

C. Porcelain

D. Cast ceramics

E. Metals

55.2014U4A1024 Comparison with amalgam filling: The advantages of casting inlays are ( )

A. Excellent mechanical properties

B. Good retention

C. Short marginal lines

D. Less tooth incision

E. Easy to make

56.2010U4A1073 Indications for inlays, except ( )

A. Adolescent permanent teeth

B. Severe wear of the occlusal surface requires occlusal reconstruction

C. Occlusal brackets should be placed on the occlusal cavity

D. Tooth defect with broken proximal contact

E. None of the above

57.2000U4A1006 The purpose of preparing the inlay edge bevel does not include ( )

A. Increase the edge fit of the inlay

B. Increase the strength of the inlay

C. Reduction of micro-leakage

D. Prevent enamel breakage

E. Increase the closure of the inlay and the edges

58.2010U4A1076 Which of the following requirements for the 3/4 crown cut bevel of the anterior teeth is incorrect ( )

A. At a 90° angle to the long axis of the tooth

B. Prepare a gap of 0.7mm or more

C. The fangs should form two bevels, the mesiodistal and distal

D. The upper anterior teeth are obliquely from the labial side to the lingual side

E. The lower anterior teeth are obliquely from the lingual side to the labial side

59.2010U4A1077 The main function of the 3/4 crown sulcus of the anterior teeth is ( )

A. Prevent occlusion dislocation

B. Prevent lingual dislocation

C. Prevent mesiodistal and distal dislocations

D. Prevent labial dislocation

E. Prevent gingival displacement

60.2014U4A1025 Which of the following requirements for the front teeth 3/4 crown cut bevel is wrong ( )

A. At a 90-degree angle to the long axis of the tooth

B. Prepare a gap of 0.7mm or more

C. The fangs should form two bevels, the mesiodistal and distal

D. The cutting end groove can be prepared in the cutting bevel

E. Lower anterior teeth are prone to metal exposure

61.2010U4A1078 3/4 crown disadvantage ( )

A. Easy to get in place

B. It cannot be used as a fixed bridge retainer

C. Molars

D. Long outline line, not conducive to caries prevention

E. None of the above

62.2010U4A1079 The direction of the 3/4 crown sulcus of the upper anterior teeth is ( )

A. Parallel to the long axis of the tooth

B. Parallel to the labial cut 2/3

C. Parallel 1/3 to the labial face

D. Parallel to the labial gingiva 1/3

E. Parallel to the lingual carina

63.2010U4A1080 The direction of the 3/4 crown sulcus of the upper posterior teeth is ( )

A. Parallel to the buccal occlusion 2/3

B. Parallel to buccal gingiva 1/3

C. Parallel to the shaft wall

D. Parallel to the lateral wall of the tongue

E. None of the above

64.2010U4A1081 The position of the 3/4 crown sulcus of the upper posterior teeth is ( )

A. Adjacent cheek 1/3

B. Junction of the adjacent cheek 1/3 and the middle 1/3

C. 1/3 of the adjacent surfaces

D. The 1/3 of the adjacent surface is bordered by the 1/3 of the tongue

E. 1/3 of the adjacent tongue

65.2000U4A1007 Artificial crown fully in place sign, not included ( )

A. The bite is basically appropriate

B. No warping

C. Edge sealing

D. The edge reaches the design position

E. Proper tightness of the contact points

66.2014U4A2080 Male, 20 years old. Traumatic defect of upper anterior teeth for 7 days. No symptoms. Oral examination: right upper 1 mesial incision angle defect, gingival diameter greater than 2/3 of the crown, no penetration of the pulp cavity, normal temperature test, percussion (-), no loosening. X-ray shows that there is no root fold, and the most appropriate treatment is ( )

A. Nail-retained amalgam repair

B. Nail-retained glass ion repair

C. Nail-retained composite resin restoration

D. Nail-retained zinc phosphate cementing powder repair

E. Endodontic treatment followed by porcelain crown restoration

67.2003U4A1016 Which of the following statements about the possible causes of spontaneous pain after full crown bonding is false ( )

A. Pulpitis

B. Metallic microcurrent stimulation

C. Apical periodontitis

D. Gingivitis

E. All of the above is wrong

68.2003U4A2078 Spontaneous pain on the second day after cementation of mandibular bicuspids, pulp, and full crown restoration, the most likely cause is ( )

A. Pulpitis

B. Occlusal trauma

C. Apical periodontitis

D. Pulp congestion

E. None of the above

69.2003U4A1017 Gingivitis after full crown bonding, its possible causes, except ( )

A. Poor axial wall protrusion

B. The crown margin is too long

C. Crown margins are not sealed

D. Cementant residue in the sulcus

E. Early occlusal contact

70.2011U4A1041 Which of the following is not included in the causes of gingivitis after full crown repair ( )

A. Food impaction

B. Occlusal imbalance

C. The edges are too long

D. Misfitting edges

E. Poor finish with rough edges

71.2003U4AN123 47-year-old male. The maxillary anterior teeth have been fixed and restored for 3 years, and now they are loose and have painful bite. Oral examination: the upper left 2 is missing, the upper left 123 porcelain fixed bridge is repaired, and the upper left 13 is the porcelain full crown retainer, and the edges are not sealed. Deep caries can be detected on the distal proximal surfaces of the upper left 1 and upper left 3, and the upper left 3 percussion (++) shows red and swollen gingiva without periodontal pockets. The remaining teeth are healthy and the bite is normal. The reason for the loosening of the original fixed prosthesis is ( )

A. Breakage of the original fixed restoration

B. The edges of the original fixed prosthesis are not sealed

C. Gingivitis

D. Periodontitis

E. Poor abutment preparation

72.2003U4A1018 Short-term occlusal pain after full crown bonding, the main reason is ( )

A. Pulp hyperemia

B. Loose crown

C. Periodontitis

D. Traumatic occlusion

E. None of the above

73.2011U4A1042 Causes of Bond Repair Failure Do Not include ( )

A. Removal of the marrow protection material from the cave wall

B. The adhesive is too thick

C. The tooth surface is not thoroughly cleaned

D. Bonded restorations were moved before curing

E. Poor moisture insulation

74.2004U4A2084 patient, 2 months after the repair of the upper left 7 casting crown, loosened and fell off, examination: the preparation was good, and the occlusal examination revealed that there was a significant early contact point at the tip of the mesial tongue. The correct treatment is ( )

A. Align the jaw cusp

B. Alignment of the jaw early contact point

C. Use a better quality adhesive for bonding

D. Adjust the early contact points on the crown and re-glue after polishing

E. Redo the full crown

75.2011U4A1043 The most ideal restoration for the treatment of stumps is ( )

A. Inlays

B. Pile crown

C. Metal crown

D. Porcelain crown

E. 3/4 crown

76.2010U4A1082 According to the degree of tooth defect from small to large, the largest degree of tooth defect should be selected ( )

A. Full crown

B. Inlays

C. Pile crown

D. Partial crown

E. Onlays

77.2013U4A1009 Eligible for the indications for pile crown are ( )

A. Lateral puncture of the root canal wall

B. Root canal treatment has been performed, and the fistula orifice is not closed

C. Crown stump for fixed denture abutments

D. The anterior teeth are obliquely folded up to 1/3 of the roots

E. The root canal is slightly curved

78.2004U4A2085 A patient, 3/4 of the crown of the right upper central incisor is defective, no percussion pain, no loosening, no redness and swelling of the gums, X-ray shows that the tooth has been treated with a perfect root canal, and there is no shadow at the root tip, the most suitable treatment plan is ( )

A. Inlays

B. 3/4 crown

C. Open crown

D. Pile core + full crown

E. Full crown

79.2004U4A2086 A patient with 6 large areas of amalgam filling in the upper right requires repair treatment. Examination: a large area of mercury filling on the occlusal surface, less remaining hard tissue in the crown, only the thin wall of the buccal tongue remains, no loosening, no percussion pain, and the root canal treatment has been perfected. Best treatment plan ( )

A. Inlays

B. Onlays

C. Cast metal full crown

D. Porcelain full crown

E. Pile nucleus + full crown

80.2004U4A1037 Which item is wrong ( )

A. Preserve as much residual crown tissue as possible to increase the length of the crown pile

B. The root canal is prepared into an oval shape to reduce the taper of the root canal wall

C. Use the length of the root canal as much as possible, and the crown pile can reach the apex if needed

D. Shoulder preparation at the root and neck to increase the stability of the crown

E. Use cast crown piles to increase the tightness of the crown piles and the root canal wall

81.2004U4A2087 A patient, a large part of the crown of the anterior tooth is defected, and the length of the root canal preparation should reach the root length ( )

A. 1/5

B. 1/4

C. 2/5

D. 2/3

E. 4/5

82.2010U4A1083 The following method of increasing the crown pile retention, the incorrect is ( )

A. Prepare a small shoulder table at the root canal orifice

B. Increase the tightness of the pile and the pipe wall

C. Choose excellent binders

D. Preserve the residual crown hard tissue as much as possible

E. Remove the residual crown hard tissue as much as possible

83.2010U4A1084 Enhancement of pile core retention method, except ( )

A. Preserve the residual crown hard tissue as much as possible

B. Make use of root canal length as much as possible

C. Increase the tightness of the pile and the pipe wall

D. The root canal is prepared into a cylindrical shape

E. Proper use of binders

84.2010U4A1085 Which of the following statements about crown retention is false ( )

A. Adhesion is the most important retention force

B. The pile should be tightly sealed with the root canal wall

C. The longer the pile, the better the retention

D. The pile diameter is related to the retention

E. Pile morphology affects retention

85.2010U4A1086 Which of the following statements about the pile crown is false ( )

A. The longer the pile, the better the retention

B. The retention force depends on the friction and adhesion force

C. After the pile crown is repaired, the occurrence of apical disease is increased

D. Perfect root filling must be performed before repair

E. Retainer that can be used as a fixed bridge

86.2010U4A1087 In the repair of pile core crown, the error handling of the repaired residual crown is ( )

A. Cut along the top line of the papillae

B. Removal of decay

C. Remove the thin walls

D. Removal of baseless glaze

E. Preserve the residual crown hard tissue as much as possible

87.2010U4A1088 The following description of the shape of the pile crown, the correct one is ( )

A. The cross-section is circular

B. The transverse diameter is 1/2 of the root diameter

C. Seal with the root canal wall

D. Consistent with root length

E. Cylindrical from root canal mouth to root tip

88.2010U4A1089 The following incorrect operation in the preparation of the pile crown root canal is ( )

A. Look at the dental film first

B. Determine the pile length

C. Remove the root charge with a round drill

D. Use a crack drill to remove the root filling and trim the pipe wall for 1 time

E. Correct the drilling direction at any time when drilling

89.2011U4A1044 Temporary crown is not intended for ( )

A. Avoid re-irritation of the pulp

B. Maintain the tooth position of the affected tooth

C. Avoid wear on the occlusal surface

D. Maintain the mesial and distal space

E. Facilitation of the wearing of crowns

90.2000U4A2083 Male, 40 years old, dead pulp tooth of right lower first molar, restored with PFM full crown after root canal treatment, what kind of treatment is still needed before the full crown is first worn after tooth preparation and impression taking?

A. No processing is required

B. Temporary protective repair with a plastic crown

C. Protective repair with a metal crown

D. Make removable dentures to maintain clearance

E. Make a gap holder

91.2004U4A2088 Patient, 6 dead pulp teeth in the lower right, restored with a metal porcelain crown after root canal treatment, between the preparation of the tooth and the first wearing of the metal porcelain crown, ( )

A. Protective repair with a metal crown

B. Protective repair with a plastic crown

C. Make a gap protector

D. No Processing

E. Protect your gums with a gum protector

92.2003U4AN131 patient, a 30-year-old female, with 7 residual crowns in the upper left, has undergone root canal treatment and is now planned to be protected by crown repair. The most common material for the top 7 temporary crown on the left is self-coagulation resin. The cements commonly used for bonding temporary crowns are ( )

A. Zinc polycarboxylate cement

B. Zinc phosphate cement

C. Vitreous ionic cement

D. Calcium hydroxide cement

E. Zinc oxide cement

93.2003U4A1019 The most common causes of tooth defects are ( )

A. Caries disease

B. Trauma

C. Abrasion

D. Wedge defect

E. Developmental malformations

94.2011U4A1045 Definition of tooth defect ( )

A. Various tooth tissues with varying degrees of texture damage or abnormality

B. Damage or abnormality of various tooth tissues with varying degrees of physiological anatomy

C. Damage or abnormality of various tooth tissues with varying degrees of texture and physiological anatomical shape

D. Damage or abnormality of various tooth hard tissues with varying degrees of texture and physiological anatomy

E. Damage or abnormality of the soft tissues of the tooth to varying degrees

95.2010U4A1090 Which of the following factors is not related to the cause of tooth defect ( )

A. Wedge defect

B. Severe abrasion

C. Periodontal disease

D. Dental trauma

E. Caries disease

96.2010U4A1091 Which of the following causes of tooth defects is wrong ( )

A. Acid erosion

B. Plaque enamel

C. Tetracycline teeth

D. Night grinding

E. Wear occlusal pads

**GPT replied**

1. E。 Due to its excellent physical and aesthetic properties, the golden porcelain crown is suitable for a variety of dental restorations, including anterior misalignment, tapered teeth, fixed bridge retainers, and tetracycline tooth coloring.

2. C。 Metal porcelain crowns have good aesthetics and high strength, which is suitable for repairs with high aesthetic requirements and greater strength.

3. E。 The main functions of opaque porcelain include masking the metallic color, increasing the brightness of the porcelain layer, promoting the chemical bonding of gold porcelain and forming the base color of the gold porcelain crown, so option E (all of the above) is incorrectly described.

4. A。 The requirements for the performance of porcelain alloys include good mechanical strength, good casting properties, small shrinkage deformation and good wettability, and the minimum elastic mold is not one of the requirements.

5. A。 For porcelain alloys, the elastic modulus is moderate to ensure that the restoration has sufficient elasticity and deformation resistance, so the description of option A is incorrect.

6. D。 In gold-porcelain bonding, chemical bonding is considered to be the most important bonding force because it provides a strong bond between metal and porcelain.

7. C。 Reinforced gold alloy porcelain gold bonding is usually done by sand blasting, pre-oxidation, degassing and electrolytic etching, ultrasonic cleaning is not a direct way to enhance the bond.

8. B。 The cut end of the anterior gold porcelain crown is at least 1.5mm ground to ensure that there is enough space to place the porcelain and ensure the strength and beauty of the crown.

9. B。 The thickness of the lip porcelain layer in the anterior gold porcelain crown is generally 1mm, which can ensure the beauty and maintain sufficient strength.

10. E。 The description options for the labial gingival margin of the gold porcelain crown do not correctly match the description of the current dental restorative practice, so E is chosen (none of the above).

11. C。 The bladed joints of gold porcelain may cause the porcelain layer to detach or be damaged, so this is an incorrect description.

12. B。 PFM full crown shoulders are typically 1.0mm wide, which provides sufficient porcelain support while avoiding over-cutting of the tooth.

13. E。 The porcelain cover area of the metal porcelain crown does not exceed 2mm in order to ensure the strength and beauty of the crown, so this description is wrong.

14. E。 If the neck edge probe is reachable and the adjacent point dental floss barely passes, the edge is not tight, which is important for the long-term success of the restoration and gum health.

15. B。 The 1 mm space on the lingual side of the anterior teeth is unlikely to be the cause of the porcelain breakage, and is the least likely relative to other options.

16. A。 Excessive axonal process of the restoration can cause gingival hyperplasia because it can irritate or damage the gums, leading to inflammation and tissue growth.

17. E。 The functions of dental defect restorations include preventing the further progression of dental lesions, restoring normal physiological function, preventing teeth, and periodontal support tissues

Lesion occurrence and maintenance of the oral and maxillary system and coordination of various departments.

18. E。 The requirements for tooth preparation include removal of diseased tissue, elimination of axial wall inverts, opening up of space occupied by the restoration, and preparation of retained and resistant shapes.

19. E。 The significance of close contact between the adjacent surface and the adjacent tooth does not include an increase in chewing force, as this is mainly determined by the design of the occlusal surface and the structure of the tooth itself.

20. E。 In tooth preparation, water mist cooling is done to reduce the impact of thermal damage on the pulp, so option E (water mist cooling) is the right thing to do, not a misstatement.

21. E。 Ways to reduce damage to the pulp include water mist cooling, intermittent cutting, short time cutting, and light pressure grinding, so option E (which is true of all of the above) is correct.

22. E。 In order to prevent secondary caries, the prosthesis needs to be properly restored to the anatomical shape and function of the affected tooth, with the edge line located in the self-cleaning zone to prevent bacterial invasion as much as possible.

23. D。 One of the disadvantages of the unsightly upper edge of the full crown gingiva in the anterior area is that it may affect the smile line and the aesthetic effect.

24. A。 The size of the tooth force is not a necessary factor when determining the gingival margin position of a fixed restoration, as factors such as periodontal health, oral hygiene, aesthetics, and age are the main considerations.

25. C。 Usually the best choice for the gingival edge on the labial side of the anterior metal porcelain crown is the supragingival shoulder margin, which helps to achieve a good aesthetic effect and edge adaptation.

26. B。 Often, the best choice for a cast metal full crown gingival margin of the posterior teeth is the subgingival shoulder margin, which helps to provide adequate prosthetic support and is easy to clean.

27. A。 The gingival edge of the crown is preferably a 1 mm shoulder to provide good margin sealing and prosthetic support.

28. A。 The edge of the blade is the least strong because it is prone to stress concentrations that can lead to breakage of the restoration.

29. D。 The concave edge type has the worst fit because its shape is not easy to fit and fit the edges.

30. E。 The factor that is not related to the design of the occlusal surface morphology of the full crown is whether the contralateral crown is intact or not, because the design mainly considers retention factors, resistance factors, periodontal status, and adjacent surface morphology.

31. A。 The use of good materials is one aspect of increasing the resistance of the restoration, not not the measure of increasing the resistance of the restoration.

32. A。 Reducing the volume and thickness of the restoration may weaken the strength of the restoration and is not the right measure to increase the resistance of the restoration.

33. B。 The all-porcelain crown is made entirely of porcelain material, so it belongs to the non-metallic full crown.

34. E。 One disadvantage of porcelain full crown is that it is brittle and relatively low strength, excluding "small brittleness and high strength".

35. D。 When the tooth defects of deciduous teeth and juvenile permanent teeth with pulp affect the aesthetics, conservative restoration measures are more likely to be taken, and porcelain full crown is not appropriate.

36. E。 Indications for full crown casting include retaining as a fixed denture, modification of tooth contour, restoration of occlusion, severe posterior tooth loss, and severe dentin hypersensitivity with tooth defect.

37. B。 In the case of normal abutment tooth morphology, a full crown provides the greatest retention force because it covers the entire tooth.

38. B。 Cast full crowns are suitable for cases where the longitudinal fissure of the occlusal surface is hidden and the pulp is involved, especially in the case of a short clinical crown and a tight occlusion, providing a firm restoration and protection.

39. E。 The indication for casting a full crown does not include stumps, as stumps need to be properly reconstructed first.

40. C。 When preparing a cast metal full crown tooth, the occlusal surface gap usually needs to be prepared 1.0mm to ensure sufficient metal thickness to ensure the strength of the restoration.

41. B。 The occlusal surface gap provided by the preparation of the cast full crown tooth is generally 0.8~1.5mm, which can ensure sufficient material strength and appropriate thickness of the restoration.

42. E。 Preventive extension is not recommended when preparing for inlay teeth as it may result in unnecessary removal of tooth tissue.

43. B。 The casting temperature of cast gold alloy is generally 850~1000°C, which is suitable for the casting process of most gold alloys.

44. E。 The occlusal morphology of the full crown is related to retention, but the buccal-lingual diameter of the preparation also has an effect on retention, as it determines the surrounding friction of the restoration.

45. A。 Increasing the polymerization angle during tooth preparation may reduce friction and is therefore not conducive to increasing the friction of the artificial crown.

46. C。 The type of alloy used mainly affects the physical and chemical properties of the restoration, rather than the retention force.

47. C。 The depth of the nail hole retention shape is generally 3.5mm to ensure sufficient retention force.

48. E。 All the options given correctly describe the characteristics of the nail hole retained shape, there are no false statements.

49. E。 The retaining forms of the prosthesis tooth preparation include box retention, dovetail retention, inverted concave retention, and nail hole retention, and groove retention is also a form of retention, so there is nothing that is not included.

50. E。 Consistent with the orientation of the in-place tract is a mischaracterization, as the groove position usually coincides with the direction of the primary resistance to resist dislocation.

51. E。 It is correct that the preparation of the dove tail has an effect on the resistance of the preparation, this option is not

52. B。 The slope of the edge of the hole is generally recommended to be 45°, which is an angle that effectively increases the edge strength of the inlay and reduces microleakage.

53. D。 Tongue-occlusal inlays are not double-sided inlays because double-sided inlays involve occlusal surfaces and at least one adjacent face.

54. B。 Self-coagulating resin is not suitable as a material for inlay restoration, because its physical properties are inferior to other materials, and it is not suitable for long-term resistance to mechanical loads in the oral cavity.

55. A。 The advantages of cast inlays over amalgam fillings include better mechanical properties, which make them more durable and suitable for restoring the shape and function of the tooth.

56. E。 All options are indications for inlays, there are no unsuitable cases, so the correct answer is E.

57. E。 The purpose of preparing the inlay edge bevel does not include increasing the sealing effect of the inlay with the edge, which is not the direct purpose of the edge bevel preparation.

58. A。 It is incorrect to be at an angle of 90° to the long axis of the tooth, as the 3/4 crown bevel should be angled to the long axis of the tooth to ensure the aesthetics and functionality of the restoration.

59. C。 The main function of the 3/4 crown sulcus of the anterior teeth is to prevent mesiodistal and distal dislocations and ensure the stability of the prosthesis in the oral cavity.

60. E。 It is a mistake for the lower anterior teeth to be exposed to metal, as properly designed and made anterior 3/4 crowns should avoid metal exposure and remain aesthetically pleasing.

61. B。 One of the disadvantages of the 3/4 crown is that it cannot be used as a retainer for a fixed bridge, as it does not provide sufficient support and retention force.

62. B。 The orientation of the 3/4 crown sulcus of the upper anterior teeth should be parallel to the labial incision 2/3, which contributes to the aesthetics and functionality of the restoration.

63. A。 The orientation of the 3/4 crown sulcus of the upper posterior teeth should be parallel to the buccal occlusion 2/3 to ensure the adaptability and function of the restoration.

64. C。 The position of the 3/4 crown sulcus of the upper posterior teeth should be located in the middle 1/3 of the adjacent surface, which contributes to the stability and aesthetics of the restoration.

E。 The proper tightness of the contact point is not directly related to the signs that the artificial crown is fully in place, and the main signs include proper occlusion, no warping, and edge sealing.

65.E。 The proper tightness of the contact point is not directly related to the signs that the artificial crown is fully in place, and the main signs include proper occlusion, no warping, and edge sealing.

66.C。 Nail-retained composite resin restoration is suitable for the repair of defects that do not penetrate the pulp cavity, because it is beautiful and can restore the tooth shape well.

67.E。 Possible causes of spontaneous pain include pulpitis, metallic microcurrent irritation, apical periodontitis, and gingivitis, so option E "all of the above is wrong" is wrong.

68.A。 The most likely cause of spontaneous pain after a full crown repair is pulpitis, as this is the most direct cause of pain.

69.E。 Early occlusal contact is not the cause of gingivitis after full crown bonding, and all other options may cause gingivitis.

70.E。 Poor finish and rough edges do not directly lead to gingivitis, but can lead to a build-up of bacteria, which in turn can cause gingivitis.

71.B。 Loose edges of the original fixed restorations are a direct cause of loosening of the restorations, as the unfitting edges can lead to bacterial intrusion, which in turn can cause dental problems.

72.D。 Traumatic occlusion is the leading cause of occlusal pain in the short term after full crown bonding, as inappropriate occlusal force can act directly on the tooth.

73.A。 Removal of the wall sep material is not the cause of the failure of the adhesive repair, but is a necessary step in some cases.

74.D。 Adjusting the early contact points on the full crown and re-cementing after polishing is the correct way to deal with it, as the early contact points can cause the full crown to loosen.

75.B。 Pile crown is the most ideal restoration for stump treatment because it provides good retention and support.

76A。 A full crown should be used when the tooth is most defective, as it provides the most comprehensive coverage and protection.

77.C。 Crowns and remnants for fixed denture abutments are eligible for pile-crowning because they provide the necessary support and retention for damaged teeth.

78D。 Pile nucleus + full crown is the most suitable treatment option, as most crown defects require additional support to restore function and aesthetics.

79.C。 A cast metal full crown is the best treatment option because it provides enough strength to support a tooth with little remaining hard tissue.

80.E。 Increasing the fit of the crown pile with a cast crown pile and the root canal wall is the right way to enhance the crown retention, not the wrong one.

81.D。 The length of the root canal preparation should reach 2/3 of the root length to ensure adequate support and retention.

82. E。 When crown pile retention is added, the residual crown hard tissue should be preserved to provide better support and retention.

83. D。 Preparing the root canal into a cylindrical shape does not necessarily enhance core retention, as it may result in a thinner root canal wall and reduced retention force.

84. C。 The length of the pile is not always better, as too long a pile may cause stress concentration at the root and increase the risk of root fracture.

85. C。 Pile crown restoration does not increase the occurrence of apical disease, but rather restores the function and aesthetics of the tooth.

86. E。 In the core crown restoration, the residual crown hard tissue should be preserved as much as possible to provide better support and retention.

87. C。 The shape of the pile crown should be tightly confined to the root canal wall to provide good retention.

88. D。 Using a crack drill to remove the root filling and trim the tube wall at the same time may result in unwanted root canal damage.

89. E。 The purpose of a temporary crown is not to facilitate the wearing of the crown, but mainly to protect and maintain the tooth position.

90. B。 A temporary protective restoration should be performed with a plastic full crown to protect the tooth after tooth preparation before the full crown is first worn.

91. B。 Between the first wear of the metal porcelain crown, a plastic full crown is usually used for protective restoration.

92. A。 The cementing commonly used for bonding temporary crowns is the zinc polycarboxylate cement, because of its good adhesion and easy removal.

93. A。 The most common cause of tooth defects is caries, as it is the main cause of tooth tissue destruction.

94. D。 Tooth defects are defined as damage or abnormalities in the texture and physiological anatomical shape of various tooth hard tissues.

95. C。 Periodontal disease is a disease that affects the tissues around the teeth and is not directly related to the cause of the tooth loss.

96. E。 Occlusal pads are worn to prevent abnormal contact between teeth and are not the cause of tooth loss.

Chapter 4 Fixed partial denture restoration of dentition defects

1.1999U4A1009 Which of the following is the most important support base for fixed bridges ( )

A. Periodontal ligament

B. Alveolar bone

C. Gums

D. Binding epithelium

E. Mucous membranes

2.2011U4A1048 Among the following oral conditions, fixed restoration can be used ( )

A. Absence of the distal free end of the dentition

B. The number of tooth missing teeth is large and the gap span is large

C. Anterior region with severe jaw defect

D. Anterior teeth are missing, and the abutments on both sides are stable

E. All of the above conditions are suitable for fixed restoration

3.2014U4A1026 Which of the following is not an indication for fixed bridges ( )

A. Single anterior tooth is missing

B. The occlusal relationship is normal

C. Periodontal tissue health of abutments

D. Anterior tooth loss with massive bone defects

E. Orthomaxillary teeth are normal

4.2000U4A1009 Need to consider increasing the number of abutment teeth for the purpose of ( )

A. The abutment tooth is a single tooth

B. Slight tilt of the abutment

C. Abutment periodontal ligament widening

D. Abutments without countermaxillary function

E. Alveolar bone resorption of more than 1/3

5.2008U4A1002 as the abutment of the fixed bridge, must be ( )

A. Pulp tooth

B. The tooth tissue is healthy and intact

C. Periodontal tissue health

D. The abutment teeth are aligned normally without inclination

E. The abutment teeth should not be bitten too tightly

6.2008U4A1003 Which item is the least important when choosing a fixed abutment tooth ( )

A. Ability to retain abutments

B. Common alignment of the abutments

C. The abutment tooth must be a pulp tooth

D. Periodontal tissue health of the abutments

E. Abutment support capacity

7.2011U4A2090 Fixed dentures can only be made if the alveolar ridge in the missing area is well healed, and the fixed bridge should be made after tooth extraction ( )

A. Half Moon

B. January

C. March

D. June

E. 1 year

8.2014U4A1027 The best time for fixed bridge repair after tooth extraction is ( )

A. 1 month

B. 2 months

C. 3 months

D. 4 months

E. 6 months

9.2010U4A1094 What kind of auxiliary examination should be done first if there is occlusal pain after a period of fixed bridge repair?

A. Test the pulp viability of the abutment teeth

B. Check tooth looseness

C. Take an X-ray

D. Inspect the right teeth

E. CT scan of the brain

10.2007U4A1007 Persistent pain in the affected tooth after cementing the pulp restoration, most likely ( )

A. Has developed pulpitis

B. Presence of gingivitis or periodontitis

C. Has developed apical periodontitis

D. Dentin hypersensitivity

E. Has secondary caries but has not developed pulpitis

11.2013U4A2079 male, 30 years old. The right lower 6 metal crown fell off after 1 week of cementation, and the bite often had instantaneous pain. Oral examination showed that the patient had a tight bite, a short crown, an amalgam filling on the opposite surface of the jaw, and a complete crown that had fallen off. The most likely cause of momentary pain in the bite is ( )

A. Pulpitis

B. Apical periodontitis

C. Metal microcurrent stimulation

D. Periodontitis

E. Gingival marginitis

12.2011U4A1049 The advantages of the fixed bridge do not include ( )

A. Stable, retained, well supported, able to fully restore part of the chewing function lost due to missing teeth

B. Similar in size and shape to the tooth of the same name in the original dentition, there is no foreign body sensation, and it does not affect the functional activity of the tongue

C. There are many tooth tissues to be removed, and the scope of application is small

D. High aesthetics

E. The positive force of the teeth is shared by the abutment teeth

13.2011U4A1050 The selection of fixed abutment conditions is not important ( )

A. Abutment support capacity

B. Ability to retain abutments

C. Common alignment of the abutments

D. Periodontal tissue health of the abutments

E. The abutment tooth must be a living pulp

14.2010U4A1095 When using fixed bridge restoration, the influencing factors to determine the number of abutment teeth are ( )

A. Root condition of adjacent teeth

B. Alveolar bone condition of adjacent teeth

C. Periodontal ligament condition of adjacent teeth

D. Occlusion

E. All of the above

15.2011U4A1051 When selecting a fixed abutment, the minimum clinical crown-root ratio is ( )

A. 1:1

B. 1:2

C. 2:3

D. 3:2

E. 3:4

16.2010U4A1096 The conditions that the retainer should have are ( )

A. The retained body should have a good retained shape and resistance shape

B. The retainers should be able to obtain the common alignment channels required to secure the bridge

C. The material used in the retainer should achieve a certain mechanical strength

D. The retainer should be able to protect the tooth tissue

E. All of the above

17. 2011U4A1052 Which retainer design is wrong ( )

A. Inlays have the worst retention effect

B. The longer the span of the bridge, the more curved, and the greater the tooth force, the smaller the retention force of the retaining body

C. The retention force of the retained body at both ends of the abutment teeth should be basically equal

D. There must be a common path of inposition between the retainers

E. Abutment cusp fracture should be prevented

18.2014U4A1028 The following statement about the double-ended fixed bridge retainer is wrong ( )

A. There must be a common way to be in place

B. The retention force of the retainer at both ends should be substantially equal

C. The retention force of the retainer should be adapted to the size of the tooth resultant force

D. The co-alignment tract should be parallel to the long axis of the tooth

E. The retention force of the retainer should be adapted to the span of the bridge

19.2011U4A1053 The occlusal surface of the bridge body should be made ( )

A. The limbic crest morphology should be restored correctly

B. The occlusal surface of the bridge body should form the buccal and lingual grooves

C. A certain abduction gap and adjacent gap should be formed between the bridge and the retained body

D. Occlusal surface: Functional, cusp contact with countermaxillary teeth should be even

E. All of the above should be done

20.2003U4A1020 There is at least 3mm gap between the gingival surface and the mucosa of the suspended bridge, mainly because ( )

A. It is easy for food to pass through and has a self-cleaning effect

B. Does not irritate and compress gum tissue

C. Simple to make

D. The patient feels comfortable

E. Convenient food massage on the gums

21.2008U4A1004 The degree of bending of the bridge body is the same as ( ) when the fixed bridge is stressed.

A. The cube of the bridge thickness is inversely proportional

B. The cube of the length of the bridge is proportional

C. Bridge width is inversely proportional

D. The hardness of the bridge is related

E. All of the above

22.2014U4A1029 Under the same conditions, if the thickness of the fixed bridge body is halved, its deflection deformation becomes ( )

A. Doubled the original size

B. Increase to 4 times

C. Increase to a factor of 6

D. Increase to 8 times

E. Increase to a factor of nine

23.2014U4A1030 The following statement about semi-fixed bridges is wrong ( )

A. Tilt the abutments to obtain a common alignment tract

B. Protect the abutment tooth with weak support on the side of the gap

C. Multi-unit fixed bridge with intermediate abutment to protect intermediate abutment

D. The bolt of the movable connector is located on the retainer

E. Movable connectors are generally bolted attachments

24.2003U4AN125 47-year-old male. The maxillary anterior teeth have been fixed and restored for 3 years, and now they are loose and have painful bite. Oral examination: the upper left 2 is missing, the upper left 123 porcelain fixed bridge is repaired, and the upper left 13 is the porcelain full crown retainer, and the edges are not sealed. Deep caries can be detected on the distal proximal surfaces of the upper left 1 and upper left 3, and the upper left 3 percussion (++) shows red and swollen gingiva without periodontal pockets. The remaining teeth are healthy and the bite is normal. The best restoration method for the upper left 13 after correct tooth treatment is ( )

A. Porcelain full-crown fixed bridge restoration

B. Removable denture restoration

C. Cast metal fixed bridge repair

D. Full mucosal support removable denture restoration

E. Retained fixed denture restoration with 3/4 crown

25.2000U4A2084 Female, 55 years old. Due to the loss of 67 in the upper right position, the 4567 fixed denture in the upper right was repaired, and the abutment teeth had bite pain and loosening one year later. The main reasons for this are ( )

A. Unreasonable design

B. Insufficient retention force

C. Low number of abutments

D. End side sinking

E. Early occlusal contact

26.2011U4A1054 Reasons for poor retention of double-ended fixed bridge are ( )

A. Improper design of the retainer and insufficient retention

B. The number of abutments at both ends is unequal

C. When the abutment tooth is prepared, the axial surface polymerization is too small

D. Insufficient bridge strength

E. The retention force of one end of the retainer is slightly higher than that of the other end

27.2011U4A1055 Which is not a component of a fixed denture ( )

A. Retainers

B. Abutments

C. Bridge

D. Connectomes

E. All of the above components are fixed dentures

28.2011U4A1056 Which of the following fixed bridges belongs to special structure fixed bridges ( )

A. Double-ended fixed bridge

B. Single-ended fixed bridge

C. Semi-fixed bridges

D. Composite fixed bridges

E. Implant abutment fixed bridge

29.2010U4A1097 The main advantages of designing a semi-fixed bridge are ( )

A. Stress buffering

B. In order to attain a common path of inauguration

C. Aesthetics

D. Easy to make

E. Inexpensive

30.2011U4A1057 Which of the following statements is wrong about composite fixed bridges ( )

A. Generally includes 4 or more dentals

B. It is more difficult to achieve a common path

C. The abutment teeth are evenly stressed and are not easy to loosen and fall off

D. Composed of a combination of 2 or more simple fixed bridges

31.2007U4A1008 In the narrative about composite fixed bridges, the correct one is ( )

A. Contains four or more dental units

B. Contains more than two abutment teeth

C. Consists of a combination of two or three basic types of fixed bridges

D. The number of abutments is large and scattered, and it is not easy to obtain a common alignment tract

E. All of the above

32.2004U4A1038 The resultant force of the teeth is completely borne by one side of the abutment, which fixed bridge ( )

A. Double-ended fixed bridge

B. Single-ended fixed bridge

C. Semi-fixed bridges

D. Composite fixed bridges

E. Spring Bridge

33.2011U4A1058 The upper right 2 is missing, and the condition for designing a single-ended fixed bridge with the upper right 3 as the abutment is ( )

A. The upper right 2 has a large gap

B. Upper right 3 alveolar bone resorption exceeds 1/3 of the root length

C. The gap in the upper right 2 is small and the force is not large

D. The patient's tooth force is too large

E. The upper right 3 crowns are short

34.2004U4A2089 40-year-old female, 67 missing lower left, 8 lower left not erupting, and the remaining teeth of the oral cavity are healthy. The occlusion is normal. Pre-fixed denture restoration, the correct design is ( )

A. The lower left 45 was used as an abutment for a single-ended fixed denture

B. Single-ended fixed denture restoration for abutment teeth in lower left 345

C. Single-ended fixed denture restoration for the abutment tooth in the lower left 2345

D. Single-ended fixed denture restoration for the abutment tooth in the lower left 5

E. Single-ended fixed denture restoration is not recommended

35.2004U4A2095 Patient, female, 26 years old, lost an anterior tooth due to trauma 2 months ago, and now requires porcelain restoration. Oral examination: the left upper incisor is missing, the space is normal, and the alveolar ridge has no obvious absorption. 1/2 of the crown of the left upper central incisor is defective, the pulp has been exposed, the probe is slightly sensitive, the percussion is negative, and there is no loosening. The crown of the left upper canine is good, negative to percussion, and there is no loosening. There was no abnormality in the remaining teeth. The most suitable treatment options are ( )

A. Pile crown partial dentures

B. Overdentures

C. Pile core and double-ended fixed bridge

D. Intraroot retainer fixed bridge

E. None of the above

36.2004U4A2096 45-year-old male, the lower right 6 is missing, the lower right 7 is stable, the lower right 5 periodontal condition is poor, if it is repaired according to the double-ended fixed bridge, it should be designed as ( )

A. The lower right 5 is best designed with a full crown retainer

B. Lower right 5 is best designed with intracoronal retainers

C. Fixed denture restoration with the lower right 457 as the abutment

D. Reduce the tip slope of the bridge surface

E. Appropriately reduce the buccal-lingual diameter of the pontine body

37.2004U4A1044 The quality standards that should be met by the restorations are ( )

A. Sufficient retention force, correct shape, adjacency and occlusion

B. The edge is close to the affected tooth

C. The bite force is coordinated with the periodontal tissues to restore the function of the oral and maxillary system

D. It should be in place smoothly, the color is natural and beautiful, the surface is smooth and clean, the self-cleaning effect is good, and there is no adverse stimulation

E. All of the above

38.2014U4A1038 The best material used for the gingival surface of the bridge body is ( )

A. Ceramics

B. Medium-melt alloys

C. High-melt alloys

D. Thermoset plastics

E. Self-corositing plastics

39.2010U4A2133 If one end of the double-ended fixed bridge is designed as a 3/4 crown retainer, and the other end is a full crown retainer, which of the following methods should be used when making the wax type of the fixed bridge?

A. Made directly in the mouth

B. Fabrication on a model

C. Make it on the model first, then finish it in the mouth

D. First make it in the mouth and then finish it on the model

E. None of the above is appropriate

GPT replied

1. B. Alveolar bone. Alveolar bone provides the primary support for fixing the bridge.
2. D. Anterior teeth are missing, and the abutments on both sides are stable. Fixed restorations are suitable for cases where there are solid abutments on both sides.
3. D. Anterior tooth loss with massive bone defects. Bone defects may require pre-emptive bone regeneration therapy and is not an immediate indication.
4. E. Alveolar bone resorption of more than 1/3. When alveolar bone resorption is high, an increase in the number of abutments may be needed to provide more support.
5. C. Periodontal tissue health. The abutment teeth must have healthy periodontal tissue to provide stable support.
6. C. The abutment tooth must be a pulp tooth. Whether or not the abutment is a pulp tooth is not the most important thing in choosing the abutment tooth to act as a fixed bridge.
7. D. June. It is generally recommended to wait for a period of time after tooth extraction to allow the alveolar ridge to heal before proceeding with the bridge fixation.
8. E. 6 months. Waiting 6 months after tooth extraction is ideal for the alveolar bone to heal.
9. A. Test the pulp viability of the abutment teeth. Painful bite can be a problem with the pulp, and pulp viability needs to be tested first.
10. A. Has developed pulpitis. Prolonged persistent pain after cementation may be pulpitis.
11. C. Metal microcurrent stimulation. Metal crowns and amalgam fillings may form galvanic couples that generate microcurrent stimulation.
12. C. There are many tooth tissues to be removed, and the scope of application is small. This is a disadvantage rather than an advantage of fixed bridges.
13. E. The abutment tooth must be a living pulp. When choosing a fixed bridge abutment, it does not matter whether the abutment is a pulp tooth or not.
14. E. All of the above. Determining the number of abutments is influenced by a number of factors, including the root condition of the adjacent teeth, alveolar bone, periodontal ligament and occlusion.
15. A. 1:1。 The minimum clinical crown-to-root ratio is usually 1:1 to ensure adequate support.
16. E. All of the above. The retainer needs to have a good retention shape and resistance shape, have a common positioning channel, sufficient mechanical strength, and be able to protect the tooth tissue.
17. B. The longer the span of the bridge, the more curved, and the greater the tooth force, the smaller the retention force of the retaining body. This statement is false, in fact, the greater the retention force requirement.
18. D. The co-alignment tract should be parallel to the long axis of the tooth. This statement is false, and the common alignment tract is not necessarily parallel to the long axis of the tooth.
19. E. All of the above should be done. The occlusal surface of the bridge should be designed to ensure correct marginal ridge morphology, formation of buccal and lingual grooves, proper interspacing, and uniform contact with the opposing teeth.
20. A. It is easy for food to pass through and has a self-cleaning effect. Leaving a gap helps food pass through and reduces food impaction.
21. E. All of the above. The degree of bending of a bridge is related to its thickness, length, width and hardness.
22. D. Increase to 8 times. With the thickness halved, the amount of flexural deformation increases significantly according to the principles of material mechanics.
23. A. Tilt the abutments to obtain a common alignment tract. This statement is false, the semi-fixed bridge was not designed to obtain a common positioning channel.
24. A. Porcelain full-crown fixed bridge restoration. After proper dental treatment, a porcelain full crown bridge is a suitable restoration.
25. D. End side sinking. Abutment teeth have painful occlusion and loosening, possibly due to the end side of the tooth sinking.
26. A. Improper design of the retainer and insufficient retention. Poor retention is often caused by improper retaining body design or insufficient retention force.
27. E. All of the above components are fixed dentures. Retainers, abutments, bridges, and connectors are all components of fixed dentures.
28. C. Semi-fixed bridges. A semi-fixed bridge is a fixed bridge of a special structure.
29. A. Stress buffering. Semi-fixed bridges are designed primarily to cushion stress and protect abutment and periodontal tissues.
30. C. The abutment teeth are evenly stressed and are not easy to loosen and fall off. This statement is incorrect, because the composite fixed bridge may cause some of the abutment teeth to be overstressed due to uneven distribution of the abutments.
31. E. All of the above. These are the characteristics of composite fixed bridges.
32. B. Single-ended fixed bridge. The positive force of the teeth is completely borne by one abutment tooth, which is the characteristic of single-ended fixed bridges.
33. C. The gap in the upper right 2 is small and the force is not large. Single-ended fixed bridges are suitable for situations where the force requirement is not significant.
34. A. The lower left 45 was used as an abutment for a single-ended fixed denture. According to the description of the title, this is the most appropriate design.
35. C. Pile core and double-ended fixed bridge. Considering that the left upper central incisor has exposed pulp, the pile-nucleus and double-ended fixed bridge are suitable treatment options.
36. D. Reduce the tip slope of the bridge surface. For abutments with poor periodontal conditions, the effect of bite force should be reduced.
37. E. All of the above. The quality criteria to be met for restorations include retention force, shape, adjacency and occlusal relationship, edge sealing, coordinated occlusion and aesthetics.
38. A. Ceramics. The application of ceramic materials on the gingival surface of the bridge body has good aesthetics and biocompatibility.
39. B. Fabrication on a model. Making on the model allows for more precise control over the shape and size of the retainers.

**Chapter 5 Removable partial denture restoration of dentition defects**

1.2014U4A1031 The reason for the difficulty in placing a removable partial denture cannot be ( )

A. The ring arm is too tight

B. The snap ring body enters the abutment tooth inverted

C. The base enters the inverted

D. High occlusion of occlusal support

E. Deformation of dentures

2.2011U4A1059 The reason for the deformation of the bracket is the most unlikely ( )

A. The impression material is not of good quality

B. Improper design

C. The casting channel is not set up reasonably

D. Model defects

E. The coefficient of thermal expansion of the high-temperature embedding material is insufficient

3.2006U4A1003 What does not make it difficult to remove partial dentures is ( )

A. The base enters the tissue inverted

B. The snap ring arm is too tight

C. Inposition in the wrong direction

D. The tip of the arm enters the inverted hole too deeply

E. The base does not fit into the mucosal tissue

4.2008U4A1005 Kennedy Type 1 Dentition Defect ( )

A. Unilateral free deletion

B. Bilateral free deletions

C. Non-dissociative absence

D. Absence of space

E. Anterior tooth loss

5.2008U4A1006 According to Kennedy's taxonomy, 8721|1278 deletions belong to ( )

Category I, subcategory I

B. Category II, subcategory I

C. Category II, subcategory II

D. Category III and subcategory II

E. Category IV and subcategory II

6.2008U4A1007 According to the Kennedy taxonomy, 8765278 deletions belong to ( )

Category I, subcategory I

B. Category I and subcategory II

C. Category II, subcategory I

D. Category II, subcategory II

E. Category III, subcategory I

7.2008U4A1008 According to the Kennedy classification of dentition defects, those with 8743211278 defects belong to ( )

A. Category I, subcategory II

B. Category I, subclass I

C. Category II, subcategory I

D. Category IV and subcategory II

E. Category IV

8. 2011U4A1060 Bilateral free end deletion belongs to Kennedy classification ( )

A. Category I

Category II

C. Category III

Category IV

E. Category I. Classification

9.2011U4A1061 Dentition defect Kennedy classification class 1 ( )

A. Unilateral free end is missing

B. Bilateral free end deletion

C. Unilateral non-free end deletion

D. Bilateral non-free end deletions

E. Anterior tooth loss

10.2008U4A1009 According to the Kennedy taxonomy, 8765246 deletions belong to ( )

A. Category I, subcategory II

B. Category I, subcategory III

C. Category II, subcategory II

D. Category II and sub-categories

E. Category III and subcategory

11.2008U4A1010 According to the Kennedy classification of dentition defects, those with 652112346 defects belong to ( )

A. Category III and subcategory II

B. Category III, subcategory I

C. Category II, subcategory II

D. Category IV and subcategory II

E. Category IV

12.2008U4A1011 According to the Kennedy classification of dentition defects, those with 432112 defects belong to ( )

A. Category III and subcategory II

B. Category III, subcategory I

C. Category III

D. Category IV and subcategory II

E. Category IV

13.2004U4A2090 Maxillary Left 1234 Right 123456 Missing Category Which category does Kennedy classification belong to ( )

A. Category III and subcategory II

B. Category III and subcategory III

C. Category III

Category IV, subcategory I

E. Category IV

14.1999U4A2080 A patient has a loss of lower right 6, and the lower right 5 and lower right 7 are healthy, and the gap is normal. The fulcrum line of a removable partial denture can be designed as ( )

A. Slash

B. Straight

C. Horizontal line

D. Longitudinal type

E. Flat type

15.2000U4A2085 Male, 60 years old, wearing dentures for 2 days, felt pain when moving the upper lip downward, and difficulty in removing the dentures. Check: 12 partial dentures can be removed from the upper right 1234567 upper left, 37 single-arm snap rings on the upper left, the snap rings fit with the abutments, the base of the upper anterior arch area is too long, and the resistance to remove the dentures is greater. The rest is unusual. Causes of pain and difficulty in removing dentures are other than ( )

A. The snap ring is too tight

B. The base fits snugly against the tooth surface

C. The base enters the undercut area

D. The denture base area is large

E. The patient has not mastered the method of removing the denture

16.2004U4A1039 Causes of poor retention of removable partial dentures include, in addition to ( )

A. Abutment crowns are small

B. The base does not fit well with the tissue, and the edges are not well sealed

C. The snap ring does not fit the abutment tooth

D. Improper placement of artificial teeth

E. The base cushioning of the undercut area is insufficient

17.2003U4AN142 A patient has missing 5678 left and 678 right mandible, left lower 4 is not loose, no caries, and the alveolar ridge is full. Cast bracket denture lower left 4 lower right 5 distal middle occlusion support, three-arm card retainer, tongue rod large connector. After wearing the denture for 1 week, he complained of base tenderness and abutment bite pain. Intraoral examination found that there was a small ulcer at the base of the tongue frenulum, the mucosa of the left mandibular convex area was red and swollen, the lower left 4 percussion pain (+), the dentures were tightly sealed, and the bite was not high. The cause of ulceration at the base of the tongue-tie is that the tongue shaft is too low. The treatment of left mandibular bulge tenderness is ( )

A. Denture base edge grinding off

B. Buffering at the corresponding point of the tissue surface of the denture base

C. Relining of the denture base tissue surface

D. Adjust the occlusion

E. Adjust the retention force of the lower left 4 snap rings

18.2003U4AN146 A patient has missing 5678 in the right mandible, and the adhesive denture is repaired, with 4 three-arm cards in the lower right and 47 gaps in the lower left. One week after wearing the denture, he complained of tenderness at the free end of the denture and discomfort to chew. Intraoral examination found that all parts of the denture were tightly sealed, the retention and stability were good, there was no high point in the occlusion, the base extension did not affect the soft tissue activity, and there was no obvious tenderness, redness, swelling or ulceration of the missing mucosa. The cause of denture tenderness is ( )

A. Poor clamp ring retention force

B. Kito sinking

C. Pitch of the base

D. The alveolar ridge is overburdened

E. Improper Use

19.2011U4A1062 The oral examination that needs to be carried out before the start of the restoration is ( )

A. External oral examination

B. Examination in the mouth

C. Preparation of model checks

D. X-rays

E. All of the above tests are required

20.2014U4A2081 When designing the gap card to prepare the tooth, it cannot be prepared into a wedge, nor can it destroy the contact point of two adjacent teeth, the reason for this is ( )

A. Make the most of natural gaps

B. Prevent interabutment food impaction

C. Minimal tooth tissue is removed

D. Prevent decay of the abutments

E. Avoid the formation of a wedge force that displaces the abutment

21.2011U4A1063 What kind of impression must be trimmed when taking ( )

A. Anatomical impressions

B. Impressions of tooth-supported dentures

C. Impressions of mucosal support dentures

D. Impressions of hybrid support dentures

E. Individual pallet impressions

22.2004U4A1040 The purpose of taking a functional impression is ( )

A. Accurately reflects the position and morphology of the perioral muscles during functional activity

B. Evenly compress the oral tissues

C. Round the edge of the impression

D. See anatomical landmarks clearly

E. Margins and mucosal transitions are clear

23.2004U4A2091 A patient has 67 upper right maxillary remainder and 67 lower left lower right mandib, which method should be used in order to establish the correct occlusal relationship on the model ( )

A. Recording the occlusal relationship of the remaining teeth by wax biting

B. Record vertical distances with wax embankments

C. Record the median relationship with a wax embankment

D. Record the vertical distance with the median relationship with the wax embankment

E. Determine the vertical distance and median relationship directly with the model occlusion

24.2013U4AN125 When wearing the full denture for the first time, it was found that the lower total denture was warped left and right, and the patient felt pain during afterburner. Possible causes of warping are not included ( )

A. The kito is stretched too long

B. Inaccurate impressions

C. The base is not buffered into the undercut area

D. Kito deformation

E. The base tissue plane corresponding to the hard zone is not buffered

25.2006U4A1004 The purpose of the removable partial denture is that the width of the buccal and lingual diameter of the artificial posterior teeth is smaller than that of the natural tooth ( )

A. Improve chewing efficiency

B. Balance

C. Prevent cheek biting

D. Reduce the load on the support organization

E. Enhanced retention

26.2014U4A1032 According to the direction and degree of abutment tooth inclination, the wire can be divided into three types, the correct one is ( )

A. The wire drawn by the abutment tooth tilted to the gap side is the I. type wire

B. Ι-type wire is close to the occlusal surface on the near gap side of the abutment

C. The wire drawn by the abutment tooth tilted in the opposite direction of the gap is a type II wire

D. Type II wire is far from the occlusal surface on the distal gap side

E. When there is no obvious inverted indentation on the near and distal gap side of the abutment tooth or the abutment tooth is inclined to the mesiodistal and middle of the abutment, the wire drawn is a type III wire

27. 2010U4A1098 I. type observation line of the undercut is mainly in ( )

A. The proximal notch side of the abutment tooth is inverted very smallly

B. Distal notched side of the abutment, the proximal notch side is very small

C. There are large inverted concaves on the near and distal gap sides of the abutments

D. There is no large inverted depression on the near and distal gap sides of the abutment

E. Adjacent faces near the gap

28.2011U4A1064 Type II. The undercut area of the abutment of the lateral line is in ( )

A. Near the gap side

B. Distal gap side

C. Buccal side

D. Lingual

E. The inverted concave area of the near and far gap sides is large

29.2004U4A1041 Which of the following is NOT a component of a removable partial denture ( )

A. Retainers

B. Abutments

C. Artificial teeth

D. Kitto

E. Connectors

30.2004U4A1042 The width and length of the molar casting occlusal support are ( )

A. The width is 1/3 of the buccal tongue diameter and the length is 1/4 of the mesiodistal middle diameter

B. The width is 1/4 of the buccal-tongue diameter and the length is 1/4 of the mesiodistal middle diameter

C. The width is 1/4 of the buccal-tongue diameter and the length is 1/3 of the mesiodistal middle diameter

D. The width is 1/3 of the buccal-tongue diameter and the length is 1/3 of the mesiodistal middle diameter

E. The width is 1/2 of the buccal tongue diameter and the length is 1/2 of the mesiodistal diameter

31.2011U4A1065 Which of the following descriptions of occlusal support function is incorrect ( )

A. Prevent gingival displacement of dentures

B. Maintain the position of the snap ring on the abutment

C. Prevent food impaction

D. Prevent lateral displacement of dentures and occlusion

E. Restoration of occlusal relationship

32.2010U4A1100 The angle between the concave bottom of the occlusal support and the long axis of the abutment is ( )

A. 90°

B. 10°

C. 20°

D. 25°

E. 30°

33.2010U4A1100 The angle between the concave bottom of the occlusal support and the long axis of the abutment on the abutment is ( )

A. 20°

B. 50°

C. 60°

D. 75°

E. 90°

34.2010U4A1101 Which of the following dentition defects requires indirect retainer ( )

A. Anterior teeth are missing

B. Most posterior teeth are missing

C. Dissociative deletion at the end

D. Missing gaps

E. Most anterior teeth are missing

35.2008U4A1012 The function of the indirect retainer is ( )

A. Enhance the retention, stability and strength of the denture

B. Enhance the retention, stability and aesthetics of the denture

C. Enhance denture retention, support, and strength

D. Enhance denture retention, support, and stability

E. Enhance denture retention, support, and comfort

36.2011U4A1066 Indirect retainer not included ( )

A. Canine support

B. Interproximal hook of anterior teeth

C. Snap ring

D. Metal tongue plate

E. Metal palatal plate

37.2011U4A1067 The retention force of the buccal and lingual snap arm of the posterior teeth should be equal, mainly for ( )

A. Strengthen retention

B. Strengthen the role of enveloping

C. Prevent denture warping

D. Prevent dentures from wobbling

E. Prevent denture damage

38.2003U4AN143 A patient has missing 5678 left and 678 right in the lower jaw, and the lower left 4 is not loose and caries, and the alveolar ridge is full. Cast bracket denture lower left 4 lower right 5 distal middle occlusion support, three-arm card retainer, tongue rod large connector. After wearing the denture for 1 week, he complained of base tenderness and abutment bite pain. Intraoral examination found that there was a small ulcer at the base of the tongue frenulum, the mucosa of the left mandibular convex area was red and swollen, the lower left 4 percussion pain (+), the dentures were tightly sealed, and the bite was not high. In order to reduce the torsional force on the lower left 4, the following measures can be taken, except for ( )

A. Add indirect retainers

B. Use RPI snap rings instead

C. Use a pull-back ring instead

D. Reduction of free end bases

E. Reduction of the number and diameter of artificial teeth

39.2014U4A1033 The role of the snap ring body ( )

A. It plays a role in retaining the position and prevents the denture teeth from moving in the closed direction

B. Stabilizes and prevents the denture from moving laterally

C. Acts as a supporter to prevent the denture from moving in the gingival direction

D. Acts as retention and stabilization to prevent dentures from moving in the closed and lateral directions

E. Stabilizes and supports the lateral and gingival movement of the denture

40.2014U4A1034 Description of casting snap rings, inappropriate is ( )

A. The ring arm is wide and thick at the beginning and narrower towards the tip

B. The tip of the snap ring arm has the functions of retention, support and stability

C. There are three commonly used types of snap ring arms: round, semicircular and flat

D. The snap ring body is located in the non-chamfered area of the abutment

E. The tip of the snap ring arm is located in the abutment inverted area

41.2007U4A1009 The tip of the snap ring retained arm should be located in the abutment ( )

A. Contour high point line

B. Conformal high-point line occlusion

C. Shape of the high point line gingival square

D. Occlusal of the wire

E. Gingival side of the wire

42.2011U4A1068 Compared with cast snap rings, the characteristics of forging wire snap rings are ( )

A. It is not easy to adjust

B. Fit snugly with the tooth surface of the abutment

C. Good elasticity

D. Good interaction and confrontation

E. Good support

43.2008U4A1013 Half snap ring for ( )

A. Excessive teething

B. Abutments with poor crown shape

C. Distal solitary molars with cheek or tongue slanted

D. Isolated bicuspids, molars

E. Loose abutments

44.2000U4A2086 Male, 50 years old, 567 missing lower right, 6 missing lower left, 8 anterior lower right, poor contact with the opposite teeth, normal residual teeth, how to design the upper snap ring of the lower right 8 when designing a dental support denture ( )

A. Three-armed snap ring

B. Three-arm snap and enlarge the support to restore its occlusion

C. Ring snap ring

D. Ring the ring and enlarge the support to restore its occlusion

E. Barb the ring, and enlarge the support to restore its occlusion

45.2007U4A1011 is ( )

A. Gaps

B. Upper reverse snap ring

C. Ring snap ring

D. Three-armed snap ring

E. Pull-back snap ring

46.2008U4A1014 PULL BACK SNAP RING FOR ( )

A. Anterior teeth are missing

B. Absence of spacers

C. Posterior tooth free loss

D. Posterior teeth are not free and missing

E. Individual tooth loss

47.2008U4A1015 Pull Back Snap Ring( )

A. The tip of the arm is located in the inverted area of the labial and buccal surface of the abutment, bypassing the mesial surface of the abutment and connecting with the brace and the mesial lingual side of the abutment

B. The tip of the arm is located in the inverted area of the labial and buccal surface of the abutment, bypassing the mesial surface of the abutment and connecting with the connector on the distal lingual side

C. The tip of the arm is located in the inverted area of the labial and buccal surface of the abutment, bypassing the distal surface of the abutment and connecting with the brace, and connecting with the connector on the mesial lingual side

D. The tip of the arm is located in the inverted area of the labial and buccal surface of the abutment, bypassing the distal surface of the abutment and connecting with the brace and the distal lingual side of the abutment

E. The tip of the arm is located in the inverted area of the labial and buccal surface of the abutment, bypassing the mesial surface of the abutment and connecting with the brace, and connecting with the connector on the mesial midbuccal side

48.2008U4A1016 Pull back snap ring has stress interrupting effect ( )

A. Because the distal occidental support is connected to the base, the positive force is transmitted to the abutment through the support, which reduces the burden on the alveolar ridge

B. Because the distal occisidal support is connected to the base, the positive force is transmitted to the alveolar ridge through the artificial tooth and the base, reducing the burden on the abutment

C. Because the distal occlusal support is not connected to the base, the resultant force is transmitted to the abutment through the support, reducing the burden on the alveolar ridge

D. Because the distal occisidal support is not connected to the base, the positive force is transmitted to the alveolar ridge through the artificial tooth and the base, which reduces the burden on the abutment

E. Because the distal occlusal support is not connected to the base, the positive force is transmitted to the alveolar ridge through the artificial tooth and the base, reducing the burden on the alveolar ridge

49.2003U4A1021 What is effective in preventing food impaction is ( )

A. RPI snap ring

B. Pull back snap ring

C. Joint snap ring

D. Half snap rings

E. Rod snap rings

50.2010U4A1102 The snap ring with stress interrupting effect is ( )

A. Three-armed snap ring

B. Ring snap ring

C. Pull back snap ring

D. Half snap rings

E. Extension snap rings

51.2011U4A2091 Kennedy Class 1 dentition defects, when the hybrid support denture design is adopted, the main purpose of using the stress interrupting snap ring for the terminal abutment is ( )

A. Increase denture retention force

B. Enhanced denture stabilization

C. Facilitates a more uniform distribution of the load over the abutment and alveolar ridge

D. Prevent alveolar ridge mucosal tenderness

E. Reduce the burden on the alveolar ridge

52.2011U4A1069 PRI snap ring is composed of ( )

A. Distal occlusal support, mesial proximal panel, and lingual I-bar

B. Medial occlusal support, distal proximal panel, and buccal I-bar

C. Medial occlusal support, distal proximal panel, and buccal T-ring

D. Distal occissal support, mesial proximal panel, and lingual T-bar

E. None of the above

53.2007U4A1012 The main disadvantage of the rod ring and the ring ring is ( )

A. Poor retention

B. Poor stabilization

C. Poor support

D. Poor elasticity

E. Great damage to the abutment

54.2008U4A1017 Rod-shaped snap ring ( )

A. Good elasticity, small contact area with abutment teeth, weak push-type retention, less damage to abutment teeth, and better stability than annular snap rings

B. Good elasticity, small contact area with abutment, strong push-type retention, small damage to abutment, and better stability than annular snap ring

C. Good elasticity, small contact area with abutment, weak push-and-hang retention, little damage to abutment, and less stable effect than annular ring

D. Good elasticity, small contact area with the abutment, strong push-type retention, great damage to the abutment, and the stabilization effect is not as good as the annular snap ring

E. Good elasticity, small contact area with abutment, strong push-type retention, small damage to abutment, and less stable effect than annular ring

55.2008U4A1018 Comparison with distal occlusal support ( )

A. Medial occlusion reduces the torsional force on the abutments and reduces the burden on the alveolar ridge

B. Medial occlusion minimally reduces the torsional force on the abutments but increases the burden on the alveolar ridge

C. The mesial occlusion supports increase the torsional force on the abutments and also increase the burden on the alveolar ridge

D. Medial occlusion supports increase the torsional force on the abutments, but reduce the burden on the alveolar ridge

E. Medial occlusal support does not reduce the torsion on the abutments or the burden on the alveolar ridge

56.2008U4A1019 The main function of the RPI snap ring adjacent panel is ( )

A. Prevent the base from sinking

B. Reduce alveolar ridge stress

C. Aesthetics

D. Retention of enhanced dentures

E. Prevent food impaction

57.2003U4A2079 Patients with missing 5678 left and right mandibles. If the remaining alveolar ridge is full, 1/3 of the left and right lower 4 alveolar bones are resorbed. Left and right lower 4 retainers should be used ( )

A. Three-armed snap ring

B. Gaps

C. RPI snap ring

D. Extension snap ring

E. Ring snap ring

58.2000U4A1010 Type III snap ring (suitable for type 3 observation line) is characterized by ( )

A. Good retention and stabilization, medium support

B. Good retention, stability and support

C. Poor retention, stability and support

D. Good retention, good support, medium stability

E. Good stability and support, poor retention

59.2010U4A1103 Compared with the posterior palatal bar, the anterior palatal bar is characterized by ( )

A. Thick and wide, thickness about 1.5~2mm, width about 3.5mm

B. Thin and wide, thickness about 1~1.5mm, width about 3~3.5mm

C. Thin and wide, thickness about 1mm, width about 8mm

D. Thin and wide, thickness about 0.5mm, width about 5mm

E. Thick and wide, thickness about 2mm, width about 5mm

60.2008U4A1020 The relationship between the lateral palatal bar and the gingival margin is ( )

A. Contact with the gingival margin

B. Leave 1~3mm

C. Leave 4~6mm

D. Leave 7~10mm

E. Leave 11~15mm

61.2008U4A1021 The two ends of the posterior palatal bar are bent forward to ( )

A. Second pair of cuspids

B. Between the second bicuspid and the first molar

C. First molars

D. Between the first and second molars

E. Second molars

62.2008U4A1022 The posterior palate bar is located in ( )

A. Before the maxillary hard area

B. Maxillary hard area

C. After the maxillary hard area, before the flutter line

D. Vibrator line

E. After the flutter line

63.2008U4A1023 ANTERIOR PALATAL BAR SHOULD BE LOCATED ( )

A. Palatal folds

B. After the palatal fold, before the hard area of the upper palate

C. Hard area of the palate

D. After the hard area of the palate

E. Before the flutter line

64.2011U4A1070 The following are small connectors ( )

A. Palate bar

B. Tongue bar

C. Lip rod

D. Tongue plate

E. Booster Network

65.2008U4A1024 The tongue bar should be away from the gingival margin and should have a certain width. When using the tongue bar, the floor of the mouth should be deep enough, and the distance from the gingival margin of the lingual side of the lower anterior teeth to the floor of the mouth should be greater than ( )

A. 4mm

B. 8mm

C. 10mm

D. 15mm

E. 20mm

66.2011U4A2092 Left and right lower 56 missing, design tongue bar, alveolar ridge is inverted concave placement position is ( )

A. Gentle contact with mucous membranes

B. Depends on the type of denture design

C. Only 0.5 mm gap from the mucosa

D. Located in a non-inverted area

E. Located in the undercut area

67.2000U4A2087 Male, 55 years old, wore a mandibular brace removable partial denture for 3 days, and felt severe pain. Check: the lower right 67 and the lower left 67 can be removed partial dentures, the tongue rod is connected, the anterior alveolar ridge lingual side is sloped, and each part of the denture fits well with the tissue. Mucosal ulceration at the lower edge of the tongue shaft, which does not affect the soft tissue activity of the floor of the mouth. The cause of the pain is the tongue bar ( )

A. Compression of the mucosa

B. The edges are not smooth

C. Improper location

D. Inelasticity

E. Excessively thick

68.2008U4A1025 In which case, the tongue rod needs to leave the mucosa 0.3~0.4mm( )

A. The alveolar bone morphology on the lingual side of the mandibular is vertical and non-free missing

B. The alveolar bone morphology of the mandibular lingual side is vertical and free and absent

C. The alveolar bone morphology of the mandibular lingual side is sloped and not free missing

D. The alveolar bone morphology of the mandibular lingual side is sloped and free is missing

E. The alveolar bone morphology of the lingual side of the mandible is inverted

69.2010U4A1104 If the alveolar ridge on the lingual side of the mandible is sloped, the tongue bar should be ( )

A. Parallel to the mucosa, leave the mucosa a little or lightly touched

B. Parallel to the mucosa, without contact, 3~4mm away from the mucosa

C. Parallel to the mucous membranes and in close contact

D. Perpendicular to the mucous membrane and leave a little

E. None of the above

70.2000U4A1011 Removable partial denture base does not have the function of ( )

A. Bear and concentrate the tooth resultant force

B. Protect mucosa and alveolar bone

C. Connect the dentures into a single unit

D. Enhanced denture retention and stabilization

E. Repair of defective soft and hard tissues

71.2000U4A2088 Male, 70 years old. I wore a mandibular removable denture for half a year, and it broke off when I bit something yesterday. Check: lower right 24567 lower left 24567 mucosal support removable partial denture. 3 lingual bases folded longitudinally. The two broken ends are about 1.5mm thick, and the occlusal contact is good. The cause of the breakage of the base is ( )

A. The base is too thin

B. Biting hard food

C. Habitual unilateral chewing

D. Incorrect denture removal

E. Alveolar ridge resorption, the base is not well attached to the tissue

72.2011U4A1071 The thickness of the plastic base is generally ( )

A. 0.5mm

B. 1.0mm

C. 1.5mm

D. 2.0mm

E. 3.0mm

73.2011U4A1072 Which is wrong in the following description of the extension range of the tooth support RPD base ( )

A. Keep the base as small as possible

B. Posterior edge of the free end of the maxillary posterior teeth extended to the pterygomaxillary notch

C. Distal midbuccal aspect of the posterior edge of the maxillary jaw covers the maxillary tubercle

D. The edge protrudes into the inverted area of the tissue

E. The posterior edge of the mandible covers the front 1/2~2/3 of the posterior molars pad

74.2011U4A1073 The parts of the RPD base tissue surface that do not need to be buffered are ( )

A. Buccal side of maxillary tubercles

B. Maxillary hard area

C. Mandibular bulge

D. Post-molar pads

E. Medial oblique crest

75.2011U4A1074 The tip inclination of semi-anatomical teeth in removable partial dentures is ( )

A. 0°

B. 10°

C. 20°

D. 30°

E. 40°

76.2014U4A1035 The reason why hard plastic teeth or porcelain teeth should be chosen for artificial posterior teeth is ( )

A. Good anatomy

B. Easy to grind or adjust occlusion

C. It is not easy to fall off

D. High hardness and wear resistance

E. All of the above are true

77.2007U4A1013 The part of the removable partial denture that plays the role of connection, stability and retention is ( )

A. Retainers

B. Artificial teeth

C. Kitto

D. Large connectors

E. Small connectomes

78.2003U4A1022 In addition to the retention effect, the extension snap ring also has ( )

A. Stress interrupting

B. Prevent food impaction

C. Splint fixation

D. Anti-caries effect

E. Self-cleaning

79.2008U4A1026 Factors related to the functional restoration of removable partial dentures are ( )

A. Location and number of missing teeth

B. Abutment conditions

C. Mucosa and alveolar bone

D. Occlusal relationship of the remaining teeth

E. All of the above

80.2007U4A1014 Methods for reducing denture net force in removable partial dentures, excluding ( )

A. Reduce the buccal-lingual diameter of the artificial tooth

B. Reduced cusp inclination

C. Use plastic teeth

D. Reduce occlusal contact of artificial teeth

E. The number of artificial teeth can be reduced in free end denture restoration

81.2011U4A1075 Removable partial denture artificial posterior teeth occlusion cheek tongue diameter under what circumstances should the diameter of the cheek tongue be narrower than the natural tooth ( )

A. In any case

B. The opposite jaw is a natural tooth

C. Severe resorption of the maxilloalveolar ridge

D. There is more absorption of the alveolar ridge in the edentulous area

E. The number of missing teeth is low

82.2007U4A1015 The conditions that must be met by the removable partial denture retainer do not include ( )

A. No foreign body sensation

B. No corrective displacement of abutments

C. It is not easy to accumulate food

D. Avoid using different types of metals in the mouth

E. When taking and wearing, there is no lateral pressure on the abutment

83.2008U4A1027 The relationship between denture retention force and the depth of the ring arm entering the abutment indentation and the slope of the indentation ( )

A. Enter the inverted deep depth, the inverted concave slope is large, and the retention force is strong

B. Enter the inverted deep depth, the inverted concave slope is small, and the retention force is strong

C. Entering the inverted concave shallow, the inverted concave slope is large, and the retention force is strong

D. Entering the inverted concave shallow, the inverted concave slope is small, and the retention force is strong

E. The retention force and the depth of the undercut are independent of the slope

84.2000U4A1012 The measures to adjust the retention force of removable partial dentures are as follows, except ( )

A. Adjust the denture in place

B. Regulate the dispersion between the abutments

C. Increase or decrease the number of direct retainers

D. If you need to increase the transverse retention force, you should choose a casting snap ring

E. Adjust the depth and slope of the snap ring arm into the undercut area

85.2011U4A1076 Concave In-place Channel Refers to ( )

A. The angular bisector of the long axis extension of the abutment teeth on both sides is the in-place channel

B. By the inclination of the model, the undercut is concentrated on one side, and the direction of the tooth force is in place

C. The inverted concave is concentrated on one side by the inclination of the model, and the direction of the tooth force is not in the same direction

D. The positioning tract is consistent with the long axis of the abutment

E. The alignment of the tract and the direction of the tooth resultant force

86.2007U4A1016 In the principle of selecting removable partial denture abutments, the wrong one is ( )

A. Choose a healthy tooth for the abutment

B. Have a dental disease but have been treated

C. Periodontal disease is under control

D. The closer the tooth is to the gap for abutment retention, the better the support effect

E. When using multiple abutments, the more abutments and tighter the better

87.2014U4A1036 In order to eliminate the instability of removable partial dentures, the following method is wrong ( )

A. Increase the adversarial equilibrium retainer

B. Strive to design mucosal support dentures

C. Equilibrium is applied on the opposite side of the fulcrum or fulcrum line

D. Eliminate the fulcrum formed by occlusal support and snap ring on the remaining teeth

E. Eliminate the fulcrum formed by the base and the tissue

88.2007U4A1017 In order to eliminate the instability of removable partial dentures, the wrong method is ( )

A. Increase the adversarial equilibrium retainer

B. Try to design mucosal support dentures to avoid fulcrums

C. Equilibrium is applied on the opposite side of the fulcrum or fulcrum line

D. Eliminate the fulcrum formed by occlusal support and snap ring on the remaining teeth

E. Eliminate the fulcrum formed by the base and the tissue

**GPT replied**

1. C. The base enters the inverted. Because the base entry into the undercut is the correct design and will not make it difficult for the denture to be in place.

2. A. The impression material is not of good quality. Because the quality of the impression material does not directly affect the deformation of the stent, the deformation of the stent is mostly related to the design and production process.

3. E. The base does not fit into the mucosal tissue. The misfit of the base to the mucosal tissue will not directly lead to difficulty in removing and may lead to uncomfortable wearing.

4. B. Bilateral free deletions. Kennedy's first type of dentition defect is defined as the free loss of bilateral posterior teeth.

5. C. Category II, subcategory II. According to the number, the missing tooth is located on both sides, not the free end, and belongs to the second category, and there are missing teeth on both sides, which is the second subcategory.

6. B. Category I, Subcategory II. According to the number, the missing tooth is located on one side, which is the free end, and belongs to the first category, and there are multiple missing teeth, which is the second subcategory.

7. E. Category IV. According to the number, the missing teeth are located in the anterior area and belong to the Kennedy fourth category.

8. A. Category I. Bilateral free end deletions belong to the first category of the Kennedy classification.

9. B. Bilateral free end deletion. The first category of the Kennedy classification refers to the absence of free ends of bilateral posterior teeth.

10. D. Category II and subcategory III. According to the number, the missing tooth is not a free end and there are multiple missing teeth, which belongs to the second category, the third subcategory.

11. E. Category IV. According to the number, missing teeth include the anterior tooth area and belong to the Kennedy fourth category.

12. C. Category III. According to the number, the missing tooth is located on one side, not the free end, and belongs to the third category.

13. E. Category IV. According to the number, missing teeth include the anterior tooth area and belong to the Kennedy fourth category.

14. B. Straight. Because the lower right 5 and lower right 7 are healthy and the gap is normal, they can be designed as a straight fulcrum line.

15. E. The patient has not mastered the method of removing the denture. Because this is a problem that can be solved through patient education, not the denture itself.

16. E. Undercut base cushioning is insufficient. Because the undercut base is not cushioned enough, it will not directly lead to poor retention, but it will affect comfort.

17. A. Denture base edge grinding off. Because abrasion of the base edge can reduce the compression of the mucous membrane and relieve pain.

18. D. The alveolar ridge is overburdened. Because none of the other options directly cause tenderness, overburdening the alveolar ridge may cause tenderness.

19. E. All of the above tests are required. Before the restoration begins, a comprehensive oral examination is required, including external, internal, model, and X-ray examinations.

20. B. Prevent food impaction between the abutments. The gap card is designed not to break the contact points to prevent food impaction.

21. C. Impressions of mucosal support dentures. Mucosal support dentures require myofunctional trimming to ensure accurate impressions.

22. A. Accurately reflect the position and morphology of the perioral muscles during functional activity. The purpose of a functional impression is to reflect the true condition of the oral tissues during functional activity.

23. B. Record vertical distances with wax embankments. Because vertical distance is essential to establish the correct occlusal relationship.

24. D. Demomorphic Kitto. Because if the base is not deformed, all other options can cause warping and pain.

25. C. Prevent cheek biting. The width of the buccal and lingual diameter of the artificial back teeth is smaller than that of the natural teeth to prevent biting of the cheeks.

26. A. The wire drawn by the abutment tooth inclined to the gap side is the I. type wire. This is the type of wire defined according to the direction of inclination of the abutment.

27. A. The proximal notch side of the abutment tooth, the distal notch side is very inverted. The inverted characteristics of the I-type observation line.

28. B. Distal gap side. The inverted concave area of the type II. lateral line is mainly on the distal gap side.

29. B. Abutments. Abutments are natural teeth and are not components of removable partial dentures.

30. A. The width is 1/3 of the diameter of the buccal tongue and the length is 1/4 of the mesiodistal diameter. This is a regular design size to guarantee the effect of the support.

31. E. Restoration of occlusal relationship. The main function of the occlusal support is to prevent the gingival displacement of the denture and not to directly restore the occlusal relationship.

32. A. 90°。 The angle between the concave base of the occlusal support and the long axis of the abutment is designed at a right angle to ensure optimal support.

33. A. 20°。 The concave base of the occlusal support on the abutment is usually at a 20° angle to the long axis of the abutment to facilitate the adaptation and comfort of the abutment.

34. C. Dissociative deletion at the end. In the case of dissociative terminal loss, an indirect retainer is required to provide additional support to prevent the denture from warping.

35. D. Retention, support and stabilization of enhanced dentures. The role of indirect retainers is to enhance the overall retention, support and stability of the denture.

36. D. Metal tongue plate. The metal tongue is not an indirect retainer, it is a form of connector.

37. C. Prevent denture warping. The retention force of the buccal lingual snap arm of the posterior teeth should be equal, mainly to prevent the denture from warping.

38. E. Reduction of the number and diameter of artificial teeth. Reducing the number and size of artificial teeth does not directly reduce the torsion on the abutments.

39. D. Acts as a retention and stabilizer to prevent the denture from moving in the closed and lateral directions. The main function of the snap ring body is to retain and stabilize.

40. E. The tip of the snap ring arm is located in the abutment inverted area. The tip of the snap ring arm should not be located in the abutment inverted area, which will affect the retention and stability of the snap ring.

41. B. Shape of the high point line occlusion. The tip of the snap ring-retained arm should be located at the high point of the abutment tooth and the occlusal of the line.

42. C. Good elasticity. The forging wire ring is characterized by good elasticity, easy adjustment, and good fit with the abutment.

43. C. Distal solitary molars with cheek or tongue inclination. The halves are suitable for teeth that are distal and isolated and have a slope.

44. B. Three-arm snap ring and enlarge the support to restore its occlusion. This provides both retention and restoration of occlusion by enlarging the support.

45. E. Pull back snap ring. Type II snap rings are pull-back snap rings, which are suitable for inclined abutments.

46. C. Posterior tooth free loss. The pull-back ring is suitable for cases where the posterior teeth are free and missing, as it provides good retention.

47. A. The tip of the arm is located in the inverted area of the labial and buccal surface of the abutment, bypassing the mesial surface of the abutment and connecting with the connector on the mesial lingual side. This is the correct description of the pull-back snap ring.

48. C. Because the distal occisidal support is not connected to the base, the positive force is transmitted to the abutment through the support, which reduces the burden on the alveolar ridge. This is an explanation of the stress interrupting action.

49. E. Rod Ring. The design of the rod ring can effectively prevent food impaction.

50. C. Pull back snap ring. The snap ring that has a stress interrupting effect is the pull back snap ring.

51. C. Facilitates a more uniform distribution of the load over the abutment and alveolar ridges. The purpose of the stress interrupting ring is to distribute the load and protect the abutment and alveolar ridge.

52. A. Distal occlusal support, mesial proximal panel, and lingual I-bar. This is an integral part of the RPI snap ring.

53. A. Poor retention. Compared with the ring ring, the main disadvantage of the rod ring is the poor retention effect.

54. C. Good elasticity, small contact area with the abutment, weak push-and-hang retention, little damage to the abutment, and the stabilizing effect is not as good as the annular snap ring. The rod ring is characterized by good elasticity, but the stabilizing effect is not as good as that of the ring ring.

55. A. Medial occlusion minimizes the torsion on the abutments and reduces the burden on the alveolar ridge. The design of the mesial median occlusal support helps to reduce the burden on the abutment and alveolar ridge.

56. E. Prevention of food impaction. The main function of the RPI ring adjacent plate is to prevent food impaction.

57. C. RPI snap ring. For cases where the remaining alveolar ridge is full and 1/3 of the alveolar bone is resorpted, the RPI ring is a suitable choice.

58. A. Good retention and stabilization, medium support. Type III snap ring is suitable for type 3 observation line, which is characterized by good retention and stabilization, and medium support.

59. B. Thin and wide, thickness about 1~1.5mm, width about 3~3.5mm. The anterior palatal bar is usually thinner and wider than the posterior palatal bar.

60. B. Leave 1~3mm. The lateral palate bar is usually kept at a distance from the gingival margin so as not to compromise comfort and cleanliness.

61. B. Between the second bicuspid and the first molar. Cause: The posterior palatal bar design provides good support between the second bicuspid and the first molar.

62. C. After the maxillary hard area, before the flutter line. Causes: The posterior palate bar should be located behind the maxillary hard area and before the flutter line to avoid affecting tongue movement.

63. B. After the palatal fold, before the hard area of the upper palate. Causes: The anterior palatal bar should be positioned to avoid the palatal fold area to ensure comfort and stability.

64. D. Tongue plate. Cause: The tongue plate is a small connector that connects different parts of the denture.

65. B. 8mm。 Reason: To ensure adequate space and comfort, there should be at least 8mm of distance under the tongue bar.

66. E. is located in the undercut. Reason: The shape of the alveolar ridge should be considered in the design of the tongue bar to accommodate different inverted shapes.

67. C. Improper location. Causes: If the dentures fit well with the tissue, mucosal ulcers at the lower edge of the tongue may be caused by improper positioning of the tongue.

68. D. The alveolar bone morphology of the mandibular lingual side is sloped and absent. Reason: In this case, the tongue bar needs to have some clearance to accommodate the pressure and movement of the tissue.

69. A. Parallel to the mucosa, leave the mucosa a little or lightly touched. Causes: When the alveolar bone on the lingual side is sloped, the tongue bar should be parallel to the mucosa to avoid excessive pressure.

70. E. Repair of defective soft and hard tissues. Reason: The base of a removable partial denture is not used to repair hard and soft tissues, but to support and fix the denture.

71. A. The base is too thin. Cause: If the base is too thin, it may not be able to withstand normal chewing force and is prone to breaking.

72. C. 1.5mm。 Reason: The thickness of the plastic base is generally 1.5mm to ensure proper strength and comfort.

73. D. The edge protrudes into the inverted area of the tissue. Causes: The edge of the base should not protrude into the tissue inverted area to avoid unnecessary pressure on the tissue.

74. B. Maxillary hard area. Reason: The maxillary hard area is a relatively stable area and does not need to be buffered.

75. C. 20°。 Reason: The tip slope of semi-anatomical artificial teeth is usually 20° to mimic the chewing efficiency of natural teeth.

76. D. High hardness and wear resistance. Reason: Artificial posterior teeth should be made of hard plastic or porcelain teeth, as they are hard and wear-resistant.

77. D. Large connectors. Reason: Large connectors connect, stabilize and retain in removable partial dentures.

78. A. Stress interrupting. Reason: In addition to the retention effect, the extension snap ring also has a stress interrupting effect and disperses the chewing force.

79. E. All of the above. Causes: These factors are all related to the functional restoration of removable partial dentures.

80. C. Use plastic teeth. Reason: The choice of plastic teeth is not a way to reduce the joint force of denture teeth, but a matter of material selection.

81. C. Severe resorption of the maxilloalveolar ridge. Causes: When resorption of the maxilloalveolar ridge is severe, the buccal-lingual diameter of the artificial tooth should be narrower than that of the natural tooth to reduce pressure.

82. D. Avoid using different kinds of metals in the mouth. Reason: This condition has nothing to do with what the retainer must have.

83. B. Enter the inverted deep incessation, the inverted concave slope is small, and the retention force is strong. Reason: The depth and slope of the inverted abutment of the ring arm are important factors in determining the retention force.

84. E. Adjust the depth and slope of the snap ring arm into the undercut area. Reason: Adjusting the depth and slope of the snap ring arm into the undercut zone is one way to adjust the retention force and should not be ruled out.

85. B. By the inclination of the model, the undercut is concentrated on one side, in line with the direction of the tooth resultant force. Reason: The concave positioning channel is designed by the tilt of the model to align with the direction of the tooth resultant force.

86. E. When choosing multiple abutments, the more abutments and tighter the better. Reason: When choosing multiple abutments, it is not that the more abutments the better, but that the quality and location of the abutments should be considered.

87. B. Make every effort to design mucosal support dentures. Causes: Designing mucosal support dentures does not necessarily eliminate instability and may cause other problems.

88. B. Whenever possible to design mucosal support dentures to avoid the formation of fulcrums. Reason: As above, this design does not necessarily eliminate instability and may cause other problems.

**Chapter 7 Implant Dentures**

1.2011U4A1008 Which of the following does not generally cause fracture of the base of the full denture ( )

A. Imbalance of tooth resultant force

B. The base is thinner

C. Alveolar bone continues resorption

D. The base does not fit the mucosa

E. Insufficient recovery of vertical distance

2.2010U4A1031 The following situations require denture relining, except for ( )

A. The denture does not fit well when it is first worn and needs to be relined when it is not properly retained

B. Poor retention of full dentures due to alveolar ridge resorption after a period of wear

C. Broken denture resulting in malfitting after repair

D. Patients with a bladed alveolar ridge with generalized tenderness after full denture restoration

E. Wrong jaw position

3.2011U4A1009 According to the characteristics of edentulous jaw tissue, the zygomatic process belongs to ( )

A. Main support area

B. Sub-support area

C. Buffers

D. Rear embankment area

E. Marginal Closure Zone

4.2011U4A1010 The area where the edge of the denture can not be stretched more is ( )

A. Alveolar ridge

B. Distal mid-buccal angle region

C. Buccal flange area

D. Maxillary tubercles

E. zygomatic process

5.2004U4A1003 The incisor mastoids are an anatomical landmark of the aligned upper central incisors because ( )

A. The incisor mastoid process is located at the anterior end of the maxillary midpalatal suture

B. There is a more stable relationship between the incisor mastoid process and the maxillary central incisors

C. The incisor hole is below the incisor mastoid process, and it is necessary to prevent compression here when arranging the teeth

D. Less variation in the position of the incisor mastoid process

E. The boundary line of the two upper central incisors should be based on the incisor mastoid

6.2004U4A1004 The function of the incisor mastoids is ( )

A. Reference markers for aligning maxillary central incisors

B. Determine the landmarks of the occlusal plane

C. Signs to determine the extent of the base extension

D. Determine the landmarks of the alveolar crest

E. Identification of the rear embankment area

7.2010U4A1032 The purpose of simulating palatal fold in the anterior palatal area of maxillary base is ( )

A. Fabrication of artificial dentures

B. Aesthetics

C. Helps with pronunciation

D. Pleasant taste

E. Increase base strength

8. 2004U4A1005 Anatomical landmarks unrelated to the posterior edge of a full denture are ( )

A. Palate fovea

B. Palatal lobe

C. Posterior fibrillation line

D. Pterygomaxillary notch

E. Post-molar pads

9.2004U4A1006 Palate fovea located in ( )

A. Anterior incisor mastoid process

B. Between the palatal fold and the incisor mastoid process

C. Posterior palatal fold

D. Posterior edge of the maxillary alveolar ridge

E. Junction of hard and soft palate, flanked by midpalatal suture

10.2004U4A1007 The role of the rear embankment area is ( )

A. Positioning of the trailing edge of the base

B. Edge closure

C. Supporting role

D. Tooth Row Marker

E. Buffering

11.2004U4A1008 Maxillary denture posterior embankment area is located in ( )

A. Post-molar pads

B. Anterior palatal fovea

C. Before the anterior fibrillation line

D. Between anterior and posterior flutter lines

E. After the posterior fibrillation line

12.2007U4A1002 The area between the alveolar ridge and the hard area of the maxillary common denture is ( )

A. Main support area

B. Sub-support area

C. Buffers

D. Edge stretch area

E. Marginal Closure Zone

13.2010U4A1033 Which of the following descriptions of edentulous sub-support areas is incorrect ( )

A. Sub-supporting area refers to the labial, buccal, and lingual sides of the maxillary alveolar ridge (excluding the hard area)

B. There is no clear boundary between the secondary and main support areas

C. The support of the sub-supporting area is poor and cannot withstand large chewing pressure

D. There are mucosa, submucosa, and muscle attachment points on the bone surface in this area

E. The mucosa in this area is closely attached to the bone surface, and the mucosa is stable and immovable, which is conducive to denture retention

14.2004U4A1009 According to the characteristics of the tissue structure of each part of edentulous jaw and its relationship with full dentures, edentulous jaw is divided into the following four areas, except for ( )

A. Main support area

B. Sub-support area

C. Marginal Closure Zone

D. Rear embankment area

E. Buffers

15.2004U4A1011 belongs to the edge of the closed area ( )

A. Maxillary alveolar crest

B. Post-molar pads

C. Incisor mastoids

D. Buccal vestibule

E. Mandibular carina

16.2004U4A1012 Belongs to buffer ( )

A. Maxillary alveolar crest

B. Post-molar pads

C. Incisor mastoids

D. Buccal vestibule

E. Buccal frenulum

17.2011U4A1011 According to the characteristics of edentulous jaw tissue structure, the mandibular hyoid crest belongs to ( )

A. Main support area

B. Sub-support area

C. Marginal Closure Zone

D. Buffers

E. Embankment area

18.2011U4A1012 Which of the following is NOT the purpose of the base as a buffer ( )

A. Prevent tenderness

B. Prevent pronunciation from being impaired

C. Prevent compression of gingival tissue

D. Prevent crushing of mucosal tissue

E. Prevent the base from warping

19.2004U4A1013 The part that belongs to the buffer zone is ( )

A. Incisor mastoid area

B. Posterior molar cushion area

C. Embankment area

D. Alveolar crest region

E. Buccal flange region

20.2004U4A1014 The surface most closely related to the action of the labial and buccal muscles of the full denture is ( )

A. Tooth plane

B. Polished surface

C. Occlusal surface

D. Organizational surface

E. Adsorption surface

21.2010U4A2129 When the full denture is first worn, if there is an open anterior tooth, the possible causes are, except for ( )

A. Error in intraoral jaw removal

B. Error in the process of transferring the jaw position

C. Early contact in the distal middle of the maxillary base

D. High occlusion in the posterior region

E. Patient will not use dentures

22.2011U4A1013 The lower dental arch is found to be significantly receding when the full denture is first worn, and the reason may be ( )

A. Improper alignment of artificial teeth

B. The patient's mandible is too receding

C. Occlusion moves back and forth

D. Extension of the lower jaw when determining the horizontal jaw position

E. The vertical distance is too low

23.2010U4A2130 When the denture is first worn, the reason for the need to grind and adjust the occlusion, except for ( )

A. Any occlusion does not fully correspond to the patient's actual situation

B. Improper operation during the denture making process

C. The shape of the artificial tooth cusp is not standard

D. A simple occlusal frame was used for the fabrication

E. The vertical distance is usually predetermined to be higher when taking the jaw relationship

24.2010U4A2131 Full denture first wearing, lateral occlusion and grinding, usually should be adjusted ( )

A. Upper canine

B. Upper anterior teeth

C. Inferior canine

D. Lower anterior teeth

E. Upper posterior teeth

25. 2010U4A1034 When the full denture is first worn, the wrong way to select and grind is ( )

A. In the case of early contact between the median occlusion, the mesiodistal marginal crest and fossa corresponding to the early contact support tip are mainly selected

B. When selecting and grinding lateral occlusal interference, select and grind a small number of occlusal interference points on the non-supporting cusp

C. When the lateral occlusion interference is selected, if it is a canine, the interference is usually dominated by the upper canine

D. When the anterior occlusion does not interfere, such as when the anterior teeth are in contact but the posterior teeth are not in contact, the lower anterior lip bevel is mainly selected

E. When the anterior occlusion is not disturbed, such as when the posterior teeth are in contact but the anterior teeth are not, the distal oblique plane of the upper cusp or the mesial oblique plane of the lower cusp should be grinded

26.2011U4A1014 The median occlusion is found to be normal when the full denture is first worn, and the anterior occlusion and anterior teeth should be sharpened ( )

A. Upper incisor incisor tongue bevel

B. Upper incisor incisal lip bevel

C. Upper incisor lingual fossa

D. Incisor margin of the lower incisor

E. Lower incisor tongue bevel

27.2010U4A1035 When the full denture is first worn, the wrong thing in the patient's medical order is ( )

A. Initial foreign body sensation, nausea, and slurred pronunciation, but gradually improves

B. Dentures should be cleaned after each meal

C. Do not remove your dentures while sleeping

D. If you have pain, you should go to the hospital for follow-up in time

E. Soak in cold water when not wearing

28.2010U4A1036 Full dentures are difficult to place for ( )

A. The inverted indentation is too large

B. The vertical distance is too high

C. The edge of the base is too long

D. Small nodules on the tissue plane

E. None of the above

29.2004U4A1015 The base tissue surface needs to be partially buffered is ( )

A. Misplaced relationship

B. Poor denture retention

C. Poor occlusion function of the anterior teeth

D. Tenderness at the mandibular carina

E. Denture loss when speaking and opening widely

30.2004U4A1016 When the denture is first worn, the wrong thing in the doctor's order given to the patient is ( )

A. If you have a foreign body sensation or slurred pronunciation, you should wear it consistently

B. Eat small pieces of soft food first

C. Avoid biting hard objects

D. There may be pain, so keep wearing it

E. Try to chew with both back teeth at the same time

31.2010U4A1037 Causes of pain in dentures at the first time, except ( )

A. Small nodules in the tissue plane

B. Early contact with individual teeth

C. Impression deformation

D. Plaster model damage

E. The vertical distance is too short

32.2004U4AN124 A patient who wore a full denture for one week complained of laborious chewing, mucosal tenderness, and facial soreness. The solution is ( )

A. Wear it consistently and get used to it gradually

B. Occlusion

C. Base tissue surface buffering

D. Reproduction

E. Relining

33.2003U4AN133 Patients complained that the dentures were easy to loosen after wearing full dentures for 1 week. When taking a medical history, it is important to ask about ( )

A. How to loosen

B. How loose it is

C. When the denture is loose

D. Have you worn dentures in the past

E. Ability to eat

34.2003U4AN137 Patients complained that the dentures were easy to loosen after wearing full dentures for 1 week. When to reliner ( )

A. Unstable dentures

B. Warping of the denture on both sides

C. Unbalanced bite of dentures

D. Poor or poor attachment of denture edges

E. Insufficient cushioning of the maxillary carina

35.2003U4AN134 The patient complained that the denture was easy to loosen after wearing the full denture for 1 week. Check the diagnostics are ( )

A. Check that the bite is stable

B. Let the patient eat something first

C. Check the retention force first

D. Start by opening the patient's mouth wide

E. Make the patient yawn first

36.2003U4AN147 A patient has missing 5678 in the right mandible, and the adhesive denture is repaired, with 4 three-arm cards in the lower right and 47 gaps in the lower left. One week after wearing the denture, he complained of tenderness at the free end of the denture and discomfort to chew. Intraoral examination found that all parts of the denture were tightly sealed, the retention and stability were good, there was no high point in the occlusion, the base extension did not affect the soft tissue activity, and there was no obvious tenderness, redness, swelling or ulceration of the missing mucosa. The cause of denture tenderness is the overloading of the alveolar ridge. Solution( )

A. The kito tissue is heavily lined

B. Diameter reduction or reduction of artificial teeth

C. Add snap rings and supports in the lower left 6

D. A, B and C

E. B and C

37.2003U4AN140 A 59-year-old male patient with missing upper dentition, healthy lower right 8, mesial lingual slope, full alveolar ridge, maxillary scattered at the bony apex, and normal intermaxillary distance. The most closely related factor to denture retention is whether the impression is accurate. After wearing a denture for a week, it is necessary to have a follow-up consultation to adjust the occlusion, and the reason for the denture is sinking. The patient reported nausea from wearing teeth at the follow-up visit, what could be the reason ( )

A. Early posterior tooth contact

B. The posterior margin of the maxillary is too long

C. Poor denture retention

D. Mandibular posterior teeth are aligned too far to the lingual side

E. Yes

38.2013U4A1003 What can cause nausea and increased saliva after wearing maxillary dentures is ( )

A. The posterior edge of the denture base is under-sealed

B. At the buccal frenulum, the base is not cushioned enough

C. Poor shape of the polished surface

D. Posterior teeth aligned on the hemibuccal side

E. The posterior edge of the denture base is too short

39.2003U4AN135 Patients complain that the dentures are easy to loosen after wearing full dentures for 1 week. If the patient states that the denture is easy to fall off only when yawning, what should be checked except ( )

A. Denture edge extension

B. Denture polished surface morphology

C. Suitability of the rear embankment area

D. Whether the straps are out of the way

E. Stability of the denture

40.2003U4AN136 Patients complained that the dentures were easy to loosen after wearing full dentures for 1 week. If the patient states that the denture is prone to fall out only when eating, what should be the focus of the check-up ( )

A. Check the balance occlusion of the dentures

B. Check the base edge extension

C. Check the arrangement of the artificial teeth

D. Check how the embankment area is

E. Check that the lacing is out of the way

41.2003U4AN128 A 65-year-old female with most teeth missing in the whole mouth, only the upper left 78 remained, and the looseness was III.°, the gingiva was red and swollen, the alveolar ridge was of moderate height, and the intermaxillary distance was normal. The work that should be done before repair is to remove the upper left 78. The proposed restoration method is full denture restoration. If the correct method is followed, the impression is made in the following way: ( )

A. Functional impressions were taken on both the upper and lower jaws

B. Anatomical impressions were taken on both the upper and lower jaws

C. Take a functional impression of the upper jaw and an anatomical impression of the lower jaw

D. Anatomical impressions were taken on the upper jaw and functional impressions were taken on the lower jaw

E. Either of the above

42.2004U4AN123 A patient who wore a full denture for one week complained of frequent bite of the left back tooth and no other discomfort. The most likely reason is ( )

A. Left posterior tooth coverage is too large

B. Left posterior tooth coverage is too small

C. Left posterior occlusion interference on the working side

D. Left posterior tooth balance side occlusion interference

E. Interference of occlusion on the balance side of the right posterior teeth

43.2011U4A1015 Cheek biting phenomenon with full dentures, need to be sharpened ( )

A. Upper posterior teeth lingual apical lingual bevel and lower posterior teeth lingual apical buccal bevel

B. Upper posterior teeth lingual apical buccal bevel and lower posterior lingual apical lingual bevel

C. Upper posterior buccal bevel and lower posterior buccal bevel

D. Upper posterior buccal aperical tongue bevel and lower posterior blucial acupuncture bevel

E. Upper posterior blucial aical lingual bevel and lower posterior pacual apical buccal bevel

44.2003U4AN141 A 59-year-old male patient with missing upper dentition, healthy lower right 8, mesial lingual slope, full alveolar ridge, maxillary scattered at the bony apex, and normal intermaxillary distance. The most closely related factor to denture retention is whether the impression is accurate. After wearing a denture for a week, it is necessary to have a follow-up consultation to adjust the occlusion, and the reason for the denture is sinking. The patient reported nausea from tooth wearing at the follow-up visit, which may be the cause of the long posterior edge of the maxillary. The patient bites his lip and cheek while eating due to ( )

A. Insufficient extension of the denture margin

B. The vertical distance is too high

C. The median relationship is incorrect

D. The arrangement of artificial teeth covers too little

E. All of the above are true

45.2004U4A2078 A 65-year-old female patient underwent full denture restoration after extraction due to periodontal disease 3 years ago, and the effect was satisfactory. Recently, he had pain in his bite. Possible causes of pain ( )

A. The alveolar ridge has a bony tip

B. Excessive bite force

C. The edge of the base is too long after alveolar ridge resorption

D. Inflammation of the mucous membranes

E. Unbalanced occlusion

46.2004U4A1017 The retention of full dentures refers to ( )

A. Dentures do not fall out when chewing

B. The denture does not fall out when the mouth is wide open

C. The denture does not fall out when speaking

D. The denture does not fall out when the tongue is raised

E. Resistance when removing dentures from the mouth

47.2007U4A1003 In the restoration of total dentures, the force acting between saliva and the base should be called ( )

A. Adhesive strength

B. Attractiveness

C. Adhesion

D. Adhesion

E. Adhesion

48.2011U4A1016 Full denture retention mainly depends on ( )

A. Adhesion

B. Adhesion

C. Cohesion

D. Tongue and buccal muscle pressure

E. Adsorption

49.2014U4A1018 The factors most closely related to adsorption are ( )

A. The degree of tightness of the base tissue surface to the mucosa

B. Whether or not you have worn an old denture

C. Amount of saliva

D. Whether or not to use soft lining materials

E. Thickness of the base

50.2000U4A2080 A patient, female, 82 years old, with missing dentition, alveolar ridge stenosis, and low chewing efficiency after full denture restoration, the reason for which could not be ( )

A. Being too old

B. Alveolar ridge narrowing with poor retention

C. The vertical distance is too low

D. Fewer occlusal contact points

E. The artificial tooth model is too small

51.2011U4A1017 Factors not related to atmospheric pressure are ( )

A. Contact area of the base with the mucosa

B. Tightness of the base to the mucosa

C. Denture edge closure effect

D. Polishing degree of polishing surface of the base

E. All of the above are true

52.2014U4A1019 What has nothing to do with full denture retention is ( )

A. Base area

B. Alveolar ridge morphology

C. Mucosal properties

D. Saliva properties

E. Occlusal balance

53.2010U4A1038 Factors related to full denture retentionWhich of the following is false ( )

A. Anatomical morphology of the jaw

B. Nature of the oral mucosa

C. Alveolar bone density

D. Stretching of the base

E. Quality and quantity of saliva

54.2004U4A1018 The mucosal condition that is favorable for the retention and support of the full denture is ( )

A. Thick and fluffy

B. Thick and tough

C. Thin

D. Thin and dry

E. B or D

55.2013U4AN124 When wearing the full denture for the first time, it was found that the maxillary common denture was tilted from side to side, and the patient felt pain during afterburner. Among the following causes of denture warpage, the first reason to consider should be ( )

A. The kito is stretched too long

B. Kito deformation

C. Inaccurate impressions

D. Inaccurate model

E. The base tissue plane corresponding to the hard zone is not buffered

56.2010U4A1039 Full denture suitable concave polished surface form can be ( )

A. Reduced chewing efficiency

B. Make the pronunciation clear

C. Help with denture retention

D. Avoid biting your cheeks and tongue

E. Increases facial fullness

57.2011U4A1018 Edentulous oral special examination content does not include ( )

A. Size, morphology, and location of the maxillary arches and alveolar ridges

B. Alveolar ridge resorption

C. Examination of the oral mucosa

D. The size, shape, and functional status of the tongue

E. Saliva pH

58.2011U4A1019 Surgical excision is generally not advocated before repair ( )

A. Sharp bony protrusions, bony apex, and bony ridges

B. Formation of a large inverted mandibular carina underneath

C. The labial and buccal folds are too shallow

D. Laborobuccal frenulum attachment close to the alveolar ridge

E. Soft alveolar ridge

59.2004U4A1019 Surgical excision is generally advocated before repair ( )

A. Inverted maxillary tubercles

B. Sharp bony apex, bony procession, and bony ridge

C. Hyperplasia of mucosal tissue

D. Lalabial and buccal frenulum attachment points close to the alveolar ridge

E. All of the above

60.2010U4A1040 Alveolar bone trimming is not included in the parts that are commonly required before denture restoration.

A. Maxillary nodules

B. Maxillary labial side

C. Post-molar pads

D. Lingual side of mandibular bicuspids

E. Extraction wound site

61.2004U4A2079 A patient with edentulous jaw had a normal relationship between the upper and lower mandibular arches, a plump alveolar ridge, and a sharp bone spur on the labial side of the alveolar ridge at the upper jaw equivalent to the left canine position. This patient will need to undergo ( )

A. Deepening of labial-buccal folds

B. Maxillary tubercle trimming

C. Alveolar bone trimming

D. Lip tie trimming

E. Buccal frenulum trimming

62.2011U4A1020 The method of increasing the relative height of the alveolar ridge is ( )

A. Lip-tongue frenulum correction

B. Alveolar ridge repair

C. Bony carina

D. Vestibular sulcus deepening

E. Alveolar ridge reconstruction

63.2003U4AN138 A 59-year-old male patient with missing upper dentition, healthy lower right 8, mesial lingual slanting, full alveolar ridge, maxillary scattered at the bony apex, and normal intermaxillary distance. The factors most closely related to denture retention are ( )

A. Accuracy of the impression

B. Whether the jaw position is recorded correctly

C. Whether the position of the tooth row is correct

D. Whether the edge of the base is stretched correctly

E. Whether the tooth selection is suitable

64.2004U4A1020 What does not meet the requirements of edentulous impressions is ( )

A. The impression is detailed and clear

B. Stretch the edges as far as possible

C. The edges should be round and blunt, with a certain thickness

D. Tissue compression is uniform

E. Take a functional impression

65.2011U4A1021 Which is wrong about the full denture model description ( )

A. Shows signs of muscle function trimming

B. The width of the edge of the model should be 3~5mm

C. The thinnest part of the model should not be less than 5mm

D. The posterior edge of the model should be no less than 2 mm behind the palatal fovea

E. Mandibular model with posterior molars pad not less than 10 mm from the anterior edge

66.2007U4A1004 When selecting an antulous maxillary tray, its trailing edge length should be ( )

A. On the line between the pterygomaxillary notch and the palatal fovea

B. 0.5 mm above the flutter line

C. 1 mm above the flutter line

D. Flush with the fibrillation line

E. 3~4mm over the flutter line

67.2006U4A1001 When recording the maxillary position relationship of full dentures, the erroneous statement about occlusion is ( )

A. The occlusal tray is composed of a base and a wax bank

B. There are two types of base, temporary base and Henderson base

C. A base made of base wax tablets is called a temporary base

D. A base made of self-coagulation plastic is called a henderson

E. Temporary base is finally replaced by thermoformed plastic

68.2004U4A1021 Clinically used to record the relationship between the jaw ( )

A. Kitto

B. Temporary Basis

C. Occlusal embankment

D. Occlusal brace

E. Wax base

69.2010U4A1041 Determination of the maxillary relationship of full dentures refers to ( )

A. Restore the appropriate height of the lower 1/3 of the face and the physiological posterior position of the condyles on both sides

B. Restore facial physiology

C. Determine the correct intermaxillary distance

D. Correction of habitual jaw misalignment

E. Determine the vertical distance of the face

70.2014U4A1020 Determining the jaw relationship refers to ( )

A. Restore facial physiology

B. Restore the appropriate vertical distance of the face

C. Determine the correct intermaxillary distance

D. Restore the physiological posterior position of the condyle and the lower 1/3 height of the face

E. Correction of habitual protrusion of the jaw

71.2004U4A1022 When making the rear embankment area on a plaster cast, the width at the widest point is ( )

A. 1mm

B. 3mm

C. 5mm

D. 8mm

E. 10mm

72.2011U4A1022 The main purpose of using occlusion in the production of full dentures is ( )

A. Easy tooth arrangement

B. Restore vertical distance

C. Maxillary recording

D. Restore facial shape

E. Determine the occlusal plane

73.2010U4A1042 Which of the following descriptions of the occlusal surface of the full crown is wrong ( )

A. The original occlusal surface size and shape must be restored

B. It should be in harmony with the morphology of the adjacent occlusal surface

C. It should be in harmony with the morphology of the occlusal surface

D. The direction of the positive force is close to the long axis of the tooth

E. No early contact

74.2004U4A1023 The occlusal embankment plane of the posterior area should be parallel to ( )

A. Upper lip

B. Pupil connection

C. Bottom of the nose

D. Alar tragus line

E. Orbital auricular plane

75.2004U4A1024 The relationship between the embankment plane and the lower edge of the upper lip is ( )

A. 2 mm above the lip

B. Lip level

C. 2 mm below the lip

D. 3 mm below the lip

E. 4 mm below the lip

76.2014U4A1021 Occlusal vertical distance refers to ( )

A. The distance between the pupil line and the ostom

B. The distance from the base of the nose to the base of the chin when the upper and lower teeth of the natural dentition come into contact

C. The distance between the maxillary and maxillary alveolar crests

D. The distance from the base of the nose to the base of the mind when the natural dentition is in the midmaxillary position

E. Distance between the upper and lower jaws of edentulous jaws

77.2004U4A1025 The distance between the alveolar crest of the upper and lower mandibular crest when the lower jaw is in the median position in edentulous patients is called ( )

A. A. Attancy

B. Occlusal space

C. Vertical distance

D. Intermaxillary distance

E. Overbite

78.2011U4A1023 The method of determining the vertical distance by using the jaw position is ( )

A. The distance from the base of the nose to the base of the chin is measured in the jaw position, which is the vertical distance

B. When measuring the jaw position, the distance from the bottom of the nose to the base of the chin is subtracted by 2~3mm, which is the vertical distance

C. The distance from the corner of the mouth to the base of the chin is the vertical distance when measuring the rest and jaw position

D. The distance from the corner of the mouth to the base of the chin is subtracted by 2~3mm when measuring the jaw position, which is the vertical distance

E. None of the above methods are correct

**GPT replied**

1. E。 Insufficient vertical distance recovery generally does not directly lead to a fracture of the base of a full denture, but may lead to occlusal problems.

2. E。 Errors in jaw alignment require realignment rather than relining.

3. B。 The zygomatic process acts as a sub-supporting area, which is not the area that mainly bears masticatory force.

4. E。 The zygomatic process is the sub-support area, and the edge of the denture should not be stretched too much in this area, so as not to affect comfort and stability.

5. B。 There is a stable relationship between the incisor mastoid process and the maxillary central incisors, which provides an important anatomical landmark for the alignment of the upper central incisors.

6. A。 The incisor mastoids are the reference landmarks for aligning the maxillary central incisors.

7. C。 Simulated palate puckers are mainly to aid in pronunciation, as they can affect the position and movement of the tongue.

8. E。 The posterior molar pad is not related to the posterior edge of the full denture and is located in the lower jaw rather than the upper jaw.

9. E。 The palatal fovea is located at the junction of the hard and soft palate, on either side of the middle palatal suture.

10. B。 The posterior embankment area has an edge closure effect to help prevent the rear end of the denture from upturning.

11. D。 The posterior embankment area of the maxillary denture is located between the anterior and posterior flutter lines.

12. B。 The area between the alveolar ridge and the hard area of the maxillary common denture is the sub-supporting area.

13. E。 The mucosa in the sub-support area does not always fit tightly to the bone surface, and the mucosa may move, which is not conducive to denture retention.

14. E。 The buffer zone is not an area that is separated according to the structural characteristics of the edentulous jaw parts and their relationship to the full denture.

15. D。 The buccal vestibule belongs to the marginal occlusion.

16. E。 Buccal frenulum belongs to the buffer zone.

17. B。 The mandibular hyoid crest belongs to the sub-supporting area.

18. E。 Preventing the base from warping is not the purpose of the base buffer, which is mainly to protect the tissue.

19. E。 The buccal flange area is a buffer zone.

20. B。 The polished surface is most closely related to the action of the labial and buccal muscles.

21. E。 Patients who do not use dentures generally do not cause the anterior teeth to open and close.

22. D。 When determining the horizontal jaw position, mandibular protrusion may result in significant receding of the lower arch at initial wear.

23. E。 The vertical distance is usually predetermined to be higher when the jaw position is taken and this is not the reason for the selection of the occlusion.

24. C。 Lateral occlusion is ground and the lower canines should usually be adjusted.

25. E。 When the anterior occlusion does not interfere, such as when the posterior teeth are in contact but the anterior teeth are not, the posterior molars should be chosen instead of the anterior teeth.

26. B。 Early contact between the anterior teeth should be adjusted to the upper incisor incisor lip bevel.

27. C。 It is a mistake not to remove the denture while sleeping, as it helps the oral mucosa to rest and recover at night.

28. E。 The above options A, B, C, and D are all possible causes of difficulty in placing the denture, so E is correct.

29. D。 Local cushioning is required on the base tissue surface primarily to prevent tenderness, such as tenderness at the mandibular carina.

30. D。 If there is pain, you should not insist on wearing it, but should consult a doctor.

31. C。 Impression distortion usually does not directly cause pain when wearing a denture for the first time, while other options can be a direct cause of pain.

32. E。 If chewing effort and mucosal tenderness occur after wearing a full denture for a week, it may be that the basal tissue surface is not adapted to the mucosa and needs to be relined to improve the fit.

33. D。 Asking if they have worn dentures in the past can help understand how well the patient is comfortable with the dentures and possible retention issues.

34. D。 If the edges of the denture are poorly closed or do not fit snugly, it may result in the denture loosening easily and need to be relined to improve the fit.

35. C。 Checking the retention force is the first step in determining the cause of denture loosening, and poor retention force may be the main cause of denture loosening.

36. A。 Relining the basal tissue surface relieves tenderness by reducing the burden on the alveolar ridge.

37. B。 An excessively long posterior edge of the maxillary may cause nausea in the patient, as this may irritate the soft palate or pharynx.

38. E。 A short posterior edge of the denture base may cause nausea and increased salivation, as it may interfere with normal sensation in the pharynx.

39. E。 If the denture is prone to falling off only when yawning, it has little to do with the stability of the denture, as it is a dynamic process, while the other options are all related to retention at static.

40. A。 If the patient states that the denture is prone to loss only when eating, it should be important to check for occlusion, as an unbalanced bite is a common cause of loose dentures during eating.

41. B。 Given the patient's moderate alveolar ridge height and normal intermaxillary distance, an anatomical impression may be more appropriate because there is no tooth interference.

42. A。 Overcovering the left back teeth too much may lead to a bite on the cheek, as the over-covered teeth can touch the cheeks while chewing.

43. B。 Cheek biting is usually due to improper run-in between the upper posterior teeth and the lower posterior lingual bevel and requires abrasion to reduce bite.

44. E。 If a patient experiences lip and cheek biting while eating, all of these factors could be the cause.

45. E。 Occlusal imbalance is a common cause of biting pain because it causes local pressure points.

46. E。 Retention refers to the stability of the denture in the mouth, including not falling out under various movements, as well as proper resistance when removed.

47. E。 Adhesion refers to the force between saliva and the base, which helps to keep the denture in place.

48. A。 Full dentures are retained primarily by adhesion, which is due to the force generated by saliva on the thin film formed between the denture base and the mucous membrane.

49. A。 The adsorption force is closely related to the degree of adhesion between the base tissue surface and the mucosa, because a good fit can enhance the adsorption.

50. A。 Age is not a direct cause of low chewing efficiency, and other options can affect chewing efficiency.

51. D。 The degree of polishing of the polished surface of the base is independent of the effect of atmospheric pressure.

52. E。 Occlusal balance is not related to the retention of the denture, but rather to chewing efficiency and comfort.

53. C。 The density of the alveolar bone is not directly related to the retention of the denture, but to the support of the bone.

54. B。 A thick, tough mucosa is beneficial for retaining and supporting full dentures, as it is better able to withstand pressure while providing stability.

55. C。 If a maxillary full denture is found to be tilted from side to side, the accuracy of the impression should be considered first, as inaccurate impressions can lead to poor fit between the base and mucosa.

56. D。 A proper concave polished shape can avoid cheek and tongue bites, as it guides food and tongue to the correct position, reducing bites.

57. E。 In edentulous oral examinations, saliva pH is not routinely examined.

58. C。 A shallow labial-buccal fold generally does not require surgical excision as it does not negatively affect the retention and support of the denture.

59. E。 All of the above options are generally advocated for surgical excision prior to restoration, as they may affect the suitability and comfort of the denture.

60. C。 Alveolar bone correction is usually not required for molars pads as it does not directly affect the retention and support of dentures.

61. C. Alveolar bone trimming. Cause: Sharp bone spurs need to be removed by alveolar bone revision surgery in order to fit a full denture.

62. E. Alveolar ridge reconstruction. Causes: Reconstructive surgery can increase the height of the alveolar ridge.

63. A. Accuracy of impressions. Why: Accurate impressions are the basis for ensuring denture retention.

64. E. Take functional impressions. Reason: Edentulous impressions should be static, and functional impressions should be used for toothed jaws.

65. A. Showing signs of muscle function trimming. Reason: The model of a full denture should not show signs of muscle function trimming.

66. C. 1 mm above the flutter line. Causes: The trailing edge of the tray should slightly exceed the fibrillation line to ensure that all necessary anatomical structures are covered.

67. E. Temporary base was eventually replaced by thermoformed plastic. Reason: Temporary base can be replaced by thermoformed plastic, but this is not a false statement, it is temporary.

68. D. Occlusion. Cause: Occlusion brackets are used to document the jaw position.

69. C. Determine the correct intermaxillary distance. Reason: Determination of the jaw position relationship involves ensuring the correctness of the distance between the jaws.

70. B. Restore the appropriate vertical distance of the face. Causes: Determining the jaw position involves ensuring that the vertical distance of the face is appropriate.

71. C. 5mm。 Reason: The width of the widest part of the rear embankment area is usually 5mm to ensure adequate support.

72. C. Maxillary recording. Reason: The main purpose of the occlusal bracket is to document the jaw position.

73. A. The original occlusal surface size and shape must be restored. Reason: Full crown restoration does not necessarily have to completely replicate the size and shape of the original occlusal surface, but should be designed according to functional and aesthetic needs.

74. D. Alar tragus line. Reason: The plane of the occlusal embankment in the posterior region should be parallel to the tragus line of the alar of the nose, which is one of the reference planes.

75. B. Lip level. Causes: The level of the embankment is usually flush or nearly flush with the lower edge of the upper lip.

76. B. The distance from the base of the nose to the base of the chin when the upper and lower teeth of the natural dentition come into contact. Cause: The occlusal vertical distance usually refers to the distance between two specific points at the occlusal position.

77. C. Vertical distance. Causes: The distance between the upper and lower mandibular alveolar crests in the median position of the lower jaw in edentulous patients is called the vertical distance.

78. A. The distance from the base of the nose to the base of the chin is measured in the jaw position, i.e. the vertical distance. Cause: The distance from the base of the nose to the base of the chin measured during the jaw rest position can be used to determine the vertical distance.

Endodontics

**Chapter 1 Introduction to Caries Diseases**

1.2012U2A1093 Which of the following diseases is not associated with genetic factors ( )

A. Congenital edentulism

B. Periodontal disease

C. Dental caries

D. Malocclusion

E. Opalescent dentin

2.2009U2A1086 The first good tooth surface for caries is ( )

A. Labrobuccal surface

B. Tongue and palatine surface

C. Near-median surface

D. Distal plane

E. Occlusal surface

3.2012U2A1054 The distribution characteristics of dental caries are ( )

A. More neck than occlusal surface

B. More lower jaw than upper jaw

C. There are more anterior teeth than posterior teeth

D. More proximal surfaces than occlusal surfaces

E. More buccal surface than occlusal surface

4. 2012U2A1055 The most common site of caries is ( )

A. Molar occlusal surface pit and fissure

B. Posterior tooth adjudication surface

C. Anterior tooth neck

D. Pits and fissures, adjacency surfaces, and tooth necks

E. Neck of posterior teeth

5.2004U2A1075 Caries is caused by the hard tissue of the tooth ( )

A. Acute progressive destruction

B. Acute intermittent destruction

C. Chronic progressive destruction

D. Chronic intermittent disruption

E. All of the above types of destruction exist

6.2004U2A1076 Caries can also be referred to as hard tissue of teeth ( )

A. Bacterial infectious diseases

B. Viral infectious diseases

C. Immunoreactive diseases

D. Allergic diseases

E. Chemical irritant diseases

7.2012U2A1084 Caries is more common in teeth ( )

A. Self-cleaning area

B. Marginal crest

C. Detention area

D. Tongue surface

E. Cusp

8.2013U2AN148 Among the 300 students in the sixth grade (12-year-old age group) of a school, 180 had caries, lost or filled permanent teeth, 328 untreated caries, 3 teeth lost due to caries, and 69 filled teeth due to caries. The caries rate of students in this grade was ( )

A. 0.64

B. 0.62

C. 0.6

D. 0.58

E. 0.56

GPT-4

· B. Periodontal disease. Causes: Periodontal disease is associated with a variety of factors, including environment, lifestyle, and genetics, but genetic factors are not the only determining factor.

· E. Occlusal surface. Causes: Caries is most common here as occlusal pits and fissures are prone to food residue and bacterial accumulation.

· D. More proximal surfaces than occlusal surfaces. Causes: Adjacent surfaces, especially those of the back teeth, are prone to plaque accumulation due to difficulty in cleaning, leading to caries.

· D. Pits and fissures, adjacency surfaces, and tooth necks. Causes: These areas are common areas for food residue and bacterial accumulation and are prone to caries.

· C. Chronic progressive destruction. Causes: Caries is a slow and continuous process of destruction of the hard tissues of the tooth caused by acids metabolized by bacteria.

· A. Bacterial infectious diseases. Causes: Caries is a disease of the hard tissue of teeth caused by bacteria (such as Streptococcus mutans) metabolizing food scraps to produce acids.

· C. Detention area. Causes: The retention area is prone to the accumulation of food debris and plaque, and is a high-risk area for caries.

· A. 0.64。 Reason: caries rate = number of patients / total number of people, 180/300 = 0.60, but the data in the question may refer to other considerations, option A is the closest to the calculation result.

**Chapter 2 Etiology and Pathogenesis of Caries**

1.2016U2A1031 Foods that are particularly cariogenic are ( )

A. Vegetables

B. Milk

C. Peanuts

D. Cookies

E. Eggs

2.2016U2A1048 The correct statement about plaque is ( )

A. There was no significant difference in plaque structure among the sites

B. There was no significant difference in bacteria within the same oral plaque

C. Plaque consists of a sticky matrix and bacteria embedded in it

D. The main components of the matrix are salivary glycoproteins, the intracellular polymers of bacteria

E. In the plaque-dental interdental layer, the bacteria are scalloped and attach directly to the enamel

3. 2009U2A1093 It is currently believed that the main cariogenic bacteria in humans are ( )

A. Lactobacillus

B. Streptococcus salivarius

C. Hemostreptococcus

D. Streptococcus mutans

E. light streptococcus

4.2009U2A1096 The correct order of supragingival plaque formation process is ( )

A. Acquired membrane formation → initial aggregation→ bacteria grow and multiply rapidly→ plaque matures

B. Initial aggregation → acquired membrane formation→ bacteria grow rapidly and multiply→ plaque matures

C. Initial aggregation→ rapid bacterial growth and multiplication→ acquired membrane formation→ plaque maturation

D. Acquired membrane formation→ bacteria grow rapidly and multiply→ initial aggregation → plaque maturation

E. Initial aggregation→ bacteria grow rapidly → plaque matures

5.2009U2A1103 Food is a cause of caries, which of the following is wrong ( )

A. Retention of food on the tooth surface can cause caries

B. The amount of sugar eaten is more important than the number of times you eat sugar for the occurrence of caries

C. Phosphorus content in food is associated with the occurrence of caries

D. Physical properties of food are closely related to the occurrence of caries

E. Sucrose must be bacteria to cause caries

6.2008U2A1047 The following minerals or elements with anti-caries effect are ( )

A. Fluorine

B. Carbonates

C. Selenium

D. Zinc

E. Iron

7.2008U2A1058 Time required for the formation of an acquired film on a clean tooth surface ( )

A. Minutes

B. 1~2 hours

C. 2~10 hours

D. 10~24 hours

E. 24~48 hours

8. 2010U2A1064 The enzyme that plays a major role in the adhesion of Streptococcus mutans to the tooth surface is ( )

A. Hyaluronidase

B. Glucosyltransferase

C. Lactate dehydrogenase

D. Glucose polymerase

9.2010U2A1068 Streptococcus mutans was divided into 8 serotypes A~H according to the bacterial myrosaccharide antigen, among which the serotype with the highest detection rate in humans was ( )

A. acf

B. def

C. cef

D. dce

E. gef

10.2010U2A1070 Which of the following is wrong about the acquired membrane ( )

A. Its number is inversely correlated with the occurrence of caries

B. Acts as a diffusion barrier to repair and protect the enamel surface

C. Affects the adhesion of bacteria on the tooth surface

D. as a substrate and source of nutrients for bacterial metabolism

11.2012U2A1053 The bacteria that are currently thought to be most closely associated with caries are ( )

A. Lactobacillus

B. Actinomycetes

C. Hemostreptococcus

D. Streptococcus mutans

12.2012U2A1056 Antibodies most closely associated with the onset of caries in human saliva ( )

A. IgA

B. SIgA

C. IgM

D. IgM and IgA

1. D. Cookies. Because foods high in sugar, such as biscuits, are more likely to cause caries.
2. C. Plaque consists of a sticky matrix and bacteria embedded in it. This is a correct description of the basic structure of plaque.
3. B. Streptococcus salivarius. It is the most common cariogenic bacteria.
4. A. Acquired membrane formation → initial aggregation→ bacteria grow and multiply rapidly→ plaque matures. This is the correct order in which supragingival plaque is formed.
5. B. The amount of sugar eaten is more important than the number of times you eat sugar for the occurrence of caries. In fact, frequent sugar intake has a greater impact on caries.
6. A. Fluorine. Fluorine has a pronounced anti-caries effect.
7. A. Minutes. Acquired films can be formed in a matter of minutes.
8. B. Glucosyltransferase. This enzyme plays a major role in the adhesion process of Streptococcus mutans.
9. Not sure, but I'm guessing it's C. CEF, as it may be a common serotype.
10. B. Acts as a diffusion barrier to repair and protect the enamel surface. This description seems to be more consistent with the function of tooth enamel than with the acquired membrane.
11. D. Streptococcus mutans. It is widely considered to be most closely associated with caries.
12. B. SIgA。 It is the main antibody in saliva and is closely related to the prevention of caries.

**Chapter 3 Clinical Manifestations and Diagnosis**

1.2003U2A1007 Transillumination method, which needs to be carried out in a dimly lit room, can be used to inspect ( )

A. Adjacent caries

B. Whether the pulp is necrotic

C. Cracked teeth

D. Root canal orifice

E. Either of the above

2.2016U2A2079 Some occlusal surfaces (pits and fissures) caries are tapered, with the bottom facing the dentin and the tip pointing to the enamel surface.

A. Wet caries

B. Violent caries

C. Stealth caries

D. Resting caries

E. Linear enamel caries

3. 2008U2A1036 The most commonly used routine diagnostic method for superficial caries is ( )

A. Temperature stimulation test

B. Inspection method

C. Transillumination

D. X-rays

E. Percussion

4.2008U2A1051 When caries in dentin, the changes produced by the dentin pulp complex are ( )

A. Pulp hyperemia

B. Chronic inflammation of the pulp

C. Pulp degeneration

D. Secondary dentin formation

E. Restorative dentin formation

5. 2010U2A1067 The pain of deep caries is manifested as ( )

A. Provoke pain

B. Spontaneous severe pain

C. Both

D. Neither

6. 2003U2A1017 Caries that is more common in patients with Sjogren's syndrome is called ( )

A. Occult caries

B. Resting caries

C. Rampant caries

D. Root caries

E. Annular caries

7.2013U2A1023 Which of the following methods can be used to help diagnose secondary caries that are not easy to diagnose in clinical practice ( )

A. Visitation

B. Temperature test

C. X-ray

D. D. Dyeing

E. Anesthesia

8. 2012U2A1081 The clinical manifestations of deep caries are ( )

A. Less severe pain in the presence of cold, heat and chemical stimuli

B. Cavities form, painful and sometimes spontaneous in response to cold, heat, and chemical stimuli

C. There may be release pain, which cannot be localized

D. Cavities are formed, pain is caused by cold, heat and chemical stimuli, and the symptoms disappear immediately after the stimulation is removed

E. Cavities form, pain in the presence of cold, heat, and chemical stimuli, and pain persists for a longer time after the stimulus is removed

9.2011U2A1019 Which type of caries classification is most suitable for clinical practice ( )

A. Classification according to the incidence of the disease

B. Classification by rate of progress

C. Classification by anatomical site of injury

D. Classification by depth of lesion

E. Other classifications

10.2012U2A2125 Male, 35 years old. The pain in the right upper posterior tooth is evident when eating, and there is no spontaneous pain. The right upper posterior tooth has been treated with a "filling". Oral examination: there is an amalgam filling on the upper right 6 occlusal surfaces, caries lesions around the filling, percussion (-), probing (-), no loosening. Temperature stimulation test (+), symptoms disappear immediately after the stimulation is removed. Can be diagnosed as ( )

A. Resting caries

B. Secondary caries

C. Violent caries

D. Sneaking caries

E. Root caries

11.2014U2A2126 Female, 18 years old. The upper anterior teeth are dotted with blackness between the teeth. There is no spontaneous pain and pain of cold and heat irritation. Oral examination: punctate dark spots on the upper left and right 1 proximal and mid-adjacent surfaces, rough surface, percussion (-), probing (-). The most likely diagnosis is ( )

A. Superficial caries

B. Medium caries

C. Deep caries

D. Resting caries

E. Secondary caries

12. 2014U2A2127 patient, female, 20 years old. Present to the doctor because of the discomfort of sweet food in the left back tooth. Examination: cavities on the lower left 6 occlusal surfaces, medium depth, soft and slightly sensitive, slightly sensitive after cold stimulation into the cavity, and the hot and cold test was the same as that of the control teeth. The diagnosis of the affected tooth may be ( )

A. Superficial caries

B. Medium caries

C. Deep caries

D. Pulp congestion

E. Chronic pulpitis

13. 2014U2A2128 patient female, 20 years old. Present with pain in impacted food when eating in the left back tooth. Examination: caries cavity on the occlusal surface of the lower left 6, sensitive to the bottom of the cave, percussion (-), pain after cold stimulation into the hole, immediate disappearance of the removal of the stimulus, and the same as the control tooth for thermal measurement. The dental diagnosis may be ( )

A. Superficial caries

B. Medium caries

C. Deep caries

D. Chronic pulpitis

E. Acute pulpitis

14.2014U2A2130 The patient sought medical attention with sweet tooth pain in the right lower back teeth. Examination: lower right 6 distal median caries, sensitive, hot and cold measurement with control teeth. The pigmentation of the lower right 7 cheeks can be jammed, and the cold test is the same as that of the control tooth, and the diagnosis is ( )

A. Superficial caries in the lower right 7 buccal surfaces, and distal median caries in the lower right 6 faces

B. Distal median surface in the lower right 6 and middle buccal surface in the lower right 7

C. Middle caries in the lower right 7 buccal faces, and deep caries in the distal face in the lower right 6

D. Superficial caries on the lower right 7 buccal surfaces, and deep caries on the distal and median surfaces in the lower right 6 below

None of the above diagnoses are correct

1. E。 Causes: Transillumination can be used to check for a variety of dental problems, including proximal caries, pulp status, cracked teeth, and root canal orifice.
2. C。 Causes: Latent caries is usually tapered in shape, with the base facing the dentin and the tip pointing toward the enamel surface.
3. B。 Causes: Visual inspection is the most commonly used method for diagnosing superficial caries because it allows direct visualization of damage to the tooth surface.
4. E。 Causes: When caries occurs in the dentin, the formation of restorative dentin is often triggered to protect the pulp.
5. C。 Causes: Deep caries may cause irritating pain and spontaneous excruciating pain.
6. D。 Causes: A common caries disease in patients with Sjogren syndrome is root surface caries.
7. C。 Causes: X-rays can help diagnose secondary caries that are not easily detected.
8. B。 Causes: Clinical manifestations of deep caries usually include cavities formation and pain in response to heat, cold, and chemical stimuli.
9. D。 Causes: The classification of caries by depth of lesion is most clinically appropriate because it directly reflects the severity of the disease.
10. B。 Causes: Secondary caries refers to the formation of new caries lesions around the filling, which is appropriate in this patient.
11. A。 Causes: punctate blackening and rough surface, no spontaneous pain and pain of hot and cold irritation, may be shallow caries.
12. B。 Cause: Medium-deep cavities, soft and slightly sensitive, indicating medium caries.
13. A。 Causes: Pain with impaction of food during eating, sensitive to the bottom of the cave, but pain disappears immediately after cold stimulation and may point to superficial caries.
14. A。 Causes: The lower right 6 distal surface is sensitive, and the lower right 7 cheek surface can be stuck with the probe, indicating that the lower right 7 is shallow caries, and the lower right 6 is middle caries.

**Chapter 4 Treatment Plan for Caries Disease**

1.2009U2A1101 Treatment of dental caries ( )

A. Pharmacotherapy

B. Pharmacotherapy + abrasion

C. Filling

D. Filling + drug therapy + abrasion

E. Medication + Filling + Extraction

2.2012U2A1029 When filling the cavity with composite resin, which of the following disinfectants should be used ( )

A. 50% vanillol ethyl musk alcohol solution

B. Camphor phenol mixture

C. 75% ethanol

D. Chlorophenol camphor mint

3.2010U2A1075 When the composite resin is filled, the cavity disinfectant used is ( )

A. 50% thymol

B. Camphrol

C. Clove oil

D. Wood grenade oil

E. 75% ethanol

4. 2000U2AN148 female, 35 years old, came to the doctor because the left lower back tooth was sensitive to hot and cold stimuli for 1 week. Examination found that the occlusal surface of the left lower first molar was deeply caries, and the bottom of the cave was sore, and the cold test reaction was transiently sensitive, and percussion pain (-). There is no abnormality in the gums. The diagnosis of the tooth is reducible pulpitis and is managed by ( )

A. Palliative treatment

B. The base of zinc phosphate adhesive powder is permanently filled

C. The base of zinc polycarboxylate cemented powder is permanently filled

D. Zinc oxide clove oil cementing powder pad is permanently filled

E. Direct permanent filling

5. 2015U2AN150 patient, a 25-year-old male, had pain due to eating hot and cold food in the right lower back tooth for 1 week, and had no history of spontaneous pain or nocturnal pain. Examination: cavities on the lower right 7 cheeks, slightly sensitive, percussion (-), loose (-), gingiva (-), cold and hot measurements with control teeth. Cavities on the lower right 6 cheeks, sensitive, percussion (-), loose (-), gingival (-), pain caused by hot and cold measurements, disappears immediately after removal. There is no abnormality in the remaining teeth, the best way to deal with it ( )

A. Amalgam filling on the lower right 7 cheeks, zinc oxide clove oil paste on the lower right 6 cheeks soothing

B. Silver-mercury filling of the lower right 7 cheeks

C. Right lower 67 buccal zinc oxide clove oil paste to soothe

D. Amalgam filling of the lower right 7 buccal surfaces, and endodontic treatment of the lower right 6 buccal faces

E. None of the above is optimal

6. 2010U2A1088 Drug treatment of caries is not suitable for ( )

A. Resting caries

B. Rampant caries

C. Superficial dentin caries

D. Shallow caries that is not holed

E. Deciduous tooth caries

7.2014U2A1033 The purpose of drug treatment for dental caries cannot be achieved ( )

A. Maintain intact dentition

B. Restore the appearance

C. Inhibition of caries progression

D. Avoid complications

E. Protect the pulp

8.2005U2A1042 Over time, once a cavity is formed, the tooth ( )

A. There is a possibility of self-repair

B. After antimicrobial treatment, there is a possibility of self-repair

C. After being treated by a special induction method, there is a possibility of self-repair

D. There is no possibility of self-repair

E. Rarely self-repairing

1. D。 Causes: Treatment of dental caries usually involves a combination of pharmacotherapy, abrasion, and filling.
2. C。 Reason: When filling cavities with composite resins, 75% ethanol is often used as a disinfectant.
3. E。 Why: 75% ethanol is a commonly used disinfectant for cavity disinfection.
4. C。 Causes: Reducible pulpitis is usually treated with a permanent filling of a polycarboxylate zinc cemented powder base.
5. D。 Reason: The lower right 6 cheek face endodontic fillings are performed after treatment, and the lower right 7 cheeks can be filled with mercury.
6. A。 Causes: Quiescent caries does not require medication because it no longer progresses.
7. E。 Causes: Medications for dental caries do not protect the pulp, and their main purpose is to control the disease and slow the progression of caries.
8. D。 Cause: Once the cavity is formed, there is no possibility of self-repair of the tooth and artificial treatment is required.

**Chapter 5 Abnormal Dental Development and Stained Teeth**

1. 2014U2A1038 Clinical manifestations of deep caries, incorrectly described ( )

A. The caries lesion has reached deep into the dentin

B. Most have hot and cold triggering pain

C. Sensitive to sweet and sour foods

D. Spontaneous pain may also occur occasionally

E. Caries embedded in food is painful

2. 2000U2A1033 is inconsistent with the clinical manifestations of deep caries ( )

A. The bottom of the cave is deep, close to the medullary cavity

B. Food embedding pain

C. Occasional dull pain at night

D. Sweet and sour irritation

E. Pulp viability test normal

3.2003U2A1009 The pathogenesis and pathology of enamel tooth are incorrect ( )

A. When fluorine concentration is too high, alkaline phosphatase activity decreases

B. The surface enamel is porous

C. Poor interstitial mineralization of the glaze columns, over-mineralization of the glaze columns

D. Fluoride has a greater effect on dentin than on enamel

E. Fluoroplamine is caused by the adsorption of foreign pigments

4.2004U2A1072 Structural abnormalities of teeth include ( )

A. Enamel hypoplasia

B. Opalescent dentin

C. Dental fluorosis

D. Tetracycline teeth

E. All of the above

5.2016U2A1034 Deformed central cusp is commonly found in ( )

A. Left and right down 5

B. Left and right on 5

C. Left and right lower 4

D. Left and right on 4

E. Left and right up 45

6.2016U2A1037 Enamel hypoplasia at the incision margin of maxillary lateral incisors indicates that the causative factor has been extended to ( )

A. Before birth

B. Under 1 year of age

C. Year 2 after birth

D. After 2~3 years old

E. After the age of 4

7.2009U2A1097 Which is wrong about the distinguishing point between dental fluorosis and enamel hypoplasia ( )

A. Dental fluorosis is more common in permanent teeth, and tooth enamel hypoplasia is prone to occur in both baby teeth and permanent teeth

B. Enamel hypoplasia with well-defined chalky spots and less defined dental fluorosis

C. Enamel hypoplasia can occur in a single tooth or a group of teeth, whereas dental fluorosis is more common in many teeth

D. Patients with dental fluorosis may have a history of living in areas with high fluoride

E. Enamel hypoplasia: Chalky lines coincide parallel to the enamel growth line, whereas dental fluorosis does not coincide

8. 2009U2A1104 Drugs that can cause tetracycline teeth are ( )

A. Pioneermycin and tetracycline

B. Oxytetracycline and norchlortetracycline

C. Penicillin and tetracycline

D. Streptomycin and chloramphenicol

E. Jethromycin and gentamicin

9. 2008U2A2082 patient, 11 years old, found that the newly erupted left lower 5 with a pointed and long deformed central cusp, the correct treatment is ( )

A. Regular observations

B. Adjust the opposite teeth appropriately, and at the same time grind the tip several times in small quantities

C. Under anesthesia and strict disinfection, the tip is ground off once and the medulla is routinely treated

D. B and C

E. Apical induction

10. 2011U2A1011 Deformed central cusp is most likely to occur in the crown of the tooth ( )

A. Buccal crest

B. Fovea

C. Near median fossa

D. Distal fossa

E. Tongue crest

11.2011U2A1015 glaze beads are often located in ( )

A. Within or near the root fork

B. Tooth nature

C. On tooth enamel

D. Apex

E. 1/3 of the root

12.2000U2A1018 Turner (Turer) tooth is due to ( )

A. Caused by taking tetracycline

B. Caused by excessive fluoride content in drinking water

C. Severe febrile illness in the child

D. Congenital developmental defects

E. Severe periapical infection of deciduous teeth

13.2015U2A2091 The patient was 30 years old, due to the treatment of other decayed teeth, it was found that there was a round blunt conical protrusion in the center of the occlusal surface of the lower left 5, the occlusal relationship between the upper left 45 and the lower right 45 was normal, percussion (-), the cold and heat measurement was the same as the control tooth, how should the tooth be treated ( )

A. No treatment is necessary, regular observation

B. Silver-mercury filling of the preparation hole

C. Filling of glass ions in the preparation hole

D. Directly capping the medulla

E. Cutting of the living myel

14.1999U2A2079 The patient requested aesthetic treatment of the anterior teeth, and felt that there were spots on the posterior tooth surface from the eruption of the teeth, and the surrounding adjacent surfaces also had similar manifestations. Check: scattered yellow-brown and chalky spots of varying degrees can be seen in the whole mouth of the dentition. The tooth is diagnosed with ( )

A. Enamel hypoplasia

B. Dental fluorosis

C. Tetracycline teeth

D. Superficial caries

E. Hereditary opalescent dentin

15. 2012U2A2113 The 30-year-old patient presented with recurrent swelling and pain in the left lower back tooth. Check: no caries in the lower left 5, central round black ring, dentin axis visible on occlusal surface, probe unresponsive, percussion (+), X-ray: apical lesion, apical foramen is flare-shaped. The tooth may be caused by ( )

A. Abrasion

B. Cracked teeth

C. Deformed central cusp

D. Occlusal trauma

E. Enamel hypoplasia

16.2012U2A2114 The patient requested treatment due to white spots on the upper anterior teeth. Examination: chalky spots on the left and right upper 1 labial surfaces, smooth and hard. Yu (-). The tooth is diagnosed with ( )

A. Superficial caries

B. Enamel hypoplasia

C. Dental fluorosis

D. Tetracycline teeth

E. All of the above diseases are possible

17.2003U2A1026 Fluoridation of water source is an effective means of preventing caries, and the standard fluorine concentration of water quality in China is ( )

A. 0.05ppm or more ~ 0.1ppm or less

B. 0.1ppm or more ~ 0.5ppm or less

C. 0.5ppm or more ~ 1ppm or less

D. More than 1ppm ~ less than 5ppm

E. More than 5ppm ~ less than 10ppm

18.2004U2A1080 The following correct statement about the abnormal number of teeth is ( )

A. Manifested by insufficient and excessive numbers

B. Insufficient number of teeth, including congenital and acquired missing teeth

C. Congenital edentulism is a manifestation of mesodermal hypoplasia

D. Wisdom teeth are the most common type of extra teeth

E. Congenital edentulism does not occur in baby teeth

19.2012U2A1072 Among the following diseases, the second bicuspid of the mandibular is more common ( )

A. Deformed central cusp

B. Deformed lingual fossa

C. Deformed root sulcus

D. Deformed lingual tip

E. Glazed beads

20.2012U2A1082 Turner tooth is due to ( )

A. Caused by chickenpox or scarlet fever in children

B. Caused by severe dyspepsia in children

C. Severe periapical infection of primary teeth in children affects the inheritance of permanent teeth

D. Pregnant women with rubella or toxemia

E. Caused by decreased parathyroid function

· E。 Food embedded in cavities usually does not cause pain directly, but rather pain due to inflammation of the pulp caused by cavities.

· E。 A normal pulp viability test is usually inconsistent with the clinical manifestations of deep caries, which often causes inflammation or damage to the pulp.

· D。 The effect of fluorine on dentin is generally not greater than the effect on enamel, and the effect of fluorine on plaque enamel is mainly related to the effect of fluorine during enamel mineralization.

· E。 The above options are all examples of structural abnormalities in the teeth, including enamel hypoplasia, opalescent dentin, dental fluorosis, and tetracycline teeth.

· B。 Deformed central cusps are common in the anterior teeth, especially the maxillary anterior teeth, so the left and right upper 5 is a reasonable option.

· A。 The appearance of enamel hypoplasia is usually related to prenatal factors, such as genetics or maternal influences during pregnancy.

· C。 Enamel hypoplasia can occur on a single tooth or a group of teeth, whereas dental fluorosis usually affects multiple teeth.

· A。 Both tetracycline and pioneer mycin can cause tetracycline teeth.

· B。 Treatment of the deformed central cusp can be performed by appropriately adjusting the jaw teeth and grinding the cusp several times in small quantities.

· B。 The malformed central cusp is most likely to occur in the central fossa of the crown.

· A。 Glaze beads are usually located within or near the root branch.

· E。 Turner teeth are usually caused by a severe periapical infection during the deciduous period.

· A。 If the tooth is asymptomatic and the hot and cold tests are normal, only regular observation can be done.

· B。 Yellowish-brown and chalky spots may be characteristic of dental fluorosis.

· A。 The tooth is caries-free, but there is a central round black ring and abrasion, which may be due to abrasion.

· B。 White spots are smooth and hard and may be a sign of enamel hypoplasia.

· C。 China's water quality standard fluoride concentration is generally recommended to be more than 0.5ppm ~ 1ppm to prevent tooth decay.

· A。 An abnormal number of teeth can manifest as insufficient or excessive number.

· A。 Malformed central cusps are commonly found in the anterior teeth, including the mandibular second bicuspid.

· C。 Turner teeth are usually affected by severe infections during the baby teeth that affect the development of permanent teeth.

**Chapter 6 Dental Trauma**

1.2003U2A1001 Tooth fracture often occurs in the following tooth positions ( )

A. Upper front teeth

B. Lower anterior teeth

C. Single cuspid

D. Premolars

E. Teeth grinding

2.2016U2A1045 root fold often occurs in ( )

A. 1/3 of the root apex of mature permanent teeth

B. 1/3 of the roots of young permanent teeth

C. 1/3 of the roots of mature permanent teeth

D. 1/3 of the root neck

E. 1/3 of the root apex of young permanent teeth

3.2000U2A1017 The description of tooth concussion is correct ( )

A. Pulp viability test was performed on the affected tooth with tooth convulsion, and the response was mixed

B. The pulp of a young permanent tooth will not lose its vitality after concussion

C. Young permanent teeth lose their vitality quickly after shock

D. Teeth are usually displaced after concussion

E. Tooth concussion is usually accompanied by a defect in the tooth tissue

4. 2015U2A2092 The patient, 20 years old, had a traumatic injury 1 hour ago, and his upper anterior teeth became shorter and required treatment. Check: the upper left 1 crown is 3mm shorter than the adjacent tooth, the crown is intact, percussion (+), the gingiva is mildly red and swollen, X-ray: periapical periapical membrane disappears. The dental treatment is ( )

A. Wait for it to emerge naturally and observe it regularly

B. Post-reduction fixation, regular observation

C. Reduction and fixation after endodontic treatment

D. Endodontic treatment after 4 weeks of reduction and fixation

E. Uprooting

5. 2015U2A2093 Patient 20 years old, traumatic 10 minutes ago, upper anterior teeth loss, requiring treatment. Check: 1 complete dislocation on the left, 2 on the left and 1 on the right with a complete crown, percussion (±), loose (-), what should the patient do ( )

A. Immediate replantation fixation with regular observation

B. After endodontic treatment, reduction and replantation are fixed

C. 4 weeks after reduction and fixation, root canal treatment

D. Ask the patient to repair the wound after healing

E. Immediate Fix

6. 2015U2A2095 The patient suffered a bruise on his upper anterior teeth during exercise one year ago, and had bite pain at that time, but no other discomfort and was not treated. Discoloration of the crown is found later, which may be due to ( )

A. Pigmentation

B. Pulp necrosis

C. Pulp degeneration

D. Dentin degeneration

E. None of the above

7.2015U2A2098 Trauma to the upper anterior teeth in adults, 1/3 of the crown fold, exposing the pulp, percussion (-). The principle of treatment is ( )

A. Vital myeloidectomy

B. Directly capping the medulla

C. Dry pulp surgery

D. Root canal treatment

E. Plastination therapy

8.2013U2A2124 Patient with upper anterior tooth trauma, present. Check: the upper left 1 crown is loose III. degree, gingival bleeding, percussion (++), X-ray shows that the root fold is located at 1/3 of the neck, and the remaining root is about 15mm long.

A. Fixation

B. Extraction of the affected tooth

C. Remove the crown and restore it directly

D. The crown of the tooth is extracted and the root is treated with a root canal

E. Direct photoadhesive repair

9.2013U2A2130 The patient was 16 years old, male, 1 hour ago, upper anterior tooth trauma, checked: upper left 1 lip dislocation, loose II. degree, percussion (±), mild redness and swelling of the gums, upper right 1 upper left 2 percussion (-), loose (-), pulp viability measurement was the same as that of the control tooth, no root fracture was found on X-ray, the patient was treated as ( )

A. Self-healing

B. Occlusion observation

C. Reduction fixation, observation

D. Reduction and fixation after endodontic treatment

E. Uprooting

10.2012U2A1068 Loss of pulp vitality of young permanent teeth can be done after injury at the latest ( )

A. 4 months

B. 6 months

C. 8 months

D. 10 months

E. More than 1 year

1. A. Upper front teeth. Because the upper anterior teeth are more susceptible to external impact due to their position and functional characteristics, resulting in fracture.
2. B. 1/3 of the roots of young permanent teeth. The roots of young permanent teeth are not fully developed, and the middle 1/3 part is weak, which is prone to breakage.
3. A. Pulp viability test was performed on the affected tooth with tooth convulsion, and the response was mixed. Because the pulp response after tooth concussion can vary depending on the degree of injury, it does not necessarily lead to loss of pulp viability.
4. B. Post-reduction fixation, regular observation. Because the crown is intact and mildly red, it may only be necessary to reduce it and fix it and observe the pulp regularly.
5. A. Immediate replantation fixation with regular observation. A completely dislocated tooth should be replanted and fixed as soon as possible to promote alveolar bone healing.
6. B. Pulp necrosis. Crown discoloration is usually a sign of pulp necrosis because blood breakdown products in the dead pulp can cause the crown to discolor.
7. D. Root canal treatment. Root canal therapy should be considered to remove the infected pulp tissue when the crown folds the pulp.
8. B. Extraction of the affected tooth. The root fold is located at 1/3 of the neck, and the remaining root is not long enough to support the crown and may need to be extracted.
9. C. Reduction fixation, observation. Misaligned labial and slightly loose teeth can be treated with reduction and fixation to see how well the teeth heal.
10. E. More than 1 year. The loss of vitality of the pulp may not occur immediately and can sometimes take more than 1 year to fully manifest itself.

**Chapter 7 Chronic Dental Injury**

1.2003U2A1013 Wedge defects often occur in ( )

A. Upper front teeth

B. Lower anterior teeth

C. Upper and lower mandibular bicuspids

D. Maxillary molars

E. Mandibular molars

2.2016U2A1032 The main symptoms of cracked teeth are ( )

A. Pulp hyperemia

B. Acute pulpitis

C. Acute apical inflammation

D. Chronic pulpitis

E. Any of the above symptoms may occur depending on the location and degree of the fissure

3.2016U2A2078 Superficial fissure, no obvious symptoms, and normal pulp viability, the treatment should be the first choice ( )

A. Open marrow inactivation

B. Full crown restoration

C. Fill the hole

D. Occlusion therapy

E. Extraction of the affected tooth

4.2016U2A1049 The following are not chronic dental injuries ( )

A. Abrasion

B. Wedge defect

C. Acid etching

D. Crown folding

E. Cracked teeth

5.2009U2A1084 Cracked tooth may be accompanied by symptoms ( )

A. Temperature irritation pain

B. Painful bite

C. Spontaneous pain

D. No obvious symptoms

E. All of the above are possible

6. 2008U2A2084 The patient was 72 years old with spontaneous labor pain in the lower anterior teeth. The anterior teeth were obviously worn, the left and right lower 1 were cold (++), percussion (-), and the X-ray showed that the periodontal condition was normal. The route of infection of the pulp is ( )

A. Periodontal infection

B. Bloodborne infections

C. C. Cies

D. Cracked teeth

E. Abrasion

7. 2008U2A1053 The following description of root longitudinal fissure is wrong ( )

A. Most of the patients are middle-aged and elderly

B. Maxillary first molars have the highest incidence

C. X-rays are important for diagnosing root longitudinal cracks

D. The absence of a pulp tooth is one of the important factors

E. For longitudinal root fissures with obvious looseness and wide and deep periodontal pockets, which are ineffective by conservative treatment, they should be extracted

8.2000U2A1015 The main causes of temporomandibular joint disorder caused by severe occlusal wear are ( )

A. The vertical distance between the jaws is too short, causing joint damage

B. Defects of the marginal ridge and developmental sulcus, resulting in incomplete occlusal surfaces

C. Uneven wear leaves high and steep cusps, resulting in occlusal trauma

D. Dentin hypersensitivity, resulting in insufficient dental force and damage to the joints

E. Long-term chewing concentrates the stress of the tooth resultant force and damages the joint

9. 2015U2A2096 The patient requested treatment for the right posterior tooth occlusal pain, hot and cold pain for half a year. Check: no caries in the upper right 6, suspicious fissure in the near and middle, pain caused by hot and cold measurements, after the stimulus is removed, it lasts for a few seconds, and percussion (±). The right posterior tooth is worn to varying degrees, sensitized. The patient's chief complaint is that the principles of dental treatment are ( )

A. No treatment

B. Occlusion

C. Desensitization

D. Full crown restoration after endodontic treatment

E. Fill the hole

10. 2012U2A2110 The patient's left upper back tooth has been biting unwell for half a year, and occasionally has sharp pain when biting a certain place. Examination: no caries and periodontal pocket were found in the upper left 4567, suspicious cracks in the proximal occlusal surface of the upper left 6, cold sensitivity, and no abnormalities were found on X-ray. Which of the following diseases is most likely to suffer from in this tooth ( )

A. Wedge defect

B. Wear and tear

C. Hidden fissures

D. Bruxism

E. Chronic pulpitis

1. A。 The upper anterior region is more prone to wedge defects due to the direction of the bite force and the characteristics of the tooth structure.
2. E。 The symptoms of a cracked tooth vary depending on the location and depth of the crack and may include all of the above symptoms.
3. C。 If the superficial fissure is superficial and the pulp viability is normal, conservative treatment such as cavity filling should be preferred.
4. D。 Corona fracture is an acute injury and is not considered chronic.
5. E。 The symptoms of a cracked tooth vary from person to person, and may range from temperature irritation, biting pain, spontaneous pain, or no obvious symptoms.
6. E。 Abrasion is a possible pathway to pulp infection, especially in older people, where abrasion can lead to pulp exposure.
7. C。 X-rays are often limited in diagnosing longitudinal root cracks because the cracks are small and not easily detected by x-rays.
8. C。 Severe occlusal surface wear can lead to uneven bite forces, leaving a high and steep cusp, resulting in occlusal trauma that affects the temporomandibular joint.
9. D。 The patient complains of occlusal pain and hot and cold pain and requires endodontic treatment, followed by a full crown restoration to restore tooth function.
10. C。 Given the patient's discomfort and sharp pain in the bite, as well as the suspicious crack that is not obvious on examination, the tooth is most likely to be cracked.

**Chapter 8 Dentin Sensitivity**

1.2015U2A2097 The patient's whole mouth and teeth are sensitive to cold and heat and require treatment. Examination: The neck of multiple teeth in the whole mouth is sensitive, there is no defect, the gingiva is slightly withdrawn, and the cold test sensitive removal irritation can be relieved. Which treatment should be used in this patient ( )

A. Fluoride desensitization

B. Ammonia silver nitrate desensitization

C. Electrocoagulation

D. Filling therapy

E. Endodontic treatment

2.2010U2A1093 The preferred treatment for dentin hypersensitivity is ( )

A. Drug desensitization

B. Endodontic treatment

C. Periodontal cleaning

D. Underfilling

E. Resin filling

3.2011U2A1029 The most commonly used method for clinical examination of dentin hypersensitivity is ( )

A. Cold diagnosis

B. Percussion

C. Sweet and sour stimulation

D. The probe is crossed over the sensitive spot

E. Inspection

4. 2014U2A1037 Definite neurodistribution of dentin is ( )

A. Peritubular dentin

B. Intertubule dentin

C. Prodentin

D. Mask dentin

E. Clear dentin

5.2015U2A2116 Male, 60 years old, complained of tooth hypersensitivity during chewing, heavy wear and tear on the tooth surface, sensitivity, cold (-), percussion (-), not loose, calculus I°, probable diagnosis ( )

A. Superficial caries

B. Medium caries

C. Dentin hypersensitivity

D. Retrograde pulpitis

E. Reducible pulpitis

6. 2015U2A2117 male, 37 years old. The left upper back tooth is sore when eating hot and cold food and biting hard objects for 1 week. No spontaneous pain. Oral examination: 67 wear on the left to reach the dentin, probing soreness. The temperature irritation test is sore, and the symptoms disappear immediately after the stimulus is removed. Not loose, periodontal examination (-). The preferred treatment is ( )

A. Endodontic treatment

B. Drug desensitization

C. Periodontal cleaning

D. Amalgamma filling

E. Composite resin filling

7. 2000U2A1021 The mechanism of dentin hypersensitivity is considered to be ( )

A. Fluid Doctrine

B. Chemical bacteriological doctrine

C. The doctrine of the living body

D. Fluid dynamics theory

E. Doctrine of protein lysis

8. 2011U2A1044 The mechanism of treatment of dentin hypersensitivity is ( )

A. Passivation of peripheral pulp nerves

B. Calcification of odontoblast protrusions

C. Degeneration of odontoblasts

D. Make the dentinal tubule diameter smaller or closed

E. Strengthens the barrier function of the enamel dentin boundary

9. 2003U2A1052 Silver compound for the treatment of dentin hypersensitivity is the use of its ( )

A. Antimicrobial effect

B. Anti-plaque effect

C. Mechanical obstruction

D. Inhibition of adhesion

E. Reduces acid production

10.2000U2A1022 In the following discussion on the treatment of dentin hypersensitivity, the wrong one is ( )

A. Dentin hypersensitivity can heal on its own and does not need to be treated

B. Allergic spots on the occlusal surface can be desensitized with thymol

C. The neck of the tooth can be desensitized with NaF

D. More confined sensitive areas can be filled for treatment

Effective treatment of dentin hypersensitivity must be to close the dentin tubules

1. A. Fluoride desensitization. Causes: Fluoride desensitization is a commonly used method to relieve symptoms caused by dentin hypersensitivity and is suitable for multiple sensitive teeth.
2. A. Drug desensitization. Causes: Drug desensitization is often the preferred treatment for dentin hypersensitivity to relieve sensitivity by reducing the opening of dentin tubules.
3. A. Cold diagnosis. Causes: Cold diagnosis is the most commonly used method to check for dentin hypersensitivity, and sensitivity is diagnosed by judging how the tooth responds to cold.
4. B. Intertubule dentin. Causes: Intertubule dentin has a neural distribution that conducts pain sensations.
5. C. Dentin hypersensitivity. Cause: Tooth hypersensitivity without significant caries or pulpitis, most likely dentin hypersensitivity according to the symptom description.
6. B. Drug desensitization. Causes: Patients feel pain only when stimulated and their symptoms are relieved quickly, and drug desensitization therapy is appropriate.
7. D. Fluid dynamics theory. Causes: The mechanism of dentin hypersensitivity is generally believed to be the fluid dynamics theory, that is, the fluid movement through the dentinal tubules causes stimulation of the pulp nerve.
8. D. Make the dentinal tubule diameter smaller or closed. Causes: The mechanism of treatment of dentin hypersensitivity is mainly to reduce sensitivity by reducing or closing the dentin tubules.
9. C. Mechanical obstruction. Reason: Silver compounds for dentin hypersensitivity are mainly through their mechanical occlusive effect, which closes the dentin tubules.
10. A. Dentin hypersensitivity can heal on its own and does not need to be treated. Reason: This statement is false, dentin hypersensitivity usually requires treatment to reduce symptoms.

**Chapter 9 Prosthodontic Treatment**

1.2015U2A2102 The patient requested treatment due to a hole in the upper anterior teeth. Check: left and right upper 1 near the middle surface caries, the ink-soaked edge crest is not destroyed, the cold test is the same as the control tooth, and the diagnosis is medium caries. During treatment, the superficial layer of dentin is removed. The tooth can be selected after one treatment ( )

A. Glass ionomer cement, composite resin filling

B. Composite resin filling

C. Vitreous ionic cement filling

D. Zinc phosphate cement cushion bottom, composite resin filling

E. Zinc oxide clove oil paste at the bottom, composite resin filling

2.2012U2A1094 Which of the following tooth defects cannot be treated by filling ( )

A. Posterior occlusal cavity

B. Anterior incision defect

C. The bite needs to be elevated

D. Posterior occlusal cavity

E. Wedge defects

3.2017U2A1001 The following are not contraindications for composite resin repair ( )

A. Those who cannot effectively isolate the treatment area

B. When the restoration extends to the root plane

C. Restoration of V. cavities in anterior teeth

D. Patients with deep abrasion or bruxism

E. If all occlusions are on the restoration

· A。 Vitreous ionomer cements can provide a backing effect, and composite resins are used for filling, which can better restore tooth shape and function.

· C。 Tooth defects that require an elevated bite often cannot be treated with filling alone and may require more complex restorations.

· C。 Restoration of anterior teeth V. cavities is not a contraindication to composite resin restoration, which is often used for the restoration of such cavities.

**Chapter 10 Tooth Preparation and Amalgam Filling**

1.2016U2A1041 Which of the following is not the resistance form of the cavity ( )

A. Inverted

B. Depth of the hole

C. Box cavity

D. Ladders

E. The shape of the cavity

2.2016U2A1056 The adjacent part of the occlusal part of the occlusal cavity is larger than the occlusal side because ( )

A. Prevent lateral dislocation of the restoration

B. Prevent vertical dislocation of the restoration

C. Affected by the direction of the glazed columns

D. Affected by the orientation of the dentinal tubules

E. Saving filling materials

3.2009U2A1089 The purpose of preparing dovetails is ( )

A. Obtain a good retention

B. Obtain a good resistance shape

C. To facilitate the complete removal of decayed tooth tissue

D. To make the cave shape aesthetically pleasing

E. To facilitate filling

4.2010U2A1054 The main purpose of making dove buckles is ( )

A. Avoid accidental puncture

B. Maximize the preservation of natural tooth tissue

C. Facilitates the restoration of adjacency

D. Prevent the filling from falling off horizontally during chewing

5.2010U2A1055 Standard cavity needs to have ( )

A. Cave walls, corners, edges, fixed shapes and resistance shapes

B. Cave walls, corners, edges and resistance shapes

C. Retained shape and cave walls, corners, and edges

D. Retained and resistant

6.2010U2A2105 15 distal adjacent occlusal surface cavities, according to the Black cavity classification method, should be ( )

A. Class I Cave

B. Class II Caves

C. Class V. Holes

D. Class IV Caves

7.2012U2A1031 Which of the following holes belongs to the category BlackII. cave ( )

A. 43 Adjacent Cave

B. 14 adjacent to the cave

C. 15 buccal side neck hole

D. 36 buccal occlusal cavity

8.2012U2A1032 Which of the following is not conducive to resistance in order to achieve a good resistance shape ( )

A. The cavity should have a certain depth in the dentin

B. The cavity is required to be box-shaped

C. Removal of empty suspension enamel

D. The entrance is small and the bottom is large

9. 2012U2A1033 The definition of resistance shape is ( )

A. Enable the filling to withstand chewing force

B. Make the filling and teeth able to withstand chewing force

C. Enabling the teeth to withstand chewing force

D. Keep the filling from loosening and falling off

10.2012U2A1034 During the filling operation, the purpose of the pad is ( )

A. Isolate irritation and protect the pulp

B. To strengthen the resistance

C. To enhance disinfection

D. To strengthen retention

11. The basic structure of the 2012U2A1037 hole consists of ( )

A. Cave walls and corners

B. Cave walls and cave edges

C. Cave walls and corners, cave edges

D. Walls and corners, edges, bottoms

12.2012U2A2100 47 buccal occlusal cavities, according to the Black cavity classification should be ( )

A. Class II Caves

B. Class I Cave

C. Class III Caves

D. Class IV Caves

13.2012U2A1041 Black Taxonomy is based on ( )

A. Extent of decay

B. Tooth surface where the cavity is located

C. The location of the caries and the characteristics of caverning

D. None of the above

14.2012U2A1042 Distal neck caries of maxillary posterior teeth should be classified as Black ( )

A. Class I Cave

B. Class II Caves

C. Class IV Caves

D. Class V Cave

15.2012U2A1044 Occlusal surface box-shaped hole, generally should penetrate deep into the dentin ( )

A. 0.2～0.5mm

B. 0.5～1mm

C. 1.0～1.5mm

D. 1.5～2.0mm

16.2010U2A1069 The basic characteristics of the box-shaped hole, which of the following is wrong ( )

A. The bottom of the cave should be flat

B. The corners of the line should be round and blunt

C. There should be a certain amount of depth

D. Inward inclination of the cave wall

17.2010U2A1057 Which of the following is wrong in order to achieve a good resistance shape ( )

A. Removal of baseless enamel

B. The cavity has a certain depth in the dentin

C. The bottom angle of the hole is clear

D. Removal of thin-walled weak tips

18.2010U2A1061 Neck hole in the mesial face of the posterior teeth is classified by Black ( )

A. Class II Caves

B. Class III Caves

C. Class I Cave

D. Class V Cave

19.2010U2A1065 According to the classification of black holes, the class IV holes are ( )

A. Begins in the hole of the pit and fissure

B. Posterior tooth proximal cavity

C. Anterior tooth proximal cavity does not include chamfers

D. Anterior tooth adjacent surface holes including climacto

E. Cavities in the third of the teeth of the labial, buccal, or lingual neck

20.2012U2A1048 Posterior occlusal hole application ( )

A. Sidewall retention

B. Subtentive retention

C. Dovetail retention

D. Nail retention

21. The basic characteristics of the box-shaped hole, which of the following is wrong ( )

A. The bottom of the cave should be flat

B. The corners of the line should be round and blunt

C. There should be a certain amount of depth

D. Dovetail buckles should be prepared

22.2010U2A1074 Preferred material for temporary sealing of cavities ( )

A. Zinc oxide, clove oil, cement

B. Zinc phosphate cement

C. Calcium hydroxide

D. Teether

E. Cave protection paint

23.2010U2A1076 According to the Black Cave Classification Method Class III. Cave is ( )

A. Begins in the hole of the pit and fissure

B. Posterior tooth proximal cavity

C. Anterior tooth proximal cavity does not include chamfers

D. Anterior tooth adjacent surface holes including climacto

E. Cavities in the third of the teeth of the labial, buccal, or lingual neck

24. Posterior tooth adjacent occlusal hole application ( )

A. Sidewall retention

B. Subtentive retention

C. Dovetail retention

D. Stent retention

E. Nailing

25.2012U2A1052 The treatment principle of the empty hanging glazed column is ( )

A. Removal

B. Retention in its entirety

C. Lower the height

D. None of the above

26.2015U2A2090 A 20-year-old female presents with a decayed tooth. Check: left 7 buccal occlusal surface dotted caries, soft probe, card probe, cold and hot measurement with the control tooth, multiple teeth in the mouth due to caries filling treatment, the best treatment of the affected tooth is ( )

A. Amercury fills the area involved in decay

B. Vitreous ion-filled parts of the caries involvement

C. Preventive extension, amalgam filling

D. Preventive expansion, glass ion filling

E. Precautionary expansion, composite resin filling

27.2012U2A1061 II. type cavity type occlusal surface dovetail shape design according to ( )

A. Depth of caries on the adjacent surface

B. Location of adjacent holes

C. Tooth position of the adjacent caries

D. Types of proximal caries

E. Size of the adjacent defect

28.2013U2A1024 According to G · V · Black's cavity classification method, which of the following sentences is correct ( )

Class A. I holes are all single-sided holes

B. Class II holes are all double-faced holes

C. Class I cavity is a cavity prepared by posterior pit and fissure caries

D. Type III cavity is a cavity prepared by caries of the adjacent surface of the anterior tooth without damaging the incision

E. V. type hole is a hole prepared by the destruction of the posterior labial (buccal) and lingual (palatal) surfaces near the gingiva

29.2003U2A1020 The medullary wall of the staircase and ( )

A. Gingival walls are parallel

B. Gingival wall vertical

C. Shaft walls are parallel

D. Parallel to the mesial wall

E. Distal wall parallel

30.2003U2A1021 Which of the following measures is wrong when preparing for deep caries ( )

A. The bottom of the cave is flat, the side walls are straight, and the two are perpendicular

B. Remove the decay

C. Protect the pulp

D. The edge of the cave is round and blunt

E. Preserve healthy tooth tissue as much as possible

31.2003U2A1022 When preparing the posterior tooth class II. hole, the width of the dovetail gorge is ( )

A. 1/4 of the buccal and lingual apical spacing

B. 1/4~1/3 of the buccal and lingual apical spacing

C. 1/3~1/2 of the buccal and lingual cusp spacing

D. 1/2~2/3 of the buccal and lingual apical spacing

E. 2/3~3/4 of buccal-lingual cusp spacing

32.2003U2A1023 The posterior tooth II type cavity type of dove tail gorge is located in ( )

A. The angle of the medullary line of the axis of the cave floor is close to the midline

B. At the angle of the medullary line of the axis of the cave bottom

C. The axis of the bottom of the cave is close to the edge crest

D. Dispose of as appropriate according to the caries

E. Any part of the buccal tongue cusp

33.2003U2A1024 trapezoidal retention can be used for ( )

A. Occlusal cavity

B. Adjacent (labial) buccal cavity

C. Adjacent tongue (palatal) facial hole

D. Posterior buccal occlusal cavity

E. All of the above:

34.2012U2A2121 Female, 47 years old. Food impaction in the left lower back tooth was uncomfortable for 3 months. No spontaneous pain. Oral examination: there is a cavity on the adjacent occlusal surface of the lower left 7, percussion (-), probing (±), no loosening. According to the classification method, the cave shape should belong to ( )

A. Cave I type

B. Class II Caves

C. Class III Caves

D. Class IV Caves

E. Class VI Cave

35.2015U2A2101 The patient requested treatment due to a hole in the lower left posterior tooth. Examination: deep cavities in the lower left 6 proximal occlusal surfaces, sensitization, percussion (-), normal cold measurement, transient sensitivity of ice water into the cavity, diagnosis of deep caries. Processed as ( )

A. Amalgam filling

B. Zinc polycarboxylate cement-filling

C. Zinc polycarboxylate cement, silver amalgam filling

D. Vitreous ionic cement filling

E. Endodontic treatment

36.2013U2A1024 According to G · V · Black's cavity classification method, which of the following sentences is correct ( )

Class A. I holes are all single-sided holes

B. Class II holes are all double-faced holes

C. Class I cavity is a cavity prepared by posterior pit and fissure caries

D. Type III cavity is a cavity prepared by caries of the adjacent surface of the anterior tooth without damaging the incision

E. V. type hole is a hole prepared by the destruction of the posterior labial (buccal) and lingual (palatal) surfaces near the gingiva

37.2003U2A1029 Which of the following items is not required by the resistance form ( )

A. The cavity should be flat at the bottom, straight in the wall, clear and round and blunt

B. The depth of the cavity should reach 0.5 mm below the enamel dentin boundary

C. The shape of the hole is rounded and gentle

D. Use root canal piles or dentin nails

E. The angle of the dove tail gorge and the axis medullary line should not be on the same plane

38.2012U2A1089 The following is ( )

A. Dovetail

B. Inverted

C. Sidewall retention

D. Trapezoidal

E. All of the above

39. 2011U2A1021 In the preparation of the cavity, the following does not belong to the resistance design is ( )

A. Removal of baseless glaze

B. Removal of thin-walled weak tips

C. Steps for the preparation of the occlusal cavity

D. Preparation of trapezoidal shape for the occlusal cavity of the adjacent occlusal surface

E. The shape of the cavity is a gentle curve

40.2011U2A1022 The function of the compound hole to make a ladder does not include ( )

A. Dispersion of the tooth resultant

B. Less incision of tooth tissue

C. Conducive to filling

D. Protect the pulp

E. Resistance

41.2011U2A1023 The width of the dovetail gorge in the posterior teeth is the distance between the buccal tip of the tongue ( )

A. 2/3～1/2

B. 1/2

C. 1/3～1/2

D. 1/4～1/3

E. 1/5～1/4

1. D. Ladders. Reason: The stepped shape is often used to reinforce the structure, not as a resistance shape.
2. A. Prevent lateral dislocation of the restoration. Reason: The design of the occlusal cavity takes into account the stability of the restoration, making it less likely to move sideways when chewing.
3. B. Obtain a good resistance shape. Reason: Dovetails are designed primarily to provide enough resistance to prevent the filling from moving or falling off under chewing force.
4. C. Facilitates the restoration of adjacency. Why: The dovetail buckle is designed to help restore the correct contact between the teeth, ensuring function and aesthetics.
5. A. Cave walls, corners, edges, fixed shapes and resistance shapes. Why: All of these elements are considered in the standard cavity design to ensure the stability and durability of the filling.
6. B. Class II Caves. Causes: According to the Black classification, distal occlusal cavities are usually classified as Class II.
7. B. 14 adjacent to the cave. Causes: Type Black II cavities mainly involve caries lesions on multiple adjacent surfaces, and the adjacent cavities of tooth 14 fit this classification.
8. D. The entrance is small and the bottom is large. Cause: A larger base is not conducive to the formation of a resistance shape, as it may reduce the contact area between the filling and the tooth.
9. B. Make the filling and teeth able to withstand chewing force. Cause: The purpose of the resistance shape is to ensure that the filling is firmly bonded to the tooth and can withstand the chewing force together.
10. A. Isolate irritation and protect the pulp. Causes: The main purpose of the pad is to protect the pulp from heat, cold, or chemical irritation.
11. D. Walls and corners, edges, bottoms. Why: These are the basic structures that make up the hole and are crucial to the success of the filling.
12. A. Class II Caves. Causes: 47 buccal occlusal cavities are generally classified as Class II according to the Black classification.
13. C. The location of the caries and the characteristics of caverning. Causes: The Black classification is based on the location of decay and the morphology and characteristics of the pits.
14. D. Class V Cave. Causes: In the Black classification, neck caries is usually classified as class V.
15. C. 1.0～1.5mm。 Causes: Typical box-shaped holes should penetrate 1.0 to 1.5 mm deep into the dentin to ensure adequate strength and support.
16. D. Inward inclination of the cave wall. Reason: The box-shaped hole should be characterized by a vertical or slightly outward incline of the cave wall, rather than an inward inclination.
17. C. The bottom angle of the hole is clear. Reason: Resistant-shaped designs usually require smooth bottoms and line corners to reduce stress concentrations.
18. B. Class III Caves. Causes: The mesial neck hole of the posterior teeth is usually classified as Class III by Black.
19. D. Anterior tooth adjacent surface holes including climacto. Causes: Class IV holes usually refer to the adjacent faces of the anterior teeth, including the chamfered part.
20. C. Dovetail retention. Cause: Dovetail retention is a common retention method for posterior occlusal cavities to provide additional support and stability.
21. D. Dovetail buckles should be prepared. Reason: The basic characteristics of the box-shaped hole do not include the preparation of the dovetail buckle, which contradicts the requirements of the title.
22. A. Zinc oxide, clove oil, cement. Reason: Because of its good antibacterial and sealing properties, it is often the first choice as a temporary sealing material.
23. C. Anterior tooth proximal cavity does not include chamfers. Causes: Type III holes refer to the adjacent surface holes of the anterior teeth, but do not include the chamfered parts.
24. C. Dovetail retention. Causes: Dovetail retention is often used to increase the stability of the filling in the occlusal cavity of the posterior teeth.
25. A. Removal. Reason: The empty hanging glaze column should be removed during the repair process to avoid falling off after filling.
26. E. Precautionary expansion, composite resin filling. Why: In younger patients, the use of composite resin filling after a prophylactic extension can better protect the tooth and provide an aesthetic restoration.
27. E. Size of the adjacent defect. Reason: The design of the dovetail of the occlusal surface of Class II. cavity type mainly considers the size of the adjacent surface defect to ensure sufficient strength and support.
28. D. Type III cavity is a cavity prepared by caries of the adjacent surface of the anterior tooth without damaging the incision. Reason: This reflects the principle of the Black taxonomy, which states that type III caves refer specifically to the proximal cavities of the anterior teeth and do not involve chamfering.
29. A. Gingival walls are parallel. Cause: In the preparation of the facet hole, the medullary wall is parallel to the gingival wall to provide good support and force dispersion.
30. D. The edge of the cave is round and blunt. Cause: When preparing a deep cavity cavity, the edge line should be as sharp as possible to ensure that all decay is removed and crack propagation is prevented.
31. B. 1/4~1/3 of the buccal and lingual apical spacing. Reason: This is the usual ratio of the Havetail Gap width design and is designed to provide adequate support while maximizing tooth tissue preservation.
32. A. The angle of the medullary line of the axis of the cave floor is close to the midline. Why: This contributes to the stability and force dispersion of the filling.
33. E. All of the above: Reason: Trapezoidal retention can be used for many types of cavities because of its effective force dispersion.
34. B. Class II Caves. Cause: The cavities in the lower left 7 near the occlusal surface belong to Black's class II cavity.
35. C. Zinc polycarboxylate cement, silver amalgam filling. Causes: For deep caries, the pulp can be effectively protected by using a polycarboxylate cement, followed by an amalgam filling.
36. D. Type III cavity is a cavity prepared by caries of the adjacent surface of the anterior tooth without damaging the incision. Reason: This is in line with G · As defined by the V.· Black taxonomy, class III holes refer specifically to cavities adjacent to the anterior teeth and without chamfering.
37. D. Use root canal piles or dentin nails. Reason: The use of root canal piles or dentin nails is not directly related to the resistance shape, but more related to the design of the retention shape.
38. E. All of the above. Why: All of these options are part of the retained design to increase the stability of the restoration.
39. C. Steps for the preparation of the occlusal cavity. Reason: The ladder is designed primarily for retention, not resistance.
40. D. Protect the pulp. Reason: The main purpose of the staircase is to disperse the tooth force and protect the filling material, not to protect the pulp directly.
41. D. 1/4～1/3。 Reason: The width of the Dove Gap is usually designed between 1/4 and 1/3 of the buccal-lingual tip distance to provide proper support and preserve tooth tissue.

**Chapter 11 Application Basis of Dental Prosthetic Materials and Bonding Materials**

1.2010U2A1001 Bonding Mechanism of Etching Composite Resin ( )

A. Chelation

B. Chemical bonds

C. van der Waals

D. Mechanical bonding

E. Binding copolymerization

2.2010U2A1002 Full etching system refers to ( )

A. Preparations that can acidify enamel

B. Preparations that can acidify dentin

C. Preparations that can corrode cementum

D. Preparations that etch both enamel and dentin

E. Preparations that etch both dentin and cementum

3.2016U2A1040 Which term is wrong to describe acid etching ( )

A. Removes the surface layer of tarnished enamel and acts as a mechanical retention

B. Increase enamel surface free energy

C. It can enhance the chemical binding ability of enamel and filling material

D. Increase tooth surface area and roughness

E. Activation of the enamel surface

4.2012U2A1049 The main reason why dentin wet bonding can improve bond strength is ( )

A. Able to bond with intertubular dentin

B. Able to bond with peritubular dentin

C. Formation of resin processes chimeric with dentinal tubules

D. Forms a hybrid layer with the bulging collagen fiber network in dentin

5.2011U2A1016 The retention effect of the fourth-generation adhesive on dentin is produced by ( )

A. Micromechanical chimerism

B. Bonding

C. Chelation

D. Hydrogen bonding

E. van der Waals

6.2012U2A1062 Glass ionomer cement, as a filling material, is characterized by ( )

A. Tooth-colored material

B. Good abrasion resistance

C. Releasing fluorine

D. Limited operating time

E. Chemical binding to tooth tissue

7.2012U2A1085 The effect of acid etching does not include ( )

A. Remove the surface layer of tarnished enamel

B. Reduction of surface roughness

C. Increase enamel surface free energy

D. Activation of the enamel surface

E. Increase tooth surface area

8.2010U2A1086 Acid etching can not play a role ( )

A. Increase the surface area of the teeth

B. Mechanical cleaning action

C. Protect pulp viability

D. Expose clean and fresh enamel

E. A+B+D

9.2003U2A1047 The bonding method of dentin adhesive is mainly ( )

A. Mechanical bonding

B. Chemical bonding

C. Mechanical and chemical bonding

D. Retained cavity

E. None of the above

10.2003U2A1048 Bonding method between composite resin and enamel surface ( )

A. Mechanical locking bonding

B. Chemical bonding

C. Mechanical and chemical bonding

D. Retained cavity

E. Chelation

1. D. Mechanical bonding. Because the bonding of composite resins by etching method is mainly achieved by forming micropores in the enamel and tooth nature, increasing the mechanical mosaic force.
2. D. Preparations that etch both enamel and dentin. A full etching system refers to an acid etching agent that can etch both enamel and dentin.
3. C. It can enhance the chemical binding ability of enamel and filling material. Acid etching mainly increases the roughness physically, and does not directly enhance the chemical binding ability.
4. C. Formation of resin processes chimeric with dentinal tubules. Wet bonding technology enhances bond strength by keeping dentin slightly moist to facilitate the mating of resinous processes and dentinal tubules.
5. A. Micromechanical chimerism. Fourth-generation adhesives achieve dentin retention by forming a micromechanical chimerism.
6. C. Releasing fluorine. A major feature of glass ionomer cements is that they can release fluoride, which helps prevent secondary caries.
7. B. Reduction of surface roughness. The purpose of acid etching is to increase the surface roughness rather than decrease it in order to improve bond strength.
8. C. Protect pulp viability. Acid etching does not directly protect pulp viability, it mainly increases surface area and cleans.
9. C. Mechanical and chemical bonding. Dentin adhesives are usually enhanced by both mechanical and chemical bonding.
10. A. Mechanical locking bonding. The bonding of composite resins to the enamel surface is mainly achieved by mechanical locking.

**Chapter 12 Dental Bonding Restoration**

1.2011U2A1005 The following statement about the sandwich technique is correct ( )

A. The glass ions are mechanically mated to the hard tissue of the tooth

B. The ionic surface of the glass should be acid-etched

C. The binding mode of glass ions to composite resin is chemical bonding

D. Reduced the adhesion of the composite resin to the cave wall

E. All of the above are true

2.2003U2A1015 Which of the following is not the purpose of making an inclined plane of the cave wall when the composite resin is filled ( )

A. Increase the etching area

B. Increased Resistance

C. Increased retention

D. Reduce enamel cracks caused by polymerization shrinkage of composite resins

E. More aesthetically pleasing

3.2010U2A1059 When preparing the occlusal surface hole during resin filling, the hole edge should be formed ( )

A. Right angles

B. 45-degree incline

C. Acute angles

D. 60-degree inclined plane

4.2011U2A1040 Composite resin filling class V hole, the following design is correct ( )

A. Small and medium defects should be designed as conservatively as possible

B. Bevel only on the enamel wall

C. Defects with a large range should be prepared for retention

D. The design of the retained shape can be a retained groove

E. All of the above are true

5.2005U2A1053 The composite resin filling body is to widen the width of the enamel acid etching band, and the enamel wall of the hole edge should be made ( )

A. 30° angle short bevel

B. 45° angle short bevel

C. 60° angle short bevel

D. Inclined plane within a 135° angle

E. 120° angle within the inclined plane

6.2003U2A1046 Causes of Adhesive Restorations of Composite Resin Filling Materials, Except ( )

A. The tooth surface is not thoroughly cleaned

B. Acid-etched tooth surface contact with saliva

C. The composite resin filler is thinner

D. The tooth surface is not dry

E. Retainers were prepared

7.2002U2A1088 Notes should be paid to the preparation of composite resin filling hole shape ( )

A. The point line angle should be round and blunt, the enamel wall of the cave edge should be beveled, and the inverted concave should be arc-shaped

B. The bottom should be flat, the wall of the cave should be straight, and the shape of the cave must be deep

C. To remove the baseless enamel and increase the resistance shape

D. In order to obtain a good retention, normal tooth tissue must be removed

E. There is no need to remove baseless enamel or prepare short bevels at the edge corners

8.2003U2A1050 The reasons for impaction after filling are as follows, except for ( )

A. Touchpoints are not restored

B. Decay of adjacent teeth

C. Small abduction gap

D. Overhang of the filling body

E. Forming sheets are used for filling

· C。 The glass ions are chemically bound to the composite resin.

· E。 The main purpose of making inclined planes is not for aesthetics, but to enhance the resistance and retention of the structure.

· B。 When preparing occlusal cavities, it is usually recommended that the edge of the cavity be beveled at 45 degrees to enhance resistance and maintain aesthetics.

· E。 Class V cavities should be designed with conservative treatment of small to medium defects, beveled in both enamel and dentonature, and retained for large defects.

· B。 In order to widen the enamel acid etching band, the enamel wall of the cave margin should be made with a short bevel at an angle of 45°, which helps to increase the bonding area.

· E。 The retained shape is prepared to increase the stability of the filling material and is not the cause of the prosthesis to fall off.

· A。 The preparation should make the point line angle round and blunt, the enamel wall of the cave margin should be beveled, and the inverted concave should be arc-shaped to reduce the stress concentration.

· E。 The forming disc is used for filling to prevent overhang or overflow of the filling, so it does not cause impaction.

**Chapter 13 Deep Caries Treatment and Pulp Capping**

1. 2013U2A2135 The 10-year-old child had cold irritation and pain in the front teeth for several days. Examination: the right upper central incisor is near the middle and deep cavity, there is no abnormality on percussion, no loosening, and transient pain is measured coldly. Treatment: decomposing clean, very close to the pith, sensitive. What kind of treatment to choose ( )

A. Secondary derotting

B. Zinc oxide clove oil paste to soothe

C. Calcium hydroxide indirectly caps the pulp

D. Directly capping the medulla

E. Cutting of the living myel

2.2003U2A1025 In the process of hole preparation and filling, the measures to protect the pulp are ( )

A. High-speed drilling rigs must have cooling measures

B. Keep the pit dry when the slow mobile phone is derotted

C. Intermittent abrasion should be used to cut the tooth tissue

D. The bottom of the deep hole should be filled

E. Includes the above

3. 1999U2A2078 Patient female with pain and discomfort in her lower left lower back tooth when eating sweet and sour food. Diagnosis of deep caries. The preparation hole is sensitive, the caries can be exhausted, and the pulp is not penetrated, and ( ) should be selected during treatment

A. Indirect capping

B. Direct capping

C. Palliative treatment

D. Underfilling

E. Endodontic treatment

4.2012U2A2115 Patients with deep caries have more severe excitation pain, and the soft caries at the bottom of the cave can be completely cleaned, and the treatment method should be selected ( )

A. Double layer of bottom, complete the filling treatment at one time

B. Pulp opening and inactivation after local anesthesia, endodontic treatment

C. Tranquilization therapy is done first, and after the symptoms are eliminated at the follow-up visit in one to two weeks, then fill with a double layer

D. Perform a myelinotomy

E. Indirect capping of the medulla and double layer of the bottom to complete the filling treatment at one time

5.2015U2A2103 The patient requested treatment due to a hole in the upper anterior teeth, and there was no spontaneous pain. Check: left and right upper 1 near mid caries, sensitization, cold measurement of transient pain, removal of irritation can be relieved, heat measurement with control teeth. Diagnosis of deep caries and the best treatment of the tooth on the day is ( )

A. Zinc oxide clove oil paste to soothe

B. Ca(OH)₂ preparation for marrow protection, photosensitive resin filling

C. Photosensitive resin filling

D. Vitreous ionic cement filling

E. Endodontic treatment

6. The child was 10 years old, and the anterior teeth were cold and irritated and painful for several days. Examination: the right upper central incisor is near the middle and deep cavity, there is no abnormality on percussion, no loosening, and transient pain is measured coldly. Treatment: decomposing clean, very close to the pith, sensitive. What kind of treatment to choose ( )

A. Secondary derotting

B. Zinc oxide clove oil paste to soothe

C. Calcium hydroxide indirectly caps the pulp

D. Directly capping the medulla

E. Cutting of the living myel

7. 2013U2A2127 The patient was 18 years old, and during the treatment of other teeth, he found that the upper right 2 deformed lingual fossa, deep, cochin probe, and the temperature measurement was the same as that of the control tooth. The treatment of this tooth should be ( )

A. No treatment

B. Precautionary filling

C. Directly cap the medulla

D. Vital myelinotomy

E. Root canal treatment

8.2016U2A1027 pH of calcium hydroxide preparations used to cover the pulp ( )

A. 6.5～7.5

B. 8～9

C. 9～12

D. 12～14

E. 14

9.2016U2A2080 Reducible pulpitis of young permanent teeth, the preferred treatment for patients with deputy and no pulp exposed is ( )

A. Pulp opening and drainage under local anesthesia

B. Palliative treatment

C. Indirect capping of the medulla

D. Vital myelinotomy

E. Local anesthesia extraction

10.2004U2A1079 Pathological changes of the pulp after pulpectomy ( )

A. Calcification of the pulp

B. Intra-root canal absorption

C. Pulp viability is normal

D. Root pulp fixation fibrosis

E. Root canal orifice forms a hard tissue bridge

11.2012U2A1071 Reducible pulpitis caused by deep caries, when indirect pulp capping is used, the commonly used pulp capping agent is ( )

A. Calcium hydroxide

B. Zinc phosphate cohesive powder

C. Glass ionic cementing powder

D. Zinc oxide clove oil cement powder

E. Iodoform paste

12.2010U2A1085 For the tooth with accidental pulp penetration, what kind of pulp capping agent is generally used ( )

A. Calcium hydroxide

B. Zinc phosphate cement

C. Zinc oxide clove oil paste

D. Arsenic anhydride

E. Iodoform zinc oxide

· C. The binding mode of glass ions to composite resin is chemical bonding. The binding between glass ions and composite resins is mainly achieved by chemical bonding, not just mechanical chimerism.

· E. More aesthetically pleasing. The main purpose of the original bevel is to increase the etching area, increase resistance and retention, and reduce the cracks caused by polymerization shrinkage, and aesthetics are usually not the main consideration for making bevels.

· B. 45-degree incline. When preparing the occlusal surface cavity, the hole margin is generally made with a 45-degree inclined plane to increase the contact area and bond strength between the filling material and the tooth tissue.

· E. All of the above are true. When filling Class V holes with composite resins, consideration should be given to the size of the defect, conservatism, preparation of the retained shape, and treatment of the enamel wall, all of which are reasonable design considerations.

· B. 45° angle short bevel. Widening the width of the enamel acid etching band is usually achieved by making a shorter bevel, with a 45° angle bevel being a common design choice.

· C. Calcium hydroxide indirectly caps the pulp. The cold irritation and pain of the anterior teeth of the child indicates that the pulp is inflamed, but there is no sign of irreversible damage, so calcium hydroxide indirect pulp capping is a suitable choice.

· B. Precautionary filling. The deformed lingual fossa is deep and can be probed, suggesting a possible risk of caries development, and prophylactic filling can prevent further injury.

· C. 9～12。 The pH value of calcium hydroxide is generally between 9~12, which helps to inhibit bacterial growth and promote dentin formation.

· C. Indirect capping of the medulla. In reducible pulpitis, indirect pulp capping is preferred in the absence of pulp exposure to promote pulp self-repair.

· A. Calcification of the pulp. The pulp may become calcified after pulpotomy, resulting in loss of pulp viability.

· A. Calcium hydroxide. Calcium hydroxide is commonly used in indirect pulp capping because it is beneficial to stimulate the formation of new dentin and has a certain antibacterial effect.

· A. Calcium hydroxide. Calcium hydroxide is a commonly used pulp capping agent for tooth with accidental pulp perforation because it aids in dentin regeneration.

**Chapter 15 Etiology and Pathogenesis of Pulp Apical Disease**

1.2009U2A1092 Harmful substances produced by bacteria entering the pulp do not include ( )

A. Endotoxin

B. Collagenase

C. Amylase

D. Hyaluronidase

Chondroitinase sulfate

2. 2009U2A1100 The most common causes of pulp infection are ( )

A. Trauma

B. Periodontitis

C. Bloodborne infections

D. Caries disease

E. Dental malformations

3.2008U2A1048 If periapical lesions continue to worsen while receiving root canal treatment, the possibility of bacterial infection around the apical should be considered ( )

A. Porphyria

B. Prevotella

C. Actinomycetes

D. Eubacterium

E. Viron cocci

4. 2011U2A1012 Root canal and periapical infection is ( )

A. Isolated aerobic infection

B. Simple anaerobic infection

C. Co-infections predominantly aerobic infections

D. Co-infections with predominantly anaerobic infections

E. Co-infections of aerobic and anaerobic bacteria are equally important

5.2011U2A1014 Endodontic disease is caused by the infection route ( )

A. Retrograde infection of deep caries and periodontal disease

B. Periodontal disease retrograde infection, deep caries, and chemical irritation

C. Electrical stimulation, occlusal trauma, systemic chronic wasting systemic disease

D. Bloodborne infection, retrograde infection of periodontal disease, deep caries, or dental injury exposing the pulp

E. Trauma, developmental malformations, and accidental perforation

6.2012U2A1073 Physical factors causing endodontic diseases, except ( )

A. Cavity preparation process

B. Occlusal trauma

C. Cavity disinfection

D. Metal material filling

E. Dental trauma

7. 2010U2A1080 The endemic pathogen of pulp infection that occurs only in the infected root canal is ( )

A. Porphyria pulp

B. Porphyria gingivals

C. Clostridium

D. Digestive streptococcus

E. Viron cocci

8. 2011U2A1030 The main causes of pulpitis are ( )

A. Occlusal trauma

B. Chemical irritation

C. Physical stimuli

D. Bacterial infection

E. Allergic reactions

9.2002U2A1071 During the treatment of the root canal of infection, there is often a sudden outbreak of pain within minutes to hours after the enclosure of a certain drug, which may be due to ( )

A. Acute onset of apical outbreak infection

B. Root canal instrument damage to periapical tissues

C. Root canal instruments push bacteria around the apical to cause acute infection

D. Encapsulated drug acts as a hapten to elicit an acute immunoallergic reaction

E. Pulpitis occurs in the residual pulp

10.2006U2A1027 Causes of periapical tissue disease do not include ( )

A. Trauma

B. Chemical irritation

C. Infection

D. Electrical stimulation

E. Immune factors

11.2006U2A1028 The most important route of periapical infection is ( )

A. Deep periodontal pockets

B. Wedge defect

C. Abrasion

D. Accessory root canal

E. Deep caries

12.2000U2A1024 Factors leading to pulp necrosis of the affected tooth due to occlusal trauma are ( )

A. Physical factors

B. Chemical factors

C. Local infection

D. Immunity

E. Lesion infection

1. C. Amylase. This is because amylase is primarily involved in the digestive system and not in endodontic conditions.
2. D. Caries disease. Caries is the most common cause of pulp infection because cavities can provide a passage for bacteria to enter the pulp.
3. C. Actinomycetes. Because actinomycetes are one of the common refractory bacteria in periapical infections.
4. D. Co-infections with predominantly anaerobic infections. A common infection in the root canal system is a co-infection of anaerobic bacteria.
5. D. Bloodborne infection, retrograde infection of periodontal disease, deep caries, or dental injury exposing the pulp. All of these factors can lead to direct or indirect infection of the pulp.
6. C. Cavity disinfection. Cavity disinfection is done to remove bacteria and not the direct physical cause of endodontic disease.
7. A. Porphyria pulp. Specific pathogens are often associated with infections at specific sites.
8. D. Bacterial infection. Pulpitis is mainly caused by bacterial infections, especially bacteria that enter through cavities.
9. C. Root canal instruments push bacteria around the apical to cause acute infection. During treatment, bacteria may be pushed around the apex, leading to acute infection and pain.
10. D. Electrical stimulation. Electrical stimulation is not a common cause of periapical tissue disease.
11. E. Deep caries. Deep caries is the main route of pulp and periapical infection, as it can directly expose the pulp to bacteria.
12. A. Physical factors. Occlusal trauma is a physical factor that can lead to damage or necrosis of the pulp.

**Chapter 16 Clinical manifestations and diagnosis of endodontic diseases**

1.2009U2A1069 The clinical manifestations of pulp necrosis are ( )

A. Usually asymptomatic, but x-rays show intradental resorption

B. Teeth are dark, discolored, opaque, and have no response to temperature and electrical viability tests

C. Tooth discoloration, rot and odor in the pulp cavity, slow response to temperature and electrical viability test, lower reading than control tooth

D. No obvious spontaneous pain, which can be caused by temperature changes

E. Usually asymptomatic, x-ray shows uneven enlargement of the root canal

2.2003U2A1010 Which of the following is inappropriately described in the clinical classification of endodontics ( )

A. Pulp hyperemia

B. Nonreducible pulpitis

C. Pulp necrosis

D. Calcification of the pulp

E. Intradental resorption

3.2016U2A1030 Which item is not a characteristic of acute pulpitis pain ( )

A. Pain is worse at night than during the day

B. Cannot be located

C. Pain worsening with temperature stimulation

D. Spontaneous pain, paroxysmal exacerbation

E. The sitting position is heavier than the lying position

4.2016U2A1047 Pulpitis based on X-ray results as an important diagnostic basis is ( )

A. Reducible pulpitis

B. Acute pulpitis

C. Chronic pulpitis

D. Calcification of the pulp

E. Pulp necrosis

5. 2016U2A1050 The key points of diagnosis of reducible pulpitis are ( )

A. Complaints of transient sensitivity to temperature stimuli

B. No history of spontaneous pain

C. Dental lesions or periodontal tissue lesions that can cause pulp lesions

D. Decreased response domain values to pulp viability tests

E. All of the above

6.2016U2A1051 Acute pulpitis is characterized by pain ( )

A. Spontaneous pain, exacerbation of heat stimulation pain, reduction of cold stimulation pain, pain localization, percussion pain

B. Hot and cold stimuli, short duration of pain, no spontaneous pain

C. Spontaneous, sharp pain, paroxysmal episodes, intensifying pain with cold or hot stimuli, pronounced nocturnal pain, referred pain

D. Food impaction pain is severe, temperature irritation pain is obvious, and there is no obvious spontaneous pain

E. Persistent dull pain, bite pain, radiating pain

7. 2016U2A1053 The common clinical manifestations of chronic pulpitis at presentation are as follows, except for ( )

A. Bite pain, light percussion pain

B. Severe spontaneous pain

C. Pain of food embedding

D. Hot and cold irritation pain

E. Caries cavities can be probed to perforators

8.2009U2A1083 Reducible pulpitis is distinguished from the serous phase of acute pulpitis by ( )

A. No temperature irritation

B. No chemical irritation pain

C. No spontaneous pain

D. No probing pain

E. No percussion

9.2009U2A1102 Pain characteristics of acute pulpitis do not include ( )

A. Spontaneous, paroxysmal pain

B. Pain exacerbated by temperature stimulation

C. Nocturnal pain

D. Pain cannot be localized

E. Pain is loose or referential, sometimes to the contralateral area of the affected tooth

10.2008U2A1040 The following types of diseases can be pointed out in the pain area, except ( )

A. Acute apical abscess

B. Acute papillitis gingivals

C. Acute pulpitis

D. Dentin sensitivity

Acute periodontal abscess

11.2008U2A1043 Pain characteristics of chronic pulpitis ( )

A. Severe spontaneous pain

B. Severe nocturnal pain

C. Referred pain

D. Mild spontaneous dull pain

E. A+B+C

12.2008U2A1044 The most valuable diagnostic indication for residual myelitis is ( )

A. X-rays

B. Temperature Stimulation Test

C. Electrical test

D. Remove the original supplement to probe for sensation or pain deep at the root of the affected tooth

E. Percussion

13.2008U2A1049 The following are not reducible pulpitis manifestations ( )

A. Food embedding pain

B. Hot and cold irritation pain

C. Sweet and sour irritation

D. Tooth defects close to the pulp tissue

E. Spontaneous pain

14.2008U2A2079 A 32-year-old patient presents with pain in his left lower posterior tooth. The lower left 6 occlusal surfaces showed mercury filling, mild pain on percussion, flaccid pain with cold stimulation, X-ray showed that the filling had reached the pulp cavity, no root filling image, and the apex was normal. Diagnosed as ( )

A. Chronic apical periodontitis

B. Reducible pulpitis

C. Myelitis remnant

D. Deep caries

E. Acute pulpitis

1. B。 Pulp necrosis is usually asymptomatic, but darkening, discoloration, opacity, and failure to respond to temperature and electrical viability tests are typical clinical manifestations of pulp necrosis.
2. E。 Intradental resorption is not a classification of endodontic disease, but a possible consequence of endodontic disease.
3. E。 Sitting heavier than lying down is not a feature of acute pulpitis pain, but is characteristic of some other types of toothache or headache.
4. E。 The diagnosis of pulp necrosis is largely based on x-rays, as clinical symptoms may not be obvious.
5. E。 All of the above options are essential points for the diagnosis of reducible pulpitis.
6. C。 Acute pulpitis is characterized by spontaneous, sharp pain, paroxysmal episodes, increased pain with cold or heat stimuli, pronounced night pain, and referred pain.
7. B。 Chronic pulpitis presents with uncommon severant, spontaneous pain.
8. C。 One of the differences between reducible pulpitis and the serous phase of acute pulpitis is that reducible pulpitis does not have spontaneous pain.
9. D。 The pain characteristics of acute pulpitis are usually localizable, whereas loose or referred pain means that the pain may radiate to other areas, which does not contradict pain localization.
10. C。 Pain localization in acute pulpitis is ambiguous because the pain may be loose or involve other areas.
11. D。 Chronic pulpitis is usually characterized by a mild, spontaneous, dull ache.
12. D。 The most valuable diagnostic indication for residual myelitis is to explore deep within the root of the affected tooth for sensation or pain after removing the original filling.
13. E。 Reducible pulpitis usually does not have spontaneous pain.
14. B。 According to the narrative, the patient's symptoms are consistent with those of reducible pulpitis, which is cold stimulation and flaccid pain, and x-rays are unremarkable.

**Chapter 17 Clinical manifestations and diagnosis of periapical disease**

1.2015U2A1012 Diseases that must be diagnosed by X-ray examination are ( )

A. Occlusal caries

B. Acute pulpitis

C. Chronic pulpitis

D. Acute apical periodontitis

E. Chronic apical periodontitis

2. When the periapical transmission area of the 2013U2A1018 X-ray film includes several teeth, the main basis for confirming the pathogenic tooth is the affected tooth ( )

A. Whether there is periodontal disease

B. Presence or absence of sinus tracts

C. Pulp viability

D. Presence or absence of cavities

E. Presence or absence of percussion

3.2016U2A1036 What stage of acute apical periodontitis in clinical practice has the highest pain ( )

A. Acute serous apical periodontitis

B. Acute periapical abscess

C. Acute subperiosteal abscess

D. Acute submucosal abscess

E. Osteitis compact

4.2016U2A1038 According to the typical natural development process of acute purulent apical periodontitis, the clinically visible purulent drainage route does not include ( )

A. Drainage of pus from the crown defect through the root canal from the apical foramen

B. Perforate the bone wall and break through the mucosa to drain pus

C. Perforate the bone wall and break through the skin to drain pus

D. Drainage of pus through the maxillary sinus wall

E. Breakthrough of the nasal floor mucosa to drain pus

5.2016U2A1039 The best auxiliary examination method for chronic apical periodontitis is ( )

A. Percussion

B. Electro-test of pulp viability

C. X-rays

D. Bite diagnosis

E. Transillumination

6.2016U2A1043 What kind of apical periodontitis is common in clinical practice ( )

A. Serous phase of acute apical periodontitis

B. Acute apical periodontitis suppurative phase

C. Apical abscess

D. Acute apical periodontitis

E. Chronic apical periodontitis

7.2008U2A1055 Which is the most common method of purulent drainage in the typical purulent route of acute purulent apical periodontitis ( )

A. Perforating the bone wall and breaking through the mucosa

B. Penetrate the bone wall and break through the skin

C. Drainage through the apical foramen through the root canal

D. Drainage of pus through the maxillary sinus wall

E. Breakthrough of the nasal floor mucosa to drain pus

8. Typical local symptoms of patients with acute subperiosteal abscess in patients with 2000U2A1019 are ( )

A. Degree III loosening, percussion pain (+++) of the affected tooth

B. Red and swollen gingiva at the apex, red and swollen gingiva at the root tip, and painful gingiva

C. Regional lymphadenopathy is swollen and tender

D. Migration groove becomes shallower and deep fluctuations

E. Corresponding facial soft tissue reactive edema

9.2000U2AN134 male, 32 years old. On the 2nd, I had a toothache in the lower right back and could not bite anything. Half a year ago, I found that there was a small bag in the lower right posterior gum, and there was no obvious discomfort at ordinary times. Examination showed deep caries in the distal and median face of the upper right 6, deep caries in the upper left 1, no sensation in the perforation of the medullary hole, percussion (±), there was a fistula opening in the gingiva at the apex of the lower right 8 and 7 lower right sides, and a little pus was squeezed. The lower right 8 anterior leaning impaction, the gingiva is red and swollen obviously, and there is pus in the deep part of the gingival pocket. X-ray showed that the X-ray transmission area around the distal apical area of the upper right 6 was irregular and the boundary was blurred. The diagnosis of the tooth causing the presenting symptom is acute wisdom tooth pericoronitis, which is the source tooth of the gingival fistula and should be done ( )

A. Fistula probing

B. Periodontal pocket probing

C. X-rays

D. Fistula diagnostic silk x-ray

E. Periodontal pocket diagnostic silk X-ray

10.2000U2AN136 male, 14 years old, maxillary gums often bleed pus for many days. Deep caries was examined, no perforation of the medullary hole was detected, loose II. degree, percussion (±), temperature measurement was unresponsive, and there was a fistula at the apex of the labial side of the affected tooth. To confirm the diagnosis, the tests that need to be done are ( )

A. Electrical vitality test

B. Smear test

C. Fistula examination

D. X-rays

E. Puncture

11.2000U2AN143 male, 36 years old. 2 weeks ago, I found that there was a small bag in the gums of the right lower back teeth, and there was no obvious discomfort at ordinary times. Examination showed deep caries on the occlusal surface of the right lower first molar, no sensation through the medullary foramen, percussion (±), and a fistula opening in the gingiva at the root apex of the right lower second molar, and a little pus was squeezed out. X-rays showed that the transmission area of the proximal apical apex of the right lower first molar was irregular and the boundary was blurred. The presenting tooth should be diagnosed as ( )

A. Chronic pulpitis

B. Chronic alveolar abscess

C. Apical cyst

D. Apical granulomas

E. Chronic periodontitis

12.2000U2AN144 male, 36 years old. 2 weeks ago, I found that there was a small bag in the gums of the right lower back teeth, and there was no obvious discomfort at ordinary times. Examination showed deep caries on the occlusal surface of the right lower first molar, no sensation through the medullary foramen, percussion (±), and a fistula opening in the gingiva at the root apex of the right lower second molar, and a little pus was squeezed out. X-rays showed that the transmission area of the proximal apical apex of the right lower first molar was irregular and the boundary was blurred. To confirm the diagnosis of a tooth with gingival fistula, ( )

A. Fistula probing

B. X-rays

C. Periodontal pocket probing

D. Fistula diagnostic silk x-ray

E. Periodontal pocket diagnostic silk X-ray

13.2012U2A1064 The following diseases need to be diagnosed by X-ray examination, but excluding ( )

A. Acute apical periodontitis

B. Adjacent caries and secondary caries

C. Intradental and external resorption

D. Chronic apical periodontitis

E. Myeloliths and diffuse calcification

14.2013U2AN145 male, 35 years old. Due to the swelling and pain of the lower right back tooth for 5 days, I came to the doctor today with general discomfort. The patient's painful face was examined, and the right cheek was swollen more obviously. The distal neck caries of the lower right first premolar is deeply perforated with medulla, no probing pain, third-degree loosening, percussion pain (+++), gingival redness and swelling are obvious, and the transition groove is flattened. Tests that should also be done for the patient are as follows, with the exception of ( )

A. Take your temperature

B. Pulp temperature test

C. Check the white blood cell count

D. X-rays

E. Palpation of submandibular lymph nodes

1. E. Chronic apical periodontitis. Causes: Chronic apical periodontitis is often accompanied by bone destruction at the apex, which should be visualized on x-rays.
2. C. Pulp viability. Causes: The loss of pulp viability directly affects the occurrence of periapical lesions, which is an important basis for diagnosis.
3. B. Acute periapical abscess. Causes: The acute periapical abscess stage is most painful due to inflammation involving abundant nerve endings.
4. C. Perforate the bone wall and break through the skin to drain pus. Causes: Intraoral infections usually do not break directly through the skin to drain pus, which is an atypical route of purulent drainage.
5. C. X-rays. Causes: X-rays can clearly show the bone destruction characteristic of chronic apical periodontitis.
6. E. Chronic apical periodontitis. Causes: Clinically, chronic apical periodontitis is more common due to its slow course and frequent occurrence.
7. A. Perforating the bone wall and breaking through the mucosa. Causes: The most common mode of drainage in acute purulent apical periodontitis is through the bone wall through the mucosa.
8. C. Regional lymphadenopathy is swollen and tender. Causes: Acute subperiosteal abscesses often cause regional lymph node reactions, including enlargement and tenderness.
9. D. Fistula diagnostic silk x-ray. Causes: Using fistula diagnostic wires and taking x-rays can help determine the origin of the fistula and the source of the tooth.
10. D. X-rays. Causes: X-rays can help determine the condition of the affected tooth and the extent and nature of the lesion.
11. C. Apical cyst. Causes: Long-term apical inflammation can lead to the formation of apical cysts, which manifest clinically as fistulas and X-ray transmission areas.
12. B. X-rays. Causes: X-rays can help determine the exact location of the fistula and the extent of the lesion to diagnose the source tooth.
13. B. Adjacent caries and secondary caries. Causes: Diagnosis of adjacent and secondary caries is usually based on clinical examination rather than relying solely on x-rays.
14. B. Pulp temperature test. Causes: In acute cases, pulp temperature testing is not as necessary as other tests, such as X-rays or palpation of lymph nodes.

**Chapter 18 Treatment plan for periapical endodontic disease**

1.2015U2A2127 Female, 60 years old. Left upper back teeth. Severe spontaneous pain for 2 days. There is release pain. Percussion (+), probing of deep cavities, perforation of medullary holes, probing pain (+++). No loosening, periodontal examination (-). The best emergency management is ( )

A. Palliative treatment

B. Taking anti-inflammatory painkillers

C. Pulp opening and drainage

D. Acupuncture analgesia

E. Incision to drain pus

2. 2015U2AN139 A 55-year-old male patient presented with a painful face, complaining of severe pain in the right upper back tooth and not daring to bite and touch the pain. It was found that the gingiva in the upper right 6 was swollen relative to the apical area, the transitional sulcus became shallow, and there was a deep fluctuation sensation on palpation. The upper right 6 percussion (+++) is loose III. degree, which can probe the pulp cavity, and there is no response, the upper right 7 teeth are intact, and the buccal side can detect the root bifurcation, which cannot be penetrated, and percussion (++) is loose III. degree. The right cheek is swollen and tender to the touch. then it should be dealt with first as ( )

A. Intravenous antibiotics

B. Incision to drain pus

C. Removal of decayed tissue

D. Adjust the occlusion

E. A+B

3. 2013U2A2068 patient, male, 24 years old, complained of cold and hot irritation pain in the right lower back tooth for 3 days at night. On examination, the lower right third molar is impacted in the near middle and low position, there is more food impaction between the lower right 78, the lower right 8 does not detect caries, and the distal blind pocket is slightly red and swollen, and it is painful to touch. Cold (-). Detection of caries in the distal neck of the lower right 7 is profound, deep, soft, and cold stimulation causes severe pain. The best treatment for the first visit is ( )

A. Remove the lower right 8

B. Circumcoronal irrigation of the lower right 8 crowns

C. Lower right 7 open and drainage

D. Lower right 7 after derotting ZOE appeased

E. Intravenous broad-spectrum antibiotics

4.2003U2A1011 Seal deactivator should be noted ( )

A. Do not push the inactivator when temporarily sealing the cavity

B. Inactivator is placed in the perforamen

C. Cavities located under the gums can be sealed in layers

D. Attention should be paid to the dryness of the cavity environment before sealing the medicine near the gums

E. Attention should be paid to all of the above

5.2004U2A1073 Pulp inactivation is generally used ( )

A. Paraformaldehyde inactivator

B. Metal arsenic inactivators

C. Arsenite inactivator

D. A or B

E. A, B, C are acceptable

6.2016U2A1026 Which of the following is NOT the principle of emergency treatment of acute apical periodontitis ( )

A. Anti-inflammatory and analgesic

B. Palliative treatment

C. Adjust the teeth and relieve pain

D. Extraction of the affected tooth

E. Establish drainage

7.2016U2A1042 The most effective emergency treatment for acute pulpitis is ( )

A. Anti-inflammatory and analgesic

B. Palliative treatment

C. Pulp opening and drainage

D. Adjustment and abrasion

E. Acupuncture analgesia

8.2016U2A1052 Emergency treatment of acute apical periodontitis is correct ( )

A. Under local anesthesia

B. Open the medullary cavity and drain the root canal

C. Perforation through the apical hole

D. Incision and drainage of abscess if there is an abscess

E. All of the above

9.2009U2A1088 In the treatment of acute pulpitis, the first question that should be considered is ( )

A. Anti-inflammatory

B. Destroy pathogenic bacteria

C. Painless or minimized

D. Prevent the spread of infection

E. Protect the living pulp

10.2009U2A1091 The most effective treatment for submucosal abscess in acute apical inflammation is ( )

A. Take anti-inflammatory painkillers

B. Drainage

C. Incision to drain pus

D. Adjustment and abrasion

E. Acupuncture for pain relief

11. 2008U2A1052 Which point is wrong about the treatment of endodontic disease and periapical disease ( )

A. During root canal treatment, it is not advisable to seal the medicine repeatedly

B. Acute pulpitis with severe pain Oral painkillers are difficult to work

C. Calcium hydroxide preparations can also be used for root canal disinfection

D. There is no need to wait until all symptoms are gone before doing a root canal filling

E. Endodontic disease and periapical disease are mostly caused by bacterial infection, so aseptic procedures can be de-emphasized in treatment

12.2000U2AN142 male, 40 years old. In the past six months, the left upper back tooth has encountered hot and cold pain, but there is no spontaneous pain, and in the past 1 month, in addition to cold and heat allergies, there is a dull pain. Spontaneous pain in the past 1 week, worsening paroxysmal episodes, nocturnal pain. Examination revealed that the left upper second molar had deep caries on the mesial occlusal surface, and the pain was evident and percussion (±). The effective treatment of the presenting tooth is ( )

A. Anti-inflammatory and analgesic

B. Topical application

C. Caries cavity filling

D. Endodontic treatment

Periodontal treatment

13.2013U2A2131 A 25-year-old patient with poor oral hygiene, the first permanent molar of the left lower left had a pulp-periodontal joint lesion, and the pain was severe, but the affected tooth was not loosened. The first measures to be taken to eliminate the pain and restore health ( )

A. Early filling

B. Open and drain to stop the spread of inflammation

C. Anti-inflammatory and analgesic

D. Promote periodontal soft tissue health

Use of fluoride

14.2012U2A1066 Pulp Plasticization Treatment, the requirements for pulp extraction are ( )

A. Retain 1/3 of the pulp at the apex

B. Extract the pulp as much as possible

C. Puncture the apical hole

D. The pulp can be removed without extraction

E. Retain the pulp at the apical 2/3

1. C. Pulp opening and drainage. Because the patient has severe pain and perforation, urgent treatment is required to relieve symptoms.
2. B. Incision to drain pus. Patients have significant swelling and a deep fluctuating sensation, suggesting abscess formation that requires drainage to alleviate symptoms.
3. C. Lower right 7 open and drainage. Because patients present with nocturnal pain and sensitivity to cold stimuli, there may be pulp inflammation and open and drainage may relieve symptoms.
4. E. Attention should be paid to all of the above. All of the listed precautions are important when using closure deactivators.
5. E. A, B, C are acceptable. Although arsenic-containing deactivators are not recommended now, these have historically been used as pulp inactivators.
6. C. Adjust the teeth and relieve pain. The principles of emergency treatment of acute apical periodontitis are mainly anti-inflammatory, analgesic, and drainage, and occlusion is not the preferred emergency treatment.
7. C. Pulp opening and drainage. The most effective treatment for acute pulpitis is to deal directly with the endodontics, and open and draining the pulp can provide immediate relief of symptoms.
8. E. All of the above. Management of acute apical periodontitis includes local anesthesia, opening of the medullary cavity drainage, and, if necessary, incision and drainage of pus.
9. C. Painless or minimized. When treating acute pulpitis, the primary goal is to reduce the patient's pain.
10. C. Incision to drain pus. For acute apical inflammation in the submucosal abscess phase, the most effective treatment is direct incision and drainage to relieve symptoms.
11. E. Endodontic disease and apical periapical disease are mostly caused by bacterial infections, so aseptic procedures can be de-emphasized in treatment. This is wrong, as aseptic manipulation must be emphasized during treatment to avoid cross-contamination.
12. D. Endodontic treatment. Because patients have long-term symptoms and pulp exposure, endodontic therapy is an effective treatment.
13. B. Open and drain to stop the spread of inflammation. Because the pain is severe, the endodontic problem needs to be treated immediately to alleviate the symptoms.
14. B. Extract the pulp as much as possible. When performing endoplasty treatment, it is usually necessary to remove the pulp tissue as much as possible to avoid inflammation or infection.

**Chapter 19 Root Canal Therapy**

1.2017U2A1015 The following statements about root canal treatment are incorrect ( )

A. X-rays were taken before surgery to understand periapical lesions and root resorption

B. Remove the entire pulp under local anesthesia or after the pulp is inactivated

C. It is suitable for the affected tooth whose pulp inflammation involves the root pulp and is not suitable for pulpectomy

D. Remove the necrotic pulp tissue in the pulp chamber and root canal, and file the root canal appropriately

E. Rinse and sterilize the root canal thoroughly, using 5% hydrogen peroxide solution

2.2016U2A1055 Which treatment is best for juvenile apical periodontitis ( )

A. Dry myeloid surgery

B. Root canal treatment

C. Plastinization therapy

D. ATC therapy

E. Palliative treatment

3. 2000U2AN137 male, 14 years old, maxillary gums often bleed pus for many days. Deep caries was examined, no perforation of the medullary hole was detected, pine II.°, percussion (±), temperature measurement was unresponsive, and there was a fistula at the root apex of the labial side of the affected tooth. Clinical treatment should be used ( )

A. Cutting of the living myel

B. Plastination therapy

C. Open drainage

D. Root canal treatment

E. Dry myeloid surgery

4. 2000U2AN140 male, 60 years old, has a right upper posterior toothache for one year, which mostly occurs in the evening and involves the right lower orbit and temporal region. The patient had a history of headache and runny nose several years ago. Check: deep caries on the upper right 5 distal occlusal surfaces and 6 upper right occlusal surfaces, percussion pain (+), palpital pain (-), upper right 7 and upper right 4 crowns intact, percussion pain (-), upper right 8 stubs. The most important thing in the treatment design of the chief complaint disease is ( )

A. Bottom filling

B. Root canal treatment

C. Endomedulla therapy

D. Root uprooting

E. ENT visits

5. 2000U2AN145 male, 36 years old. 2 weeks ago, I found that there was a small bag in the gums of the right lower back teeth, and there was no obvious discomfort at ordinary times. Examination showed deep caries on the occlusal surface of the right lower first molar, no sensation through the medullary foramen, percussion (±), and a fistula opening in the gingiva at the root apex of the right lower second molar, and a little pus was squeezed out. X-rays showed that the transmission area of the proximal apical apex of the right lower first molar was irregular and the boundary was blurred. The treatment of the complaining tooth is ( )

A. Filling therapy

B. Plastination therapy

C. Root canal treatment

D. Apical surgery

E. Uprooting

6.2013U2A2126 The patient did not dare to bite due to wedge-shaped defect, after being filled with composite resin, the cold and heat stimulated the pain. Check: 456 buccal composite resin filling on the upper left, 46 cold pain on the upper left, which can be relieved after removal, 5 percussions (+) on the upper left, obvious pain in hot and cold measurement, and lasting for a period of time after removal. The principle of handling the upper left 5 should be ( )

A. No treatment, observation

B. Desensitization therapy

C. Remove the old backfill and refill it

D. Remove the old filler, soothe with zinc oxide clove oil paste

E. Endodontic treatment

7. 2013U2A2129 The patient presented with cold and hot pain in the left tooth. Check: 6 severe uneven wear in the lower left, no perforation of the pulp hole, pain caused by thermal measurement and lasted for 2 minutes, percussion (-), the treatment principle of the affected tooth is ( )

A. Ask the patient to eat a small hard diet

B. Desensitization

C. Adjust the occlusion

D. Endodontic treatment

E. Full crown restoration

8.2014U2A1043 Which of the indications for root canal treatment is not exact ( )

A. Pulpitis of all kinds

B. Pulp necrosis

C. Various types of apical periodontitis

D. Apical periodontitis of deciduous and permanent teeth

E. Pulp periodontal disease

9.2014U2A1044 Observation time of postoperative efficacy evaluation of root canal therapy is ( )

A. Half a year

B. 1 year

C. 2 years

D. 3~5 years

E. More than 5 years

10.2003U2A1012 In root canal treatment, the instruments used for rotation should not be rotated more than ( )

A. 45°

B. 90°

C. 180°

D. 270°

E. 360°

11.2011U2A2081 Root canal treatment but periapical lesions are worsening, which of the following bacteria should be considered ( )

A. Actinomycetes

B. Fusobacterium

C. Peptic streptococcus

D. Melanin-producing Pruvotea

E. eubacterium

1. E。 The use of a 5% hydrogen peroxide solution may not be standard practice, and sodium hypochlorite solution is usually used to flush the root canal.
2. B。 Juvenile apical periodontitis is best treated with a root canal approach because it removes the inflamed tissue and saves the tooth.
3. D。 Fistulas and pus drainage are typical symptoms of apical periodontitis, and root canal treatment is the appropriate option.
4. B。 Because patients have toothache and deep caries, root canal treatment is probably the most important way to deal with this condition.
5. C。 Fistulas and deep caries indicate periapical lesions that require root canal treatment.
6. C。 Because the patient is sensitive to hot and cold stimuli, which may be a problem with the filling material or a root canal, removing the old filling and refilling it is an appropriate course of action.
7. D。 Because the persistent pain caused by the heat test indicates inflammation of the pulp and requires endodontic treatment.
8. E。 Endodontic-periodontal disease may not be a direct indication for root canal therapy.
9. A。 The efficacy after root canal treatment is usually assessed within six months to confirm the success of the treatment.
10. E。 The instrument used for rotation should not be rotated more than 360° to prevent breakage.
11. A。 Worsening periapical lesions may be associated with actinomycete infection, as it often leads to worsening periapical lesions.

**Chapter 21 Root Canal Preparation and Disinfection**

1.2010U2A1087 The statement about calcium hydroxide preparations is incorrect ( )

A. It is strongly alkaline

B. Has anti-inflammatory and antibacterial properties

C. Promote restorative dentin formation

D. Conducive to the recovery of pulp function

E. Insoluble in saliva, the solubility is the smallest among the substrate materials

2.2003U2A1014 Which of the following statements is incorrect about H file ( )

A. The cross-section is comma-shaped

B. Suitable for loose root canal preparation

C. The cutting edge is nearly perpendicular to the root canal wall

D. Rotation and lifting movements can be used during operation

E. Easier to break

3.2016U2A1046 In general, the main file should be larger than the primary file ( )

A. 1 device number

B. 3 device numbers

C. 4 instrument numbers

D. 5 instrument numbers

E. Arbitrary

4.2004U2A1078 Root surface chemical treatment, the most commonly used drug is ( )

A. 37% phosphoric acid

B. 10% citrate

C. 20% phosphoric acid

D. 10% acetic acid

E. 10% hydrochloric acid

5. 2010U2A1078 Which is wrong about root canal preparation ( )

A. Use reaming files and reaming drills, from fine to coarse, in order of serial number

B. The root canal mouth can be enlarged by ball drill, large reamer drill or crack drill

C. Root canal stenosis, calcification, or foreign bodies in the root canal can be treated with the chelating agent ethylenediaminetetraacetic acid

D. All instruments used for rotation should not be rotated more than half a turn

E. A+C+D

6. 2011U2A1034 The following statements about root canal irrigation fluid are incorrect ( )

A. The alternating use of 5.25% sodium hypochlorite and 3% hydrogen peroxide can produce new ecological oxygen and have foaming effect

B. Sodium hypochlorite is a good solvent for necrotic tissue

C. EDTA is effective for enlarging, hardening, or bending root canals

D. Fluids can provide lubrication

E. Sodium hypochlorite solution is selected for root canal irrigation with root hypoplasia

7. 2011U2A1035 The most commonly used root canal disinfection method in clinical practice is ( )

A. Laser treatment

B. Microwave therapy

C. High-frequency therapy

D. Electrolysis therapy

E. Drug disinfection

8.2002U2A1079 The depth of root canal preparation should stop at the cementum boundary of dentin, usually about ( ) from the apex

A. 0.5mm

B. 1mm

C. 1.5mm

D. 2mm

E. 1/3 of the apex

9.2006U2A1043 With the increase of age, the working length of the root canal continues ( )

A. Reduction

B. Additions

C. No change

D. Can be reduced, can be increased

E. All of the above are possible

10.2000U2A1030 The purpose of mechanical preparation of root canal is as follows, except ( )

A. Removal of infection in the main root canal

B. Removal of infection from the root canal wall

C. Enlarge the apical foramen to facilitate drainage

D. Reduce the curvature of the bent root canal

E. Prepare root canal morphology to facilitate filling

1. D。 Reason: Calcium hydroxide preparation is not directly conducive to the recovery of pulp function, it is mainly used to promote the formation of dentin and has anti-inflammatory and antibacterial effects.
2. E。 Reason: H files are stiffer instruments and are made and designed to break when operated, especially in narrow or crooked root canals.
3. A。 Reason: The primary tip file is usually 1 device size larger than the primary tip file to gradually increase the diameter of the root canal.
4. B。 Reason: The commonly used drug for root chemical treatment is 10% citrate, which is used to remove smoky stones and residues from the root canal wall.
5. D。 Reason: For instruments used in rotation, especially nickel-titanium instruments, the recommended angle of rotation is usually less than or equal to 360 degrees to reduce the risk of breakage, rather than no more than half a turn.
6. C。 Reason: EDTA is mainly used to remove inorganic components in the root canal, and is not directly effective in expanding hardening or bending the root canal, its main role is to chelate calcium ions.
7. E。 Reason: The most commonly used root canal disinfection method in clinical practice is drug disinfection because it is simple, effective, and inexpensive.
8. B。 Causes: The depth of root canal preparation should be stopped at a distance of about 1 mm from the root apex to reduce the risk of apical perforation.
9. A。 Causes: As we age, the working length of the root canal may decrease due to wear and tear and physiological deposition of the cementum.
10. D。 Reason: The purpose of mechanical preparation does not include reducing the curvature of the curved root canal, but rather to remove infection, enlarge the apical foramen to facilitate drainage, and to create a suitable root canal morphology to facilitate filling.

**Chapter 27 Medical History Collection and Clinical Examination**

1.2013U2A2116 Male, 50 years old, 1 week old right back tooth bite discomfort, cold water caused pain. In the past 4 days, night pain has affected sleep and caused pain in half of the head and face, and the pain cannot be located. On examination, it was found that both the upper and lower first molars on the right side had occlusal cavities. If the jaw position of the affected tooth cannot be determined after examination, ( )

A. Bite

B. Anesthetic test

C. Temperature test

D. Periodontal pocket probing

E. X-rays

2.2016U2A1029 The temperature of the thermal stimulus should be higher than ( )

A. 20℃

B. 40℃

C. 30℃

D. 60℃

E. 50℃

3. 2009U2A1082 When diagnosis of caries, the result of the normal tooth surface temperature test is ( )

A. Normal

B. Transient sensitivity

C. Sensitive

D. Sluggishness

E. No Response

4.2009U2A1090 The most reliable examination method for judging pulp viability is ( )

A. Temperature test

B. Electrical test

C. Selective anesthesia

D. Experimental cave preparation

E. X-rays

5.2008U2A1038 The following contraindications to the electroviability test of the pulp are ( )

A. Hyperthyroidism

B. Coronary heart disease

C. High blood pressure

D. Pacemakers

Epilepsy

6.2008U2A1045 Causes of false negative reaction of electron pulp viability test, excluding ( )

A. Use of anesthetic prior to testing

B. Newly erupted tooth whose root tip is not fully developed

C. Teeth that have undergone pulptomy

D. Teeth with excessive calcification in the root canal

E. Teeth that have just been traumatized

7.2008U2A1057 Electro-endodontic viability test is commonly used in diagnosis ( )

A. Calcification of the pulp

B. Inflammation of the pulp

C. Acute apical periodontitis

D. Pulp necrosis

E. Chronic apical periodontitis

8.2012U2A1063 Precautions for tooth temperature test are as follows, except ( )

A. Measure the affected tooth first

B. Thermal temperature is above 60°C

C. Isolate saliva

D. Xiaoice sticks for cold measurement

E. Test in the middle 1/3 of the labial (buccal) or tongue (palatal) surface of the teeth

9. 2012U2A1078 The cause of the false positive reaction of the electrododontic viability test is correct ( )

A. The probe is in contact with a large area of metal restoration

B. The test tooth is not adequately insulated from moisture or dryness

C. The pulp has liquefaction necrosis

D. Patient is overly nervous and anxious

E. All of the above

10.2013U2A1022 False reaction may occur in the electroviability test of the pulp, and the cause of the false positive reaction does not include which of the following ( )

A. The probe or electrode touches a large area of metal restoration or gum, allowing the current to flow to the periodontal tissue.

B. Inadequate moisture isolation or drying of the test tooth to leak into the periodontal tissue.

C. Liquefied necrotic pulp has the potential to conduct an electric current around the apex, and the patient may have a slight reaction when the current is adjusted to the maximum scale

D. The patient is so nervous and anxious that he or she responds when the probe first touches the tooth surface or when asked about his feelings.

E. The patient has used analgesics, anesthetics, or alcoholic beverages beforehand and cannot perceive electrical stimuli normally.

1. E. X-rays. Causes: X-rays can provide detailed information about the tooth and its surrounding tissues when the jaw position of the affected tooth cannot be determined by routine examination.
2. B. 40℃。 Reason: Thermal stimulation tests typically use temperatures close to or slightly above body temperature, and 40°C is an option that meets this criteria.
3. A. Normal. Causes: The tooth surface of a tooth decay usually shows a normal response on a temperature test, as this mainly detects the response of the pulp.
4. B. Electrical test. Reason: Electrotesting is the most straightforward and reliable way to determine pulp viability because it provides accurate information about pulp nerve activity.
5. D. Pacemakers. Causes: Pulp viability testing may interfere with pacemaker function and is therefore contraindicated in patients with pacemakers.
6. A. Use of anesthetic prior to testing. Causes: The use of anesthetics may affect the electroviability test results of the pulp, but it results in a false positive, not a false negative.
7. D. Pulp necrosis. Causes: Electro-pulp viability tests are commonly used to diagnose whether the pulp is alive, i.e., whether pulp necrosis is present.
8. B. Thermal temperature is above 60°C. Reason: Temperatures up to 60°C are not usually used for temperature tests, as this may cause tissue damage.
9. E. All of the above. Causes: All of these factors can cause a false positive on the electrododontic viability test.
10. E. Patient has used analgesics, anesthetics or alcoholic beverages beforehand, etc. Causes: The use of these substances may reduce the patient's perception, but they often result in false negatives rather than false positives.

**Chapter 29 Endodontics Internship Course**

1.2020U1A1058 The materials commonly used in non-traumatic repair therapy are ( )

A. Glass ions

B. Composite resins

C. New composite resins

D. Amalgam

E. Pit and fissure sealants

2.2015U2A2107 Yao, male, 2 years old and 7 months, parents found that the child's upper anterior teeth were "blackened" and asked for treatment, and the left and right upper I. proximal and median adjacent surface were examined for superficial caries. Treatment is generally used ( )

A. Medication

B. Filling therapy

C. Restorative treatment

D. Root canal treatment

E. Observation and follow-up

3.2008U2A1035 Clinical manifestations of periodontal lesions caused by apical periodontitis ( )

A. Pulp no viability or abnormal viability

B. Periodontal lesions are confined to one tooth

C. X-ray shows that the periapical destruction zone is flask-like

D. Often accompanied by dental lesions

E. All of the above

4.2008U2A1037 Which of the following pulp lesions may cause periodontal disease ( )

A. Acute serous pulpitis

B. Acute suppurative pulpitis

C. Chronic pulpitis

D. Acute periapical abscess

E. Residual myelitis

5. The pathological changes of 2018U2A1007 acute serous apical periodontitis are ( )

A. Minimal neutrophil infiltration

B. Alveolar bone hyperplasia at the apex

C. Squamous epithelial cord of hyperplasia within the lesion

D. Formation of a large amount of granulation tissue in the lesion

E. Apical cementum resorption

6.2017U3A1004 The total daily saliva of normal people is 1000~1500ml, of which the secretion of parotid gland and submandibular gland accounts for ( )

A. 50%

B. 60%

C. 70%

D. 80%

E. 90%

7. 2014U3A2074 Hypertensive patient, male 65 years old, maxillary right 67 stump, no history of heart disease, need to extract teeth, the nerve that should be anesthetized during extraction is ( )

A. Infraorbital nerve + posterior palatine nerve

B. Superior antalveolar nerve + superior postalveolar nerve + anterior palatine nerve

C. Superior posterior alveolar nerve + superior middle alveolar nerve + nasopalatine nerve

D. Superior posterior alveolar nerve + superior middle alveolar nerve + anterior palatine nerve

E. Superior postalveolar nerve + anterior palatine nerve

8. 2014U3AN143 female, 22 years old, requested extraction due to low ambush impaction of right lower wisdom tooth. In order to reduce bleeding and maintain a clear surgical field, epinephrine is often added to the anesthetic, and the nerve that should be anesthetized to extract the tooth is the inferior alveolar nerve + buccal nerve + lingual nerve on the same side.

A. Anesthesia of the inferior alveolar nerve

B. Anesthetized masticatory nerve

C. Buccal nerve anesthetized

D. Anesthetized the facial nerve

E. Oral hormones

9.2019U3A1003 The nerve anesthetized for maxillary first molars extraction is ( )

A. Nasopalatine nerve, anterior palatine nerve, posterior palatine nerve

B. Superior postalveolar nerve, superior alveolar middle nerve, anterior palatine nerve

C. Superior posterior alveolar nerve, superior middle alveolar nerve, nasopalatine nerve

D. Superior postalveolar nerve, superior alveolar middle nerve, superior antalveolar nerve

E. Superior alveolar middle nerve, nasopalatine nerve, anterior palatine nerve

10.2018U3A2111 Male, 2 years old. For cleft lip repair, an infraorbital nerve block anesthesia is added, and the needle insertion point is ( )

A. 1.5 cm below the midpoint of the infraorbital margin, the midpoint of the line from the alar of the nose to the tragus

B. 0.5 cm below the midpoint of the inferior orbital margin, from the tip of the nose to the midpoint of the tragus

C. 1.5 cm below the midpoint of the inferior orbital margin, the midpoint of the nasal alar to the lateral eyelid line

D. 0.5 cm below the midpoint of the inferior orbital margin, from the tip of the nose to the midpoint of the lateral eyelid line

E. 0.5 cm below the midpoint of the inferior orbital margin, the midpoint of the nasal alar to the lateral eyelid line

11.2003U3AN103 "Oral Interpretation" male, 45 years old. He complained of discomfort in his left mandibular posterior teeth for 6 months. Oral examination: 56 residual crown in the lower left, percussion (±), loose (-), endodontic treatment has been done, the gums are slightly red, can not be repaired, and are to be extracted. There were no obvious abnormalities in the other teeth in the mouth. X-rays showed that there was a shadow at the apex of the distal root in the lower left 6, which was close to the mandibular neural canal. The lower left 56 removed nerves that do not require anesthesia are ( )

A. Inferior alveolar nerve

B. Tongue nerve

C. Masseter nerve

D. Buccal nerve

E. All of the above nerves require anesthesia

12. 2013U3A2109 patient, a 24-year-old male, complained of hot and cold irritation pain in the right lower back tooth for 3 days at night. On examination, the lower right third molar is impacted in the near middle and low position, there is more food impaction between the lower right 78, the lower right 8 does not detect caries, and the distal blind pocket is slightly red and swollen, and it is painful to touch. Cold (-). Detection of caries in the distal neck of the lower right 7 is profound, deep, soft, and cold stimulation causes severe pain. Probable diagnosis of presenting tooth ( )

A. Hypo8 coronitis in the lower right

B. Acute apical inflammation in the lower right 7

C. Right lower 78 periodontitis

D. Acute pulpitis in the lower right 7

E. Near-mid-low impaction in the lower right 8

1. B。 Composite resin is currently a commonly used material in non-invasive reconstructive treatment because of its good aesthetics and adhesion.
2. B。 For superficial caries in children aged 2 years and 7 months, filling therapy is usually used to prevent further progression of caries.
3. E。 Periodontal lesions caused by apical periodontitis will be manifested as pulp viability or abnormal viability, the lesion is confined to a certain tooth, and X-ray shows that the periapical destruction area is flask-like, often accompanied by dental lesions, so E is chosen.
4. B。 Acute suppurative pulpitis may cause periodontal disease because the infection can spread to the periodontal tissue through the apical foramen.
5. A。 The pathological changes in acute serous apical periodontitis are predominantly infiltrated by a small number of neutrophils, which is an incipient inflammatory response.
6. C。 In a normal person, the parotid and submandibular glands account for about 70% of the total daily saliva.
7. D。 When the right maxillary 67 stump is extracted, the nerve that should be anesthetized is the superior postalveolar nerve + the middle superior alveolar nerve + the anterior palatine nerve.
8. D。 Insufficiency of the right eyelid after anesthetic injection may be due to accidental numbing of the facial nerve, which controls facial expression muscles.
9. B。 When extracting the maxillary first molar, the nerves that need to be anesthetized are the superior postalveolar nerve, the superior alveolar middle nerve, and the anterior palatine nerve.
10. A。 The needle insertion point for infraorbital nerve block anesthesia is 1.5 cm below the midpoint of the infraorbital margin, at the midpoint of the nasal alar to tragus connection.
11. E。 All of the listed nerves are anesthetized when the left lower 56 is extracted, as these nerves are involved in the sensation of the mandibular teeth.
12. D。 Based on the patient's symptoms and examination results, the diagnosis of the presenting tooth is acute pulpitis in the lower right 7, because severe pain caused by cold stimulation is a typical symptom of pulpitis.

**Periodontology**

**Chapter 2 Applied Anatomy and Physiology of Periodontal Tissue**

1.2008U2A1022 The epithelium of the inner wall of the gingival sulcus is ( )

A. Non-keratinized stratified squamous epithelium

B. Keratinized stratified squamous epithelium

C. Infully keratinized stratified epithelium

D. Laminated columnar epithelium

E. Laminated epithelium without spikes

2.2008U2A1025 The distance from the normal alveolar crest to the enamel cementum boundary is ( )

A. 1~2mm, no more than 2.5mm

B. 1~1.5mm, not more than 2mm

C. 1.5~2mm, not more than 3mm

D. 2~3mm, not more than 3mm

E. 0.5~2.5mm, not more than 3mm

3.2004U2A1057 Factors to keep the tooth position normal and undisplaced include ( )

A. Adequate periodontal support tissue

B. Dental force and labial-buccal-tongue muscle strength

C. Integrity of dentition

D. Normal tooth morphology and adjacency

E. All of the above

4.2005U2A1017 The tooth position that is least prone to periodontal destruction is ( )

A. Maxillary incisors and mandibular molars

B. Mandibular incisors and maxillary molars

C. Maxillary canines and mandibular premolars

D. Mandibular canines and maxillary premolars

E. Maxillary and mandibular premolars

5.2013U2A1008 How many mm is the distance from the alveolar crest to the enamel cementum boundary on X-ray that exceeds the alveolar bone resorption ( )

A. 1mm

B. 2mm

C. 3mm

D. 4mm

E. 5mm

6.2013U2A1011 How much gingival sulcus depth exceeds should be recorded ( )

A. 1mm

B. 2mm

C. 2.5mm

D. 3mm

E. 3.5mm

7. 2011U2A1007 The width of the periodontal ligament is generally ( )

A. 0．5～1mm

B. 0.15～0.38mm

C. 1．2～2mm

D. 0.5～0.1mm

E. 1～2mm

8. 2007U2A1001 Gull of the gum is the first site of periodontal disease because ( )

A. Loose intercellular connections in the epithelium

B. The epithelium is keratinized and non-self-cleaning

C. The epithelium is awholly keratinized and the epithelial thickness is thin

D. There is no connective tissue in the subepithelial lamina propria at the site

E. The area is prone to food impaction, resulting in bacterial retention and multiplication

9.2007U2A1002 Which of the following structures is the biological width composed of ( )

A. Connective tissue combining the width of the crown root of the epithelium + the crown square of the bony crest

B. Width of the crown-roots direction of the combined epithelium + depth of the gingival sulcus

C. Connective tissue combining epithelial width + gingival sulcus depth + bony crest crown square

D. Depth of probing + connective tissue of the crown of the bony crest

E. Probing depth minus gingival sulcus depth

10.2005U2A1019 The thickness of the periodontal ligament is different in different people and different teeth, generally ( )

A. 0.01～0.38mm

B. 0.15～0.38mm

C. 0.05～0.38mm

D. 0.10～0.38mm

E. 0.20～0.38mm

GPT-4

1. A。 Cause: The inner wall epithelium of the sulcus is usually a non-keratinized stratified stratified squamous epithelium.
2. A。 Reason: The distance from the normal alveolar crest to the enamel cementum boundary is usually 1~2mm, not more than 2.5mm.
3. E。 Causes: Factors that keep the tooth in position normal include adequate periodontal support tissue, dental synergy and laboclucial-tongue muscle strength, intact dentition, normal tooth morphology, and adjacency, so all of them are.
4. B。 Causes: The most susceptible to periodontal destruction are usually mandibular incisors and maxillary molars, as these positions are more evenly stressed and periodontal hygiene is easier to maintain.
5. B。 Cause: The distance from the alveolar crest to the enamel cementum boundary of more than 2 mm on X-ray can be regarded as alveolar bone resorption.
6. C。 Causes: It is generally accepted that sulcus depth greater than 2.5 mm should be recorded, as this may indicate the presence of periodontal disease.
7. B。 Reason: The width of the periodontal ligament is generally narrow, about 0.15~0.38mm.
8. B。 Causes: The gingival valley is the starting site of periodontal disease because the epithelium is keratinized and non-self-cleaning.
9. A。 Cause: The biological width consists of the coronal root width that binds the epithelium, and the connective tissue that connects the crown square of the bony crest.
10. B。 Reason: The thickness of the periodontal ligament is different in different people and different teeth, and the general range is 0.15~0.38mm.

**Chapter 4 Microbiology of Periodontal Disease**

1.2009U2A1051 Correlation between bacteria in plaque ( )

A. Symbiosis

B. Antagonism

C. Competition

D. Symbiosis, antagonism, and competitive coexistence

E. Varies from person to person

2.2009U2A1068 The following table shows the characteristics of subgingival plaque, pointing out the wrong one of the attached plaques and non-attached plaques ( )

A. Does not extend to the bound epithelium, extends to the bound epithelium

B. No contact with the sulcus epithelium, contact with the sulcus epithelium

C. Permeable epithelium and connective and may be connected to supragingival plaque

D. G+ bacteria are dominant, and Gˉ active bacteria are dominant

E. Associated with calculus formation root caries, related to periodontal tissue inflammation

3. 2009U2A1076 Supragingival plaque is correctly defined ( )

A. Unmineralized bacterial sediments, firmly adhered to tooth surfaces and restoration surfaces, composed of bacteria

B. Unmineralized bacterial sediment, firmly adhered to the tooth surface and the surface of the restoration, consisting of a sticky matrix and bacteria embedded in it

C. Partially mineralized bacterial sediment, firmly adhered to the tooth surface and the surface of the restoration, consisting of a sticky matrix and bacteria embedded in it

D. Mineralized bacterial sediments, firmly adhered to tooth surfaces and restorations, consisting of a sticky matrix and bacteria embedded in them

E. None of the above

4.2009U2A1078 Periodontal bacteria lead to the occurrence of periodontal destruction, and the mechanism is ( )

A. Direct invasion of bacteria into the periodontal tissues

B. The bacteria directly invade the periodontal tissues, causing an immune response

C. Bacteria multiply in the body

D. Inhibition and evasion of the host's defense function

E. All of the above

5. 2009U2A1080 The most important bacteria that cause gingivitis are ( )

A. Gram-positive bacilli

B. Gram-negative bacilli

C. Spirochetes

D. Chlamydia

E. Actinomycetes viscosus

6. 2016U2A1020 Microorganisms implicated in the occurrence of periodontal disease, the most well-documented are ( )

A. With Actinobacillus actinomyceter, Prevotella intermediates, and Fusobacterium nucleata

B. Porphyromonas gingivals, Prevotella intermediates, Trephelum donticula

C. With Actinobacterium, Porphyromonas gingivalis and Pseudobacteroides fersa

D. Pruzewala intermediates, Pseudobacteroides fersa, Treponebacterium dentensis

E. with Actinobacillus, Pseudobacteroides fersia, Fusobacterium nucleatus

7. 2016U2A1023 The bacterial components that are not related to periodontal destruction are ( )

A. Bacterial surface material

B. Gliase and hyaluronidase secreted by bacteria

C. Bacterial toxins

D. Bacterial metabolites

E. Bacterial size and morphology

8.2008U2A1023 Which of the following descriptions is incorrect about non-adherent subgingival plaque ( )

A. Under certain conditions, the amount of non-adherent subgingival plaque is greatly increased

B. Its composition is dominated by anaerobic bacteria

C. Closely related to the destruction of alveolar bone

D. Causes root caries

E. Loose structure, direct contact with the pouch epithelium and gingival sulcus

9.2013U2A1014 Which of the following suspected periodontal pathogens is associated with recurrence or further exacerbation of periodontitis after treatment ( )

A. Meprevotesa

B. Fersaibacter

C. Fusobacterium nucleatus

D. Porphyromonas gingivals

E. Actinobacillus actinomyceter

10.2011U2A1008 Which of the following bacterial surface substances is the most toxic to periodontal tissues ( )

A. Lipoteichoic acid

B. Outer membrane proteins

C. Endotoxin

D. Ciliary proteins

E. Membrane vesicles

11. 2010U2A1049 The main basis of the non-specific plaque theory ( )

A. Plaque extract can cause subcutaneous abscess in animals

B. If there is a lot of plaque and tartar, the gum inflammation is more severe

C. Removal of plaque and tartar can reduce inflammation

D. Plaque overall microbial co-pathogenicity

E. All of the above

12.2007U2A1003 Subgingival non-attached plaque is mainly composed of the following bacteria ( )

A. Gram-positive cocci, bacilli, and filamentous bacteria

B. Gram-negative anaerobes, actine bacteria

C. Gram-positive facultative and anaerobic bacteria

D. Gram-positive aerobic and facultative bacteria

E. Gram-positive aerobic and gram-negative anaerobes

13.2007U2A1004 Main points of the specific plaque theory ( )

A. Periodontal disease occurs as a result of mixed infection of the oral flora

B. Periodontal destruction is due to an increase in the number of bacteria and increased virulence

C. Periodontal destruction of different types and sites is associated with specific bacteria

D. Periodontal destruction is due to low defenses at the lesion site

E. All of the above

14.2002U2A1030 Which of the following is related to the formation of subgingival tartar ( )

A. Supragingival plaque

B. Attached subgingival plaque

C. Non-adherent subgingival plaque

D. White scale

E. Food crumbs

GPT-4

1. D。 Causes: There is a symbiotic, antagonistic and competitive relationship between bacteria in plaque.
2. E。 Causes: Plaque attachment is associated with the formation of root caries in tartar, while non-plaque is associated with inflammation of periodontal tissue.
3. B。 Cause: Supragingival plaque is an unmineralized bacterial deposit that adheres firmly to the tooth surface and the surface of the restoration, and consists of a sticky matrix and bacteria embedded in it.
4. E。 Causes: Periodontal bacteria lead to periodontal destruction, and the mechanisms include direct invasion of periodontal tissues, immune response, and inhibition and evasion of host defenses.
5. A。 Causes: The most important bacteria that cause gingivitis are gram-positive bacilli.
6. C。 Causes: Organisms involved in the development of periodontal disease, with the most abundant evidence being Aggregator actinobacterium, Porphyromonas gingivalis and Pseudobacterium fersa.
7. E。 Cause: The bacterial component that is not associated with periodontal destruction is the size and morphology of the bacteria.
8. D。 Causes: Non-attached subgingival plaque is not a major contributor to root caries.
9. D。 Causes: The presence of Porphyromonas gingivalis is associated with recurrence of periodontitis after treatment or continued exacerbation of the condition.
10. C。 Cause: Endotoxins are the most toxic to periodontal tissues among bacterial surface substances.
11. E。 Causes: The non-specific plaque theory is mainly based on the overall microbial co-pathogenesis of plaque.
12. B。 Cause: Subgingival non-attached plaque is mainly composed of gram-negative anaerobic bacteria and actine bacteria.
13. C。 Causes: The main idea of the specific plaque theory is that periodontal destruction of different types and sites is related to specific bacteria.
14. B。 Causes: Associated with the formation of subgingival tartar is attached subgingival plaque.

**Chapter 6 Immune inflammatory responses and promoters in periodontal disease hosts**

1.2008U2A1020 The main components of gingival crevicular fluid are derived from ( )

A. Blood

B. Serum

C. Plasma

D. Adjacent periodontal tissue

E. Bacteria

2.2004U2A1059 Effect of smoking on periodontal tissue, excl. ( )

A. Reduction of local oxygen tension

B. Inhibition of fibroblast growth

C. Inhibition of neutrophil function

D. Not conducive to oral hygiene maintenance

E. Inhibition of collagenase activity

3.2004U2A1063 Effect of gingival crevicular fluid ( )

A. Flush the sulcus with foreign material

B. Has antimicrobial effect

C. Rich in plasma proteins

D. Provide the inorganic components of tartar mineralization

E. All of the above

4. 2010U2A1040 Factors related to the severity of periodontal destruction in diabetic patients are ( )

A. Types of diabetes

B. Degree of metabolic control

C. The length of the duration of diabetes

D. Presence or absence of systemic complications

E. All of the above

5.2015U2A1007 Saliva function ( )

A. Mechanical cleaning

B. Lubrication and protection

C. Buffering

D. Antimicrobial

E. All of the above

6. 2010U2A1042 The possible relationship between diabetes mellitus and periodontal disease is ( )

A. Diabetes mellitus is one of the risk factors for periodontal disease

B. Periodontal disease does not affect diabetes

C. There is a two-way relationship between diabetes and periodontal disease

D. A+B

E. A+C

7.2005U2A1036 Type I diabetic periodontitis is more common in ( )

A. Maxillary incisors

B. Mandibular incisors

C. Upper and mandibular incisors and bicuspids

D. Upper and mandibular incisors and first molars

E. Upper and mandibular incisors and second molars

8.2015U2A1021 Systemic contributing factors for periodontitis include ( )

A. Gastric ulcers

B. Dental deformities

C. Mental stress

D. Hepatitis B

E. Pyelonephritis

9.2017U2A1021 What is incorrect in the narrative about gingival crevicular fluid is ( )

A. Very small amounts of gingival crevicular fluid in healthy gums

B. Significant increase in gingival crevicular fluid during inflammation

C. The liquid component is mainly derived from serum

D. Immunoglobulins in the gingival crevicular fluid have the effect of anti-specific pathogenic bacteria

E. There are no defense cells such as leukocytes in the gingival sulcus fluid

1. A。 Cause: The main component of gingival crevicular fluid comes from the blood.
2. E。 Causes: The effects of smoking on periodontal tissues include all options except inhibition of collagenase activity.
3. E。 Causes: The action of gingival sulcus fluid includes flushing of foreign substances in the sulcus, antimicrobial effect, abundant plasma proteins, and inorganic components that provide tartar mineralization.
4. E。 Causes: Factors related to the severity of periodontal destruction in diabetic patients include the type of diabetes, the degree of metabolic control, the length of the duration of diabetes, and the presence or absence of systemic complications.
5. E。 Causes: Saliva functions include mechanical cleaning, lubrication and protection, cushioning, anti-microbial, etc.
6. E。 Causes: There is a two-way relationship between diabetes and periodontal disease, and diabetes is one of the risk factors for periodontal disease.
7. D。 Causes: Periodontitis in type I diabetes mellitus is more common in the upper and lower incisors and first molars.
8. C。 Causes: Systemic contributing factors to periodontitis include mental stress.
9. E。 Cause: There are defense cells such as leukocytes in the gingival crevicular fluid, so option E is incorrectly described.

**Chapter 7 Main symptoms and clinical pathology of periodontal disease**

1.2009U2A1052 Which is wrong about the relationship between clinical manifestations and histopathology of periodontal pockets ( )

A. The gums are dark red because of the destruction of gingival fibers in the connective tissue and collagen around the blood vessels

B. The gums are dark red due to local circulatory blockage

C. The gingival surface is shiny, and the stippling disappears due to atrophy of the gingival surface and edema of the tissue

D. The cause of bleeding after probing is degeneration, thinning, and ulceration of the epithelium in the periodontal pocket

E. Sometimes there is pus leaking in the pouch due to purulent inflammation in the pouch

2.2016U2A1002 The most common presenting symptom of gingivitis is ( )

A. Pain

B. Itching

C. Drying

D. Bleeding

E. Abscess

3.2016U2A1008 Complex bag refers to ( )

A. Periodontal pockets involving 1 tooth surface

B. Periodontal pockets involving 2 tooth surfaces

C. Periodontal pockets involving 3 tooth surfaces

D. Periodontal pockets involving 4 tooth faces

E. Spiral pouch, originating from one tooth face, twisting and rotating in more than one tooth face or root bifurcation area

4.2016U2A1011 The most common alveolar bone resorption mode in periodontitis is ( )

A. Horizontal alveolar bone resorption

B. Vertical alveolar bone resorption

C. Pit alveolar bone resorption

D. Anti-wavy alveolar bone resorption

E. Buttress bone formation

5.2008U2A1004 Generally speaking, the correct process of periodontal pocket formation is ( )

A. Inflammation of gingival connective tissue → destruction of collagen fibers → proliferation of epithelial roots

B. Collagen fiber disruption → gingival connective tissue inflammation → combined with epithelial root proliferation

C. Gingival connective tissue inflammation → combined with epithelial root proliferation → destruction of collagen fibers

D. Collagen fiber destruction → combined with epithelial root proliferation → gingival connective tissue inflammation

E. Combined epithelial root proliferation→ gingival connective tissue inflammation → collagen fiber destruction

6.2008U2A1008 Loose teeth have little to do with which of the following factors ( )

A. Alveolar bone resorption

B. Trauma

C. Acute inflammation of the periodontal ligament

D. Changes in female hormone levels

E. Severe inflammation of the gums

7.2008U2A1014 The probing depth obtained during clinical probing is average ( )

A. Can be greater or less than the histological depth of the gingival sulcus

B. Less than histological sulcus depth

C. Equal to histological gingival sulcus depth

D. Greater than histological sulcus depth

E. Varies depending on the degree of gum inflammation

8.2008U2A1018 When the resultant force exceeds the supporting potential of the periodontal tissue, the final pathological change is ( )

A. Tissue degeneration

B. Tissue damage

C. Tissue repair

D. Organizational restructuring and reconstruction

E. Tooth loss

9.2008U2A1021 The mechanism of periodontal pocket formation is ( )

A. Associated epithelial root proliferation caused by gingival inflammation

B. Collagen fibers in connective tissue are massively destroyed

C. Bacteria and their products cause gum inflammation

D. A large number of neutrophils invade the bound epithelium and disrupt its junctions

E. All of the above

10.2008U2A1024 Which item is wrong about the description of the subbony pocket ( )

A. It is often accompanied by occlusal trauma

B. The bottom of the pouch is located at the root of the bony crest

C. Alveolar bone vertical resorption

D. Transseptal fibers attach from the cementum at the bottom of the pouch over the crest to the cementum of the adjacent teeth

E. Gingival hyperplasia and hypertrophy

11. 2008U2A1028 One of the most important clinical manifestations of periodontal disease is ( )

A. Tooth displacement

B. Periodontal discharge

C. Bad breath

D. Periodontal pocket formation

E. Swollen, bleeding, sore gums

12.2008U2A1029 Which of the following is wrong about the concept of periodontal tissue disease ( )

A. Periodontal tissue disease is a chronic inflammatory disease of the gums, periodontal ligament, cementum, and alveolar bone

B. Periodontitis, once diseased, is always a continuous process of destruction

C. Disease progression often alternates between active and quiescent phases

D. Different individuals have different sensitivities to periodontal pathogenic factors

E. Plaque, tartar is closely related to periodontal tissue disease

13.2008U2A1030 Site-specific manifestations of periodontal disease ( )

A. Destruction occurs in all parts of the oral cavity at the same time

B. Destruction occurs only at the site of a pre-existing disease

C. Destruction occurs only in the new area, and the pre-existing disease heals

D. Occurrence of destruction of new sites and exacerbation of destruction of pre-existing disease sites

E. All of the above

14.2004U2A1056 Periodontal pocket probing bleeding showed ( )

A. Inflammation in the periodontal pocket

B. Residual tartar on the root surface

C. Periodontal flap surgery is required

D. Gingivectomy is required

E. Periodontal pouch administration is required

15.2004U2A1060 Which is incorrect about the description of the active destruction period of periodontal inflammation ( )

A. Massive increase in subgingival non-attached plaque

B. Symptoms of retrograde pulpitis are present

C. Loss of attachment

D. Alveolar bone resorption

E. Increasing proportion of anaerobic bacteria and spirochetes in plaque

16.2004U2A1066 Subosseous pouch is classified according to the following ( )

A. Number of tooth surfaces involved

B. Degree of bone destruction

C. Number of remaining bone walls

D. Relationship between the floor of the periodontal pocket and the crest of the alveolar crest

E. Depth of periodontal pockets

17.2004U2A1068 The difference between a false periodontal pocket and a true periodontal pocket is ( )

A. Depth of probing

B. Number of tooth surfaces involved

C. Position of the bottom of the periodontal pocket

D. Whether there is a subbony pocket

E. Gingival morphology and degree of bleeding on probing

18.2010U2A1041 The inferior pouch with the worst treatment effect is ( )

A. A wall of bone bags

B. Two-wall bone bag

C. Three-walled bone bag

D. Four-walled bone pouch

E. Mixed stud bag

19. 2015U2A1006 Alveolar bone resorption that is difficult to show on X-ray is ( )

A. Horizontal alveolar bone resorption

B. Vertical alveolar bone resorption

C. Pit alveolar bone resorption

D. Anti-wavy alveolar bone resorption

E. Buttress bone formation

20.2012U2A1008 Bleeding symptoms on gingival probing first appeared in ( )

A. Normal gums

B. Primary gingivitis lesions

C. Early gingivitis lesions

D. Establish gingivitis lesions

E. Advanced periodontitis lesions

21.2012U2A1010 To affect the junction and nutrition of epithelial cells, infiltrated leukocytes need to reach a bound epithelial volume of ( )

A. 0.2

B. 0.3

C. 0.4

D. 0.5

E. 0.6

1. B。 The reason is that dark red gums are usually associated with vascular congestion rather than local circulatory obstruction.
2. D。 Bleeding is the most common symptom of gingivitis because inflammation causes the gums to become brittle and bleed easily.
3. E。 Complex pockets are spiral in shape and can involve multiple tooth surfaces or root bifurcation areas.
4. A。 The most common form of alveolar bone resorption in periodontitis is horizontal.
5. B。 Destruction of collagen fibers occurs first, followed by inflammation of the connective tissue of the gums, and finally proliferation in combination with epithelial roots.
6. D。 Changes in female hormone levels are usually less associated with loose teeth.
7. D。 The depth of clinical probing is usually greater than the depth of the histological sulcus because of inflammation leading to thickening of gingival tissue.
8. B。 When the resultant force exceeds the periodontal tissue support potential, the final pathological change is tissue damage.
9. E。 The mechanism of periodontal pocket formation includes a variety of factors, such as inflammation, destruction of collagen fibers, and bacterial invasion.
10. E。 The mistake in the description of the subosseous pocket is gingival hyperplasia and hypertrophy, because the subosseous pocket is associated with alveolar bone resorption rather than gingival hyperplasia.
11. D。 Periodontal pocket formation is one of the most important clinical manifestations of periodontal disease.
12. B。 Periodontitis may present with an intermittent process of destruction rather than always a continuous process.
13. D。 Site-specific manifestations are the occurrence of destruction of new sites and the aggravation of destruction of pre-existing disease sites.
14. A。 Bleeding on probing indicates inflammation within the periodontal pocket.
15. B。 The presence of retrograde pulpitis symptoms is not characteristic of the active destruction phase of periodontitis.
16. D。 Subosseous pockets are classified according to the relationship between the floor of the periodontal pocket and the alveolar crest.
17. D。 The main difference between a false periodontal pocket and a true periodontal pocket is whether or not a subbony pocket is involved.
18. D。 Four-wall bone pockets are the least effective because they have the most loss of supporting tissue.
19. B。 Vertical alveolar bone resorption is difficult to show on x-rays.
20. C。 Bleeding on probing of the gingiva first appears in the early stage of gingivitis lesions.
21. D。 Leukocyte infiltration needs to reach 0.5 of the bound epithelial volume to affect epithelial cell junction and nutrition.

**Chapter 8 Examination and Diagnosis of Periodontal Disease**

1.2009U2A1042 In order to obtain a clinically accurate periodontal pocket depth or attachment level, the key points that should be mastered are ( )

A. Use standardized probes

B. Master the correct probing power

C. Probing direction parallel to the long axis of the tooth

D. Sequential walk-in probing process and stable fulcrum

E. All of the above

2.2009U2A1048 Occlusion examination, wax bite tablets are often used to check ( )

A. Median occlusion

B. Anterior occlusion

C. Lateral occlusion

D. Crossbite

E. All of the above

3.2016U2A1007 Special instruments for clinical examination of periodontal tissue( )

A. Oral scope, probe, forceps

B. Periodontal probe, electrovinometer, thin wax sheet

C. Periodontal probe, teether stick, dental floss, stain

D. Periodontal probe, dental floss, occlusal paper, thin wax sheet

E. Periodontal probes, bite papers, thin wax films, X-rays

4.2016U2A1012 II. Degree loose teeth refer to ( )

A. Vertical loosening

B. Slightly greater than physiological mobility within 1 mm

C. Shake from the normal position to the buccal tongue or mesiodistal direction with a movement greater than 2 mm

D. Shake from the normal position to the buccal tongue or mesiodistal direction, with a movement of 1~2mm

E. Shake from the normal position to the buccal tongue or mesiodistal direction with a movement greater than 3 mm

5.2016U2A1021 Inspection method for accurately judging the presence or absence of periodontal pockets ( )

A. Inspect for swelling of the gums

B. Inspection and inspection of the gum color

C. Occlusion examination

D. Periodontal probe examination

E. X-rays

6.2013U2A1003 Which item is incorrect about the present periodontal history ( )

A. The timing, course and possible triggers of the onset

B. How the disease was treated

C. Bleeding gums and periodontal abscess

D. Whether the parents have periodontitis

E. Oral hygiene

7.2008U2A1009 Methods used to check early contact points do not include ( )

A. Inspection

B. Examination

C. Occlusion paper method

D. Wax bite tablet method

E. Floss

8. 2008U2A1013 II. degree bone resorption describes the bone destruction ( )

A. Alveolar bone resorption is within 1/3 of the root neck

B. Alveolar bone resorption exceeds 1/3 of the root length, but is within 2/3 of the root length or up to 1/2 of the root length

C. Alveolar bone resorption accounts for more than 2/3 of the root length

D. Alveolar bone resorption accounts for more than 1/2 and less than 3/4 of the root length

E. Alveolar bone resorption within 1/2 of the root neck

9.II. Degree of tooth loosening refers to ( )

A. The tooth loosening amplitude is less than 1mm

B. The tooth loosening amplitude is within 0.5~lmm

C. The tooth loosening amplitude is within 2~3mm

D. The tooth loosening amplitude is within 1~3mm

E. The tooth loosening amplitude is within 1~2mm

10.2008U2A1017 Which of the following describes occlusion interference ( )

A. When the anterior extension is occluded, there is extensive contact between the anterior teeth

B. Non-working cusp contact during lateral occlusion

C. When the anterior extension teeth are occlusal, the anterior teeth are in single-point contact

D. When the lateral occlusion, the working side cusps are widely contacted

E. Deep overbite

11. 2008U2A1019 III. degree bone resorption describes the bone destruction as ( )

A. Alveolar bone resorption is within 1/3 of the root neck

B. Alveolar bone resorption exceeds 1/3 of the root length, but is within 2/3 of the root length or up to 1/2 of the root length

C. Alveolar bone resorption accounts for more than 3/4 of the root length

D. Alveolar bone resorption accounts for more than 1/2 and less than 3/4 of the root length

E. Alveolar bone resorption greater than 2/3 of the root length

12.2008U2A1027 The best non-invasive method for judging alveolar bone dynamics is ( )

A. Periodontal probing with pressure-sensitive probes

B. Dental mobility test

C. Dental force force test

D. X-ray digital subtraction technology

E. Gingival crevicular fluid examination

13.2008U2A1031 The force used in periodontal pocket probing is ( )

A. 10～20g

B. 20～25g

C. 25～30g

D. 30～35g

E. 35～40g

14.2013U2A1004 Measure the adhesion level, the appropriate distance is ( )

A. Gingival margin-enamel cementum boundary

B. Tooth neck-pouch bottom

C. Gingival margin - bottom of the bag

D. Enamel bony boundary-pouch bottom

E. Tooth neck-enamel cementum boundary

15.2013U2A1005 Periodontitis bone resorption on X-ray as alveolar bone ( )

A. Height reduction

B. The morphology remains unchanged

C. Increased density

D. Thickening of bony plates

E. Narrowing of the periodontal ligament

16.2015U2A2071 Female, 29 years old, healthy. Complaints: Bleeding gums in the past 3 months and bleeding gums when chewing hard foods. The following tests should be done, with the exception of ( )

A. Oral hygiene status

B. Determination of tooth resultant force

C. Inflammation of the gums

D. Loss of attachment

E. Depth of probing

17.2004U2A1055 Measurement of periodontal pocket depth, to detect ( )

A. Six sites on the buccal lingual side: mesial, central, and distal

B. Near-median and distal loci on the buccal and lingual side

C. Four sites on the buccal side: mesial, central, distal, and lingual center

D. Four loci on the lingual side: mesial, central, distal, and buccal center

E. Two central sites on the buccal lingual side

18.2013U2A1015 Which method cannot be used for the examination of occlusal trauma ( )

A. Examination

B. Inspection

C. Wax bite tablet method

D. Articulation paper test

E. Periodontal probing

19.2002U2A1035 In addition to measuring the depth of the bag, periodontal probing should also be explored ( )

A. Whether the gums are bleeding

B. Amount and distribution of subgingival calculus

C. Whether the root fork is involved

D. Location of the gingival margin

E. All of the above

20.2002U2A1036 Factors that cause loose teeth in periodontitis do not include ( )

A. Alveolar bone resorption

B. Occlusal trauma

C. Acute inflammation of the periodontal ligament

D. Periodontal pocket formation

E. A+B+C+D

21.2012U2A1024 The following should be checked during occlusion examination, except for ( )

A. Whether the dentition is intact

B. Whether there is crowding, dislocation, overbite, and overlay

C. Whether the overbite and coverage relationship are normal

D. Type of occlusion

E. Night grinding habits

22.2012U2A1025 occlusal trauma can be manifested as ( )

A. Horizontal resorption of alveolar bone

B. Asymmetric gingival recession and clefts

C. Formation of pseudoperiodontal pockets

D. Severe inflammation of the gums

E. Redness of the gums during occlusal movements

1. E。 Accurate measurement of pocketage depth and attachment level requires a combination of standardized probes, correct probe strength, proper probe orientation, and orderly and stable probe technology.
2. E。 Wax mounts can be used to check for a variety of occlusal relationships, including median, anterior, lateral, and crossbite.
3. C。 Clinical examination of periodontal tissues requires special instruments such as periodontal probes, teether sticks, dental floss, and dyes to fully assess periodontal condition.
4. D。 II. Loose teeth refer to the shaking of the teeth in the normal position to the buccal tongue or mesiodistal direction, and its movement is between 1~2mm.
5. D。 The presence of periodontal pockets is usually accurately determined by periodontal probe examination, which is confirmed by measuring the depth of the probe into the gums.
6. D。 Regarding the history of present periodontal disease, whether a parent has periodontitis is usually outside the scope of the standard inquiry because it focuses more on genetic factors than on the individual's periodontal condition.
7. E。 Early contact points are usually examined without flossing, and inspection, palpation, occlusion, and wax bite are commonly used.
8. B。 II. Bone resorption refers to alveolar bone resorption exceeding 1/3 of the root length, but within 2/3 of the root length or reaching 1/2 of the root length.
9. E。 II. Tooth loosening refers to the tooth loosening amplitude within 1~2mm.
10. B。 In lateral occlusion, contact of the non-working cusp is considered occlusal interference.
11. C。 Third-degree bone resorption describes alveolar bone resorption accounting for more than 3/4 of the root length.
12. D。 The best non-invasive method for determining alveolar bone dynamics is digital subtraction on X-rays, as it provides a detailed image of the bone structure.
13. C。 The force usually used for periodontal pocket probing is 25~30g.
14. C。 Measure the level of attachment at the appropriate distance from the gingival margin to the bottom of the pouch.
15. A。 Periodontitis usually appears on x-rays as a decrease in alveolar bone height.
16. B。 Dental resultant force measurement is not a routine test for bleeding gum symptoms.
17. A。 Periodontal pocket depth should be measured at six sites on the buccal lingual side: mesial, central, and distal.
18. E。 Periodontal probing cannot be used to examine occlusal trauma because it is primarily used to evaluate periodontal disease.
19. E。 In addition to measuring the depth of the pocket, periodontal probing should also be used to check for bleeding gingiva, the amount and distribution of subgingival calculus, the involvement of root bifurcations, and the location of the gingival margin.
20. E。 Factors for tooth loosening include alveolar bone resorption, occlusal trauma, and acute inflammation of the periodontal ligament and should not include periodontal pocket formation.
21. E。 Night grinding is not something that should be checked during an occlusion test.
22. A。 Occlusal trauma may manifest as alveolar bone level resorption.

**Chapter 9 Gum Disease**

1.2009U2A1044 Gingival tumor during pregnancy that interferes with eating, if surgery is necessary, optional ( )

A. Surgical excision at 4~6 months of pregnancy

B. Surgical excision at 3~8 months of pregnancy

C. Only plaque control is done, not excision

D. Resolves spontaneously as the gestation months increase

E. All of the above

2.2009U2A1045 Which is wrong to describe the lesions of mild chronic gingivitis ( )

A. Involvement of free gingiva

B. Involvement of the gingival papillae

C. Involvement of attached gingiva

D. Changes in gum color

E. Stippling can disappear

3.2009U2A1046 The most common gum disease is ( )

A. Multiple gingival abscesses

B. Single gingival abscess

C. Gingival papillitis

D. Chronic gingivitis

E. Gingival hyperplasia

4.2009U2A1050 In acute gingival papillitis, the clinical examination shows that the affected tooth has mild percussion pain ( )

A. Periodontal ligament below the gingival papilla is inflamed and edematous

B. Inflammation around the apex

C. Have a periodontal abscess

D. Periodontal trauma

E. Have acute pulpitis

5. The clinical manifestations of 2009U2A1074 proliferative gingivitis are ( )

A. Deep overbite of anterior teeth

B. Hypertrophy of the gingival margin, bulbous hyperplasia of the gingival papillary, and bleeding on probing

C. Periodontal pocket formation

D. Significant halitosis and pain

E. All of the above

6.2016U2A1006 Clinical manifestations of gingivitis ()

A. Significant pain

B. Spontaneous bleeding

C. Periodontal pocket formation

D. Crowded dental arrangement

E. There are often tartar and soft plaque deposits on the neck of teeth

7.2009U2A2108 male, 25 years old, the upper and lower anterior gingival papillae in the oral cavity disappeared and sunken, showing an anti-wavy shape, the buccal and lingual sides of the intergingival papillae were separated, which could be turned over from the tooth surface, and there was tartar and tartar below, and there was no gingival necrosis. The possible causes are ( )

A. Herpetic gingivostomatitis

B. Neutrophil deficiency causes gingival necrosis

C. Chronic gingivitis

D. Chronic necrotizing gingivitis

E. Gingival papillitis

8.2016U2A1022 The most common areas of gingivitis in adolescence are ( )

A. Maxillary molars buccal gingival margin

B. Palatarian gingival margin of maxillary molars

C. Anterior teeth, labial gingival margin, and gingival papillae

D. Maxillary bicuspid buccal gingival margin

E. Mandibular bicuspid interdental papilla

9.2008U2A1005 What are the main causative factors of gingivitis in adolescence ( )

A. Poor restorations

B. Food impaction

C. Tartar

D. Plaque

E. Occlusal trauma

10.2008U2A1011 The suspected pathogen of gingivitis in pregnancy is ( )

A. Actinomycetes viscosus

B. Prevotella intermediates

C. Endomycetes

D. Microdigestive streptococcus

E. Gingival carbon dioxide fibrophage

11.2008U2A1012 Among the plaque in women with gingivitis during pregnancy, the bacteria with the highest detection rate were ( )

A. intermediates

B. Porphyrimonas gingivals

C. Nuclusionella

D. Pseudobacterium fersa

E. Ekenbacterium

12.2013U2A2094 male, 21 years old, short upper lip, clinical examination: hypertrophy of the gingival margin and gingival papilla on the labial side of the upper anterior teeth, covering 1/3 of the crown, and the texture is tough. The most likely diagnosis is ( )

A. Proliferative gingivitis

B. Marginal gingivitis

C. Acute necrotizing gingivitis

D. Drug-induced gingival hyperplasia

E. Gingival fibromatosis

13.2010U2A1034 Gingival tumors during pregnancy are more common in ( )

A. Maxillary bicuspid buccal papilla

B. Mandibular bicuspid buccal alcrual papilla

C. Maxillary molars, buccal gingival papillae

D. Lower anterior labial gingival papillae

E. Mandibular molars, buccal gingival papillae

14.2000U2AN127 male, 27 years old, with sore gums, automatic bleeding for 3 days. Examination: putrefactive halitosis, loss and flattening of the tips of the gingival papillae of multiple teeth, and eroded necrosis of the gingival margins of the lower incisors covered with a grayish-white membrane. The most likely diagnosis of the disease is acute necrotizing gingivitis, and the most helpful ancillary tests are ( )

A. Smear Gram stain

B. Check for white blood cells

C. Photograph curved tomographs

D. Occlusal examination

E. Electromyography

15.2000U2AN129 male, 27 years old, with sore gums and spontaneous bleeding for 3 days. Examination: putrefactive halitosis, loss and flattening of the tips of the gingival papillae of multiple teeth, and eroded necrosis of the gingival margins of the lower incisors covered with a grayish-white membrane. The most likely diagnosis of the disease is acute necrotizing gingivitis, and the main causative organism that causes the disease is ( )

A. Actinobacillus actinobacillus

B. Porphyromonas gingivals

C. Staphylococcus

D. Clostridium and spirochetes

E. Actinomycetes

16.2000U2AN130 male, 27 years old, with sore gums and automatic bleeding for 3 days. Examination: putrefactive halitosis, loss and flattening of the tips of the gingival papillae of multiple teeth, and eroded necrosis of the gingival margins of the lower incisors covered with a grayish-white membrane. The most likely diagnosis of the disease is acute necrotizing gingivitis, and if the diagnosis is clear, and it is considered that systemic drugs should be used in treatment, the best choice of drug is ( )

A. Penicillin

B. Amoxicillin

C. Anti-inflammatory

D. Metronidazole

E. Synergistic bisulfonate tablets

17.2013U2A2095 female, 28 years old, complained: a tumor on the lingual side of the lower anterior teeth grew for 3 months, and slowly enlarged, affecting feeding. If the patient is diagnosed with a gestational tumor and needs to be resected, the following factors should be noted, except ( )

A. Inflammation of the gums

B. Timing of pregnancy

C. Whether or not to take medication during pregnancy

D. Oral hygiene

E. Selection of surgical method (laser, etc.)

18.2010U2A1035 For gestational gingival tumors that require surgical resection, the timing of surgery should be selected in ( )

A. Within 1 month of pregnancy

B. Pregnancy period of 2~3 months

C. Pregnancy period 4~6 months

D. Pregnancy period of 6~8 months

E. 9 months of gestation

19.2004U2A1050 Oral manifestations of leukemia patients do not include ( )

A. The gums are pale or dark red and inelastic

B. The gums have a tendency to bleed and are not easy to stop bleeding

C. Gingival margin is often necrosis

D. Loose and displaced teeth are common

E. Swollen gums that spread to all teeth

20.2013U2AN140 female, 28 years old, in the past 4 months, the gums of the whole mouth have gradually enlarged, the gums are easy to bleed when brushing teeth, and there is a history of occasional automatic bleeding of the gums. If the patient is diagnosed with gevicitis during pregnancy at 6 months of pregnancy, the clinical manifestations are most likely to be ( )

A. Sore, foul-smelling gums

B. Loose teeth arrangement

C. The gums are fibrous and enlarged

D. The gums are bright red and shiny

E. Gingival necrosis

21.2013U2AN141 female, 28 years old, has gradually enlarged gums in the past 4 months, gums are easy to bleed when brushing teeth, and occasionally there is a history of automatic bleeding of gums. If the patient is not pregnant, the oral manifestations of leukemia are suspected, and the diagnosis is confirmed by ( )

A. Biopsy

B. Exfoliated cell smear

C. Leukocyte phagocytosis

D. Leukocyte chemotaxis

E. Blood check

22.2013U2AN142 female, 28 years old, has gradually swollen gums in the past 4 months, gums are easy to bleed when brushing teeth, and occasionally have a history of automatic gingival bleeding. If leukemia is diagnosed, it is also manifested in the oral cavity ( )

A. The gums are pale, fluffy and fragile

B. The gums are bright red, shiny and soft

C. Fluctuating gums

D. Pseudomembrane on the surface of the gums

E. Herpes on the surface of the gums

23.2008U2A1032 The principles of treatment of gingival lesions in leukemia are ( )

A. Remove plaque and tartar immediately and thoroughly

B. Conservative treatment, hemostasis, and anti-infection

C. Removal of hypertrophied gums

D. Root surface leveling

E. Periodontal stoppers are used to prevent bleeding gums

24.2015U2A1001 The anaerobic microorganisms with the largest increase in the number of lesions in acute necrotizing gingivitis are ( )

A. Actinomycetes viscosus with spirochetes

B. With Actinobacillus

C. Fusobacterium and spirochetes

D. Clostridium and Porphyromonas gingivals

E. Porphyrimonospora gingivals

25.2015U2AN135 Female, 27 years old. Complained of swollen gums, bleeding and significant pain for 3 days. Oral examination: full-mouth gingiva swelling affecting the interdental papilla, marginal gingiva and attached gingiva, pale in color, necrotic ulcer at the gingival margin, and local bleeding. Hemorrhage spots may be seen on the oral mucosa, and the ancillary tests that should be done to further confirm the diagnosis are ( )

A. Probing the periodontal pocket

B. Percussion

C. X-rays

D. Perform routine blood tests in a timely manner

E. Perform bacteriological examinations

26.2015U2A1003 The most sensitive antibacterial drugs for the treatment of acute necrotizing ulcerative gingivitis are ( )

A. Tetracycline

B. Chlortetracycline

C. Sulfonamides

D. Metronidazole

E. Penicillin

27.2015U2A2072 Female, 29 years old, complaining: gums bleed easily after brushing teeth in the past 3 months, and gums bleeding when chewing hard food. If the patient is diagnosed with gingivitis of pregnancy, the most likely finding on clinical examination is ( )

A. Gingival papillae flattening

B. Sore, bleeding, foul-smelling gums

C. Bleeding gums and loose teeth

D. Hyperplasia of the gingiva covering 2/3 of the tooth surface

E. The gingival margin and papillae are bright red, soft and shiny

28.2004U2A1051 Swollen gums in leukemia are due to ( )

A. Gingival fibrous connective tissue hyperplasia

B. Gingival epithelial hyperplasia

C. Fibroblast proliferation

D. Massive lymphocyte infiltration

E. Massive infiltration and aggregation of immature, non-functioning leukocytes

29.2004U2A1052 The main pathological manifestations of gingival lesions in leukemia are ( )

A. The gums are filled with a large number of naïve white blood cells

B. There is a large number of capillary hyperplasia in the gums

C. There is a large amount of collagen fiber growth in the gums

D. Gingival epithelial hyperplasia

E. All of the above

30.2015U2AN136 Female, 27 years old. Complained of swollen gums, bleeding and significant pain for 3 days. Oral examination: full-mouth gingiva swelling affecting the interdental papilla, marginal gingiva and attached gingiva, pale in color, necrotic ulcer at the gingival margin, and local bleeding. Hemorrhagic spots may be seen on the oral mucosa. Routine blood count: abnormally increased white blood cell count and immature white blood cells. The initial diagnosis was: gingival manifestations of acute leukemia. Which of the following processing is wrong ( )

A. Cooperate with the physician in the treatment in a timely manner

B. Oral treatment should be conservative

C. Surgery or biopsy histopathology if needed

D. Bleeding can be stopped with local compression or medications

Rinse topically with 3% hydrogen peroxide solution gently

31.2015U2A2073 Female, 27 years old, examination: full mouth gingival hyperplasia covering 1/3~1/2 of the crown. Diagnosis of drug-induced gingival hyperplasia, which one is incorrect about the cause of gingival hyperplasia ( )

A. Increased division activity of fibroblasts

B. Increased secretion of inactive collagen-lytic enzymes

C. Not related to pre-existing gingival inflammation

D. Synthesis is greater than degradation

E. Increased ability to synthesize collagen

32.2008U2A1033 Clinical manifestations of drug-induced gingival hyperplasia ( )

A. Affects all oral teeth, and the upper and lower anterior teeth are heavier

B. The growth can range from marginal gingiva to attached gingiva

C. The gum texture is tough and elastic

D. The gingiva are spherical and nodal hyperplasia

E. All of the above

33.2004U2A1053 What is the obvious relationship between the degree of drug-induced gingival hyperplasia ( )

A. Oral hygiene status

B. Duration of medication

C. Dosage

D. Age

E. Gender

34.2010U2A1036 Drugs that can cause drug-induced gingival hyperplasia are ( )

A. Phenytoin

B. Phenytoin + nifedipine

C. Phenytoin + cyclosporine

D. Nifedipine + cyclosporine

E. Phenytoin + nifedipine + cyclosporine

35.2015U2A1004 Drugs that can cause gingival hyperplasia are ( )

A. Cyclosporine

B. Penicillin

C. Metronidazole

D. Tetracycline

E. Ibuprofen

36.2012U2A2107 female, 55 years old, with a history of hypertension for many years. Clinical: congestion and edema of the gingival margin and papilla, and the whole mouth gingival hyperplasia covers 1/3~1/2 of the crown.

A. Dalontinoids

B. Cyclosporine

C. Nifepyridine

D. Eliminate heartache

E. Reserpine

37.2005U2A1030 The causes of gingivitis in pregnancy are ( )

A. Endocrine changes

B. Occlusal trauma

C. Pregnancy itself

D. Malnutrition

E. All of the above

38.2010U2A1039 Which of the following is NOT a direct cause of gingivitis during pregnancy ( )

A. Plaque

B. Tartar

C. Food impaction

D. Poor restorations

E. Pregnancy

39.2015U2A1008 The drugs that can cause gingival hyperplasia are ( )

A. Nifepyridine

B. Metronidazole

C. Spiramycin

D. Dimethylaminetetracycline

E. Penicillin

40.2013U2A2096 Female, 55 years old, with a history of hypertension for many years, diagnosed with drug-induced gingival hyperplasia. Which of the patient's treatment options is incorrect ( )

A. Periodontal basic treatment

B. Systemic antibiotics

C. Oral health education

D. It is better to switch to another class of blood pressure medication

E. Periodontal surgery may be performed if necessary

41.2013U2A1016 Gingival fibromatosis is characterized by ( )

A. Soft gums

B. Never occurs in baby teeth

C. Gingival hyperplasia covers 2/3 of the crown

D. No involvement of attached gingiva

E. Normal gum color

42.2013U2A2097 Male, 30 years old, complains: automatic bleeding of the gingiva. Pain for more than 3 days with low-grade fever, the most likely diagnosis in this patient is ( )

A. Interdental papillitis

B. Hypertrophic gingivitis

C. Acute necrotizing gingivitis

D. Herpetic gingivostomatitis

E. Juvenile periodontitis

43.2013U2A2105 Male, 28 years old, red and swollen gums in the anterior teeth area for 1 year, bulbous hypertrophy of the gingival papilla, mouth breathing habits, tartar (+), good general condition. The most likely diagnosis in this patient is ( )

A. Adolescent gingivitis

B. Ulcerative gingivitis

C. Juvenile periodontitis

D. Proliferative gingivitis

E. Drug-induced gingival hyperplasia

44.2013U2A2106 female, 23 years old, with gum pain and easy bleeding in the past 1 week, abnormal bad breath, and necrosis of the papilla on the labial side of the mandibular anterior teeth, the most likely diagnosis and pathogenic bacteria are ( )

A. Gingivitis of pregnancy, Prevotella intermediates

B. Acute necrotizing ulcerative gingivitis, spirochetes and clostridium

C. Chronic gingivitis, Actinomyces viscosus

D. Adolescent gingivitis, Porphyrimonas gingivals

E. Proliferative gingivitis, Actinomyces nepheri

45.2003U2A2083 A 29-year-old male patient is in good health. Complained of spontaneous bleeding from the gums for 1 month, and after staying up late for 3 days, the bleeding was severe and accompanied by gingival pain and halitosis, accompanied by low-grade fever and general malaise. The most likely diagnosis is ( )

A. Marginal gingivitis

B. Herpetic gingivostomatitis

C. Juvenile periodontitis

D. Interdental papillitis

E. Acute necrotizing ulcerative gingivitis

46.2013U2A2108 Male, 55 years old. Severe pain and bleeding in the anterior mandibular teeth for 3 days. Bad taste in the mouth. The patient smokes a lot. On oral examination 43211234 gingival margin on the left and right lower lip sides is like an insect erosion, covered with a gray-brown pseudofilm, which can be wiped off to leave a bleeding surface. The gingival papillae and marginal gingiva are in a straight knife shape. No loosening, no periodontal pockets. There is a peculiar putrid odor in the mouth. Submandibular lymphadenopathy is swollen and tender. Possible diagnoses are ( )

A. Gingival manifestations of acute leukemia

B. Acute papillitis gingivals

C. Acute necrotic ulcerative gingivitis

D. Acute necrotic ulcerative periodontitis

E. Acute herpetic gingivitis

47.2013U2A2109 Male, 30 years old. Spontaneous swelling and pain in the lower left back tooth, bleeding easily when sucking. He complained of picking his teeth with a toothpick 5 days ago. Oral examination: the left 56 interdental papillae are red and swollen with obvious probing pain and easy bleeding. There is no caries and non-caries disease in the tooth. Percussion (+), no loosening, no periodontal pockets probed. Possible diagnoses are ( )

A. Acute pulpitis

B. Acute periodontitis

C. Acute apical periodontitis

D. Acute papillitis gingival

E. Acute necrotizing ulcerative gingivitis

48.2000U2AN128 Male, 27 years old, with sore gums, spontaneous bleeding for 3 days. Examination: putrefactive halitosis, loss and flattening of the tips of the gingival papillae of multiple teeth, and eroded necrosis of the gingival margins of the lower incisors covered with a grayish-white membrane. The most likely diagnosis of the disease is ( )

A. Marginal gingivitis

B. Proliferative gingivitis

C. Acute necrotizing gingivitis

D. Chronic periodontitis

E. Rapidly progressive periodontitis

49.2012U2A1015 The following statements about the main clinical manifestations of ANUG are incorrect ( )

A. The main characteristic lesion is necrosis of the gingival papilla and marginal gingiva

B. The course of the disease is short, generally a few days to 1~2 weeks

C. Lesions tend to involve only the attached gingiva

D. Significant pain

E. Corrupt bad breath

50.2013U2A2111 16-year-old girl, anterior teeth are irregular, crowded, anterior teeth lip side of the papillae are bulbous protrusions, soft and shiny texture, a small amount of plaque, etc. The most likely diagnosis in this patient is ( )

A. Gingival fibromatosis

B. Gingivitis of pregnancy

C. Drug-induced gingival hyperplasia

D. Adolescent gingivitis

E. Leukemia

51.2012U2A1016 ANUG patients who are not treated in a timely and appropriate manner, which bacteria can be co-infected with which bacteria can cause chancre ( )

A. aeruginosa aeruginosa

B. Staphylococcus aureus perfringens

C. Aeroperfringens

D. Escherichia coli

E. mutans

52.2000U2AN131 female, 50 years old. Gingival hyperplasia affects chewing for a year. Examination reveals hypertrophy of the whole mouth gingival hypertrophy, covering about 1/2 of the tooth surface, nodular shape. There was bleeding during probing, and some teeth were displaced in areas where gingival hyperplasia was severe. To aid in the diagnosis, the most important history to ask is ( )

A. Menstrual status

B. Children's situation

C. Dietary status

D. Medication history

E. Epidemiological history

53.2000U2AN132 female, 50 years old. Gingival hyperplasia affects chewing for a year. Examination reveals hypertrophy of the whole mouth gingival hypertrophy, covering about 1/2 of the tooth surface, nodular shape. There was bleeding during probing, and some teeth were displaced in areas where gingival hyperplasia was severe. To aid in the diagnosis, the most important history to ask is a history of medication, and if the patient has had a kidney transplant and has been treated for a year, the most likely diagnosis is ( )

A. Drug-induced gingival hyperplasia

B. Gingival fibromatosis

C. Necrotizing gingivitis

D. Rapidly progressive periodontitis

E. Gum lesions in leukemia

54.2010U2A1046 Manifestations of chronic gingivitis are ( )

A. Significant pain

B. Periodontal pocket formation

C. The teeth are not loose

D. X-rays show horizontal resorption of alveolar bone

E. Tooth displacement

55.2000U2AN133 female, 50 years old. Gingival hyperplasia affects chewing for a year. Examination reveals hypertrophy of the whole mouth gingival hypertrophy, covering about 1/2 of the tooth surface, nodular shape. There was bleeding during probing, and some teeth were displaced in areas where gingival hyperplasia was severe. In order to aid in the diagnosis, the most important medical history to ask is a history of medication, and if the patient has had a kidney transplant and has been treated for a year, the most likely diagnosis is drug-induced gingival hyperplasia, the main cause of which is ( )

A. Immunosuppressant cyclosporine

B. Genetic factors

C. Spirochetes

D. Massive leukocyte infiltration

E. High blood pressure

56.2007U2A1005 Which description is incorrect about ANUG ( )

A. It is more common in young and middle-aged men

B. Grayish-yellow necrotic tissue on the gingival margin

C. Bad breath, easy bleeding or spontaneous bleeding

D. Improper treatment can turn into chronic recurrent attacks

E. X-ray showing alveolar bone resorption

57.2007U2A1006 Causes of acute papillitis gingival do not include ( )

A. Mechanical stimulation

B. Chemical irritation

C. Plaque irritation

D. Poor prosthetic irritation

E. Food impaction stimulation

58.2002U2A1037 The earliest gum disease that can occur in early childhood is ( )

A. Chronic gingivitis

B. Proliferative gingivitis

C. Hereditary gingival fibromatosis

D. Gingival tumors

E. Drug-induced gingival hyperplasia

59.2002U2A1038 Which of the following gum diseases is most common ( )

A. Acute necrotic ulcerative gingivitis

B. Acute papillitis gingivals

C. Chronic gingivitis

D. Gingivitis of pregnancy

E. Gingival tumors

60.2002U2A1039 The clinical manifestations of chronic gingivitis are ( )

A. Periodontal pocket formation

B. Alveolar bone resorption

C. Loose teeth

D. Tooth displacement

E. Probing for bleeding gums

61.2014U2A2096 Female, 25 years old, bleeding from tooth brushing for 3 weeks, examination: gingival papilla mild congestion, edema, tartar (+), most of the probing depth is 2-3mm, and there is no loss of attachment. This patient is most likely to be ( )

A. Interdental papillitis

B. Marginal gingivitis

C. Adolescent gingivitis

D. Juvenile periodontitis

E. Acute necrotizing gingivitis

62.2014U2A1015 Causes of bleeding gums do not include ( )

A. Inflammation

B. Thrombocytopenic purpura

C. Acute leukemia

D. Aplastic anemia

E. Addison's illness

63.2014U2A2098 18-year-old patient complains of bleeding from tooth brushing for several months, found loose teeth (-), red and swollen gums, periodontal probing depth ≤3mm, tartar (++), bleeding on probing, best treatment plan ( )

A. Taking amoxicillin and metronidazole

B. Oral hygiene education and supragagingival hygiene

C. Oral hygiene education, supragingival cleaning, root planing

D. Take periodontine tablets and use gargles

E. Perform gingivectomy

64.2014U2A2099 male, 58 years old, history of hypertension for many years, clinical: congestion and edema of the gingival margin and gingival papilla of the whole mouth, gingival hyperplasia covering 1/2 of the crown, and loose displacement of the lower anterior teeth. The most likely diagnosis is ( )

A. Hypertrophic gingivitis

B. Gingival fibromatosis

C. Rapidly progressive periodontitis

D. Drug-induced gingival hyperplasia

E. Interdental papillitis

1. A。 Reason: During pregnancy, surgical resection is usually performed in the second trimester (4~6 months), when the fetus is relatively stable and the surgical risk is low.
2. C。 Causes: Mild chronic gingivitis primarily affects free gingiva and papillae rather than attached gingiva.
3. D。 Causes: Chronic gingivitis is the most common form of gum disease and is often caused by plaque.
4. A。 Causes: In acute papillitis, mild percussion usually indicates inflammation and edema of the periodontal ligament below the papilla.
5. B。 Causes: The typical clinical manifestations of proliferative gingivitis are hypertrophy of the gingival margin and bulbous hyperplasia of the gingival papillary, and bleeding is susceptible to probing.
6. E。 Causes: Gingivitis is characterized by the presence of tartar and soft plague deposits around the necks of teeth, leading to inflammation of the gums.
7. C。 Causes: The symptoms described are consistent with chronic gingivitis, which is characterized by the disappearance, indentation of the gingival papillae, and the presence of tartar and tartar.
8. C。 Causes: Adolescent gingivitis tends to occur on the labial margins of the anterior teeth and the papillae, as these areas are more prone to plaque accumulation.
9. D。 Causes: The main causative factor of gingivitis in adolescence is plaque, as hormonal changes can increase the risk of plaque formation and gingivitis development.
10. B。 Causes: Gingivitis in pregnancy may be associated with Pruvotea intermediates because of the high detection rate of plaque in gingivitis in pregnancy.
11. B。 Causes: The bacterium with the highest detection rate of plaque in pregnant gingivitis women is Porphyromonas gingivals.
12. A。 Causes: The described symptoms, such as hyperplasia of the gingival margin and gingival papilla on the labial side of the upper anterior teeth, covering 1/3 of the crown, are tough in texture, and are consistent with the characteristics of hypertrophic gingivitis.
13. D。 Causes: Gingival tumors during pregnancy are common in the labial papillae of the lower anterior teeth because this area is more susceptible to hormonal changes.
14. A。 Causes: In the diagnosis of acute necrotizing gingivitis, Gram stain on the smear is helpful and helpful in detecting specific bacterial types.
15. D。 Causes: The main causative microorganisms of acute necrotizing gingivitis are Fusobacterium and spirochetes.
16. D。 Causes: Metronidazole is the best drug choice for the treatment of acute necrotizing gingivitis because it is effective against anaerobic bacteria such as Fusobacterium.
17. C。 Causes: When removing gingival tumors during pregnancy, whether or not to take medication during pregnancy is not a major consideration, as the risks of surgery are more influenced by other factors.
18. C。 Reason: The timing of surgical resection of gingival tumor during pregnancy is usually selected at 4~6 months of pregnancy, and it is relatively safe to perform surgery at this time.
19. E。 Causes: The gums of people with leukemia usually do not affect the whole mouth, and the main symptoms are pale or dark red gums and a tendency to bleed.
20. D。 Causes: Gingivitis during pregnancy is most likely to manifest clinically as bright red, shiny gums, as changes in hormone levels during pregnancy can cause this manifestation.
21. E。 Causes: Leukemia is usually diagnosed by checking blood tests, which can be seen with abnormal increases in white blood cells.
22. E。 Causes: Leukemia is less common in oral manifestations of herpes on the surface of the gums, more often with soft, bleeding or bleeding tendencies.
23. B。 Reason: The principle of treatment of gingival lesions in leukemia is conservative treatment, including hemostasis and anti-infection.
24. C。 Causes: The anaerobic organisms with the largest increase in the number of lesions in acute necrotizing gingivitis are Fusobacterium and spirochetes.
25. D。 Causes: To further confirm the diagnosis, routine blood tests should be done promptly, especially if there is an acute inflammatory response.
26. D。 Causes: Metronidazole is the most sensitive bacteriostatic agent for the treatment of acute necrotizing ulcerative gingivitis because it is effective against anaerobic bacteria.
27. E。 Causes: The most likely clinical findings of gingivitis during pregnancy are bright red, soft and shiny gingival margins and nipples.
28. E。 Causes: Swollen gums in leukemia are mainly caused by the infiltration and aggregation of immature, non-functioning white blood cells.
29. A。 Causes: The main pathological manifestation of gingival lesions in leukemia is that the gums are filled with a large number of naïve white blood cells.
30. E。 Causes: Gentle irrigation with a 3% hydrogen peroxide solution may cause irritation to the gums and should not be used in the gingival presentation of acute leukemia.
31. B。 Causes: In drug-induced gingival hyperplasia, the increased secretion of collagen-lytic enzyme is incorrect, as collagen synthesis is actually increased.
32. E。 Causes: The clinical manifestations of drug-induced gingival hyperplasia can affect the whole mouth teeth, and the gingival texture is firm and elastic, and it is spherical or nodal growth.
33. B。 Causes: The degree of drug-induced gingival hyperplasia is significantly related to the duration of medication, and the longer the time, the more significant the hyperplasia may be.
34. E。 Causes: Phenytoin, nifedipine, and cyclosporine can all cause drug-induced gingival hyperplasia, and the combination may increase the risk.
35. A。 Causes: Cyclosporine is one of the drugs known to cause gum hyperplasia.
36. C。 Causes: The use of certain antihypertensive drugs, such as nifedidine (nifedipine), in people with high blood pressure, can cause drug-induced gingival hyperplasia.
37. A。 Causes: Gingivitis during pregnancy is mainly caused by endocrine changes, which lead to changes in hormone levels that affect gingival response.
38. E。 Causes: Pregnancy itself does not directly cause gum inflammation, but rather hormonal changes during pregnancy that affect the gums' response to plaque.
39. A。 Causes: Nifedidine (niferidine) is a drug known to cause gingival hyperplasia.
40. B。 Causes: Systemic antibiotics are not routine treatment for drug-induced gingival hyperplasia unless an infection has occurred.
41. E。 Causes: Gingival fibromatosis usually presents with normally colored gums but growths covering the crown.
42. C。 Causes: Symptoms such as autobleeding, pain, and low-grade fever may indicate acute necrotizing gingivitis.
43. D。 Causes: Red and swollen gums in the anterior area, spherical hypertrophy of the papilla with tartar, and good general condition, the most likely diagnosis is proliferative gingivitis.
44. B。 Causes: Symptoms described such as sore gums, easy bleeding, papillary necrosis of the gingival papillary, most likely acute necrotizing ulcerative gingivitis, caused by spirochetes and Fusobacterium.
45. E。 Causes: Symptoms such as spontaneous bleeding, pain, halitosis, low-grade fever, and malaise are consistent with acute necrotizing ulcerative gingivitis.
46. C。 Causes: Symptoms described such as severe pain, bleeding, foul mouth odor, grayish-brown pseudomembrane covering, consistent with acute necrotizing ulcerative gingivitis.
47. D。 Causes: Redness and swelling of the interdental papilla, obvious probing tenderness and easy bleeding, positive percussion, may be acute gingival papillitis.
48. C。 Causes: Loss and flattening of the tip of the gingival papilla, colonial margin worm-like necrosis, grayish-white membrane covering, characteristic of acute necrotizing gingivitis.
49. C。 Causes: The characteristic lesions of ANUG (acute necrotizing ulcerative gingivitis) are mainly necrosis of the gingival papillae and marginal gingiva and are not limited to the attached gingiva.
50. D。 Cause: 16-year-old girl with misaligned teeth and bulbous protrusions of interdental papillae on the labial side of the anterior teeth, the most likely diagnosis is adolescent gingivitis.
51. E。 Causes: ANUG is not treated promptly and appropriately, and co-infection with Streptococcus mutans can lead to chancre.
52. D。 Causes: Considering the hypertrophy of the whole mouth gingival hypertrophy, nodularity, and bleeding on probing, the most important medical history that should be noted is the history of medication.
53. A。 Causes: Long-term immunosuppressive therapy, such as cyclosporine, may result in drug-induced gingival hyperplasia in kidney transplant patients.
54. E。 Causes: Chronic gingivitis is often absent from tooth displacement, mainly manifested by bleeding on probing without tooth loosening.
55. A。 Causes: Drug-induced gingival hyperplasia is mainly due to the use of immunosuppressants such as cyclosporine.
56. E。 Causes: ANUG (acute necrotizing ulcerative gingivitis) does not usually show alveolar bone resorption on x-rays.
57. B。 Causes: The etiology of acute papillitis gingival is usually not related to chemical irritation and is mainly caused by machinery, plaque, etc.
58. A。 Causes: Chronic gingivitis can occur in early childhood and is the earliest gum disease that can occur.
59. C。 Causes: Chronic gingivitis is the most common gum disease.
60. E。 Causes: The typical clinical presentation of chronic gingivitis is probing gingival bleeding.
61. B。 Causes: mild hyperemia, edema, probing depth of 2-3 mm, no loss of attachment, most likely marginal gingivitis.
62. E。 Causes: Addison's disease does not directly cause bleeding gums, it is adrenal insufficiency.
63. B。 Causes: Oral hygiene education and supragingival scaling are the best treatment options, as the symptoms are mainly gingival inflammation and tartar deposition.
64. D。 Causes: In patients with long-term hypertension, there is clinical gingival hyperplasia covering the crown, which may be drug-induced gingival hyperplasia.
65. C。 Causes: Given the patient's age and symptoms of gingival hyperplasia, a history of hypertension should be emphasized, as certain antihypertensive drugs may cause drug-induced gingival hyperplasia.
66. B。 Causes: Oval growths, bleeding on probing, and x-rays show decreased alveolar bone mineral density, which is more consistent with gingival tumors.
67. E。 Causes: Patients with necrotizing gingivitis may present with putrefactive halitosis, low-grade fever, decreased body resistance, and submandibular lymphadenopathy, but the presence of protoblasts in peripheral blood is not a feature.
68. B。 Causes: A history of acute hepatitis is not very helpful in diagnosing gingival hyperplasia, and more attention should be paid to information related to drug use, such as pregnancy history and epilepsy history.
69. E。 Causes: Emergency management of acute necrotizing ulcerative gingivitis should include mild removal of necrotic tissue and initial scaling of large pieces of tartar and irrigation with a 3% hydrogen peroxide solution, while systemic medical therapy is considered.
70. E。 Causes: Symptomatic management of fish stab wounds can include removal of irritants such as tartar, alternating rinsing with 3% hydrogen peroxide solution and normal saline, topical application of 2% iodine glycerin, and gargling with 0.2% chlorhexidine solution.
71. E。 Causes: The clinical features of gingival hypertrophy include dark red or dark red gingiva, soft and inflamed gingiva, smooth surface, hypertrophy of the gingival margin, bulbous gingival papillae, often covering 1/3 or more of the crown of the tooth near the neck, and easy bleeding.
72. E。 Causes: Gingivitis in pregnancy can be caused by a variety of causes, including endocrine changes, occlusal trauma, pregnancy itself, and malnutrition.
73. C。 Causes: Clinical changes in gingivitis during pregnancy are consistent with progesterone levels, as changes in progesterone levels can affect gingival response.
74. D。 Causes: Changes in sex hormone levels during pregnancy can exacerbate or alter the characteristics of pre-existing chronic gingivitis.
75. B。 Causes: Women on long-term oral contraceptives may experience gingivitis symptoms similar to those of pregnancy due to changes in hormone levels.
76. C。 Causes: when the history is sold, a history of hemichewing is not very helpful in diagnosing the cause of generalized gingival hyperplasia throughout the mouth.
77. C。 Causes: Bleeding from continuous tooth brushing, bleeding when touching while eating, probing depth of ≤3mm, no alveolar crest resorption, consistent with the characteristics of chronic gingivitis.
78. D。 Causes: The main causative bacteria of acute necrotizing ulcerative gingivitis are Fusobacterium and spirochetes.
79. D。 Causes: Acute gingival abscess usually does not cause general redness and swelling of the oral mucosa, ulcers, and pseudomembranes.
80. A。 Causes: In the diagnosis of acute necrotizing ulcerative gingivitis, plaque smear is the most valuable adjunct.
81. D。 Causes: Bacteriological smears of lesions are commonly used as an adjunct to the diagnosis of acute necrotizing gingivitis.
82. E。 Causes: The best treatment for the first time is to remove large pieces of tartar and necrosis and rinse with 3% hydrogen peroxide to control infection and inflammation.
83. B。 Causes: Clinical features of acute necrotizing ulcerative gingivitis include knife-cut lesions of the gingival papilla, painful bleeding, and putrefactive halitosis.
84. B。 Causes: Chronic gingivitis usually does not involve the formation of gingival pockets greater than 3 mm deep, which is more common in periodontitis.
85. A。 Causes: Treatment of acute multiple gingival abscess usually includes topical therapy with antibiotic therapy.
86. A。 Causes: Bone marrow images are usually not the primary basis for differentiating gingival hyperplastic disorders.
87. D。 Causes: Gingivitis in pregnancy is most likely to manifest clinically as bright red and shiny gums.
88. D。 Causes: The most closely related factor to the development of gingivitis during pregnancy is the changes in sex hormones in the body.
89. A。 Causes: The high point of the occlusal filling is a factor causing periodontitis and not directly causing chronic gingivitis.
90. A。 Causes: Cyclosporine A is one of the drugs closely associated with drug-induced gingival hypertrophy.
91. E。 Causes: Drug-proliferative gingival hypertrophy usually begins in the buccal gingival papillae.
92. E。 Causes: Morphine does not cause drug-induced gingival hyperplasia.
93. A。 Causes: Gingival lesions in leukemia are usually global rather than localized.
94. E。 Causes: Acute necrotizing ulcerative gingivitis is short-lived, rapidly progressive, and does not persist for a long time, and loose teeth are usually not an early symptom.
95. A。 Causes: Cyclosporine-induced gingival hyperplasia has more components of blood vessels and chronic inflammatory cells in the tissue, which is different from phenytoin-induced hyperplasia.
96. B。 Causes: Normal neutrophils are unlikely to be seen in gingival lesions in leukemia.
97. E。 Causes: Although chlorhexidine gargle has a bitter taste, it does not cause long-term changes in taste, and the effects are usually short-lived.
98. C。 Why: The gingival sulcus bleeding index is a better indicator of the activity of gingivitis because it is directly related to the health of the gums.

**Chapter 10 Periodontitis**

1. 2009U2A1054 Which is wrong to describe rapidly progressive periodontitis ( )

A. Onset is between adolescence and 35 years

B. Lesions are diffuse and involve most teeth

C. Severe and rapid bone destruction that continues to develop

D. Most patients have neutrophil dysfunction

E. Sometimes accompanied by systemic symptoms

2.2009U2A1056 Palmoplantar keratosis-periodontal destruction syndrome is characterized by ( )

A. There is significant chronic inflammation of the wall of the periodontal pocket

B. Osteoclast activity is evident and osteogenic activity is low

C. Predominantly plasma cell infiltration

D. The cementum at the root of the affected tooth is very thin

E. All of the above

3.2009U2A1073 Main causes of periodontitis in adolescents ( )

A. There are special microorganisms in the plaque

B. Defective neutrophil chemotaxis

C. Familial hereditary

D. Neutrophil phagocytosis defect

E. All of the above

4.2009U2A1075 Clinical manifestations of juvenile periodontitis exclude ( )

A. The age of onset is more common from adolescence to 25 years of age

B. Tartar irritation and gum inflammation are obvious

C. Significant tooth displacement

D. Significant loosening of teeth

E. Deeper periodontal pockets on the adjacent surface

5. 2016U2A1001 Palmoplantar Keratosis-Periodontal Destruction Syndrome belongs to ( )

A. Autosomal recessive inheritance

B. Autosomal dominant inheritance

C. Sex chromosome recessive inheritance

D. Sex chromosome dominant inheritance

E. Genetic disorders are not associated with plaque

6.2016U2A1015 Which is wrong in the description of the clinical manifestations of palmoplantar keratosis-periodontal destruction syndrome ( )

A. Skin and periodontal lesions usually co-occur before the age of 4 years

B. The child is intellectually disabled but has normal physical development

C. Invasion of both primary and permanent teeth

D. There are deep periodontal pockets with rapid alveolar bone resorption

E. This disease is rare, and the prevalence in the population is about 1/1 million ~ 4/1 million

7.2016U2A1018 Which of the following is false about the narrative of juvenile periodontitis ( )

A. It occurs mainly in young people from adolescence to 25 years of age, and is more common in women than in men

B. In early patients, plaque and tartar are scarce, and the degree of periodontal tissue destruction is not proportional to the amount of local irritants

C. X-ray showed that the first molars were absorbed horizontally in the near and distal directions, and the incisors were mostly in an arc of absorption

D. The course of the disease progresses rapidly

E. There may be a family history of hereditary history

8.2016U2A1025 Juvenile periodontitis, tooth displacement direction is ( )

A. Upper incisor labial displacement

B. Labial displacement of the lower incisor

C. Distal displacement of the upper incisors

D. Distal displacement of the lower incisors

E. Distal displacement of the labial side of the upper and lower incisors

9. 2008U2A1007 Characteristic manifestations of early juvenile periodontitis do not include ( )

A. Bleeding gums

B. It is more common in adolescents between adolescence and 25 years of age

C. The amount of plaque and tartar is small, and the gingival inflammation is mild, but there are deep periodontal pockets

D. X-rays showing typical "arc absorption" of the first molars

E. Early appearance of loose and displaced teeth

10. 2008U2A1016 Closely related to the occurrence of localized juvenile periodontitis is ( )

A. Aggregator actinomyces

B. Actinomycetes viscosus

C. prawela intermediates

D. Porphyrimonas gingivals

E. Pseudobacterium fersa

11.2000U2A1008 Periodontitis dominated by alveolar bone resorption is ( )

A. Compound periodontitis

B. Advanced adult periodontitis

C. Juvenile periodontitis

D. Rapidly progressive periodontitis

E. Periodontal abscess

12.2015U2A1002 The most obvious loosening and displacement of teeth in juvenile periodontitis is ( )

A. Mandibular premolars

B. Mandibular molars

C. Mandibular anterior teeth

D. Maxillary molars

E. Maxillary anterior teeth

13.2015U2AN137 male, 33 years old, chief complaint: In the past 1~2 years, he felt that his whole mouth teeth were loose, his chewing strength was weakened, and his front teeth were displaced. The most likely initial impression is ( )

A. Chronic periodontitis

B. Rapidly progressive periodontitis

C. Juvenile periodontitis

D. Acute multiple periodontal abscess

E. Acute necrotizing gingivitis

14.2015U2A2074 female, 33 years old, chief complaint: In the past 1~2 years, most of the teeth in the whole mouth have been loosened and the chewing ability has weakened. If rapidly progressive periodontitis is diagnosed, the most likely findings on clinical examination are ( )

A. Most teeth are displaced

B. Multiple periodontal abscesses

C. Molar root bifurcation lesion 3rd degree

D. Most alveolar bones are moderately and severely absorbed

E. Most gingival recession is significant

15.2015U2A2075 female, 28 years old, X-ray: alveolar bone destruction of 6 and 12 above and below left and right, but the alveolar bone of other teeth was also damaged, and the degree varied. The most likely diagnosis for this patient is ( )

A. Chronic periodontitis

B. Rapidly progressive periodontitis

C. Juvenile periodontitis

D. Acute necrotizing gingivitis

E. Multiple periodontal abscesses

16.2015U2AN138 male, 34 years old, complained: In the past 1~2 years, he felt that his teeth were loose and his chewing strength was weakened. If the patient is diagnosed with rapidly progressive periodontitis, the most common cause of loose teeth in this patient is ( )

A. Inflammation of the gums

B. Resorption of alveolar bone

C. Primary occlusal trauma

D. Secondary occlusal trauma

E. Periodontal ligament inflammation

17.2010U2A1038 Which of the following patients with periodontal disease is more likely to have peripheral blood neutrophil dysfunction ( )

A. Chronic gingivitis

B. Juvenile periodontitis

C. Periodontitis in adults

D. Acute necrotic ulcerative gingivitis

E. Periodontal atrophy

18.2015U2A1005 Periodontal maintenance interval for juvenile periodontitis, initial should be ( )

A. 1~2 months

B. 4~6 months

C. 6~8 months

D. 8~10 months

E. 6~12 months

19.2015U2A1009 The most characteristic marker that distinguishes early periodontitis from gingivitis is ( )

A. Visiting ≥ 3 dishes

B. Bleeding on probing

C. Loose gums

D. Weakness in chewing

E. Whether the enamel cementum boundary can be explored

20.2013U2A1007 Compound Periodontitis ( )

A. Both localized and systemic damage

B. Both supra-bony and subbony pockets

C. Gingivitis and periodontitis are present at the same time

D. Inflammatory damage coexists with trauma

E. None of the above

21.2013U2A2100 female, 21 years old, distal displacement of upper incisors, widening of the gap, sought treatment. Examination: periodontal probing of the upper incisors was 6mm, the first permanent molars were loosened I degree, periodontal probing was 6mm, and X-rays showed that the alveolar bone in the upper anterior area was horizontally resorpted, and the first molars were arc-shaped resorption. Diagnosed as ( )

A. Rapidly progressive periodontitis

B. Chronic periodontitis

C. Localized juvenile periodontitis

D. Preadolescent periodontitis

E. Diffuse juvenile periodontitis

22. 2013U2A2102 patient, female, 23 years old. Complaints of chewing weakness. oral examination; The periodontal pocket of the upper and lower first molars was 6 mm, loose I.°, plaque index and gingival index 1, and the preliminary diagnosis was juvenile periodontitis. The main pathogens of the disease are ( )

A. Actinobacillus actinobacillus

B. Prevotella intermediates

C. Nuclusionella

D. Porphyromonas gingivals

E. Campylobacter rectum

23.2013U2A2104 Male, 17 years old, bleeding from tooth brushing, loose and displaced front teeth for 1 year, sometimes swollen and painful gums. If you want to diagnose juvenile periodontitis, you also need to check ( )

A. Attachment level

B. Periodontal pocket depth

C. X-rays

D. Occlusal relationship

E. All of the above examinations are required

24.2010U2A1047 The microorganisms that play an important role in AIDS-related periodontal disease are ( )

A. Streptococcus

B. Candida albicans

C. Viruses

D. Chlamydia

E. Mycoplasma

25.2007U2A1007 Periodontitis is now recognized as ( )

A. Infectious diseases with multifactorial pathogenesis

B. Geneticly related diseases

C. Tissue degeneration with bacterial infection

D. Occlusal trauma is one of the main causative factors-

E. Diseases caused by bacteria and their products

26.2002U2A1040 The main clinical symptoms of early adult periodontitis patients are ( )

A. Tooth displacement

B. Food impaction

C. Bleeding gums when brushing or eating

D. Gum recession

E. Loose teeth

27.2002U2A1041 Factors to be excluded in the diagnosis of rapidly progressive periodontitis do not include ( )

A. Whether there is a serious malocclusion

B. Whether the age is between puberty and 25 years

C. Whether or not incorrect orthodontic treatment has been received

D. Presence of significant local contributing factors, such as food impaction

E. Whether there is a concomitant systemic disease such as diabetes mellitus

28.2002U2A1042 Clinical features of juvenile periodontitis include ( )

A. It is more common in adolescents between adolescence and 25 years of age

B. Prediscriminate in the first permanent molars and upper and lower incisors

C. The lesion progresses rapidly, and the tooth is loose and displaced in the early stage

D. X-ray showing arc bone resorption of the first permanent molar

E. All of the above

29.2002U2A1043 Localized juvenile periodontitis is ( )

A. Maxillary incisors

B. Mandibular incisors

C. Premolars and upper and lower incisors

D. First molars and upper and lower incisors

E. Second molars and upper and lower incisors

30.2014U2A2097 Female 20 years old, complaining of masticatory weakness, periodontal pocket 6mm on the left and right upper 6 buccal sides of oral examination, loose II.°, general oral hygiene, calculus I°, probable diagnosis ( )

A. Periodontitis in adolescence

B. Chronic gingivitis

C. Simple periodontitis

D. Compound periodontitis

E. None of the above

31.2014U2A2106 Male, 43 years old, bleeding from tooth brushing for 2 years, soreness from hot and cold stimuli, no spontaneous pain, nocturnal pain, and weak occlusion of lower anterior teeth for half a year. Examination: the tartar index is 3, the gingiva is congested and swollen, the lower anterior teeth are loose II., the gingival recession is 2mm, the periodontal probing depth of the upper and lower molars is 4~5mm, and the diagnosis should be ( )

A. Gingivitis

B. Chronic periodontitis

C. Rapidly progressive periodontitis

D. Pulpitis

E. Diffuse juvenile periodontitis

32.2014U2A2107 Male, 38 years old. Bleeding from the whole mouth, weakness to chew. And have just completed thorough periodontal system treatment. No significant systemic disease. Oral examination: the depth of the periodontal pocket was increased compared with that before treatment, and the X-ray showed that alveolar bone resorption had developed significantly. Good oral hygiene. Initial diagnosis is ( )

A. Juvenile periodontitis

B. Periodontitis associated with diabetes

C. Periodontitis in adults

D. Rapidly progressive periodontitis

E. Refractory periodontitis

33.2014U2A2108 Male, 17 years old. complained of tooth bleeding and chewing weakness for more than 1 month. Oral examination: incisors and first molars are loosened I.°, incisors are displaced labially, periodontal pockets are 5~6mm, periodontal pockets of first molars are 6mm, plaque index and gingival index are 1, and gingival bleeding is probed. Initial diagnosis is ( )

A. Periodontitis in adults

B. Juvenile periodontitis

C. Rapidly progressive periodontitis

D. Adolescent gingivitis

E. Prepubertal periodontitis

34.2014U2A1016 Juvenile periodontitis is characterized by ( )

A. More common in adolescent girls

B. Lots of tartar

C. Severe gum inflammation

D. It is more common to have fangs

E. Alveolar bone is mostly absorbed horizontally

35.2014U2A1017 The perceived symptoms of patients in the early stage of juvenile periodontitis are often ( )

A. Weakness in tooth chewing

B. Significant bleeding from the gums

C. Significant redness and swelling of the gums

D. Bad breath

E. Periodontal pocket discharge

1. D。 Neutrophil deficiencies are more common in systemic disease than specific to rapidly progressive periodontitis.
2. E。 Palmoplantar keratinization-periodontal destruction syndrome involves chronic inflammation of the periodontal pocket wall, increased osteoclast activity and decreased osteogenic activity, and cementum thinning.
3. E。 Juvenile periodontitis can be caused by a variety of factors, including specific organisms, neutrophil dysfunction, and hereditary properties.
4. E。 The clinical manifestations of juvenile periodontitis do not usually include a deep periodontal pocket on the adjacent surface, which is more common in adult periodontitis.
5. B。 Palmoplantar keratinization-periodontal destruction syndrome is an autosomal dominant disorder.
6. B。 Intellectual disability is not characteristic of palmoplantar keratinization-periodontal destruction syndrome, which primarily affects the skin and periodontal.
7. A。 Juvenile periodontitis does not particularly skew any gender and can affect both sexes.
8. E。 Tooth displacement in juvenile periodontitis usually does not have a specific orientation and can include labial and distal displacement of the upper and lower incisors.
9. E。 Early juvenile periodontitis is uncommon with loose and displaced teeth, which are signs of disease progression.
10. E。 Bacteroides fersa is closely associated with the occurrence of localized juvenile periodontitis.
11. D。 Rapidly progressive periodontitis is characterized by alveolar bone resorption.
12. C。 In juvenile periodontitis, the loosening and displacement of teeth is most pronounced in the anterior mandibular teeth.
13. B。 Rapidly progressive periodontitis can lead to loosening of teeth and weakened chewing power throughout the mouth.
14. D。 Clinical examination of rapidly progressive periodontitis is most likely to reveal moderate to severe resorption of alveolar bone.
15. B。 Symptoms and x-ray descriptions are consistent with features of rapidly progressive periodontitis.
16. B。 The most common cause of tooth loosening is resorption of alveolar bone.
17. B。 Neutrophil deficits are more common in juvenile periodontitis.
18. A。 The periodontal maintenance interval for juvenile periodontitis should be short, with an initial period of 1~2 months.
19. A。 The most characteristic marker that distinguishes early periodontitis from gingivitis is the probing depth of ≥3 mm.
20. B。 Compound periodontitis refers to the presence of both supra-bone and sub-bone pockets.
21. C。 The diagnosis of localized juvenile periodontitis is based on the patient's age, symptoms, and radiographic findings.
22. D。 Porphyromonas gingivalis is the main causative organism of juvenile periodontitis.
23. E。 Diagnosing juvenile periodontitis requires a thorough examination, including the level of attachment, the depth of the pockets, x-rays, and assessment of the occlusal relationship.
24. B。 Candida albicans plays an important role in AIDS-associated periodontal disease.
25. A。 Periodontitis is a multifactorial infectious disease.
26. C。 The main clinical symptom in early adult periodontitis is bleeding gums when brushing or eating.
27. B。 Age between puberty and 25 years of age is not the first factor to rule out in diagnosing rapidly progressive periodontitis.
28. E。 The clinical features of juvenile periodontitis include the rapid progression of lesions, loosening and displacement of teeth in the early stage, and X-ray showing arc resorption of alveolar bone.
29. D。 Localized juvenile periodontitis tends to occur with the first molars and upper and lower incisors.
30. D。 Compound periodontitis may be the most appropriate diagnosis, given the patient's age and symptoms, as well as periodontal condition.
31. B。 Considering the patient's symptoms and clinical presentation, chronic periodontitis is a possible diagnosis.
32. D。 Rapidly progressive periodontitis is a possible diagnosis, given the increased pocket-depth and marked development of alveolar bone resorption, and the absence of systemic disease.
33. B。 Juvenile periodontitis is a reasonable initial diagnosis given age, clinical presentation, and symptoms.
34. E。 One of the characteristics of juvenile periodontitis is that the alveolar bone is mostly absorbed horizontally.
35. B。 In the early stages of juvenile periodontitis, the symptom is often marked bleeding from the gums.

**Chapter 11 Associated Lesions of Periodontitis**

1.2009U2A1053 Pathogenesis factors of periodontal abscess do not include ( )

A. Purulent inflammation of the wall of the periodontal pocket develops deeply, and pus cannot drain out of the pocket

B. The periodontal pocket is tortuous and involves multiple tooth surfaces, and the purulent exudate cannot be drained smoothly

C. Scaling, scaling is rough, pushing the tartar fragments deep into the periodontal pocket

D. The foreign body penetrates the interdental papilla, causing purulent inflammation

E. Perforation of the medullary floor or root canal wall during root canal treatment, causing purulent inflammation

2.2009U2A1067 Pathogenesis associated with root bifurcation lesions includes ( )

A. Plaque and tartar

B. Occlusal trauma

C. Anatomical morphology of the tooth root

D. Spread of pulp infection

E. All of the above

3.2016U2A1010 Key points for differentiating periodontal abscess from alveolar abscess do not include ( )

A. Presence or absence of deep periodontal pockets

B. Pulp viability

C. Percussion

D. X-ray findings

E. Degree of gum inflammation

4.2008U2A1010 Main causative factors of root bifurcation lesions ( )

A. Anatomical morphology of the tooth root

B. Trauma

C. Plaque

D. Enamel protrusion in the neck of the tooth

E. Pulp infection of molars spreads through the accessory root canal at the base of the pulp chamber to the root bifurcation zone

5.2000U2A1007 The following periodontal diseases are caused by local irritants, except ( )

A. Simple gingivitis

B. Periodontitis in adults

C. Multiple gingival abscesses

D. Gingival papillitis

E. hypertrophic gingivitis

6.2000U2AN125 female, 60 years old, gum swelling and pain for 2 weeks, had swelling and pain 1 year ago, but no treatment. Examination: The gingiva on the lower left 6 buccal sides is swollen, there is a fistula, the fistula points to the apical direction, and there are periodontal pockets of 5~6mm in the center of the buccal side, distal middle and lingual side. An important test to confirm the diagnosis is ( )

A. Bleeding on probing

B. Tooth looseness

C. X-rays

D. Probing of root forks

E. Probe the subgingival calculus

7.2000U2AN126 female, 60 years old, gum swelling and pain for 2 weeks, had swelling and pain 1 year ago, but no treatment. Examination: The gingiva on the lower left 6 buccal sides is swollen, there is a fistula, the fistula points to the apical direction, and there are periodontal pockets of 5~6mm in the center of the buccal side, distal middle and lingual side. An important test to confirm the diagnosis is to take x-rays, and the most likely diagnosis of the tooth is ( )

A. Pulpitis

B. Chronic periodontitis

C. Marginal gingivitis

D. Root bifurcation lesions

E. Combined periodontal-pulp lesions

8. 2015U2A2070 A 25-year-old patient with poor oral hygiene, the first permanent molars in the left lower left had a pulp-periodontal joint lesion, severe pain, but no loosening of the affected tooth. In order to eliminate the pain and restore health, the first measure taken is to open the marrow and drain the marrow to stop the inflammation from spreading. The above treatment measures are tertiary prevention. At the same time, it should also be taken ( )

A. Desensitization of the first permanent molar in the lower left

B. Filling of the lower left first permanent molar

C. Periodontal treatment of the lower left first permanent molar

D. Occlusion of the first permanent molar in the lower left

E. Extraction of the first permanent molar in the lower left

9.2015U2A2077 male, 45 years old, after treatment by experienced doctors, bleeding was still detected at the root bifurcation of the right upper 6 cheeks. Causes affecting the prognosis of root bifurcation lesion treatment, except ( )

A. The root fork entry is too small

B. Enamel process

C. Patient is not flossing

D. Incomplete removal of plaque tartar

E. Most molars have root bifurcation close to the cementum of the enamel tooth

10.2015U2A2078 A patient, complaining: swollen and painful gingiva in the right upper posterior tooth for 2 days. Examination: oval swelling of the gingiva on the right upper 6 cheeks, fluctuating sensation (+) PD: 7mm, pus leakage in the probing bag, and normal electrical viability measurement. The most likely diagnosis is ( )

A. Acute alveolar abscess

B. Acute multiple periodontal abscess

C. Retrograde pulpitis

D. Acute periodontal abscess

E. Root bifurcation lesions

11.2004U2A1064 The local reasons affecting the treatment effect of root bifurcation lesions are mainly ( )

A. Anatomical features

B. Bacteria are more virulent

C. High number of bacteria

D. Occlusal surface morphology

E. The number of roots is high

12.2010U2A1037 The following methods of treatment of acute periodontal abscess are inappropriate ( )

A. Abscess incision

B. Gingival flap surgery

C. Anti-inflammatory and analgesic

D. Adjust the occlusion

Topical irrigation of periodontal pockets

13.2013U2A1006 IV. Root bifurcation lesions, which treatment is not needed ( )

A. Gingivectomy

B. Periodontal flap surgery

C. Cleansing

D. Curettage

E. Root planing

14.2013U2A2099 Adult male, diagnosed with retrograde pulpitis, whether the affected tooth can be preserved mainly depends on ( )

A. Patient's financial status

B. Efficacy of endodontic therapy

C. Patient's general condition

D. Oral hygiene habits

E. Extent of periodontal lesions and prognosis

15.2013U2A2101 female, 47 years old, has been diagnosed with the third degree root bifurcation lesion of the left lower first molar, loose (-), generally not used ( )

A. Guided tissue regeneration

B. Root splitting

C. Tunneling

D. Pocket wall resection

E. Root reduction flap surgery

1. C。 Because scaling is usually aimed at removing tartar and bacteria from the periodontal pocket, rather than pushing it deep in.
2. E。 Factors contributing to root bifurcation include plaque and tartar, occlusal trauma, root anatomy, and spread of pulp infection.
3. E。 The degree of gingival inflammation is characteristic of periodontal disease, and the differentiation of periodontal abscess from alveolar abscess should focus on deep periodontal pockets, pulp viability, percussion, and x-ray findings.
4. E。 Pulp infection of molars may be the main causative factor for spreading through the pararoot canal at the base of the pulp chamber to the root bifurcation region.
5. A。 Simple gingivitis is usually caused by plaque rather than a local irritant.
6. C。 X-rays can help determine the condition of the teeth and surrounding tissues and are important for diagnosis.
7. B。 Based on symptoms and examination results, chronic periodontitis is the most likely diagnosis.
8. C。 Periodontal lesions should be treated first to eliminate the cause and stop the inflammation from spreading.
9. E。 The proximity of root bifurcation to the cementum boundary of most molars does not directly affect the prognosis of the treatment of root bifurcation lesions.
10. D。 Symptoms of acute periodontal abscess include swollen and painful gums and pus leaking in the probing pocket.
11. A。 The treatment effect of root bifurcation lesions is mainly affected by anatomical characteristics.
12. B。 Gingival flap surgery is usually not the preferred treatment for acute periodontal abscesses.
13. A。 IV. Lesions in the area of the root bifurcation usually do not require gingivectomy.
14. B。 Whether the tooth in retrograde pulpitis can be preserved depends mainly on the efficacy of endodontic treatment.
15. D。 In the case of loose (-), pouch wall resection is generally not used.

**Chapter 15 Basic Periodontal Treatment**

1.2009U2A1040 The purpose of root surface planarization is ( )

A. Removal of root surface infected cementum

B. Removal of residual tartar

C. Remove granulation tissue from the inner wall of the bag

D. Remove subgingival plaque

E. All of the above

2.2009U2A1041 No need to do after cleaning ( )

A. Use a pointed probe to check whether the tartar has been removed

B. Rinse the periodontal pocket with 3% hydrogen peroxide solution

C. Intragingival sulcus application

D. Immediate root desensitization

E. Check the gums for damage

3.2009U2A1043 The focus of the basic treatment of periodontal disease is ( )

A. Medication

B. Occlusal adjustment

C. Plaque control

D. Loose tooth fixation

E. Treatment of food impaction

4.2009U2A1049 Selective grinding method in food impaction treatment is suitable for ( )

A. All vertical food impaction

B. Partial vertical food impaction

C. All horizontal food impaction

D. Partial horizontal food impaction

E. B+C

5.2009U2A1057 Methods that do not belong to self-control plaque are ( )

A. Brush your teeth

B. Gargle

C. Use of dental floss

D. Cleanliness

E. Use of toothpicks

6.2009U2A1059 Wrong grinding method ( )

A. Generally, the early contact area in the center is ground first, and the functional cusp is not easily polished

B. The grinding of the side should take into account both the working side and the non-working side

C. The grinding work should be completed at one time to reduce the number of patient visits

D. At the end of grinding, the tooth surface must be polished with a hard rubber wheel to reduce the accumulation of plaque

E. For those who have allergic symptoms due to the exposure of dentin by grinding, desensitization treatment should be performed

7.2009U2A1060 Treatment that can eliminate periodontal pocket inflammation and increase the level of attachment ( )

A. Oral health education

B. Supragingival cleansing

C. Cleansing and root leveling

D. Periodontal pocket medication

Periodontal irrigation

8.2009U2A1061 Plaque control index, if it can be achieved, it is basically controlled ( )

A. Less than 10 per cent

B. Less than 15 per cent

C. Less than 20 per cent

D. Less than 25 per cent

E. Less than 30 per cent

9.2009U2A1066 Which is correct for the description of the grinding method ( )

A. Ground the median early contact point first

B. Don't change too much at once

C. Loose teeth with inflammation at the root tip should be reground after the inflammation is eliminated

D. Restore the spherical shape of the tooth as much as possible

E. All of the above

10.2009U2A1077 What factors should be considered first in the etiology of periodontitis to guide treatment ( )

A. Genetic factors

B. Endocrine factors

C. Immunological factors

D. Plaque factor

E. Nutritional factors

11.2016U2A1005 When there is early contact between median occlusion and non-median occlusion, it should be abrasional ( )

A. Bevel of the buccal tip of maxillary molars

B. Early contact area of the lingual fossa or fossa

C. Early contact cusps

D. Incisal margin of the inferior anterior teeth of early contact

E. C+D

12.2016U2A1009 Which of the following brushing methods is most effective in removing plaque near the gingival sulcus and in the adjacent space ( )

A. Light brushing

B. Horizontal fibrillation method

C. Vertical rotation method

D. Re-brushing method

E. Horizontal brushing method

13.2009U2A1079 Temporary splint fixation precautions are wrong ( )

A. The original position of the teeth should be maintained without traction

B. Suitable for those with intact dentition

C. After ligation and fixation, adequate occlusion should be performed with or without occlusal trauma

D. Strengthen oral hygiene guidance and strictly control bacteria

E. No need to fix your teeth to bite hard objects

14.2016U2A1014 Commonly used plaque display agent ( )

A. Magenta solution

B. Gentian violet liquid

C. Iodized oil

D. Methylene blue

E. Sodium fluorescein

15.2016U2A1016 Periodontal system treatment is ( )

A. Adjust the occlusion

B. Upper and lower gingival cleansing

C. Root surface leveling

D. Plaque control

E. Administered therapy

16.2008U2A2078 Anterior teeth have early contact in median occlusion, anterior occlusion coordination, should be abrasional modification ( )

A. Upper cusps

B. Lower cusps

C. Upper lingual fossa

D. Labial surface of the lower teeth

E. Upper tongue bevel

17.2008U2A1026 The gargle that can color the mucous membrane is ( )

A. Dobell′s

B. 1％NaHCO3

C. Saline

D. Chlorhexidine

E. 3% hydrogen peroxide (diluted 2-fold)

18.2004U2A1062 When cleaning with hand instruments, attention should be paid to ( )

A. The suitability of the instrument to the tooth surface and soft tissue

B. The working angle between the working end and the tooth surface

C. Use the push and pull of the wrist

D. The direction of cleaning is vertical, horizontal, and oblique

E. All of the above

19.2004U2A1070 Commonly used plaque display agent ( )

A. Basic magenta

B. Gentian violet

C. Red mercury

D. Congo red

E. Iodine tincture

1. E。 Cause: The goal of root planing is to remove infected cementum, residual tartar, granulation tissue from the inside of the pouch, and remove plaque from the root surface, so all of these options are included.
2. D。 Causes: Root desensitization is usually not required immediately after cleansing because it is not part of the standard procedure.
3. C。 Causes: The basic treatment of periodontal disease mainly focuses on plaque control, as this is the main cause of the onset and progression of periodontal disease.
4. E。 Reason: The selective grinding method is suitable for some vertical and horizontal types of food impaction, because not all food impaction needs to be treated by the selective grinding method.
5. D。 Reason: Cleansing is a process performed by professionals and is not a self-controlled plaque method.
6. C。 Reason: Grinding should not be done in one go, as excessive grinding can lead to tooth sensitivity or other problems.
7. C。 Causes: Scalculation and root planing are effective in eliminating periodontal pocket inflammation and increasing the level of attachment.
8. B。 Reason: Generally, if the plaque control index reaches less than 15%, it is considered to have been basically controlled.
9. E。 Why: All of these descriptions correctly state the principles of the grinding method.
10. D。 Causes: Plaque should be considered first in the treatment of periodontitis, as this is the most important causative factor.
11. E。 Causes: When there is early contact between median and nonmedian occlusions, the areas that should be abrasive may include the cusps and the incisal margin of the inferior anterior teeth.
12. C。 Cause: Vertical rotation (also known as Belleville brushing) is most effective at removing plaque near and adjacent spaces of the gingival sulcus.
13. D。 Causes: Oral hygiene instruction should be reinforced with temporary splinting, but adjustment of the occlusion is not required unless occlusal trauma is present.
14. E。 Why: Sodium fluorescein is a commonly used plaque display agent that can help visualize plaque.
15. D。 Why: Plaque control is a constant part of periodontal system treatment, as it is the basis for the prevention and treatment of periodontal disease.
16. E。 Causes: When there is early contact between the anterior teeth in the median occlusion, the lingual bevel of the upper teeth should be ground to eliminate the early contact.
17. D。 Causes: Chlorhexidine contains ingredients that may cause mucosal discoloration.
18. E。 Why: All of these aspects should be taken into account when using handmade instruments to ensure the effectiveness and safety of the treatment.
19. B。 Why: Gentian violet is a commonly used plaque display agent that can help identify plaque in the mouth.

**Chapter 17 Surgical Treatment of Periodontal Disease**

1.2009U2A1062 It is strictly forbidden to touch the tooth surface and root surface when using electric knife to cut the gingiva

A. Can cause tissue loss

B. Can cause osteonecrosis

C. Can cause soft tissue necrosis

D. Can irritate the pulp and cause pulp lesions

E. Can cause alveolar osteonecrosis

2. The concentration of citrate used for root treatment in 2009U2A1072 GTR surgery is ( )

A. 10% saturated citrate

B. 20% saturated citrate

C. 30% saturated citrate

D. 50% saturated citrate

E. 70% saturated citrate

3.2016U2A1004 Which of the following is not an absorbable GTR biocompatible membrane material ( )

A. Polymerized collagen membrane

B. PTFE

C. Polylactic acid film

D. Lactic acid-acetic acid copolymer membrane

E. All of the above

4.2016U2A1024 The best site for vertical incision in periodontal surgery should be selected in ( )

A. Midpoint of buccal/lingual gingiva adjacent to a healthy tooth

B. Near and distal axial angles of adjacent healthy teeth

C. Adjacent to the gingival papilla of a healthy tooth

D. The most prominent root of the adjacent healthy tooth

E. Bone margins in the lesion area

5.2008U2A1003 The main effect of the plugging agent does not include ( )

A. Protective

B. Bactericidal effect

C. Haemostasis

D. Analgesic

E. Immobilization

6.2000U2A1009 The incision that can completely remove the epithelium and infected tissue on the inner wall of the bag during periodontal flap surgery is ( )

A. Interdental incisions

B. Intrasulcus incision

C. External bevel incision

D. Internal bevel incision

E. Longitudinal incision

7.2004U2A1058 Purpose of pre-gingivectomy bag ( )

A. Determine the direction of the incision

B. Understand periodontal pocket depth

C. Know the gum thickness

D. Determine the incision location

E. All of the above

8.2004U2A1061 The ideal way for tissue to heal after periodontal surgery is ( )

A. New Attachment

B. Re-attachment

C. Long epithelial union

D. General Repairs

E. Bony union

9.2004U2A1065 Bone resorption of distal buccal root of maxillary first molar is more than 2/3, bone resorption of mesial buccal root and palatal root is about 1/3, distal and buccal root bifurcation lesion III. degree, selectable ( )

A. Amputation of the meso-buccal root

B. Truncation of the distal buccal root

C. Hemitomy

D. Guided tissue regeneration

E. Alveolar osteoplasty

10.2004U2A1067 During the healing process of periodontal surgery, the cells that preferentially occupy the root surface are usually ( )

A. Alveolar bone marrow cells

B. Gingival connective tissue cells

C. Gingival epithelial cells

D. Periodontal ligament cells

E. Odontoblasts

11.2004U2A1069 The advantages of internal bevel incision are ( )

A. Direct excision of the epithelial and inflammatory tissue on the inner wall of the bag

B. Preservation of attached gingiva on the surface of the periodontal pocket

C. Less postoperative gingival recession

D. The gingival shape is better after healing

E. All of the above

12.2013U2A1010 During periodontal surgery, if the buccal and lingual gingival tissues are of equal height and tension, and the suture needle can pass through both gingival flaps at the same time, the method used is ( )

A. Interdental intermittent sutures

B. Interdental "figure 8" suture

C. Suspension suture

D. Horizontal mattress suture

E. Anchor suture

13. 2012U2A1009 Indications for guided tissue regeneration, except ( )

A. II. Root bifurcation lesions

B. IV. Root bifurcation lesions

C. Hypopalasal pouch

D. Three-wall sub-bone pocket

E. Angular bone resorption

14.2012U2A1011 Which of the following should not be treated for gingivectomy ( )

A. Hyperplasia of gingival tissue

B. Chronic abscess in suprabony pockets

C. Posterior teeth attach to moderately deep supraosseous pockets where the gingiva is too narrow

D. Wisdom tooth pericrown blind pocket formation

E. Gingival tumors

1. C. Can cause soft tissue necrosis. High temperatures when using an electric knife to cut the gums can lead to thermal necrosis of soft tissue cells.
2. B. 20% saturated citrate. The root surface is usually treated with 20% saturated citric acid to remove tartar and contaminants.
3. B. PTFE. PTFE is a non-absorbent material and is not part of the category of absorbable GTR membranes.
4. B. Near and distal axial angles of adjacent healthy teeth. This position reduces postoperative incision tension and promotes healing.
5. D. Analgesic. Stoppers are mainly used for protection, sterilization, hemostasis and fixation, and are not directly used for pain relief.
6. C. External bevel incision. The external oblique incision allows complete excision of the epithelium and infected tissue on the inner wall of the bag.
7. E. All of the above. The pouch is fixed to help determine the direction, location, and depth of the incision, as well as the thickness of the gums.
8. B. Re-attachment. The ideal way for tissue to heal after periodontal surgery is to return the periodontal tissue to normal by reattaching.
9. D. Guided tissue regeneration. Given the degree of bone resorption and the extent of the lesion, guided tissue regeneration is the most appropriate option.
10. C. Gingival epithelial cells. During the healing process, gingival epithelial cells usually preferentially occupy the root surface because of their rapid proliferation.
11. E. All of the above. The advantages of the internal oblique incision include direct excision of the inflammatory tissue, preservation of the attached gingiva, less postoperative gingival recession, and better appearance.
12. B. Interdental "figure 8" suture. When the gingival tissue is of equal height and the tension is consistent, the figure-8 suture can effectively fix the gingival flap.
13. B. IV. Root bifurcation lesions. IV. The severity of the root bifurcation lesion is usually not amenable to guided tissue regeneration.
14. D. Wisdom tooth pericrown blind pocket formation. Due to the anatomical characteristics of the wisdom tooth area and the particularity of the blind pocket, the wisdom tooth pericrown blind bag is not suitable for gingivectomy.

**Oral mucosal diseases**

**Infectious diseases of the oral mucosa**

1. 2001U1A1014 Acyclovir antiviral ability in the correct order is ( )

A. HSV-2, HSV-1, varicella-zoster virus, Epstein-Barr virus

B. HSV-1, HSV-2, varicella-zoster virus, Epstein-Barr virus

C. HSV-1, HSV-2, Epstein-Barr virus, varicella-zoster virus

D. HSV-2, HSV-1, Epstein-Barr virus, varicella-zoster virus

E. HSV-1, Epstein-Barr virus, HSV-2, varicella-zoster virus

2. 2010U2A2104 female, 50 years old, white patches of buccal mucosa near the corners of the mouth for nearly l years, can not be wiped off. Histology shows epithelial hyperplasia with neutrophil infiltration and scattered microabscesses, PAS-positive hyphae perpendicular to the epithelium in the keratotic layer, and chronic inflammatory cell infiltration in connective tissue. The most likely pathological diagnosis is ( )

A. Vitiligo

B. Erythema

C. Oral tuberculitis

D. Candidiasis

E. Chronic discoid lupus erythematosus

3.2009U2A1022 Typical clinical manifestations of herpes zoster in the face and mouth ( )

A. Clusters of erythema, blisters are symmetrically distributed along the trigeminal nerve and are painful

B. Clusters of erythema, blisters along one side of the trigeminal nerve, severe pain

C. Clusters of erythema and blisters are distributed along one side of the trigeminal nerve, and in severe cases, they can be distributed beyond the midline to the contralateral trigeminal nerve, and the pain is severe

D. Erythema appears on the face, along one side of the trigeminal nerve, followed by blisters, secondary infection and pustules, and finally crusting, leaving scars after scabbing off

E. None of the above

4.2001U3A1018 Oral tuberculosis anti-tuberculosis chemotherapy regimen medium and long-term (standard) chemotherapy drugs do not include ( )

A. Isoniazid

B. Streptomycin

C. Rifampicin

D. Ethambutol

E. p-Aminosalicylic acid

5. 2009U2A1027 is not an infectious disease ( )

A. Herpes zoster

B. Recurrent herpetic stomatitis

C. Oral tuberculosis

D. Snow mouth disease

E. Bullous pemphigoid

6.2009U2A1033 Herpes simplex virus can be transmitted by the following routes, except ( )

A. Skin

B. Cornea

C. Mucous membranes

D. Digestive tract

E. Respiratory

7.2009U2A1038 Candida angular cheilitis in elderly patients is more often related to which of the following ( )

A. Older people are susceptible to systemic diseases

B. Older people have lower resistance

C. Long-term use of various medications, especially antibiotics

D. The vertical distance of the occlusion is shortened, and saliva is easy to overflow from the corners of the mouth and is in a long-term wet state

E. None of the above

8.2009U2A1039 Primary herpetic gingivostomatitis has a course of ( )

A. 2~5 days

B. 4~6 days

C. 7~10 days

D. 9~11 days

E. 9~13 days

9.2000U2A1003 Oral mucosa and skin manifestations of dense herpes with unilateral distribution along the nerve are ( )

A. Recurrent herpetic stomatitis

B. Acute herpetic gingivostomatitis

C. Herpangina

D. Herpetiform aphthous ulcers

E. Shingles

10.2000U2A1004 Oral candidiasis often occurs in the following situations, except ( )

A. Long-term use of broad-spectrum antibiotics

B. Long-term use of immunosuppressants

C. Chronic wasting disease

D. Chronic stress

E. Candida albicans itself is more virulent

11.2000U2A1006 Herpetic gingivostomatitis is more common in ( )

A. Infants and toddlers aged 6 months ~ 2 years old

B. Preschoolers

C. Adolescents

D. Middle-aged

E. Older persons

12.2000U2AN122 female, 2.5 years old. High fever for 3 days, canker sores for 2 days. Crying, salivating, refusing to eat. Physical examination found that the child's gingiva was red and swollen all over the mouth, and dense needle-sized transparent blisters could be seen on the palate mucosa, some of which had broken into superficial ulcers, and the surrounding mucosa was congested and edema was extensive. The most likely diagnosis in this case is ( )

A. Thrush

B. Foot-and-mouth disease

C. Mouth sores in Belleson

D. Stomatitic aphthous ulcers

E. Herpetic gingivostomatitis

13.2000U2AN123 female, 2.5 years old. High fever for 3 days, canker sores for 2 days. Crying, salivating, refusing to eat. Physical examination found that the child's gingiva was red and swollen all over the mouth, and dense needle-sized transparent blisters could be seen on the palate mucosa, some of which had broken into superficial ulcers, and the surrounding mucosa was congested and edema was extensive. The most likely diagnosis in this case is herpetic gingivostomatitis, and depending on the clinical presentation of the disease, the preferred ancillary tests are ( )

A. Urinalysis

B. Routine blood test and differential examination

C. Biopsy

D. T cell subset assay

E. Liver and kidney function

14.2000U2AN124 female, 2.5 years old. High fever for 3 days, canker sores for 2 days. Crying, salivating, refusing to eat. Physical examination found that the child's gingiva was red and swollen all over the mouth, and dense needle-sized transparent blisters could be seen on the palate mucosa, some of which had broken into superficial ulcers, and the surrounding mucosa was congested and edema was extensive. The most likely diagnosis of this case is herpetic gingivostomatitis, and the measures that are not suitable for treatment in this case are ( )

A. Take breaks to strengthen your nutrition

B. Systemic use of antiviral drugs

C. Nebulized inhalation with topical corticosteroids

D. Supplement with plenty of vitamins

E. Traditional Chinese Medicine

15. 2012U2A1001 Which of the following diseases generally does not leave scars after healing ( )

A. Periglandular aphthous ulcers

B. Herpes zoster

C. Fibrosis of the oral mucosa

D. Genital ulcers in Behcet's disease

E. Pressure ulcers

16.2012U2A1002 The following statements about candidal cheilitis are incorrect ( )

A. Candidal angular cheilitis is usually bilateral

B. Characterized by wet white erosions

C. Angular cheilitis in older patients is often associated with shortened vertical occlusal distance

D. It often occurs in children during the cold winter months

E. Often complicated by glossitis, cheilitis, scrotitis

17.2004U2A1019 Regarding varicella-zoster virus, the following correct statement is ( )

A. Attack T lymphocytes

B. Violation of the trigeminal nerve

C. Causes submucosal fibrosis

D. Low serum antibody titers after infection

E. Increase the chance of infection with other viruses

18.2004U2A1027 The following diseases can cause papillae atrophy, except ( )

A. Lichen planus

B. Sjögren's syndrome

C. Discoid lupus erythematosus

D. B vitamin deficiency

E. Oral Candida infection

19.2004U2A1037 The clinical features of chronic proliferative candidiasis are ( )

A. White papules with cross-reticular streaks on the oral mucosa, and hyperemia of the basal mucosa

B. White curd-like spots or patches on the oral mucosa without obvious symptoms

C. Well-defined erythema can be seen in the oral mucosa, and tongue papillae atrophy with erosion and chapping of the mucosa of the oral corners

D. Dense white plaques appear on the mucosa on the medial side of the mouth corners, with a rough surface, accompanied by bilateral redness and chapping of the corners of the mouth

E. A diamond-shaped, filamentous papillary atrophy zone appears in the posterior central mucosa of the dorsal tongue, and nodular hyperplasia may appear on the surface, which is hard on examination but soft at the base

20.2010U2A1012 Varicella-zoster virus can cause in adults and the elderly ( )

A. Chickenpox

B. Hand-foot-mouth disease

C. Herpangina

D. Herpes zoster

E. A+D

21.2010U2A1016 ( )

A. Lupus vulgaris

B. Snow mouth sores

C. Herpes zoster

D. Pemphigus vulgaris

E. Hand-foot-mouth disease

22.2010U2A1017 Antivirals that are highly selective against herpes simplex virus types I and II are ( )

A. Ribavirin

B. Interferon

C. Polymyocytes

D. Acyclovir

E. Thymosin

23.2010U2A1023 An important item used to distinguish between herpetiform aphthous ulcers and herpetic stomatitis is ( )

A. Good or bad prognosis

B. Lesion size

C. Pain level

D. Presence or absence of skin lesions

E. Acupuncture reaction

24.2010U2A1024 Which of the following drugs should not be used in patients with herpes zoster with serious complications ( )

A. Acyclovir

B. Interferon

C. Prednisone

D. Cimetidine

E. Transfer factors

25. The common sites of recurrent infection with herpes simplex virus type I in 2010 are ( )

A. Buccal mucosa

B. Mucosa dorsal of the tongue

C. Mucosa of the tongue and lateral border of the tongue

D. Labial mucosa

E. Lip redness and peripheral skin

26.2012U2A2102 Female, 40 years old, erythema around the lips 1 week ago, burning itching, clusters of needle-tip blisters appeared one day later, scabbed after the blisters broke and erosion one day later, there was a history of cold before the illness, probable diagnosis ( )

A. Angular cheilitis

B. Discoid lupus erythematosus

C. Cold sores

D. lichen planus

E. Thrush

27.2010U2A1028 Membranous stomatitis is caused by ( )

A. Fusophilia

B. Caused by mycobacteria

C. Coxsackievirus M

D. Herpes simplex virus type I

E. Staphylococcus aureus and hemolytic streptococcus

28.2010U2A1029 Oral herpes simplex virus infection route ( )

A. Droplets

B. Saliva

C. Herpes fluid

D. Birth canal

E. All of the above

29.2010U2A1030 The cause of primary herpetic stomatitis is infection ( )

A. HSVI

B. HSVⅡ

C. HIVI

D. HIVⅡ

E. None of the above

30.2002U2A1012 Herpetic recurrent aphthous ulcer is characterized by ( )

A. Generally, there are 1~5 ulcers with a diameter of 5~10mm, which mostly occur in the lip and cheek mucosa

B. It is mostly a single large ulcer, with a diameter of more than 1 cm, and mostly occurs in the cheeks, soft palate, etc

C. The number and diameter vary greatly, and obvious local stimuli can be seen

D. The ulcer is single, long-lasting, and excavating

E. Multiple ulcers can reach dozens, with a diameter of 1~2mm, and can also be fused into patches, mostly seen in the mucosa of the floor of the mouth and tongue and abdomen

31.2002U2A1013 The incidence of neonatal snow mouth disease is ( )

A. 0.02

B. 0.03

C. 0.04

D. 0.05

E. 0.06

32.2002U2A1014 Among the following infectious oral mucosal diseases, the clinical characteristics of pseudomembrane lesions are ( )

A. Herpes simplex

B. Herpes zoster

C. Hand-foot-mouth disease

D. Coccal stomatitis

E. Oral tuberculosis

33.2002U2A1017 The following ulcers will leave scars after healing ( )

Aphthous ulcers

B. Severe aphthous ulcers

C. Herpetiform aphthous ulcer

D. Behcet's disease canker sores

E. All of the above

34.2012U2A2103 A 40-year-old female who had clusters of pinhead-sized blisters on her lower lip and around her lips after a cold, crusted over after bursting, and was locally itching and painful. The patient's possible illness is ( )

A. Cold sores

B. Chronic cheilitis

C. Herpetic stomatitis

D. Fixed drug eruption

E. Recurrent aphthous ulcers

35.2012U2A2104 Xie, female, 34 years old, 2 days after fever, felt itching and burning pain in the left side of the maxillary gum, and soon found that the left maxillary gum was red and swollen, and clusters of transparent blisters the size of millet grains appeared on the surface, which became small ulcers after bursting. Which of the following treatments is the most important ( )

A. Keep your mouth clean

B. Intramuscular injection of penicillin

C. Oral antipyretic analgesics

D. Oral administration of acyclic guanosine or radix of banyan root

E. Bed rest and multivitamin supplementation

36.2012U2A2105 Baby male, 5 days old, crying and refusing to eat, the oral mucosa appears slightly convex soft white dots, wiped off to reveal the bleeding surface, probable diagnosis ( )

A. Early eruption of baby teeth

B. Vitiligo

C. Infantile snow mouth disease

D. Keratinized epithelial beads

E. None of the above

37.2012U2A2106 Infant, female, 7 days old, crying and refusing to eat. Slightly convex soft white dots appear in the oral mucosa, which can reveal bleeding spots after wiping, and the probable diagnosis is ( )

A. Vitiligo

B. Early deciduous teeth

C. Candidiasis albicans

D. Keratinized epithelial beads

E. Herpetic stomatitis

38.2013U2A2079 Baby girl, 7 days old. The mother complained of irritability, crying, and refusal to eat. Oral examination showed scattered snow-white soft spots on the left cheek and tip of the tongue, and obvious basal mucosal hyperemia. With a little force, it can be wiped off, leaving a bleeding erosion. Initial diagnosis is ( )

A. Acute pseudomembranous candidal stomatitis

B. Acute erythematous candidal stomatitis

C. Chronic hypertrophic candidal stomatitis

D. Candida chronic erythematous stomatitis

E. Candidal cheilitis

39.2013U2A2080 Infant girl, 2 months old, white curd-like spots and plaques on the oral mucosa that can be wiped, redness, crying, and difficulty in breastfeeding of the basal mucosa, should be suspected as ( )

A. Thrush

B. Recurrent canker sores

C. Herpetic gingivostomatitis

D. Coccal stomatitis

E. Crohn disease

40.2013U2A2081 Male, 60 years old, suffering from diabetes for 10 years. In the past 2 weeks, the mouth has been dry, the thirst has worsened, the corners of the mouth have been cracked, bleeding, and there is a significant burning sensation when eating. Bilateral redness of the corners of the mouth with fissure, mandibular and mandibular restoration, and redness of the mucosa in the denture support area, which is consistent with the denture. A large number of hyphae can be seen on the surface smear of the denture. Which method should be selected for clinical treatment is the most effective ( )

A. Gargle with 3%~5% sodium bicarbonate solution every day

B. Maintain oral hygiene and pay attention to the isolation and disinfection of tableware

C. Replacement or relining of dentures

D. Topical application of nystatin or dacnine cream in the mouth

E. Supplement with iron, vitamin B12 and folic acid

41.2013U2A2082 Male, 10 years old. The left tongue margin ulcerated for more than 3 months. The earliest ulceration was a small nodule that was painless, and the left submandibular lymph node was painful. Oral examination: 1 ulcer with a diameter of 1 cm can be seen on the left tongue margin, there is induration around it, there is a little purulent discharge at the base, dark red mulberry-like granuloma can be seen in the removal of exudate, and the edge of the ulcer is rat-like.

A. Tuberculin test

B. Erythrocyte sedimentation rate

C. Concentrated culture of collected bacteria

D. Biopsy histopathological examination

E. All of the above

42.2013U2A2083 Male, 35 years old. Pain in tongue ulceration for 1 week. 10 days ago, he had a "cold and fever" accompanied by multiple erosions in the mouth. Oral examination: an erosion surface with a diameter of 2cm can be seen in the center of the dorsum of the tongue, with obvious hyperemia and edema, a yellow-brown pseudomembrane on the surface, which is easy to wipe off, leaving a bloody erosion surface, obvious pain, and non-specific halitosis. Probable( )

A. Oral lichen planus

B. Acute pseudomembranous candidal stomatitis

C. Recurrent herpetic stomatitis

D. Recurrent severe aphthous ulcers

E. Coccal stomatitis

43.2013U2A2084 A 22-year-old female presents with swollen and painful gums with spontaneous bleeding. The patient was examined for poor oral hygiene, scaltar grade III, and obvious halitosis. The gingiva of the whole mouth is red and swollen, the gingival papillae on the left and right lower 12 lips are knife-shaped, the surface is covered with gray-white pseudo-membrane, the necrotic membrane is removed, and the base is a fresh wound that is easy to bleed. The case is intended to be diagnosed as ( )

A. Acute infectious stomatitis

B. Radiation stomatitis

C. Necrotizing gingivostomatitis

D. Herpetic gingivostomatitis

E. Leukemia

44.2013U2A2085 An 8-year-old male presented with 1 week of necrosis and ulceration of the gums and oral mucosa with high fever for 10 days. The initial diagnosis was necrotizing gingivostomatitis. Which of the following drugs is not suitable for topical oral treatment ( )

A. 3% hydrogen peroxide

B. 1% gentian violet

C. 10% silver nitrate

D. Periodontal plug preparations

E. Nourishing Yin and Shengjisan or Ice Boron

45.2010U2A2104 Female, 50 years old, white patches of buccal mucosa near the corners of the mouth for nearly l years, which cannot be erased. Histology shows epithelial hyperplasia with neutrophil infiltration and scattered microabscesses, PAS-positive hyphae perpendicular to the epithelium in the keratotic layer, and chronic inflammatory cell infiltration in connective tissue. The most likely pathological diagnosis is ( )

A. Vitiligo

B. Erythema

C. Oral tuberculitis

D. Candidiasis

E. Chronic discoid lupus erythematosus

46.2005U2A1011 The cause of herpetic gingivostomatitis is ( )

A. Bacteria

B. Viruses

C. Fungi

D. Chlamydia

E. Rickettsia

47.2005U2A1012 The cause of gangrenous stomatitis may be ( )

A. Poor oral hygiene

B. Vitamin deficiencies

C. Local trauma

D. Viral infection

E. Bacterial infections

48.2004U2A1038 Recurrent herpetic stomatitis is generally common ( )

A. Lips

B. Buccal mucosa

C. Back of the tongue

D. Gingiva

E. Palatal mucosa

49.2004U2A1039 Oral tuberculosis pathogenic microorganisms are ( )

A. Viruses

B. Bacteria

C. Fungi

D. Chlamydia

E. Mycoplasma

50.2004U2A1020 The preferred drug for gangrenous stomatitis is ( )

A. Penicillin

B. Acyclic guanosine

C. Corticosteroids

D. nystatin

E. Postsamycin plus metronidazole

51.2002U2A1022 Systemic drug of choice for the treatment of acute herpetic gingivostomatitis ( )

A. Stomatitis powder

B. Acyclic guanosine

C. B vitamins

D. Corticosteroids

E. Broad-spectrum antibiotics

52.1999U2AN128 A 45-year-old female presents with redness and blistering of the skin on the left cheek and mucosa on the left tongue for 3 days, accompanied by severe pain. On physical examination, the body temperature was 38.5°C, dense patches of miliary transparent blisters were seen on the left facial skin and the left dorsum of the tongue and buccal mucosa, and congestive erythema was seen on the surrounding skin and mucosa. Assays: WBC 7.8×19/L, neutral 62%, lymphatic 34%. Diagnosis of herpes zoster. The main lesion sites in this case are ( )

A. Trigeminal nerve 1

B. Trigeminal nerve 2nd branch

C. 3rd branch of the trigeminal nerve

D. Facial nerve

E. Cervical nerve

53.1999U2AN129 A 45-year-old female presents with redness and blistering of the skin on the left cheek and mucosa on the left tongue for 3 days, accompanied by severe pain. On physical examination, the body temperature was 38.5°C, dense patches of miliary transparent blisters were seen on the left facial skin and the left dorsum of the tongue and buccal mucosa, and congestive erythema was seen on the surrounding skin and mucosa. Assays: WBC 7.8×19/L, neutral 62%, lymphatic 34%. Diagnosis of herpes zoster. The main differentiators between herpes zoster and herpes simplex are ( )

A. The etiology is different

B. Different prodromal symptoms

C. The duration of the disease varies

D. Clinical manifestations are different

E. Systemic symptoms vary

54.1999U2AN130 A 45-year-old female presents with redness and blistering of the skin on the left cheek and mucosa on the left tongue for 3 days, accompanied by severe pain. On physical examination, the body temperature was 38.5°C, dense patches of miliary transparent blisters were seen on the left facial skin and the left dorsum of the tongue and buccal mucosa, and congestive erythema was seen on the surrounding skin and mucosa. Assays: WBC 7.8×19/L, neutral 62%, lymphatic 34%. Diagnosis of herpes zoster. The most serious complications that can occur in this case are ( )

A. Pneumonia

B. Encephalitis

C. Conjunctivitis

D. Keratitis

E. Facial paralysis

55.1999U2AN132 A 45-year-old female presents with redness and blistering of the skin on the left cheek and mucosa on the left tongue for 3 days, accompanied by severe pain. On physical examination, the body temperature was 38.5°C, dense patches of miliary transparent blisters were seen on the left facial skin and the left dorsum of the tongue and buccal mucosa, and congestive erythema was seen on the surrounding skin and mucosa. Assays: WBC 7.8×19/L, neutral 62%, lymphatic 34%. Diagnosis of herpes zoster. Which of the various treatment measures you think is unreasonable at this point ( )

A. Oral antiviral drugs such as acyclic guanosine, etc

B. Injection of polymyocytes

C. Vitamin B₁₂ 500μg + vitamin B₁ 100mg intramuscularly once every other day

D. Skin lesions are coated with calamine lotion

E. Oral partial closure of dexamethasone + procaine

56.1999U2AN131 A 45-year-old female presents with redness and blistering of the skin on the left cheek and mucosa on the left tongue for 3 days, accompanied by severe pain. On physical examination, the body temperature was 38.5°C, dense patches of miliary transparent blisters were seen on the left facial skin and the left dorsum of the tongue and buccal mucosa, and congestive erythema was seen on the surrounding skin and mucosa. Assays: WBC 7.8×19/L, neutral 62%, lymphatic 34%. Diagnosis of herpes zoster. The patient was given intramuscular penicillin injection for 3 days, laser irradiation of local lesions and oral multivitamins and other measures in the hospital, and the symptoms improved, but did not disappear completely, especially the pain symptoms were still obvious. The reasons for poor efficacy are ( )

A. No supportive care was given

B. No injection of polymyocytes or transfer factors

C. Incorrect diagnosis

D. Topical anti-inflammatory gargle is not used

E. Carbamazepine or intramuscular vitamin B₁+ vitamin B₁₂ was not given

57.2004U2A1048 Herpes simplex virus susceptible sites do not include ( )

A. Oral cavity

B. Skin

C. Eye

D. Nervous system

E. Digestive tract

58.2016U2A1060 Diseases characterized by secondary ulcers of the oral mucosa are ( )

A. Chronic cheilitis

B. Vitamin A deficiency

C. Angular cheilitis

D. Herpes simplex

E. Map tongue

59.2017U2A1055 Herpes simplex virus nucleocapsid is made up of ( )

A. Composition of 160 shell particles

B. Composition of 161 shell particles

C. Composition of 162 shell particles

D. Composition of 163 shell particles

E. Composition of 164 shell particles

60.2017U2A1054 The following is wrong about the mode of transmission of oral herpes simplex virus ( )

A. Respiratory

B. Oral cavity

C. Nose

D. Conjunctiva of the eye

E. Digestive tract

61.2017U2A1053 The clinical manifestations of acute erythematous candida stomatitis are ( )

A. The mucosa of the lesion is congested with small soft spots as white as snow

B. The mucosa of the lesion appears as nodular or granular hyperplasia, or as a tightly fixed white keratin plaque

C. The mucosa in the lesion area is bright red edema, or a mottled pseudomembrane is seen

D. The mucosa in the lesion area is congested and erosive, the papillae on the back of the tongue are clumps and atrophied, and the surrounding tongue coating is thickened

E. Pearlescent white reticulation with hyperemia on the mucosa of the lesion

62.2017U2A1052 For elderly patients with chronic hypertrophic type in candidal stomatitis, the preferred diagnostic method is ( )

A. Saliva culture

B. Saliva and serum Candida antibody determination

C. Biopsy

D. Direct smear microscopy

E. Blood CEA assay

63.2020U1A1006 Typical clinical manifestations of acute pseudomembranous oral candidiasis are ( )

A. The dorsal mucosa of the tongue is bright red with tongue papilla atrophy

B. The mucosa in the denture support area is bright red

C. Medial triangle of the mouth with granular hyperplasia

D. The oral mucosa is congested with curd-like patches

E. The mucosa in the corner of the mouth is wet and white, erosive, and cracked

64.2020U1A1007 The typical clinical manifestations of chronic proliferative oral candidiasis are ( )

A. The dorsal mucosa of the tongue is bright red with tongue papilla atrophy

B. The mucosa in the denture support area is bright red

C. Congestion of the angular symphysis with granular hyperplasia

D. The oral mucosa is congested with curd-like patches

E. The mucosa in the corner of the mouth is wet and white, erosive, and cracked

GPT-4

1. B. HSV-1, HSV-2, varicella-zoster virus, Epstein-Barr virus. Causes: Acyclovir has the strongest antiviral effect against HSV-1, followed by HSV-2, then varicella-zoster virus, and the weakest effect against Epstein-Barr virus.
2. D. Candidiasis. Causes: White patches, PAS-positive hyphae in the keratotic layer, and microabscesses are typical histologic features of candidiasis.
3. B. Clusters of erythema, blisters along one side of the trigeminal nerve, severe pain. Causes: Herpes zoster typically presents with unilateral painful erythema and blisters along the nerves.
4. B. Streptomycin. Causes: Streptomycin is not a long-term (standard) chemotherapy drug commonly used in antituberculosis treatments.
5. E. Bullous pemphigoid. Causes: Bullous pemphigoid is an immune-mediated skin disease that is not caused by an infection.
6. E. Respiratory. Causes: The herpes simplex virus is usually transmitted through contact with the skin and mucous membranes, not through the respiratory tract.
7. C. Long-term use of various medications, especially antibiotics. Causes: Long-term antibiotic use in older adults can lead to an imbalance in the oral flora and increase the risk of Candida infection.
8. B. 4~6 days. Causes: The course of primary herpetic gingivostomatitis is usually 4 to 6 days.
9. E. Shingles. Causes: Herpes zoster is characterized by dense herpes that is distributed along the nerves and usually appears unilaterally.
10. E. Candida albicans itself is more virulent. Causes: Oral candidiasis is common in people with weakened immunity or long-term use of antibiotics or immunosuppressants, not because Candida albicans itself is more virulent.
11. B. Preschoolers. Causes: Herpetic gingivostomatitis is usually more common in preschool children.
12. E. Herpetic gingivostomatitis. Causes: High fever, mouth ulcers, and pin-sized clear blisters are typical of gingivostomatitis.
13. B. Routine blood test and differential examination. Causes: The diagnosis of gingivostomatitis herpetic can be confirmed by routine blood tests and differential examinations, and changes in white blood cell count can be observed.
14. C. Nebulized inhalation with topical corticosteroids. Causes: For gingivostomatitis herpetic, topical corticosteroids may exacerbate the infection and are therefore not appropriate.
15. A. Periglandular aphthous ulcers. Causes: Periglandular aphthous sores usually heal without scarring.
16. D. It often occurs in children during the cold winter months. Causes: Candidal angular cheilitis is not a seasonal condition, and children are not particularly susceptible.
17. B. Violation of the trigeminal nerve. Causes: The varicella-zoster virus specifically invades the nervous system, including the trigeminal nerve.
18. E. Oral Candida infection. Causes: Oral candida infections usually cause white plaques to appear on the oral mucosa and do not directly cause tongue papillae to atrophy.
19. D. Dense white plaques appear on the mucosa on the medial side of the mouth corners, with a rough surface, accompanied by bilateral redness and chapping of the corners of the mouth. Causes: Chronic proliferative candidiasis manifests as angular cheilitis, dense white patches on the oral mucosa, and other symptoms.
20. E. A+D。 Causes: The varicella-zoster virus can cause chickenpox in children and shingles in adults and older adults.
21. A. Lupus vulgaris. Causes: Lupus vulgaris is an autoimmune disease, not an infectious disease.
22. D. Acyclovir. Causes: Acyclovir is highly selective for herpes simplex virus types I and II.
23. D. Presence or absence of skin lesions. Causes: An important difference between herpetiform aphthous sores and herpetic stomatitis is the presence or absence of skin lesions.
24. C. Prednisone. Causes: Prednisone is a glucocorticoid that can be aggravated in people with shingles who have serious complications.
25. D. Labial mucosa. Causes: Recurrent HSV type 1 infection is common in the lips, particularly the labial mucosa.
26. C. Cold sores. Causes: Fever after a cold and clusters of blisters on the lips are typical symptoms of cold sores.
27. D. Herpes simplex virus type I. Causes: Membranous stomatitis is usually caused by herpes simplex virus type I.
28. E. All of the above. Causes: The oral herpes simplex virus can be transmitted through a variety of routes, including droplets, saliva, herpes fluid, and the birth canal.
29. A. HSVI。 Causes: Primary herpetic stomatitis is usually caused by HSVI (herpes simplex virus type I).
30. E. Multiple ulcers can reach dozens, with a diameter of 1~2mm, and can also be fused into patches, mostly seen in the mucosa of the floor of the mouth and tongue and abdomen. Causes: Herpetic recurrent aphthous ulcers are characterized by small, multiple ulcers that coalesce into patches.
31. B. 0.03。 Causes: According to epidemiological data, the incidence of neonatal snow mouth disease is about 0.03.
32. D. Coccal stomatitis. Causes: Coccal stomatitis is typically characterized by the formation of a pseudomembrane.
33. D. Behcet's disease canker sores. Causes: Behcet's canker sores often leave scars after they heal.
34. A. Cold sores. Causes: Small blisters and crusts around the lips that appear after a cold, accompanied by burning pain, typical of cold sores.
35. A. Keep your mouth clean. Causes: Maintaining oral hygiene is one of the most important treatments for oral diseases.
36. C. Infantile snow mouth disease. Causes: Slightly convex soft white dots appear on the oral mucosa, which bleed after wiping, which is consistent with the typical symptoms of snow mouth disease.
37. C. Candidiasis albicans. Causes: Slightly convex soft white dots appear in the mouth of newborns, bleeding spots can be seen after wiping, common in candidiasis albicans.
38. A. Acute pseudomembranous candidal stomatitis. Causes: The infant cries and refuses to eat, and the oral examination shows small snow-white soft spots, and the basal mucosa is hyperemic, which is consistent with the characteristics of acute pseudomembranous candida stomatitis.
39. A. Thrush. Causes: White curd-like spots on the oral mucosa can be wiped with redness of the base, commonly seen in thrush.
40. D. Topical application of nystatin or dacnine cream in the mouth. Causes: Symptoms of the patient and a large number of hyphae on the surface smear of the denture are consistent with Candida infection, so topical antifungal drugs are effective.
41. D. Biopsy histopathological examination. Causes: To confirm the diagnosis of ulcerative lesions, biopsy is required to observe the tissue structure and nature of the lesion.
42. B. Acute pseudomembranous candidal stomatitis. Cause: Oral pain, yellow-brown pseudomembrane easy to wipe off, consistent with the clinical manifestations of acute pseudomembrane candidal stomatitis.
43. C. Necrotizing gingivostomatitis. Cause: Red and swollen gingiva with grayish-white pseudomembrane and fresh bleeding wounds, consistent with necrotizing gingivostomatitis.
44. E. Nourishing Yin and Shengjisan or Ice Boron. Causes: Necrotizing gingivostomatitis requires anti-inflammatory and tissue repair, while Yangyin Shengjisan or Bingboron Powder is more suitable for treating dry or non-infectious diseases.
45. D. Candidiasis. Cause: Histologic features show PAS-positive hyphae, indicating Candida infection.
46. B. Viruses. Causes: Herpetic gingivostomatitis is mainly caused by viruses, particularly herpes simplex virus.
47. E. Bacterial infections. Causes: Gangrenous stomatitis is usually caused by a severe bacterial infection.
48. A. Lips. Causes: Recurrent herpetic stomatitis is often more common on the lips.
49. B. Bacteria. Causes: Oral tuberculitis is caused by Mycobacterium tuberculosis, which is a bacterium.
50. A. Penicillin. Causes: Gangrenous stomatitis is mainly caused by bacteria, and penicillin is an effective antibacterial drug.
51. D. Corticosteroids. Causes: Acute herpetic gingivostomatitis is often treated with corticosteroids to reduce inflammation and pain.
52. B. Trigeminal nerve 2nd branch. Causes: Shingles usually distributes along the nerves, and symptoms on the cheeks and tongue may indicate that the 2nd branch of the trigeminal nerve is affected.
53. D. Clinical manifestations are different. Causes: Herpes zoster is characterized by a banded herpes that runs along the nerves, whereas herpes simplex is more widespread and does not follow the nerves.
54. E. Facial paralysis. Causes: Shingles affecting the facial nerve can lead to facial paralysis, which is a serious complication.
55. E. Oral partial closure of dexamethasone + procaine. Causes: Topical oral treatment is not the standard treatment for shingles and may not be appropriate for this systemic disease caused by a virus.
56. C. Incorrect diagnosis. Causes: The patient's symptoms do not disappear completely, possibly because the initial diagnosis is inaccurate and the diagnosis needs to be reevaluated.
57. E. Digestive tract. Causes: Herpes simplex virus mainly invades the skin and mucous membranes and rarely the digestive tract.
58. D. Herpes simplex. Causes: Secondary ulcers of the oral mucosa are common in herpes simplex virus infection.
59. C. Composition of 162 shell particles. Causes: The nucleocapsid of the herpes simplex virus consists of 162 shell particles.
60. E. Digestive tract. Causes: The herpes simplex virus is mainly transmitted through the respiratory tract and contact, not the digestive tract.
61. C. The mucosa in the lesion area is bright red edema, or a mottled pseudomembrane is seen. Causes: Acute erythematous candida stomatitis presents with mucosal redness and swelling and pseudomembrane formation.
62. C. Biopsy. Causes: For chronic hypertrophic candidiasis, biopsy can help visualize histologic changes and confirm the diagnosis.
63. D. The oral mucosa is congested with curd-like patches. Causes: The typical clinical manifestations of acute pseudomembranous candidiasis are hyperemia and curd-like patches of the oral mucosa.
64. D. The oral mucosa is congested with curd-like patches. Causes: Chronic proliferative oral candidiasis usually presents with mucosal hyperemia and curdled patches.

**Chapter 3 Allergic Diseases of the Oral Mucosa**

1.2009U2A1028 Erythema multiforme complicated by skin lesions is characterized by ( )

A. Erosion

B. Ulcers

C. Exudation is excessive, forming a grayish-white pseudofilm

D. Significant pain

E. Erythema iridis

2. 2009U2A1036 Erythema multiforme oral lesions, the correct clinical manifestations are ( )

A. The most common lesion is a large erosion with a large amount of fibrinous exudate forming a thick pseudomembrane on the surface

B. Thick, black crusts often form on the lips

C. Significant pain that interferes with eating

D. Submandibular lymph nodes are swollen and tender

E. All of the above

3. 2010U2A1010 may belong to allergic diseases ( )

A. Glyphic tongue

B. Erythema multiforme exudative erythema

C. Map tongue

D. Glandular cheilitis

E. Angular cheilitis

4.2010U2A1011 Which of the following may be the trigger for the onset of angioedema ( )

A. Bacterial infection

B. Food

C. Autoimmunity

D. Trauma

E. Viruses

5. 2010U2A1015 Toxic epidermal necrolysis clinical manifestations are correct ( )

A. Epidermal lysis, positive Nikolsl sign, lesions appear only on the skin

B. The oral mucosa is red and swollen, bullae appear, and the Nikols sign is negative

C. Blistering erythema on the skin, redness and swelling of the oral mucosa, bullae without epidermal release

D. Positive Nikolsz sign only on the oral mucosa

E. Skin epidermal lysis, positive Nikol's sign, redness and swelling of the oral mucosa, bullae, large erosions, and easy bleeding

6. 2010U2A1027 Which of the following descriptions is wrong about erythema multiforme exudative ( )

A. is an allergic disease

B. Oral mucosa exhibits extensive erosions

C. Skin lesions are erythematous, blisters, and papules

D. Ocular lesions are iridocyclitis and empyema

E. May be accompanied by obvious systemic reactions, such as high fever, headache, etc

7.2002U2A1015 Which of the following allergic diseases is related to the season ( )

A. Erythema multiforme

B. Drug-allergic stomatitis

C. Allergic contact stomatitis

D. Angioedema

E. Toxic epidermal necrolysis

8. 2013U2A2086 A 38-year-old female patient, Xiao, had unexplained ulceration of the lips and mouth for 2 weeks, severe pain, and difficulty in speaking and eating. The patient had a similar illness in the last year. Examination: erosion of the lips, easy bleeding, bloody crusts. There are large erosions on the dorsum of the tongue, the floor of the mouth, and the right cheek, covered with a yellow pseudomembrane. Both palms have round or oval erythema. The possible diagnosis for this patient is ( )

A. Pemphigus

B. Drug-induced stomatitis

C. Discoid lupus erythematosus

D. Erythema multiforme exudative erythema

E. erosive lichen planus

9.2004U2A1030 The following clinical features of drug allergic stomatitis are correctly described as ( )

A. Skin bullae, white scars of the oral mucosa

B. Purple papules on the skin with white streaks in the mouth

C. Erythema and blisters on the skin, blisters and erosions of different sizes in the oral cavity

D. Skin blisters or maculopapular rashes, small clear blisters that merge with each other in the mouth

E. Erythema nodosum of the skin, recurrent ulcers in the mouth

10.2004U2A1041 Angioneuroedema occurs in which part of the most dangerous part ( )

A. Lips

B. Tongue

C. Palate

D. Epiglottitis

E. Eyelids

11.2004U2A1042 Serious complications that may occur with angioedema are ( )

A. Asthma

B. Swollen eyelids

C. Esophageal stricture

D. Restricted tongue movement

E. Laryngeal edema, suffocation

12.2004U2A1043 Which of the following locations does not generally occur angioneurotic edema ( )

A. Tongue

B. Lips

C. Eyelids

D. Palate

E. Throat

13.2002U2A1023 Which of the following descriptions is consistent with the clinical features of erythema multiforme ( )

A. The onset is seasonal, and it is more common in autumn and winter

B. Generally not accompanied by systemic reactions

C. The onset of the disease is slow, often prolonged, and not self-limited

D. Lesions are characterized by erythema, edema, bullae, erosions, etc

E. Lesions may occur in the oral cavity, but not on the skin, eyes, or genitals

14.2009U2A1070 The antibody produced by the body during the drug allergic stomatitis is ( )

A. IgA

B. IgG

C. IgM

D. IgD

E. IgE

GPT-4

1. E。 Erythema iris is a characteristic manifestation of erythema multiforme and is more characteristic than other options.
2. C。 Oral lesions of erythema multiforme often cause significant pain, which interferes with the patient's feeding.
3. B。 Erythema multiforme exudative is an allergic disorder.
4. B。 Food is one of the known triggers of angioedema.
5. E。 Typical clinical manifestations of toxic epidermal necrolysis include cutaneous epidermal lysis and a positive Nikols sign, with similar symptoms present with the oral mucosa.
6. D。 Ocular lesions are usually not a feature of erythema multiforme, so this description is incorrect.
7. A。 Erythema multiforme is associated with seasonal changes, especially in spring and fall.
8. D。 The patient's symptoms are consistent with the typical presentation of erythema multiforme exudative, including oral and skin lesions.
9. C。 Drug-allergic stomatitis usually manifests itself as inflammation and blisters of the skin and oral mucosa.
10. D。 Angioedema in the epiglottis is the most dangerous because it can cause airway obstruction.
11. E。 Serious complications of angioedema include laryngeal edema and asphyxia.
12. E。 Angioedema generally does not occur in the throat.
13. D。 Lesion features of erythema multiforme include erythema, edema, bullae, erosions, etc.
14. E。 Drug-allergic stomatitis is often associated with IgE-mediated reactions.

**Chapter 4 Oral Mucosal Ulcer Diseases**

1.2004U2A1017 The following factors may cause traumatic ulcers of the oral mucosa, except ( )

A. Eat hot noodles

B. Mechanical friction

C. Orthodontic wire

D. Chew gum

E. Sharp cusp irritation

2.2009U2A1030 Which of the following is a non-self-injurious ulcer ( )

A. Pressure ulcers

B. Self-injurious ulcers

C. Chemical burn ulcers

D. Heat-injured ulcers

E. All of the above

3.2009U2A1037 Among the following diseases, the main occurrencies on the oral mucosa are ( )

A. Recurrent aphthous ulcers

B. lichen planus

C. Erythema multiforme

D. Behcet's disease

E. Lupus erythematosus

4.2000U2A1005 Among the treatment measures for recurrent oral ulcers, the best short-term efficacy is ( )

A. Local oral anti-inflammatory, analgesic and healing

B. Surgical excision

C. Injection of transfer factor or oral levamisole

D. Treatment of systemic and local factors associated with the onset of the disease

E. Nutritional supplementation

5.2004U2A1018 Intensive use of corticosteroids may cause the following adverse consequences, except ( )

A. Diabetes mellitus

B. Decreased blood pressure

C. Decreased serum calcium

D. Candida infection

E. Peptic ulcers

6.2004U2A1028 Which of the following is NOT an oral allergic disease ( )

A. Riga's disease

B. Contact stomatitis

C. Drug-induced stomatitis

D. Sjohn's syndrome

E. Angioneurotic edema

7. 2004U2A1034 Clinical features of recurrent oral ulcers do not include ( )

A. It is more common in teenagers

B. The course of the disease is 7~14 days, and it is self-limited

C. It is more common in non-keratinized mucous membranes such as lips, cheeks, and tongue

D. Recurrent ulcers are round or oval in shape with a yellow pseudomembrane on the surface and red around them

E. Lesions can affect the perioral skin

8. 2012U2A2101 Male, 8 months old, tongue-tie ulceration and mass for 1 month, affecting eating and tongue activity, sought treatment. Examination: the deciduous incisors erupted, the edges were sharp, and ulcers of 1.0 cm × 0.8 cm in size could be seen at the tongue frenulum, the ulcers proliferated, the edges were raised and turned out, and there was a gray-white pseudomembrane on the surface, which was tough to the touch. This child may be diagnosed with ( )

A. Tumors

B. Riga's disease

C. Bednar aphthous ulcers

D. Periglandular aphthous ulcers

E. Pressure ulcers

9.2013U2A2076 Male, 33 years old. There are many ulcers in the mouth, the pain can not eat, the mucous membrane of the cheeks and the back of the tongue, more than ten ulcers of millet grains can be seen, scattered and distributed, and the surrounding mucosa is widely congested and swollen. History of multiple previous episodes of mild aphthous ulcers. Should be diagnosed as ( )

A. Herpetic gingivostomatitis

B. Herpangina

C. Herpetiform aphthous ulcers

D. Herpes zoster

E. Behcet's disease

10.2013U2A2078 Male, 8 months old, tongue-tie ulceration in January, mandibular breast middle incisors erupted, 1.0 cm × 0.8 cm ulcers were visible on both sides of tongue frenulum with ulcer hyperplasia, elevated margins and eversion valgus, and gray-white pseudomembranes. Palpation is firm. This child may be diagnosed with ( )

A. Lee-Freund's disease

B. Mouth sores in Pei's mouth

C. Periglandular aphthous ulcers

D. Decubitus ulcers

E. Thrush

11. 2002U2A1016 The clinical manifestations of mild aphthous ulcer are wrong ( )

A. The ulcer is depressed in the middle and the base is not hard

B. There is a redness band of hyperemia about 1 mm around the perimeter

C. The surface is covered with a light yellow pseudofilm

D. Burning sensation is obvious

E. Healing is not until treatment

12.2013U2A2087 Female, 35 years old. He complained of pain and ulceration on the left side of the lower lip for 4 days, affecting eating. The same history of 3~6 attacks per year. Oral examination: a 4mm diameter ulcer can be seen on the mucosa on the left side of the lower lip, the periphery is red, there is a yellow pseudomembrane on the surface of the ulcer, and the bottom of the ulcer is slightly depressed, and the tenderness is obvious. Clinically diagnosable as ( )

A. Recurrent mild aphthous ulcers

B. Recurrent herpetiform stomatitis

C. Recurrent severe aphthous ulcers

D. Erythema multiforme

E. Herpetic stomatitis

13.2013U2A2088 A 30-year-old female complained of moderate pain in the lower lip for 2 days, which was aggravated by eating irritating food; Oral examination: a 5 mm diameter ulcer was found on the mucosa of the lower left 1 labial side, covered with pseudomembrane, and the ulcer base was congested and tender. The chief complainant has a history of similar diseases for about 3 years, and has 4~6 attacks per year. The ulcer heals on its own. Probable( )

A. Discoid lupus erythematosus

B. Herpetiform stomatitis

C. Mild aphthous ulcers

D. Severe aphthous ulcers

E. Erythema multiforme

14.2013U2A2089 female, 46 years old, mouth ulcers recurrent for 3 years, with an interval of 1~2 months, mostly before menstruation, ranging from 1~3 each time, mainly located in the lower lip and tongue and other parts, the pain is obvious, and it heals in about 7~10 days. In order to achieve the desired therapeutic effect, it is advisable to take ( )

A. Local symptomatic treatment of the oral cavity

B. Systemic antibiotics

C. Multivitamin supplementation

D. Oral estrogen + oral local symptomatic therapy

E. Injection of transfer factors

15.2013U2A1029 The following description of the clinical manifestations of severe aphthous ulcer is incorrect ( )

A. The edge of the ulcer is neat, crater-like

B. The edge of the ulcer is uneven, rat-like, and has granulation tissue at the bottom

C. The ulcer is regular-shaped, red, swollen and raised

D. The diameter of the ulcer can reach 10~30mm

E. Ulcers deep into the submucosa up to the muscular layer

16. 2013U2A2091 patient, male, 50 years old. Oral and palate ulceration pain for more than 1 month. Has a history of recurrent seizures. Oral examination: 1 large and deep ulcer on the soft palate, 1.5cm in diameter, like a crater, red, swollen and bulging around but neat, slightly concave at the bottom, with a pseudomembrane on the surface, and a defect in the palatal lobe. Initial diagnosis is ( )

A. Cancerous ulcers

B. Tuberculous ulcers

C. Traumatic ulcers

D. Recurrent severe aphthous ulcers

E. Necrotizing salivary gland metaplasia

17.2013U2A2092 Jiang, female, 36 years old, with multiple oral ulcers for 1 week, pain and inability to eat. Examination: More than 10 ulcers of millet grains were found on the mucosa of both cheeks and tongue, scattered and distributed, and the surrounding mucosa was congested and red. The patient has a history of multiple similar seizures in the past. The patient should be diagnosed with ( )

A. Behcet's disease

B. Herpes zoster

C. Herpetiform aphthous ulcers

D. Herpangina

E. Herpetic gingivostomatitis

18.2013U2A2093 Male 40 years old, found that the palate was swollen and ulcerated for more than 2 months. Investigation: A deep ulcer was seen at the junction of the soft and hard palate, which could reach the bone surface, with a diameter of 1.3cm, irregular shape, clear boundaries, and a raised ulcer edge. Biopsy revealed tissue necrosis and squamous metaplasia of the salivary ducts and acinar epithelium. Probable( )

A. Palatal squamous cell carcinoma

B. Oral tuberculosis

C. Periglandular aphthous ulcers

D. Necrotizing salivary gland metaplasia

E. Palatal adenocarcinoma

19.2014U2A2086 A 30-year-old male patient, Duan, visited the hospital for two years due to repeated ulceration of the oral mucosa, and was examined: there were 2 small ulcers on the right lingual margin, the ulcers were slightly concave, showing yellow pseudomembrane, and the tenderness was obvious. Ocular conjunctivitis, facial boils, and ulcers of the external genitalia should be considered for diagnosis ( )

A. Gonorrhea

B. Behcet's disease

C. Herpes zoster

D. Herpetic stomatitis

E. Recurrent mouth ulcers

20.2014U2A2087 Male, 69 years old. Pain at the base of the left tongue for 3 days, affecting eating. Oral examination: only the lower left 6 remained, the crown remained. The missing tooth has been restored with a denture. An ulcer with a diameter of 1 cm was seen at the base of the left tongue, which was tender to the touch. The appropriate treatment is ( )

A. Ulcer excision

B. Remove the lower left 6

C. Chemotherapy

D. Sharpen off the lower left 6 sharp edges

E. Prednisone plus lidocaine ulcer basal infiltration injection

21.2014U2A2088 Male, 69 years old, only the lower left 7 remains, and the rest are removable dentures. complained of accidentally biting a hard object two weeks ago, a small piece of the crown of the tooth collapsed, because the pain was not obvious and was not paid attention to, recently there was pain at the root of the left tongue, and the oral examination showed that there was an ulcer at the root of the left tongue, and the more appropriate treatment was ( )

A. Remove the lower left 7

B. Excision of the ulcer on the left edge of the tongue

C. Radiotherapy

D. Adjust the sharp edge of the lower left 7 sharp, followed by observation and follow-up

E. Prednisone plus lidocaine infiltrated into the base of the ulcer 10 times

22.2014U2A2089 Hu, male, 66 years old, nasopharyngeal carcinoma was subjected to radiotherapy after surgery, and the oral mucosa ulcerated in a large area after two radiotherapy treatments, and the pain was obvious. Seek treatment. The possible diagnosis of this patient is ( )

A. Allergic stomatitis

B. Contact stomatitis

C. Radiation stomatitis

D. Candida albicans infection

E. Acute infectious stomatitis

23.2014U2A2090 male, 60 years old, nasopharyngeal carcinoma after postoperative radiotherapy, after two radiotherapy treatments, the oral mucosa ulcerated in a large area, and the pain was obvious. The possible diagnosis of this patient is ( )

A. Candida albicans infection

B. Radiation stomatitis

C. Acute infectious stomatitis

D. Allergic stomatitis

E. Contact stomatitis

24.2003U2A1002 Clinical manifestations of herpetiform ulcer ( )

A. Blisters and neuralgia in unilateral banded clusters

B. Sudden onset of acute inflammation with a history of medication prior to onset

C. History of trauma, ulcer morphology often coincident with mechanical stimuli

D. In the early stage, the mucosal of the affected area is congested, scattered with small spots as white as snow, which can soon merge into white patches and continue to expand and spread

E. Ulcers are small and numerous, scattered in any part of the mucosa, less than 2 mm in diameter, and similar ulcers can coalesce into patches

25.2002U2A1024 The following ulcers have recurrence ( )

Aphthous ulcers of severe aphthous ulcers

B. Cancerous ulcers

C. Tuberculous ulcers

D. Traumatic ulcers

E. Necrotizing salivary gland metaplasia

26.2002U2A1025 Adrenocorticosteroids can be injected locally in ( )

A. Tuberculous ulcers

B. Snow mouth disease

C. Periglandular aphthous ulcers

D. Herpetic stomatitis

E. None of the above

27.1999U2A1005 Which of the treatment measures for recurrent oral ulcers has the best effect ( )

A. Local oral anti-inflammatory, analgesic and healing-promoting oral cavity

B. Surgical excision

C. Injection of transfer factor or oral levamisole

D. Whenever possible, identify and treat systemic and local factors associated with the onset of the disease

E. Nutritional supplementation

28.2015U2A1018 The most commonly used method for the treatment of severe recurrent aphthous ulcers that do not heal for a long time is ( )

A. Oral antibiotics

B. Localized cautery of 10% silver nitrate solution

C. Triamcinolone acetonide suspension for local injection

D. Surgical excision of the lesion

E. Fluconazole paste is applied topically

29.2015U2A2133 Ulceration of the mucosa at the junction of the hard and soft palate of the infant due to artificial feeding with a hard rubber nipple, called ( )

A. Bednar ulcer

B. Riga-Fede ulcer

C. Periglandular aphthous ulcers

D. Traumatic ulcers

E. Tuberculous ulcers

30.2015U2A2134 Ulcers formed by the corresponding mucosa caused by the stimulation of the sharp edges of the stump and the residual crown are called ( )

A. Bednar ulcer

B. Riga-Fede ulcer

C. Periglandular aphthous ulcers

D. Traumatic ulcers

E. Tuberculous ulcers

31.2019U2A1004 Infants sucking their thumbs or using too hard rubber nipples cause ulcers on the mucosal surface of the bilateral wing hooks, which is called ( )

A. Traumatic ulcers

B. Pressure ulcers

C. Bednar ulcer

D. Riga-Fede ulcer

E. Recurrent aphthous ulcers

32.2017U2A2079 In the systemic treatment of recurrent aphthous ulcers, the correct method of using adrenocorticosteroids is ( )

A. Prednisone 10~30mg/d, divided into 3 oral doses, and gradually reduce the dose after ulcer control

B. Prednisone 60mg/d, divided into 3 oral doses, reduce the dose after controlling the disease

C. Prednisone 60~80mg/d, divided into 3 oral doses, reduce the dose after controlling the disease

D. Prednisone 80~100mg/d, divided into 3 oral doses, reduce the dose after controlling the disease

E. Prednisone 100~120mg/d, divided into 3 oral doses, reduce the dose after controlling the disease

33.2017U2A1056 The description of the characteristics of recurrent oral ulcers is correct ( )

A. Specific, recurrent, and self-limited

B. Contagious, cyclical and self-limiting

C. Specific, infectious, and recurrent

D. Periodic, recurrent, and self-limited

E. Clustered, specific, and cyclical

GPT-4

1. D。 Chewing gum usually does not cause a traumatic ulcer because it is not hard or sharp enough to injure the oral mucosa.
2. A。 Pressure ulcers are caused by prolonged stress and are not self-injurious ulcers.
3. A。 Recurrent aphthous ulcers occur mainly on the oral mucosa.
4. C。 Injection of transfer factor or oral levamisole is the treatment for recurrent oral ulcers and has good short-term efficacy.
5. B。 Heavy corticosteroid use may increase blood pressure, not lower it.
6. A。 Riga disease is not an allergic disease of the oral cavity.
7. E。 Recurrent canker sores generally do not affect the perioral skin.
8. C。 Bednar aphthous sores usually occur in infants and are associated with sharp tooth cuts.
9. E。 Behcet's disease can cause sores in many locations, including the mouth.
10. B。 Belle's mouth sores can occur near the tongue tie and are associated with the eruption of baby teeth in babies.
11. E。 Mild aphthous ulcers usually heal on their own and don't necessarily require treatment to heal.
12. A。 Recurrent minor aphthous ulcers are characterized by recurrent small ulcers.
13. C。 Mild aphthous sores are described as consistent with this condition and are self-healing.
14. D。 In women, particularly for ulcers associated with the menstrual cycle, oral estrogen plus topical symptomatic therapy may be effective.
15. B。 The edges of severe aphthous ulcers are usually neat and not rat-like.
16. D。 Recurrent severe aphthous ulcers can lead to large, deep ulcers.
17. C。 Symptoms of herpetiform aphthous sores include multiple small sores and recurrent flare-ups.
18. D。 Necrotizing salivary metaplasia may result in squamous metaplasia deep in ulcers and salivary duct epithelium.
19. B。 Behcet's disease should be considered in the setting of multiple sites of recurrent ulcers.
20. D。 Grinding away sharp edges may alleviate the resulting ulcers.
21. D。 Sharpening the edges reduces mechanical irritation, which may reduce pain and promote healing.
22. C。 Large ulceration of the oral mucosa after radiation therapy may be a cause of radiation stomatitis.
23. B。 Radiation stomatitis is a common complication after radiotherapy.
24. E。 Herpetiform ulcers are characterized by small, numerous, scattered ulcers.
25. A。 Severe aphthous ulcers are recurrent.
26. E。 Adrenocorticosteroids are generally not used as local injections to treat these types of ulcers.
27. D。 Identifying and treating systemic and local factors associated with the onset of the disease as much as possible is the most effective treatment for recurrent canker sores.
28. C。 Local injections of triamcinolone acetonide suspension are commonly used to treat severe recurrent aphthous ulcers that do not heal for a long time.
29. B。 Riga-Fede ulcers usually occur in infants and are associated with the use of too hard pacifier feeding.
30. D。 Traumatic ulcers are often caused by irritation of the sharp edges of the stump and crown.
31. D。 Riga-Fede ulcers are sores caused by babies sucking their thumbs or using too hard pacifiers.
32. A。 Prednisone 10~30mg/d, divided into 3 oral doses, and gradually reducing the dose after ulcer control is a common treatment for recurrent aphthous ulcer.
33. D。 Recurrent canker sores are characterized by periodic, recurrent, and self-limited.

**Chapter 5 Oral Mucosal Bullous Diseases**

1. 2004U2A1014 Pemphigoid can have the following clinical manifestations, except ( )

A. Skin blisters

B. Microstoma

C. Positive Nikol's sign

D. Exfoliative gingivitis

E. Bulbar conjunctival adhesions

2. 2004U2A1015 The most common site of lesions in the oral cavity of cicatricial pemphigoid is ( )

A. Gums

B. Lower lip

C. Soft palate

D. Palate

E. Buccal mucosa

3.2009U2A1021 Among the following diseases, spontaneous remission is ( )

A. Pemphigus vulgaris

B. Cicatricial pemphigoid

C. Erosive lichen planus

D. Bullous pemphigoid

E. Epidermolysis bullosa

4.2009U2A1023 Blisters can be quickly formed by gently pushing the skin or mucous membranes with a finger on the normal appearance, which is called ( )

A. Positive peeling test

B. Epidermolysis

C. Positive Nikol's sign

D. Positive OT test

E. Positive penicillin test

5.2009U2A1026 Among the following bullous diseases, which can be life-threatening, are ( )

A. Cicatricial pemphigoid

B. Pemphigus vulgaris

C. Bullous pemphigoid

D. Pemphigoid multiforme

E. Bullous lichen planus

6. 2009U2A1031 Among the various types of pemphigus, the least damage to the oral mucosa is ( )

A. Pemphigus vulgaris

B. Proliferative pemphigus

C. Pemphigus foliaceus

D. Pemphigus erythematosus

E. The probability of occurrence of the above types is approximately the same

7. 2009U2A1034 Hormone therapy for pemphigus is characterized by ( )

A. The condition generally does not recur after stopping the drug

B. Emphasis on large volume, speed, and rapid control of inflammation

C. Emphasize gradual, impatient, and slowly increase from low amounts

D. Can be replaced by traditional Chinese medicine to reduce the adverse effects of hormones

E. It can be dynamically divided into four stages: initiation, control, consolidation and maintenance

8.2009U2A1035 Among the following diseases, the loss of connections between epithelial cells is an autoimmune disease.

A. Behcet's disease

B. Pemphigus

C. Pemphigoid

D. Lupus erythematosus

E. erythema multisexual

9. 2012U2A1004 The most accurate description of the characteristics of hormone therapy for pemphigus is ( )

A. Quantity is large and fast

B. Increment from low amount

C. Initiation, control, consolidation, and maintenance

D. Can be replaced by traditional Chinese medicine

E. No recurrence of the condition after discontinuation of the drug

10. 2004U2A1029 Benign mucous membrane pemphigoid also known as ( )

A. Bullous pemphigoid

B. Erythematous pemphigoid

C. Proliferative pemphigoid

D. Scarring pemphigoid

E. Deciduous pemphigoid

11.2004U2A1033 The following diseases are autoimmune diseases ( )

A. Erythema multiforme, pemphigus, lupus erythematosus

B. Pemphigus, pemphigoid, lupus erythematosus

C. Pemphigoid, erythema multiforme, lupus erythematosus

D. Behcet's disease, lupus erythematosus, angioedema

E. Lupus erythematosus, angioedema, erythema multiforme

12. 2010U2A1013 What type of pemphigus usually presents with oral lesions ( )

A. Proliferative pemphigus

B. Pemphigus vulgaris

C. Pemphigus foliaceus

D. Bullous pemphigoid

E. Pemphigus erythematoform

13.2010U2A1031 Which of the following lesions will form a scar after healing ( )

A. Pemphigus vulgaris

B. Bullous pemphigoid

C. Cicatricial pemphigoid

D. Pemphigoid multiforme

E. B+C

14.2002U2A1018 Cicatricial pemphigoid can occur anywhere in the oral cavity, and the highest incidence is ( )

A. Gums

B. Soft palate

C. Hard palate

D. Tongue

E. Buccal mucosa

15.2014U2A2091 Male, 68 years old, oral ulcer 3 months after visiting the clinic, physical examination found that the buccal mucosa was a large area of erosion, with marginal expansion, and the surface of the erosion surface was covered with a thick gray-white pseudofilm, which was patchy. The diagnosis of pemphigus was probable by exfoliative cytology. The blood test routine and classification of white blood cells were 1.6×10⁹/L, and neutrophils accounted for 82%. The most realistic treatment of choice for this case is ( )

A. 1%~2% procaine solution gargle to relieve pain

B. Topical application of corticosteroid ointment

C. Systemic antibiotics and oral chlortetracycline 0.25% gargle

D. Nystatin in the mouth, alkaline mouthwash topical in the mouth

E. Maintain oral hygiene and remove irritants

16.2014U2A1009 Exfoliative gingival lesions may be a sign of the following diseases, but do not include ( )

A. Pemphigus

B. Pemphigoid

C. Vitiligo

D. Lichen planus

E. Lupus erythematosus

17.2014U2A1010 The following diseases do not usually manifest as lip erosions are ( )

A. Chronic cheilitis

B. Discoid lupus erythematosus

C. Erythema multiforme

D. Proliferative pemphigus

E. Bullous pemphigoid

18.2005U2A1013 Pemphigus belongs to ( )

A. Allergic diseases

B. Autoimmune diseases

C. Immunodeficiency disorders

D. Infectious diseases

E. Communicable diseases

19.2004U2A1044 Which of the following statements related to pemphigoid is false ( )

A. Have autocirculating antibodies

B. Anti-basement membrane antibodies

C. Anti-cellular-cell antibodies

D. Direct immunofluorescence can be used

E. Anti-basement membrane zone antibodies are mainly IgG

20.2002U2A1026 Which of the following is NOT a clinical manifestation of cicatricial pemphigoid ( )

A. Skin blisters

B. Microstoma

C. Exfoliative gingivitis

D. Positive Nikolsz sign

E. Bulb conjugated membrane adhesions

GPT-4

1. B. Microstoma. Causes: Pemphigoid is characterized by blisters of the skin and mucous membranes, and micromouth is not typical.
2. E. Buccal mucosa. Causes: Cicatricial pemphigoid is most commonly found in the mouth on the buccal mucosa.
3. C. Erosive lichen planus. Causes: erosive lichen planus is self-limiting and resolves spontaneously.
4. C. Positive Nikol's sign. Causes: A positive Nikols sign means that gentle pushing of the skin or mucous membranes can lead to blister formation.
5. C. Bullous pemphigoid. Causes: Bullous pemphigoid can be life-threatening due to extensive skin damage and possible complications.
6. E. The probability of occurrence of the above types is approximately the same. Causes: There is no significant difference in the probability of oral mucosal lesions in each type of pemphigus, and oral lesions can occur in each type.
7. E. It can be dynamically divided into four stages: initiation, control, consolidation and maintenance. Causes: Hormone therapy for pemphigus is usually a phased process that includes initiation, control, consolidation, and maintenance.
8. B. Pemphigus. Causes: Pemphigus is an autoimmune disease characterized by loss of connections between epithelial cells.
9. C. Initiation, control, consolidation, and maintenance. Causes: Hormone therapy for pemphigus is characterized by being carried out in stages.
10. D. Scarring pemphigoid. Causes: Benign mucous membrane pemphigoid is often referred to as scarring pemphigoid because it is relatively mild.
11. B. Pemphigus, pemphigoid, lupus erythematosus. Causes: All three of these diseases are known to be autoimmune diseases.
12. B. Pemphigus vulgaris. Causes: Pemphigus vulgaris is a common oral lesion.
13. C. Cicatricial pemphigoid. Causes: Cicatricial pemphigoid can form scarring after healing.
14. E. Buccal mucosa. Causes: Cicatricial pemphigoid occurs most often on the buccal mucosa.
15. B. Topical application of corticosteroid ointment. Causes: Topical corticosteroid therapy is usually preferred for pemphigus.
16. C. Vitiligo. Causes: Vitiligo is a skin condition caused by depigmentation and is not directly related to exfoliative gingivitis.
17. D. Proliferative pemphigus. Causes: Proliferative pemphigus does not usually present as lip erosions.
18. B. Autoimmune diseases. Causes: Pemphigus is an autoimmune disease characterized by blisters on the skin and mucous membranes.
19. B. Anti-basement membrane antibodies. Causes: Pemphigoid is characterized by intercellular antibodies, not anti-basement membrane antibodies.
20. A. Skin blisters. Causes: Cicatricial pemphigoid mainly affects the mucous membranes, and skin blisters are not a typical finding.

**Chapter 6 Oral Markings Diseases**

1.2004U2A1016 Discoid lupus erythematosus is the most common site in the oral mucosa ( )

A. Cheeks

B. Soft palate

C. Back of the tongue

D. Upper lip redness

E. Lower lip redness

2.2009U2A1016 Diseases that do not undergo malignant transformation are ( )

A. Vitiligo

B. Chronic discoid lupus erythematosus

C. lichen planus

D. Oral erythema

E. Recurrent aphthous ulcers

3.2009U2A1029 The most effective treatment for oral leukatosis is ( )

A. Surgical excision

B. Oral vitamin A acid

C. Topical application of vitamin A acid preparation

D. Removal of local irritants

Local injection of 2.5% prednisone suspension

4.2000U2A1002 Oral mucosal leukoplakia may have the following clinical manifestations, except for ( )

A. White or off-white homogeneous plaques or crumpled paper-like plaques

B. White irregular bulges with papillary protrusions

C. Granular red-white interlesions

D. Ulceration formation on the basis of white plaques

E. White curd-like pseudomembrane

5. 2012U2A1003 Oral erythema also has the following names, in addition to ( )

A. Red spots

B. Red proliferative lesions

C. Erythema multiforme

D. Erythema proliferative

E. Quillette erythema

6.2004U2A1031 Which of the following statements is true about the pathogenic factors of vitiligo ( )

A. Quitting smoking can lead to vitiligo

B. Chewing betel nut can cause white patches

C. Plaque irritation can lead to white patches

D. Vitiligo has a familial predisposition to hereditary properties

E. Immunocompromised can cause vitiligo

7. 2004U2A1035 Adverse reactions of quinoline chloride include the following, except ( )

A. Visual impairment

B. Decreased blood pressure

C. Nausea, vomiting

D. Liver impairment

E. Decreased white blood cell count

8. 2010U2A1014 Granular leukoplakia is more common in ( )

A. Mucosa in the corner of the mouth

B. Soft palatal pharynx

C. Dorsopharyngeal part of the tongue

D. Floor of the mouth and tongue

E. Gums

9. 2010U2A1018 is an autosomal dominant disease ( )

A. Vitiligo

B. Gingivitis

C. White wrinkle

D. Adolescent periodontitis

E. Color blindness

10.2010U2A1020 Epidemiological statistics, females are more likely to occur than males ( )

A. Vitiligo

B. Discoid lupus erythematosus

C. C. Cies

D. Map tongue

E. Gum cancer

11.2010U2A1021 The following are not the most common sites of oral leukratosis( )

A. Hard palate

B. Lip redness

C. Floor of the mouth

D. Dorsum of the tongue

E. Buccal mucosa

12.2010U2A1022 Skin lichen planus lesions are mainly manifested as ( )

A. Reticulated type

B. Flat papules

C. Annular type

D. Plaque type

E. Streaked type

13.2010U2A1025 Which of the following chronic discoid lupus erythematosus laboratory test results is incorrect ( )

A. Reduction of OKT4/OKT8 ratio

B. Accelerated erythrocyte sedimentation rate

C. Elevated globulin

D. Rheumatoid factor positive

E. Positive for antinuclear antibodies

14.2011U2A1006 Histopathological manifestations of OLP ( )

A. Hypogoniosis or dysplasia

B. Epithelial hyperkeratosis

C. Hypoangulation

D. Liquefaction and degeneration of basal cells

E. Dense bands of lymphocyte infiltration in the connective tissue of the lamina propria

15.2013U2A2077 Male, 22 years old, white lesions on the tongue for 5 years, aggravated for half a year. Examination: On the left and right sides of the dorsal part of the tongue, two white plaques of about 1.1~1.3 cm can be seen respectively, with irregular edges, and striped white lines on the mucosa on the inner side of the lower lip. The clinical diagnosis should be ( )

A. Lichen planus

B. Candidiasis albicans

C. Herpes simplex

D. Cancerous ulcers

E. Coccal stomatitis

16.2010U2A1033 The best parts of oral lichen planus are ( )

A. Gums

B. Soft palate

C. Hard palate

D. Tongue

E. Buccal mucosa

17. 2002U2A1019 Diseases that may be related to light exposure are ( )

A. Erythema multiforme

B. Angioedema

C. Pemphigoid

D. Discoid lupus erythematosus

E. Angular cheilitis

18.2004U2A1040 Which cell-mediated inflammatory disease of oral lichen planus ( )

A. NK cells

B. B lymphocytes

C. T lymphocytes

D. Neutrophils

E. Macrophages

19.2014U2A1011 Oral manifestations of lichen planus excl. ( )

A. Erosion

B. Blisters

C. Herpes

D. Plaque

E. Chapped

20.2014U2A2092 Male, 55 years old. A white patch appeared on the red part of the right lip for 1 year. No symptoms. The patient has a habit of smoking and drinking. Oral examination: a white patch can be seen on the red part of the right lip, which is homogeneous and cannot be wiped off, and no spores or hyphae were found in 3 microscopic examinations of the smear. Improper treatment is ( )

A. Stop smoking

B. Oral vitamin A

C. Topical application of 0.2% tretinoin

D. Silver nitrate topical treatment of white plaques

E. Regular follow-up, if there is an indication of malignant transformation, it should be excised immediately

21.2014U2A2093 Male, 58 years old, a white plaque appeared on the mucosa of the right cheek, homogeneous, could not be wiped off, no obvious pain on palpation, no spores or hyphae were found in the microscopic examination of the smear three times, the lower right 3456 was a poor restoration, no history of smoking and alcoholism, and the treatment measures were inappropriate ( )

A. Removal of defective restorations

B. Oral vitamin A

C. Topical application of 0.2% tretinoin

D. Silver nitrate is treated topically with white plaques to promote plaque regression

E. Regular follow-up, early surgical resection may be considered if there are indications of malignant transformation

22.2008U2A1001 The following measures can be used for vitiligo treatment, except for ( )

A. Quit smoking

B. Surgical excision

C. Cod liver oil

D. Silver nitrate cautery

E. Oral vitamin A

23.2005U2A1014 Among the precancerous lesions, the highest chance of carcinogenesis is ( )

A. Lichen planus

B. Vitiligo

C. Erythema

D. Warts

E. Chronic ulcers

24.2005U2A1015 Which of the following statements is false about the clinical classification of vitiligo ( )

A. Divided into homogeneous and non-homogeneous types

B. The heterogeneous type is further divided into verrucous type, granular type, and ulcer type

C. The homogeneous type manifests as homogeneous plaques or wrinkles on the surface

D. The verrucous type is heterogeneous

E. Heterogeneous types include verrucous, granular, and atrophic

25.2004U2A1045 There are ( ) that are not related to the pathogenesis of OLP

A. Neuropsychiatric factors

B. Viral infection

C. Excessive smoking and drinking

D. Immunological factors

E. Certain chemical drugs, possible genetic factors

26.2004U2A1046 Which of the following is a stimulus for oral leukatosis ( )

A. Smoking

B. Alcoholism

C. Likes spicy food

D. Prefer to eat too hot food

E. Prefer crunchy, hard foods

27.2004U2A1047 Chronic discoid lupus erythematosus skin lesions are characterized by ( )

A. Erythema

B. Hair follicle keratin plugs, scales, keratin plugs

C. Hyperpigmentation and/or hypopigmentation

D. Telangiectasia, atrophy, and scarring

E. All of the above

28.2003U2A1003 Treatment of discoid lupus erythematosus can be used ( )

A. Ribavirin

B. Chloroquine

C. Tretinoin

D. Fluconazole

E. Chlorpheniramine

29.2002U2A1027 Epidemiological statistics show that women are more likely to occur than men ( )

A. Chronic discoid lupus erythematosus

B. Chronic cheilitis

C. Gum cancer

D. Glyph tongue

E. Caries disease

30.1999U2A1006 Which of the following is NOT a pathological manifestation of lichen planus ( )

A. Incomplete keratinization of the epithelium

B. Liquefaction and denaturation of basal cells

C. Banded infiltrates of lymphocytes in the lamina propria

D. Presence of colloidal bodies

E. Collagen fibrillary degeneration

31.1999U2A1007 The following items can be used to distinguish white edema from vitiligo, except for ( )

A. White edema with soft texture

B. White edema with unclear borders

C. White edema with a smooth surface

D. White edema without epithelial keratinization

E. White edema, swelling of acanthocytes

32.2015U2A1019 The auxiliary examination to confirm the carcinogenesis of oral leukoplakia is ( )

A. Potassium hydroxide smear

B. PAS staining

C. Histopathological examination

D. Exfoliated cell examination

E. Toluidine blue staining

33.2018U2A1012 The oral mucosal diseases most closely related to smoking and drinking are ( )

A. Oral lichen planus

B. Mucous membrane pemphigoid

C. Submucosal fibrosis

D. Oral leukoplakia

E. White spongy nevus

34.2017U2A1059 Diagnosis of vitiligo can be based on ( )

A. Can be made based on clinical examination

B. Available from blood tests

C. Can be confirmed based on family history

D. Must be confirmed by pathological examination

E. Must be made by indirect immunofluorescence

35.2017U2A1058 Which of the following measures should not be used for the treatment of vitiligo ( )

A. Oral vitamin A

B. Silver nitrate cautery

C. Quit smoking

D. Surgical excision

E. Topical cod liver oil

36.2017U2A1057 The pathogenesis of oral lichen planus may be related to the following factors: ( )

A. Smoking, trauma, psychological factors

B. Genetics, smoking, alcoholism, bacterial infections

C. Psychological factors, smoking, poor restoration

D. Endocrine factors, viral infections, autoimmunity

E. Psychological factors, poor prosthetics, endocrine factors

37.2017U2A2080 In the epithelial lamina propria of oral lichen planus (OLP), a large number of lymphocytes are infiltrated in a dense band, which of the following factors is related to ( )

A. Psychological factors

B. Endocrine factors

C. Microcirculatory disorder factors

D. Immune factors

E. Heredity

38.2020U1A1001 The pathological characteristics of oral lichen planus are ( )

A. Excessive positive angulation of the surface

B. Clear basement membrane

C. Basal cell keratinization

D. Lamina propria lymphocyte cell infiltration zone

E. Abnormal hyperplasia of the spinous layer

GPT-4

1. A. Cheeks. Causes: Discoid lupus erythematosus is commonly found in the buccal mucosa in the mouth.
2. B. Chronic discoid lupus erythematosus. Causes: Chronic discoid lupus erythematosus usually does not become malignant.
3. D. Removal of local irritants. Causes: Effective treatment of oral leukatoses begins with the removal of possible local irritants.
4. E. White curd-like pseudomembrane. Causes: White curd-like pseudomembranes usually refer to Candida infections rather than oral leukoplakia.
5. E. Quillette erythema. Causes: Quilaite erythema is not another name for oral erythema.
6. B. Chewing betel nut can cause white patches. Causes: Betel nut chewing is a known risk factor for oral leukoplakia.
7. B. Decreased blood pressure. Causes: Adverse effects of quinoline chloride do not typically include a decrease in blood pressure.
8. A. Mucosa in the corner of the mouth. Causes: Granular leukoplakia is more common in the mucosa of the corner of the mouth.
9. A. Vitiligo. Causes: Vitiligo is an autosomal dominant disorder.
10. B. Discoid lupus erythematosus. Causes: Discoid lupus erythematosus is more common in women than in men.
11. A. Hard palate. Causes: Oral leukatoses do not usually occur in the hard palate.
12. B. Flat papules. Causes: Cutaneous lichen planus lesions are predominantly flat papules.
13. D. Rheumatoid factor positive. Causes: Laboratory findings for chronic discoid lupus erythematosus are infrequently positive for rheumatoid factor.
14. B. Epithelial hyperkeratosis. Causes: Histopathological manifestations of lichen planus usually do not include epithelial hyperkeratosis.
15. A. Lichen planus. Causes: According to the described clinical presentation, white plaques and streaks of the mucosa on the dorsum of the tongue and medial of the lower lip are consistent with the characteristics of lichen planus.
16. E. Buccal mucosa. Causes: Oral lichen planus is most commonly found in the buccal mucosa.
17. D. Discoid lupus erythematosus. Causes: The onset of discoid lupus erythematosus is associated with sun exposure.
18. C. T lymphocytes. Causes: Oral lichen planus is an inflammatory disease mediated by T lymphocytes.
19. B. Blisters. Causes: The oral manifestations of lichen planus usually do not include blisters.
20. E. Regular follow-up, if there is an indication of malignant transformation, it should be excised immediately. Causes: This is an inappropriate approach because if there is an indication for cancer, further diagnosis and treatment should be done immediately, rather than just regular follow-up.
21. D. Silver nitrate is treated topically with white plaques to promote plaque regression. Causes: Silver nitrate should not be used to treat vitiligo as this may mask the true nature of the lesion.
22. D. Silver nitrate cautery. Causes: Silver nitrate cautery is not recommended for the treatment of vitiligo.
23. B. Vitiligo. Causes: Vitiligo has a relatively high chance of becoming cancerous and is considered a precancerous lesion.
24. E. Heterogeneous types include verrucous, granular, and atrophic. Causes: Atrophic forms are usually not included in the classification of heterogeneous types.
25. C. Excessive smoking and drinking. Causes: Excessive smoking and alcohol consumption are associated with the pathogenesis of OLP.
26. A. Smoking. Causes: Smoking is a known irritant of oral leukatosis.
27. E. All of the above. Causes: These are all features of skin lesions in chronic discoid lupus erythematosus.
28. B. Chloroquine. Causes: Chloroquine is a commonly used drug to treat discoid lupus erythematosus.
29. A. Chronic discoid lupus erythematosus. Causes: Chronic discoid lupus erythematosus is more common in women than in men.
30. D. Presence of colloidal bodies. Causes: Colloidal bodies are characteristic of systemic lupus erythematosus, not lichen planus.
31. D. White edema without epithelial keratinization. Causes: The difference between white edema and vitiligo is not the presence or absence of epithelial keratosis.
32. C. Histopathological examination. Causes: Histopathological examination is the gold standard for confirming the diagnosis of oral leukoplakia carcinogenesis.
33. D. Oral leukoplakia. Causes: Oral leukoplakia is closely related to smoking and alcohol consumption.
34. D. Must be confirmed by pathological examination. Causes: The diagnosis of vitiligo requires pathological examination to confirm it.
35. B. Silver nitrate cautery. Causes: Silver nitrate cautery is not recommended for the treatment of vitiligo.
36. C. Psychological factors, smoking, poor restoration. Causes: The pathogenesis of oral lichen planus is associated with psychological factors, smoking, and poor restorations.
37. D. Immune factors. Causes: Banded infiltration of lymphocytes in oral lichen planus (OLP) is associated with immune factors.
38. D. Lamina propria lymphocyte cell infiltration zone. Causes: One of the pathological features of oral lichen planus is banded infiltrates of lymphocytes in the lamina propria.

**Chapter 8 Diseases of the Lips and Tongue**

1. The clinical manifestations of 2009U2A1019 May-Roche syndrome are ( )

A. Cracked tongue, angular cheilitis, glandular cheilitis

B. Cracked tongue, facial paralysis, granulomatous cheilitis

C. Geographic tongue, angular cheilitis, anemia

D. Geographic tongue, facial paralysis, anemia

E. Tongue papillitis, facial paralysis, granulomatous cheilitis

2.2009U2A1025 Which of the following is NOT a clinical feature of the geographic tongue ( )

A. It is more common in adults

B. It is more common on the dorsum of the tongue, the tip of the tongue and the margin of the tongue

C. Filiform papillae atrophy of the tongue

D. Diurnal "wandering" conjugation features

E. Generally no pain or other adverse sensations

3. 2004U2A1021 Which of the following is NOT a clinical manifestation of the geographic tongue ( )

A. The red zone can be eroded

B. The exfoliation zone is slightly depressed

C. Filiform papillae flakes

D. Fungal papillae are clearly visible

E. The dorsum of the tongue forms a red smooth area

4. The clinical manifestations of 2004U2A1025 Mero syndrome are ( )

A. Glyphical tongue, facial paralysis, angular cheilitis

B. Glyphical tongue, facial paralysis, glandular cheilitis

C. Glyphical tongue, facial paralysis, granulomatous cheilitis

D. Geographic tongue, facial paralysis, angular cheilitis

E. Geographic tongue, angular cheilitis, granulomatous cheilitis

5.2004U2A1032 The following factors can cause angular cheilitis, except ( )

A. Malnutrition

B. Cold stimulation

C. Bacterial infection

D. Fungal infection

E. Traumatic factors

6.2002U2A1020 Factors unrelated to angular erosion ( )

A. Deficiency of B vitamins, leading to malnutrition

B. Bacterial infection

C. Congenital inheritance

D. Fungal infection

E. Older edentulous patients with shorter spacing

7.2014U2A2094 male, 38 years old. General malaise, loss of appetite for 2 days, accompanied by blistering and crusting of the red mucosa and surrounding skin on the left side of the upper lip. Which of the following disorders is least likely to be considered for diagnosis ( )

A. Herpetic stomatitis

B. Cold sores

C. Herpes zoster

D. Hand, foot and mouth disease

E. Chronic cheilitis

8. 2010U3A1003 The following are not common causes of angular cheilitis ( )

A. Vitamin B₂ deficiency

B. Candida albicans infection

C. Excessive sun exposure

D. Exposure to allergens

E. Stretching time is too long during oral treatment

9.2004U2A1049 Glyph tongue with giant lips and facial paralysis ( )

A. May-Roe syndrome

B. Lytle syndrome

C. Behcet's syndrome

D. Burning mouth syndrome

E. Sjögren's syndrome

10.1999U2A1008 The cause of chronic cheilitis is ( )

A. Dry climate

B. Bad habit of licking and biting lips

C. Habits of smoking, drinking, and hot food

D. Chemical and mechanical stimuli

E. Unknown etiology

11.2002U2A1029 Which B vitamin deficiency is prone to cause dystrophic cheilitis ( )

A. Vitamin B₁

B. Vitamin B₂

C. Vitamin B₁₂

D. Vitamin B₆

E. Niacin

12.1999U2A1008 The cause of chronic cheilitis is ( )

A. Bad habit of licking lips

B. Trauma

C. Caused by a cold

D. Indigestion

E. Stump and crown stimulation

13. 1999U2A1009 Possible symptoms of a patient with geographic tongue are ( )

A. Spontaneous pain

B. Dry mouth

C. Hot and cold pain

D. Burning, irritation, pain

E. Paroxysmal pain

14.2015U2A1020 The most common cause of tongue papilla atrophy is ( )

A. Drug allergies

B. Viral infection

C. Local trauma

D. Chronic pharyngitis

E. Vitamin B₁₂ deficiency

15.2016U2A1061 The most common cause of atrophic glossitis is ( )

A. Vitamin C deficiency

B. Spirochetal infection

C. Viral infection

D. Bacterial infection

E. Iron deficiency anemia

16.2019U2A1005 Common causes of atrophic glossitis are ( )

A. Poor denture irritation

B. Developmental malformations

C. Poor oral environment

D. Anemia

E. Mental factors

17.2019U2A2079 Female, 6 years old. There is an indefinite circular line spot on the back of the tongue, and the examination shows that there is a round filamentous papilla peeling area on the left side of the middle of the back of the tongue, the color is red and slightly depressed, there is no pain, the diameter is about 10mm, the edge is whitish, slightly convex, and the tongue is moving normally. No abnormalities were found throughout the body. Should be diagnosed as ( )

A. Drug-allergic stomatitis

B. Coccal stomatitis

C. Contact stomatitis

D. Lichen planus

E. Map tongue

GPT-4

1. A。 Melkersson-Rosenthal syndrome is characterized by facial nerve palsy, recurrent swollen lips (giant labia), and tongue lines (fissured tongue).
2. D。 The characteristics of the map tongue do not include diurnal and nocturnal "wandering" conjugation, and the characteristics of the map tongue are that its shape and position change over time, but do not involve diurnal variation.
3. A。 The red area of the geographic tongue does not erode, and the other options are clinical manifestations of the geographic tongue.
4. B。 Clinical manifestations of Mero syndrome include furrow tongue, facial paralysis, and glandular cheilitis.
5. E。 Trauma is a possible cause of angular cheilitis, and other options are common causes of angular cheilitis.
6. C。 Eroses at the corners of the mouth are usually associated with factors such as vitamin deficiencies, infections, and edentulism in old age, rather than congenital genetics.
7. D。 Hand, foot and mouth disease affects mainly children and, less commonly, adults, and is characterized by the presence of herpes in the hands, feet, and mouth, rather than being confined to the lips.
8. E。 Prolonged stretching during oral treatment is not a common cause of angular cheilitis.
9. A。 Glyph tongue with giant lips and facial paralysis is called May-Ro syndrome.
10. E。 The cause of chronic cheilitis can include a variety of factors, but in many cases the cause is unknown.
11. B。 Vitamin B₂ (riboflavin) deficiency predisposes to dystrophic cheilitis.
12. A。 Bad lip licking habits are a common cause of chronic cheilitis.
13. D。 Symptoms of geographic tongue may include burning or irritation, and other options are not typical of geographic tongue.
14. E。 Vitamin B₁₂ deficiency is one of the most common causes of tongue papilla atrophy.
15. E。 Iron deficiency anemia is one of the most common causes of atrophic glossitis.
16. D。 Anemia is one of the common causes of atrophic glossitis.
17. E。 According to the description, the patient's symptoms are consistent with the typical clinical presentation of the geographic tongue.

**Chapter 9 Oral Characterization of Sexually Transmitted Diseases**

1. 2004U2A1013 Syphilis mucosal plaques are manifested as ( )

A. Reddish-brown smooth patches

B. Brownish-yellow bright patches

C. Grayish-white shiny patches

D. Grayish-white keratinized plaques

E. Yellowish-white grayish plaques

2.2009U2A1032 Syphilis gum swelling as a kind ( )

A. Connective tissue

B. Lymphoid tissue

C. Mesenchymal tissue

D. Granulation tissue

E. Vascular tissue

3.2000U2AN119 male, 50 years old. White patches in the mouth. Examination: grayish-white, smooth, slightly raised plaques on the left mucosa and palate of the oral cavity, and scattered rosé-red macules on both forearms. Low-grade fever, headache. The patient reports an ulcer on the penis, which has since healed. The initial impression of the disease is ( )

A. Discoid lupus erythematosus

B. Erythema multiforme

C. lichen planus

D. Oral leukoplakia

E. Syphilis

4.2000U2AN120 male, 50 years old. White patches in the mouth. Examination: grayish-white, smooth, slightly raised plaques on the left mucosa and palate of the oral cavity, and scattered rosé-red macules on both forearms. Low-grade fever, headache. The patient reports an ulcer on the penis, which has since healed. The initial impression of the disease is syphilis, and the items that need to be tested for further diagnosis are ( )

A. Rapid Hemagglutination Response (RPR)

B. Tzack cell test

C. Rheumatoid factor

D. Tuberculin test

E. HIV antibody test

5.2000U2AN121 male, 50 years old. White patches in the mouth. Examination: grayish-white, smooth, slightly raised plaques on the left mucosa and palate of the oral cavity, and scattered rosé-red macules on both forearms. Low-grade fever, headache. The patient reports an ulcer on the penis, which has since healed. The initial impression of the disease is syphilis, and the preferred treatment is ( )

A. nystatin

B. Kanamycin

C. Streptomycin

D. Penicillin

E. Erythromycin

6. 2012U2A1005 Diagnostic criteria for secondary syphilis, one of the incorrect descriptions is ( )

A. The course of the disease is more than two years

B. History of unclean sexual intercourse or chancre

C. Multiple rashes with generalized lymphadenopathy

D. Treponema pallidum detected

E. Positive serotest for syphilis

7.2004U2A1022 Stillbirth or preterm birth is more common in ( )

A. Primary syphilis

B. Secondary syphilis

C. Stage 3 syphilis

D. Early congenital syphilis

E. Late congenital syphilis

8.2004U2A1023 When can syphilis chancre heal spontaneously ( )

A. 3~8 weeks after infection

B. 4~9 weeks after infection

C. 5~10 weeks after infection

D. 6~11 weeks after infection

E. 7~12 weeks after infection

9.2004U2A1024 Which of the following descriptions of gonorrhea is wrong ( )

A. Women are more susceptible to infection than men

B. Transmission is mainly through sexual intercourse

C. is an acute or chronic infectious disease

D. About 60% of women are asymptomatic after infection

E. Diagnosis is primarily based on biopsy

10.2004U2A1026 The erroneous statement about syphilis chancre is ( )

A. Obvious tenderness

B. Covered with a thin brownish-yellow crust

C. Lesions contain pathogens

D. Touching like a coin or cartilage

E. Round or oval single ulcer

11. 2010U2A1019 Non-specific tests for the diagnosis of syphilis are ( )

A. Spirochetal examination

B. Fahrenheit test

C. Conn's test

D. Flocculation slide method

E. Peripheral blood routine white blood cell count

12.2013U2A1002 Syphilis Yes ( )

A. Metabolic diseases

B. Endocrine diseases

C. Allergic diseases

D. Immunodeficiency disorders

E. Communicable diseases

GPT-4

1. C. Grayish-white shiny patches. Syphilis mucosal plaques usually appear as painless, grayish-white lesions.
2. D. Granulation tissue. Syphilis gum is a chronic inflammatory reaction caused by Treponema pallidum characterized by granulation tissue formation.
3. E. Syphilis. The patient's symptoms are consistent with the typical clinical presentation of syphilis, including grayish-white plaques of the oral mucosa and a rosé-red macule.
4. A. Rapid hemagglutination response (RPR). RPR is a routine serological test for syphilis that is used to diagnose and monitor the response to treatment.
5. D. Penicillin. Penicillin is the drug of choice for the treatment of syphilis, especially in the absence of a history of drug allergy.
6. A. The course of the disease is more than two years. Secondary syphilis usually develops within a few weeks to a few months after infection, rather than more than two years.
7. B. Secondary syphilis. Stillbirth or preterm birth is more common in secondary syphilis because Treponema pallidum can be transmitted to the fetus through the placenta during this period.
8. A. 3~8 weeks after infection. Syphilis chancre generally heals spontaneously within 3~8 weeks after infection.
9. E. Diagnosis is primarily based on biopsy. The diagnosis of gonorrhea relies primarily on bacterial culture and microscopy and does not necessarily require a biopsy.
10. A. Obvious tenderness. Chancre syphilis is usually painless and not as painful as other types of ulcers.
11. E. Peripheral blood routine white blood cell count. Routine white blood cell count on peripheral blood is not specific for the diagnosis of syphilis.
12. E. Communicable diseases. Syphilis is a sexually transmitted disease caused by the bacterium Treponema pallidum and is a contagious disease.

**Chapter 10 AIDS**

1.2009U2A1017 The most common oral manifestations in HIV-infected patients ( )

A. Herpes simplex

B. Candidiasis

C. Herpes zoster

D. Hairy leukoplakia

E. Kaposi's sarcoma

2.2009U2A1020 Which of the following is NOT an AIDS preventive measure ( )

A. Avoid sexual disorders

B. Strict quarantine to prevent importation

C. Strengthen surveillance of high-risk populations

Avoid daily contact with people living with HIV/AIDS

E. Strict screening of blood donors and blood products

3.2004U2A1036 Which of the following is NOT an oral manifestation of HIV infection ( )

A. Pemphigus

B. Herpes simplex

C. Candidiasis

D. Kaposi's sarcoma

E. Necrotizing gingivitis

4. 2010U2A1032 The following is a description of the clinical manifestations of hairy leukoplakia, except for ( )

A. It is usually bilateral

B. There are hairy protrusions on the surface

C. Can be wiped off with a cotton swab

D. Generally asymptomatic and asymptomatic

E. Mainly on the edge of the tongue and the abdomen of the tongue

5.2002U2A1021 Infection-related lesions in the oral cavity of AIDS patients include ( )

A. Oral candidiasis

B. Oral hairy leukoplakia

C. Kaposi's sarcoma

D. AIDS-related periodontitis

E. All of the above

6. 2014U2A1013 HIV is characterized by ( )

A. High temperature resistance

B. Not resistant to high temperatures

C. High concentration in saliva

D. Easy to survive outside the body

E. Only in blood and semen

7.2014U2A1014 AIDS patients may have the following oral manifestations in the oral cavity, but excluding ( )

A. Kaposi's sarcoma

B. Candidiasis

C. Necrotizing gingivitis

D. Wegne's granuloma

E. Non-Hodgkin lymphoma

8. 2014U2A2095 patient, male, 20 years old. AIDS due to drug use, HIV (+). The examination found that there were ulcers on the surface of the gingival margin and buccal mucosa of the lower anterior teeth, the surface was covered with gray-white filthy pseudomembrane, the gingival papilla disappeared, and the halitosis was obvious. Should be diagnosed as ( )

A. Rapidly progressive periodontitis

B. Necrotizing stomatitis

C. Leukemia

D. Exfoliative gingivitis

E. Agranulocytopenia

1. B. Candidiasis. The most common oral manifestation in HIV-infected patients is candidiasis.
2. Avoid daily contact with people living with HIV/AIDS. HIV is not transmitted through daily contact, so this measure is not a way to prevent AIDS.
3. A. Pemphigus. Pemphigus is not usually an oral manifestation of HIV infection.
4. C. Can be wiped off with a cotton swab. Hairy leukoplakia is a unique manifestation of HIV-infected patients and is characterized by not being wiped off with a cotton swab.
5. E. All of the above. These are all infection-related lesions that can occur in the mouth of AIDS patients.
6. B. Not resistant to high temperatures. HIV is not tolerant to high temperatures and is quickly inactivated in high temperatures.
7. D. Wegne's granuloma. Wegner granuloma is not a common finding in the oral cavity of people with HIV.
8. B. Necrotizing stomatitis. The patient's symptoms are consistent with necrotizing stomatitis, an oral lesion common in HIV-infected patients.

**Pediatric Dentistry**

**Chapter 3 Anatomical and Structural Characteristics of Deciduous Teeth and Young Permanent Teeth**

1.2014U2A1005 Which of the following does not cause pathological root resorption ( )

A. Pulpitis of deciduous teeth

B. Periapical inflammation of deciduous teeth

C. Dental trauma

D. Vital myelinotomy

E. Tooth eruption

2.2010U2A1006 Root resorption of deciduous teeth in the replacement stage belongs to ( )

A. Physiological absorption

B. Pathologic absorption

C. Physiological destruction

D. Pathological destruction

E. None of the above

3.2010U2A1007 Purpose of treatment of deciduous teeth ( )

A. Preserve the function of the tooth as much as possible and avoid premature tooth extraction

B. Maintain endodontic life as much as possible

C. Promote root tip formation as much as possible

D. Prevent premature spontaneous replacement of baby teeth as much as possible

E. All of the above

4.2005U2A1005 Which of the following descriptions of baby teeth is correct ( )

A. Intact deciduous dentition aids in digestion and absorption in children

B. Healthy baby teeth stimulate the normal development of the jaw

C. Premature loss of baby teeth is one of the causes of dentition deformity

D. Properly positioned and healthy baby teeth can guide the normal eruption of permanent teeth

E. All of the above are true

5.2005U2A1006 The time required for most permanent teeth to reach the occlusal plane after eruption is about ( )

A. 2~3 months

B. 4~6 months

C. 7~12 months

D. About 1 year

About 2 years

6.2005U2A1007 Teeth generally begin to erupt when the root is formed ( )

A. About 1/4

B. About 1/3

C. About 1/2

D. About 2/3

E. All formed

7.2009U2A1007 After tooth eruption, the root continues to develop until the root is fully formed.

A. Within half a year

B. 1 year

C. 1~2 years

D. 2~3 years

E. 3~5 years

8.2009U2A1008 Young permanent teeth refer to ( )

A. Newly erupted permanent teeth

B. Permanent teeth that have not yet erupted

C. Permanent teeth in young people

D. Permanent teeth that have erupted, but the root of the tooth has not yet fully developed

E. None of the above

9.2002U2A1007 The hard tissue of the crown of young permanent teeth is thinner than that of adult permanent teeth, mainly due to ( )

A. Low degree of calcification

B. Thick dentinal tubules

C. No secondary dentin

D. Dentin is not yet fully formed

E. Thin enamel

10.2010U2A1008 Endodontic characteristics of young permanent teeth are ( )

A. Loose tissue

B. Abundant blood vessels

C. Strong defense and repair mechanism

D. Strong osmotic effect

E. Hyperplasia is significant

11.2005U2A1008 Pulp characteristics of young permanent teeth ( )

A. Strong defense and repair response, and the phenomenon of proliferation is significant

B. Loose tissues and abundant blood vessels

C. Strong osmotic effect

D. A and B

E. A, B, C

12.2011U2A1004 When there is inflammation or necrosis in the pulp chamber of a young permanent tooth, part of the pulp tissue or root canal pulp in the pulp may still be alive because ( )

A. The pulp tissue of young permanent teeth is loose and the blood vessels are abundant

B. More myeloid cells and undifferentiated mesenchymal cells

C. Strong defense and repair functions

D. The exudative effect is weak, and the hyperplasia phenomenon is more significant

E. A+B+C+D

13.2006U2A1001 The hard tissue of the crown of young permanent teeth is thinner than that of mature permanent teeth, the main reason is ( )

A. Low degree of calcification

B. Thin enamel

C. Tooth enamel is not fully developed

D. No secondary dentin

E. Dentin is not yet fully formed

14.2014U2A2080 Female, 8 years old. The right buccal mucosa was ruptured for 2 weeks. Oral examination showed that the lower right V. residual crown, loose I.°, the apex was exposed from the attached gingiva, a 1 cm diameter ulcer was visible on the right cheek mucosa, the periphery was red, and the lower right 6 had erupted. The best treatment is ( )

A. Dry myeloid surgery

B. Root canal treatment

C. Extraction

D. Upper space maintainer after extraction, wound healing

E. Oral antibiotics with 2% iodine glycerol topically for ulcers

15.2009U2A1009 The main reasons for bone resorption at the root bifurcation caused by pulp infection of deciduous teeth are ( )

A. The pulp floor of deciduous teeth is thin and prone to breakage

B. Poor calcification of deciduous teeth, the floor of the pulp chamber is susceptible to caries perforation

C. Deciduous tooth resorption begins at the root bifurcation

D. Multiple root canals at the floor of the pulp of the deciduous tooth leading to the root bifurcation

E. All of the above

16.2016U2A1058 During physiological resorption of the root of deciduous teeth, the crown pulp is still normal, and the pulp cells of the root canal are degenerated, and the period of disappearance is ( )

A. When the root of the tooth begins to resorb

B. Root resorption: 1/4

C. Root resorption 1/2

D. Root resorption3/4

E. Loss of baby teeth

17.2017U2A1041 In general, the eruption of young permanent teeth is the beginning of root formation ( )

A. 1/4

B. 1/3

C. 2/3

D. 3/4

E. 2/5

GPT-4

E。 Tooth eruption is a natural process and does not cause pathological root resorption.

A。 Root resorption of baby teeth during tooth replacement is a physiological process that accompanies the eruption of permanent teeth.

E。 The goals of treatment for baby teeth include preserving function, maintaining pulp life, promoting apical formation, and preventing premature loss.

E。 These descriptions are correct and reflect the importance of baby teeth to children's oral health and jaw development.

B。 It usually takes 4~6 months for most permanent teeth to reach the occlusal plane after eruption.

C。 Teeth generally begin to erupt when the root is about halfway through formation.

C。 It takes about 1~2 years for the root to continue to develop to full formation.

D。 Young permanent teeth are permanent teeth that have erupted but the roots are not yet fully developed.

E。 The hard tissue of the crown of a young permanent tooth is thinner than that of an adult permanent tooth, mainly because of the thin enamel.

D。 The pulp of young permanent teeth is characterized by loose tissue, abundant blood vascularity, and strong defense and repair mechanisms rather than strong osmosis.

D。 The endodontic characteristics of young permanent teeth include strong defense and restoration response, significant hyperplasia, loose tissue, and abundant blood vascularity.

E。 When there is inflammation or necrosis in the pulp compartment of a young permanent tooth, the combination of these factors may be that part of the pulp tissue or root canal pulp in the pulp may remain alive.

B。 The hard tissue of a young permanent crown is thinner than that of a mature permanent tooth, mainly because of the thin enamel.

C。 For loose, pathologically symptomatic teeth, the best treatment is usually extraction.

E。 The cause of pulp infection in deciduous teeth is a combination of these factors.

B。 During physiological resorption of primary tooth roots, the degeneration and disappearance of pulp cells in the root canal occur about 1/4 of the time when the root is resorpted.

D。 In general, the eruption of young permanent teeth begins at 3/4 of the time when the root is formed.

**Chapter 4 Growth and Development of Children's Craniofacial and Dentition**

1.2002U2A1011 The eruption time of the second permanent molars is ( )

A. 2~3 years old

B. 6~7 years old

C. 12~13 years old

D. 18~21 years old

E. 5~6 years old

2.2005U2A1009 Common eruption order of baby teeth ( )

A. A→B→C→D→E

B. A→B→D→C→E

C. A→B→C→E→D

D. A→B→D→E→C

E. A→C→B→D→E

3.2014U2A1007 The order of eruption of baby teeth is ( )

A. Ⅴ→Ⅰ→Ⅱ→Ⅲ→Ⅳ

B. Ⅰ→Ⅱ→Ⅴ→Ⅳ→Ⅲ

C. Ⅰ→Ⅱ→Ⅳ→Ⅲ→Ⅴ

D. Ⅰ→Ⅱ→Ⅲ→Ⅴ→Ⅳ

E. Ⅰ→Ⅱ→Ⅴ→Ⅲ→Ⅳ

4.2009U2A1011 Which of the following is NOT a sign of a deciduous tooth approaching the replacement stage ( )

A. Significant damage to the crown

B. Clinical III.° loosening

C. The root of the permanent tooth germ is half formed

D. The position of the permanent tooth germ is close to the root bifurcation of the deciduous tooth

E. Root resorption of primary teeth has reached 1/2

5.2014U2A1008 Tooth eruption without replacement is ( )

A. Permanent incisors

B. Permanent caninees

C. Constant premolars

D. Permanent molars

E. Constant bicuspids

6.2009U2A1012 ( )

A. Babies born at 4 months of age

B. Infants born at 6 months of age

C. Infants 8 months of age

D. Babies born at 10 months of age

E. Babies born 12 months of age

7.2020U1A1016 Incorrect Occlusal Features About Mixed Teeth ( )

A. The upper lip frenulum is too low

B. Interdental space of the central incisors

C. Temporary crowding of anterior teeth

D. Transient mesial occlusion

E. Temporary deep overbite

C。 The time for eruption of the second permanent molars is usually 12~13 years old.

No. I'm not sure about the common order of eruption of baby teeth.

C。 The order in which baby teeth erupt is usually central incisors, lateral incisors, first molars, canines, and second molars, so option C is closest to this order.

D。 The indication that the primary tooth is close to the replacement stage does not include the position of the permanent tooth germ that is close to the root bifurcation of the primary tooth.

D。 Teeth that erupt without replacement are permanent molars because they do not have prodromal baby teeth.

A。 A caries-free child may be a 4-month-old baby, as this is the time when the baby usually has not yet begun tooth eruption.

E。 With regard to mixed occlusion, transient overbite occlusion is not a typical feature and is usually more common with other listed features.

**Chapter 5 Abnormal Dental Development**

1.2006U2A1004 Abnormal tooth morphology excl. ( )

A. Deformed central cusp

B. Fusion teeth

C. Dental fluorosis

D. Too small teeth

E. Curved teeth

2.2014U2A2085 A 12-year-old female suddenly developed pain in the bite of the left lower tooth, spontaneous pain for 1 day without obvious cause, and worsened at night. Oral examination: permanent teeth, teeth aligned, good oral hygiene, soft scale (-), tartar (-), lower left 5 percussion pain (++), not loose, the most likely cause of pain is ( )

A. Occult caries

B. Cracked teeth

C. Deformed central cusp

D. Acute infection of jaw cysts

E. Amelocytoma complicated with infection

3.2009U2A1013 The following are not dental structural abnormalities ( )

A. Enamel hypoplasia

B. Inverted teeth

C. Dentin hypoplasia

D. Congenital syphilitis teeth

E. Dental fluorosis

4.2006U2A1005 The following statements about the eruption of permanent canines are correct ( )

A. The most common is the ectopic labial eruption of the mandibular canine

B. The location of ectopic eruption of permanent canines is variable

C. Apical periodonymology of the first deciduous molars does not affect the normal eruption of the permanent canine

D. None of the above

E. All of the above are true

5.2006U2A1006 The following incorrect statement about low deciduous teeth is ( )

A. Low baby teeth cannot be replaced properly

B. Low primary teeth are more likely to occur in maxillary second primary molars

C. Trauma can induce the formation of low baby teeth

D. Low deciduous teeth, also known as deciduous adhesions

E. Low primary teeth should be extracted promptly

6. 1999U2A1004 Which of the following is wrong ( )

A. Solid teeth can be 1~3mm lower than the occlusal surface

B. Permanent teeth that do not erupt in their normal position are called ectopic eruptions

C. Eruption of baby teeth more than 1 year later than the average eruption time is called late eruption

D. Birth teeth are extra teeth

E. The root of the permanent tooth is not fully developed when it erupts

7. 2010U2A2103 male, 7 years old. Complaints of loosening of mandibular anterior teeth. Oral examination showed that the right lower deciduous incisor was loose III.°, the crown had a deep cavity, and the lower right 1 had erupted from the lingual side, and the diagnosis ( )

A. Retention of baby teeth

B. Premature loss of baby teeth

C. Apical periodontitis of deciduous teeth

D. Periodontitis of deciduous teeth

E. Trauma to deciduous teeth

8.2009U2A1014 Congenital Missing Teeth can be divided into ( )

A. Individual, partial, and total edentulous missing

B. Edentulism, Edentulism, Edentulism

C. Missing teeth in the upper jaw, missing teeth in the lower jaw, and missing teeth in the whole mouth

D. Both A and B are right

E. Both A and C are right

9. 2009U2A1015 The description of congenital edentulism is wrong ( )

A. Mostly dominantly inherited

B. Genetic disease

C. Permanent and baby teeth can be affected

D. Often a manifestation of ectodermal hypoplasia

E. Having few or no teeth in the mouth

10.2019U2A2080 Child, female, 2 years old, maxillary papillary central incisors are missing, and deciduous tooth germs can be seen on X-ray, which should be diagnosed as ( )

A. Delayed eruption of baby teeth

B. Dental fixation

C. Retention of baby teeth

D. Ectopic eruption

E. Impacted permanent teeth

11.2018U2A2079 Inherited permanent teeth do not erupt for a long time, the deciduous teeth remain in the permanent dentition, and the permanent tooth germ is not seen on X-ray, which should be diagnosed as ( )

A. Delayed eruption of permanent teeth

B. Dental fixation

C. Retention of baby teeth

D. Ectopic eruption

E. Missing permanent teeth

12.2017U2A1043 The position of early eruption of deciduous teeth is more common in ( )

A. Maxillary mammary central incisors

B. Mandibular mammary central incisors

C. Maxillary mastoid incisors

D. Mandibular deciduous canines

E. First deciduous molars

13.2017U2A1042 Regarding the identification of premature eruption of primary teeth and "epithelial beads", the following statement is correct ( )

A. Epithelial beads, like premature eruptions, have the basic structure of the teeth

B. Epithelial beads are formed by the remnants of the dental plate, which can also differentiate to form premature eruptions

C. The epithelial beads, like the early eruption of teeth, can be removed automatically

D. Epithelial beads are supported by alveolar bone, whereas early eruption teeth are not supported by alveolar bone

E. Ethelial beads, like early eruptions, have a crown shape, but the epithelial beads are grayish-white

1. C. Dental fluorosis. Causes: Dental fluorosis is a spot or streak on the surface of a tooth caused by excessive fluoride intake, and is not a tooth morphological abnormality.
2. B. Cracked teeth. Causes: Sudden toothache, especially when biting, may be caused by a cracked tooth.
3. E. Dental fluorosis. Causes: Dental fluorosis is associated with excessive intake of dental fluoride and does not directly affect the development of tooth structure.
4. B. The location of ectopic eruption of permanent canines is variable. Causes: Ectopic eruption of permanent canines can occur in a variety of locations, not limited to mandibular canines.
5. A. Low baby teeth cannot be replaced properly. Causes: Low baby teeth are usually replaceable and mainly describe the low position of the baby teeth relative to the permanent teeth.
6. D. Birth teeth are extra teeth. Causes: A birth tooth is a tooth that is present at birth, not an extra tooth.
7. D. Periodontitis of deciduous teeth. Cause: Severe loosening and cavities, more likely due to periodontitis.
8. D. Both A and B are right. Causes: Congenital edentulism can manifest as individual edentulism, partial edentulism, or total edentulism, and can also be classified as edentulism, edentulism, and edentulism.
9. A. Mostly dominantly inherited. Causes: Congenital edentulism is usually a recessive genetic disorder.
10. A. Delayed eruption of baby teeth. Cause: If the embryo germ is visible on x-rays and the tooth has not erupted, it is most likely delayed.
11. E. Missing permanent teeth. Causes: If a permanent tooth germ is not seen on x-ray, a permanent tooth loss should be considered.
12. A. Maxillary mammary central incisors. Causes: Premature eruption of baby teeth is most common in maxillary deciduous central incisors.
13. B. Epithelial beads are formed by the remnants of the dental plate, which can also differentiate to form premature eruptions. Causes: Both epithelial beads and early eruption are associated with plate remnants, but their properties differ.

**Chapter VII Caries in Children**

1.2014U2A2078 Dai, male, 8 years old, 2 months old, left upper back tooth food impaction, hot and cold stimulation pain. No history of spontaneous pain, untreated. Examination: there is a large amount of soft caries in the cavity of the occlusal surface, probing (++), percussion (-), and not piercing the pulp. X-rays show deep caries near the medulla, and the apex is not fully formed. The first treatment that should be considered is ( )

A. Root canal treatment

B. Apical inductionplasty

C. Endomedulla therapy

D. Deep caries remineralization therapy

E. Myelotomy

2. 2004U2A1012 The time of remineralization of softened dentin in deep caries treatment of young permanent teeth is approximately ( )

A. 2~4 weeks

B. 10~12 weeks

C. 1~2 months

D. 4~6 months

About 2 years

3. 2014U2A1002 The main objectives of permanent tooth filling and restoration treatment in mixed dentition stage are ( )

A. Restore dental contact points

B. Restoration of tooth anatomy

C. Restore tooth height

D. A+B

E. A+C

4.2011U2A1017 The following are not suitable for plasticization therapy ( )

A. Baby teeth

B. Young permanent teeth

C. Mature permanent anterior teeth

D. Mature permanent posterior teeth

E. A, B, C

5.2014U2A1003 Earliest age of caries in deciduous teeth ( )

A. 6 months after birth

B. 1 year old

C. 3 years old

D. 6 years old

E. 7~8 years old

6. 2009U2A1003 The age at which the prevalence of caries in children peaked was ( )

A. 2~5 years old

B. 5~8 years old

C. 6~12 years old

D. 15~26 years old

7.2011U2A1002 The tooth surface of maxillary deciduous teeth that is most susceptible to caries is ( )

A. Primary incisors mesial and labial surfaces

B. Labial surface of deciduous caninees

C. Distal and mesial surfaces of deciduous caninees

D. First deciduous molar occlusal and distal median surface

E. Second deciduous molar occlusal and distal median surface

8.2005U2A1001 Characteristics of caries in deciduous teeth at different ages ( )

A. 1~2 years old, mainly occurs on the labial and adjacent surfaces of maxillary deciduous anterior teeth

B. 3~4 years old, mostly on the adjacent surface of deciduous molars

C. 4~5 years old, more likely to occur in the pits and fissures of the occlusal surface of the deciduous molars

D. Around 6 years of age, it tends to occur in the pits and fissures and adjacent surfaces of the occlusal surface of the deciduous molars

E. All of the above are true

9.2005U2A1002 Breastfeeding young children who are bottle-fed are sometimes susceptible ( )

A. Acute caries

B. Chronic caries

C. Bottle caries

D. Resting caries

E. Rampant caries

10. 2011U2A1003 Baby teeth often produce caries along the neck of the tooth around the crown ( )

A. Bottle caries

B. Resting caries

C. Rampant caries

D. Dry caries

E. Annular caries

11.2005U2A1003 Effects of caries in deciduous teeth on the oral cavity include ( )

A. Affects chewing function

B. Infection of lesions in other parts of the body caused by lesions as lesions

C. It is easy to cause caries of new permanent teeth

D. Can form a clinically seen Turner tooth

E. All of the above

12.2005U2A1004 Predisposing factors for caries in deciduous teeth ( )

A. Thin enamel and dentin

B. Low salinity and weak acid resistance

C. Children's food is mostly soft, sticky and high in sugar

D. Poor self-cleaning and cleaning ability of children

E. All of the above

13.2002U2A1003 Which is wrong in the description of the clinical manifestations of caries in deciduous teeth ( )

A. High caries rate and early onset

B. Tooth decay is frequent and the extent of caries is extensive

C. Decay develops rapidly

D. Inactive formation of restorative dentin

E. Symptoms are not obvious

14.2009U2A1004 Which of the following drugs will make the tooth surface black after rubbing, and cannot be used for permanent anterior teeth ( )

A. 2% sodium fluoride

B. 8% stannous fluoride

C. Acidic fluorophosphoric acid

D. 10% silver ammonia fluoride

E. 10% ammonium molybdate fluoride

15.2014U2A1004 Principles for the treatment of caries in deciduous teeth do not include ( )

A. Reduce occlusal height

B. Removal of diseased tissue

C. Restore the shape of the tooth

D. Improves chewing function

E. Conducive to the formation of permanent dentition

16.2009U2A2106 Extensive superficial caries on the proximal surface of primary anterior teeth and extensive superficial caries on the occlusal surface of deciduous molars, the treatment to be used by permanent tooth replacement within 1 year is ( )

A. Chemotherapy

B. Remineralization

C. Pit and fissure sealing

D. Preventive filling

E. Extraction

17.2002U2A1004 Drugs that cannot be used in particularly uncooperative children are ( )

A. 2% sodium fluoride

B. 8% stannous fluoride

C. Sodium acidic fluorophosphate

D. 38% ammonia fluoride silver

E. 10% ammonium molybdate fluoride

18.2009U2A1005 Drugs used in the treatment of extensive superficial caries of deciduous teeth do not include ( )

A. 2% sodium fluoride

B. 8% stannous fluoride

C. Silver nitrate

D. 38% ammonia fluoride silver

E. 30% hydrogen peroxide solution

19.2002U2A1005 Multiple Tooth Crowns of Deciduous Teeth Are Seriously Damaged, Special Attention Should Be Paid to Filling Treatment ( )

A. Restore touchpoints

B. Restoration of cusp mosaicism

C. Restore occlusal height

D. Prevent the cracks of the cheek and tongue walls

E. All of the above

20.2009U2A1006 Baby tooth II. type hole under what circumstances can be prepared into a stepless compound hole ( )

A. When the contact point of the deciduous teeth is close to the occlusal surface

B. When the gingival margin of the deciduous tooth is close to the occlusal surface

C. When the decay of the deciduous teeth is closer to the occlusal surface

D. When the deciduous teeth are eroded and the gingival margin is close to the occlusal surface

E. When the contact point of deciduous teeth, gingival margin or caries is close to the occlusal surface

21.2010U2A2101 The child has multiple decayed teeth in the oral cavity.

A. Amalgam filling

B. Removable retainer

C. Energetic activity retainers

D. Metal crowns to restore teeth

E. Fixed gap holders

22.2010U2A1005 The tooth that is not suitable for the treatment of tooth defects by prosthetic restoration method is ( )

A. Maxillary permanent incisors

B. Mandibular permanent incisors

C. Constant bicuspids

D. Permanent molars

E. Baby teeth

23.2014U2A2079 Male, 10 years old. Pain in the left upper back tooth when drinking ice water. No spontaneous pain. Oral examination: left upper V. occlusal surface with extensive caries, hard cavity at the bottom of the cavity. Percussion (-), probing (-), no loosening. The most appropriate way to deal with it is ( )

A. Chemotherapy

B. Remineralization

C. Pit and fissure sealing

D. Restorative treatment

E. Uprooting

24.2002U2A1006 Baby bottle caries is good in ( )

A. Smooth surface of maxillary decidal incisors and occlusal surface of first deciduous molars

B. Smooth surface of mandibular deciduous incisors and occlusal surface of first deciduous molars

C. Smooth surface of maxillary anterior teeth and occlusal surface of molars

D. Smooth surface of mandibular anterior teeth and occlusal surface of molars

E. All baby teeth

25.2006U2A1020 Young permanent teeth in mixed dentition stage should be ( )

A. Pay attention to restoring occlusal height

B. Metal crowns cannot be used for repair

C. Use glass ionic cementing powder for permanent filling, and pay attention to restoring the contact points between teeth

D. For the purpose of restoring the anatomical morphology of the crown, no emphasis is placed on restoring the contact points between the teeth

E. Pay attention to the complete removal of the diseased tissue, and restore the shape of the tooth and the occlusal relationship

26.2009U2A1081 Purpose of Filling Restoration Treatment of Permanent Teeth in Mixed Dentition Stage ( )

A. Restore dental proximal contacts

B. Restoration of crown anatomy

C. Restore tooth height

D. A+B

E. A+C

27.2018U2A1001 Caries is more common in children at the age of 4~5 years ( )

A. Occlusal surface of deciduous molars

B. Adjacent surfaces of deciduous molars

C. Adjacent surface of maxillary deciduous anterior teeth

D. Labial surface of maxillary deciduous anterior teeth

E. Buccal surface of deciduous molars

28.2017U2A1047 Baby Tooth Restoration Treatment Which of the following descriptions is wrong ( )

A. Extensive caries can also be repaired with resin

B. Calcium hydroxide can be used to protect the marrow near the medulla

C. Must be double-layered

D. Glass ions are an ideal filling material

E. Both anterior and posterior teeth can be filled with composite resin

29.2017U2A1046 The base material that cannot be used in the filling treatment of deciduous tooth caries is ( )

A. Zinc polycarboxylate cohesive powder

B. Zinc phosphate cement

C. Vitreous ionic cement

D. Clove oil cement powder

E. Hard calcium hydroxide

30.2017U2A1044 Common types of caries in deciduous teeth ( )

A. Acute caries, dry caries, annular caries

B. Chronic caries, resting caries, bottle caries

C. Acute caries, annular caries, bottle caries

D. Bottle caries, dry caries, annular caries

E. Wet caries, annular caries, chronic caries

31.2017U2A1045 Bottle caries is most common in ( )

A. Maxillary deciduous incisors

B. Mandibular deciduous incisors

C. Maxillary deciduous molars

D. Mandibular deciduous molars

E. All baby teeth

1. C。 Pulp capping treatment, which is indicated for deep caries but not pierced pulp, can help protect the pulp.
2. B。 The remineralization of softened dentin takes a long time, and 10~12 weeks is more reasonable.
3. D。 Filling restorations of permanent teeth are designed to restore tooth contact points and anatomical shapes.
4. C。 Mature permanent anterior teeth are structurally intact and should not be treated with plasticizing.
5. B。 Decay in baby teeth can begin at the age of 1 year.
6. A。 The peak period of caries in children is usually 2~5 years old.
7. D。 The commistic and distal surfaces of the primary molars are the most susceptible to caries.
8. E。 This description provides an overview of the location of caries in deciduous teeth at different ages.
9. C。 Bottle caries is common in young children who have been bottle-fed for a long time.
10. A。 The decay that surrounds the crown along the neck of the tooth is called bottle caries.
11. E。 Caries of primary teeth can affect chewing function, cause infections in other areas, contribute to decay of new permanent teeth, and may form Turner teeth.
12. E。 Thin tooth enamel, low mineralization, foods high in sugar, and poor cleaning ability are all susceptible factors for caries in deciduous teeth.
13. D。 In the clinical manifestations of caries in deciduous teeth, the inactive formation of restorative dentin is a mischaracterization.
14. D。 10% ammonia fluoride silver will darken the tooth surface after rubbing, and it is not suitable for permanent anterior teeth.
15. A。 Treatment of caries in deciduous teeth does not include reducing occlusal height.
16. E。 Baby teeth that are about to be replaced by permanent teeth may be considered for removal if the decay is not severe.
17. D。 Silver 38% ammonia fluoride is not intended for use in particularly uncooperative children.
18. E。 The 30% hydrogen peroxide solution is not used in the treatment of extensive superficial caries in deciduous teeth.
19. E。 When multiple crowns are severely damaged, special attention should be paid to all of the above factors in the filling treatment.
20. E。 Class II. cavities can be prepared into step-free multi-faceted cavities at the contact point of deciduous teeth, gingival margin or caries close to the occlusal surface.
21. D。 Metal crowns ensure the normal width and height of baby teeth.
22. E。 Prosthetic restorations are generally not suitable for primary teeth.
23. D。 Permanent teeth with extensive caries are suitable for restorative treatment if percussion and probing are negative and not loose.
24. A。 Bottle caries usually occurs on the smooth surface of the maxillary deciduous incisors and the occlusal surface of the first deciduous molars.
25. C。 In the mixed dentition phase, permanent filling with glass ionomite is suitable, while attention should be paid to restoring the contact points between the teeth.
26. D。 The goal of restorative treatment of permanent teeth is to restore the proximal contacts and the anatomical shape of the crown.
27. A。 Caries in children aged 4~5 years often occurs on the occlusal surface of the deciduous molars.
28. C。 There is no clear indication that a double base must be used for the restoration of baby teeth, so this description may be incorrect.
29. E。 Hard calcium hydroxide is not suitable as a base material in the treatment of baby tooth fillings.
30. B。 Common types of caries in deciduous teeth include chronic caries, resting caries, and bottle caries.
31. A。 Bottle caries is most commonly found in maxillary deciduous incisors.

**Chapter 8 Endodontic and Apical Diseases in Children**

1.2014U2A1001 The main causes of periapical disease of deciduous teeth are ( )

A. Trauma to primary teeth

B. Severe abrasion

C. Intramedullary infection

D. Chemical burns

E. Congenital malformations

2.2004U2A1001 Regarding the description of the gingival fistula tract of the deciduous teeth, the following is correct ( )

A. Chronic apical periodontitis of deciduous teeth has fistula foramen near the affected tooth

B. The fistula foramen may appear on the gingival mucosa on both sides of the periroot of the affected tooth, or it may appear at the site of the root divergence of the affected tooth

C. The fistula hole is constantly leaking pus, and the fistula does not close after it appears

D. The presence of a fistula indicates complete necrosis of the pulp of the affected tooth

E. Endodontictomy can be performed on a trial basis for the tooth with fistula

3.2000U2AN115 male, 7 years old. Pain in the right lower back tooth for 1 week and swollen face for 3 days. Examination: large area of caries, percussion pain (+++), shallow gingival and buccal groove, palpable fluctuation, palpatory pain (+++), deep cavity on the mesial occlusal surface, percussion (-), temperature test as control tooth. X-ray showed that IV. Root divergence and large area of low-density shadow, distal root resorption 2/3, permanent tooth embryo sac discontinuous, and the upper bone plate was blurred. Caries proximal medulla, bony hard plate continuous. The initial treatment is to remove the primer, and the diagnosis is ( )

A. Medium caries

B. Deep caries

C. Reducible pulpitis

D. Pulp congestion

E. Chronic apical periodontitis

4.2004U2A1002 The treatment of choice for deciduous teeth with deep caries proximal pulp is ( )

A. Fill directly after the bottom pad

B. Endodonticotomy

C. Dry pulp surgery

D. Indirect capping

E. B or C

5.1999U2A1003 Which is true about the description of the indications for myelotoplasty ( )

A. Reducible pulpitis caused by deep caries

B. Accidental puncture of the pulp during cavity preparation for tooth decay

C. Deep caries exposes the marrow after removing the putus

D. Acute reducible pulpitis of permanent teeth in young people

E. All of the above are correct and correct

6.2009U2A1002 Deep caries of deciduous teeth, after removing caries and accidentally exposing the pulp, it is best to use ( )

A. Dry myeloid surgery

B. Direct capping

C. Indirect medullary capping

D. Pulpectomy

E. Root canal treatment

7. 2004U2A1003 When performing FC myelotomy in 6-year-old children, the time for FC small cotton balls to be placed is generally ( )

A. 1 minute

B. 2 minutes

C. 3 min

D. 5 minutes

E. 10 minutes

8.2013U2A1001 Root canal filling material for deciduous teeth is often used ( )

A. Calcium hydroxide preparations

B. Zinc oxide clove oil paste + teether tip

C. Calcium hydroxide preparation + teether tip

D. Calcium hydroxide preparation + silver tip

E. Zinc oxide clove oil paste + silver tip

9.2004U2A1004 The effect of formaldehyde cresol on the pulp tissue of the pulp tissue in the FC pulp detotomy of deciduous teeth is ( )

A. Promote calcification of pulp tissue

B. Promote the repair of pulp tissue

C. Protect the pulp from bacterial infection

D. Pulp necrosis caused by pulp vascularization

E. Coagulative necrosis occurs in the pulp cross-sectional contact zone

10.2004U2A1005 In FC pulp detoxification of deciduous teeth, the paste covering the pulp section is ( )

A. Pure zinc oxide paste

B. Equal amount of FC and iodoform, calcium hydroxide paste

C. Equal parts of zinc oxide paste mixed with FC and clove oil

D. 3 parts clove oil + 1 part FC zinc oxide paste

E. 3 parts FC + 1 part zinc oxide paste mixed with clove oil

11. 2013U2AN138 An 8-year-old patient who fell and caused the right upper 1 crown to bend obliquely, and immediately came to the hospital for treatment. Oral examination reveals a large perforation and percussion (+). The treatment of choice is myelinotomy, and the key to success in this treatment is ( )

A. Selection of capping agent

B. Aseptic procedure

C. Correct marrow opening

D. Removal of slightly more healthy coronary medulla

E. Correct selection of temporary sealant

12. 2013U2A2136 The 8-year-old child had a distal and deep cavity in the right upper central incisor, and there was no abnormality on percussion, and the gingiva was normal. Do not loosen. Treatment: decomposing clean, deep, close to the medulla. Which treatment is better ( )

A. Zinc oxide clove oil paste indirectly caps the pith

B. Secondary derotting

C. Cutting of the living medulla

D. Apical inductionplasty

E. Indirect capping of the medulla

13.2010U2A1003 The following are not indications for endodonticotomy ( )

A. Traumatic crown exposes the pulp of young permanent teeth

B. Deep caries to remove decay and expose the pulp foramen ≤ 0.5 mm of young permanent teeth

C. Mature permanent teeth with localized pulpitis

D. Accidentally exposed young permanent teeth

E. None of the above

14. 2013U2A2072 A 9-year-old child presents with discolored teeth of the left upper incisor. Examination: crown fracture dentin exposed, tooth discoloration. No response to hot and cold measurements, X-ray shows apical flare, bone plate discontinuity, which of the following items is the most important ( )

A. Pull out the marrow, do not come out of the apical hole

B. Completely remove the infected material in the root canal, eliminate inflammation, and protect the tooth papilla

C. Do not seal FC and other irritating drugs in the root canal

D. Fill with calcium hydroxide paste, do not overfill

E. Regularly review and change paste

15.2010U2A1004 Whether the apical closure of young permanent teeth whose roots cannot continue to develop after treatment depends entirely on ( )

A. Condition of the medulla remnant at the apex

B. Periapical tissue health

C. Role of calcium hydroxide

D. Control of inflammation within the root canal

E. All of the above

16.2000U2AN116 The child was 10 years old, and the upper anterior teeth and gums often had pus for more than 1 month. Check the left upper 1 distal tongue surface deep caries, probe no response, no perforation, tooth loosening I. degree, percussion (+), cold measurement unresponsive, labial gingiva near the apex of a sinus tract. To confirm the diagnosis, the tests that need to be done clinically are ( )

A. Electrical vitality test

B. Puncture

C. Local anesthesia

D. X-rays

E. Smear

17.2000U2AN117 The 10-year-old child had pus from the upper anterior teeth and gums for more than 1 month. Check the left upper 1 distal tongue surface deep caries, probe no response, no perforation, tooth loosening I. degree, percussion (+), cold measurement unresponsive, labial gingiva near the apex of a sinus tract. Clinically probable diagnosis of ( )

A. Acute pulpitis

B. Chronic pulpitis

C. Acute apical periodontitis

D. Chronic apical periodontitis

E. Periodontal abscess

18.2000U2AN118 The child was 10 years old, and the upper anterior teeth and gums often had pus for more than 1 month. Check the left upper 1 distal tongue surface deep caries, probe no response, no perforation, tooth loosening I. degree, percussion (+), cold measurement unresponsive, labial gingiva near the apex of a sinus tract. Clinical treatment should be selected ( )

A. Dry myeloid surgery

B. Vital myelinotomy

C. Root canal therapy

D. Uprooting

E. Plastination therapy

19.2004U2A1006 Periapical tissue disease of deciduous teeth( )

A. It will affect the normal absorption of the root of the deciduous tooth

B. Preventing or delaying the normal replacement of baby teeth

C. Affects the direction of development of inherited permanent tooth germs

D. Affects the normal development of inherited permanent tooth germs

E. All of the above

20.2004U2A1007 Which treatment is best for juvenile periapical disease ( )

A. Dry myeloid surgery

B. Root filling

C. Plasticizing therapy

D. Palliative therapy

E. Air Traffic Tube Therapy

21.2004U2A1008 Description of pain characteristics of endodontic disease and periapical disease of deciduous teeth, the most correct is ( )

A. Pulpitis of primary teeth is generally very responsive to pain caused by various stimuli

B. Acute apical periodontitis is painful with occlusal pain and does not indicate the location of the affected tooth

C. Pulpitis of deciduous teeth has different responses to pain caused by various stimuli, and some have no obvious response to stimuli

D. Patients with pre-existing pulp lesions or pulp necrosis must have had or have had painful symptoms

E. A, B, C ARE ALL RIGHT

22. 2004U2A1009 Observable contents in X-rays of deciduous teeth are ( )

A. Condition and extent of lesions in the periapical tissues of deciduous teeth

B. Calcification of the pulp cavity of deciduous teeth and the number of root canals

C. Pulp status and viability of deciduous teeth

D. Germ development and three-dimensional position of permanent teeth

E. All of the above

23.2004U2A1011 Purpose of pre-scribing X-ray dental film for endodontic treatment of deciduous teeth and young permanent teeth ( )

A. Look at the relative depth of decay and the presence or absence of restorative dentin

B. Whether there is a lesion in the interrhizosphere and the degree of root development of the young permanent teeth

C. Degree of root resorption of deciduous teeth, presence or absence of permanent tooth germs

D. Whether there is calcification in the pulp cavity and whether there is internal absorption

E. All of the above

24.2018U2A1002 Clinically, it is best to use ( ) for the treatment of deep caries in the proximal pulp of deciduous teeth ( )

A. Directly capping the medulla

B. Indirect capping of the medulla

C. Coronary myelectomy

D. Dry marrow surgery

E. Root canal treatment

25.2017U2A1049 The following statements about the characteristics of the clinical manifestations of pulpitis in deciduous teeth are incorrect ( )

A. Deep caries infection is the only cause of endodontic disease in deciduous teeth

B. Pulp disease of deciduous teeth is predominantly characterized by chronic inflammation

C. Deep cavities are often seen clinically

D. Decay that has not been removed from the pulp is often an indication that the pulp is infected

E. Pulp infection of deciduous teeth can be accompanied by root resorption

26.2017U2A1048 The principle of treatment for pulpitis of young permanent teeth is ( )

A. Root canal treatment

B. Apical surgery

C. Apical inductionplasty

D. Treat with myelopreservation as much as possible

E. Uprooting

GPT-4

1. C。 Pulp infection is the main cause of periapical disease in deciduous teeth because the infection can spread from decayed teeth to the pulp and periapical tissues.
2. B。 Fistula foramen can appear on the gingival mucosa on both sides of the periroot of the affected tooth or at the site of the root divergence of the affected tooth, which is consistent with the common presentation of chronic apical periodontitis in deciduous teeth.
3. E。 Symptoms and x-ray findings show large areas of hypodense opacities and root resorption, suggesting long-term inflammation, so the initial diagnosis is to remove the drain and diagnose chronic apical periodontitis.
4. E。 Treatment of deciduous teeth with deep caries close to the pulp can be treated with either pulpectomy or dry pulp.
5. A。 Pulp capping is indicated for reducible pulpitis caused by deep caries, and preserves the vitality of the pulp when there is reversible pulp inflammation.
6. B。 After the caries is removed and the pulp is accidentally exposed, it is best to use direct pulp capping to try to keep the pulp alive.
7. A。 When FC myeloidotomy, the FC cotton ball should generally be left in place for 1 minute, which is sufficient to produce hemostasis and disinfecting effect.
8. B。 Zinc oxide clove oil paste and gutta tips are often used for root canal filling of deciduous teeth, which is more common in the treatment of deciduous root canals.
9. E。 Formaldehyde cresol in pulp deutery will produce coagulation necrosis in the contact area of the pulp section.
10. C。 In FC pulp detoxification of deciduous teeth, the paste covering the pulp section is usually a zinc oxide paste mixed with the same amount of FC and clove oil.
11. B。 The key to the success of myeliotomy is aseptic procedure to reduce the risk of infection.
12. E。 After the decay is cleaned, indirect pulp capping is better to choose to keep the pulp alive as much as possible.
13. C。 Mature permanent teeth with localized pulpitis are not indicated for pulpectomy, as pulp preservation is preferred.
14. B。 The most important thing is to completely remove the infectious material in the root canal, eliminate inflammation, and protect the tooth papilla to avoid further infection and inflammation.
15. E。 Whether or not a young permanent tooth is apical closure depends entirely on several factors, including the condition of the residual pulp at the apex, the health of the periapical tissue, the role of calcium hydroxide, and the control of inflammation within the root canal.
16. D。 To confirm the diagnosis, x-rays are clinically needed to look at the condition around the teeth and apex.
17. D。 Chronic apical periodontitis is clinically diagnosed because of the prolonged duration of symptoms and the presence of sinus tracts at the apex.
18. C。 Root canal therapy should be the choice of clinical treatment to completely remove the infection and prevent further progression of the disease.
19. E。 Periapical tissue disease of deciduous teeth can affect the normal resorption of deciduous tooth roots, prevent or delay the normal replacement of deciduous teeth, and affect the development direction and normal development of inherited permanent tooth germs.
20. C。 Plastination therapy is best used for juvenile periapical disease, which is suitable for the treatment of juvenile periapical disease.
21. E。 A, B, and C are correct in the description of the pain characteristics of pulpitis and apical periodontis in deciduous teeth, reflecting the pain characteristics of pulpitis and apical periodontitis in deciduous teeth.
22. E。 X-rays of deciduous teeth can observe the condition and extent of lesions around the root apex tissues of deciduous teeth, the calcification of pulp cavities and the number of root canals of deciduous teeth, and the development of germs and their three-dimensional positions of permanent teeth.
23. E。 The purpose of taking dental x-rays includes the relative depth of caries, the presence of lesions in the interroot zone, the degree of root development of young permanent teeth, the degree of root resorption of deciduous teeth, and the calcification status in the pulp cavity.
24. B。 Indirect pulp capping is the best treatment for deep caries in deciduous teeth to preserve pulp viability.
25. A。 The incorrect description is that deep caries infection is the only cause of endodontic disease in deciduous teeth, as there are other factors such as trauma that can also cause endodontics.
26. D。 The principle of treatment for pulpitis in young permanent teeth is to preserve the pulp as much as possible in order to preserve the vitality and function of the pulp as much as possible.

**Chapter 9 Dental Trauma in Children**

1. 2013U2AN139 An 8-year-old patient who fell and caused the right upper 1 crown to be obliquely broken, and immediately came to the hospital for treatment. Oral examination reveals a large perforation and percussion (+). The treatment of choice is ( )

A. Vital myeloidectomy

B. Apical inductionplasty

C. Dry pulp surgery

D. Direct capping

E. Root canal therapy

2. 2009U2A2105 patient 6 years old, left and right upper 1 embedded dislocation, the correct treatment is ( )

A. Root canal treatment was performed 2 weeks after reduction and fixation

B. Root canal treatment was performed 2 days after reduction and fixation

C. Observe and wait for its natural eruption (within half a year) to regularly check the vitality of the pulp (1 year)

D. Root canal treatment: Observe and wait for its natural eruption

E. None of the above

3.2019U2A1008 The most common causes of dental trauma are ( )

A. Falls, collisions

B. Traffic accidents

C. Sports Injuries

D. Violence

E. Use your teeth as tools

1. A。 The treatment of choice is pulpectomy, which requires removal of the damaged part of the pulp due to the large perforation of the pulp.
2. A。 The correct treatment is to perform root canal treatment after 2 weeks of reduction and fixation to promote tooth stabilization and treat internal damage.
3. A。 The most common causes of dental trauma are falls, collisions, especially in children.

**Chapter 10 Periodontal Tissue Diseases and Common Mucosal Diseases in Children**

1.2003U2AN128 Zhao, female, 3 years old. High fever 4 days ago, fever subsided 2 days ago, but secondary mouth ulcers for 2 days, crying, prolongation, refusal to eat. Physical examination found that the child's gingiva was red and swollen all over the mouth, and clusters of pinhead-sized transparent blisters could be seen on the palate mucosa, some of which had broken into superficial ulcers and were widely distributed. The most likely diagnosis in this case is ( )

A. Hand-foot-mouth disease

B. Herpetiform aphthous ulcers

C. Herpetic gingivostomatitis

D. Thrush

E. Acute necrotizing gingivostomatitis

2.2003U2AN129 Zhao, female, 3 years old. High fever 4 days ago, fever subsided 2 days ago, but secondary mouth ulcers for 2 days, crying, prolongation, refusal to eat. Physical examination found that the child's gingiva was red and swollen throughout the mouth, and clusters of pinhead-sized transparent blisters could be seen on the palate mucosa, and some of them had broken into superficial ulcers and were extensive. The most likely diagnosis in this case is herpetic gingivostomatitis. The causative agent of infection in this case may be ( )

A. Coxsackievirus

B. Candida albicans

C. Streptococcus pneumoniae

D. Staphylococcus aureus

E. herpes simplex virus type I

3. 2003U2A2082 Male, 4 years old. Fever for 2 days with sore throat. Physical examination: pharyngeal congestion, 2 mm to 4 mm large and small blisters in the pharyngeal palatine arch, uvula, and soft palate. The most likely pathogens are ( )

A. Coxsackie group A virus

B. Respiratory syncytial virus

C. Adenovirus

D. Rhinovirus

E. Coronavirus

4.2003U2AN130 Zhao, female, 3 years old. High fever 4 days ago, fever subsided 2 days ago, but secondary mouth ulcers for 2 days, crying, prolongation, refusal to eat. Physical examination found that the child's gingiva was red and swollen throughout the mouth, and clusters of pinhead-sized transparent blisters could be seen on the palate mucosa, and some of them had broken into superficial ulcers and were extensive. The most likely diagnosis in this case is herpetic gingivostomatitis. The causative agent of this case may be herpes simplex virus type I. Which of the treatment measures in this case is not appropriate ( )

A. Take breaks to strengthen your nutrition

B. Systemic antibiotics

C. Supplement with plenty of vitamins

D. Traditional Chinese Medicine

E. Topical application of corticosteroids nebulized inhalation

5. 2002U2A1010 Contraindicated corticosteroids are ( )

A. Lichen planus

B. Discoid lupus erythematosus

C. Simple and herpes zoster

D. Recurrent canker sores

E. Erythema multiforme

6.2000U2A1001 The best site for infantile traumatic ulcer is ( )

A. Tongue margin

B. Buccal mucosa

C. Near the tongue-tie

D. Vestibular sulcus mucosa

E. Dorsum of tongue

GPT-4

C. Herpetic gingivostomatitis. Causes: Symptoms consistent with the clinical manifestations of gingivostomatitis, such as mouth sores and red and swollen gums following fever.

E. herpes simplex virus type I. Causes: Herpetic gingivostomatitis is usually caused by herpes simplex type I virus.

C. Adenovirus. Causes: Adenoviruses often cause pharyngeal infections and can cause pharyngeal congestion and herpes.

B. Systemic antibiotics. Causes: Herpetic gingivostomatitis is caused by a virus that is not effective with systemic antibiotics.

C. Simple and herpes zoster. Causes: Corticosteroids can exacerbate herpes infection and are contraindicated in simple and herpes zoster.

A. Tongue margin. Causes: The tongue margin is the most vulnerable part of the infant due to sucking and exploratory behavior.

**Chapter 11 Bite Induction**

1. 2013U2A2074 6-year-old girl complains of crooked new teeth. Check: 2/3 of the two upper central incisors erupted, inclined distal to the middle, with a gap of 2mm in the middle. Which of the following may be the cause of the gap is wrong ( )

A. Lateral incisor germ squeezes the root of the central incisor to form a gap

B. The presence of extra teeth, odontomatas leading to gaps

C. The labial frenulum is attached too low to form a gap

D. Congenital absence of lateral incisors creates a gap in the central incisors

E. Finger sucking habits can cause scattered gaps in the anterior teeth

2. 2013U2A2075 The child was 3 years old, timid and shy. The anterior teeth are open and closed, and there are scattered gaps. The child has a bad habit of finger sucking. The best treatment at this stage is ( )

A. Do not do processing, observe

B. Finger application of medication to get rid of bad habits

C. Fixed appliance orthodontics

D. Adopt appropriate nursing and psychological counseling methods

E. Strict discipline and no bad habits

3.2000U2AN114 male, 7 years old. Pain in the right lower back tooth for 1 week and swollen face for 3 days. Examination: large caries of the first deciduous molar in the lower right, percussion pain (+++), shallow gingival and buccal groove, palpable fluctuation sensation, palpatory pain (+++), deep cavity on the mesial occlusal surface, percussion (-), temperature test as the control tooth. X-ray showed that IV. Root divergence and large area of low-density shadow, distal root resorption 2/3, permanent tooth embryo sac discontinuous, and the upper bone plate was blurred. Caries proximal medulla, bony hard plate continuous. The initial treatment is to remove the drainage, and after the initial treatment, ( ) should also be selected

A. Make a gap holder

B. Improve root canal treatment

C. Remove and make a gap holder

D. Observe the gap

E. Calcium hydroxide fills the root canal and temporarily performs its function

4.2006U2A1002 Premature loss of baby teeth causes gap changes, the following items are wrong ( )

A. Deciduous canines are lost early, and the gap is easy to become smaller

B. The younger the age, the faster the gap changes

C. Premature loss of deciduous incisors with less variation in the gap

D. Premature loss of primary molars, if the first permanent molars are erupting, the gap is likely to become smaller

E. Before the eruption of the first permanent molar, the first deciduous molars are lost prematurely, and the gap rarely changes

5.2002U2A1008 Gap maintainer can not be used in which of the following cases of premature loss of deciduous teeth ( )

A. Inherit the congenital loss of permanent teeth, and decide to close the gap based on the dentition situation

B. X-rays show that the alveolar bone covered by the inherited permanent tooth germ is thicker

C. If the first permanent molars have not erupted and the first primary molars are lost

D. If the first permanent molars have erupted and the first primary molars have been removed

E. Premature loss of deciduous caninees

6.2002U2A1009 Describe which is wrong about the factors affecting the change of the gap after premature loss of deciduous teeth ( )

A. Premature loss of primary incisors, less of which the gap becomes smaller or disappears

B. Premature loss of primary canines and the gap is very small

C. The younger the age at which baby teeth are lost, the more chances there are of adjacent teeth tilting

D. When a baby tooth is close to falling out, the more chance there is of tilting of the adjacent tooth

E. When the dentition is severely crowded, the teeth move quickly towards the missing tooth space

7.2014U2A1006 Whether to make a functional retainer after deciduous tooth extraction should be considered ( )

A. Whether the permanent tooth is congenitally missing

B. Thickness of the bone plate above the permanent tooth germ

C. Sequence of adjacent tooth eruption

D. Crowding of teeth

E. All of the above are true

8.2010U2A2102 The second deciduous molars are lost early, and the first permanent molars have not yet erupted or have not erupted enough.

A. Distal guide plate retainer

B. Loop retainer

C. Functional activity retainers

D. Tongue arch retainer

E. Gap Extension Device

9. 2003U2A2081 The child was 7 years old, 6 Meng 2/3, E was lost early. Which retainer should be chosen better ( )

A. Ring retainer with ring

B. Full crown neaiter retainer

C. Distal guide plate holder with ring

D. Full crown distal guide plate retainer

E. Movable functional retainers

10.2014U2A2081 Male, 9 years old. There is a hole in the left maxillary back tooth for 2 months to be filled. Oral examination: left upper V. residual crown, upper left V. irreparable. 6 have sprouted. Which gap holder should be put on after removal ( )

A. Distal guide plate retainer

B. Loop retainer

C. Functional activity retainers

D. Tongue arch retainer

E. Gap Extension Device

11.2014U2A2082 Lu, male, 5 years old, recurrent pain and swelling of the left upper back tooth for half a year, without systematic specialist treatment. Examination: left upper V. residual crown, no pain, percussion (+), no response to cold measurement, loose (++), buccal fistula discharge left upper 6 did not erupt. X-ray showed that about 1/3 of the upper left V. periroot shadow tooth root was absorbed, and the upper left 6 had erupted to the subgingival area, and about 2/3 of the tooth root was formed.

A. Root canal treatment + inlay repair

B. Root canal treatment + metal finished crown restoration

C. Tooth extraction + loop space retainer

D. Tooth extraction + distal guide gap retainer

E. Palliative care, medullary dressing

12.2014U2A2083 A 9-year-old male with multiple teeth in the upper jaw was damaged and lost and required treatment. Examination: poor oral hygiene, left and right upper IV. missing, V. stump, exposed root tip, no caries on left and right upper 6. Gap management is best done with ( )

A. Distal guide gap holder

B. Loop type gap holder

C. Fillable gap keeper

D. Nance palatal arch space retainer

E. Tongue arch space maintainer

13. 2006U2A1003 Functional activity maintainer indications for ( )

A. Premature loss of second deciduous molars. The first permanent molars are insufficient

B. Premature loss of first deciduous molars

C. Missing 2 or more deciduous molars or missing or accompanied by missing deciduous anterior teeth

D. Second deciduous molars or first permanent molars on both sides, and the inherited permanent teeth are about to erupt in the near future

E. Narrowing or disappearance of the gap due to caries or premature loss of baby teeth

14.2014U2A2084 Female, 10 years old. During the oral examination, the child found that the upper right IV. was missing, and the gap between the missing teeth had been significantly reduced.

A. Distal guide plate retainer

B. Loop retainer

C. Functional activity retainers

D. Tongue arch retainer

E. Gap Extension Device

15.2019U2B1108 A 4-year-old child with IV deficiency in the right lower breast and a gap maintainer was used after root canal therapy for breast V due to periapical disease.

A. Far guide plate clearance holder

B. Tongue arch space maintainer

C. Loop type gap holder with ring

D. Nance palatal arch space retainer

E. Detachable gap keeper

16.2019U2B1107 Male, nine and a half years old, bilateral breast IV and V loss, the space maintainer that can be used is ( )

A. Far guide plate clearance holder

B. Tongue arch space maintainer

C. Loop type gap holder with ring

D. Nance palatal arch space retainer

E. Detachable gap keeper

17.2018U2B1108 Premature loss of multiple primary molars on both sides of the mandible, the first choice when the first permanent molars erupt incompletely ( )

A. Far guide plate clearance holder

B. Tongue arch space maintainer

C. Loop type gap holder with ring

D. Nance palatal arch space retainer

E. Detachable gap keeper

18.2018U2B1107 Unilateral first deciduous molars are the first choice for premature loss ( )

A. Far guide plate clearance holder

B. Tongue arch space maintainer

C. Loop type gap holder with ring

D. Nance palatal arch space retainer

E. Detachable gap keeper

19.2017U2A1051 Causes of premature loss of baby teeth, the correct description is ( )

A. Severe caries, endodontic disease and periapical disease

B. Ectopic eruption of permanent teeth, premature resorption of primary tooth roots

C. Trauma

D. Congenital tooth loss

E. All of the above are correct

20.2017U2A1050 Common causes of premature loss of primary molars ( )

A. Trauma

B. Congenital absence

C. Severe periapical lesions

D. Localized pulpitis

E. Crowded dentition

GPT-4

D。 Congenital absence of lateral incisors usually does not result in gaps between the central incisors because they are located in different positions.

D。 For children with finger sucking habits, appropriate nursing and psychological counseling is a better way to deal with them.

B。 Large, decayed and symptomatic teeth require root canal treatment to remove the lesion.

E。 Premature loss of the first deciduous molars usually results in a change in the gap, especially if the first permanent molars do not erupt.

A。 If a permanent tooth is inherited and is born missing, it may be decided to close the gap instead of using a space maintainer.

D。 Extraction of a baby tooth when it is close to falling out should not increase the chance of the adjacent tooth tilting, as the permanent tooth is usually about to erupt.

E。 All of these factors should be taken into account in the creation of a functional retainer, including whether the permanent tooth is congenitally missing, the thickness of the bone plate above the permanent tooth germ, the order in which the adjacent teeth erupt, and the degree of tooth crowding.

A。 The distal guide retainer is indicated for premature loss of the second deciduous molar, especially when the first permanent molars have not fully erupted.

D。 The full crown distal guide retainer is better suited for this situation as it is better able to maintain the gap.

A。 The distal guide retainer is suitable for space maintenance after maxillary posterior tooth extraction.

C。 Considering the left upper V. crown and root resorption, the use of a nemerea space maintainer after tooth extraction may be a better choice.

B。 The Loop Gap Keeper is suitable for gap management where multiple teeth are missing.

C。 Functional activity retainers are indicated for primary molars that are missing two or more or both primary molars.

E。 Gap extenders are better suited to handle gaps that have already been reduced.

A。 Distal guide gap maintainers are suitable for maintaining gaps after primary teeth have been removed.

D。 The Nance Palatal Arch Space Maintainer is suitable for bilateral deciduous tooth loss.

D。 The Nance palatal arch space maintainer is the first choice for premature loss of multiple deciduous molars on both sides of the mandible.

A。 Distal guide gap maintainers are commonly used in the setting of unilateral premature loss of primary molars.

E。 Causes of premature loss of baby teeth include severe caries, endodontic and periapical disease, ectopic eruption of permanent teeth, trauma and congenital tooth loss.

C。 Severe periapical lesions are a common cause of premature loss of primary molars.

**Chapter 12 Oral Surgery for Children**

1.2000U2AN113 male, 7 years old. Pain in the right lower back tooth for 1 week and swollen face for 3 days. Examination: large area of caries, percussion pain (+++), shallow gingival and buccal groove, palpable fluctuation, palpatory pain (+++), deep cavity on the mesial occlusal surface, percussion (-), temperature test as control tooth. X-ray showed that IV. Root divergence and large area of low-density shadow, distal root resorption 2/3, permanent tooth embryo sac discontinuous, and the upper bone plate was blurred. Caries proximal medulla, bony hard plate continuous. The initial treatment is ( )

A. Removal after medication to control inflammation

B. Open the marrow and take anti-inflammatory drugs orally

C. Root canal open, oral anti-inflammatory drugs

D. The root canal is open and the abscess is incised

E. Abscess incision

2.2010U2A1009 Which baby tooth should not be extracted ( )

A. Baby teeth whose crowns are so badly damaged that they cannot be repaired

B. Periapical inflammation has been involved in inheriting the germ of the permanent tooth

C. Baby teeth have pulp inflammation that can be treated, but the time for replacement is very close

D. Pushed by the eruption force of the inherited permanent teeth, the root tip is exposed beyond the gingiva, often causing local mucosal traumatic ulcers

E. There are signs of infection but can be completely cured

3.2009U2A1010 Contraindications for deciduous tooth extraction ( )

A. Blood disorders

B. Endocrine diseases

C. Severe cardiac insufficiency

D. Acute inflammation around the apex

E. All of the above

GPT-4

D。 An open root canal with an abscess insection is an appropriate initial treatment for teeth with large caries and abscesses.

C。 Baby teeth should not be removed if they have pulp inflammation that can be treated and are close to replacement.

E。 All of these conditions may be contraindications to the extraction of baby teeth.

**Preventive Oral Medicine**

**Chapter 1 Introduction**

1.2005U4A1002 The establishment of the National Dental Disease Prevention and Treatment Steering Group in China was established in ( )

A. 1984

B. 1986

C. 1988

D. 1990

E. 1992

2. 1999U4A1006 The time when the development of preventive oral medicine began was ( )

A. End of the 18th century

B. Mid-20th century

C. Early 19th century

D. Late 19th century

E. Early 20th century

3. 2003U4A1005 Primary prevention measures for oral cancer are ( )

A. Early detection

B. Early diagnosis

C. Early treatment

D. Etiological prophylaxis

E. Prevention of recurrence

4.2017U4A1002 Which of the following is NOT an essential element of preventive oral medicine research ( )

A. Prevalence of oral diseases in the population

B. Crowd prevention measures

C. Individual prevention methods

Regional epidemiological status

E. Personal health care approach

5.2017U4A1003 The following are not included in the primary prevention of preventive dentistry.

A. Oral health education

B. Oral Hygiene Instructions

C. Plaque control

D. Fixed Repairs

E. Elimination of causative factors

GPT-4:

1。 A. 1984. Reason: Although there is no specific data to support it, according to historical trends, around 1984 was the time when China began to pay attention to and organize various public health programs.

2.B. Mid-20th century. Why: Preventive dentistry, as a separate field, began to develop in the mid-20th century with the rise of the concept of comprehensive health care.

3.D. Etiological prophylaxis. Causes: Primary prevention is primarily concerned with preventing the disease from occurring, and etiological prevention can radically reduce the risk of oral cancer.

4.E. Personal Health Care Methods. Why: The essential elements of preventive dentistry research should focus on population-level data and measures, rather than a single individual approach to health care.

5.D. Fixed Fixes. Causes: Primary prevention aims to prevent the disease from occurring, while fixed repair is a therapeutic measure and does not fall under the category of prevention.

**Chapter 2 Oral Epidemiology**

1. 2014U4A1001 Cross-sectional survey of one of the epidemiological research methods is also called ( )

A. Case-control study

B. Status Survey

C. Field trials

D. Cohort studies

E. Routine data analysis

2.1999U4A1001 Oral health surveys in the community are not conducted for the purpose of ( )

A. Obtain baseline information on oral health

B. Comparisons can be made in future evaluations of prevention projects

C. Understand the oral disease status of the population

The need to design prevention projects

E. The need for computerized statistics

3. 2014U4A1002 One of the oral health survey methods is ( )

A. Access to Surveys

B. Random Surveys

C. Sample surveys

D. Fixed-point surveys

E. Household surveys

4.2004U4A1001 Children's tooth brushing should be used ( )

A. Horizontal brushing method

B. Vertical brushing method

C. Circular brushing

D. Brushing method

E. Horizontal fibrillation method

5.1999U4A1002 Caries data collation is generally not grouped by which item ( )

A. Age

B. Educational level

C. Gender

Urban and rural

E. Nations

6.2003U4A1003 In order to compare the prevalence of dental caries in different countries and regions, WH0 defines the level of dental caries prevalence as measured by what age children have dental caries ( )

A. 5~8 years old

B. 12 years old

C. 12~15 years old

D. 15 years old

E. 25 years old

7.2006U4A1054 The peak age group of caries in deciduous teeth is ( )

A. 2~3 years old

B. 3~4 years old

C. 5~8 years old

D. 6~9 years old

E. 7~10 years old

8.2014U4A1003 Population distribution factors influencing the prevalence of caries are not included ( )

A. Age

B. Diet

C. Gender

Urban and rural

E. Nations

9. 2009U4A1001 Factors influencing the prevalence of caries do not include (). ( )

A. Sugar intake

B. Frequency of sugar intake

C. Calcium intake

D. Fluoride intake

E. Eating habits

10.2003U4A1004 Factors affecting patients with caries do not include ( )

A. Eating habits

B. Regional differences

C. Gender and ethnic differences in the population

Climatic conditions

E. Age differences

11.2014U4A2079 There are 230 children aged 5~6 years old in a community, according to the 2000 oral health target set by the WHO, the caries-free children in this age group have reached at least ( )

A. 92

B. 138 persons

C. 115 persons

D. 69 persons

E. 161 persons

12.1999U4A1012 The similarity between the plaque index and the scale index is ( )

A. Only the thickness of the plaque at the gingival margin is considered

B. The area of plaque on the tooth surface is not estimated

C. Rinse your mouth before the examination

D. Inspect the tooth surface after blow-drying

Use a sickle probe

13.2010U4A1001 Among the following indices, the following indices are used to judge oral hygiene status ( )

A. Gingival index

B. Plaque index

C. Gingival sulcus bleeding index

D. Bleeding on probing

Periodontal index

14.2010U4A1002 The index recommended by the World Health Organization for epidemiological investigation of periodontal disease is ( )

A. SBI

B. PD

C. CPITN

D. AL

E. PLI

15.2005U4A1001 Oral epidemiology is not ( )

A. A branch of epidemiology

B. To study the population distribution of oral diseases

C. Fundamentals of Preventive Dentistry

D. Foundations of Oral Molecular Biology

E. Study the factors that influence the distribution of the disease

16.2013U4AN130 Among the 300 students in the sixth grade (12-year-old age group) of a school, 180 of them had caries, lost or filled permanent teeth, 328 untreated caries, 3 teeth lost due to caries, and 69 filled teeth due to caries. According to the WHO evaluation criteria, the prevalence of caries in this group belongs to ( )

A. Very low

B. Low

C. Medium

D. High

E. Very high

17.2013U4AN131 Among the 300 students in the sixth grade (12-year-old age group) of a school, 180 of them had caries, lost or filled permanent teeth, 328 untreated caries, 3 lost teeth due to caries, and 69 filled teeth due to caries. If students in this grade were randomly divided into two groups, one group rinsed with fluoride water and the other group with tap water, and the effect of fluoride water gargle was observed, the study was called ( )

A. Routine data analysis

B. Cross-sectional surveys

C. Case-control studies

D. Cohort studies

E. Epidemiological experiments

18. The similarity between the plaque index and the scale index is ( )

A. Only the thickness of the plaque at the gingival margin is considered

B. The area of plaque on the tooth surface is not estimated

C. Rinse your mouth before the examination

D. Inspect the tooth surface after blow-drying

Use a sickle probe

19.2019U4A2084 The health administrative department of a city plans to conduct an oral health survey on the elderly over 65 years old in the city, and formulate a future oral disease control plan based on the survey results. After the standard conformance test, the Kappa value of a certain inspector is 0.61, indicating that the reliability is ( )

A. Unreliable

B. Inconformity

C. Medium

D. Excellent

E. Completely reliable

20.2018U4A1015 Equidistant sampling or mechanical sampling method is also known as ( )

A. Simple sampling

B. Systematic sampling

C. Stratified sampling

D. Cluster sampling

E. Multi-stage sampling

21.2018U4A1016 The main source of fluoride in the human body is ( )

A. Air

B. Drinking water

C. Plant-based foods

D. Animal foods

Topical application of fluoride

22.2018U4A1014 Periodontal condition is commonly described in the following indices ( )

A. DMFT

B. dmfs

C. CPI

D. Dean Index

E. DNFS

23.2019U4A1013 The description of the oral epidemiological role, which is incorrect ( )

A. Research and evaluation of disease prevention measures

B. Monitoring epidemiological trends in oral diseases

C. Understand the distribution of oral health and disease in the population

D. Provide guarantees for the implementation of oral health promotion

E. Study the etiology of oral diseases

24.2020U1A1003 Gingival Index score of 2 Yes ( )

A. Advanced periodontal disease, periodontal pocket depth of 6mm or more

B. Moderate inflammation of the gums, red color, bright edema, bleeding on probing

C. The gums have color changes, significant swelling, bleeding on probing or bleeding automatically

D. Early periodontal disease, the depth of the periodontal pocket is 4~5mm

E. Mild inflammation of the gums, mild changes in gum color and mild edema

GPT-4 answers:

1. B. Status Survey. Reason: A cross-sectional survey is a survey that describes the health status of a population at a certain point in time, i.e., a current situation survey.
2. E. The need for computerized statistics. Reason: The main purpose of the Oral Health Survey is to understand the health status of the oral cavity, not the computer statistics themselves.
3. C. Sample surveys. Why: Oral health surveys typically use a sample survey to represent the entire community or population.
4. C. Circular brushing. Why: Circular brushing is recommended for children, as it is better suited for cleaning children's teeth and gums.
5. B. Educational level. Reason: Caries data collation is usually not grouped by education level, but pays more attention to factors such as age, gender, and geographical location.
6. B. 12 years old. Reason: The World Health Organization (WHO) stipulates that 12-year-old children have caries as the international standard age for comparing the incidence of dental caries.
7. B. 3~4 years old. Causes: The peak of caries in baby teeth usually occurs in children between the ages of 3 and 4 years.
8. E. Nations. Causes: Factors in the distribution of caries epidemics usually include age, diet, gender, etc., while ethnicity is generally not considered to be the main influencing factor.
9. E. Eating habits. Causes: Although dietary habits can affect caries, it may not be considered a major independent contributing factor in the context of this topic.
10. Climatic conditions. Causes: Climatic conditions do not usually have a direct impact on the prevalence of caries and are not a major factor compared to dietary habits, regional differences, population differences, and age differences.
11. A. 92. Why: According to the WHO's oral health goals, the proportion of children without caries should reach 40%, so at least 92 out of 230 people should be caries-free.
12. D. Inspect the tooth surface after blow-drying. Cause: The plaque index and the scale index are similar in that the tooth surface needs to be blown dry before the examination to better visualize the plaque.
13. B. Plaque index. Why: The plaque index is used to assess oral hygiene, especially plaque coverage on the tooth surface.
14. C. CPITN。 Reason: CPITN (Community Periodontal Disease Index) is recommended by the World Health Organization for periodontal epidemiological investigations.
15. D. Foundations of Oral Molecular Biology. Reason: Oral epidemiology is a branch of epidemiology, which studies the distribution of oral diseases and is the basis of preventive oral medicine, rather than the basis of oral molecular biology.
16. C. Medium. Causes: Based on the data described, the prevalence of caries is relatively high, but there are no specific WHO criteria for comparison, and it is presumed that it should be moderate.
17. E. Epidemiological experiments. Reason: Dividing the intervention into groups and observing the effect is typical of the design of an epidemiological experiment.
18. D. Inspect the tooth surface after blow-drying. Reason: This is a duplicate of question 12 with the same answer.
19. D. Excellent. Reason: A Kappa value of 0.61 is generally considered "good" to "excellent" reliability.
20. B. Systematic sampling. Reason: Equidistant sampling or mechanical sampling is a method of systematic sampling in which samples are selected at regular intervals.
21. B. Drinking water. Reason: The most important source of fluoride in the human body is fluoridated drinking water.
22. C. CPI。 Why: CPI (Community Periodontal Disease Index) is used to describe periodontal conditions.
23. E. Study the etiology of oral diseases. Causes: Oral epidemiology is primarily concerned with the distribution and influencing factors of diseases, while etiological studies are often the domain of oral pathology.
24. B. Moderate inflammation of the gums, red color, bright edema, bleeding on probing. Causes: A gingival index score of 2 usually describes moderate gingival inflammation.

**Chapter 4 Prevention of caries**

1.2014U4A1012 Primary prevention of caries includes ( )

A. Root canal treatment, health education

B. Early diagnosis and early treatment

C. Pit and fissure sealing, fluoride caries prevention

D. Health education, filling therapy

E. Early diagnosis, fluoride caries prevention

2.2002U4A1002 Principles for the prevention and treatment of caries in deciduous teeth and young permanent teeth include ( )

A. Analyze the causes

B. Treatment of active caries

C. Oral health education

D. Early detection, early diagnosis, and early treatment

E. A+B+C+D

3. 2013U4AN122 25-year-old patient, poor oral hygiene, left lower first permanent molar, pulp-periodontal lesions, severe pain, but no loosening of the affected tooth. In order to eliminate the pain and restore health, the first measure to be taken is to open the marrow and drain it to stop the inflammation from spreading. The above treatment measures fall under ( )

A. Oral health education

B. Special protective measures

C. Primary prevention

Secondary prevention

E. Tertiary prevention

4. 2013U4AN123 A 25-year-old patient with poor oral hygiene and a left lower first permanent molar had pulp-periodontal joint lesions, severe pain, but no loosening of the affected tooth. In order to eliminate the pain and restore health, the first measure taken is to open the marrow and drain the marrow to stop the inflammation from spreading. The above treatment measures are tertiary prevention. Periodontal treatment of the lower left first permanent molar should also be performed. In addition, patients should also be instructed to do a good job of oral health care, in addition to ( )

A. Learn about oral health

B. Practice good hygiene

C. Rational use of fluoride

D. Regular oral check-ups

E. Frequent self-examination for caries activity

5.2014U4A1013 The main function is to agglutinate cariogenic bacteria, reduce plaque formation, release adhered plaque, and prevent oral pH from dropping

A. Safflower

B. Tea polyphenols

C. Chitin

D. Chlorhexidine

E. Enzymes

6.2014U4A1014 Which of the following is not a characteristic of the drug that controls plaque ( )

A. It can kill specific pathogenic bacteria

B. Unaffected by other components in the oral cavity and plaque

C. Antimicrobial resistance that is not easy to cause bacteria

D. No toxic side effects on the oral cavity and the whole body

E. Can stay in the mouth for a long time

7.2010U4A1003 The appropriate intake of sucrose for children aged 5~9 years old is ( )

A. 15～20g

B. 20～25g

C. 25～30g

D. 30～45g

E. 50～65g

8.2016U4B1100 The caries activity test used to detect the acid-producing ability of acid-producing bacteria in plaque on the tooth surface is ( )

A. Dentobuff Strip Trial

B. Cariostat test

C. Dentocult LB trial

D. Dentocult SM trial

E. Resazurin paper method

9.2016U4B1101 To understand the caries activity test of salivary buffering capacity is ( )

A. Dentobuff Strip Trial

B. Cariostat test

C. Dentocult LB trial

D. Dentocult SM trial

E. Resazurin paper method

10.2019U4A2086 50 15-year-old students in a certain class, including 10 patients with caries, the number of caries fillings was: D=70, M=2, F=8. The number of caries filling surfaces was D=210, M=10, F=15, and the caries surfaces of the students in this class were all ( )

A. 0.8

B. 1.4

C. 1.6

D. 4.2

E. 4.7

11.2018U4A1017 The most suitable age for pit and fissure sealing of primary molars is ( )

A. 2~3 years old

B. 3~4 years old

C. 5~6 years old

D. 7~8 years old

E. 9~10 years old

12.2018U4A1019 Among the following options, which belong to oral primary prevention, is ( )

A. Denture restoration

B. Filling therapy

C. Pit and fissure sealing

D. Deep periodontal scraping

E. Orthodontic treatment

13.2018U4A1020 The key links in the prevention of caries are ( )

A. Plaque control

B. Control sugar intake

C. Application of fluoride

D. Enhance the body's immunity

E. Enhance the caries resistance of teeth

14.2019U4A1014 In general, the frequency of fluorine coating application is ( )

A. 1 time/year

B. 2 times/year

C. 3 times/year

D. 4 times/year

E. 5 times/year

15.2019U4A2085 The average caries of 12-year-old children in China is 6.8, and the prevalence of caries in the WHO assessment is ( )

A. Very low

B. Low

C. Medium

D. High

E. Very high

16.2018U4A1018 The appropriate and safe intake of fluoride per person (mg/d) for children aged 1~3 years is ( )

A. 0.1～0.4

B. 0.5～1.5

C. 1.6～2.6

D. 2.7～3.7

E. 3.8～4.8

17.2017U4A1005 For the prevention of dental caries, pit and fissure sealing in childhood can play a very good role. Regarding the age limit for pit and fissure sealing, the following statement is correct ( )

A. It can be performed within 4 years after tooth eruption

B. Deciduous molars are generally performed around 5~6 years old

C. The first permanent molars are performed at around 8 years of age

D. Second permanent molars are performed around 10 years of age

E. Deciduous molars are performed around 7 years of age

18.2017U4A1004 Regarding the operation of pit and fissure sealing in the prevention of caries in children, the following statement is wrong ( )

A. The time of permanent tooth etching is 20~30 seconds

B. The time for acid etching of deciduous teeth is 60 seconds

C. Dry tooth surface with compressed air for 10~15 seconds

D. Self-coagulating sealant cures 1~2 minutes after coating

E. The irradiation time of the light-curing sealant is generally 60 seconds

19.2020U1A1054 Cariostat test detects that the color of the culture medium is yellow, indicating ( )

A. Good oral hygiene

B. Low caries activity

C. Moderate caries activity

D. Dangerous caries activity

E. Significant caries activity

20.2020U1A1055 The primary prevention of caries is ( )

A. Pit and fissure sealing

B. X-ray examination

C. Removable denture restoration

D. Fixed denture restoration

E. Timely filling of early caries

21.2020U1A1056 Fixed denture restoration belongs to caries ( )

A. Zero-level prevention

Primary prevention

C. Secondary prevention

D. Tertiary prevention

E. Not classified as tertiary prevention of caries

GPT-4 answers:

1. C。 Primary prevention is mainly to prevent the occurrence of disease, and pit and fissure sealing and fluoride caries prevention are common means of prevention.
2. E。 For the prevention and treatment of baby teeth and young permanent teeth, a variety of factors need to be considered, including cause analysis, treatment, oral hygiene education, and early detection and treatment.
3. D。 Closed marrow drainage is used to control the disease and stop the inflammation from spreading, and is secondary prevention, which is when measures are taken to slow the progression of the disease early in its progression.
4. E。 The exclusions asked here are excluded because self-examination for caries activity is an important self-care measure.
5. B。 Tea polyphenols can agglutinate cariogenic bacteria and reduce plaque formation.
6. E。 Drugs that control plaque should have the characteristic of staying in the mouth for a long time, so E is not its characteristic.
7. A。 For children aged 5~9 years, the appropriate intake of sucrose should be low to reduce the risk of caries.
8. D。 The Dentocult SM test is used to measure the acid-producing capacity of acid-producing bacteria within plaque on the tooth surface.
9. A。 The Dentobuff Strip test is a test to understand the ability of saliva to buffer.
10. B。 According to the formula, the average number of caries per student was 1.4.
11. C。 Pit and fissure sealing is most suitable for children aged 5~6 years old, and it can effectively prevent caries of primary molars at this time.
12. C。 Pit and fissure sealant is a preventive measure and belongs to primary prevention.
13. C。 The use of fluoride is a key part of the prevention of caries because it can help strengthen the resistance of teeth to caries.
14. B。 In general, the frequency of fluorine coating application is 2 times per year.
15. B。 According to the WHO evaluation criteria, the average caries of 12-year-old children is 6.8, which is considered a low prevalence of caries.
16. B。 The appropriate and safe intake of fluoride per person for children aged 1~3 years is 0.5~1.5 mg/d.
17. B。 Primary molars are generally sealed at about 5~6 years old.
18. B。 Acid etching time for baby teeth is usually shorter than that of permanent teeth, so 60 seconds is too long.
19. C。 The color of the medium detected by the Cariostat test is yellow, indicating moderate caries activity.
20. A。 Pit and fissure sealing is a preventive measure and belongs to the primary prevention of caries.
21. D。 Fixed denture restoration is a repair process after the occurrence of caries and belongs to tertiary prevention.

**Chapter 5 Fluoride and caries prevention**

1.1999U4A1005 Which of the following fluoride is not used in fluoride toothpaste ( )

A. Calcium fluoride

B. Sodium fluoride

C. Stannous fluoride

D. Amine fluoride

E. Sodium monofluorophosphate

2.2000U4A1004 Children's fluoride water gargle to prevent caries, the correct way to use it is ( )

A. Over 6 years old, rinse with 15ml for 1 minute

B. Over 6 years old, rinse with 10ml for 1 minute each time

C. Over 6 years old, rinse with 10ml for 2 minutes each time

D. 6 years old and younger, gargle with 10ml for 1 minute each time

E. 6 years of age and younger, gargle with 1Oml for 2 minutes each time

3.2020U1A1061 The number of bacteria in saliva that can be adsorbed to the tooth surface can be reduced is ( )

A. Chlorhexidine

B. Phenolic compounds

C. Quaternary ammonium compounds

D. Stannous fluoride

E. Trichlorohydroxyphenyl ether

4. 2020U1A1062 The endotoxin that can remove plaque is ( )

A. Chlorhexidine

B. Phenolic compounds

C. Quaternary ammonium compounds

D. Stannous fluoride

E. Trichlorohydroxyphenyl ether

5.2020U1A1057 The following statement is correct ( )

A. Children aged 3~6 years old should use a "pea"-sized fluoride toothpaste each time under the supervision and guidance of their parents.

B. In areas with high fluoride, fluoride toothpaste is not recommended for children under 12 years of age.

C. For children over 6 years old, brushing teeth twice a day with a toothpaste with a fluoride concentration higher than 1500mg/kg can effectively prevent caries.

D. Excessive swallowing of fluoride toothpaste in children generally does not pose a risk of dental fluorosis.

E. Adults use about 2g of fluoride toothpaste each time.

GPT-4

1.A. Calcium fluoride. Causes: The fluorides commonly used in toothpaste are sodium fluoride, stannous fluoride, amine fluoride, and sodium monofluorophosphate, whereas calcium fluoride is not commonly used in fluoride toothpaste.

2.A. Over 6 years old, rinse with 15ml for 1 minute each time. Causes: It is generally recommended for children 6 years and older to rinse their mouths with an appropriate amount of mouthwash to prevent tooth decay, but the exact amount depends on the actual product description.

3.A. Chlorhexidine. Reason: Chlorhexidine has the effect of reducing the number of bacteria in the mouth.

4.A. Chlorhexidine. Why: Chlorhexidine is widely used for its antimicrobial properties, which remove endotoxins from plaque.

5.A. Children aged 3~6 years old should use fluoride toothpaste the size of "pea" each time under the supervision and guidance of parents. Why: Children in this age group need to use an appropriate amount of fluoride toothpaste under adult supervision to prevent excessive intake.

**Chapter 6 Clinical Oral Preventive Techniques**

1.2000U4A1002 Preventive resin filling without one of the following operations ( )

A. Removal of diseased tooth enamel or dentin from pits and fissures

B. Adopt a preventive extension of the hole preparation method

C. Acid etching technology is adopted

D. Fill with resin material

E. Apply a layer of sealant to the occlusal surface

2.2014U4A1004 The most effective prevention method for punctate pit and fissure caries is ( )

A. Educate parents about pits and fissures

B. Regular check-ups, early detection and early treatment

C. Control your diet and brush your teeth before bedtime

D. Use fluoride, fluoride toothpaste, and fluoride water to rinse your mouth

E. Punctate pit and fissure sealant

3.2000U4A1003 The most suitable age for pit and fissure sealing of primary molars is ( )

A. 1~2 years old

B. 3~4 years old

C. 5~6 years old

D. 7~8 years old

E. 9~10 years old

4.2014U4A1005 The correct view about pit and fissure sealing is ( )

A. Baby molars do not do pit and fissure sealing

B. Pit and fissure closure of the first permanent molar

C. Pit and fissure sealant can be done as long as there are pit and fissure

D. Adults do not need to have pit and fissure sealants

E. Anterior teeth with malformed tongue tip do not require pit and fissure sealing

5.2010U4B1136 N-N dihydroxyethyl-p-toluidine is ( )

A. Fissure sealant diluent

B. Pit and fissure sealants, visible light-curing initiators

C. Etching agent prepared with pit and fissure sealant

D. Pit and fissure sealants, self-coagulation and curing initiators

E. Pit and fissure sealant resin matrix

6.2010U4B1137 Bisphenol A-glycidyl methacrylate is ( )

A. Fissure sealant diluent

B. Pit and fissure sealants, visible light-curing initiators

C. Etching agent prepared with pit and fissure sealant

D. Pit and fissure sealants, self-coagulation and curing initiators

E. Pit and fissure sealant resin matrix

7.2010U4B1138 Camphor ester is ( )

A. Fissure sealant diluent

B. Pit and fissure sealants, visible light-curing initiators

C. Etching agent prepared with pit and fissure sealant

D. Pit and fissure sealants, self-coagulation and curing initiators

E. Pit and fissure sealant resin matrix

8.2010U4B1139 Methacrylate is ( )

A. Fissure sealant diluent

B. Pit and fissure sealants, visible light-curing initiators

C. Etching agent prepared with pit and fissure sealant

D. Pit and fissure sealants, self-coagulation and curing initiators

E. Pit and fissure sealant resin matrix

GPT-4

1.E. Apply a layer of sealant to the occlusal surface. Cause: The steps involved in preventive resin filling do not include the application of sealant on the occlusal surface, which is often the practice of pit and fissure sealing.

2.E. Punctate pit and fissure sealant. Causes: Punctate pit and fissure sealant is an effective way to prevent pit and fissure caries, especially in children.

3.C. 5~6 years old. Reason: Deciduous molars are fully erupted when children are about 5~6 years old, which is the ideal time for pit and fissure sealing.

4.C. Pit and fissure sealing can be done as long as there are pits and fissures. Causes: The purpose of pit and fissure sealing is to prevent the occurrence of pit and fissure caries, so any tooth with pit and fissure can be considered for sealing.

5.B. Pit and fissure sealants, visible light curing initiators. Reason: N-N dihydroxyethyl-p-toluidine is commonly used as an initiator in light-curing systems.

6.E. Pit and fissure sealant resin matrix. Cause: Bisphenol A-glycidyl methacrylate is commonly used in the preparation of resin matrices as part of fissure sealants.

7. I'm not sure exactly what camphor ester is used for, but the most likely option is B. Pit and fissure sealants, visible light curing initiators.

8. I'm not sure exactly what methacrylates are used for, but the most likely option is E. Pit and fissure sealant resin matrix.

**Chapter 7 Prevention of Periodontal Disease**

1.2009U4A1002 Commonly used plaque stain is ( )

A. Gentian violet

B. Iodine tincture

C. Neutral red

D. Tetraiodofluorescein sodium

E. C+D

2.2014U4A1006 Which drug is not suitable for long-term use as a method to control plaque and prevent periodontal disease ( )

A. Chlorhexidine

B. Antibiotics

C. Sanguinarine

D. Phenolic compounds

E. Quaternary ammonium compounds

3. The main role of 2014U4A1007 quaternary ammonium compound in plaque control is ( )

A. Interfere with plaque attachment

B. Disruption of plaque matrix

C. Removal of plaque endotoxins

D. Altering the permeability of bacterial cell membranes

E. Control plaque reproduction

4.2016U4A1012 Chlorhexidine antibacterial plaque main effect bacteria ( )

A. Nucleus

B. Cytoplasm

C. Cell membrane

D. Cytoplasm

E. Cell wall

5.2019U4A1025 Adult toothbrush handle length is ( )

A. 120～130mm

B. 140～150mm

C. 155～160mm

D. 160～180mm

E. 180～200mm

6.2019U4A1018 Brushing methods that are easy to learn and master for young children are ( )

A. BASS BRUSHING METHOD

B. Horizontal fluttering brushing method

C. Fones brushing method

D. Smith brushing method

E. Modified Smith brushing method

7.2019U4A1017 The following are not toothpaste ingredients ( )

A. Friction agent

B. Wetting agents

C. Coagulants

D. Adhesives

E. Preservatives

8.2019U4A1016 When chlorhexidine and stannous fluoride are used in combination for plaque control, the correct way to use it is ( )

A. 15-20 minutes after chlorhexidine is applied before stannous fluoride

B. Use stannous fluoride 90 minutes after chlorhexidine application

C. Use stannous fluoride 5 minutes after application of chlorhexidine

D. 6-10 minutes after chlorhexidine is applied before stannous fluoride

E. 30-60 minutes after application of chlorhexidine followed by stannous fluoride

9.2019U4A1015 Simple brushing can remove plaque on average ( )

A. About 40 per cent

B. About 50%.

C. About 60%.

D. About 70%.

E. About 80 per cent

10.2018U4A1021 2010 Global Oral Health Goals: The rate of edentulism at 35~44 years old is ( )

A. Not more than 2 per cent

B. Not more than 4 per cent

C. Not more than 8 per cent

D. Not more than 10 per cent

E. Not more than 12 per cent

11.2020U1A1060 Belonging to the tertiary prevention of periodontal disease is ( )

A. Oral health education and mentoring

B. Oral health education

C. Supragingival cleansing

D. X-rays

Periodontal surgery

GPT-4

1. A. Gentian violet. Causes: Gentian violet is often used for plaque staining to help identify plaque on teeth.
2. B. Antibiotics. Causes: Long-term use of antibiotics may lead to the development of resistance and are therefore not suitable for use as long-term control plaque.
3. D. Altering the permeability of bacterial cell membranes. Cause: Quaternary ammonium compounds control plaque mainly by altering the permeability of bacterial cell membranes.
4. C. Cell membrane. Causes: Chlorhexidine mainly acts on the cell membrane of bacteria, causing it to rupture.
5. B. 140～150mm。 Why: According to conventional designs, adult toothbrush handles are generally between 140 and 150 mm in length.
6. C. Fones brushing method. Why: The Fones brushing method is easy to learn and suitable for young children.
7. C. Coagulants. Reason: Coagulants are usually not included in the composition of toothpaste.
8. E. 30-60 minutes after application of chlorhexidine followed by stannous fluoride. Reason: To avoid chemical interactions, stannous fluoride should be used some time after the use of chlorhexidine.
9. B. About 50%. Why: Brushing alone only removes about 50% of the plaque.
10. B. No more than 4 per cent. Why: The 2010 Global Oral Health Targets aim to keep the rate of edentulism in the 35 to 44 age group to no more than 4 percent.
11. Periodontal surgery. Causes: Tertiary prevention of periodontal disease usually involves the treatment and control of the disease, including periodontal surgery.

**Chapter 8 Self-Oral Health Care Methods**

1.2003U4A1001 The advantages of nylon wire bristles do not include which item ( )

A. The hair ends are rounded, which has a good cleaning effect on teeth and massage on teeth

B. Good elasticity

C. Abrasion resistance

D. Poor water absorption

E. Not easy to curl at high temperatures

2. 2003U4A1002 Aromatics in toothpaste are ( )

A. Peppermint

B. Glycerol

C. Benzoates

D. Silica

E. Sodium lauryl sulfate

3.2002U4A1001 Which brushing method is best ( )

A. Brush lightly

B. Rebrush

C. Brush up and down

D. Brush up and down

E. Horizontal brushing

4.2000U4A1001 Cannot be effectively achieved by brushing teeth ( )

A. Remove tartar and plaque from the neck of the teeth

B. Remove tartar and plaque from the gingival sulcus

C. Remove food debris from the occlusal surface

D. Remove tartar and plaque from the adjacent surfaces of the teeth

E. Massage the gums to enhance the resistance of periodontal tissues

5. 2016U4B1102 Sodium laurate sulfate in toothpaste is ( )

A. Adhesives

B. Wetting agents

C. Preservatives

D. Friction agents

E. Detergents

6.2016U4B1103 Silica in toothpaste is ( )

A. Adhesives

B. Wetting agents

C. Preservatives

D. Friction agents

E. Detergents

GPT-4

1. D. Poor water absorption. Why: Nylon silk bristles have the advantage of being abrasion-resistant and elastic, rather than being less absorbent.
2. A. Peppermint. Why: Peppermint is a common fragrance in toothpastes and is used to provide a fresh taste.
3. E. Horizontal brushing. Cause: Horizontal brushing is not the best brushing method as it can cause gum damage.
4. E. Massage the gums to enhance the resistance of periodontal tissues. Why: Brushing teeth is mainly used to remove tartar and food debris, and has limited effect on massaging the gums and strengthening the resistance of periodontal tissues.
5. E. Detergents. Reason: Sodium laurate sulfate is commonly used as a cleansing agent in toothpaste.
6. D. Friction agents. Why: Silica acts as a friction agent in toothpaste to help remove stains from your teeth.

**Chapter 9 Prevention of Other Oral Diseases**

1.2016U4A1011 The daily smoking volume with a risk of oral cancer of 6.0 is ( )

A. 10~19 sticks

B. 40~49 pcs

C. 1~9 pcs

D. 20~29 pcs

E. 30~39 pcs

2. 2019U4A1019 Optical radiation is the main cause of skin cancer, and the wavelength is more common ( )

A. 200～300nm

B. 320～400nm

C. 320～450nm

D. 350～450nm

E. 350～550nm

3.2019U4A1020 The following epidemiological characteristics of oral cancer, the correct one is ( )

A. The South-East Asia region has the highest prevalence

B. Onset is not temporal

C. There is a tendency to have a lower age of illness

D. The male-to-female ratio is close to 1:1

E. Incidence is similar across ethnicities

4.2019U4A1021 Prevention methods for dental etching are ( )

A. Eat more fruits

B. Chewing acidic medications

C. Eat acidic foods

D. Refusal of chewing gum

E. Reduce the consumption of carbonated beverages

5. 2020U1A1064 is not part of the category of raising public awareness of oral cancer warning signs ( )

A. There are sores in the mouth that have not healed for more than 2 weeks

B. White patches in the mouth

C. Unexplained swelling and swollen lymph nodes in the mouth and neck

D. Unexplained recurrent bleeding in the mouth

E. Odor in the mouth

6.2020U1A1071 Actively prevent malocclusion ( )

A. Infants

B. Young children

C. School-age children

D. Young adults

E. Older persons

GPT-4

1. B. 40~49 pcs. Causes: Smoking between 40 and 49 cigarettes per day is thought to increase the risk of oral cancer.
2. B. 320～400nm。 Cause: Ultraviolet rays (320~400nm) in the UVB spectrum range are the main cause of skin cancer.
3. A. The South-East Asia region has the highest prevalence. Causes: Southeast Asia has a higher incidence of oral cancer.
4. E. Reduce the consumption of carbonated beverages. Causes: Reducing your intake of acidic foods and beverages, such as carbonated drinks, can prevent dental erosion.
5. E. Odor in the mouth. Causes: Bad taste in the mouth is not a direct warning sign of oral cancer, but is more associated with other oral problems.
6. C. School-age children. Causes: Aggressive prevention and treatment of malocclusion is usually done in school-age children so that they can be corrected in time.

**Chapter 10 Oral Health Care for Specific Populations**

1.2019U4B1111 The suitable population for pit and fissure sealing of the first permanent molars is ( )

A. Infants

B. Young children

C. Older persons

D. Middle-aged

E. Preschool

2. 2019U4B1112 The population susceptible to root caries is ( )

A. Infants

B. Young children

C. Older persons

D. Middle-aged

E. Preschool

3.2019U4B1113 The first oral examination should be done in ( )

A. Infants

B. Young children

C. Older persons

D. Middle-aged

E. Preschool

4.2020U1A1065 People exposed to acidic working environments are susceptible ( )

A. Acid erosion

B. Caries disease

C. Osteoporosis

D. Periodontal disease

E. Dental fluorosis

5.2020U1A1070 Before going to bed, the mother's fingers should be wrapped with clean gauze or the gums and palate should be scrubbed with latex finger sleeves ( )

A. Infants

B. Young children

C. School-age children

D. Young adults

E. Older persons

6.2020U1A1072 THE ROLE OF THE MOUTHGUARD ( )

A. Protect the teeth and other tissues in the mouth

B. Prevent jaw fractures

C. Reduce the likelihood of concussion

D. Enhance athletes' sense of safety

E. All of the above

7.2020U1A1075 The appropriate period for the treatment of oral diseases in pregnant women is ( )

A. 1~3 months

B. 3~6 months

C. 4~6 months

D. 5~7 months

E. 3~5 months

GPT-4

B. Young children. Causes: First permanent molar pit and fissure closure is usually recommended in early childhood because early childhood is the period when permanent teeth begin to erupt.

C. Older persons. Causes: Older people are prone to root caries due to gum recession, exposing more of the root surface of the tooth.

E. Preschool. Causes: A child's first oral examination should be done in the preschool years to detect and prevent oral problems early.

A. Acid erosion. Cause: Acidic work environments increase the risk of acid erosion because acids can corrode teeth.

A. Infants. Reason: Babies need their mother's help cleaning their mouths because they can't do it on their own.

E. All of the above. Why: Mouthguards protect teeth and other tissues in the mouth, prevent jaw fractures, and may reduce the likelihood of concussion while enhancing an athlete's sense of safety.

C. 4~6 months. Reason: The second trimester of pregnancy (4~6 months) is a relatively safe period for oral treatment.

**Chapter 11 Oral Health Promotion**

1.2013U4A1001 The decisive role in oral health promotion is ( )

A. Public

B. Dental Defense Guidance Group

C. Health administrative leadership

D. Dental health workers

E. Faculty of Stomatology

2.2014U4A1011 The methods of oral health education are generally ( )

A. Individual conversations, online exchanges, mass communication channels, organization of community events

B. Individual conversations, small seminars, mass communication channels, organization of community events

C. Individual conversations, small seminars, school lessons, and community activities

D. Expert consultations, mini-seminars, mass communication channels, organization of community events

E. Expert consultation, networking, mass communication channels, organization of community events

3.2020U1A1073 The core components of oral health promotion are ( )

A. Oral health education

B. Oral Health Protection

C. Oral disease prevention

D. Primary prevention of oral diseases

E. Community oral health care

4.2020U1A1074 Oral health education methods with large coverage and quick ability to attract public attention and focus on oral health problems to be solved are ( )

Mass media

B. Community activities

C. Mini-seminars

D. Individual conversations

E. Expert seminars

GPT-4

D. Dental health workers. Why: Dental health workers play a decisive role in oral health promotion because they are directly involved in oral health assessment, treatment, and education.

B. Individual conversations, small seminars, mass communication channels, organization of community events. Why: These methods contribute to educational purposes by combining direct communication with individuals and extensive public education.

A. Oral health education. Why: Oral health education is a core component of oral health promotion because it provides the knowledge and skills to prevent oral diseases.

Mass media. Why: Mass media can reach a large number of people's attention and quickly attract the public's attention to focus on oral health issues.

**Chapter 14 Evidence-Based Dentistry and Its Applications**

1.2014U4A1008 Vinyl gloves are often used under what circumstances ( )

A. Surgery

B. Inspection

C. Hand allergies

D. Cleaning the instrument

E. Teeth cleaning

2.1999U4A1004 Mobile phone disinfection can not use which of the following methods ( )

A. Formaldehyde vapor fumigation

B. Autoclaving

C. Ultraviolet disinfection

D. Wipe with 2% iodine tincture and then wipe with 75% alcohol

E. Soak in 0.1% Xinjieer solution

3.2014U4A1009 The ideal immersion solution for pre-cleaning is ( )

A. 1:16 glutaroxonol solution

B. 8%~10% sodium hypochlorite solution

C. 1:213 iodophor

D. 75% ethanol

E. 1:32 synthetic phenol solution

4.2014U4A1010 The first step of instrument pre-cleaning is ( )

A. Rinse

B. Chemical immersion

C. Wipe

D. Drying

E. Docking

5. The time required to kill HBV at 95°C at 2001U4B1099 is ( )

A. 1 minute

B. 2 minutes

C. 3 min

D. 4 minutes

E. 5 minutes

6. 2001U4A1001 The diameter of the droplet when propagating is ( )

A. >2μm

B. >5μm

C. >10μm

D. >15μm

E. >20μm

7. 2001U4B1100 HBV is a heat-resistant virus that takes several minutes to kill at 95°C ( )

A. 1 minute

B. 2 minutes

C. 5 min

D. 10 minutes

E. 15 minutes

8.2019U4A1024 In oral health care, the main microorganisms transmitted by contact are ( )

A. Hepatitis C virus

B. Chickenpox virus

C. Measles virus

D. Rubella virus

E. Mumps virus

9.2019U4A1023 Diseases that are mainly transmitted by air are ( )

A. Adenovirus

B. AIDS

C. Syphilis

D. Viral hepatitis

E. Herpes

10.2019U4A1022 Gingival drain belongs to ( )

A. High-Risk Devices

B. Intermediate-risk devices

C. Low-risk devices

D. Medium-level sterilized instruments

E. High-level sterilization of instruments

11.2018U4A1022 Diseases transmitted mainly by contact are ( )

A. Herpes

B. Tuberculosis

C. Measles

D. Chickenpox

E. Rubella

GPT-4

1. C. Hand allergies. Why: Vinyl gloves are often used in people who have an allergic reaction to latex in case of hand allergies.
2. E. Soak in 0.1% Xinjieer solution. Reason: Mobile phone disinfection is usually not soaked with Xinjieer Disinfectant as it can be harmful to the device.
3. B. 8%~10% sodium hypochlorite solution. Why: Sodium hypochlorite solutions are commonly used in the pre-cleaning of medical devices due to their effectiveness in removing proteins and organics.
4. A. Rinse. Why: The first step in pre-cleaning instruments is rinsing to remove most of the dirt and organics.
5. B. 2 minutes. Reason: It is generally believed that 2 minutes at 95°C is sufficient to kill HBV.
6. B. >5μm。 Causes: Droplets are generally considered to be larger than 5 microns in diameter.
7. B. 2 minutes. Cause: 2 minutes at 95°C is usually sufficient to kill heat-resistant HBV.
8. A. Hepatitis C virus. Causes: In oral health care, the predominantly contact-transmitted microorganisms are usually blood-borne viruses, such as hepatitis C virus.
9. E. Herpes. Causes: Herpes is mainly spread through the air, especially through droplets.
10. A. High-Risk Devices. Why: Gingival drains are used for subgingival procedures and are high-risk devices because they can come into contact with blood.
11. D. Chickenpox. Causes: Chickenpox is spread mainly by contact, especially through contact with herpes fluid.
